# Rapid Peptide Cyclization Inspired by the Modular Logic of Nonribosomal Peptide Synthetases

**DOI:** 10.1021/jacs.4c04711

**Published:** 2024-06-06

**Authors:** Yaoyu Ding, Edward Lambden, Jessica Peate, Lewis J. Picken, Thomas W. Rees, Gustavo Perez-Ortiz, Sophie A. Newgas, Lucy A. R. Spicer, Thomas Hicks, Jeannine Hess, Martin B. Ulmschneider, Manuel M. Müller, Sarah M. Barry

**Affiliations:** † Department of Chemistry, Faculty of Natural, Mathematical, and Engineering Sciences, King’s College London, Britannia House, 7 Trinity Street, London SE1 1DB, U.K.; ‡ The Francis Crick Institute, 1 Midland Road, London NW1 1AT, U.K.

## Abstract

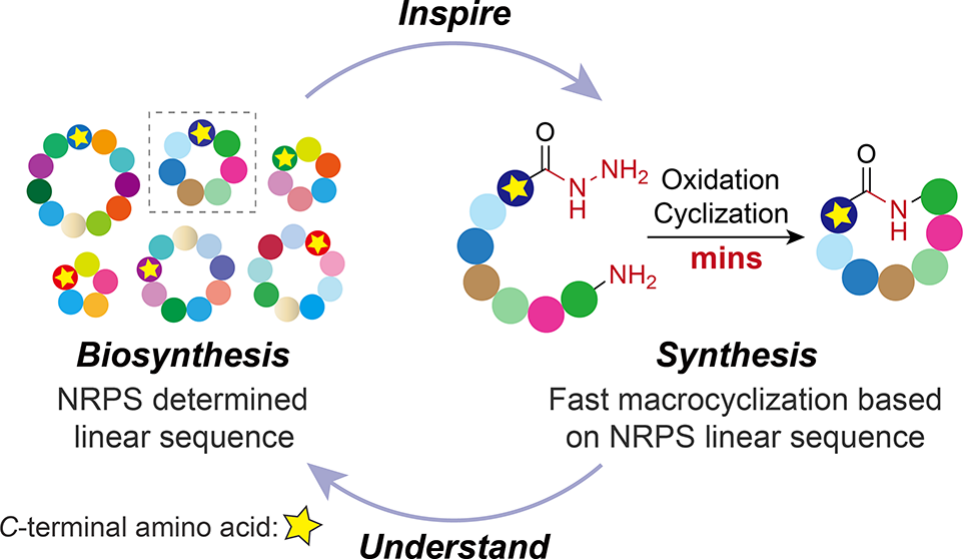

Nonribosomal cyclic peptides (NRcPs) are structurally complex natural products and a vital pool of therapeutics, particularly antibiotics. Their structural diversity arises from the ability of the multidomain enzyme assembly lines, nonribosomal peptide synthetases (NRPSs), to utilize bespoke nonproteinogenic amino acids, modify the linear peptide during elongation, and catalyze an array of cyclization modes, e.g., head to tail, side chain to tail. The study and drug development of NRcPs are often limited by a lack of easy synthetic access to NRcPs and their analogues, with selective macrolactamization being a major bottleneck. Herein, we report a generally applicable chemical macrocyclization method of unprecedented speed and selectivity. Inspired by biosynthetic cyclization, it combines the deprotected linear biosynthetic precursor peptide sequence with a highly reactive *C*-terminus to produce NRcPs and analogues in minutes. The method was applied to several NRcPs of varying sequences, ring sizes, and cyclization modes including rufomycin, colistin, and gramicidin S with comparable success. We thus demonstrate that the linear order of modules in NRPS enzymes that determines peptide sequence encodes the key structural information to produce peptides conformationally biased toward macrocyclization. To fully exploit this conformational bias synthetically, a highly reactive *C*-terminal acyl azide is also required, alongside carefully balanced pH and solvent conditions. This allows for consistent, facile cyclization of exceptional speed, selectivity, and atom efficiency. This exciting macrolactamization method represents a new enabling technology for the biosynthetic study of NRcPs and their development as therapeutics.

## Introduction

**Figure 1:**
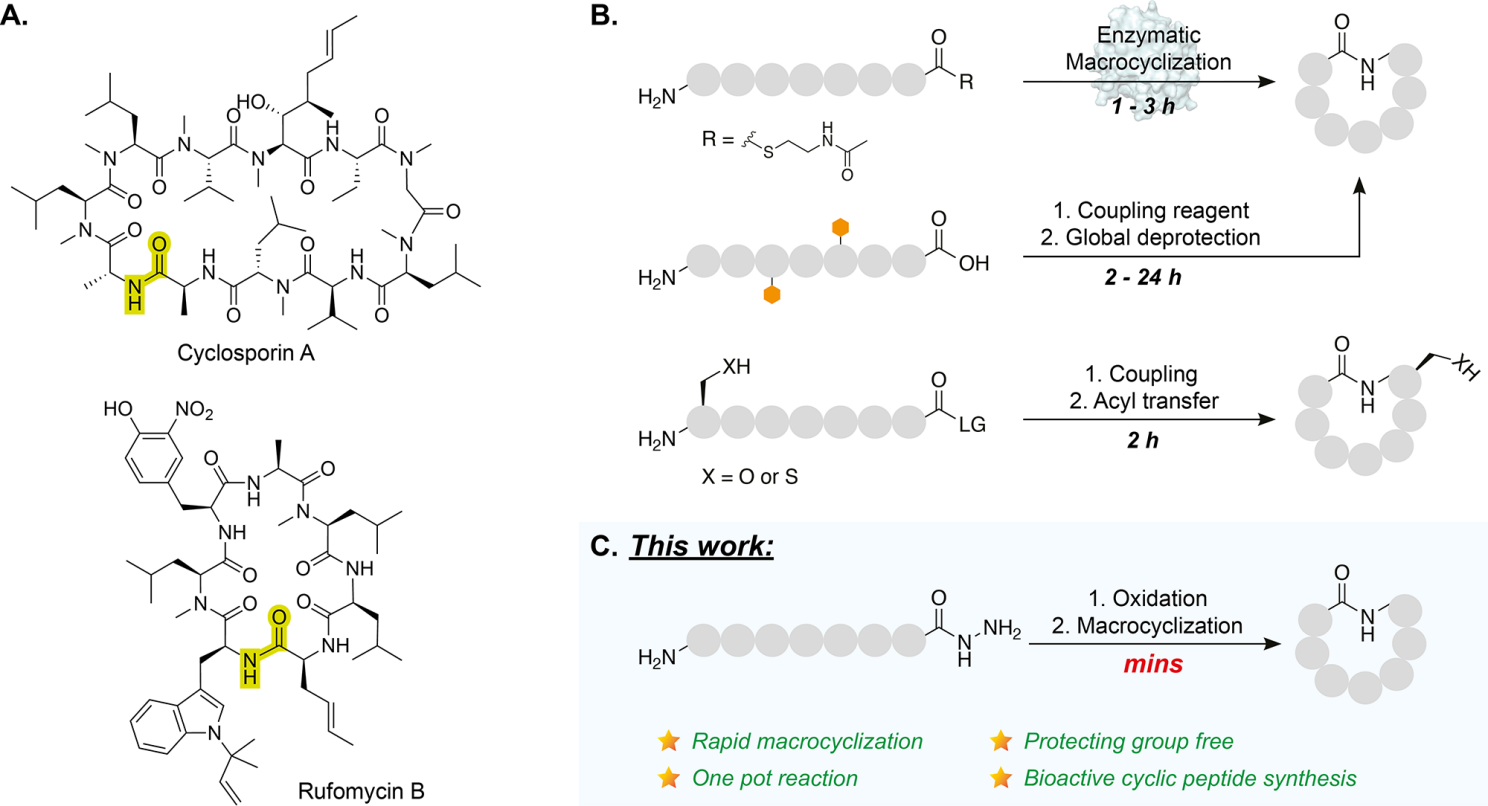
Bioactive nonribosomal cyclic peptide cyclization strategies. (A) Representative bioactive head-to-tail NRcPs. (B) Gray circles represent any amino acid. Top: enzymatic cyclization strategy utilizing thioester-linked peptides that mimic the peptide bound to the NRPS carrier protein via phosphopantetheine (pPant). R = *N*-acetylcysteamine, a typical pPant mimic used in *in vitro* TE domain-catalyzed cyclization. Middle: standard chemical peptide cyclization using coupling reagents, and orange hexagons represent protecting groups. Bottom: chemo-selective peptide cyclization via ligation strategies, which require specific amino acids, e.g., cysteine or serine, in the sequence. (C) This work: rapid, one-pot peptide cyclization was performed from a fully deprotected peptide hydrazide via an acyl azide. For NRcPs, the linear peptide sequence is equivalent to that of the NRPS biosynthetic precursor.

Natural product cyclic peptides are a vital source of pharmaceuticals, especially antibiotics.^1,2^ Their cyclic structure improves protease resistance and imposes a restricted conformation, providing a defined surface to interact with a target (Figure 1A).^3,4^ Natural product peptides are biosynthesized via two different routes, ribosomal and nonribosomal. Ribosomal peptides are genetically encoded, posttranslationally modified, and then cyclized by pathway-specific cyclases.^5^ Nonribosomal cyclic peptide (NRcP) biosynthesis employs modular multidomain enzymes known as nonribosomal peptide synthetases (NRPSs) that act as assembly lines in which each module is responsible for sequentially adding a new amino acid to the covalently bound linear peptide.^6,7^ The staggering structural diversity of NRcPs (e.g., rufomycin, cyclosporin A; Figure 1A) is due to the ability of NRPS enzymes to utilize nonproteinogenic amino acids and incorporate modifications during peptide elongation, e.g., *N*-methylation, epimerization, and oxidation.^8^ Macrocyclization, usually via amide or ester formation, is typically catalyzed by a *C*-terminal type I thioesterase (TE) domain in the NRPS. NRcPs have long been a vital source of antibiotics, and as antimicrobial resistance continues to grow as a global health crisis, facile methods to generate derivatives of these highly complex molecules are required.^1,2^ While the chemical synthesis of linear peptides is straightforward, regio- and chemo-selective peptide macrocyclization remains a frustrating synthetic challenge^9^ that, in the case of NRcPs, is exacerbated by structural complexity.

The total synthesis of NRcPs typically employs standard coupling reagents (e.g., rufomycin,^10,11^ cyclosporine,^12^ cyclomarin,^13^ and surugamide B^14^). However, this approach requires extensive protecting group chemistry and long reaction times and often produces side reactions such as epimerization and dimerization, reducing the overall yield. Recently, alternative chemo-selective ligation strategies have been developed (Figure 1B)^15^ as applied to daptomycin^16^ and murepavadin.^17^ However, these methods require a specific amino acid (e.g., serine or cysteine) in the sequence and thus are not generally applicable to NRcP synthesis. Biocatalytic approaches have resulted in the characterization of several natural product cyclases, including TE domains, for peptide cyclization of natural product peptide derivatives (Figure 1B).^18−23^ This route to natural product analogues is promising, but without engineering, these enzymes allow for limited structural diversity and are generally pathway-specific.

Thus, using rufomycin B as a model target, we aimed to develop a general approach to NRcP synthesis and macrocyclization regardless of sequence or ring size and in the process gain further insight into NRcP biosynthesis. Thus, inspired by NRPS biosynthetic cyclization and the nature of the linear substrate in TE-catalyzed cyclization, we report the development of an operationally simple and high yielding chemical macrocyclization strategy of unprecedented speed (minutes) and selectivity (Figure 1C). We utilize highly reactive *C*-terminal acyl azide peptides and mixed aqueous/organic solvent systems to synthesize a range of NRcPs and analogues of various ring sizes and cyclization modes, e.g., head to tail and side chain to tail. This has resulted in regioselective macrolactamization even in the presence of competing side chains. Our experimental and *in silico* data demonstrate that reaction success relies on a combination of factors including *C*-terminus reactivity, the correct solvent environment, and, crucially, peptide conformation. We propose that a procyclization conformation is dictated by the modular assembly line logic of NRPS biosynthesis including the placement of backbone modifications, e.g., *N*-methylation or turn-inducing residues, e.g., *L*-Pro, biasing the peptide toward selective cyclization. We also successfully applied the method to other cyclic peptides (synthetic or of unknown biosynthetic origin) to demonstrate its broader applicability to peptide cyclization. This work represents a step change in our ability to generate NRcPs for both biosynthetic studies and drug development.

## Results and Discussion

### Bioinspired Rapid Chemical Macrocyclization: *C*- and *N*-Terminal Activations

Rufomycins (also known as ilamycins) are a family of nonribosomal cyclic heptapeptides produced by several strains of the bacterium *Streptomyces atratus* (Figure 1).^24,25^ They have garnered interest as potential anticancer agents^26^ but most significantly as antibacterial agents against *Mycobacterium tuberculosis* (lowest reported minimum inhibitory concentration (MIC) 10 nM).^27−30^ This is of importance due to the global increase of antibiotic-resistant TB infections and the urgent need for new treatments.^31^ However, rufomycins are produced in low titers and as mixtures of derivatives in the bacterial culture, a factor that complicates their study and development as drugs. Additionally, during our own biosynthetic investigations of rufomycins,^32^ we found that the isolation and purification of small amounts of rufomycins limited our enzymatic studies. Thus, using rufomycins as a model system, we aimed to tackle this typical problem in the field by developing a robust macrocyclization method to enable NRcP synthesis while also shedding light on biosynthetic cyclization.

**Figure 2:**
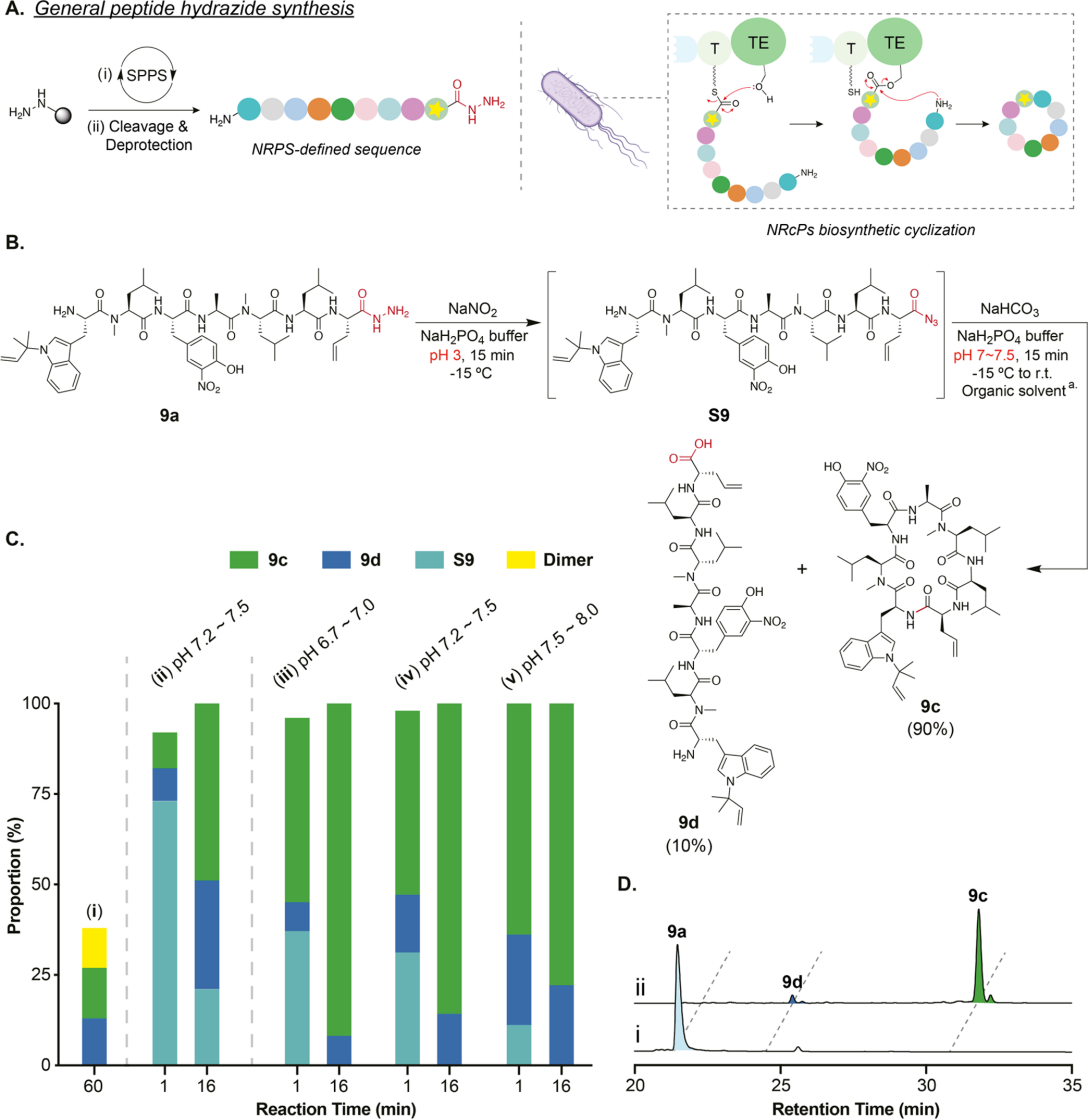
Development and optimization of macrocyclization methodology. (A) Left: synthesis of linear peptide hydrazide followed by the biosynthetic linear peptide sequence. SPPS: coupling: amino acid (4 equiv), HATU (3.9 equiv), DIPEA (8 equiv), DMF, 45 min, r.t. Fmoc deprotection: 20% piperidine in DMF, 15 min, r.t. cleavage: 1% TFA in DCM, 1 h, r.t. or 95% TFA 2.5% H_2_O and 2.5% TIS, 2 h, r.t. Right: mechanism of NRPS-TE domain-catalyzed peptide cyclization (*T* = thiolation domain also known as peptidyl carrier protein (PCP)). (B) Bioinspired one-pot chemical macrocyclization of acyl hydrazide peptide **9a** (2.2 mM). ^a^Organic solvent is MeCN, buffer (50 mM NaH_2_PO_4_, 6 M GdmCl, 1.5 mM EDTA). (C) pH and solvent dependence of bioinspired macrocyclization compared to initial silver-assisted macrolactamization (Figure S13). (i) Silver-assisted cyclization of SNAC-thioester derivative **9b**, AgOTfa, NaOAc, DMSO, pH 7.5, 1 h, r. t.; (ii) Bioinspired macrocyclization reaction in the absence of MeCN, (iii–v) Bioinspired macrocyclization containing 30% v/v MeCN. (D) HPLC analysis (355 nm) of bioinspired one-pot cyclization. (i) Purified starting material, hydrazide peptide **9a**. (ii) Crude reaction mixture 16 min after addition of NaHCO_3_ (neutralizing oxidation reaction to ∼pH 7). Acyl azide intermediate **S9** was fully converted within 16 min. An additional peak with the same mass as **9c** likely results from *C*-terminal epimerization (epimerization is observed as 0–10% conversion leading to d.e. 80–99%, depending on reaction conditions). The results are reproducible, giving 90 ± 2% conversion over 3 replicates.

The biosynthetic linear precursor of NRcPs is dictated by the order of modules in the NRPS as can be seen for rufomycin biosynthesis (Figure S10).^33^ During NRPS-catalyzed peptide elongation, the peptide is covalently bound as a thioester to the thiolation domain via a phosphopantetheinyl group^34^ and then transferred to a catalytic serine in the TE domain to form an acyl enzyme intermediate (Figure 2A). Our synthetic starting point was therefore to mimic the NRPS-bound peptide using an *N*-acetylcysteamine thioester (SNAC) peptide typically used as substrate mimics to study TE domains *in vitro* (Figures 1B and S10).^35,36^ To develop a robust one-pot synthesis for SNAC-rufomycin analogue **9b**, avoiding epimerization, we employed the peptide acyl hydrazide strategy used to form thioesters in native chemical ligation (NCL).^37−39^ The nonproteinogenic amino acid, *N*-prenyl-*L*-tryptophan, was synthesized and protected using a published methodology (Figure S1).^40,41^ The linear peptide precursor **9a**, containing a *C*-terminal acyl hydrazide, was synthesized via Fmoc solid-phase peptide synthesis (SPPS) and oxidized after mild acidic cleavage and deprotection to the acyl azide peptide **S9**.^42^ Addition of NAC at low temperature afforded SNAC peptide **9b** within minutes and without detectable epimerization. To determine if the peptide could be chemically cyclized, we employed silver-assisted thioester macrocyclization (Figure S13).^43,44^ HPLC analysis after 1 h at room temperature showed that while incomplete, the reaction had successfully resulted in cyclization (14%) alongside hydrolysis (13%) and dimerization (11%) (Figures 2Ci and S13). This result indicated that the thioester peptide was capable of cyclization. Previous reports have shown that cyclization can be promoted using the biosynthetic linear precursor variously activated at the *C*-terminus via, for example, 2-formylthiophenol,^45^ aryl thioester,^46^ and *o*-aminoanilide linker.^47^ Aryl thioesters have also been employed for peptide cyclization via native chemical ligation (NCL) chemistry (Figure 1B).^48^

However, the TE domain likely creates a more reactive *C*-terminus than a thioester (Figure 2A). TE domains activate the acyl enzyme intermediate via coordination of the ester carbonyl and subsequent stabilization of the tetrahedral intermediate, in a so-called oxy-anion hole, via, e.g., hydrogen bonding to protein backbone amide hydrogens.^49^ We therefore explored the possibility that the highly reactive *C*-terminal acyl azides might be used directly for cyclization via intramolecular aminolysis. Acyl azides have been used directly or as intermediates to synthesize heterocycles, amide bonds,^50^ and macrocycles.^51^ However, their instability and propensity to undergo the Curtius rearrangement means that, with the exception of NCL,^52^ they are not used extensively. Thus, we proposed to generate the acyl azide precursor at low temperature, and instead of adding the thiol nucleophile, the pH would be increased using NaHCO_3_. This would partially deprotonate the *N*-terminal amine to facilitate macrocyclization (Figure 2B). This approach relies heavily on the linear peptide adopting a conformation favorable to cyclization to ensure nucleophilic attack is faster than acyl azide rearrangement or hydrolysis.^53,54^

To test this hypothesis, the HPLC-purified acyl hydrazide peptide **9a** (2.2 mM) was oxidized with NaNO_2_ at pH 3 in the presence of 6 M GdmCl to generate the highly reactive acyl azide peptide intermediate **S9***in situ* (Figure 2B). Upon increasing the pH to 7–7.5, the peptide underwent cyclization (63%) or hydrolysis (37%) within 16 min, as judged by HPLC and MS analyses (Figures 2Cii, S14A, and S15A). Some epimer was detected (∼3%), but no dimerization was observed (Figure S14A). The remarkable speed and efficiency of these initial reaction conditions prompted us to optimize the reaction to further improve the ratio of cyclic to hydrolyzed products.

The reaction buffer is similar to that used in NCL and thus contains 6 M GdmCl to solubilize and denature peptides and proteins.^55^ We surmised that these conditions might negatively impact peptide procyclization conformation resulting in acyl azide **S9** hydrolysis. Attempts to reduce the GdmCl concentration in the buffer to 3 M resulted in frozen solutions at −15 °C (Figure S17A). Diluting the reaction with water during the neutralization step resulted in acyl azide **S9** precipitation, as did replacing GdmCl with 6 M urea (Figures S14B and S17B). A recent crystal structure of the TE domain responsible for valinomycin cyclization revealed a combination of polar and hydrophobic interactions with the truncated peptide substrate mimic (PDB: 6ECE).^56^ Inspired by this, we tested whether increasing the hydrophobicity of the reaction solution would be beneficial for chemical cyclization. Gratifyingly, diluting the reaction with 50% acetonitrile in water (2 vol), thereby reducing the concentration of GdmCl and increasing hydrophobicity, significantly improved conversion to the cyclic peptide (90%) (Figure S14D).

The effect of pH on the reaction was also evaluated from 6.7 to 8.0 (Figures 2C, S14D–F, and S15B–D). Neutralizing the reaction to pH 7 with the addition of solvent was found to give optimal conversion to cyclic peptide **9c** (90%) after just 16 min while minimizing hydrolysis to peptide **9d** (<10%) (Figure 2Ciii,D).

As metal ions (e.g., Cu^II^, Ag^II^) can template cyclization, we considered whether metal contamination was a contributing factor to reaction success.^57^ The reaction buffer already contained the chelating agent EDTA (1.5 mM). Removing EDTA or increasing EDTA concentration (15 mM) had no effect on the reaction outcome (Figure S15E).

Collectively, these results demonstrate that direct intramolecular aminolysis of *C*-terminal acyl azides proceeds rapidly upon “activation” of the *N*-terminus by neutralization at pH ∼ 7 and the addition of cosolvent.

### Rapid Biphasic Bioinspired Macrocyclization

**Figure 3:**
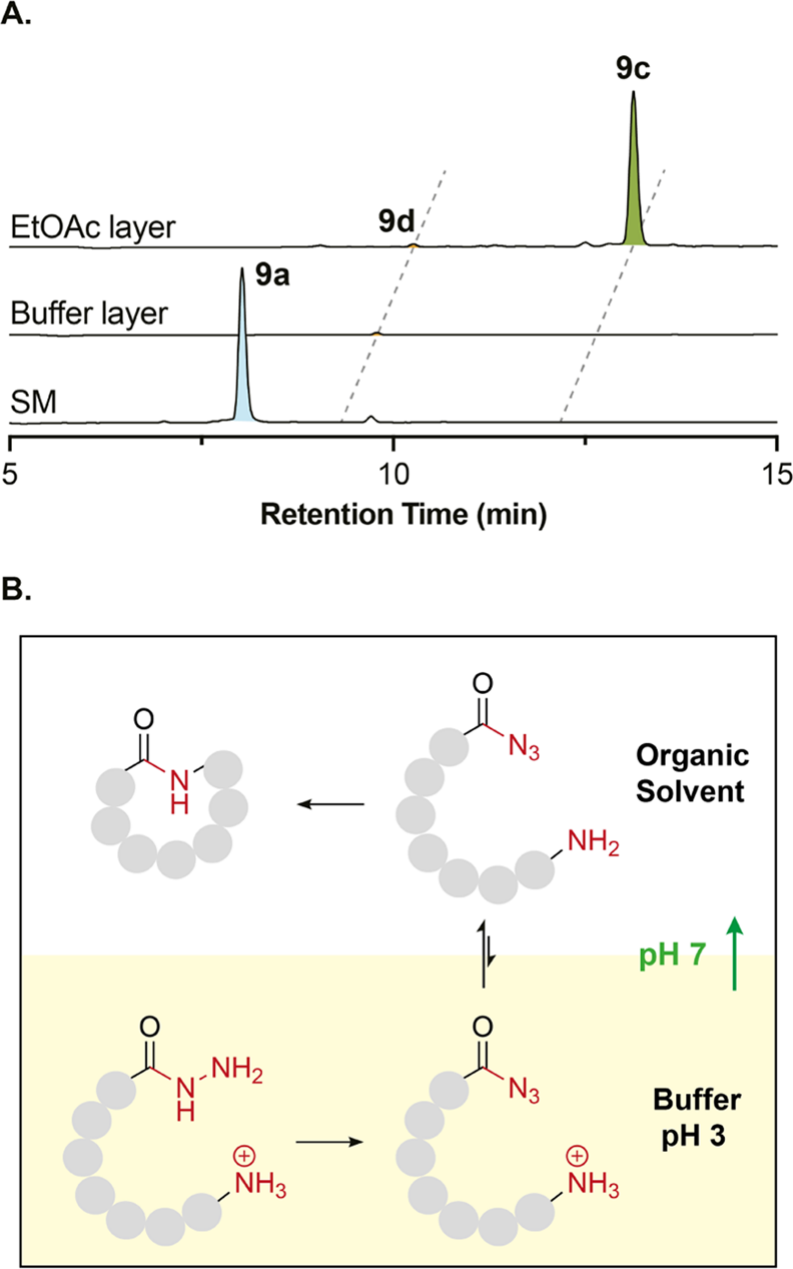
Biphasic macrocyclization. (A) HPLC (monitored at 355 nm) analysis of peptide **9a** in an analytical scale cyclization (2 mM, 1 mL) using ethyl acetate (1 mL) as cosolvent. SM = starting material. (B) Diagram illustrating the proposed role of pH and organic solvent in facilitating cyclization. No epimerization or dimerization is observed under these conditions.

Our study furnished us with a macrocyclization strategy of unprecedented speed. To facilitate scaleup, we reasoned that a biphasic mixture would promote cyclization and simultaneously enable purification by separating the product from aqueous salts (e.g., GdmCl). Purified hydrazide peptide **9a** was used to test this theory on a small scale. Following oxidation and neutralization of the reaction to pH 7, ethyl acetate (1 vol) or MeCN (2 vol) was added to the reaction vial to create two phases (Figure S14H). The reaction was initially maintained at −15 °C to minimize side reactions (e.g., Curtius rearrangement and hydrolysis) and then allowed to warm up slowly. After 16 min, HPLC analysis of the concentrated crude organic phase indicated complete consumption of the acyl azide and the production of the cyclic peptide in excellent yield (95%). The hydrolyzed peptide was detected as a minor product (<5%), but no epimerization or dimerization was observed (Figures 3A and S14C,G). Using relatively low boiling point solvents such as ethyl acetate or acetonitrile as cosolvents provides an additional advantage over other peptide cyclization methods that commonly use highly boiling DMF or NMP.

The speed and selectivity of the cyclization over dimerization can be rationalized by the preorganization of the peptide in solution and the reactivity of the acyl azide at the *C*-terminus. However, it does not fully explain the exceptional rate of this reaction over previously reported macrocyclization methods that typically require hours, even for enzyme-catalyzed processes (Figure 1B). The organic phase also appears to play a role in the reaction even when miscible with water. We propose that the presence of organic solvent in the cyclization step provides both a hydrophobic environment and shifts the reaction equilibrium in a process analogous to the Schotten–Baumann conditions.^58,59^ Thus, on changing the pH to 7, a fraction of the linear peptide becomes neutral via deprotonation of the terminal amine (estimated p*K*_aH_ of the *N*-terminal amine of peptide **9a** is 7.3, molnetworks in Chem3D) (Figure 3B).^60^ This uncharged peptide partitions into the organic phase, where isolated from GdmCl, it cyclizes quickly by adopting a preferred conformation dictated by backbone methylation. This process also further dilutes the peptide, reducing the possibility of a dimer formation. Irreversible cyclization in turn shifts the equilibrium of amine protonation, pulling more peptides into the organic phase. The only case in which dimer formation was observed was due to insufficient mixing of the two phases during a scaled-up reaction of peptide **18a** (8 mM, 40 mL reaction buffer) (Figure S30C).

### Macrocyclization: Influence of *N*-Methylation on Rufomycin Linear Peptide Conformation

**Figure 4:**
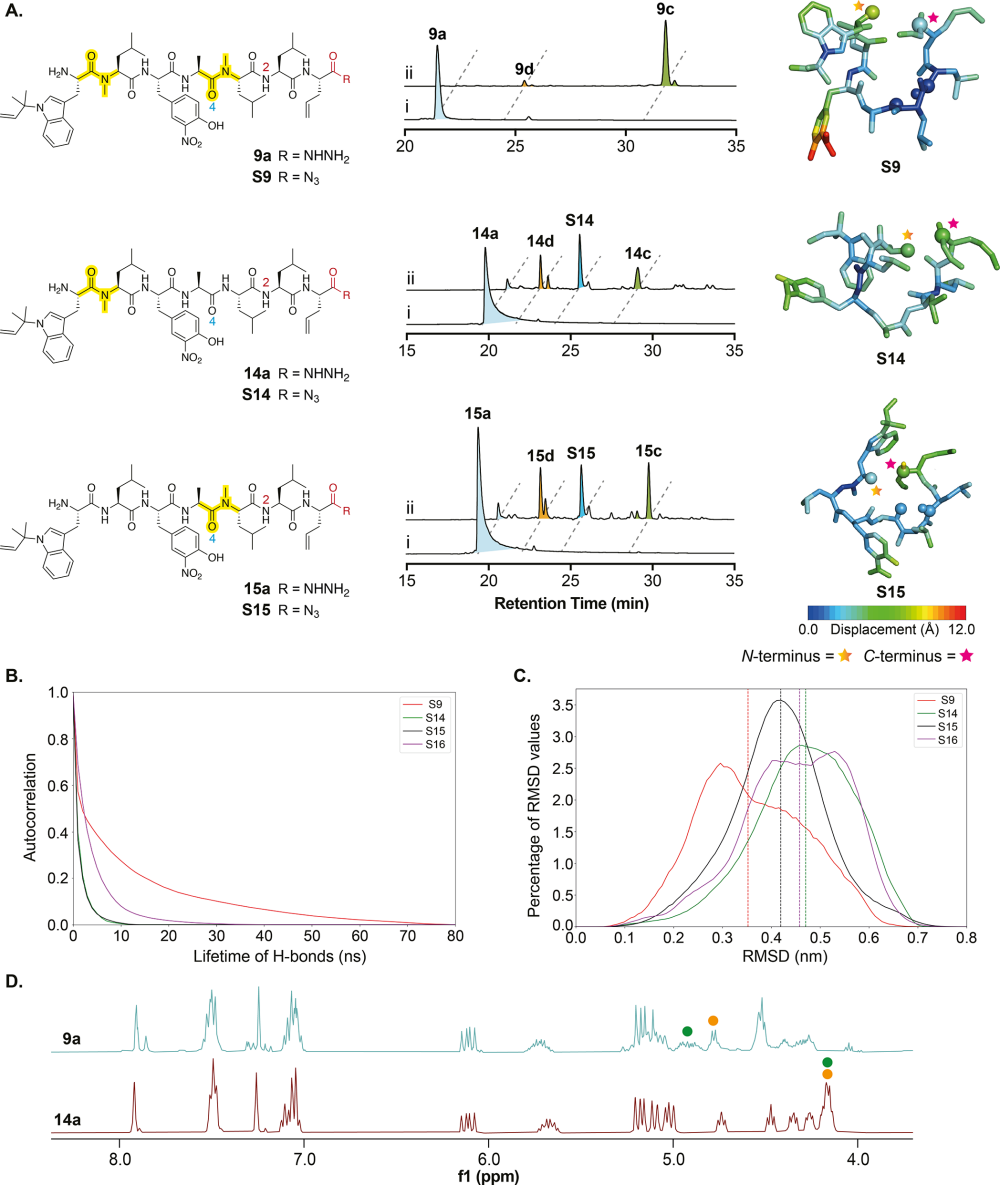
Investigating the role of backbone methylation on macrocyclization. (A) Left: chemical structures of linear peptides. Middle: HPLC analysis (355 nm) of cyclization, in each chromatogram, trace i is the starting material, trace **ii** is the cyclization reaction mixture 16 min after neutralizing to ∼pH 7. Right: representative cyclizing structures for acyl azide peptides **S9**, **S14**, and **S15**. A histogram of the deviation from this set of positions was calculated for each atom in the peptide, and the atoms are colored according to the median value of this histogram. (B) Survival probability for intrapeptide hydrogen bonding for four acyl azide peptides in solvent. Peptide **S16** (which could not be synthesized) has had both backbone *N*-methyl groups removed. H-bond interactions were calculated with an upper limit of 3.3 Å. (C) Distributions of the root-mean-square deviation (RMSD) of the atomic position for each system over the full trajectory were calculated using the gmx cluster. The median for each distribution is indicated by a dashed line. The fastest cyclizing peptide, **S9**, shows the lowest median RMSD value. (D) Stacked ^1^H NMR of linear acyl hydrazide peptides **9a** and **14a** in 10% D_2_O in *d*_3_-acetonitrile, 400 MHz (full spectra in the Supporting Information). The α-proton region (4.0–5.2 ppm) shows line broadening in **9a**. The aromatic region (7.0–8.0 ppm) indicates the presence of multiple conformations for **9a**. The greatest differences in the chemical shift between **9a** and **14a** are for α protons of *L*-Ala (orange) and *N*-Me-*L*-Leu in **9a** or *L*-Leu in **14a** (green) (Scheme S1).

The rapid rate and high conversions of linear peptide to macrocycle, as well as the absence of dimer formation, clearly indicates that the linear rufomycin precursor preorganizes in solution in a conformation that facilitates macrocyclization. We thus investigated the impact of peptide conformational flexibility, specifically the importance of the backbone *N*-methylation. Rufomycin contains two *N*-methylated *L*-leucine residues (Figure 1A). Backbone amide methylation is a common modification of nonribosomal peptides and is catalyzed by methylation domains in specific modules within the NRPS.^8^ The backbone *N*-methylation pattern is based on domain organization and is thus highly predictable (Figure S10). *N*-methylation of amides reduces the energy barrier between *cis* and *trans* amide isomers, allowing the peptide to form turns, facilitating macrocyclization. In addition, the conformation can be impacted by steric interference between *N*-methyl groups and side chains. To demonstrate the importance of *N*-methylation in this rapid chemical cyclization, peptides **14a** and **15a** were synthesized where one *N*-methyl-*L*-leucine in the sequence was replaced by *L*-leucine (Figure 4A, left). Unfortunately, hydrazide peptide **16a**, lacking both backbone methyl groups, was highly insoluble (Figure S16E). This result serendipitously demonstrated the importance of these backbone modifications to peptide solubility.^61^

Peptides **14a** and **15a** were thus assayed to determine the impact of *N*-methylation on cyclization efficiency. Both reactions resulted in poor conversion compared to that of **9a**. Hydrolysis, forming **14d** and **15d**, and probable epimerization are significant side reactions (Figure 4A, middle). Removing just one methyl group from the peptide backbone also dramatically slows the rate of macrocyclization, allowing detection of the acyl azide intermediate (**S14** and **S15**) after 16 min (Figure 4A, middle).

To explain these results, we aimed to understand how *N*-methylation impacts the linear peptide structure and thus cyclization. First, CD spectra of **9a** and **14a** were measured in 10% H_2_O in MeCN, chosen to mimic reaction conditions while maintaining solubility, and compared to those of cyclic peptide **9c** (Scheme S1D). The data indicate that while **9a** is more ordered than **14a**, it does not contain the same rigid secondary structure as cyclized peptide **9c** (Scheme S1D). ^1^H NMR in the same solvent system, shows that **9a** appears as multiple conformers as observed for the corresponding cyclic peptide **9c** and natural product rufomycin B **8c**. The peaks are also broadened, indicative of a slow exchange (Figure 4D). By contrast, the spectrum of **14a** does not indicate multiple conformers and the peaks are well resolved, indicating fast exchange. 2D experiments (e.g., ROESY) to further investigate the structures were not fruitful as it was not possible to fully resolve the spectra of **9a** due to the additional exchange peaks. Peptide **14a** formed a gel in MeCN/D_2_O over time, complicating the data collection. However, a further comparison of the ^1^H NMR data indicates that the most significant differences between the two spectra relate to the α-proton region (4.0–5.2 ppm), specifically residues *L*-Ala and *N*-Me-*L*-Leu (Figure 4D and Scheme S1A). Indeed, while most of the α-proton chemical shifts in **9a** are indicative of a random coil, the shifts of *L*-Ala and *N*-Me-Leu at the midpoint of the peptide are indicative of β sheet formation. In contrast, peptide **14a** shows no indication of a secondary structure (Scheme S1A). This partial organization in **9a** agrees with the CD data (Scheme S1D).

To explain these observations and gain further insights at the atomic level, we carried out molecular dynamics (MD) simulations on reactive acyl azide intermediates **S9** and **S14**. These simulations show **S9** to have the longest intramolecular H-bond lifetimes (Figure 4B), suggesting that aspects of its structure are indeed comparatively stable. The most frequent H-bond interaction for the fast-cyclizing peptide **S9** is the N2–O4 interaction (Figure S6), which creates a backbone conformation that brings the *N-* and *C*-termini of the peptide in close enough proximity to facilitate cyclization (Figure 4A, right). **S15**, which exhibits intermediate cyclization efficiency, behaves similarly over the course of the simulations although there is a competing H-bond from N1 to O4 (Figure S8). For both peptides, **S9** and **S15**, the backbone segment that includes N2 and O4 is stable barring occasional inversions in the magnitude of the angle (Figure S5). Comparison of the conformational flexibility in the structural ensembles of each peptide (Figure 4A, right panel) shows that the *C*-terminus of **S9** is less flexible than the *C*-termini of peptides **S14** and **S15**, indicating that **S9** has a more rigid conformation. Fluctuational analyses (Figure 4C) show that the speed at which **S9** cyclizes can also be partially explained by the fact that its structural ensemble has the lowest root-mean-square deviation (RMSD) from the mean reference conformation compatible with cyclization, while the slower cyclizing **S14** peptide has the greatest RMSD. Indeed, RMSD values have been used as a complementary screening tool to identify conformationally stable cyclic peptides.^62^ These findings are in agreement with the NMR and CD data. Taken together, these data suggest that backbone methylation and thus peptide conformation are key factors for successful and efficient macrocyclization.

### Probing the Impact of Side Chains, Sterics, and Backbone Flexibility on Reaction Rate and Conversion

**Figure 5:**
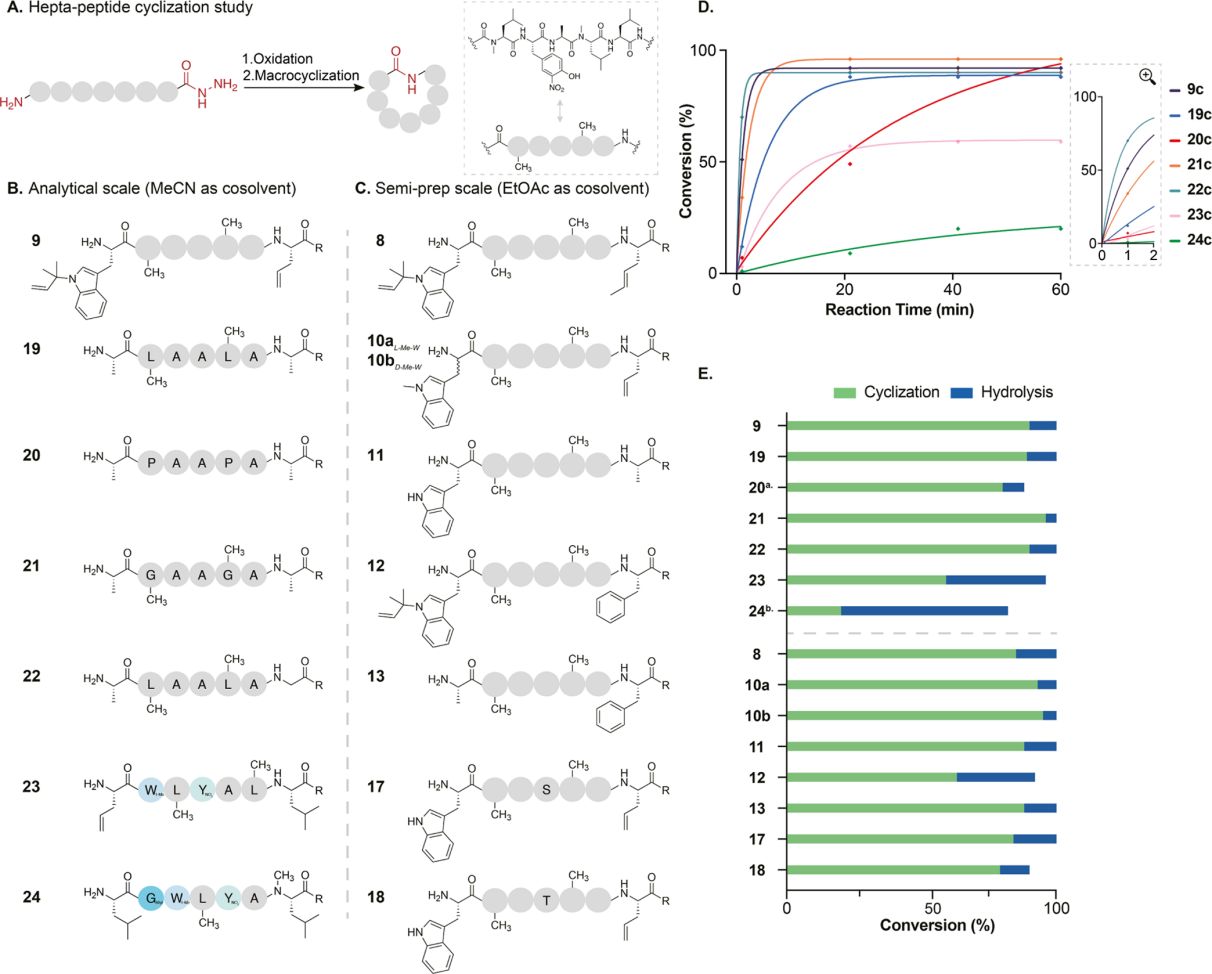
Side chain and steric effects on macrocyclization. (A) Head-to-tail peptide cyclization of rufomycin analogues. (B) Analytical scale reactions using MeCN as a cosolvent (33% v/v), 21 min reaction time. (C) Semipreparative scale biphasic reactions with EtOAc (50% v/v), 15 min reaction time. Peptide sequences based on rufomycin (including positions of backbone modifications) were used to probe cyclization reaction. (D) Relative cyclization rates for peptides **9** and **19**–**24**. Data from HPLC analysis were fitted using the nonlinear regression (time points 1, 21, 41, and 61 min) (Figures S18 and S21). (E) Conversion of linear peptide to cyclized or hydrolyzed products was calculated using HPLC at 214 nm or 355 nm for peptides containing 3-nitro-*L*-tyrosine (^a^reaction time 41 min, ^b^reaction time 81 min).

While *N*-methylation appears crucial to successful cyclization of rufomycin, we decided to also utilize this system to explore the impact of other factors including side chains, sterics, and cyclization position on the reaction rate and conversion to both cyclic and hydrolyzed products. A series of linear rufomycin derivatives (**8**–**13** and **17**–**24**) were synthesized, and following cleavage from the resin, they were subjected to chemical cyclization in their crude form (Figure 5A–C). The conversion and rate of macrocyclization were compared (Figure 5D,E). Time points are limited by the speed of the reaction and the use of discontinuous assay by HPLC analysis.

To investigate the impact of side chains, we first investigated simplified peptides **19** and **21** where several residues were changed to alanine (Figure 5B). Both peptides are cyclized with comparable conversion, but the rate of macrocyclization is slower than **9** (Figures 5B,D and S21), indicating that while *N*-methylation is the dominant factor influencing cyclization in rufomycins, side-chain interactions also play a role. Turning to the peptide backbone, the methylated amides were replaced with the turn-inducing residue *L*-proline. The cyclization of peptide **20** was accomplished but intriguingly at a significantly slower rate (requiring 40 min to complete) (Figures 5B,D and S21). Surprisingly, despite the slow reaction, there was no increase in the level of hydrolysis. The reduced reaction rate results from the slow interconversion between proline *cis*/*trans* isomers, negatively impacting the ability of the peptide to achieve a procyclization conformation. Additionally, we assayed peptide **22**, which introduces glycine at the *C*-terminus, thus removing the chiral center, reducing steric hindrance, and increasing flexibility. Indeed, the rate of reaction increases over peptide **19**, but interestingly, the ratio of cyclic to hydrolyzed products is unaffected (Figures 5E and S21). Lastly, we investigated the importance of the biosynthetically determined point of cyclization using peptides **23** and **24** (Figure S18). In line with our hypothesis, both peptides reacted very slowly, this time resulting in significant hydrolysis and low conversion. Peptide **24** cyclizes at the slowest rate and results in the poorest conversion to cyclic peptide (20%). Indeed, the level of hydrolysis of peptide **24** is greater than that of any other in the panel.

To investigate the impact of sterics at the *N*- and *C*-termini, peptides **8**–**13** and **17** and **18** were synthesized (Figure 5C). Nonproteinogenic amino acid, *trans-L-*crotyl glycine, in natural product rufomycin B **8c** was prepared with high optical purity (e.e. 97%) using the published methodology (Figures S1 and S20).^63^ All cyclic rufomycin analogues were synthesized with excellent conversion (Figure 5C,E ) including natural product, rufomycin B **8c** (Figures S4 and S19). Peptide **12**, in which both *C*- and *N*-termini are very bulky (*N*-prenyl-*L*-Trp and *L*-Phe), gave the lowest conversion (55%), indicating that side-chain steric clash at the termini can, unsurprisingly, negatively impact cyclization. **17** and **18** also demonstrate that the chemistry can tolerate unprotected alcohol containing side chains. Finally, heterochiral macrolactamization has been reported to be more favorable than homochiral macrolactamization *in vitro*,^9,64^ and in fact most NRcPs are cyclized biosynthetically with heterochiral termini (rufomycin and cyclomarin are exceptions). Thus, peptide **10b** was synthesized and it in fact leads to the highest conversion (95%) of all of the peptides (Figure S15F).

Remarkably, despite the relatively high concentration at which these reactions were performed (range from 2 to 6 mM), no dimerization products were observed. Additionally, we did not detect epimerization in these reactions. The cyclized products **8c**–**13c** were purified by reversed phase HPLC to afford ∼10 mg of each cyclic peptide, allowing full characterization by MS and NMR (see the Supporting Data). Taken together, these data indicate that the macrocyclization chemistry is robust to substantial changes in the peptide sequence if conformation is maintained, as dictated by the biosynthetic process. For rufomycin, amino acid side chains appear to have a minor impact on the reaction outcome, allowing significant alteration of the sequence, but the presence of both *N*-methyl groups and respecting the point of biosynthetic cyclization are crucial.

### Bioinspired Chemical Macrocyclization: Application to Structurally Diverse Peptides

**Figure 6:**
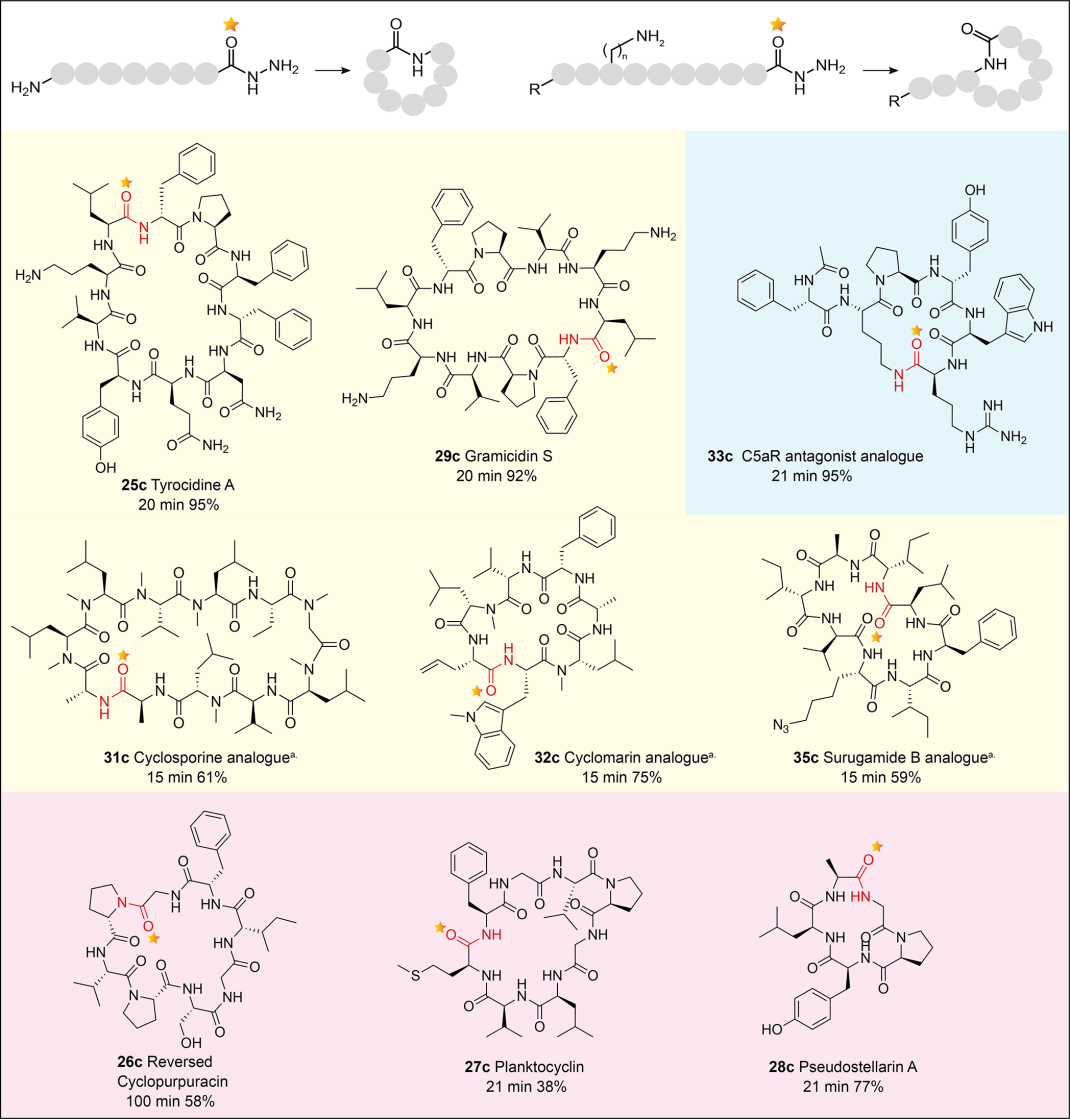
Application of bioinspired macrocyclization to cyclic peptides, varying compositions, ring sizes, and cyclization modes. Nonribosomal cyclic peptides (yellow), cyclic peptides of unconfirmed biosynthetic origin and thus unknown biosynthetic point of cyclization (red), and synthetic peptides (blue). Planktocyclin **27c** and pseudostellarin A **28c** are proposed ribosomal peptides, and R-cyclopurpuracin **26c** is of unknown biosynthetic origin. ^a^EtOAc was used as the cosolvent in the cyclization step, so the reaction is biphasic. MeCN (33% v/v) was used in all other cases; the reaction is single phase.

**Figure 7:**
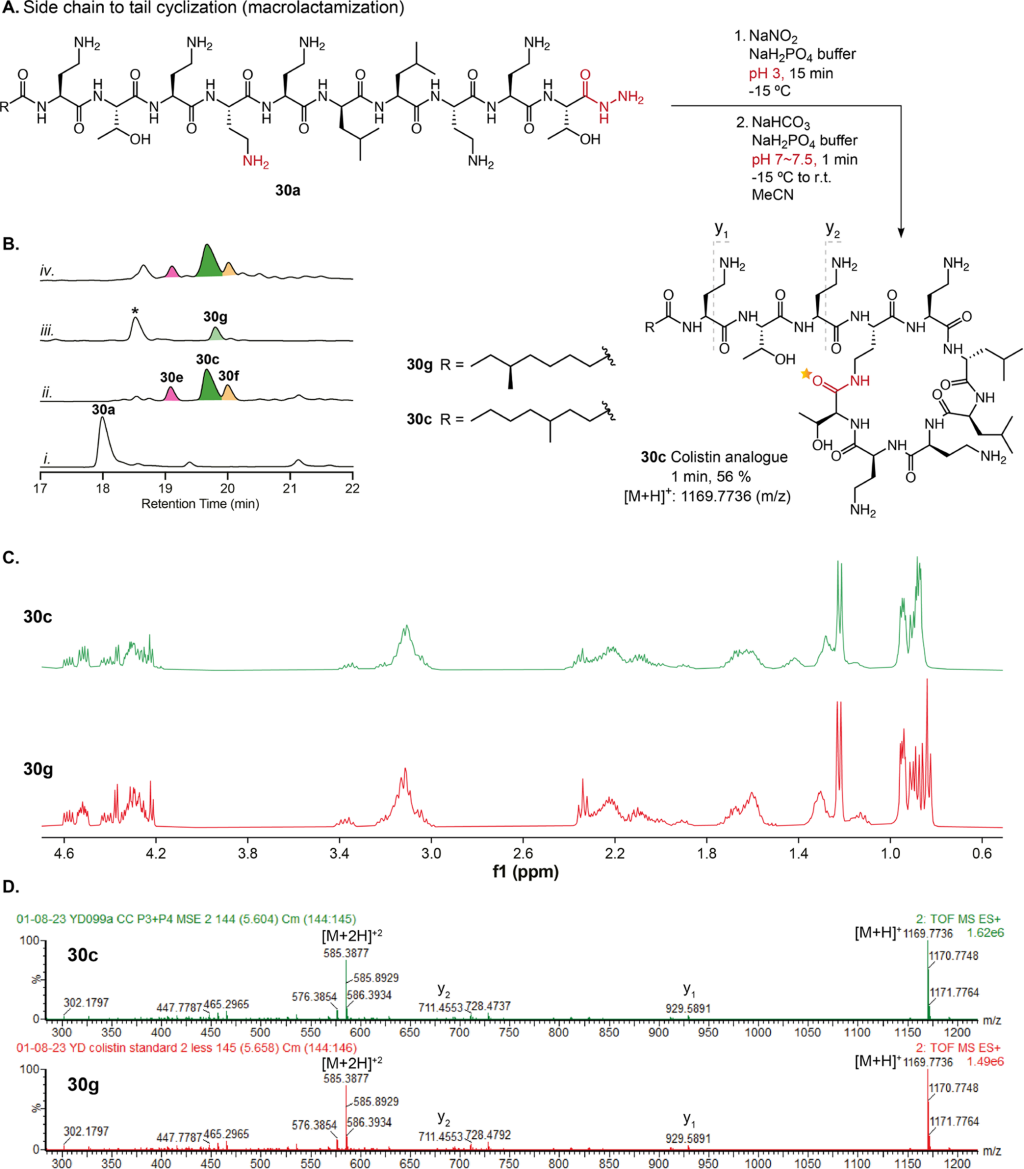
Regioselective synthesis of colistin analogue via side-chain-to-tail macrocyclization. (A) Regioselective synthesis of colistin analogue **30c**. (B) HPLC analysis (214 nm) of the chemical cyclization. (i) Crude starting linear peptide **30a**. (ii) Crude cyclization reaction 1 min after neutralization. (iii) Commercial standard colistin sold as a mixture of polymyxin E_1_**30g**, polymyxin E_2_ indicated with *. (iv) Coinjection of cyclization reaction mixture and commercial standard (1:1 mix). (C) ^1^H NMR spectra of **30g** and **30c** (400 MHz, D_2_O). Differences in the region of 0.8–1.0 ppm correspond to structural differences resulting from regioisomeric lipid chains. (D) Comparison of MS^E^ spectra confirms that **30c** and **30g** have identical fragmentation patterns. Comparison of MS^E^ spectra for peptides **30c**, **30e**, **30f**, **30g** and more detailed fragmentation analysis can be found in the Supporting Information (Figure S26 and Supporting Spectra).

To determine the broader applicability of the chemistry to NRcPs more generally, we selected targets of varying sequences, ring sizes, and modes of cyclization, i.e., head to tail and side chain to tail (Figures 6 and 7). For NRcPs, the linear precursor design mimics the biosynthetic linear precursor. The reactions were not subjected to further optimization of the reaction conditions. The only optimization considered for each new peptide was the choice of cosolvent (MeCN or EtOAc).

#### *N*-Methylated NRcP Natural Products

We first attempted the synthesis of a cyclomarin analogue **32c** where, for proof of principle, nonproteinogenic amino acids were simplified. Cyclomarin, a heptapeptide, shares structural and biosynthetic commonalities with rufomycin but has a different pattern of backbone *N*-methylation (residues 2 and 6).^65^ The resulting linear peptide **32a** was rapidly cyclized to **32c** with excellent conversion, with the biphasic buffer/EA conditions giving the cleanest reaction and minimizing epimerization (Figure S27). The clinically important immunosuppressant cyclosporine (11-mer) is cyclized biosynthetically by a specialized *N*-terminal condensation domain, common in fungal NRPS enzymes.^66−69^ Cyclosporine contains butenyl-*N*-methyl-*L*-threonine (Bmt), which is not commercially available and for this proof of principle was replaced with *N*-methyl-*L*-leucine. The purified linear cyclosporine analogue **31a** was cyclized to give **31c** within 15 min and excellent conversion (61%) (Figure S24). Cyclosporine and cyclomarin contain *N*-methylated backbones, and thus from our rufomycin studies, we propose that this property drives cyclization. No dimerization was detected in these reactions.

#### NRcP Natural Products Containing Competing Nucleophiles

Decapeptides tyrocidine A **25c** and gramicidin S **29c** are not *N*-methylated and crucially contain internal alternative nucleophiles in the form of *L*-ornithine side chains (Figure 6). The linear peptides were synthesized corresponding to the final covalently NRPS-bound peptide^66,67^ and subjected to macrocyclization, resulting in >90% conversion in 20 min (Figures S22 and S24). No branched cyclization products were detected (NMR data in the Supporting Information). This illustrates the importance of conformation and the controlled pH conditions, ensuring that due to well-understood differences in p*K*_aH_ between the *N-*terminal and side-chain amines, only the *N*-terminus will be deprotonated to a sufficient degree at pH 7. While tyrocidine A and gramicidin S do not contain backbone *N*-methylation, their linear peptides have been previously reported to be highly organized via intramolecular hydrogen bonds and the presence of *L*-proline in their sequences.^70^ No dimerization or epimerization products were detected. As further confirmation of the fully characterized products, both gramicidin S and tyrocidine A were tested for antimicrobial activity. MIC values determined for gramicidin S (*Enterococcus faecium* 2.5 μM and *Staphylococcus aureus* 3.1 μM) and tyrocidine A (*Bacillus subtilis* 3.1 μM and *S. aureus* 6.3 μM) are in line with those previously reported (Table S1).^71,72^

We also attempted the synthesis of the NRcP, surugamide B **34c**, which does not contain a turn-inducing residue or motif but does contain a potential competing *L*-Lys side chain. The linear peptide **34a** was converted rapidly (20 min), in ∼90% conversion, to a cyclic derivative (Figure S28). However, 2D NMR revealed that despite the p*K*_aH_ differences, this product resulted from cyclization onto the lysine side chain and not the *N*-terminus. Attempts to slow the reaction by altering the pH and temperature led to incomplete reaction or side reactions. A previous report of slow spontaneous cyclization of SNAC-surugamide B gives the same branched product.^14^ This result may be explained by the unusual biosynthesis of surugamide B, in which the cyclization step is catalyzed by a standalone penicillin binding protein (PBP)-like enzyme (SurE) rather than a canonical TE domain.^14^ To overcome the problem, we synthesized Boc-protected surugamide B **Boc-34a**, but the resulting highly hydrophobic peptide was poorly soluble in the reaction buffer (Figure S28). However, changing Boc-*L*-lysine to azido-*L*-lysine to produce derivative **35a** proved to be successful (Figures 6 and S28). Interestingly, **35a** cyclized slowly in MeCN/H_2_O (incomplete after 60 min) with significant levels of hydrolysis. A biphasic EtOAc/H_2_O system proved significantly faster with very good conversion to **35c** (Figures 6 and S28). These data indicate that SurE plays a vital role in protecting the amine side chain to enable head to tail cyclization.

Lastly, side-chain-to-tail cyclization is common in NRcP biosynthesis but is a significant challenge where the NRcP linear precursor contains multiple side chains of equal or similar reactivity. This is the case for the frontline antibiotic, colistin. Colistin is sold as a mixture of two related compounds that differ in their lipid tails (polymyxin E_1_ and E_2_; Figure 7Biii).^73^ The nonribosomal peptides, polymyxins, have been the focus of several total syntheses and efforts to generate analogues for improved properties (e.g., reduced toxicity) and to evade antimicrobial resistance.^74−76^ The challenge for our chemistry is the presence of multiple *L*-Dab residues as potential competing nucleophiles and the absence of any turn-inducing residues. However, this was an opportunity to determine if the linear polymixin precursor peptide was predisposed to cyclize to give the natural macrolactam or, if the cyclization conformation was induced by binding to the cyclization enzyme (TE domain) as appears to be the case for surugamide B. Thus, linear fully deprotected acyl azide peptide **30a** was synthesized according to biosynthetic logic.^73^ For this proof of concept, a commercially available regioisomer of the natural lipid tail was used (Figure 7A). The peptide was subjected to cyclization. HPLC analysis at 1 min revealed complete conversion of the acyl azide and formation of 3 major products (Figures 7Bii and S25). Excitingly, the most intense peak correlating to product **30c** (56%) was identified by MS^E^ fragmentation and ^1^H NMR to contain the same macrocycle as the commercial natural product **30g** (Figures 7B,C and S26B,C and Supporting Spectra). From MS^E^, the other two byproducts **30e** and **30f** are likely to represent larger (17%) and smaller macrolactam rings (21%) formed by the adjacent Dab residues acting as competing nucleophiles (Figures S25 and S26). The extraordinary regioselectivity and exceptional reaction rate could only be achieved through conformational bias, as determined by the linear module organization of the NRPS (PmxA, B, and E) responsible for polymyxin biosynthesis.^73^ No hydrolysis, dimerization, or epimerization products were detected in these reactions.

#### Peptides of Synthetic and Unknown Biosynthetic Origin

Finally, we aimed to demonstrate that this chemistry can be used as a general peptide cyclization method. We first targeted a synthetic C5aR antagonist peptide developed by Pfizer.^77^ For proof of concept, we designed an analogue **33c**. The precursor linear peptide underwent rapid cyclization in excellent conversion, equaling that of the reported optimized chemistry (Figures 6 and S28). We identified other natural products for which the linear biosynthetic peptide precursors, and thus the point of cyclization, was unknown and successfully prepared the plant ribosomal natural product, pseudostellarin A^78^**28c** (Figure S23), reversed natural product *R*-cyclopurpuracin^79^**26c** (Figure S22), and the cyanobacterial natural product, planktocyclin^80^**27c** (Figure S23), all in good to excellent conversion (Figure 6).

We note that *R*-cyclopurpuracin contains two proline residues and the slow rate of this reaction (complete conversion of the linear acyl azide was only achieved after 100 min) agrees with the significantly slower cyclization rate of rufomycin analogue **20**, which also contains two proline residues (Figures 5, S21, and S22). *R*-cyclopurpuracin **26c** was targeted due to reported improved activity over the natural product; however, we did not detect significant antimicrobial activity (Table S1). Planktocyclin shows the lowest conversion of all peptides tested, with hydrolysis being the most significant competing reaction. Conversion could perhaps be improved with the knowledge of the biosynthetic point of cyclization. Importantly, despite the oxidative conditions used in acyl azide formation, no methionine oxidation was detected, as previously reported.^38^

Collectively, these diverse examples demonstrate the general applicability of this method and that a wide variety of proteinogenic and nonproteinogenic amino acids are stable to the cyclization conditions. As yet, we have not identified a clear correlation between reaction rates and hydrolysis or epimerization. For example, while planktocyclin rapidly hydrolyzes (59%, 20 min), both R-cyclopurpuracin and rufomycin derivative **20** react at a much slower rate (100 min and 41 min, respectively) but result in less hydrolysis (39 and 9%, respectively). As illustrated by, for example, the azido-surugamide B analogue **35c** and cyclomarin analogue **32c**, using a cosolvent appears to have the greatest impact on these side reactions, likely due to separation of the acyl azide peptides from aqueous buffer. However, taken together, our data imply that the susceptibility of the *C*-terminus to hydrolysis or epimerization is a complex combination of several factors including reaction conditions, peptide sequences, and the nature of the *C*-terminal amino acid. Understanding this phenomenon is the focus of ongoing investigations.

## Conclusions

The lack of robust and operationally simple strategies to synthesize nonribosomal cyclic peptide natural products and their derivatives limits the investigation of NRcP biosynthesis toward pathway engineering and stimies their development as drugs. Here, we have described a novel bioinspired approach to peptide cyclization for the facile synthesis of NRcPs and cyclic peptides more generally. The method is rapid, operationally simple, and, with little optimization, achieves good to excellent conversions with no dimerization and minimal epimerization.

We have shown that the success of this chemistry relies heavily on peptide conformation. In NRcP biosynthesis, preorganization is achieved via backbone amide methylation (e.g., rufomycin and cyclosporin) or turn-inducing residues such glycine, proline, and *D*-amino acids (e.g., tyrocidine A and gramicidin S), with additional contributions from side-chain interactions. In addition, a highly reactive *C*-terminus is required alongside correctly balanced pH to activate the *N*-terminus while minimizing hydrolysis and epimerization. We achieved this using a *C*-terminal acyl azide and a combined aqueous/solvent environment. This combination of conditions leads to a robust and generalizable NRcP cyclization methodology that was applied to a wide range of peptides varying in ring size, polarity, and cyclization mode.

This chemistry is exceptional in comparison to previous reports of NRcP synthesis (reaction time: tyrocidine, 2 h,^70^ 6 h,^81^ and overnight,^46^ cyclosporin and surugamide, hrs^12,14^) even where the same cyclization point is used, resulting in high to excellent conversions. Conformationally driven macrocyclization, using unprotected peptides, also allows excellent regioselectivity in the presence of competing nucleophiles as demonstrated by our ability to synthesize a colistin analogue (56% conversion) using a fully deprotected precursor. This approach represents improved atom economy over standard coupling methods but is also protecting group tolerant, where necessary. Additionally, it has some advantages over biocatalytic approaches where multiple enzymes would be required to cyclize the same range of structurally diverse compounds (Figures 5–7).

This work has also generated insight into NRcP biosynthesis and the different roles played by NRPS cyclization domains. In cases such as rufomycin, a procyclization conformation is determined by the primary peptide sequence, as encoded by the NRPS linear module order including the backbone *N*-methylation pattern. The TE domain is required only to activate the termini and provide a hydrophobic environment to catalyze cyclization. Conversely in surugamide B biosynthesis, the enzyme’s role is also to prevent the preferred cyclization onto the lysine side chain. Polymyxin biosynthesis is a combination of these two situations in that there is a preferred conformation dictated by the NPRS module order, but the TE domain is required to transiently “protect” the competing nucleophiles to produce a single macrolactam product.

As NRcPs have long been a vital pool of therapeutics, we anticipate that this powerful new synthetic tool will both facilitate synthetic and pathway engineering approaches to NRcP production.^82−84^ More generally, we hope it will aid the generation of bioactive cyclic peptide libraries toward antibiotic development in the fight against AMR.

## Supporting Information Available

Experimental procedures and methods, characterization data and computational details (PDF)

## Abbreviations

EA: ethyl acetate
NRcP: nonribosomal cyclic peptide
DCM: dichloromethane
TE: thioesterase
GdmCl: guanidinium chloride
NRPS: nonribosomal peptide synthetase
HATU: hexafluorophosphate azabenzotriazole tetramethyl uranium
DIPEA: *N*,*N*-diisopropylethylamine
DMF: dimethylformamide
NMP: *N*-methylpyrrolidone
MeCN: acetonitrile
SPPS: solid-phase peptide synthesis
TFA: trifluoroacetic acid
RMSD: root-mean-square deviation
MD: molecular dynamics

## References

[ref1] Hutchings M. I.; Truman A. W.; Wilkinson B. Antibiotics: Past, Present and Future. Curr. Opin. Microbiol. 2019, 51, 72–80. 10.1016/j.mib.2019.10.008. 31733401

[ref2] Murray C. J. L.; Ikuta K. S.; Sharara F.; Swetschinski L.; Robles Aguilar G.; Gray A.; Han C.; Bisignano C.; Rao P.; Wool E.; Johnson S. C.; Browne A. J.; Chipeta M. G.; Fell F.; Hackett S.; Haines-Woodhouse G.; Kashef Hamadani B. H.; Kumaran E. A. P.; McManigal B.; Achalapong S.; Agarwal R.; Akech S.; Albertson S.; Amuasi J.; Andrews J.; Aravkin A.; Ashley E.; Babin F.-X.; Bailey F.; Baker S.; Basnyat B.; Bekker A.; Bender R.; Berkley J. A.; Bethou A.; Bielicki J.; Boonkasidecha S.; Bukosia J.; Carvalheiro C.; Castañeda-Orjuela C.; Chansamouth V.; Chaurasia S.; Chiurchiù S.; Chowdhury F.; Clotaire Donatien R.; Cook A. J.; Cooper B.; Cressey T. R.; Criollo-Mora E.; Cunningham M.; Darboe S.; Day N. P. J.; De Luca M.; Dokova K.; Dramowski A.; Dunachie S. J.; Duong Bich T.; Eckmanns T.; Eibach D.; Emami A.; Feasey N.; Fisher-Pearson N.; Forrest K.; Garcia C.; Garrett D.; Gastmeier P.; Giref A. Z.; Greer R. C.; Gupta V.; Haller S.; Haselbeck A.; Hay S. I.; Holm M.; Hopkins S.; Hsia Y.; Iregbu K. C.; Jacobs J.; Jarovsky D.; Javanmardi F.; Jenney A. W. J.; Khorana M.; Khusuwan S.; Kissoon N.; Kobeissi E.; Kostyanev T.; Krapp F.; Krumkamp R.; Kumar A.; Kyu H. H.; Lim C.; Lim K.; Limmathurotsakul D.; Loftus M. J.; Lunn M.; Ma J.; Manoharan A.; Marks F.; May J.; Mayxay M.; Mturi N.; Munera-Huertas T.; Musicha P.; Musila L. A.; Mussi-Pinhata M. M.; Naidu R. N.; Nakamura T.; Nanavati R.; Nangia S.; Newton P.; Ngoun C.; Novotney A.; Nwakanma D.; Obiero C. W.; Ochoa T. J.; Olivas-Martinez A.; Olliaro P.; Ooko E.; Ortiz-Brizuela E.; Ounchanum P.; Pak G. D.; Paredes J. L.; Peleg A. Y.; Perrone C.; Phe T.; Phommasone K.; Plakkal N.; Ponce-de-Leon A.; Raad M.; Ramdin T.; Rattanavong S.; Riddell A.; Roberts T.; Robotham J. V.; Roca A.; Rosenthal V. D.; Rudd K. E.; Russell N.; Sader H. S.; Saengchan W.; Schnall J.; Scott J. A. G.; Seekaew S.; Sharland M.; Shivamallappa M.; Sifuentes-Osornio J.; Simpson A. J.; Steenkeste N.; Stewardson A. J.; Stoeva T.; Tasak N.; Thaiprakong A.; Thwaites G.; Tigoi C.; Turner C.; Turner P.; Van Doorn H. R.; Velaphi S.; Vongpradith A.; Vongsouvath M.; Vu H.; Walsh T.; Walson J. L.; Waner S.; Wangrangsimakul T.; Wannapinij P.; Wozniak T.; Young Sharma T. E. M. W.; Yu K. C.; Zheng P.; Sartorius B.; Lopez A. D.; Stergachis A.; Moore C.; Dolecek C.; Naghavi M. Global Burden of Bacterial Antimicrobial Resistance in 2019: A Systematic Analysis. Lancet 2022, 399 (10325), 629–655. 10.1016/S0140-6736(21)02724-0. 35065702 PMC8841637

[ref3] Vinogradov A. A.; Yin Y.; Suga H. Macrocyclic Peptides as Drug Candidates: Recent Progress and Remaining Challenges. J. Am. Chem. Soc. 2019, 141 (10), 4167–4181. 10.1021/jacs.8b13178. 30768253

[ref4] Hill T. A.; Shepherd N. E.; Diness F.; Fairlie D. P. Constraining Cyclic Peptides To Mimic Protein Structure Motifs. Angew. Chem., Int. Ed. 2014, 53 (48), 13020–13041. 10.1002/anie.201401058. 25287434

[ref5] Arnison P. G.; Bibb M. J.; Bierbaum G.; Bowers A. A.; Bugni T. S.; Bulaj G.; Camarero J. A.; Campopiano D. J.; Challis G. L.; Clardy J.; Cotter P. D.; Craik D. J.; Dawson M.; Dittmann E.; Donadio S.; Dorrestein P. C.; Entian K.-D.; Fischbach M. A.; Garavelli J. S.; Göransson U.; Gruber C. W.; Haft D. H.; Hemscheidt T. K.; Hertweck C.; Hill C.; Horswill A. R.; Jaspars M.; Kelly W. L.; Klinman J. P.; Kuipers O. P.; Link A. J.; Liu W.; Marahiel M. A.; Mitchell D. A.; Moll G. N.; Moore B. S.; Müller R.; Nair S. K.; Nes I. F.; Norris G. E.; Olivera B. M.; Onaka H.; Patchett M. L.; Piel J.; Reaney M. J. T.; Rebuffat S.; Ross R. P.; Sahl H.-G.; Schmidt E. W.; Selsted M. E.; Severinov K.; Shen B.; Sivonen K.; Smith L.; Stein T.; Süssmuth R. D.; Tagg J. R.; Tang G.-L.; Truman A. W.; Vederas J. C.; Walsh C. T.; Walton J. D.; Wenzel S. C.; Willey J. M.; van der Donk W. A. Ribosomally Synthesized and Post-Translationally Modified Peptide Natural Products: Overview and Recommendations for a Universal Nomenclature. Nat. Prod. Rep. 2013, 30 (1), 108–160. 10.1039/C2NP20085F. 23165928 PMC3954855

[ref6] Fischbach M. A.; Walsh C. T. Assembly-Line Enzymology for Polyketide and Nonribosomal Peptide Antibiotics: Logic, Machinery, and Mechanisms. Chem. Rev. 2006, 106 (8), 3468–3496. 10.1021/cr0503097. 16895337

[ref7] Walsh C. T. Polyketide and Nonribosomal Peptide Antibiotics: Modularity and Versatility. Science 2004, 303 (5665), 1805–1810. 10.1126/science.1094318. 15031493

[ref8] Süssmuth R. D.; Mainz A. Nonribosomal Peptide Synthesis-Principles and Prospects. Angew. Chem., Int. Ed. 2017, 56 (14), 3770–3821. 10.1002/anie.201609079. 28323366

[ref9] White C. J.; Yudin A. K. Contemporary Strategies for Peptide Macrocyclization. Nat. Chem. 2011, 3 (7), 509–524. 10.1038/nchem.1062. 21697871

[ref10] Cheng Y.; Tang S.; Guo Y.; Ye T. Total Synthesis of Anti-Tuberculosis Natural Products Ilamycins E _1_ and F. Org. Lett. 2018, 20 (19), 6166–6169. 10.1021/acs.orglett.8b02643. 30252492

[ref11] Greve J.; Mogk A.; Kazmaier U. Total Synthesis and Biological Evaluation of Modified Ilamycin Derivatives. Mar. Drugs 2022, 20 (10), 632 10.3390/md20100632. 36286456 PMC9605216

[ref12] Wu X.; Stockdill J. L.; Wang P.; Danishefsky S. J. Total Synthesis of Cyclosporine: Access to N-Methylated Peptides via Isonitrile Coupling Reactions. J. Am. Chem. Soc. 2010, 132 (12), 4098–4100. 10.1021/ja100517v. 20199071 PMC2844917

[ref13] Barbie P.; Kazmaier U. Total Synthesis of Cyclomarin A, a Marine Cycloheptapeptide with Anti-Tuberculosis and Anti-Malaria Activity. Org. Lett. 2016, 18 (2), 204–207. 10.1021/acs.orglett.5b03292. 26699807

[ref14] Kuranaga T.; Matsuda K.; Sano A.; Kobayashi M.; Ninomiya A.; Takada K.; Matsunaga S.; Wakimoto T. Total Synthesis of the Nonribosomal Peptide Surugamide B and Identification of a New Offloading Cyclase Family. Angew. Chem., Int. Ed. 2018, 57 (30), 9447–9451. 10.1002/anie.201805541. 29808953

[ref15] Chow H. Y.; Zhang Y.; Matheson E.; Li X. Ligation Technologies for the Synthesis of Cyclic Peptides. Chem. Rev. 2019, 119 (17), 9971–10001. 10.1021/acs.chemrev.8b00657. 31318534

[ref16] Lam H. Y.; Zhang Y.; Liu H.; Xu J.; Wong C. T. T.; Xu C.; Li X. Total Synthesis of Daptomycin by Cyclization via a Chemoselective Serine Ligation. J. Am. Chem. Soc. 2013, 135 (16), 6272–6279. 10.1021/ja4012468. 23560543

[ref17] Chaudhuri D.; Ganesan R.; Vogelaar A.; Dughbaj M. A.; Beringer P. M.; Camarero J. A. Chemical Synthesis of a Potent Antimicrobial Peptide Murepavadin Using a Tandem Native Chemical Ligation/Desulfurization Reaction. J. Org. Chem. 2021, 86 (21), 15242–15246. 10.1021/acs.joc.1c01858. 34641669 PMC8935662

[ref18] Schmidt J. J.; Khatri Y.; Brody S. I.; Zhu C.; Pietraszkiewicz H.; Valeriote F. A.; Sherman D. H. A Versatile Chemoenzymatic Synthesis for the Discovery of Potent Cryptophycin Analogs. ACS Chem. Biol. 2020, 15 (2), 524–532. 10.1021/acschembio.9b00998. 31961651 PMC7094870

[ref19] Forneris C. C.; Seyedsayamdost M. R. In Vitro Reconstitution of OxyC Activity Enables Total Chemoenzymatic Syntheses of Vancomycin Aglycone Variants. Angew. Chem., Int. Ed. 2018, 57 (27), 8048–8052. 10.1002/anie.201802856. 29697176

[ref20] Kobayashi M.; Fujita K.; Matsuda K.; Wakimoto T. Streamlined Chemoenzymatic Synthesis of Cyclic Peptides by Non-Ribosomal Peptide Cyclases. J. Am. Chem. Soc. 2023, 145 (6), 3270–3275. 10.1021/jacs.2c11082. 36638272

[ref21] Trauger J. W.; Kohli R. M.; Mootz H. D.; Marahiel M. A.; Walsh C. T. Peptide Cyclization Catalysed by the Thioesterase Domain of Tyrocidine Synthetase. Nature 2000, 407 (6801), 215–218. 10.1038/35025116. 11001063

[ref22] Matsuda K.; Zhai R.; Mori T.; Kobayashi M.; Sano A.; Abe I.; Wakimoto T. Heterochiral Coupling in Non-Ribosomal Peptide Macrolactamization. Nat. Catal. 2020, 3 (6), 507–515. 10.1038/s41929-020-0456-7.

[ref23] Wever W. J.; Bogart J. W.; Baccile J. A.; Chan A. N.; Schroeder F. C.; Bowers A. A. Chemoenzymatic Synthesis of Thiazolyl Peptide Natural Products Featuring an Enzyme-Catalyzed Formal [4 + 2] Cycloaddition. J. Am. Chem. Soc. 2015, 137 (10), 3494–3497. 10.1021/jacs.5b00940. 25742119 PMC4425689

[ref24] Takita T.; Ohi K.; Okami Y.; Maeda K.; Umezawa H. New Antibiotics, Ilamycins. J. Antibiot., Ser. A 1962, 15 (1), 46–48. 10.11554/antibioticsa.15.1_46. 14039629

[ref25] Shibata M.; Higashide E.; Yamamoto H.; Nakazawa K.; Iwasaki H.; Ueyanagi J.; Miyake A. Studies on Streptomycetes: Part I. Streptomyces Atratus Nov. Sp., Producing New Antituberculous Antibiotics Rufomycin A and BPart II. Rufomycin A and B, New Antituberculous Antibiotics. Agric. Biol. Chem. 1962, 26 (4), 228–237. 10.1080/00021369.1962.10857966.

[ref26] Xie Q.; Yang Z.; Huang X.; Zhang Z.; Li J.; Ju J.; Zhang H.; Ma J. Ilamycin C Induces Apoptosis and Inhibits Migration and Invasion in Triple-Negative Breast Cancer by Suppressing IL-6/STAT3 Pathway. J. Hematol. Oncol. 2019, 12 (1), 60 10.1186/s13045-019-0744-3. 31186039 PMC6558915

[ref27] Choules M. P.; Wolf N. M.; Lee H.; Anderson J. R.; Grzelak E. M.; Wang Y.; Ma R.; Gao W.; McAlpine J. B.; Jin Y.-Y.; Cheng J.; Lee H.; Suh J.-W.; Duc N. M.; Paik S.; Choe J. H.; Jo E.-K.; Chang C. L.; Lee J. S.; Jaki B. U.; Pauli G. F.; Franzblau S. G.; Cho S. Rufomycin Targets ClpC1 Proteolysis in Mycobacterium Tuberculosis and M. Abscessus. Antimicrob. Agents Chemother. 2019, 63 (3), 10–1128. 10.1128/AAC.02204-18. PMC6395927 30602512

[ref28] Wolf N. M.; Lee H.; Choules M. P.; Pauli G. F.; Phansalkar R.; Anderson J. R.; Gao W.; Ren J.; Santarsiero B. D.; Lee H.; Cheng J.; Jin Y.-Y.; Ho N. A.; Duc N. M.; Suh J.-W.; Abad-Zapatero C.; Cho S. High-Resolution Structure of ClpC1-Rufomycin and Ligand Binding Studies Provide a Framework to Design and Optimize Anti-Tuberculosis Leads. ACS Infect. Dis. 2019, 5 (6), 829–840. 10.1021/acsinfecdis.8b00276. 30990022 PMC6657506

[ref29] Sun C.; Liu Z.; Zhu X.; Fan Z.; Huang X.; Wu Q.; Zheng X.; Qin X.; Zhang T.; Zhang H.; Ju J.; Ma J. Antitubercular Ilamycins from Marine-Derived *Streptomyces Atratus* SCSIO ZH16 Δ *ilaR*. J. Nat. Prod. 2020, 83 (5), 1646–1657. 10.1021/acs.jnatprod.0c00151. 32324401

[ref30] Zhou B.; Shetye G.; Yu Y.; Santarsiero B. D.; Klein L. L.; Abad-Zapatero C.; Wolf N. M.; Cheng J.; Jin Y.; Lee H.; Suh J.-W.; Lee H.; Bisson J.; McAlpine J. B.; Chen S.-N.; Cho S.-H.; Franzblau S. G.; Pauli G. F. Antimycobacterial Rufomycin Analogues from Streptomyces Atratus Strain MJM3502. J. Nat. Prod. 2020, 83 (3), 657–667. 10.1021/acs.jnatprod.9b01095. 32031795 PMC7384767

[ref31] Dadgostar P. Antimicrobial Resistance: Implications and Costs. Infect. Drug Resist. 2019, 12, 3903–3910. 10.2147/IDR.S234610. 31908502 PMC6929930

[ref32] Perez Ortiz G.; Sidda J. D.; de los Santos E. L. C.; Hubert C. B.; Barry S. M. *In Vitro* Elucidation of the Crucial but Complex Oxidative Tailoring Steps in Rufomycin Biosynthesis Enables One Pot Conversion of Rufomycin B to Rufomycin C. Chem. Commun. 2021, 57 (89), 11795–11798. 10.1039/D1CC04794A. PMC8577248 34676855

[ref33] Ma J.; Huang H.; Xie Y.; Liu Z.; Zhao J.; Zhang C.; Jia Y.; Zhang Y.; Zhang H.; Zhang T.; Ju J. Biosynthesis of Ilamycins Featuring Unusual Building Blocks and Engineered Production of Enhanced Anti-Tuberculosis Agents. Nat. Commun. 2017, 8 (1), 391 10.1038/s41467-017-00419-5. 28855504 PMC5577134

[ref34] Lambalot R. H.; Gehring A. M.; Flugel R. S.; Zuber P.; LaCelle M.; Marahiel M. A.; Reid R.; Khosla C.; Walsh C. T. A New Enzyme Superfamily—the Phosphopantetheinyl Transferases. Chem. Biol. 1996, 3 (11), 923–936. 10.1016/S1074-5521(96)90181-7. 8939709

[ref35] Sieber S. A.; Marahiel M. A. Learning from Nature’s Drug Factories: Nonribosomal Synthesisof MacrocyclicPeptides. J. Bacteriol. 2003, 185 (24), 7036–7043. 10.1128/JB.185.24.7036-7043.2003. 14645262 PMC296262

[ref36] Franke J.; Hertweck C. Biomimetic Thioesters as Probes for Enzymatic Assembly Lines: Synthesis, Applications, and Challenges. Cell Chem. Biol. 2016, 23 (10), 1179–1192. 10.1016/j.chembiol.2016.08.014. 27693058

[ref37] Camarero J. A.; Hackel B. J.; de Yoreo J. J.; Mitchell A. R. Fmoc-Based Synthesis of Peptide α-Thioesters Using an Aryl Hydrazine Support. J. Org. Chem. 2004, 69 (12), 4145–4151. 10.1021/jo040140h. 15176841

[ref38] Fang G.-M.; Li Y.-M.; Shen F.; Huang Y.-C.; Li J.-B.; Lin Y.; Cui H.-K.; Liu L. Protein Chemical Synthesis by Ligation of Peptide Hydrazides. Angew. Chem., Int. Ed. 2011, 50 (33), 7645–7649. 10.1002/anie.201100996. 21648030

[ref39] Flood D. T.; Hintzen J. C. J.; Bird M. J.; Cistrone P. A.; Chen J. S.; Dawson P. E. Leveraging the Knorr Pyrazole Synthesis for the Facile Generation of Thioester Surrogates for Use in Native Chemical Ligation. Angew. Chem., Int. Ed. 2018, 57 (36), 11634–11639. 10.1002/anie.201805191. PMC6126375 29908104

[ref40] Baran P. S.; Guerrero C. A.; Corey E. J. Short, Enantioselective Total Synthesis of Okaramine N. J. Am. Chem. Soc. 2003, 125 (19), 5628–5629. 10.1021/ja034491+. 12733890

[ref41] Luzung M. R.; Lewis C. A.; Baran P. S. Direct, Chemoselective N-Tert-Prenylation of Indoles by C—H Functionalization. Angew. Chem., Int. Ed. 2009, 48 (38), 7025–7029. 10.1002/anie.200902761. PMC2886517 19701955

[ref42] Zheng J.-S.; Tang S.; Qi Y.-K.; Wang Z.-P.; Liu L. Chemical Synthesis of Proteins Using Peptide Hydrazides as Thioester Surrogates. Nat. Protoc. 2013, 8 (12), 2483–2495. 10.1038/nprot.2013.152. 24232250

[ref43] Zhang L.; Tam J. P. Metal Ion-Assisted Peptide Cyclization. Tetrahedron Lett. 1997, 38 (25), 4375–4378. 10.1016/S0040-4039(97)00935-0.

[ref44] Zhang L.; Tam J. P. Lactone and Lactam Library Synthesis by Silver Ion-Assisted Orthogonal Cyclization of Unprotected Peptides. J. Am. Chem. Soc. 1999, 121 (14), 3311–3320. 10.1021/ja983859d.

[ref45] Tung C. L.; Wong C. T. T.; Li X. Peptide 2-Formylthiophenol Esters Do Not Proceed through a Ser/Thr Ligation Pathway, but Participate in a Peptide Aminolysis to Enable Peptide Condensation and Cyclization. Org. Biomol. Chem. 2015, 13 (25), 6922–6926. 10.1039/C5OB00825E. 26013965

[ref46] Li Y.; Yongye A.; Giulianotti M.; Martinez-Mayorga K.; Yu Y.; Houghten R. A. Synthesis of Cyclic Peptides through Direct Aminolysis of Peptide Thioesters Catalyzed by Imidazole in Aqueous Organic Solutions. J. Comb. Chem. 2009, 11 (6), 1066–1072. 10.1021/cc900100z. 19894764 PMC3121167

[ref47] Ohara T.; Kaneda M.; Saito T.; Fujii N.; Ohno H.; Oishi S. Head-to-Tail Macrocyclization of Cysteine-Free Peptides Using an o -Aminoanilide Linker. Bioorg. Med. Chem. Lett. 2018, 28 (8), 1283–1286. 10.1016/j.bmcl.2018.03.027. 29580681

[ref48] Zheng J.-S.; Tang S.; Guo Y.; Chang H.-N.; Liu L. Synthesis of Cyclic Peptides and Cyclic Proteins via Ligation of Peptide Hydrazides. ChemBioChem 2012, 13 (4), 542–546. 10.1002/cbic.201100580. 22302623

[ref49] Horsman M. E.; Hari T. P. A.; Boddy C. N. Polyketide Synthase and Non-Ribosomal Peptide Synthetase Thioesterase Selectivity: Logic Gate or a Victim of Fate?. Nat. Prod. Rep. 2016, 33 (2), 183–202. 10.1039/C4NP00148F. 25642666

[ref50] Mata A.; Weigl U.; Flögel O.; Baur P.; Hone C. A.; Kappe C. O. Acyl Azide Generation and Amide Bond Formation in Continuous-Flow for the Synthesis of Peptides. React. Chem. Eng. 2020, 5 (4), 645–650. 10.1039/D0RE00034E.

[ref51] Chatterjee J.; Laufer B.; Kessler H. Synthesis of N-Methylated Cyclic Peptides. Nat. Protoc. 2012, 7 (3), 432–444. 10.1038/nprot.2011.450. 22322216

[ref52] Huang Y.-C.; Fang G.-M.; Liu L. Chemical Synthesis of Proteins Using Hydrazide Intermediates. Natl. Sci. Rev. 2016, 3 (1), 107–116. 10.1093/nsr/nwv072.

[ref53] Blankenstein J.; Zhu J. Conformation-Directed Macrocyclization Reactions. Eur. J. Org. Chem. 2005, 2005 (10), 1949–1964. 10.1002/ejoc.200400751.

[ref54] Davies J. S. The Cyclization of Peptides and Depsipeptides. J. Pept. Sci. 2003, 9 (8), 471–501. 10.1002/psc.491. 12952390

[ref55] Agouridas V.; El Mahdi O.; Diemer V.; Cargoët M.; Monbaliu J.-C. M.; Melnyk O. Native Chemical Ligation and Extended Methods: Mechanisms, Catalysis, Scope, and Limitations. Chem. Rev. 2019, 119 (12), 7328–7443. 10.1021/acs.chemrev.8b00712. 31050890

[ref56] Huguenin-Dezot N.; Alonzo D. A.; Heberlig G. W.; Mahesh M.; Nguyen D. P.; Dornan M. H.; Boddy C. N.; Schmeing T. M.; Chin J. W. Trapping Biosynthetic Acyl-Enzyme Intermediates with Encoded 2,3-Diaminopropionic Acid. Nature 2019, 565 (7737), 112–117. 10.1038/s41586-018-0781-z. 30542153 PMC6436733

[ref57] Budruev A. V.; Sinjagina D. Yu. Reactions of Acyl Azides with Secondary Amines in the Presence of Copper(Ii) Acetate. Russ. Chem. Bull. 2013, 62 (6), 1366–1370. 10.1007/s11172-013-0194-y.

[ref58] Baumann E. Ueber eine einfache Methode der Darstellung von Benzoësäureäthern. Ber. Dtsch. Chem. Ges. 1886, 19 (2), 3218–3222. 10.1002/cber.188601902348.

[ref59] Schotten C. Ueber die Oxydation des Piperidins. Ber. Dtsch. Chem. Ges. 1884, 17 (2), 2544–2547. 10.1002/cber.188401702178.

[ref60] Grimsley G. R.; Scholtz J. M.; Pace C. N. A Summary of the Measured p *K* Values of the Ionizable Groups in Folded Proteins. Protein Sci. 2009, 18 (1), 247–251. 10.1002/pro.19. 19177368 PMC2708032

[ref61] Yudin A. K. Macrocycles: Lessons from the Distant Past, Recent Developments, and Future Directions. Chem. Sci. 2015, 6 (1), 30–49. 10.1039/C4SC03089C. 28553456 PMC5424464

[ref62] Schroeder C. I.; Smythe M. L.; Lewis R. J. Development of Small Molecules That Mimic the Binding of ω-Conotoxins at the N-Type Voltage-Gated Calcium Channel. Mol. Diversity 2004, 8 (2), 127–134. 10.1023/B:MODI.0000025656.79632.86. 15209164

[ref63] Fanelli R.; Jeanne-Julien L.; René A.; Martinez J.; Cavelier F. Stereoselective Synthesis of Unsaturated α-Amino Acids. Amino Acids 2015, 47 (6), 1107–1115. 10.1007/s00726-015-1934-0. 25715756

[ref64] Brady S. F.; Varga S. L.; Freidinger R. M.; Schwenk D. A.; Mendlowski M.; Holly F. W.; Veber D. F. Practical Synthesis of Cyclic Peptides, with an Example of Dependence of Cyclization Yield upon Linear Sequence. J. Org. Chem. 1979, 44 (18), 3101–3105. 10.1021/jo01332a003.

[ref65] Schultz A. W.; Oh D.-C.; Carney J. R.; Williamson R. T.; Udwary D. W.; Jensen P. R.; Gould S. J.; Fenical W.; Moore B. S. Biosynthesis and Structures of Cyclomarins and Cyclomarazines, Prenylated Cyclic Peptides of Marine Actinobacterial Origin. J. Am. Chem. Soc. 2008, 130 (13), 4507–4516. 10.1021/ja711188x. 18331040

[ref66] Little R. F.; Hertweck C. Chain Release Mechanisms in Polyketide and Non-Ribosomal Peptide Biosynthesis. Nat. Prod. Rep. 2022, 39 (1), 163–205. 10.1039/D1NP00035G. 34622896

[ref67] Bushley K. E.; Raja R.; Jaiswal P.; Cumbie J. S.; Nonogaki M.; Boyd A. E.; Owensby C. A.; Knaus B. J.; Elser J.; Miller D.; Di Y.; McPhail K. L.; Spatafora J. W. The Genome of Tolypocladium Inflatum: Evolution, Organization, and Expression of the Cyclosporin Biosynthetic Gene Cluster. PLoS Genet. 2013, 9 (6), e1003496 10.1371/journal.pgen.1003496. 23818858 PMC3688495

[ref68] Krätzschmar J.; Krause M.; Marahiel M. A. Gramicidin S Biosynthesis Operon Containing the Structural Genes grsA and grsB Has an Open Reading Frame Encoding a Protein Homologous to Fatty Acid Thioesterases. J. Bacteriol. 1989, 171 (10), 5422–5429. 10.1128/jb.171.10.5422-5429.1989. 2477357 PMC210379

[ref69] Mootz H. D.; Marahiel M. A. The Tyrocidine Biosynthesis Operon of Bacillus Brevis: Complete Nucleotide Sequence and Biochemical Characterization of Functional Internal Adenylation Domains. J. Bacteriol. 1997, 179 (21), 6843–6850. 10.1128/jb.179.21.6843-6850.1997. 9352938 PMC179617

[ref70] Bu X.; Wu X.; Xie G.; Guo Z. Synthesis of Tyrocidine A and Its Analogues by Spontaneous Cyclization in Aqueous Solution. Org. Lett. 2002, 4 (17), 2893–2895. 10.1021/ol0263191. 12182582

[ref71] Swierstra J.; Kapoerchan V.; Knijnenburg A.; Van Belkum A.; Overhand M. Structure, Toxicity and Antibiotic Activity of Gramicidin S and Derivatives. Eur. J. Clin. Microbiol. Infect. Dis. 2016, 35 (5), 763–769. 10.1007/s10096-016-2595-y. 26886453 PMC4840228

[ref72] Qin C.; Zhong X.; Bu X.; Ng N. L. J.; Guo Z. Dissociation of Antibacterial and Hemolytic Activities of an Amphipathic Peptide Antibiotic. J. Med. Chem. 2003, 46 (23), 4830–4833. 10.1021/jm0341352. 14584933

[ref73] Tambadou F.; Caradec T.; Gagez A.-L.; Bonnet A.; Sopéna V.; Bridiau N.; Thiéry V.; Didelot S.; Barthélémy C.; Chevrot R. Characterization of the Colistin (Polymyxin E1 and E2) Biosynthetic Gene Cluster. Arch. Microbiol. 2015, 197 (4), 521–532. 10.1007/s00203-015-1084-5. 25609230

[ref74] Slingerland C. J.; Wesseling C. M. J.; Innocenti P.; Westphal K. G. C.; Masereeuw R.; Martin N. I. Synthesis and Evaluation of Polymyxins Bearing Reductively Labile Disulfide-Linked Lipids. J. Med. Chem. 2022, 65 (23), 15878–15892. 10.1021/acs.jmedchem.2c01528. 36399613 PMC9743094

[ref75] Ramesh S.; Govender T.; Kruger H. G.; Albericio F.; De La Torre B. G. An Improved and Efficient Strategy for the Total Synthesis of a Colistin-like Peptide. Tetrahedron Lett. 2016, 57 (17), 1885–1888. 10.1016/j.tetlet.2016.03.055.

[ref76] Harris P. W. R.; Siow A.; Yang S.-H.; Wadsworth A. D.; Tan L.; Hermant Y.; Mao Y.; An C.; Hanna C. C.; Cameron A. J.; Allison J. R.; Chakraborty A.; Ferguson S. A.; Mros S.; Hards K.; Cook G. M.; Williamson D. A.; Carter G. P.; Chan S. T. S.; Painter G. A.; Sander V.; Davidson A. J.; Brimble M. A. Synthesis, Antibacterial Activity, and Nephrotoxicity of Polymyxin B Analogues Modified at Leu-7, d -Phe-6, and the N-Terminus Enabled by S-Lipidation. ACS Infect. Dis. 2022, 8 (12), 2413–2429. 10.1021/acsinfecdis.1c00347. 36413173

[ref77] Feng Y.; Liang S.; Langille J.; Pierce B. S.; Chung S.; Szeliga J.; Wilcox G.; Simonds P.; Farley K. A.; Li B.; Garcia-Irizarry C.; Jones P.; Lira R. Improved Synthesis of a Macrocyclic Peptide-Like C5aR Antagonist for Intravenous Applications. Org. Process Res. Dev. 2023, 27 (11), 2010–2019. 10.1021/acs.oprd.3c00202.

[ref78] Zhang S.; Amso Z.; De Leon Rodriguez L. M.; Kaur H.; Brimble M. A. Synthesis of Natural Cyclopentapeptides Isolated from *Dianthus Chinensis*. J. Nat. Prod. 2016, 79 (7), 1769–1774. 10.1021/acs.jnatprod.6b00152. 27326468

[ref79] Maharani R.; Yayat H. N.; Hidayat A. T.; Al Anshori J.; Sumiarsa D.; Farabi K.; Mayanti T.; Nurlelasari; Harneti D.; Supratman U. Synthesis of a Cyclooctapeptide, Cyclopurpuracin, and Evaluation of Its Antimicrobial Activity. Molecules 2023, 28 (12), 4779 10.3390/molecules28124779. 37375334 PMC10301653

[ref80] Baumann H. I.; Keller S.; Wolter F. E.; Nicholson G. J.; Jung G.; Süssmuth R. D.; Jüttner F. Planktocyclin, a Cyclooctapeptide Protease Inhibitor Produced by the Freshwater Cyanobacterium *Planktothrix Rubescens*. J. Nat. Prod. 2007, 70 (10), 1611–1615. 10.1021/np0700873. 17935298

[ref81] Qin C.; Bu X.; Wu X.; Guo Z. A Chemical Approach to Generate Molecular Diversity Based on the Scaffold of Cyclic Decapeptide Antibiotic Tyrocidine A. J. Comb. Chem. 2003, 5 (4), 353–355. 10.1021/cc0300255. 12857101

[ref82] Bozhüyük K. A.; Präve L.; Kegler C.; Schenk L.; Kaiser S.; Schelhas C.; Shi Y.-N.; Kuttenlochner W.; Schreiber M.; Kandler J.; Alanjary M.; Mohiuddin T. M.; Groll M.; Hochberg G. K. A.; Bode H. B. Evolution-Inspired Engineering of Nonribosomal Peptide Synthetases. Science 2024, 383 (6689), eadg4320 10.1126/science.adg4320. 38513038

[ref83] Kries H. Biosynthetic Engineering of Nonribosomal Peptide Synthetases. J. Pept. Sci. 2016, 22 (9), 564–570. 10.1002/psc.2907. 27465074

[ref84] Wang Y.; He J.; Alam M. S.; Wang F.; Shang Z.; Chen Y.; Sun C.; Lu Z.; Gao Y.; Zhang T.; Ju J.; Ma J. Efficient Mutasynthesis of “Non-Natural” Antitubercular Ilamycins with Low Cytotoxicity. ACS Synth. Biol. 2024, 13, 930–941. 10.1021/acssynbio.3c00730.

