# Supplementary material for: Rapid Peptide Cyclization Inspired by the Modular Logic of Nonribosomal Peptide Synthetases
Source: J Am Chem Soc. 2024 Jun 6;146(24):16787–801. doi: 10.1021/jacs.4c04711 (PMC11191687; doi:10.1021/jacs.4c04711)
Supplement: Supplementary file 1 — ja4c04711_si_001.pdf [file ja4c04711_si_001.pdf]

Supplementary information for:

Rapid Peptide Cyclization Inspired by the Modular Logic of Nonribosomal Peptide Synthetases

Yaoyu Ding[a], Edward Lambden[a], Jessica Peate[a‡], Lewis J. Picken[a‡], Thomas W. Rees[b‡], Gustavo Perez-Ortiz[a], Sophie A. Newgas[a], Lucy A. R. Spicer[a], Thomas Hicks[a], Jeannine Hess[a,b], Martin B. Ulmschneider[a], Manuel M. Müller[a], Sarah M. Barry[a*]

[a] *Department of Chemistry, Faculty of Natural, Mathematical and Engineering Sciences, King's College London, Britannia House, 7 Trinity Street, London, SE1 1DB, UK*
[b] *The Francis Crick Institute, 1 Midland Road, London, NW1 1AT, UK*

* To whom correspondence should be addressed
Email: sarah.barry@kcl.ac.uk

Table of Contents

1. General Materials **4**

2. Supplementary Methods **5**

2.1 Synthesis of Non-proteinogenic Amino Acids **5**

2.1.1 Synthesis of tert-butyl 2-((diphenylmethylene)amino) acetate 2 5
2.1.2 Synthesis of tert-butyl (S,E)-2-((diphenylmethylene)amino)hex-4-enoate 3 6
2.1.3 Synthesis of tert-butyl (S,E)-2-((((9H-fluoren-9-yl)methoxy)carbonyl)amino)hex-4-enoate S1 7
2.1.4 Synthesis of tert-butyl (E)-2-((((9H-fluoren-9-yl)methoxy)carbonyl)amino)hex-4-enoate S3 8
2.1.5 Synthesis of (S,E)-2-((((9H-fluoren-9-yl)methoxy)carbonyl)amino)hex-4-enoic acid 4 9
2.1.6 Synthesis of methyl N^{α}-(tert-butoxycarbonyl)-1-(2-methylbut-3-en-2-yl)-*L*-tryptophanate 6 10
2.1.7 Synthesis of N^{α}-(((9H-fluoren-9-yl) methoxy) carbonyl)-1-(2-methylbut-3-en-2-yl)-*L*-tryptophan 7 11

2.2 General Procedure for Solid Phase Synthesis of Linear Rufomycin Peptides and Derivatives13

2.2.1 General procedure of hydrazine functionalizing 2-chlorotrityl resin 13
2.2.2 General manual peptide coupling method and Fmoc deprotection (50 mg resin scale) 14
2.2.3 General automatic peptide coupling method and Fmoc deprotection (200~300 mg resin scale) 14
2.2.4 General peptide cleavage from resin 14
2.2.5 General SNAC peptide synthesis 15

2.3 Reverse-Phase Chromatography Methods **15**

2.3.1 Analytical HPLC 16
2.3.2 Semi-preparative HPLC 16
2.3.3 Preparative HPLC 17
2.3.4 UPLC-HRMS 18

2.4 Chemical Peptide Cyclization **19**

2.4.1 Silver assisted peptide cyclization 19
2.4.2 General procedure for bioinspired peptide cyclization (analytical scale) 19
2.4.3 General Procedure for bioinspired peptide cyclization (semi-prep scale) 20

2.5 Molecular Dynamic (MD) Simulation Methods **47**

2.6 Antibacterial Assays **54**

3. Supplementary Characterization Data **80**

3.1 ^{1}H and ^{13}C NMR spectra **80**

3.2 High Resolution Mass Spectra and Fragmentation of Peptides **146**

4. Supplementary References **231**

Figure S1. Synthesis scheme for non-proteinogenic amino acids 5

Figure S2. General synthesis of peptide hydrazide 13

Figure S3. Chemical cyclization of linear peptide 19

Figure S4. Chemical cyclization of natural product rufomycin B **8c** 20

Figure S5. Peptide backbone dihedrals analysis 48

Figure S6. Molecular dynamics simulation of peptide **S9** 49

Figure S7. Molecular dynamics simulation of peptide **S14** 50

Figure S8. Molecular dynamics simulation of peptide **S15** 51

Figure S9. Molecular dynamics simulation of peptide **S16** 53

Figure S10. Biosynthesis of rufomycins 55

Figure S11. Linear peptide library of rufomycin derivatives to probe chemical cyclization 56

Figure S12. Fmoc-3-nitro-*L*-tyrosine calibration curve 57

Figure S13. Silver-assisted SNAC peptide cyclization 57

Figure S14. HPLC analysis of chemical cyclization of **9a** 58

Figure S15. Chemical cyclization product distribution as a function reaction conditions 59

Figure S16. Effect of backbone *N*-methylation on chemical cyclisation 60

Figure S17. Investigation of the effects of reaction components on cyclisation reaction outcome 61

Figure S18. Macrocyclization of **10c** starting from different points of cyclisation 62

Figure S19. HRMS analysis of cyclic peptide **8c** rufomycin B 63

Figure S20. Chiral HPLC analysis of synthesized amino acid **S1** and **S3** 64

Figure S21. HPLC analysis (214 nm) of peptide cyclization of peptides **19a**, **20a**, **21a** and **22a** 65

Figure S22. HPLC analysis (214 nm) of peptide cyclization of **25a** and **26a** 66

Figure S23. HPLC analysis (214 nm) of peptide cyclization of **27a** and **28a** 67

Figure S24. HPLC analysis (214 nm) of peptide cyclization of **29a** and **31a** 68

Figure S25. HPLC analysis (214 nm) of peptide cyclization of **30a** 69

Figure S26. Comparison of H-NMR and MS^E spectra of colistin analogs 71

Figure S27. Effect of solvent conditions on cyclization reaction outcome HPLC analysis (214 nm) of peptide cyclization of **32a** 72

Figure S28. HPLC analysis (214 nm) of peptide cyclization of **33a** and **34a** 74

Figure S29. HPLC analysis (214 nm) of analytical scale (2.2 mM in 1 mL buffer) peptide cyclization **10b** 75

Figure S30. HPLC analysis (214 nm) of prep scale (8 mM in 40 mL buffer) peptide cyclization **18a**.. 76

Table S1. Antimycobacterial activities of peptides **25c**, **26c** and **29c**. 54

Table S2. HRMS data of synthesised hydrazide peptides **(a)** and cyclic peptides **(c)**. 77

Scheme S1: Comparison of NMR and CD of peptide **9a** and peptide **14a**. 46

1. General Materials

All the reagents and solvents were obtained from commercial suppliers and were used without further purification. Dry reactions were conducted in oven-dried glassware under a nitrogen atmosphere. Anhydrous THF, MeOH and DCM were obtained from the Inert Solvent Purification System, all other solvents were supplied as Sureseal® bottles by Sigma Aldrich, DMF (sequencing grade) was purchased from Cambridge Reagent Ltd used for Solid Phase Peptide Synthesis (SPPS). The following Fmoc-protected amino acids were used for SPPS: Fmoc-*L*-Dab(Boc)-OH, Fmoc-*L*-Thr(*t*Bu)-OH, Fmoc-*L*-Lys(Boc)-OH, Fmoc-*L*-Orn(Boc)-OH, Fmoc-*L*-Tyr(*t*Bu)-OH, Fmoc-*L*-Ser(*t*Bu)-OH, Fmoc-*L*-Trp(Boc)-OH, Fmoc-*L*-Gln(Trt)-OH, Fmoc-*L*-Arg(Pbf)-OH and Fmoc-*L*-Asn(Trt)-OH. Nuclear Magnetic Resonance (NMR) spectra were recorded using a Bruker UltraShieldTM 400 MHz or 700 MHz and at default temperature (18 °C). The chemical shifts (δ) are reported in parts per million (ppm) using the abbreviations: s, singlet; d, doublet; dd, double of doublet; t, triplet; q, quartet. Resonances that could not be easily interpreted were designated multiplets (m). Chemical shifts (δ) are referenced to the residual solvent signal. Spin-spin coupling constants *J* are quoted in Hz. Flash column chromatography was performed using 60 Å (40-64 micron) silica and solvent mixtures of hexane and ethyl acetate or dichloromethane and methanol. Analytical thin layer chromatography was performed on TLC Silica gel 60 F_{254} (Aluminium sheets). Visualization was assisted with 254 nm UV-lamp, potassium permanganate ($KMnO_4$) stain and ninhydrin stain. High-resolution mass spectra were recorded on a Waters Acquity UPLC-Class I equipped with an ACQUITY UPLC column. The detector was a Waters Xevo-G2-XS QTof with electrospray ionization source. The instrument was operated in positive mode full scan with detection window set from 50 to 2000 Da. For peptide fragmentation, a collision energy ramp from 15 V to 40 V was employed. Optical rotation readings were recorded using Anton Parr MCP100 Polarimeter. Specific rotations ($[\alpha]^{25}_{D}$) were recorded at the sodium D line (589 nm) in methanol or chloroform and are quoted in: deg cm^2 g^{-1}. Solution concentration (c) are given in 0.1. Temperatures are in degrees Celsius (°C). The prefixes (+) and (-) indicate the sign of the optical rotation. Circular Dichroism spectra were recorded on a Chirascan V100 CD at 25 °C. Bacterial strains *Escherichia coli* K12 (NCTC 10538), *Staphylococcus aureus* NCTC 12973, *Bacillus subtilis* NCTC 06276, and *Enterococcus faecium* NCTC 7171, were purchased from the UKHSA

Culture Collections as freeze-dried samples. The bacteria were resuspended and kept at -80 °C in 20% glycerol. The optical density at 600 nm (OD_{600}) was measured on a BMG Labtech CLARIOstar plate reader. The *E. coli* cartoon in Fig. 2 was created from BioRender scientific illustration software and exported under a paid subscription. Analytical chemical cyclization was carried out in Biotage® microwave reaction vial (2.0 – 5.0 mL).

2. Supplementary Methods

2.1 Synthesis of Non-proteinogenic Amino Acids

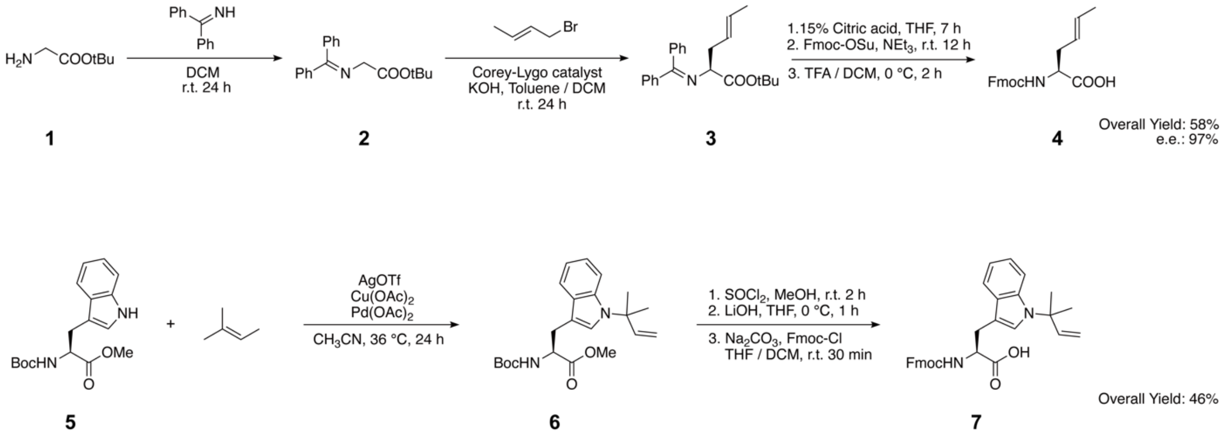

Figure S1. Synthesis scheme for non-proteinogenic amino acids. Synthesis of *N*-Fmoc *trans*-crotyl glycine **4** and *N*-Fmoc prenylated *L*-tryptophan **7** for solid phase peptide synthesis.

2.1.1 Synthesis of tert-butyl 2-((diphenylmethylene)amino) acetate 2

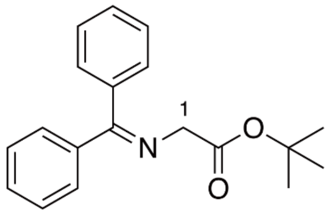

2

To a stirred suspension of glycine *tert*-butyl ester hydrochloride **1** (1.8 g, 10.73 mmol, 1 equiv.) in dichloromethane (43 mL), benzophenone imine (1.946 g, 10.73 mmol, 1 equiv.) was added and the mixture left to stir at room temperature for 24 h. The organic layer was then washed with water (2 x 20 mL), dried over magnesium sulfate and concentrated *in vacuo* to give the crude title compound **2** as a white solid in quantitative yield. The crude product was directly used for the next step without any purification.

^{1}H NMR ($CDCl_3$, 400 MHz): δ 7.68 (d, *J* = 7.0 Hz, 2H, *ortho-H*), 7.47 – 7.40 (m, 3H, *ortho-H* & *para-H*), 7.39 – 7.34 (m, 1H, *para-H*) 7.31 (dd, *J* = 8.3, 6.5Hz, 2H, *meta-H*), 7.20 – 7.15 (m, 2H, *meta-H*), 4.14 (s, 2H, CH_2-C1), 1.47 (s, 9H, H_{tBu}).
^{13}C NMR ($CDCl_3$, 101 MHz): δ 171.33 (C-C_{imine}), 169.68 (C-$C_{carbonyl}$), 139.26 (C-C_{Ar}), 136.04 (C-C_{Ar}), 130.25 (CH-C_{para}), 128.64 (CH-C_{para}), 128.63 (CH-C_{ortho}), 128.51 (CH-C_{ortho}), 127.91 (CH-C_{meta}), 127.57 (CH-C_{meta}), 80.83 (C-C_{tBu}), 56.20 (C1), 27.99 (CH_3-C_{tBu}).
HRMS (ESI^+) calc. for $C_{19}H_{21}NO_2$ calc. for $[M+H]^+$: 296.1650 m/z, found: 296.1654 m/z. (Δ = 1.35 ppm)
The spectroscopic data agree with those reported in the literature.[1]

2.1.2 Synthesis of tert-butyl (S,E)-2-((diphenylmethylene)amino)hex-4-enoate 3

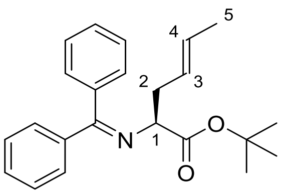

3

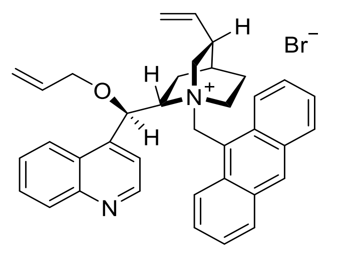

Corey Lygo catalyst

To a stirred suspension of intermediate **2** (200 mg, 0.68 mmol, 1 equiv.) and Corey-Lygo catalyst *O*-allyl-*N*-(9-anthracenylmethyl) cinchonidinium bromide (41 mg, 0.068 mmol, 10 mol%.) in 3.4 mL of toluene/ dichloromethane (v/v, 7:3), a solution of 9M potassium hydroxide (1.96 mL, 17.64 mmol, 26 equiv.) was added. The mixture was cooled to 0 °C and crotyl bromide (85% purity, mixture of *trans, cis 5:1*) (110 mg, 0.816 mmol, 1.2 equiv.) was slowly added. The reaction mixture was vigorously stirred for 24 h at 0 °C. After concentration, the residue was diluted in water (30 mL) and diethyl ether (30 mL). The aqueous layer was then extracted with diethyl ether (3 × 45 mL), and the organic layer was washed with water (2 × 45 mL), dried over magnesium sulfate and concentrated to give the title compound **3** as a pale-yellow oil in quantitative yield, the crude was directly used for the next step without any purification.
^{1}H NMR ($CDCl_3$, 400 MHz): δ 7.66 (d, *J* = 8.0 Hz, 2H, *ortho*-H), 7.48 – 7.41 (m, 3H, *ortho*-H and *para*-H), 7.40 – 7.35 (m, 1H, *para*-H), 7.36 – 7.29 (m, 2H, *meta*-H), 7.22 – 7.13 (m, 2H, *meta*-H), 5.57 – 5.43 (m, 1H, CH-C4), 5.34 (m, 1H, CH-C3), 4.05 – 3.80 (m, 1H, CH-C1), 2.58 (m, 2H, CH_2-C2), 1.62 (d, *J* = 6.32 Hz, 3H, CH_3-C5), 1.45 (s, 9H, H_{tBu}).

^{13}C NMR ($CDCl_3$, 101 MHz): δ 171.20 ($C\text{-}C_{carbonyl}$), 169.90 ($C\text{-}C_{imine}$), 139.91 (C_{Ar}), 136.89 (C_{Ar}), 130.21 (C_{Ar}), 129.13 (C_{Ar}), 128.90 (C_{Ar}), 128.55 (C_{Ar}), 128.44 (C_{Ar}), 128.33 (C_{Ar}), 128.07 (C_{Ar}), 128.02 (C_{Ar}), 127.90(C4), 127.19 (C3), 80.92 ($C\text{-}C_{tBu}$), 66.49 (C1), 37.10 (C2), 28.19 ($\underline{C}H_3\text{-}C_{tBu}$), 18.09 (C5).
HRMS (ESI^+) calc. for $C_{23}H_{27}NO_2$ calc. for $[M+H]^+$: 350.2120 m/z, found: 350.2122 m/z. (Δ = 0.58 ppm)
$[\alpha]_D^{25}$ = - 59.5 (*c* 1.0, $CHCl_3$)

2.1.3 Synthesis of tert-butyl (S,E)-2-((((9H-fluoren-9-yl)methoxy)carbonyl)amino)hex-4-enoate S1

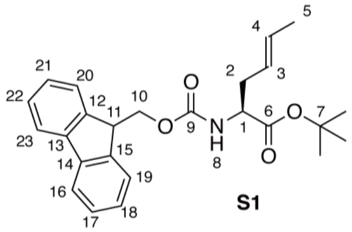

S1

To a solution of intermediate **3** (267 mg, 0.7 mmol, 1 equiv.) in tetrahydrofuran (1.4 mL) was added a solution of 15% citric acid (4.2 mL). The mixture was stirred at room temperature for 7 h. Then the THF was evaporated, and the residue was washed with diethyl ether (3 x 10 mL). The pH of aqueous solution was increased to 9 with 1M sodium carbonate before extracted with ethyl acetate (4 x 15 mL). The combined organic phases were concentrated at room temperature *in vacuo*. The residue and Fmoc-OSu (306 mg, 0.91 mmol, 1.3 equiv.) were dissolved in ethyl acetate (3.5 mL) then triethylamine (213 mg, 2.1 mmol, 3 equiv.) was added. The reaction mixture was stirred at room temperature overnight. The mixture was then washed with saturated sodium bicarbonate (3 x 15 mL). The organic phase was washed with brine (20 mL), dried over magnesium sulfate, filtered and concentrated *in vacuo*. Purification of the residue by flash column chromatography on silica gel (hexane : ethyl acetate, 6:1) afforded the title compound **S1** (181 mg, 64%, *E* : *Z* = 4 : 1, 97% e.e.) as a colorless oil. (Supplementary Fig. S20)
^{1}H NMR ($CDCl_3$, 400 MHz): δ 7.77 (d, J = 7.5 Hz, 2H, H_{Ar}), 7.62 (dd, J = 7.5, 2.9 Hz, 2H, H_{Ar}), 7.41 (t, J = 7.5 Hz, 2H, H_{Ar}), 7.32 (t, J = 7.4 Hz, 2H, H_{Ar}), 5.74 – 5.50 (m, 1H, CH-C4), 5.42 (d, J = 8.1 Hz, 1H, N*H*), 5.49 – 5.30 (m, 1H, CH-C3), 4.51 – 4.30 (m, 3H, CH-C1 & CH_2-C10), 4.25 (t, J = 7.2 Hz, 1H, CH-C11), 2.73 – 2.42 (m, 2H, CH_2-C2), 1.66 (d, J = 6.7 Hz, 3H, CH_3-C5), 1.48 (s, 9H, H_{tBu}).

^{13}**C NMR** ($CDCl_3$, 101 MHz): δ 171.08 (C6), 155.72 (C9), 144.02 & 143.93 (C12&C15), 141.35 (C13&14), 129.87 (C4), 127.74 (C_{Ar}), 127.10 (C_{Ar}), 125.22 (C_{Ar}), 125.20 (C_{Ar}), 124.64 (C3), 120.03 (C_{Ar}), 82.09 (C7), 66.98 (C1), 54.02 (C10), 47.24 (C11), 35.86 (C2), 28.11 ($\underline{C}H_3$-C_{tBu}), 18.08 (C5).
HRMS (ESI^+) calc. for $C_{25}H_{29}NO_4$ calc. for $[M+H]^+$: 408.2175 m/z, found: 408.2173 m/z (Δ = -0.5 ppm)
HPLC (AD-H chiral column, hexane (5%)-isopropyl alcohol isocratic): 27.889 min. (see Fig. S20)

2.1.4 Synthesis of tert-butyl (E)-2-((((9H-fluoren-9-yl)methoxy)carbonyl)amino)hex-4-enoate S3

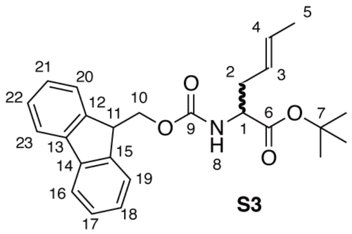

Synthesis of racemic mixture for chiral HPLC comparison.

To a stirred suspension of intermediate **2** (100 mg, 0.34 mmol, 1 equiv.) in dry tetrahydrofuran (2 mL) at -78 °C, lithium diisopropylamide (2 M in THF) (0.25 mL, 0.51 mmol, 1.5 equiv.) was slowly dropwise added, then the mixture was incubated for 30 min, crotyl bromide (85% mixture of *cis* & *trans*) (55 mg, 0.41 mmol, 1.2 equiv.) was added. The reaction mixture was stirred for 12 h at -78 °C. The reaction was quenched with 2 mL of water, then tetrahydrofuran was evaporated, the residue was diluted in diethyl ether (5 mL). The aqueous layer was then extracted with diethyl ether (3 × 5 mL), combined organic layer dried over magnesium sulfate and concentrated. Then the resulting crude mixture **S2** was directly used to prepare **S3** with the same procedure as synthesis of **S1**. (Yield **S3** 78% over 3 steps)
^{1}**H NMR** ($CDCl_3$, 400 MHz): δ 7.77 (d, J = 7.5 Hz, 2H, H_{Ar}), 7.61 (dd, J = 7.5, 2.9 Hz, 2H, H_{Ar}), 7.41 (t, J = 7.5 Hz, 2H, H_{Ar}), 7.32 (t, J = 7.4 Hz, 2H, H_{Ar}), 5.60 - 5.51 (m, 1H, CH-C4), 5.45 – 5.24 (m, 2H, CH-C3 & N*H*), 4.52 – 4.28 (m, 3H, CH_2-C10 & CH-C1), 4.24 (t, J = 7.2 Hz, 1H, CH-C11), 2.50 (m, 2H, CH_2-C2), 1.67 (d, J = 6.6 Hz, 3H, CH_3-C5), 1.48 (s, 9H, H_{tBu}).

^{13}C NMR ($CDCl_3$, 101 MHz): δ 171.12 (C6), 155.75 (C9), 144.07 (C_{Ar}), 141.40 (C_{Ar}), 129.96 (C4), 127.79 (C_{Ar}), 127.15 (C_{Ar}), 125.24 (C_{Ar}), 124.64 (C3), 120.08 (C_{Ar}), 82.16 (C7), 67.03 (C1), 54.04 (C10), 47.29 (C11), 35.91 (C2), 28.19 ($\underline{C}H_3$-C_{tBu}), 18.13 (C5).
HRMS (ESI^+) calc. for $C_{25}H_{29}NO_4$ calc. for $[M+H]^+$: 408.2175 m/z, found: 408.2173 m/z (Δ = -0.5 ppm)

2.1.5 Synthesis of (S,E)-2-((((9H-fluoren-9-yl)methoxy)carbonyl)amino)hex-4-enoic acid 4

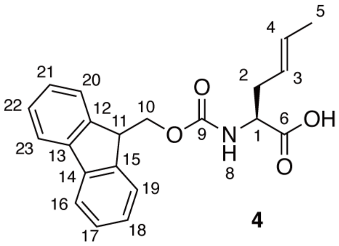

4

To a solution of intermediate **S1** (363 mg, 0.89 mmol, 1 equiv.) in dichloromethane (5 mL) at 0 °C, trifluoroacetic acid (3.4 mL) was slowly added. The resulting mixture was stirred at 0 °C and monitored by thin layer chromatography (hexane : ethyl acetate, 6:1). Once all the starting material consumed, the mixture was concentrated *in vacuo* to afford the title product **4** as a white solid in quantitative yield. The crude was directly used for the solid phase peptide synthesis without any purification.

^{1}H NMR (CD_3OD, 400 MHz): δ 7.76 (d, J = 7.8 Hz, 2H, H_{Ar}), 7.64 (dd, J = 7.4, 4.7 Hz, 2H, H_{Ar}), 7.37 (t, J = 7.5 Hz, 2H, H_{Ar}), 7.29 (td, J = 7.4, 1.3 Hz, 2H, H_{Ar}), 5.62 – 5.52 (m, 1H, CH-C4), 5.47 – 5.28 (m, 1H, CH-C3), 4.40 – 4.26 (m, 2H, CH_2-C10), 4.23 – 4.12 (m, 2H, CH-C1&CH-C11), 2.65 – 2.44 (m, 1H, CH-C2), 2.42 – 2.26 (m, 1H, CH-C2), 1.66 – 1.58 (d, J = 6.4 Hz, 3H, CH_3-C5).

^{13}C NMR (CD_3OD, 101 MHz): δ 175.57 (C6), 158.43 (C9), 145.19 (C_{Ar}), 142.54 (C_{Ar}), 129.86 (C4), 128.74 (C_{Ar}), 128.12 (C_{Ar}), 127.00 (C3), 126.22 (C_{Ar}), 120.88 (C_{Ar}), 67.97 (C1), 55.62 (C10), 48.36 (C11), 35.97 (C2), 18.13 (C5).

HRMS (ESI^+) calc. for $C_{21}H_{21}NO_4$ calc. for $[M+H]^+$: 352.1549 m/z, found: 352.1543 m/z (Δ = -1.7 ppm)

$[\alpha]_D^{25}$ = + 4.08 (c 1.0, $CHCl_3$)

2.1.6 Synthesis of methyl N$^{\alpha}$-(tert-butoxycarbonyl)-1-(2-methylbut-3-en-2-yl)-*L*-tryptophanate 6

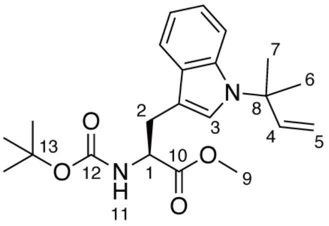

6

To a stirred suspension of methyl (*tert*-butoxycarbonyl)-*L*-tryptophanate **5** (200 mg, 0.63 mmol, 1 equiv.), copper (II) acetate (229 mg, 1.26 mmol, 2 equiv.), silver trifluoromethanesulfonate (324 mg, 1.26 mmol, 2 equiv.) and palladium (II) acetate (14 mg, 0.063 mmol, 10 mol%.) in dry acetonitrile (30 mL), 2-methyl-2-butene (133 mg, 1.89 mmol, 3 equiv.) was added at 36 °C. Then followed by three sequential additions of palladium (II) acetate (10 mol% after each hour). The resulting mixture was stirred at 36 °C for 24 h. The mixture then concentrated *in vacuo* and directly purified by flash column chromatography on silica gel (hexane : ethyl acetate, 6:1) afforded the title compound **6** (136 mg, 59%) as a yellowish oil.

^{1}H NMR ($CDCl_3$, 400 MHz): δ 7.52 (ddd, *J* = 18.3, 7.3, 2.0 Hz, 2H, H_{Ar}), 7.15 – 7.05 (m, 3H, CH-C3&C_{Ar}), 6.14 (dd, *J* = 17.5, 10.7 Hz, 1H, CH-C4), 5.26 – 5.10 (m, 2H, CH_2-C5), 5.07 (d, *J* = 8.4 Hz, 1H, N*H*), 4.66 (dt, *J* = 8.8, 5.8 Hz, 1H, CH-C1), 3.68 (s, 3H, CH_3-C9), 3.38 – 3.16 (m, 2H, CH_2-C2), 1.73 (s, 6H, CH_3-C6&C7), 1.44 (s, 9H, H_{Boc}).

^{13}C NMR ($CDCl_3$, 101 MHz): δ 172.97 (C10), 155.33 (C12), 144.21 (C4), 135.65 (C_{Ar}), 129.77 (C_{Ar}), 123.92 (C3), 120.98 (C_{Ar}), 119.06 (C_{Ar}), 118.93 (C_{Ar}), 113.89 (C_{Ar}), 113.61 (C5), 108.26 (C_{Ar}), 79.85 (C13), 59.10 (C8), 54.47 (C1), 52.24 (C9), 28.47 ($\underline{C}H_3$-C_{Boc}), 28.29 ($\underline{C}H_3$-C6 & C7), 28.05 (C2).

HRMS (ESI^+) calc. for $C_{22}H_{30}N_2O_4$ calc. for $[M+H]^+$: 387.2284 m/z, found: 387.2281 m/z. (*Δ* = - 0.77 ppm)

2.1.7 Synthesis of N$^{\alpha}$-(((9H-fluoren-9-yl) methoxy) carbonyl)-1-(2-methylbut-3-en-2-yl)-*L*-tryptophan 7

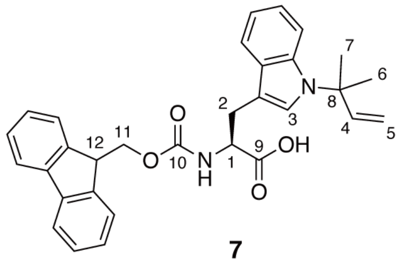

7

To a solution of intermediate **6** (211 mg, 0.546 mmol, 1 equiv.) in dry methanol (4 mL), thionyl chloride (0.09 mL, 1.092 mmol, 2 equiv.) was slowly added at 0 °C. The solution was gently heated up to 50 °C, then left for 2 h. The mixture was concentrated *in vacuo* then diluted with dichloromethane and water (10 mL each) and adjust to pH 12 with 1M sodium hydroxide. The organic layer was separated, washed with water and brine (15 mL each), dried over magnesium sulfate, filtered and the solvent was removed *in vacuo*. The residue was then dissolved in tetrahydrofuran (26 mL), 1M lithium hydroxide (8.2 mL, 8.2 mmol, 15 equiv.) was added over 3 min at 0 °C. The mixture was left for 2 h, then tetrahydrofuran was evaporated. The residues were suspended in dichloromethane (19 mL) and 1 M sodium carbonate (19 mL), followed by addition of Fmoc-Cl (fluorenylmethyloxycarbonyl chloride) (155 mg, 0.6 mmol, 1.1 equiv.). The resulting mixture was stirred vigorously for 15 min, then was acidified to pH 4~5 using sulfuric acid. Then dichloromethane was evaporated, the residues were extracted with ethyl acetate (3 x 30 mL), the combined organic phase washed with water (100 mL) and brine (100 mL), then dried over magnesium sulfate, filtered and concentrated *in vacuo* to afford the title compound **7** as a brown solid in quantitative yield. The crude was directly used for the solid phase peptide synthesis without any purification. The NMR was obtained from pure sample, which is purified flash column chromatography on silica gel (50% hexane of ethyl acetate to 100% ethyl acetate) afforded the title compound **7** as white powder.

^{1}H NMR (CD_3OD, 400 MHz) δ 7.75 (d, *J* = 7.6 Hz, 2H, $H_{Ar\text{-}Fmoc}$), 7.61 – 7.57 (m, 1H, $H_{Ar\text{-}Indole}$), 7.55 (d, *J* = 7.6 Hz, 2H, $H_{Ar\text{-}Fmoc}$), 7.47 – 7.42 (m, 1H, $H_{Ar\text{-}Indole}$), 7.34 (t, *J* = 7.1 Hz, 2H, $H_{Ar\text{-}Fmoc}$), 7.26 (s, 1H, CH-C3), 7.25 – 7.17 (m, 2H, $H_{Ar\text{-}Fmoc}$), 7.08 – 6.94 (m, 2H, $H_{Ar\text{-}Indole}$), 6.08 (dd, *J* = 17.5, 10.7 Hz, 1H, CH-C4), 5.16 – 5.01 (m, 2H, CH_2-C5), 4.51 (dd, *J* = 8.8, 4.7 Hz, 1H, CH-C1), 4.30 – 4.15 (m, 2H, CH_2-C11), 4.11 (t, *J* =

7.0 Hz, 1H, CH-C12), 3.36 (dd, J = 14.8, 5.0 Hz, 1H, CH-C2), 3.13 (dd, J = 14.7, 9.0 Hz, 1H, CH-C2), 1.66 (s, 6H, CH_3-C6&C7) ppm.

^{13}C NMR (CD_3OD, 101 MHz) δ 175.66 (C9), 158.43 (C10), 145.52 (C4), 145.19 ($C_{Ar\text{-}Fmoc}$), 145.16 ($C_{Ar\text{-}Fmoc}$), 142.48 ($C_{Ar\text{-}Fmoc}$), 136.98 ($C_{Ar\text{-}Indole}$), 130.80 ($C_{Ar\text{-}Indole}$), 128.73 ($C_{Ar\text{-}Fmoc}$), 128.15 ($C_{Ar\text{-}Fmoc}$), 126.28 ($C_{Ar\text{-}Fmoc}$), 126.21($C_{Ar\text{-}Fmoc}$), 125.24 (C3), 121.62 ($C_{Ar\text{-}Indole}$), 120.87 ($C_{Ar\text{-}Fmoc}$), 119.82 ($C_{Ar\text{-}Indole}$), 119.58 ($C_{Ar\text{-}Indole}$), 114.96 ($C_{Ar\text{-}Indole}$), 113.82 (C5), 110.13 ($C_{Ar\text{-}Indole}$), 68.04 (C11), 60.02 (C8), 56.25 (C1), 48.28 (C12), 28.68 (C2), 28.32 (C6 & C7).

HRMS (ESI^+) calc. for $C_{31}H_{30}N_2O_4$ calc. for $[M+H]^+$: 495.2284 m/z, found: 495.2272 m/z. (Δ = -2.4 ppm)

2.2 General Procedure for Solid Phase Synthesis of Linear Rufomycin Peptides and Derivatives

Solid phase peptide synthesis was performed either manually in a Bio-Rad Poly-Prep® polypropylene column with nitrogen swelling or on a Biotage Initiator+ Alstra peptide synthesizer at room temperature, using standard Fluorenylmethoxycarbonyl (Fmoc) strategy.

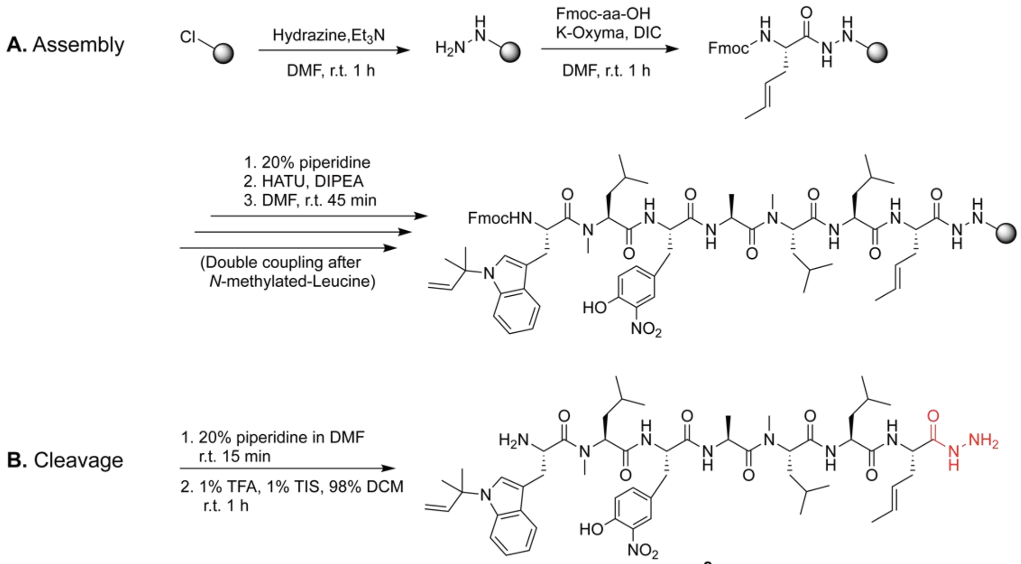

Figure S2. General peptide hydrazide synthesis. Exemplified by native linear rufomycin peptide **8**.

2.2.1 General procedure of hydrazine functionalizing 2-chlorotrityl resin

2-chlorotrityl chloride resin (substitution: 1.06 mmol/g) (200 mg, 0.2 mmol, 1 equiv.) were gently stirred in dimethylformamide (DMF) (1 mL) at 0 °C for 10 min. A mixture of triethylamine (84 µL, 0.6 mmol, 3 equiv.) and hydrazine hydrate (22 µL, 0.4 mmol, 2 equiv.) in DMF (1 mL) was added dropwise at 0 °C, and then the resulting mixture was gently stirred for 1 h at room temperature. Methanol (2 mL) was added and stirred for 15 min to quench the excess of reactive chloride sites on the resin. The resin was filtered by vacuum, washed with DMF (2 x 5 mL), water (2 x 5 mL), DMF (2 x 5 mL), methanol (2 x 5 mL) and DMF (5 x 5 mL).[2] Then, the *C*-terminal Fmoc amino acid was loaded manually, a mixture of Fmoc amino acid (0.4 mmol, 2 equiv.), K-Oxyma (72 mg, 0.4 mmol, 2 equiv.) and *N*,*N'*-Diisopropylcarbodiimide (DIC) (63 µL, 0.4 mmol, 2 equiv.) in DMF (1 mL) was added to the resin then swelling the mixture with nitrogen flow for 45 min. The resin was filtered and double coupled using the same procedure. After that, the resin was filtered and washed with DMF (5 x 2 mL) (Batch-Flow-Flow-

Batch with DMF, batch washes for 1 min, flow washes for 30 sec). Later, the Fmoc loading test was carried out (Supplementary Fig. 2A).[3,4]

2.2.2 General manual peptide coupling method and Fmoc deprotection (50 mg resin scale)

Typically, to a solution of the Fmoc-amino acid (4 equiv.) and hexafluorophosphate azabenzotriazole tetramethyl uronium (HATU) (3.9 equiv.) in DMF, *N*,*N*-diisopropylethylamine (DIPEA) (8 equiv.) was added and mixed by shaking for 2 min. The resulting pre-activated amino acid solution was added to the resin mixture followed by swelling under nitrogen for 45 min at room temperature. A small portion of resin (~ 2 mg) was taken into a test tube to check the reaction completion by Kaiser Test[5] or Chloranil Test[6] for secondary amine. Recoupling was carried out for incomplete coupling result. For Fmoc deprotection, the resin was swelled in 20% piperidine in DMF (volume used is 3 times resin volume), swelling twice at room temperature (the first deprotection for 3 min followed by an additional deprotection for 12 min). The resin was filtered and washed with DMF (5 x 2 mL) (Batch-Flow-Flow-Batch with DMF, one batch wash for 1 min, one flow wash for 30 sec).

2.2.3 General automatic peptide coupling method and Fmoc deprotection (200~300 mg resin scale)

Automatic peptide synthesis was carried out on a Biotage Initiator+ Alstra, typically, each Fmoc-amino acid (4 equiv.) or 4-methyloctanoic acid (4 equiv.) was coupled at room temperature for 1 h with DIC (4 equiv.) and Oxyma (4 equiv.). Fmoc deprotection was accomplished with 20% piperidine in DMF twice (one deprotection for 1 min followed by an additional deprotection for 15 min).

2.2.4 General peptide cleavage from resin

Peptidyl resins (300 mg) were treated with 6 mL of cleavage cocktail. Cocktail A (1% TFA, 2% TIS and 97% DCM) for peptides containing *N*-prenylated tryptophan and Cocktail B (95% TFA, 2.5% TIS and 2.5% water) for other peptides. The resulting solution was shaken at room temperature for 1 h (Supplementary Fig. 2B). The resin was filtered and washed with DCM (2 x 6 mL) and the filtrates were evaporated under a nitrogen stream. Cold diethyl ether (3 x 10 mL) was added to precipitate hydrazide peptide as a yellowish powder (colour from the presence of 3-nitro-*L*-tyrosine). The resulting peptides were directly used for further synthesis without any purification.

Except for hydrazide peptide **9a**,**14a**,**15a** and **16a**. The purification of a small portion was carried out on semi-prep C8 column use HPLC using different gradient.

Gradient 10 for hydrazide peptide **9a**

Gradient 11 for hydrazide peptide **14a** and **15a**

Gradient 12 for hydrazide peptide **16a**

2.2.5 Synthesis of SNAC peptide 9b

All the buffer solutions were freshly prepared and degassed before using. It should be noted 5 M sodium nitrite stock was prepared as using degassed water to dissolve sodium nitrite. To a suspension of crude hydrazide linear peptide **9a** (1 equiv., 2 mM) in phosphate buffer pH 3 (6 M GdmCl, 50 mM NaH_2PO_4, 1.5 mM EDTA) was added sodium nitrite stock (20 equiv.) at -15 °C. Then the solution was left to stir for 20 min before *N*-acetylcysteamine (50 equiv.) was added. The resulting mixture was stirred for 15 min at 0 °C ~ r.t., then neutralized with saturated sodium bicarbonate and extracted with ethyl acetate (3 x 10 mL). Additional water was added to facilitate better separation. Combined organic layers were concentrated *in vacuo* at room temperature to give the product in quantitative conversion (HPLC). The product redissolved in 80% acetonitrile in H_2O, then purified through HPLC using gradient. Gradient 16 to give SNAC peptide **9b** (30% isolated yield).

2.3 Reverse-Phase Chromatography Methods

Analytical high-performance liquid chromatography (HPLC) was performed on an Agilent 1260 Infinity II instrument equipped with a DAD detector with an Agilent Eclipse XDB-C18 5 μm 4.6 x 150 mm column or Eclipse XDB-C8 5 μm 4.6 x 150 mm column at a constant flow rate of 1 mL/min at 40 °C. The UV-vis absorbance of the eluent was monitored at 220 nm, 280 nm and 355 nm. Typical gradients using mixture of two solvents are described below.

Solvent A: H_2O containing 0.1% TFA

Solvent B: MeCN containing 0.1% TFA

Solvent C: 20% MeCN in H_2O containing 0.1% TFA

Solvent D: 70% MeCN in H_2O containing 0.1% TFA

Solvent E: 30% MeCN in H_2O containing 0.1% TFA

Solvent F: 80% MeCN in H_2O containing 0.1% TFA

Solvent G: H_2O containing 0.1% FA

Solvent H: MeCN containing 0.1% FA

2.3.1 Analytical HPLC

Gradient 1: linear gradient from 5-100% B over 40 min, wash 5 min with 100% B, then back to 5% B over 3 min. (C18 4.6 x 150 mm)
Gradient 2: 2 min with 5% B, linear gradient from 5-100% B over 40 min, wash 5 min with 100% B, then back to 5% over 2 min. (C8 4.6 x 150 mm)
Gradient 3: 2 min with 5% B, linear gradient from 5-40% B over 1 min, linear gradient from 40-95% B over 9 min, wash 1 min with 95% B, then back to 5% B over 0.5 min, calibrate with 5% B over 1.5 min. (C8 4.6 x 150 mm)
Gradient 4: 2 min with 5% B, linear gradient from 5-50% B over 13 min, linear gradient from 50-95% B over 1 min, wash 2 min with 95% B, then back to 5% B over 1 min, calibrate with 5% B over 1 min. (C8 4.6 x 150 mm)
Gradient 5: linear gradient from 0-20% B over 15 min, linear gradient from 20-95% B over 1 min, wash 1 min with 95% B, then back to 0% B over 0.5 min, calibrate with 0% B over 2.5 min. (C8 4.6 x 150 mm)
Gradient 6: 1 min with 5% B, linear gradient from 5-90% B over 29 min, wash 2 min with 90% B, back to 5% B over 3 min. (C8 4.6 x 150 mm)
Gradient 7: linear gradient 5-95% B over 15 min, wash 2 min with 95% B, back to 5% B over 1 min, calibrate with 5% B over 2 min. (C8 4.6 x 150mm)
Gradient 8: linear gradient 5-95% B over 20 min, wash 1 min with 95% B, back to 5% B over 1 min, calibrate with 5% B over 1 min. (C8 4.6 x 150mm)
Gradient 9: 2 min with 5% B, linear gradient from 5-50% B over 28 min, linear gradient from 50-95% B over 1 min, wash 2 min with 95% B, then back to 5% B over 1 min, calibrate with 5% B over 1 min. (C8 4.6 x 150 mm)
Gradient 24: linear gradient from 5-95% B over 15 min. (C18 4.6 x 150 mm)

2.3.2 Semi-preparative HPLC

In semi-preparative mode, an Agilent Eclipse XDB-C8 5 μm 9.4 x 250 mm column or Agilent ZORBAX 300SB-C18 5 μm 9.4 x 250 mm column or Agilent ZORBAX 300-C3 5 μm 9.4 x 250 mm column was used at a constant flow rate of 3.5 mL/min at 60 °C.
Gradient 10: linear gradient 25%~100% D in C over 37 min, then wash 2 min with 100% D, then back to 25% D in C over 2 min. (C8 9.4 x 250 mm)

Gradient 11: 2 min with 35% B, linear gradient 35%~65% B over 25 min, then 65%~95% B over 1 min, wash 5 min with 95% B, then back to 35% B over 2 min. (C8 9.4 x 250 mm)

Gradient 12: 2 min with 30% B, linear gradient from 30%~50% B over 35 min, then 50%~95% B over 1 min, wash 5 min with 95% B, then back to 30% B over 2 min. (C8 9.4 x 250 mm)

Gradient 13: 2 min with 20% B, linear gradient from 20%~95% B over 40 min, then wash 2 min with 95% B, then back to 20% B over 2 min. (C18 9.4 x 250 mm)

Gradient 19: 2 min with 5% B, linear gradient from 5~45% B over 1 min, then linear gradient from 45%~85% B over 25 min, then linear gradient from 85~95% over 0.5 min, then wash 2 min with 95% B, then back to 5% over 2 min. (C3 9.4 x 250 mm)

Gradient 22: 1 min with 5% B, linear gradient from 5%~95% B over 26 min, then wash 2 min with 95% B, then back to 5% B over 2 min. 3.5 mL /min. (C8 9.4 x 250 mm)

Gradient 23: 3 min with 2% B, linear gradient from 2~15% B over 1 min, then linear gradient from 15%~40% B over 40 min, then linear gradient from 40~95% over 1 min, then wash 2 min with 95% B, then back to 2% over 2 min. (C8 9.4 x 250 mm)

2.3.3 Preparative HPLC

HPLC purification of the peptides was performed on an Agilent 1260 Preparative HPLC system using a reversed phase Agilent Zorbax 300SB-C18 PrepHT 21.2 x 150 mm 7 μm column was used at a constant flow rate of 20 mL/min at room temperature.

Gradient 14: linear gradient from 0%~100% F in E over 40 min, then wash 5 min with 100% F, then back to 100% E over 2 min.

Gradient 15: linear gradient from 20%~70% B over 30 min, then 70%~95% B over 1 min, wash 2 min with 95% B, then back to 20% B over 2 min.

Gradient 16: linear gradient from 30%~70% B over 40 min, then 70%~95% B over 1 min, wash 2 min with 95% B, then back to 30% B over 2 min.

Gradient 17: 2 min with 10% B, linear gradient from 10~50% B over 17 min, then linear gradient from 50~95% B over 1 min, wash 2 min with 95% B, then back to 10% B over 2 min.

Gradient 20: 2 min with 2% B, linear gradient from 2~30% B over 30 min, then linear gradient from 30~95% B over 0.5 min, wash 2 min with 95% B, then back to 2% B over 2 min.

Gradient 21: 2 min with 2% B, linear gradient from 2~15% B over 2 min, then linear gradient from 15~40% B over 30 min, then linear gradient from 40~95% B over 0.5 min, wash 2 min with 95% B, then back to 2% B over 2 min.

2.3.4 UPLC-HRMS

All hydrazide peptides were analyzed on a Waters ACQUITY UPLC BEH C8, 1.7 µm, 2.1 x 50 mm column, the rest of peptides on a Waters ACQUITY UPLC BEH C18, 1.7 µm, 2.1 x 50 mm column, the proteins were analyzed on a Waters ACQUITY UPLC BEH C4, 300 A, 1.7 µm, 2.1 x 50 mm column with constant flow of 0.4 mL/min 40 °C. High-resolution mass spectra were recorded on a Waters Acquity UPLC-Class I equipped with an ACQUITY UPLC column. The detector was a Waters Xevo-G2-XS QTof with electrospray ionization source. The instrument was operated in positive mode full scan with detection window set from 50 to 2000 Da. For peptide fragmentation, a collision energy ramp from 15 V to 40 V was employed. Typical gradients are described below.

Gradient 18: 0.5 min with 5% solvent H in solvent G, linear gradient from 5~95% solvent H over 4 min, wash 1 min with 95% solvent H, then back to 5% over 0.5 min and then 0.5 min with 5% solvent H.

2.4 Chemical Peptide Cyclization

2.4.1 Silver assisted peptide cyclization

The procedure used was developed by Tam and co-workers.[7] SNAC peptide **9b** (final conc. 0.2 mM) was dissolved in 1 mL of a 1:1 mixture of DMSO and sodium acetate buffer (0.5 M, pH 7.5). A large volume of DMSO here is essential for SNAC peptide solubility. Silver trifluoroacetate (3 equiv.) was added, then the resulting mixture was shaken at room temperature for 1h. The crude mixture was directly analyzed by HPLC using Gradient 1.

2.4.2 General procedure for bioinspired peptide cyclization (analytical scale)

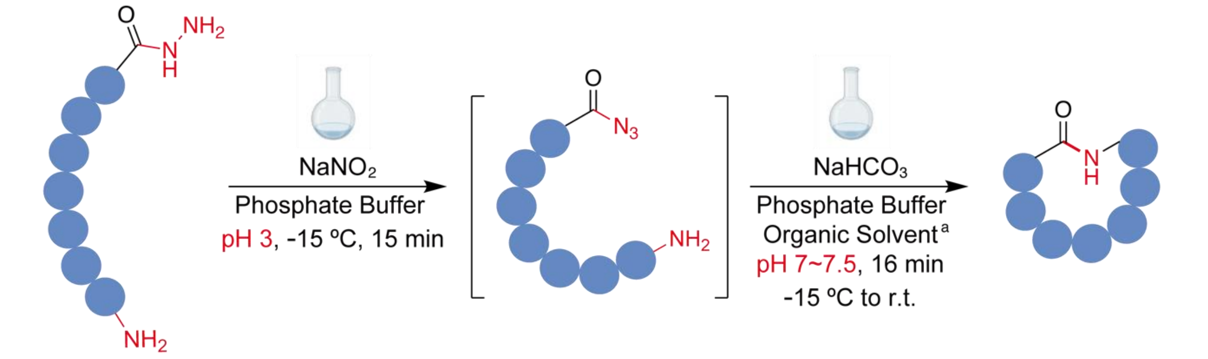

Figure S3. Chemical cyclization of linear peptide. The reaction includes two steps and is carried out in one-pot style. [a] for analytical scale reaction organic solvent is either 50% acetonitrile in water +0.1% TFA or ethyl acetate (peptide sequence dependent).

All buffers and solutions were freshly prepared and degassed before using. It should be noted 5 M sodium nitrite stock was prepared using degassed water to dissolve sodium nitrite. Typically, for analytical scale, to a solution of purified hydrazide linear peptide **9a** (2.2 µmol, 1 equiv.) in 1 mL of pH 3 phosphate buffer (6 M GdmCl, 50 mM NaH_2PO_4, 1.5 mM EDTA) was added sodium nitrite stock (44 µmol, 20 equiv.) at -15 °C under nitrogen. Then the solution was stirred for 15 min at -15 °C. The mixture was gently neutralized with saturated sodium bicarbonate to pH 6.7-7.0 (monitored using pH indicator paper). 500 µL of the solution was pipetted out and diluted with 1 mL of 50% solvent B in A in a HPLC sample vial (sample vial was sitting in the HPLC container). Then the reaction was analyzed by HPLC using Gradient 2 or Gradient 3. For the peptides, cyclosporine analogue **31a** and cyclomarin analogue **32a**, after neutralizing to pH 6.7-7.0 the ice-salt bath was removed and immediately 1 mL of ethyl acetate was added to the reaction and the resulting biphasic mixture was stirred

vigorously. After 20 min stirring, the organic phase was separated, and the aqueous phase was extracted three times with ethyl acetate (few drops water required to get better separation). The combined organic phases were concentrated *in vacuo*, then the crude mixture was analysed by HPLC. (Note: concentration of peptide hydrazides containing nitro tyrosine were estimated using Fmoc-3-nitro-*L*-tyrosine calibration curve (Fig. S12). For peptides without 3-nitro-*L*-tyrosine, the concentration was estimated based on the absorbance at 214 nm and predicted extinction coefficients. All peptides were used directly in their crude form after peptide cleavage unless otherwise stated.)

2.4.3 General Procedure for Bioinspired Peptide Cyclization (semi-prep scale)

Isolated yields follow mass loss after purification on semi-preparative HPLC to ensure isolation of peptides of >95% purity to allow accurate full characterisation of novel compounds. All reactions were performed at this scale once without further optimisation of the reaction conditions developed for peptide **9**. All buffers and solutions were freshly prepared and degassed before use.

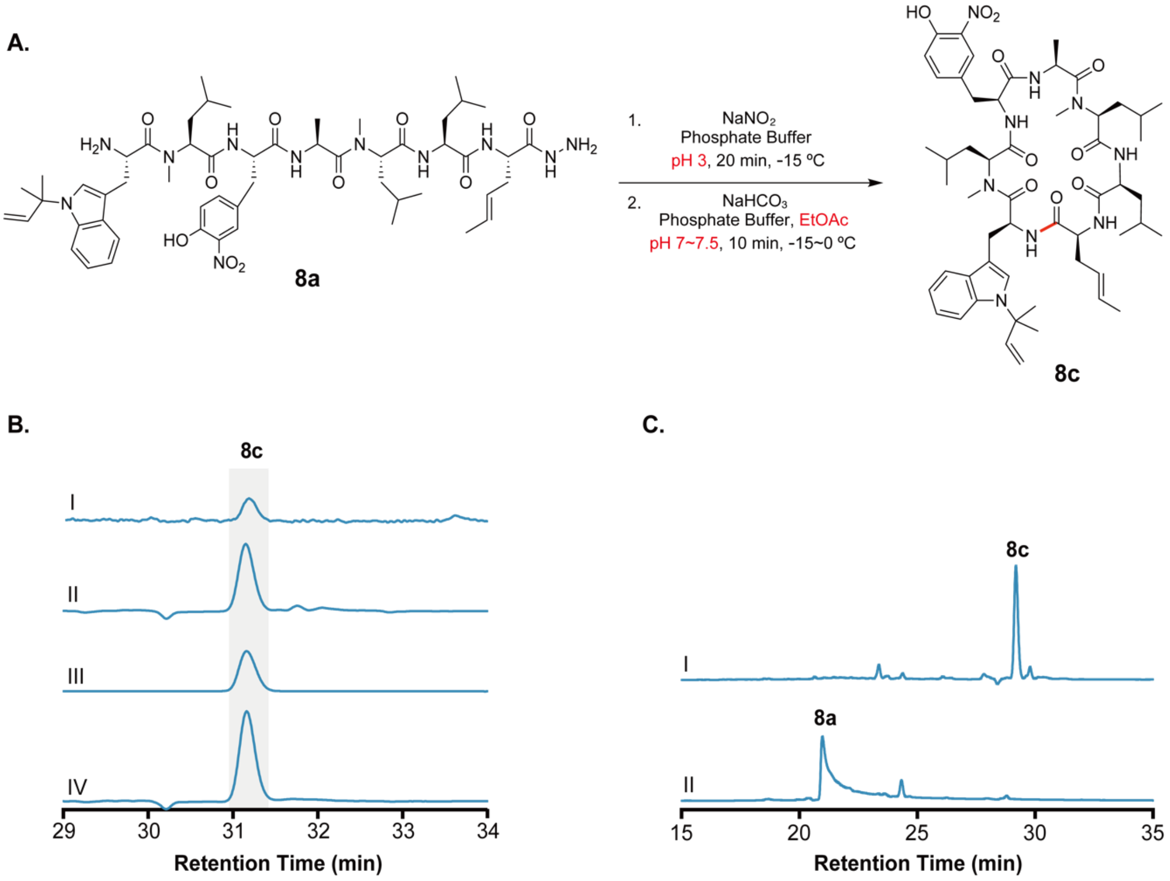

Figure S4. Chemical cyclization of natural product rufomycin B 8c. A. One-pot style, fast macrocyclization of rufomycin B including oxidation of *C*-terminal acyl hydrazide peptide to acyl azide

followed by *in-situ* head to tail cyclization. **B.** HPLC comparison of rufomycin B **8c** purified from *S. atratus* extract (I & **II,** prepared as previously reported)[23] and chemical cyclization (**III**). (**IV**) co-injection of above three. **C.** HPLC analysis of chemical cyclization of **8a.** (**II**) acyl hydrazide peptide crude starting material, (**I**) reaction mixture after extraction. All UV-vis traces were recorded at 355 nm.

To a suspension of crude hydrazide linear peptide **8a** (0.03 mmol, 1 equiv., 3 mM) in 10 mL of phosphate buffer (6 M GdmCl, 50 mM NaH_2PO_4, 1.5 mM EDTA, pH 3) was added sodium nitrite (0.60 mmol, 20 equiv.) at -15 °C under nitrogen. The resulting solution was stirred for 20 min at -15 °C before gently raising up the pH to 7.0~7.5 with saturated sodium bicarbonate. The ice-salt bath was removed and immediately 15 mL (1.5 volume) of ethyl acetate was added and the biphasic mixture was stirred vigorously for 15 min. Then the organic phase was separated, and the aqueous phase was further extracted with ethyl acetate (3 x 15 mL). The combined organic layers were concentrated *in vacuo* at room temperature and redissolved in 80% acetonitrile in water, then purified through preparative HPLC using Gradient 14. After lyophilization, 11 mg of rufomycin B **8a** was harvested as a yellowish powder. (HPLC yield: 84%, isolated yield: 39%).

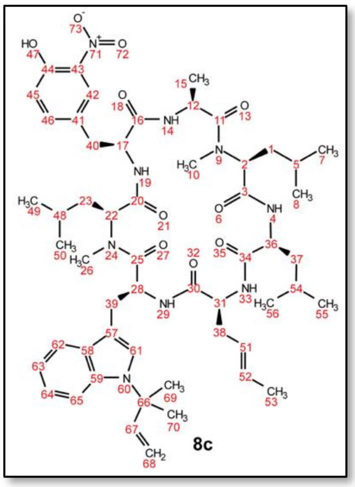

^{1}H NMR (CD_3OD, 700 MHz) δ 7.79 (d, *J* = 2.2 Hz, 1H, CH-C42), 7.54 – 7.50 (m, 2H, CH-C62 & CH-C65), 7.40 (dd, *J* = 8.7, 2.3 Hz, 1H, CH-C46), 7.13 (s, 1H, CH-C61), 7.09 – 7.00 (m, 3H, CH-C45, CH-C63 & CH-C64), 6.16 – 6.10 (m, 1H, CH-C67), 5.52 – 5.44 (m, 1H, CH-C52), 5.23 – 5.13 (m, 3H, CH-C51 & CH_2-C68), 4.94 – 4.91 (m, 1H, CH-C28), 4.89 – 4.87 (m, 1H, CH-C2, overlap with water), 4.89 – 4.84 (m, 1H, CH-C12, overlap with water), 4.72 – 4.69 (m, 1H, CH-C22), 4.63 – 4.50 (m, 3H, CH-C31, CH-C17, CH-C36), 3.42 (dd, *J* = 13.3, 11.2 Hz, 1H, CH-C39), 3.16 (dd, *J* = 13.3, 4.4 Hz, 1H, CH-C39), 3.05 (dd, *J* = 13.3, 9.6 Hz, 1H, CH-C40), 2.75 – 2.72 (m, 5H, CH-

C38, CH-C40, CH_3-C10), 2.67 (s, 3H, CH_3-C26), 2.47 – 2.40 (m, 1H, CH-C38), 1.98 – 1.95 (ddd, *J* = 13.7, 7.5, 5.9 Hz, 1H, CH-C1), 1.81 – 1.69 (m, 9H, CH_2-C37, CH-C54, CH_3-C69 & CH_3-C70), 1.64 – 1.61 (m, 1H, CH-C23), 1.58 – 1.55 (m, 3H, CH_3-C53), 1.54 – 1.51 (m, 1H, CH-C1), 1.51 – 1.45 (m, 1H, CH-C5), 1.23 (d, *J* = 6.8 Hz, 3H, CH_3-C15), 1.10 – 1.04 (m, 1H, CH-C48), 1.00 – 0.91 (m, 12H, CH_3-C55, CH_3-C56, CH_3-C7 & CH_3-C8), 0.38 (d, *J* = 6.8 Hz, 3H, CH_3-C50 or CH_3-C49), 0.18 (d, *J* = 6.7 Hz, 3H, CH_3-C49 or CH_3-C50), -0.60 – -0.66 (m, 1H, CH-C23).

^{13}C NMR (CD_3OD, 176 MHz) δ 175.08 (C11), 174.00 (C34), 173.95 (C25), 173.48 (C30), 172.31(C16), 171.55 (C3), 169.80 (C20), 154.65 (C44), 145.41 (C67), 139.27 (C46), 136.94 (C59), 135.53 (C43), 131.34 (C52), 130.42 (C58), 129.53 (C41), 126.80 (C42), 125.47 (C61), 125.30 (C51), 122.15 (C64), 121.14 (C45), 120.36 (C63), 119.46 (C62), 115.32 (C65), 114.12 (C68), 108.94 (C57), 60.21 (C66), 59.99 (C2), 59.79 (C22), 55.55 (C17), 55.00 (C36), 53.45 (C31), 51.90 (C28), 46.76 (C12), 44.38 (C37), 38.72 (C1), 38.49 (C23), 38.20 (C40), 36.22 (C38), 29.88 (C10), 29.82 (C26), 28.53 (C69 or C70), 28.42 (C69 or C70), 28.28 (C39), 25.98 (C54), 25.79 (C5), 25.47 (C48), 23.49 (C50 or C49), 23.39 (C7 or C8), 23.25 (C55 or C56), 23.06 (C7 or C8), 21.96 (C55 or C56), 21.02 (C49 or C50), 18.64 (C53), 17.01 (C15).
As for natural rufomycins this cyclic peptide appears conformers only the major species is assigned.
HRMS (ESI) calc. for $C_{54}H_{77}N_9O_{10}$ calc. for $[M+H]^+$: 1012.5872 m/z, found: 1012.5898 m/z. (*Δ* = 2.56 ppm)
Purification: Purified through preparative HPLC using Gradient 14.

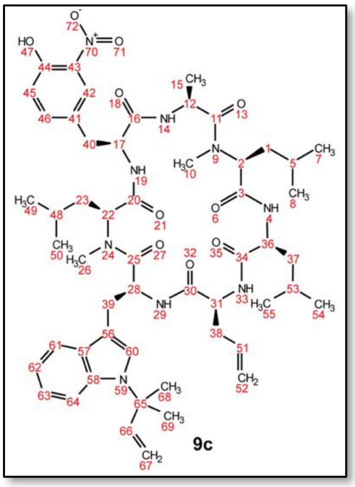

Method: Peptide **9a** was synthesised using the same cyclisation procedure as used for linear peptide **8a**. The linear peptide **9a** was present in solution at 3 mM concentration (10 mL reaction buffer). **9c** was isolated after HPLC purification as a yellowish powder. (HPLC conversion (90%), isolated yield (56%))

^{1}H NMR (CD_3OD, 700 MHz) δ 7.79 (d, *J* = 2.2 Hz, 1H, CH-C_{42}), 7.54 – 7.50 (m, 2H, CH-C61 & CH-C64), 7.42 (dd, *J* = 8.6, 2.3 Hz, 1H, CH-C46), 7.12 (s, 1H, CH-C60), 7.08 – 7.01 (m, 3H, CH-C45, CH-C62 & CH-C63), 6.16 – 6.11 (m, 1H, CH-C66), 5.65 – 5.57 (m, 1H, CH-C51), 5.22 – 5.14 (m, 2H, CH_2-C67), 5.13 – 5.06 (m, 2H, CH_2-C52), 4.93 (ddd, *J* = 11.2, 4.5, 2.1 Hz, 1H, CH-C28), 4.89 (dd, *J* = 8.2, 5.9 Hz, 1H, CH-C2), 4.86 (m, 1H, CH-C12, overlap with water), 4.72 – 4.65 (m, 2H, CH-C22 & CH-C31), 4.65 – 4.56 (m, 2H, CH-C17 & CH-C36), 3.43 (dd, *J* = 13.3, 11.1 Hz, 1H, CH-C39), 3.16 (dd, *J* = 13.3, 4.3 Hz, 1H, CH-C39), 3.07 (dd, *J* = 13.4, 9.2 Hz, 1H, CH-C40), 2.83 – 2.77 (m, 1H, CH-C38), 2.76 – 2.71 (m, 4H, CH-C40 & CH_3-C10), 2.60 (s, 3H, CH_3-C26), 2.56 – 2.49 (m, 1H, CH-C38), 1.96 (ddd, *J* = 13.6, 7.4, 5.7 Hz, 1H, CH-C1), 1.79 – 1.68 (m, 9H, CH_2-C37, CH-C53, CH_3-C68 & CH_3-C69), 1.64 – 1.58 (m, 1H, CH-C23), 1.57 – 1.52 (m, 1H, CH-C1), 1.52 – 1.45 (m, 1H, CH-C5), 1.24 (d, *J* = 6.9 Hz, 3H, CH_3-C15), 1.10 – 1.05 (m, 1H, CH-C48), 1.00 – 0.93 (m, 12H, CH_3-C54, CH_3-C55, CH_3-C7 & CH_3-C8), 0.38 (d, *J* = 6.7 Hz, 3H, CH_3-C50 or CH_3-C49), 0.18 (d, *J* = 6.6 Hz, 3H, CH_3-C49 or CH_3-C50), -0.59 – -0.67 (m, 1H, CH-C23).

^{13}C NMR (CD_3OD, 176 MHz) δ 175.14 (C11), 174.05 (C34), 173.79 (C25), 173.36 (C30), 172.47 (C16), 171.56 (C3), 169.69 (C20), 154.63 (C44), 145.44 (C66), 139.26 (C46), 136.97 (C58), 135.52 (C43), 133.15 (C51), 130.43 (C57), 129.76 (C41), 126.78 (C42), 125.48 (C60), 122.15 (C63), 121.10 (C45), 120.44 (C52), 120.35 (C62), 119.46 (C61), 115.32 (C64), 114.10 (C67), 109.00 (C56), 60.21 (C65), 60.03 (C2), 59.81 (C22), 55.60 (C17), 54.89 (C36), 53.26 (C31), 51.77 (C28), 46.80 (C12), 44.37 (C37), 38.70 (C1), 38.63 (C23), 37.90 (C40), 37.52 (C38), 29.89 (C10), 29.73 (C26), 28.52 (C68 or C69), 28.42 (C68 or C69), 28.21 (C39), 26.03 (C53), 25.83 (C5), 25.49 (C48), 23.49 (C50 or C49), 23.41 (C7 or C8), 23.25 (C54 or C55), 23.05 (C7 or C8), 21.95 (C54 or C55), 21.01 (C49 or C50), 17.05 (C15).

As for natural rufomycins this cyclic peptide appears as conformers, only the major species is assigned.

HRMS (ESI) calc. for $C_{53}H_{75}N_9O_{10}$ calc. for $[M+H]^+$: 998.5715 m/z, found: 998.5717 m/z. (Δ = 0.20 ppm)

Purification: Purified through preparative HPLC using Gradient 14.

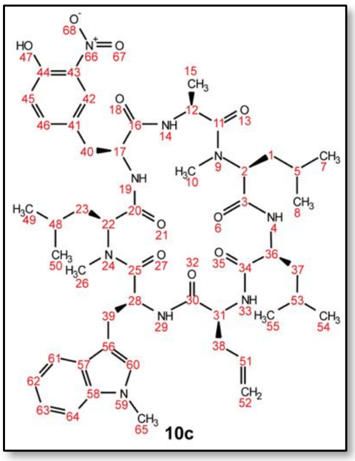

10c

Method: Peptide **10a** was synthesised using the same cyclisation procedure as used for linear peptide **8a**. The linear peptide **10a** was present in solution at 3 mM concentration (10 mL reaction buffer). **10c** was isolated after HPLC purification as a yellowish powder. (HPLC conversion (93%), isolated yield (58%)

^{1}H NMR (CD_3OD, 600 MHz) δ 7.79 (d, *J* = 2.3 Hz, 1H, CH-C42), 7.51 (d, *J* = 7.9 Hz, 1H, CH-C61), 7.42 (dd, *J* = 8.5, 2.2 Hz, 1H, CH-C46), 7.35 (d, *J* = 8.2 Hz, 1H, CH-C64), 7.17 (t, *J* = 7.7 Hz, 1H, CH-C62), 7.09 – 7.01 (m, 2H, CH-C63 & CH-C45), 6.95 (s, 1H, CH-C60), 5.64 – 5.57 (m, 1H, CH-C51), 5.15 – 5.06 (m, 2H, CH_2-C52), 4.96 – 4.92 (m, 1H, CH-C28), 4.89 – 4.86 (m, 1H, CH-C2, overlap with water), 4.83 – 4.78 (m, 1H, CH-C12), 4.68 – 4.64 (m, 1H, CH-C31), 4.64 – 4.55 (m, 3H, CH-C17, CH-C22 & CH-C36), 3.74 (s, 3H, CH_3-C65), 3.45 (dd, *J* = 13.5, 10.8 Hz, 1H, CH-C39), 3.14 (dd, *J* = 13.4, 4.1 Hz, 1H, CH-C39), 3.07 (dd, *J* = 13.2, 9.4 Hz, 1H, CH-C40), 2.86 – 2.77 (m, 1H, CH-C38), 2.77 – 2.70 (m, 4H, CH-C40 & CH_3-C10), 2.56 – 2.54 (m, 4H, CH-C38 & CH_3-C26), 1.98 – 1.92 (m, 1H, CH-C1), 1.82 – 1.67 (m, 3H, CH_2-C37 & CH-C53), 1.60 – 1.51 (m, 2H, CH-C23, CH-C1), 1.50 – 1.43 (m, 1H, CH-C5), 1.22 (d, *J* = 6.8 Hz, 3H, CH_3-C15), 1.06 – 0.99 (m, 1H, C48), 0.99 – 0.91 (m, 12H, CH_3-C54, CH_3-C55, CH_3-C7 & CH_3-C8) 0.44 (d, *J* = 6.6 Hz, 3H, CH_3-C50 or CH_3-C49), 0.21 (d, *J* = 6.5 Hz, 3H, CH_3-C49 or CH_3-C50), -0.56 (ddd, *J* = 13.1, 9.4, 3.5 Hz, 1H, CH-C23).

^{13}C NMR (CD_3OD, 151 MHz) δ 175.14 (C11), 174.18 (C34), 173.83 (C25), 173.22 (C30), 172.43 (C16), 171.61 (C3), 169.83 (C20), 154.61 (C44), 139.23 (C46), 138.52

(C58), 135.51(C43), 133.14 (C51), 129.83 (C41), 129.06 (C60), 129.05 (C57), 126.74 (C42), 122.96 (C63), 121.08 (C45), 120.43 (C52), 120.30 (C62), 119.61 (C61), 110.67 (C64), 109.87 (C56), 60.13 (C2), 59.92 (C22), 55.61 (C17), 55.01 (C36), 53.31 (C31), 52.14 (C28), 46.78 (C12), 44.56 (C37), 38.63 (C1), 38.25 (C23), 37.86 (C40), 37.46 (C38), 32.81 (C65), 29.89 (C10), 29.80 (C26), 28.37 (C39), 26.02 (C53), 25.82 (C5), 25.42 (C48), 23.40 (C50 or C49), 23.43; 23.22; 23.03; 21.96 (C7, C8, C54 & C55), 21.19 (C49 or C50), 17.04 (C15).

As for natural rufomycins this cyclic peptide appears as conformers only the major species is assigned.

HRMS (ESI) calc. for $C_{49}H_{69}N_9O_{10}$ calc. for $[M+H]^+$: 944.5245 m/z, found: 944.5281 m/z. (Δ = 3.81 ppm)

Purification: Purified through preparative HPLC using Gradient 15.

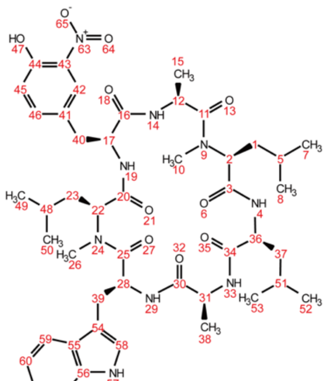

11c

Method: Peptide **11a** was synthesised using the same cyclisation procedure as used for linear peptide **8a**. The linear peptide **9a** was present in solution at 3.5 mM concentration (10 mL reaction buffer). **11c** was isolated after HPLC purification as a yellowish powder (HPLC conversion (87%), isolated yield (52%))

^{1}H NMR (CD_3OD, 700 MHz) δ 7.78 (d, *J* = 2.2 Hz, 1H, CH-C42), 7.50 (d, *J* = 8.19 Hz, 1H, CH-C59), 7.41 (dd, *J* = 8.6, 2.2 Hz, 1H, CH-C46), 7.35 (d, *J* = 8.19 Hz, 1H, CH-C62), 7.12 – 7.08 (m, 1H, CH-C61), 7.07 – 7.04 (m, 1H, CH-C45), 7.03 – 6.98 (m, 2H, CH-C60 & CH-C58), 4.91 – 4.87 (m, 2H, CH-C28 & CH-C2, overlap with water), 4.83 – 4.79 (m, 1H, CH-C12, overlap with water), 4.65 – 4.60 (m, 1H, CH-C17), 4.57 – 4.53 (m, 3H, CH-C31, CH-C22 & CH-C36), 3.41 (dd, *J* = 13.5, 10.9 Hz, 1H, CH-C39), 3.18 – 3.14 (m, 1H, CH-C39), 3.03 (dd, *J* = 13.5, 8.6 Hz, 1H, CH-C40), 2.80 – 2.75 (m, 1H, CH-C40), 2.74 (s, 3H, CH_3-C10), 2.47 (s, 3H, CH_3-C26), 1.95 (ddd, *J* = 13.7, 7.6, 5.7

Hz, 1H, CH-C1), 1.81 – 1.66 (m, 3H, CH_2-C37 & CH-C51), 1.57 – 1.44 (m, 3H, CH-C1, CH-C23, CH-C5), 1.41 (d, J = 6.9 Hz, 3H, CH_3-C38), 1.25 (d, J = 6.9 Hz, 3H, CH_3-C15), 1.03 – 0.99 (dd, J = 9.3, 6.4 Hz, 1H, CH-C48), 0.99 – 0.91 (m, 12H, CH_3-C53, CH_3-C52, CH_3-C7 & CH_3-C8), 0.43 (d, J = 6.6 Hz, 3H, CH_3-C50 or CH_3-C49), 0.27 (d, J = 6.6 Hz, 3H, CH_3-C49 or CH_3-C50), -0.51 (ddd, J = 13.1, 9.3, 3.6 Hz, 1H, CH-C23).

^{13}C NMR (CD_3OD, 176 MHz) δ 175.31 (C11), 174.66 (C30), 174.19 (C25), 173.95 (C34), 172.33 (C16), 171.69 (C3), 170.00 (C20), 154.43 (C44), 139.33 (C46), 137.91 (C56), 135.52 (C43), 129.72 (C41), 128.50 (C55), 126.85 (C42), 124.67 (C58), 122.76 (C61), 120.99 (C45), 120.18 (C60), 119.20 (C59), 112.68(C62), 110.20 (C54), 60.07 (C2), 59.91 (C22), 54.99 (C17), 54.55 (C36), 52.33 (C28), 49.72 (C31), 46.81 (C12), 43.39 (C37), 38.68 (C1), 37.95 (C40), 37.82 (C23), 29.98 (C10), 29.76 (C26), 28.71 (C39), 26.00 (C51), 25.78 (C5), 25.48 (C48), 23.37 (C52 or C53), 23.27 (C50 or C49), 23.26 (C7 or C8), 23.01 (C7 or C8), 21.97 (C52 or C53), 21.28 (C49 or C50), 19.53 (C38), 17.03 (C15).

As for natural rufomycins this cyclic peptide appears as conformers only the major species is assigned.

HRMS (ESI) calc. for $C_{46}H_{65}N_9O_{10}$ calc. for $[M+H]^+$: 904.4933 m/z, found: 904.4977 m/z. (Δ = 4.86 ppm)

Purification: Purified through preparative HPLC using Gradient 15.

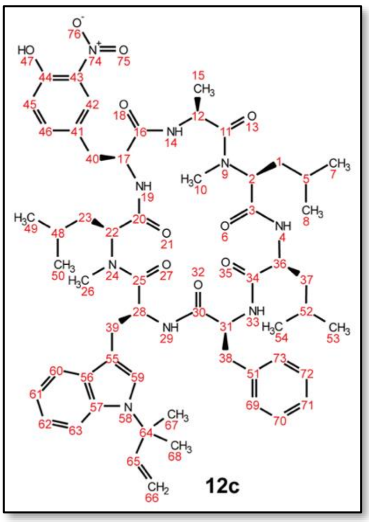

12c

Method: Peptide **12a** was synthesised using the same cyclisation procedure as used for linear peptide **8a**. The linear peptide **12a** was present in solution at 2 mM

concentration (10 mL reaction buffer). **12c** was isolated after HPLC purification as a yellowish powder. (HPLC conversion (63%), isolated yield (22%)

^{1}H NMR (CD_3OD, 700 MHz) δ 8.76 (d, *J* = 8.6 Hz, 1H, NH-N19), 7.65 (d, *J* = 2.2 Hz, 1H, CH-C42), 7.56 – 7.51 (m, 2H, CH-C60 & C63), 7.29 – 7.26 (m, 1H, CH-C46), 7.19 – 7.15 (m, 3H, CH-C70, CH-C71 & CH-C72), 7.14 – 7.11 (m, 2H, C73 & C69), 7.09 – 7.03 (m, 3H, CH-C61, CH-C62 & CH-C59), 7.03 – 7.00 (dd, *J* = 8.6, 2.5 Hz, 1H, CH-C45), 6.18 – 6.11 (m, 1H, CH-C65), 5.24 – 5.15 (m, 2H, CH_2-C66), 4.97 – 4.93 (m, 1H, CH-C28), 4.90 – 4.86 (m, 1H, CH-C2), 4.84 – 4.81 (m, 1H, CH-C31, overlap with water), 4.71 (q, *J* = 6.9 Hz, 1H, CH-C12), 4.67 – 4.60 (m, 2H, CH-C36 & CH-C22), 4.44 – 4.38 (m, 1H, CH-C17), 3.52 (dd, *J* = 13.2, 11.2 Hz, 1H, CH-C39), 3.50 – 3.45 (m, 1H, CH-C38), 3.25 – 3.19 (m, 2H, CH-C39 & CH-C38), 2.75 (s, 3H, CH_3-C26), 2.68 (s, 3H, CH_3-C10), 2.29 – 2.24 (m, 1H, CH-C40), 2.17 – 2.11 (m, 1H, CH-C40), 1.97 – 1.91 (m, 1H, CH-C1), 1.76 – 1.70 (m, 6H, CH_3-C67 & CH_3-C68), 1.70 – 1.63 (m, 2H, CH-C37 & CH-C52), 1.63 – 1.56 (m, 2H, CH-C37 & CH-C23), 1.53 – 1.46 (m, 2H, CH-C1 & CH-C5), 1.17 (dd, *J* = 6.8, 4.1 Hz, 3H, CH_3-C15), 1.11 – 1.04 (m, 1H, CH-C48), 0.99 – 0.91 (m, 12H, CH_3-C53, CH_3-C54, CH_3-C7 & CH_3-C8), 0.37 (d, *J* = 6.8 Hz, 3H, CH_3-C50 or CH_3-C49), 0.13 (d, *J* = 6.7 Hz, 3H, CH_3-C49 or CH_3-C50), -0.63 – -0.67 (m, 1H, CH-C23).

^{13}C NMR (CD_3OD, 176 MHz) δ 175.04 (C11), 174.08 (C34), 173.44 (C25), 172.94 (C30), 172.37 (C16), 171.55 (C3), 169.52 (C20), 154.51 (C44), 145.46 (C65), 139.19 (C46), 136.99 (C51), 136.97 (C57), 135.45 (C43), 131.62 (C69 & C73), 130.47 (C56), 129.94 (C41), 129.33 (C70 & C72), 128.24 (C59), 126.63 (C42), 125.57 (C71), 122.19 (C62), 120.95 (C45), 120.40 (C61), 119.47 (C60), 115.35 (C63), 114.12 (C66), 108.91 (C55), 60.24 (C64), 60.04 (C2), 59.97 (C22), 55.31 (C17), 54.94 (C36), 54.61 (C31), 51.95 (C28), 46.64 (C12), 44.53 (C37), 38.68 (C1), 38.66 (C23), 37.92 (C38), 37.86 (C40), 29.91 (C26 or C10), 29.90 (C10 or C26), 28.55 (C67 or C68), 28.44 (C67 or C68), 28.31 (C39), 25.96 (C52), 25.77 (C5), 25.51 (C48), 23.46 (C50 or C49), 23.42 (C7 or C8 and C53 or C54), 23.03 (C7 or C8), 21.67 (C53 or C54), 20.94 (C49 or C50), 17.04 (C15).

As for natural rufomycins this cyclic peptide appears as conformers only the major species is assigned.

HRMS (ESI) calc. for $C_{57}H_{77}N_9O_{10}$ calc. for $[M+H]^+$: 1048.5872 m/z, found: 1048.5884 m/z. (Δ = 1.14 ppm)

Purification: Purified through semi-preparative HPLC using Gradient 13.

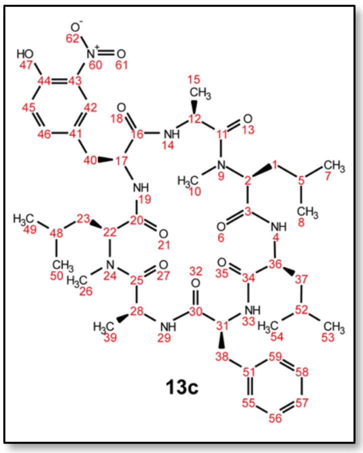

13c

Method: Peptide **13a** was synthesised using the same cyclisation procedure as used for linear peptide **8a**. The linear peptide **13a** was present in solution at 3 mM concentration (10 mL reaction buffer). **13c** was isolated after HPLC purification as a yellowish powder. (HPLC conversion (88 %), isolated yield (40%))

^{1}H NMR (CD_3OD, 700 MHz) δ 7.73 (d, J = 2.2 Hz, 1H, CH-C42), 7.33 (dd, J = 8.6, 2.2 Hz, 1H, CH-C46), 7.21 – 7.19 (m, 2H, CH-C56 & CH-C58), 7.16 – 7.13 (m, 2H, CH-C55 & CH-C59), 7.13 – 7.09 (m, 1H, CH-C57), 7.05 (dd, J = 8.5, 1.2 Hz, 1H, CH-C45), 4.91 – 4.86 (m, 2H, CH-C22, CH-C28), 4.82 – 4.81 (m, 1H, CH-C2), 4.69 – 4.59 (m, 4H, CH-C31, CH-C17, CH-C12 & CH-C36), 3.44 (dd, J = 14.1, 5.8 Hz, 1H, CH-C38), 3.18 – 3.12 (m, 1H, CH-C38), 2.70 (s, 3H, CH_3-C26), 2.68 (s, 3H, CH_3-C10), 2.50 – 2.44 (m, 1H, CH-C40), 2.40 (dd, J = 13.8, 6.2 Hz, 1H, CH-C40), 1.99 – 1.93 (m, 1H, CH-C1), 1.88 – 1.83 (m, 1H, CH-C23), 1.64 – 1.54 (m, 4H, CH-C23, CH_2-C37 & CH-C52), 1.55 – 1.44 (m, 3H, CH-C1, CH-C5 & CH-C48), 1.42 (d, J = 6.8 Hz, 3H, CH_3-C39), 1.24 (d, J = 6.9 Hz, 3H, CH_3-C15), 1.00 – 0.95 (m, 6H, CH_3-C49 & CH_3-C50), 0.95 – 0.93 (m, 6H, CH_3-C7 & CH_3-C8), 0.93 – 0.88 (m, 6H, CH_3-C53 & CH_3-C54).

^{13}C NMR (CD_3OD, 176 MHz) δ 175.17 (C11), 174.85 (C25), 174.01 (C34), 172.63 (C30), 172.35 (C16), 171.37 (C3), 170.70 (C20), 154.43 (C44), 139.26 (C46), 137.00 (C51), 135.48 (C43), 131.63 (C55 & C59), 130.25 (C41), 129.19 (C56 & C58), 128.09 (C57), 126.60 (C42), 120.92 (C45), 60.43 (C22), 60.10 (C2), 54.80 (C36), 54.75 (C17),

54.61 (C31), 46.63 (C12), 46.30 (C28), 43.93 (C37), 38.56 (C23 or C1), 38.55 (C1 or C23), 37.95 (C38), 37.50 (C40), 29.96 (C26), 29.92 (C10), 25.92 (C52), 25.76 (C5), 25.58 (C48), 23.58 (C49 or C50), 23.44 & 23.42 (C53 or C54 and C7 or C8), 22.99 (C7 or C8), 22.82 (C49 or C50), 21.65 (C53 or C54), 17.22 (C39), 17.11 (C15).

As for natural rufomycins this cyclic peptide appears as conformers only the major species is assigned.

HRMS (ESI) calc. for $C_{44}H_{64}N_8O_{10}$ calc. for $[M+H]^+$: 865.4824 m/z, found: 865.4812 m/z. (Δ = -1.38 ppm)

Purification: Purified through preparative HPLC using Gradient 15.

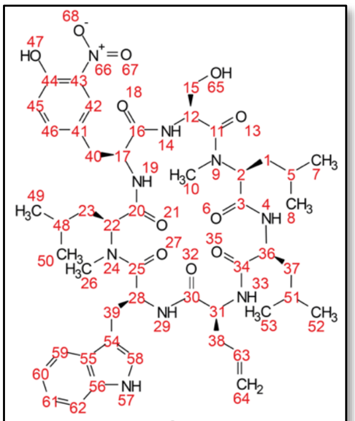

17c

Method: Peptide **17a** was synthesised using the same cyclisation procedure as used for linear peptide **8a**. The linear peptide **17a** was present in solution at 3 mM concentration (10 mL reaction buffer). **17c** was isolated after HPLC purification as a yellowish powder (HPLC conversion (84%), isolated yield (42%)

^{1}H NMR (400 MHz, $(CD_3)_2SO$) δ 10.88 (d, J = 2.4 Hz, 1H, NH-N57), 10.78 – 10.60 (brs, 1H, OH-O65), 9.22 (d, J = 7.0 Hz, 1H, NH-N29), 9.00 (d, J = 4.8 Hz, 1H, NH-N14), 8.51 (d, J = 8.7 Hz, 1H, NH-N4), 8.24 (d, J = 7.7 Hz, 1H, NH-N19), 7.62 – 7.56 (m, 2H, NH-N33 & CH-C42), 7.48 (d, J = 7.9 Hz, 1H, CH-C59), 7.32 (d, J = 7.8 Hz, 1H, CH-C62), 7.18 (dd, J = 8.6, 2.2 Hz, 1H, CH-C46), 7.07 – 7.03 (m, 2H, CH-C58 & CH-C61), 6.98 – 6.92 (m, 2H, CH-C60 & CH-C45), 5.59 – 5.43 (m, 1H, CH-C63), 5.02 – 4.90 (m, 2H, CH_2-C64), 4.87 – 4.71 (m, 3H, CH-C28, CH-C12 & CH-C2), 4.68 (dd, J = 10.2, 4.3 Hz, 1H, CH-C22), 4.60 (td, J = 7.4, 5.8 Hz, 1H, CH-C17), 4.49 – 4.39 (m, 1H, CH-C31), 4.15 – 4.05 (m, 1H, CH-C36), 3.61 – 3.49 (m, 2H, CH_2-C15), 3.28 (dd, J = 13.6, 9.1 Hz, 1H, CH-C39), 3.09 – 3.01 (m, 1H, CH-C39), 2.95 (dd, J = 13.8, 7.6 Hz, 1H, CH-C40), 2.71 – 2.65 (m, 1H, CH-C40), 2.66 (s, 3H, CH_3-C10), 2.45 – 2.39

(m, 1H, CH-C38), 2.39 (s, 3H, CH_3-C26), 2.30 – 2.23 (m, 1H, CH-C38), 1.97 – 1.76 (m, 1H, CH-C1), 1.66 – 1.53 (m, 2H, CH_2-C37), 1.52 – 1.42 (m, 3H, CH-C5, CH-C23 & CH-C51), 1.40 – 1.30 (m, 1H, CH-C1), 1.03 – 0.94 (m, 1H, CH-C48), 0.93 – 0.86 (m, 6H, CH_3-C7 & CH_3-C8), 0.86 – 0.74 (m, 6H, CH_3-C52 & CH_3-C53), 0.44 (d, J = 6.6 Hz, 3H, CH_3-C49 or CH_3-C50), 0.33 (d, J = 6.6 Hz, 3H, CH_3-C49 or CH_3-C50), -0.16 – -0.25 (m, 1H, CH-C23).

^{13}C NMR (101 MHz, $(CD_3)_2SO$) δ 171.43 (C30), 171.34 (C25), 170.66 (C16), 170.51 (C34), 170.05 (C11), 169.07 (C3), 167.50 (C20), 151.05 (C44), 136.49 (C46), 136.02 (C54), 135.93 (C55), 133.03 (C63), 127.73 (C41), 126.97 (aromatic C-Trp), 125.74 (C42), 123.80 (aromatic C-Trp), 121.03 (aromatic C-Trp), 118.91 (aromatic C-Trp), 118.41 (aromatic C-Trp), 118.19 (aromatic C-Trp), 118.08 (C64), 111.41 (aromatic C-Trp), 109.23 (aromatic C-Trp), 60.87 (C15), 57.49 (C2), 57.37 (C22), 53.01 (C17 & C36), 52.03 (C12), 51.24 (C31), 49.90 (C28), 40.89 (C37), 38.07 (C1), 36.92 (C23), 36.76 (C40), 35.75 (C38), 28.67 (C10), 28.40 (C26), 27.23 (C39), 24.40 (C5), 24.27(C51), 23.75 (C48), 23.21, 22.90, 22.78, 22.47, 21.44 & 20.95 (C7, C8, C52, C53, C49 & C50).

As for natural rufomycins this cyclic peptide appears as conformers only the major species is assigned.

HRMS (ESI) calc. for $C_{48}H_{67}N_9O_{11}$ calc. for $[M+H]^+$: 946.5038 m/z, found: 946.5082 m/z. (Δ = 4.64 ppm)

Purification: Purified through preparative HPLC using Gradient 15.

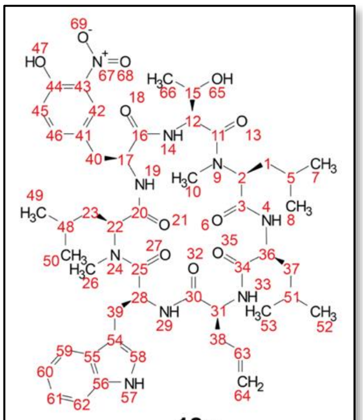

18c

Method: Peptide **18a** was synthesised using the same cyclisation procedure as used for linear peptide **8a**. The linear peptide **18a** was present in solution at 8 mM

concentration (40 mL reaction buffer). **18c** was isolated after HPLC purification as a yellowish powder. (HPLC conversion (78%), isolated yield (35%))

^{1}H NMR (400 MHz, $(CD_3)_2SO$) δ 10.88 (d, *J* = 2.4 Hz, NH-N57), 9.22 (d, *J* = 6.8 Hz, 1H, NH-N29), 8.88 (d, *J* = 5.1 Hz, 1H, NH-N14), 8.52 (d, *J* = 8.7 Hz, 1H, NH-N4), 8.26 (d, *J* = 8.1 Hz, 1H, NH-N19), 7.66 – 7.61 (m, 2H, NH-N33 & CH-C42), 7.47 (d, *J* = 7.9 Hz, 1H, CH-C59), 7.32 (d, *J* = 8.1 Hz, 1H, CH-62), 7.20 (dd, *J* = 8.6, 2.2 Hz, 1H, CH-C46), 7.07 – 7.02 (m, 2H, CH-C58 & CH-C61), 6.97 – 6.91 (m, 2H, CH-C60 & CH-C45), 5.56 – 5.47 (m, 1H, CH-C63), 5.01 – 4.92 (m, 2H, CH_2-C64), 4.83 – 4.73 (m, 3H, CH-C28, CH-C2 & CH-C17), 4.72 – 4.62 (m, 1H, CH-C22), 4.54 (t, *J* = 6.0 Hz, 1H, CH-C12), 4.46 – 4.39 (m, 1H, CH-C31), 4.12 (q, *J* = 7.6 Hz, 1H, CH-C36), 3.84 – 3.76 (m, 1H, CH-C15), 3.28 (dd, *J* = 13.6, 9.3 Hz, 1H, CH-C39), 3.03 (dd, *J* = 13.5, 5.1 Hz, 1H, CH-C39), 2.94 (dd, *J* = 13.6, 6.5 Hz, 1H, CH-C40), 2.66 (m, 4H, CH-C40 & CH_3-C10), 2.45 – 2.39 (m, 1H, CH-C38), 2.32 – 2.28 (m, 1H, CH-C38), 2.32 (s, 3H, CH_3-C26), 2.14 – 2.04 (m, 1H, CH-C1), 1.63 – 1.42 (m, 5H, CH_2-C37, CH-C51, CH-C23 & CH-C5), 1.19 – 1.10 (m, 1H, CH-C1), 1.06 (d, *J* = 6.3 Hz, 3H, CH_3-C66), 1.00 – 0.95 (m, 1H, CH-C48), 0.94 – 0.86 (m, 6H, CH_3-C7 & CH_3-C8), 0.85 – 0.78 (m, 6H, CH_3-C52 & CH_3-C53), 0.43 (d, *J* = 6.6 Hz, 3H, CH_3-C49 or CH_3-C50), 0.32 (d, *J* = 6.6 Hz, 3H, CH_3-C49 or CH_3-C50), -0.19 – -0.34 (m, 1H, CH-C23).

^{13}C NMR (101 MHz, $(CD_3)_2SO$) δ 171.42 (C25), 171.35 (C30), 171.02 (C16), 170.42 (C34), 169.78 (C11), 168.61 (C3), 167.49 (C20), 151.08 (C44), 136.57 (C46), 136.04 (C54), 135.91 (C55), 133.20 (C63), 127.96 (C41), 127.00 (aromatic C-Trp), 125.74 (C42), 123.83 (aromatic C-Trp), 121.05 (aromatic C-Trp), 118.93 (aromatic C-Trp), 118.43 (aromatic C-Trp), 118.21 (aromatic C-Trp), 118.05 (C64), 111.43 (aromatic C-Trp), 109.25 (aromatic C-Trp), 66.43 (C15), 57.46 (C2), 57.38 (C22), 55.77 (C12), 53.09 (C36), 52.77 (C17), 51.38 (C31), 50.04 (C28), 40.96 (C37), 38.55 (C1), 37.17 (C40), 36.91 (C23), 35.61 (C38), 28.84 (C10), 28.29 (C26), 27.31 (C39), 24.83 (C5), 24.31 (C51), 23.82 (C48), 23.57, 22.91, 22.70, 22.11, 21.64 & 20.96 (C7, C8, C52, C53, C49 & C50), 20.23 (C66).

As for natural rufomycins this cyclic peptide appears as conformers only the major species is assigned.

HRMS (ESI) calc. for $C_{49}H_{69}N_9O_{11}$ calc. for $[M+H]^+$: 960.5195 m/z, found: 960.5178 m/z. (*Δ* = -1.76 ppm)

Purification: Purified through preparative HPLC using Gradient 15.

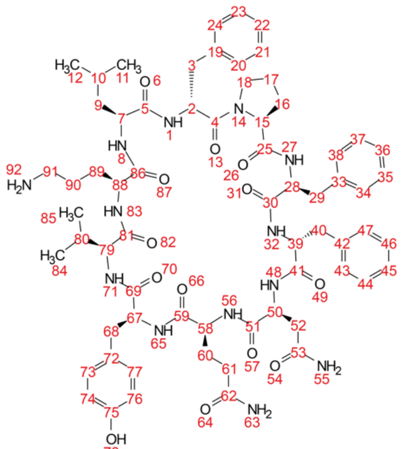

25c

Method: To the linear peptide **25a** (0.02 mmol, 1 equiv., 4 mM) was dissolved in 5 mL of reaction buffer (6 M GdmCl, 50 mM NaH_2PO_4, 1.5 mM EDTA) was added sodium nitrite stock (0.4 mmol, 20 equiv.) at -15 °C under nitrogen. Then the solution was stirred for 20 min at -15 °C. The mixture was gently neutralized with saturated sodium bicarbonate to a final pH 6.8 ~ 7.2. The ice-salt bath was removed, and immediately the solution was diluted with 10 mL of 50% MeCN (0.1% TFA) in water (0.1% TFA). After stirring at room temperature for 15 min, the reaction was filtered and directly purified by prep HPLC using Gradient 14. **25c** was isolated after HPLC purification as a white powder (HPLC conversion (95%), isolated yield (68%)).

^{1}H NMR (CD_3OD, 700 MHz) δ 9.42 (brs, 1H, NH-N48), 9.19 (brs, 1H, NH-N83), 8.77 (d, J = 9.5 Hz, 1H, NH-N65), 8.59 (brs, 1H, NH-N8), 7.89 (d, J = 9.0 Hz, 1H, NH-N71), 7.52 (d, J = 9.1 Hz, 1H, NH-N1), 7.35 (d, J = 7.6 Hz, 2H, H_{Ar}), 7.31 – 7.25 (m, 5H, H_{Ar}), 7.23 (m, 2H, H_{Ar}), 7.19 – 7.12 (m, 6H, H_{Ar}), 6.82 (d, J = 8.0 Hz, 2H, CH-C73 & CH-C77), 6.45 (d, J = 8.0 Hz, 2H, CH-C74 & CH-C76), 5.88 (t, J = 10.8 Hz, 1H, CH-C39), 5.51 (td, J = 9.1, 5.5 Hz, 1H, CH-C88), 5.01 – 4.93 (m, 1H, CH-C7), 4.89 – 4.86 (m, 1H, CH-C79, overlap with residual water peak), 4.68 (t, J = 3.9 Hz, 1H, CH-C50), 4.64 – 4.59 (m, 1H, CH-C67), 4.59 – 4.55 (m, 1H, CH-C2), 4.49 (dd, J = 11.1, 5.1 Hz, 1H, CH-C28), 4.17 – 4.13 (m, 1H, CH-C15), 4.06 (t, J = 5.7 Hz, 1H, CH-C58), 3.37 (t, J = 9.3 Hz, 1H, CH-C18), 3.34 – 3.30 (m, 2H, CH-C40 & CH-C52), 3.28 – 3.20 (m, 2H, CH_2-C29), 3.20 (dd, J = 16.8, 4.7 Hz, 1H CH-C52), 3.13 – 3.06 (m, 2H, CH_2-C68), 3.03 – 2.95 (m, 1H, CH-C91), 2.91 (t, J = 13.1 Hz, 1H, CH-C40), 2.84 – 2.79 (m, 1H, CH-C91), 2.45 – 2.38 (m, 1H, CH-C3), 2.32 – 2.24 (m, 1H, CH-C3), 2.24 – 2.14 (m,

3H, CH-C18, CH-C80 & CH-C89), 2.02 – 1.94 (m, 1H, CH-C61), 1.92 – 1.85 (m, 1H, CH-C89), 1.84 – 1.78 (m, 5H, CH_2-C60, CH-C61 & CH_2-C90), 1.74 – 1.65 (m, 2H, CH-C9 & CH-C10), 1.55 – 1.49 (m, 1H, CH-C9), 1.46 (ddd, *J* = 8.8, 6.7, 4.3 Hz, 1H, CH-C16), 1.34 – 1.28 (m, 1H, CH-C16), 1.15 (d, *J* = 6.7 Hz, 3H, CH_3-C84 or CH_3-C85), 1.13 (d, *J* = 6.8 Hz, 3H, CH_3-C84 or CH_3-C85), 1.10 (d, *J* = 6.4 Hz, 3H, CH_3-C11 or CH_3-C12), 1.07 (d, *J* = 6.4 Hz, 3H, CH_3-C11 or CH_3-C12), 1.05 – 1.02 (m, 1H, CH-17), 0.49 – 0.37 (m, 1H, CH-17).

^{13}C NMR (CD_3OD, 176 MHz) δ 178.32 (C62), 175.18 (C53), 174.12 (C59), 174.00 (C69), 173.91 (C4), 173.67 (C41), 173.48 (C5), 173.34 (C30), 173.06 (C86), 172.63 (C25), 172.26 (C81), 172.09 (C51), 157.22 (C75), 139.10 (C19), 138.91 (C42), 136.96 (C28), 131.02 (C73 & C77), 130.69 (C_{Ar}), 130.19 (C_{Ar}), 129.71 (C_{Ar}), 129.41 (C_{Ar}), 129.37 (C_{Ar}), 129.23 (C72), 128.59 (C_{Ar}), 127.84 (C_{Ar}), 127.77 (C_{Ar}), 116.29 (C74 & C76), 61.57 (C15), 59.66 (C79), 58.57 (C67), 56.77 (C58), 56.16 (C28), 55.40 (C2), 54.86 (C39), 52.74 (C88), 52.42 (C7), 51.06 (C50), 47.73 (C18), 43.17 (C9), 41.52 (C40), 40.49 (C91), 38.91 (C3), 38.39 (C68), 38.11 (C29), 36.60 (C52), 33.39 (C80), 32.87 (C89), 31.68 (C61), 29.91 (C16), 26.90 (C60), 26.41 (C10), 24.45 (C90), 23.97 (C11 or C12), 23.43 (C17), 23.01 (C11 or C12), 19.71 (C84 or C85), 19.26 (C84 or C85).

The spectroscopic data agree with those reported in the literature.

HRMS (ESI) calc. for $C_{66}H_{87}N_{13}O_{13}$ calc. for $[M+H]^+$: 1270.6625 m/z, found: 1270.6639 m/z. (Δ = 1.10 ppm)

Purification: Purified through preparative HPLC using Gradient 14.

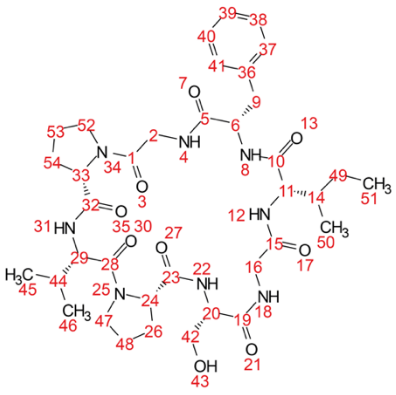

26c

Method: Peptide **26a** was synthesised using the same cyclisation procedure as used for linear peptide **25a**. The linear peptide **26a** was present in solution at 3.88 mM

concentration (15 mL reaction buffer). Following HPLC purification and lyophilisation, **26c** was isolated as a white powder (HPLC conversion (58%); isolated yield (42%)).

Purification: Purified through preparative HPLC using Gradient 21.

^{1}H NMR (400 MHz, $(CD_3)_2SO$) δ 8.21 – 8.11 (m, 1H, amide H), 7.98 – 7.77 (m, 3H, amide H), 7.63 – 7.53 (m, 2H,amide H), 7.32 – 7.13 (m, 5H, aromatic H, CH-C37, CH-C38, CH-C39, CH-C40 & CH-C41), 4.59 (qd, *J* = 9.3, 5.2 Hz, 1H), 4.49 – 4.38 (m, 2H), 4.25 – 4.16 (m, 1H), 4.07 – 3.99 (m, 2H), 3.91 – 3.80 (m, 3H), 3.79 – 3.71 (m, 4H), 3.69 – 3.28 (m, 28H), 3.10 (dt, *J* = 13.7, 5.7 Hz, 1H, CH-C9), 2.88 (td, *J* = 14.0, 9.5 Hz, 1H, CH-C9), 2.54 (s, 1H), 2.17 (tdd, *J* = 12.6, 8.0, 5.5 Hz, 1H), 2.11 – 2.04 (m, 1H), 1.98 (ddt, *J* = 14.3, 9.5, 4.8 Hz, 2H), 1.91 – 1.74 (m, 5H), 1.69 – 1.57 (m, 1H), 1.24 (dddd, *J* = 16.2, 12.8, 8.2, 4.6 Hz, 1H), 0.97 (tq, *J* = 6.6, 3.5 Hz, 1H), 0.89 (td, *J* = 6.5, 4.0 Hz, 3H), 0.82 (d, *J* = 6.7 Hz, 2H), 0.75 (t, *J* = 6.8 Hz, 4H), 0.56 (dd, *J* = 17.6, 6.7 Hz, 3H).

^{13}C NMR (101 MHz, $(CD_3)_2SO$) δ 171.77, 170.69, 170.59, 170.51, 170.34, 169.81, 168.63, 168.42, 137.31, 129.20, 128.13, 126.41, 60.94, 59.91 (C42), 59.68, 58.00, 56.41, 53.78, 47.40, 45.66, 41.82, 41.54, 40.43, 37.88, 36.40, 30.33, 28.57, 26.45, 24.87, 24.47, 24.28, 19.20, 18.91, 17.55, 15.25, 10.98.

This cyclic peptide **26c** appears as conformers only the major species is assigned.

HRMS (ESI) calc. for $C_{37}H_{54}N_8O_9$ calc. for $[M+H]^+$: 755.4092 m/z, found: 755.4119 m/z. (Δ = 3.57 ppm)

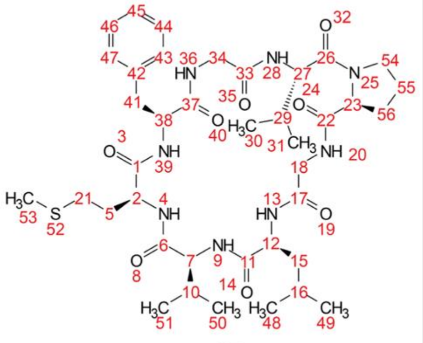

27c

Method: Peptide **27a** was synthesised using the same cyclisation procedure as used for linear peptide **25a**. The linear peptide **27a** was present in solution at 4.2 mM

concentration (10 mL reaction buffer). Following HPLC purification and lyophilisation, **27c** was isolated as a white powder (HPLC conversion (38%); isolated yield (12%)).

Purification: Purified through preparative HPLC using Gradient 12.

^{1}H NMR (400 MHz, $(CD_3)_2SO$) δ 8.73 (dd, *J* = 7.5, 4.8 Hz, 1H, NH-N20), 8.35 – 8.26 (m, 1H, NH-N9), 8.20 – 8.05 (m, 3H, NH-N39, NH-N4 & NH-N36), 7.64 (d, *J* = 8.8 Hz, 1H, NH-N13), 7.30 – 7.14 (m, 5H, aromatic, CH-C43, CH-C44, CH-C45, CH-C46 & CH-C47), 7.09 (d, *J* = 9.0 Hz, 1H, NH-N28), 4.64 – 4.50 (m, 2H, CH-C12 & CH-C27), 4.28 (m, 1H, CH-C38), 4.14 – 4.05 (m, 2H, CH23 & CH-C2), 3.96 (dd, *J* = 16.8, 7.5 Hz, 1H, CH-C18), 3.86 – 3.69 (m, 3H, CH-C34, CH-C7 & CH-C54), 3.67 – 3.42 (m, 2H, CH-C34, CH-C54), 3.38 (dd, *J* = 16.8, 4.7 Hz, 1H, CH-C18), 3.25 (dd, *J* = 14.0, 4.2 Hz, 1H, CH-C41), 2.90 (dd, *J* = 14.0, 10.9 Hz, 1H, CH-C41), 2.39 – 2.22 (m, 2H, CH_2-C21), 2.17 – 1.98 (m, 4H, CH-C10, CH-C29, CH-C5 & CH-C55), 1.96 (s, 3H, CH_3-C53), 1.85 (m, 1H, CH-C55), 1.79 – 1.64 (m, 3H, CH-C5 & CH_2-C56), 1.59 – 1.43 (m, 3H, CH_2-C15 & CH-C16), 0.98 – 0.75 (m, 18H, CH_3-C30, CH_3-C31, CH_3-C50, CH_3-C51, CH_3-C48 & CH_3-C49).

^{13}C NMR (101 MHz, $(CD_3)_2SO$) δ 173.41 (C11), 172.89 (C6), 172.41 (C22), 171.55 (C1), 171.30 (C37), 169.95 (C26), 168.56 (C17), 168.39 (C33), 138.02 (C42), 128.82 (C43 & C47), 128.09 (C44 & C46), 126.41 (C45), 61.20 (C7), 60.97 (C23), 54.75 (C27), 54.66 (C38), 53.40 (C2), 50.33 (C12), 47.48 (C54), 43.17 (C34), 42.83 (C18), 40.55 (C15), 36.12 (C41), 30.31 (C29), 29.45 (C21), 28.81 (C56), 28.49 (C10), 25.01 (C55), 24.00 (C16), 23.12 (C48 or C49), 22.01 (C48 or C49), 19.47, 19.23, 18.93, 17.65 (Val C50, C51, C30, C31), 14.36 (C53).
The spectroscopic data agree with those reported in the literature.
HRMS (ESI) calc. for $C_{39}H_{60}N_8O_8$ calc. for $[M+H]^+$: 801.4333 m/z, found: 801.4362 m/z. (*Δ* = 3.62 ppm)

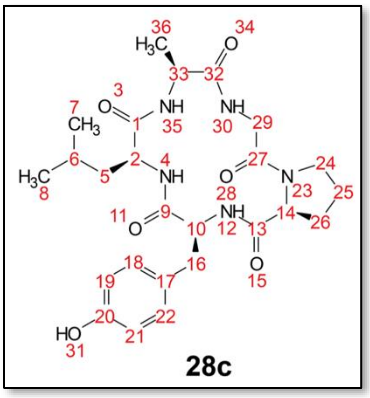

Method: Peptide **28a** was synthesised using the same cyclisation procedure as used for linear peptide **25a**. The linear peptide **26a** was present in solution at 4.9 mM concentration (10 mL reaction buffer). Following HPLC purification and lyophilisation, **28c** was isolated as a white powder (HPLC conversion (77%); isolated yield (51%)).

Purification: Purified through preparative HPLC using Gradient 20.

^{1}H NMR (400 MHz, $(CD_3)_2SO$) δ 9.18 (brs, 1H, OH-O31), 8.51 (d, *J* = 7.8 Hz, 1H, NH-N35), 8.01 (d, *J* = 7.6 Hz, 1H, NH-N4), 7.83 (t, *J* = 4.8 Hz, 1H, NH-N30), 7.28 (d, *J* = 9.3 Hz, 1H, NH-N12), 6.97 – 6.91 (m, 2H, CH-C18 & CH-C22), 6.65 – 6.60 (m, 2H, CH-C19 & CH-C21), 4.47 – 4.35 (m, 1H, CH-C10), 4.14 – 4.05 (m, 1H, CH-C33), 4.05 – 3.90 (m, 3H, CH-C14, CH-C29 & CH-C2), 3.85 – 3.76 (m, 1H, CH-C24), 3.56 – 3.46 (m, 2H, CH-C29 & CH-C24), 2.82 (dd, *J* = 13.6, 6.9 Hz, 1H, CH-C16), 2.72 (dd, *J* = 13.6, 9.1 Hz, 1H, CH-C16), 2.07 – 1.94 (m, 1H, CH-C26), 1.83 (p, *J* = 6.8 Hz, 2H, CH_2-C25), 1.66 – 1.56 (m, 1H, CH-C5), 1.54 – 1.38 (m, 2H, CH-C26, CH-C5), 1.37 – 1.27 (m, 1H, CH-C6), 1.24 (d, *J* = 7.1 Hz, 3H, CH_3-C36), 0.93 – 0.75 (m, 6H, CH_3-C7 & CH_3-C8).

^{13}C NMR (101 MHz, $(CD_3)_2SO$) δ 172.56 (C32), 171.42 (C1), 170.40 (C9 & C13), 167.68 (C27), 155.82 (C20), 129.88 (C18 & C22), 127.30 (C17), 114.81 (C19 & C21), 61.18 (C14), 55.31 (C10), 53.81 (C2), 48.71 (C33), 46.57 (C24), 41.90 (C29), 40.09 (C5, overlap with solvent peaks), 36.88 (C16), 29.10 (C26), 24.43 (C6), 24.22 (C25), 22.49 (C7 or C8), 22.08 (C7 or C8), 16.66 (C36).

HRMS (ESI) calc. for $C_{25}H_{35}N_5O_6$ calc. for $[M+H]^+$: 502.2666 m/z, found: 502.2666 m/z. (Δ = 0 ppm)

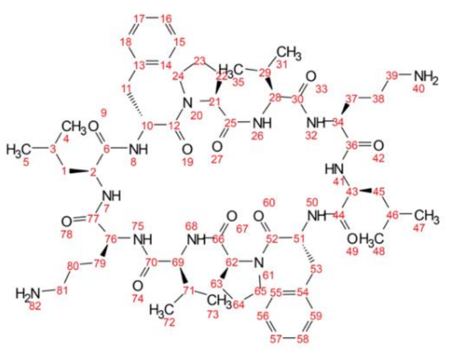

29c

Method: Peptide **29a** was synthesised using the same cyclisation procedure as used for linear peptide **25a**. The linear peptide **29a** was present in solution at 5 mM concentration (10 mL reaction buffer). Following HPLC purification and lyophilisation, **29c** was isolated as a white powder (HPLC conversion (92%); isolated yield (63%)).
Purification: Purified through preparative HPLC using Gradient 14.

^{1}H NMR (400 MHz, $(CD_3)_2SO$) δ 9.09 (d, *J* = 3.6 Hz, 2H, NH-N8 & NH-N50), 8.72 (d, *J* = 9.2 Hz, 2H, NH-N75 & NH-N32), 8.35 (d, *J* = 9.1 Hz, 2H, NH-N41 & NH-N7), 7.79 (br, 6H, NH-N40 & NH-N82), 7.32 – 7.20 (m, 12H, NH-N26 & NH-N68, aromatic CH-C14-18 & CH-C55-59), 4.78 (td, *J* = 9.4, 5.2 Hz, 2H, CH-C34 & CH-C76), 4.64 – 4.54 (m, 2H, CH-C2 & CH-C43), 4.43 – 4.33 (m, 4H, overlap, CH-C28, CH-C69, CH-C10 & CH-C51), 4.33 – 4.26 (m, 2H, CH-C21 & CH-C62), 3.59 (overlap, 2H, CH-C24 & CH-C65), 2.98 (dd, *J* = 12.8, 5.4 Hz, 2H, CH-C53, CH-C11), 2.92 – 2.80 (m, 4H, CH-C53, CH-C11, CH-C39 & CH-C81), 2.80 – 2.72 (m, 2H, CH-C39 & CH-C81), 2.48 – 2.39 (m, 2H, CH-C24 & CH-C65), 2.08 (dt, *J* = 13.8, 6.9 Hz, 2H, CH-C29 & CH-C71), 1.99 – 1.89 (m, 2H, CH-C22 & CH-C63), 1.81 – 1.69 (m, 2H, CH-C37 & CH-C79), 1.68 – 1.57 (m, 4H, CH-C23, CH-C64, CH-C80 & CH-C38), 1.57 – 1.44 (m, 8H, CH-C23, CH-C64, CH-C22, CH-C63, CH-C80, CH-C38, CH-C37 & CH-C79), 1.45 – 1.35 (m, 2H, CH-C3 & CH-C46), 1.35 – 1.22 (m, 4H, CH_2-C1 & CH_2-C45), 0.91 – 0.63 (m, 24H, CH_3-C4, CH_3-C5, CH_3-C31, CH_3-C35, CH_3-C47, CH_3-C48, CH_3-C72 & CH_3-C73).

^{13}C NMR (101 MHz, $(CD_3)_2SO$) δ 171.69 (C44 & C6), 171.21 (C12 & C52), 170.92 (C30 & C70), 170.17 (C36 & C77), 169.74 (C25 & C66), 136.18 (C13 & C54), 129.34 & 128.29 (aromatic C14, C15, C17, C18, C55, C56, C58 & C59), 126.98 (C16 & C57), 59.98 (C21 & C62), 56.92 (C28 & C69), 53.96 (C10 & C51), 50.82 (C34 & C76), 49.48

(C2 & C43), 46.11 (C24 & C65), 40.92 (C1 & C45), 38.65 (C39 & C81), 35.68 (C11 & C53), 31.03 (C29 & C71), 29.71 (C79 & C37), 29.09 (C22 & C63), 23.97 (C3 & C46), 23.11 & 23.05 (C64, C23, C38 & C80), 22.83 (Methyl Val or Leu), 22.52 (Methyl Val or Leu), 18.97 (Methyl Val or Leu), 18.10 (Methyl Val or Leu).

HRMS (ESI) calc. for $C_{60}H_{92}N_{12}O_{10}$ calc. for $[M+H]^+$: 1141.7137 m/z, found: 1141.7142 m/z. (Δ = 0.44 ppm)

The spectroscopic data agree with those reported in the literature**.**

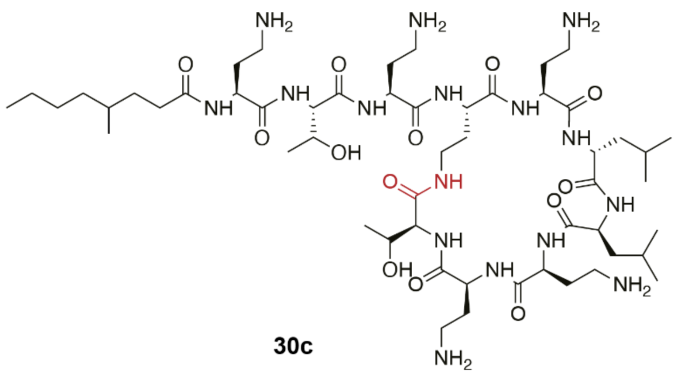

30c

Method: Linear peptide **30a** was synthesised via SPPS using the same procedure outlined in **section 2.2.3** including global deprotection as in **section 2.2.4**. The final coupling step was used to introduce the lipid tail. 4-methyloctanoic acid (CAS: 54947-74-9, Alfa Aesar) (4 equiv.) was coupled to the *N*-terminus of the resin bound peptide at room temperature for 1 h with DIC (4 equiv.) and Oxyma (4 equiv.). The linear peptide **30a** was cyclised using the same method as used to cyclise **25a**. The linear peptide **30a** was present in solution at 3 mM concentration (25 mL reaction buffer), 50 mL of 50% MeCN in water was added on neutralisation to pH 7. Following HPLC purification and lyophilisation, **30c** was isolated as a white powder (HPLC conversion (56%); isolated yield (3%)).

Purification: Purified through semi-preparative HPLC using Gradient 23.

^{1}H NMR (400 MHz, (D_2O)): The spectroscopic data agree with commercial standard (Fig. S26A). The standard **30g** was purified from a sample of commercial colistin through semi-preparative HPLC using same method.

HRMS (ESI) calc. for $C_{53}H_{100}N_{16}O_{13}$ calc. for $[M+H]^+$: 1169.7734 m/z, found: 1169.7736 m/z. (Δ = 0.17 ppm)

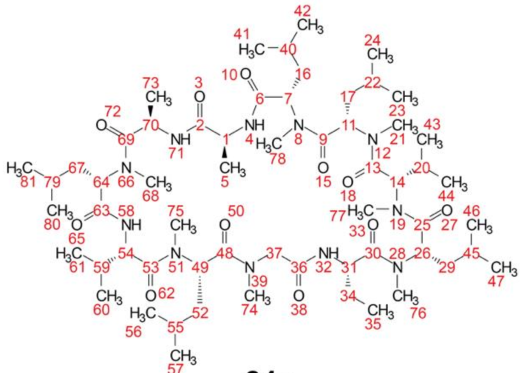

31c

Method: Peptide **31a** was synthesised using the same cyclisation procedure as used for linear peptide **8a**. The linear peptide **31a** was present in solution at 4 mM concentration (20 mL reaction buffer). Following HPLC purification and lyophilisation, **31c** was isolated as a white powder (HPLC conversion (61%); isolated yield (22%)).

Purification: Purified through preparative HPLC using Gradient 19.

^{1}H NMR (400 MHz, $(CD_3)_2SO$) δ 8.32 – 8.22 (m, 2H), 7.93 (d, *J* = 8.8 Hz, 1H), 7.47 (d, *J* = 6.0 Hz, 1H), 5.40 (dd, *J* = 11.2, 3.7 Hz, 1H), 5.26 (dd, *J* = 8.9, 5.7 Hz, 1H), 5.20 – 5.11 (m, 3H), 5.10 – 4.96 (m, 3H), 4.78 – 4.65 (m, 2H), 4.42 – 4.30 (m, 1H), 4.18 – 4.09 (m, 1H), 2.90 (s, 3H), 2.86 – 2.77 (m, 15H), 2.68 (s, 3H), 2.28 – 2.14 (m, 2H), 2.01 – 1.86 (m, 3H), 1.81 – 1.09 (m, 20H), 1.00 – 0.63 (m, 45H).

^{13}C NMR (101 MHz, $(CD_3)_2SO$) δ 172.80, 172.65, 171.96, 171.23, 170.53, 170.18, 170.02, 169.57, 168.77, 57.33, 54.35, 53.94, 53.48, 53.22, 51.97, 50.88, 49.98, 49.51, 48.30, 43.69, 37.65, 37.53, 37.15, 36.85, 33.45, 31.20, 30.26, 29.57, 29.52, 29.36, 24.67, 24.53, 24.48, 24.24, 24.00, 23.61, 23.48, 23.37, 23.34, 23.22, 23.17, 23.08, 22.23, 22.14, 21.20, 20.92, 20.85, 20.50, 19.31, 18.99, 18.79, 18.13, 17.94, 17.02, 10.32.

HRMS (ESI) calc. for $C_{59}H_{107}N_{11}O_{11}$ calc. for $[M+H]^+$: 1146.8230 m/z, found: 1146.8220 m/z. (Δ = -0.87 ppm)

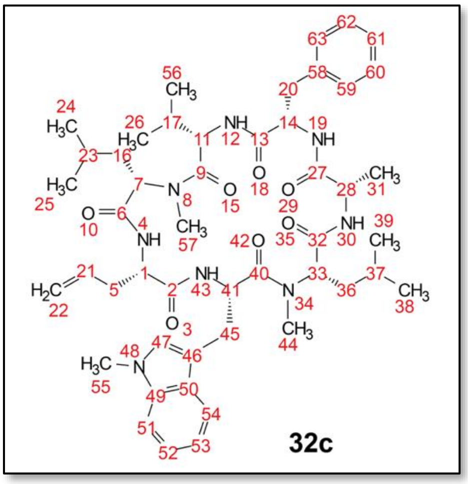

Method: Peptide **32a** was synthesised using the same cyclisation procedure as used for linear peptide **8a**. The linear peptide **32a** was present in solution at 2 mM concentration (20 mL reaction buffer). Following HPLC purification and lyophilisation, **32c** was isolated as a white powder (HPLC conversion (95%); isolated yield (50%)).

Purification: Purified through preparative HPLC using Gradient 14.

^{1}H NMR (400 MHz, $(CD_3)_2SO$) δ 9.13 (d, *J* = 7.5 Hz, 1H, NH-N12), 9.06 (d, *J* = 4.6 Hz, 1H, NH-N43), 8.37 (d, *J* = 8.1 Hz, 1H, NH-N30), 8.03 (d, *J* = 7.8 Hz, 1H, NH-N4), 7.65 (d, *J* = 8.1 Hz, 1H, NH-N19), 7.46 (d, *J* = 8.0 Hz, 1H, CH-C54), 7.37 (d, *J* = 8.0 Hz, 1H, CH-C51), 7.21 – 6.93 (m, aromatic, 8H, CH-C59 CH-C63 CH-C47 CH-C60 CH-C62 CH-C61, CH-C52 & CH-C53), 5.58 (ddt, *J* = 17.2, 10.1, 7.1 Hz, 1H, CH-C21), 5.17 – 5.07 (m, 1H, CH-C7), 4.94 – 4.80 (m, 3H, CH_2-C22 & CH-C41), 4.65 – 4.54 (m, 2H, CH-C14 & CH-C1), 4.33 (dd, *J* = 10.1, 7.7 Hz, 1H, CH-C11), 4.25 (dd, *J* = 10.5, 4.3 Hz, 1H, CH-C33), 3.98 – 3.90 (m, 1H, CH-C28), 3.72 (s, 3H, CH_3-C55), 3.19 – 2.99 (m, 2H, CH_2-C45), 2.99 – 2.91 (m, 2H, CH_2-C20), 2.68 (s, 3H, CH_3-C57), 2.62 (s, 3H, CH_3-C44), 2.26 – 2.05 (m, 5H, CH-C17, CH_2-C5, CH_2-C16), 1.52 – 1.37 (m, 2H, CH-C36 & CH-C23), 1.08 (d, *J* = 7.0 Hz, 3H, CH_3-C31), 1.00 – 0.78 (m, 13H, CH_3-C26, CH_3-C56, CH-C37, CH_3-C24, CH_3-C25), 0.48 (d, *J* = 6.6 Hz, 3H, CH_3-C38 or CH_3-C39), 0.33 (d, *J* = 6.6 Hz, 3H, CH_3-C38 or CH3-C39), -0.34 (ddd, *J* = 12.9, 8.9, 4.1 Hz, 1H, CH-C36).

^{13}C NMR (101 MHz, $(CD_3)_2SO$) δ 172.35 (C13), 171.83 (C40), 171.10 (C27), 171.06 (C9), 170.51 (C2), 168.60 (C32), 167.53 (C6), 137.35 (C58), 136.49 (C49), 133.52

(C21), 129.54 (C59 & C63), 128.43 (aromatic C), 127.91 (C60 & C62), 127.66 (aromatic C), 126.34 (aromatic C), 121.34 (C47), 118.77 (C54), 118.60 (aromatic C), 117.76 (C22), 109.59 (C51), 108.25 (aromatic C), 57.28 (C7), 57.12 (C33), 55.33 (C11), 53.35 (C14), 51.38 (C1), 50.55 (C41), 49.85 (C28), 38.71 (C16), 37.65 (C5), 37.25 (C36), 37.14 (C20), 32.37 (C55), 29.32 (C17), 28.83 (C44), 28.60 (C57), 27.47 (C45), 24.66 (C23), 23.99 (C37), 23.44 (C24 or C25), 23.18 (C38 or C39), 22.04 (C24 or C25), 20.76 (C38 or C39), 19.73 (C56 or C26), 19.21 (C56 or C26), 16.78 (C31).

HRMS (ESI) calc. for $C_{48}H_{68}N_8O_7$ calc. for $[M+H]^+$: 869.5289 m/z, found: 869.5289 m/z. (Δ = 0 ppm)

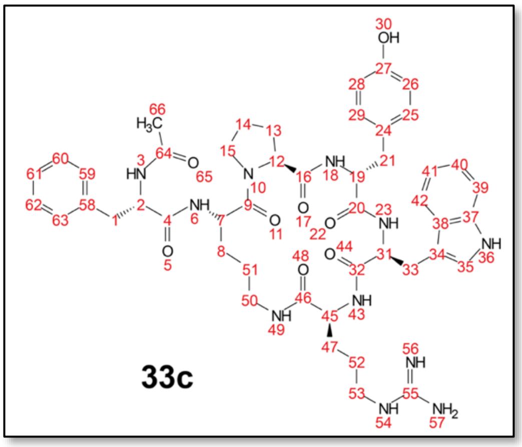

Method: Peptide **33a** was synthesised using the same cyclisation procedure as used for linear peptide **25a**. The linear peptide **33a** was present in solution at 3 mM concentration (10 mL reaction buffer). Following HPLC purification and lyophilisation, **33c** was isolated as a white powder (HPLC conversion (95%); isolated yield (62%)).

Purification: Purified through preparative HPLC using Gradient 17.

^{1}H NMR (400 MHz, $(CD_3)_2SO$) δ 10.86 (d, *J* = 2.4 Hz, 1H, NH-N36), 8.39 (d, *J* = 6.5 Hz, 1H, NH-N23), 8.11 – 8.06 (m, 2H, NH-N6 & NH-N3), 7.89 (d, *J* = 6.5 Hz, 1H, NH-N18), 7.82 (d, *J* = 8.4 Hz, 1H, NH-N43), 7.70 (t, *J* = 5.8 Hz, 1H, NH-N49), 7.54 (d, *J* = 7.8 Hz, 1H, CH-C42), 7.35 (d, *J* = 8.1 Hz, 1H, CH-C39), 7.27 – 7.14 (m, 6H, CH-C59, CH-C60, CH-C61, CH-C62, CH-C63 & CH-C35), 7.13 – 7.07 (m, 1H, CH-C40), 7.07 – 6.99 (m, 2H, CH-C4 & NH-N54), 6.40 (d, *J* = 8.5 Hz, 2H, CH-C29 & CH-C25), 6.29 (d, *J* = 8.5 Hz, 2H, CH-C26 & CH-C28), 4.58 (dd, *J* = 7.9, 2.5 Hz, 1H, CH-C12), 4.51 – 4.47 (m, 2H, CH-C7 & CH-C2), 4.41 (q, *J* = 5.9 Hz, 1H, CH-C19), 4.14 – 4.07 (m,

2H, CH-C31 & CH-C45), 3.58 (m, 1H, CH-C15), 3.42 (m, 1H, CH-C15), 3.35 – 3.23 (m, 1H, CH-C53), 3.20 – 3.12 (m, 1H, CH-C33), 3.11 – 3.04 (m, 2H, CH_2-C50), 3.05 – 2.98 (m, 1H, CH-C33), 2.94 (dd, *J* = 13.8, 4.4 Hz, 1H, CH-C1), 2.71 – 2.66 (m, 2H, CH-C1 & CH-C53), 2.64 – 2.52 (m, 2H, CH_2-C21), 2.08 – 1.95 (m, 1H, CH-C13), 1.83 (m, 3H, CH_2-C14, CH-C47 & CH-C8), 1.77 – 1.66 (m, 5H, CH-C13), 1.65 – 1.52 (m, 2H, CH-C47 & CH-C8), 1.52 – 1.23 (m, 5H, CH_2-C51, CH_2-C52).

^{13}C NMR (101 MHz, $(CD_3)_2SO$) δ 171.78 (C32), 171.47 (C20), 170.90 (C4), 170.69 (C16), 170.64 (C55), 170.51 (C9), 169.23 (C64), 156.85 (C46), 155.76 (C27), 137.94 (C58), 136.35 (C37), 130.12 (C29 & C25), 129.19 (C59 & C63), 128.04 (C60 & C62), 127.01 (C35), 126.53 (C24), 126.28 (C61), 124.18 (C38), 121.16 (C40), 118.53 (C41), 118.09 (C42), 114.77 (C26 & C28), 111.60 (C39), 109.99 (C34), 58.64 (C12), 55.78 (C31), 54.76 (C19), 53.80 (C2), 52.80 (C45), 49.95 (C7), 46.68 (C15), 40.43 (C50), 38.19 (C53), 37.55 (C1), 36.60 (C21), 28.37 (methylene), 27.87 (methylene), 26.94 (C33), 26.79 (C13), 25.49 (methylene), 24.58 (methylene), 24.37 (C14), 22.48 (C66).

HRMS (ESI) calc. for $C_{47}H_{59}N_{11}O_8$ calc. for $[M+H]^+$: 906.4626 m/z, found: 906.4641 m/z. (Δ = 1.65 ppm)

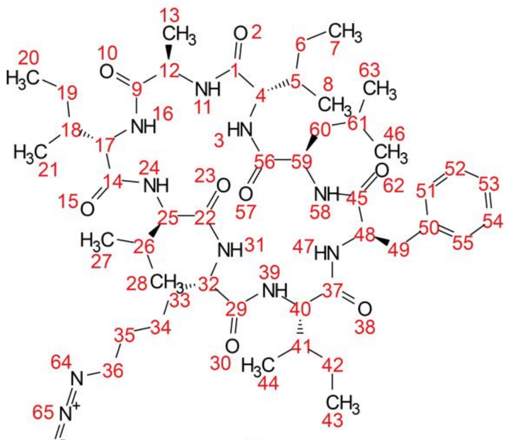

35c

Method: Peptide **35a** was synthesised using the same cyclisation procedure as used for linear peptide **8a**. The linear peptide **35a** was present in solution at 5 mM concentration (15 mL reaction buffer). Following HPLC purification and lyophilisation, **35c** was isolated as a white powder (HPLC conversion (59%); isolated yield (46%)).

Purification: Purified through preparative HPLC using Gradient 22.

^{1}H NMR (700 MHz, $(CD_3)_2SO$) δ 8.40 (d, *J* = 8.6 Hz, 1H, NH-N47), 8.16 (d, *J* = 7.9 Hz, 1H, NH-N16), 8.04 (d, *J* = 5.9 Hz, 1H, NH-N11), 7.93 (d, *J* = 8.1 Hz, 1H, NH-N24), 7.85 – 7.76 (d, *J* = 44.5 Hz, 2H, NH-N31 & NH-N39), 7.74 (d, *J* = 7.9 Hz, 1H, NH-N58), 7.27 – 7.15 (m, 6H, aromatic H & NH-N3), 4.43 (ddd, *J* = 12.0, 8.6, 3.7 Hz, 1H, CH-C48), 4.34 – 4.27 (m, 2H, CH-C59 & CH-C32), 4.24 (p, *J* = 6.8 Hz, 1H, CH-C12), 4.17 – 4.11 (m, 2H, CH-C17, CH-C4), 4.10 – 4.04 (m, 1H, CH-C25), 3.87 (t, *J* = 6.3 Hz, 1H, CH-C40), 3.30 – 3.22 (m, 3H, CH-C49 & CH_2-C36), 2.67 (dd, *J* = 14.1, 11.5 Hz, 1H, CH-C49), 1.98 – 1.91 (m, 1H, CH-C26), 1.83 – 1.75 (m, 2H), 1.71 (dtd, *J* = 10.0, 6.9, 3.1 Hz, 1H), 1.66 (dq, *J* = 8.8, 6.4 Hz, 1H), 1.60 (ddd, *J* = 13.2, 8.6, 4.6 Hz, 1H), 1.49 – 1.37 (m, 7H), 1.34 – 1.16 (m, 8H, CH_3-C13), 1.11 (dddd, *J* = 26.9, 13.4, 9.2, 7.1 Hz, 2H), 0.91 (d, *J* = 6.6 Hz, 3H), 0.86 (dd, *J* = 6.7, 4.4 Hz, 7H), 0.81 (ddt, *J* = 18.2, 7.5, 3.8 Hz, 17H), 0.68 (t, *J* = 7.4 Hz, 3H), 0.47 (d, *J* = 6.8 Hz, 3H).

^{13}C NMR (101 MHz, $(CD_3)_2SO$) δ 172.42, 172.36 x2 (C9, C56 or C29), 171.36 (C14), 170.90 (C45), 170.81 (C37), 170.60 (C22), 170.53 (C1), 138.07 (C50), 129.00 (C51 & C55), 128.09 (C52 & C54), 126.23 (C53), 58.95 (C25), 57.87 (Ile-α), 57.54 (C17), 57.05 (Ile-α), 54.24 (C48), 52.13 (C59), 51.81 (C32), 50.49 (C36), 48.31 (C1), 40.31 (C60), 36.50 (C49), 36.29, 35.84 (C41), 35.39, 31.40, 30.48 (C26), 27.50, 26.88, 24.58, 24.31, 24.20, 24.14, 23.14, 22.28, 21.51, 19.43, 18.44 (C13), 17.80, 15.32, 15.08, 14.76, 11.06, 10.81.

HRMS (ESI) calc. for $C_{47}H_{78}N_{11}O_8$ calc. for $[M+H]^+$: 924.6029 m/z, found: 924.6007 m/z. (*Δ* = -1.95 ppm)

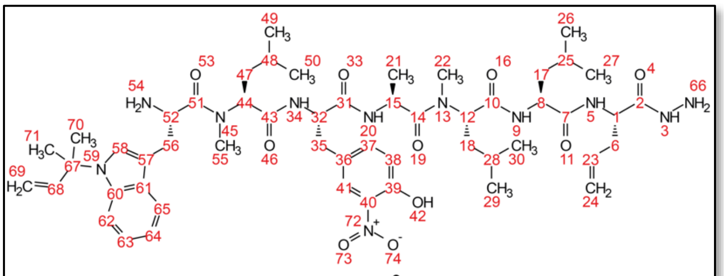

9a

^{1}H NMR (400 MHz, 90% CD_3CN 10% D_2O) δ 7.91 (d, J = 2.44 Hz, 1H, CH-C41), 7.56 – 7.45 (m, 3H, CH-C65, CH-C64 & CH-C37), 7.25 (s, 1H, CH-C58), 7.12 – 7.00 (m, 3H, CH-C62, CH-C63 & CH-C38), 6.15 – 6.04 (m, 1H, CH-C68), 5.80 – 5.64 (m, 1H,

CH-C23), 5.23 – 5.01 (m, 4H, CH_2-C69 & CH_2-C24), 4.99 – 4.85 (m, 1H, CH-C12), 4.78 (qd, J = 6.3, 3.5 Hz, 1H, CH-C15), 4.58 – 4.44 (m, 3H, CH-C44, CH-C32 & CH-C52), 4.43 – 4.34 (m, 1H, CH-C1), 4.33 – 4.22 (m, 1H, CH-C8), 3.19 – 3.07 (m, 3H, CH_2-C56 & CH-C35), 3.00 – 2.87 (m, 4H, CH-C35 & CH_3-C22), 2.75 – 2.70 (m, 3H, CH_3-C55), 2.63 – 2.35 (m, 2H, CH_2-C6), 1.80 – 1.66 (m, 6H, CH_3-C70 & CH_3-C71), 1.66 – 1.20 (m, 12H, CH_2-C47, CH_2-C18, CH_2-C17, CH-C48, CH-C28, CH-C25 & CH_3-C21), 0.97 – 0.74 (m, 18H, CH_3-C26, CH_3-C27, CH_3-C29, CH_3-C30, CH_3-C49 & CH_3-C50).

^{13}C NMR (101 MHz, 90% CD_3CN 10% D_2O) δ 174.21 (C14), 173.61, 172.16, 171.34 (C2), 170.84 (C31), 170.13, 170.06 (C51), 153.53 (C39), 144.58 (C68), 139.13 (C37), 136.13, 134.39, 133.61 (C23), 130.84, 130.47, 129.37, 126.32 (C58), 125.94 (C41), 121.64, 120.23, 119.79 118.96, 118.67 (C24), 115.76, 114.71, 113.74 (C69), 105.45 (C57), 59.70 (C67), 58.19 (C44), 56.18 (C12), 54.55 (C35), 52.54 (C8), 51.98 (C1), 51.54 (C52), 46.39 (C15), 40.35, 37.27, 36.74, 36.69, 35.99 (C35), 35.59, 33.26 (C55), 31.64 (C22), 29.89, 27.70 (C70), 27.65 (C71), 26.87 (C56), 25.07, 25.04 & 25.02 (Leu C-**γ**), 23.10, 22.94, 22.83, 21.72, 21.66 & 21.25 (Leu C-**δ**), 17.26 (C21).

This linear peptide **9a** appears as multiple conformers only the major species is assigned.

Purification: Purified through semi preparative HPLC using Gradient 10.

HRMS (ESI) calc. for $C_{53}H_{79}N_{11}O_{10}$ calc. for $[M+H]^+$: 1030.6090 m/z, found: 1030.6094 m/z. (*Δ* = 0.39 ppm)

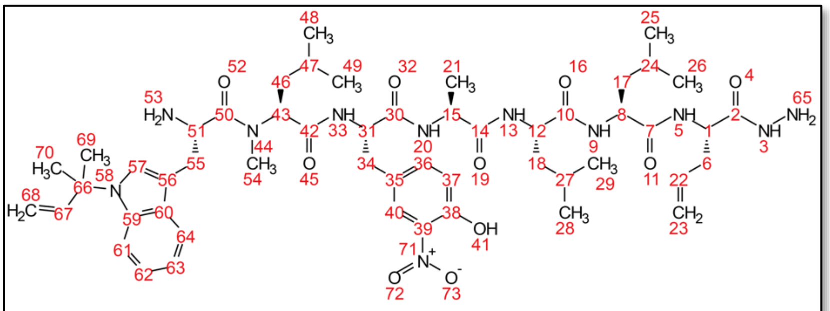

14a

^{1}H NMR (400 MHz, 90% CD_3CN 10% D_2O) δ 7.92 (d, *J* = 2.3 Hz, 1H, CH-C40), 7.53 – 7.46 (m, 3H CH-C64 CH-C63 & CH-C36), 7.26 (s, 1H, CH-C57), 7.13 – 7.02 (m, 3H, CH-C61 CH-C62 & CH-C37), 6.11 (dd, *J* = 17.5, 10.7 Hz, 1H, CH-C67), 5.73 – 5.63

(m, 1H, CH-C22), 5.22 – 5.11 (m, 2H, CH_2-C68), 5.10 – 4.99 (m, 2H, CH_2-C23), 4.74 (dd, *J* = 8.8, 6.5 Hz, 1H, CH-C43), 4.47 (dd, *J* = 8.0, 6.4 Hz, 1H, CH-C51), 4.35 (dd, *J* = 9.3, 5.8 Hz, 1H, CH-C31), 4.26 (dd, *J* = 9.0, 5.2 Hz, 1H, CH-C1), 4.20 – 4.13 (m, 3H, CH-C15, CH-C12 & CH-C8), 3.15 – 2.93 (m, 4H, CH_2-C34 & CH_2-C55), 2.78 (s, 3H, CH_3-C54), 2.54 – 2.44 (m, 1H, CH-C6), 2.37 – 2.26 (m, 1H, CH-C6), 1.72 – 1.68 (m, 6H, CH_3-C69 & CH_3-C70), 1.66 – 1.47 (m, 8H,CH-C24, CH-C27, CH_2-C17, CH_2-C18 & CH_2-C46), 1.35 (d, *J* = 7.2 Hz, 3H, CH_3-C21), 1.32 – 1.23 (m, 1H, CH-C47), 0.94 – 0.78 (m, 18H, CH_3-C25, CH_3-C26, CH_3-C28, CH_3-C29, CH_3-C48 & CH_3-C49).

^{13}C NMR (101 MHz, 90% CD_3CN 10% D_2O) δ 174.91 (C14), 174.52 (C10 or C7), 173.72 (C10 or C7), 172.69 (C30), 172.14 (C42), 171.38 (C2), 169.96 (C50), 153.58 (C38), 144.44 (C67), 138.90 (C36), 136.12 (C aromatic), 134.39 (C aromatic), 133.82 (C22), 129.84 (C35), 129.21 (C aromatic), 126.30 (C57), 125.77 (C40), 121.64 (C aromatic), 120.33 (C aromatic), 119.76 (C aromatic), 118.82 (C aromatic), 118.49 (C23), 114.73 (C aromatic), 113.77 (C68), 105.27 (C56), 59.67 (C66), 57.21 (C43), 56.19 (C31), 53.59 (C12 or C8), 53.11 (C12 or C8), 52.34 (C1), 51.62 (C51), 50.96 (C15), 40.21 (C17 or C18), 40.14 (C17 or C18), 36.88 (C46), 35.71 (C6), 35.52 (C34), 32.63 (C54), 27.66 (C69 & C70), 26.73 (C55), 25.08, 24.94, 24.81, 22.91, 22.88, 22.81, 21.58, 21.26 & 21.02 (C47, C48, C49, C27, C28, C29, C24, C25 & C26) 16.84 (C21).

Purification: Purified through semi preparative HPLC using Gradient 11.

HRMS (ESI) calc. for $C_{52}H_{77}N_{11}O_{10}$ calc. for $[M+H]^+$: 1016.5933 m/z, found: 1016.5936 m/z. (Δ = 0.30 ppm)

A.

α proton (residue)	Peptide 9a (ppm)	Peptide 14a (ppm)	*Δ (ppm)*	Alpha helix	Beta sheet	Random coil
N-prenyl-*L*-Trp	4.55-4.44	4.47	0.025	4.38[a]	5.19[a]	4.66[a]
N-Me-*L*-Leu	4.58-4.44	4.74	-0.230			
3-Nitro-*L*-Tyr	4.58-4.44	4.35	0.160	4.09[b]	5.10[b]	4.55[b]
***L*-Ala**	**4.78**	**4.20-4.13**	**0.615**	**4.03**	**4.77**	**4.32**
***N*-Me-*L*-Leu 9a or *L*-Leu 14a**	**4.99-4.85**	**4.20-4.13**	**0.755**	**4.00**	**4.82**	**4.36**
L-Leu	4.33-4.22	4.20-4.13	0.110	4.00	4.82	4.36
L-allyl-Gly	4.43-4.34	4.26	0.125			

B.

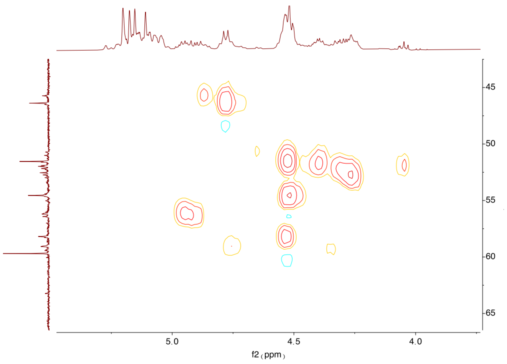

C.

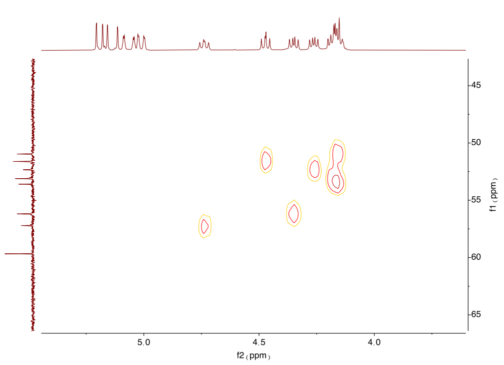

D.

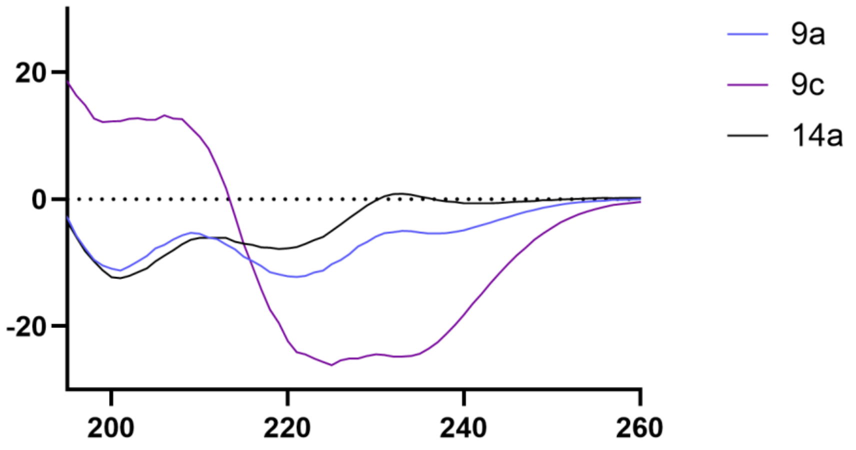

Scheme S1: A. Comparison of α proton region of peptide **9a** and peptide **14a**. Secondary structure chemical shifts taken from report by Wishart.[8] For non-proteinogenic amino acids, values for closest proteinogenic amino acid is taken [a] L-Trp, [b] L-Tyr. **B.** Zoom-in HSQC spectrum of *α*-proton region of peptide **9a.** showing multiple conformers are present. **C.** Zoom-in HSQC spectrum of *α*-proton region of peptide **14a**. **D.** CD spectra of linear hydrazide peptides **9a** and **14a** in comparison with cyclized peptide **9c**. To measure the CD spectra, pure linear hydrazide peptides **14a**, **9a** and cyclic peptide **9c** were dissolved in 90% ACN in water and diluted to a concentration of 25 µM. Samples were analyzed using a Circular Dichroism Spectrometer Chirascan V100 CD at 25°C. CD scans were performed in triplicate from 185 to 280 nm at a bandwidth of 1 nm using a quartz cuvette with a pathlength of 10 mm.

2.5 Molecular Dynamic (MD) Simulation Methods

The experimental structure for each peptide variation was assigned parameters using Open Babel[9] and the CHARMM General Force Field (CGenFF)[10] with most penalties below 10 and a maximum penalty of 17.9. The azide group parameters were poorly parametrised by CGenFF and instead taken from literature.[11] The systems were built and run using GROMACS 2021.5.[12] The aqueous boxes were 5.5 x 4 x 4.5 nm^3 in volume, containing 100 mM of sodium chloride and ~3,000 TIP3 water molecules. Energy minimization using the steepest descent algorithm was performed twice for 2,500 steps each time with a 0.1 nm step size. Equilibrations with unrestrained solvent and increasing time steps were performed in the isothermal-isobaric ensemble (constant number of particles, pressure, and temperature, NPT) for 200 ns total. In total four systems were prepared in the deprotonated state: i) standard peptide (**S9**); ii) demethylated at the *N*-terminal position (**S15**); iii) demethylated at the *C*-terminal position (**S14**); iv) both *N*- and *C*-demethylated. All unrestrained production simulations were in the NPT ensemble and performed for 1 µs. Equations of motion were integrated through the Verlet leapfrog algorithm with a 2 fs time step, and bonds connected to hydrogens were constrained using the LINCS algorithm. The cutoff distance was 1.2 nm for the short-range neighbour list and van der Waal's interactions with a smooth switching function from 1.0 nm. The particle mesh Ewald method was applied for long-range electrostatic interactions with a 1.2 nm real space cutoff.[13] The Nosé-Hoover thermostat[14,15] and Parrinello-Rahman barostat[16] were used to maintain the temperature and pressure at 258 K and 1 bar (semiisotropically) respectively. Simulations were performed in-house as part of the CREATE high performance computing cluster at King's College London[17] using a Nvidia GeForce RTX 3060 Ti GPU and 16 CPUs. Analysis of the systems were performed using Python 3.7, MDAnalysis,[18,19] GROMACS 2021.5[12] and PyMol.[20]

We examined four sets of carbon atoms along the backbone and compared their dihedrals across the four systems. The colours for each set of dihedrals are shown in the figure below.

A.

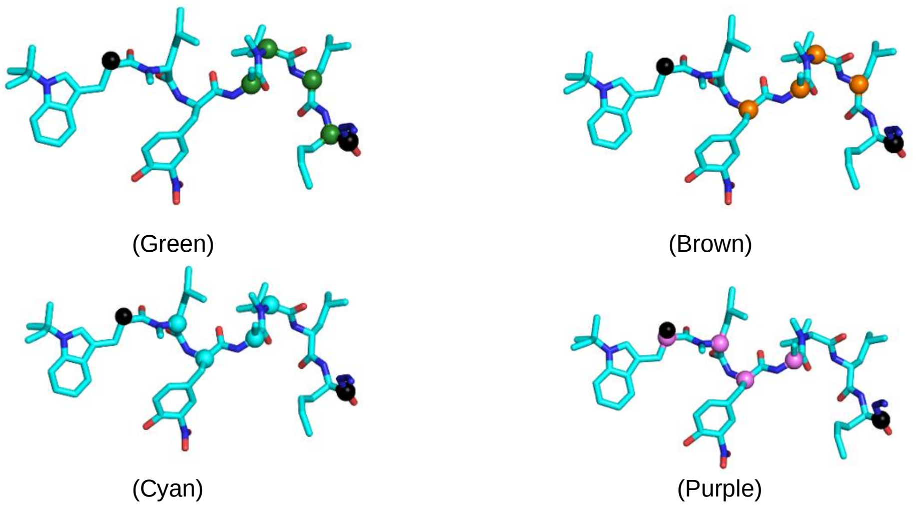

B.

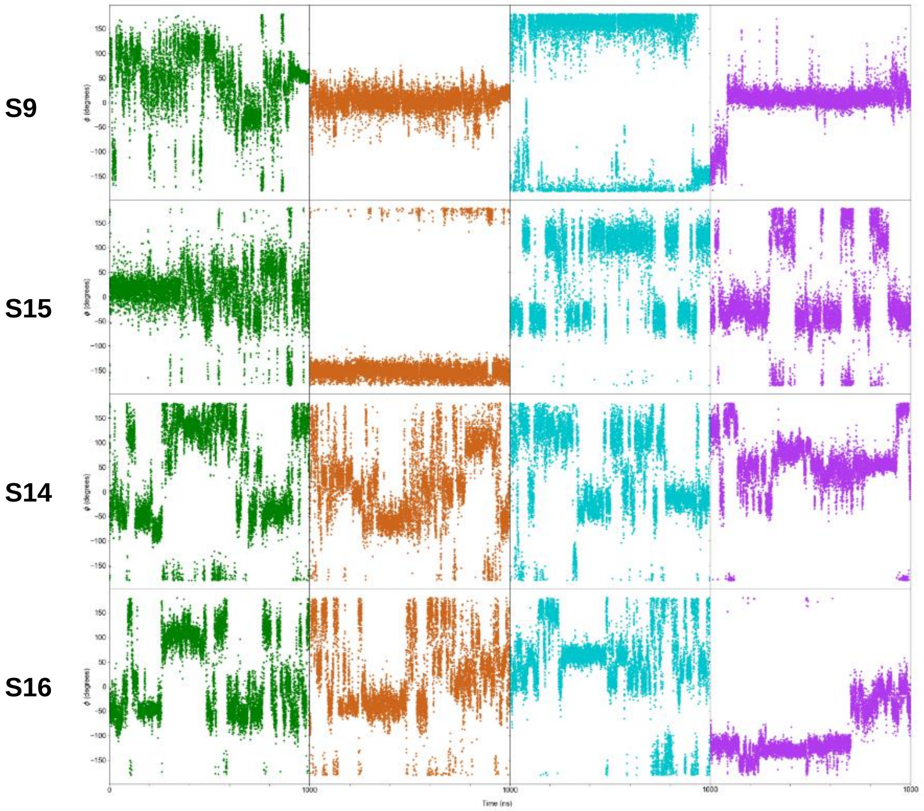

Figure S5. Peptide backbone dihedrals analysis. A. The set of four carbon dihedrals that were used to compare the dihedrals for each system. The colours exemplified by Linear peptide **S9**, corresponding to the colour of the panel in **B**. The cyclising atoms are shown in black to show the molecular orientation with more clarity. **B.** Top set: Dihedrals for the **S9** peptide. Second set: Dihedrals for the **S15** peptide. Third set: Dihedrals for the **S14** peptide. Fourth set: Dihedrals for the system with both methyl groups removed. The **S9** and **S15** peptides are the two peptides that were tested that cyclised at a faster rate and they both show stable values (baring occasional inversions in magnitude for the case of the **S15** peptide) for the second set of dihedrals. Despite the differences (~0° for S9 and ~-180° for S15), the stability of the dihedral is more important than the value. Having a stable dihedral means the plane that these atoms lie in is approximately fixed, which will be favourable for encouraging the key N2-O4

hydrogen bonding interaction. The **S9** peptide in particular has three stable sets of dihedrals, again baring occasional inversions in magnitude which correspond to a twisting of the backbone. These atoms cover the range of the N2 and O4 atoms which were shown to be a prominent and dominant H-bond for these systems.

A.

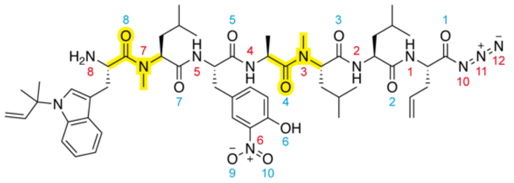

S9

B.

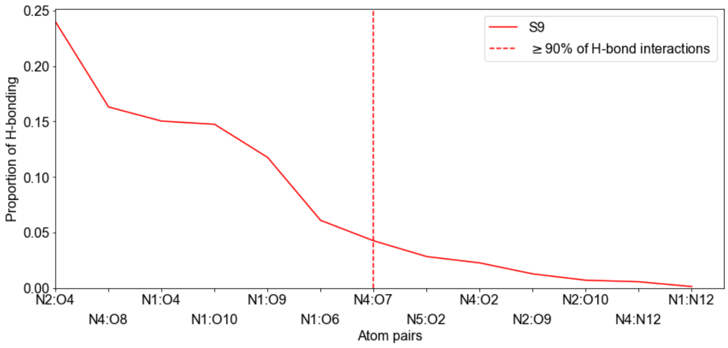

C.

Donor Name	Acceptor Name	Frequency	Proportion of HBs
N2	O4	341	0.2595
N4	O8	129	0.0982
N1	O4	215	0.1636
N1	O10	209	0.1591
N1	O9	165	0.1256
N1	O6	87	0.0662
N4	O7	60	0.0457
N5	O2	39	0.0297
N4	O2	31	0.0236
N2	O9	18	0.0137
N2	O10	10	0.0076
N4	N12	8	0.0061
N1	N12	2	0.0015

Figure S6. Molecular dynamics simulation of peptide S9. A. Atom number labelled in the molecular dynamics simulation. Nitrogen shown in red, oxygen shown in blue. **B.** Proportion of the H-bond interactions for the **S9** peptide (with the labels corresponding to A). All H-bonds have an upper limit of 3.3 Å. The **S9** peptide had the key N2-O4 H-bond as its dominant reaction, but also showed the most diversity in dominant interactions, with 6 distinct interactions constituting at least 90% of all H-bond interactions. **C.** Table of data for B. The frequency does not account for the length of the H-bond interaction, but consideration of Fig. 5B shows that the **S9** peptide had the longest lasting H-bond interactions.

A.

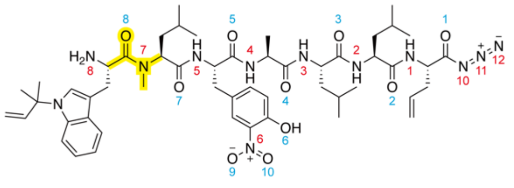

B.

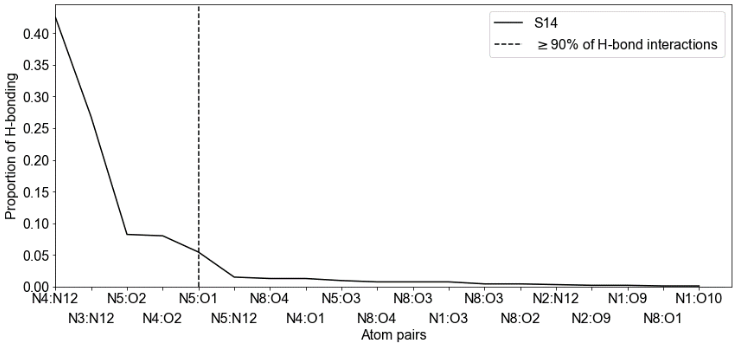

C.

Donor Name	Acceptor Name	Frequency	Proportion of HBs
N4	N12	282	0.4228
N3	N12	178	0.2669
N5	O2	55	0.0825
N4	O2	54	0.0810
N5	O1	36	0.0540
N5	N12	10	0.0150
N8	O4	9	0.0135
N4	O1	9	0.0135
N5	O3	6	0.0090
N8	O4	5	0.0075
N8	O3	5	0.0075
N1	O3	5	0.0075
N8	O3	3	0.0045
N8	O2	3	0.0045
N2	N12	2	0.0030
N2	O9	2	0.0030
N1	O9	1	0.0015
N8	O1	1	0.0015
N1	O10	1	0.0015

Figure S7. Molecular dynamics simulation of peptide S14. A. Atom number labelled in the molecular dynamics simulation. Nitrogen shown in red, oxygen shown in blue. **B.** Proportion of the H-bond interactions for the **S14** peptide (with the labels corresponding to A). All H-bonds have an upper limit of 3.3 Å. Four distinct interactions make up at least 90% of all H-bond interactions for this peptide, but nearly 70% of all interactions involve the terminal azide. For the **S9** peptide the terminal azide rarely was involved in H-bond interactions, suggesting the removal the C-terminal methyl group allows the azide to access the central part of the peptide where the N3 and N4 nitrogen atoms are located. **C.** Table of data for B. The frequency does not account for the length of the H-bond interaction.

A.

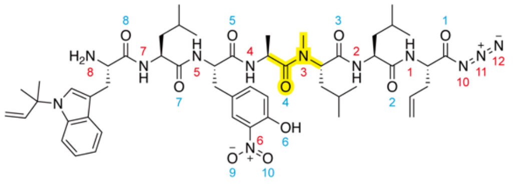

S15

B.

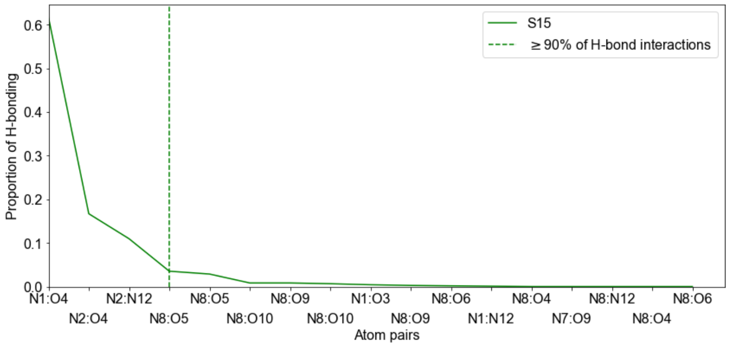

C.

Donor Name	Acceptor Name	Frequency	Proportion of HBs
N1	O4	393	0.6112
N2	O4	107	0.1664
N2	N12	71	0.1104
N8	O5	23	0.0358
N8	O5	19	0.0295
N8	O10	6	0.0093
N8	O9	6	0.0093
N8	O10	5	0.0078
N1	O3	3	0.0047
N8	O9	2	0.0031
N8	O6	2	0.0031
N1	N12	1	0.0016
N8	O4	1	0.0016
N7	O9	1	0.0016
N8	N12	1	0.0016
N8	O4	1	0.0016
N8	O6	1	0.0016

Figure S8. Molecular dynamics simulation of peptide S15. A. Atom number labelled in the molecular dynamics simulation. Nitrogen shown in red, oxygen shown in blue. **B.** Proportion of the H-bond interactions for the **S15** peptide (with the labels corresponding to A). All H-bonds have an upper limit of 3.3 Å. Just three distinct interactions make up at least 90% of all H-bond interactions for this peptide, but 61% of these interactions are between the N1-O4 atoms, and 77% involve the O4 atom. This is a stark contrast to the **S14** peptide where the N1, N2, and N4 atoms played almost no part in the H-bond interactions. **C.** Data shown in the table below. The frequency does not account for the length of the H-bond interaction.

A.

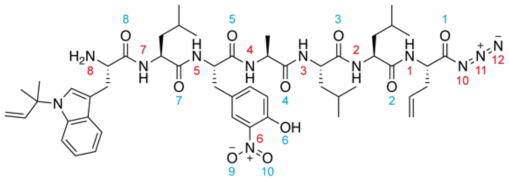

S16

B.

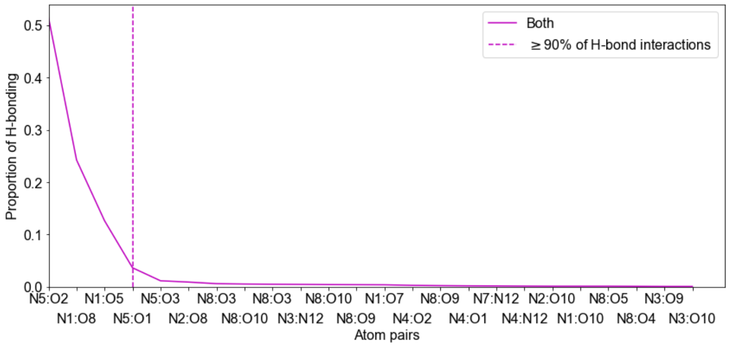

C.

Donor Name	Acceptor Name	Frequency	Proportion of HBs
N5	O2	1169	0.5107
N1	O8	551	0.2407
N1	O5	288	0.1258
N5	O1	83	0.0363
N5	O3	27	0.0118
N2	O8	21	0.0092
N8	O3	15	0.0066
N8	O10	14	0.0061
N8	O3	12	0.0052
N3	N12	11	0.0048
N8	O10	10	0.0044
N8	O9	10	0.0044
N1	O7	9	0.0039
N4	O2	7	0.0031
N8	O9	6	0.0026
N4	O1	5	0.0022
N7	N12	4	0.0017
N4	N12	4	0.0017
N2	O10	4	0.0017
N1	O10	4	0.0017
N8	O5	3	0.0013
N8	O4	3	0.0013
N3	O9	3	0.0013
N3	O10	3	0.0013
N7	O2	2	0.0009
N5	N12	2	0.0009
N8	O1	2	0.0009
N8	O1	2	0.0009
N3	O2	2	0.0009
N7	O3	1	0.0004
N2	O9	1	0.0004
N1	O9	1	0.0004
N1	O4	1	0.0004

N1	N12	1	0.0004
N8	N12	1	0.0004
N3	O8	1	0.0004
N4	O8	1	0.0004
N8	O2	1	0.0004
N8	N12	1	0.0004
N8	O2	1	0.0004
N8	O4	1	0.0004
N1	O3	1	0.0004

Figure S9. Molecular dynamics simulation of peptide S16. A. Atom number labelled in the molecular dynamics simulation. Nitrogen shown in red, oxygen shown in blue. **B.** Proportion of the H-bond interactions for the peptide with both methyl groups removed (with the labels corresponding to figure A). All H-bonds have an upper limit of 3.3 Å. Four distinct interactions make up at least 90% of H-bond interactions. Interestingly, this peptide shows the greatest diversity in its H-bond interactions with over 40 distinct interactions, but only five with more than 1% of the total interactions. With both methyl groups removed, there is less shielding at either terminus, but we can see from Fig. 5B that these interactions are short lived. **C.** Data shown in the table below. The frequency does not account for the length of the H-bond interaction.

2.6 Antibacterial Assays

20% glycerol stocks of *Escherichia coli* K12 (NCTC 10538), *Staphylococcus aureus* NCTC 12973, *Bacillus subtilis* NCTC 06276, and *Enterococcus faecium* NCTC 7171 stored at -80 °C were used to streak nutrient agar plates (brain heart infusion (BHI) agar for *E. faecium*). After incubation for 24 h at 37 °C, 1.5 mL Müller Hinton Broth II (MHBII) (BHI broth for *E. faecium*) in a 15 mL falcon was inoculated with either *E. coli*, *S. aureus*, *B. subtilis*, or *E. faecium*, (3 separate colonies were picked for each making 3 cultures for each and a total of 12 cultures) and incubated at 37 °C with shaking at 180 rpm for 16 h. The resulting suspensions were diluted 100 x (15 µL in 1.5 mL) in MHBII or BHI broth and the resultant suspensions incubated for a further 3 h at 37 °C with shaking. 15 x 96 well plates were primed with concentration gradients of the analytes and the controls (carbenicillin and vancomycin) in MHBII or BHI broth. The cells were diluted to an OD_{600} of 0.01. The plates were then inoculated with 50 µL per well of bacterial culture in MHBII or BHI broth to make 100 µL per well (final OD_{600} of 5×10^{-3}). Plates were then incubated at 37 °C for 24 h. OD_{600} was measured on a BMG Labtech CLARIOstar plate reader. The MIC is the first value at which no growth is seen. All data is derived from 3 biological replicates.

Table S1. Antimycobacterial activities of peptides **25c**, **26c** & **29c**

	**MIC (µM)**			
	B. subtilis	***E. coli***	***E. faecium***	***S. aureus***
Gramicidin S (**29c**)	-	-	2.5	3.1
Tyrocidine A (**25c**)	3.1	-	-	6.3
Carbenicillin	1.6	25	-	25
Vancomycin	-	-	0.5	-
R-cyclopurpuracin (**26c**)	-	>1000	-	>1000

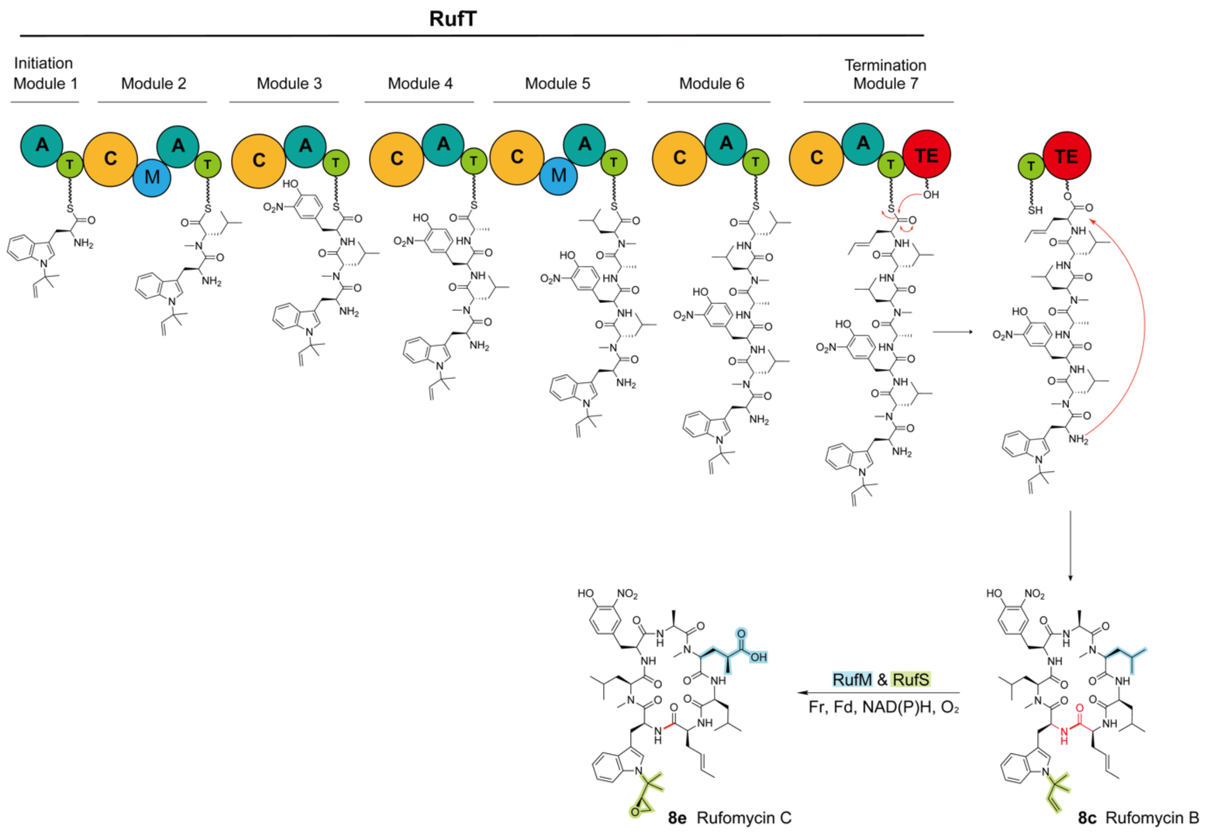

Figure S10. Biosynthesis of rufomycins. Synthesis of rufomycin B by NRPS RufT. This cyclic scaffold was further modified by the tailoring enzyme RufM (sky blue) and RufS (light green).[21,22,23]

8a

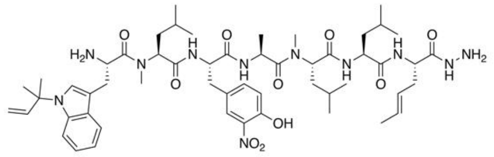

9a

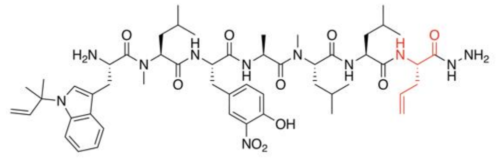

10a$_{L\text{-1-Me-Trp}}$

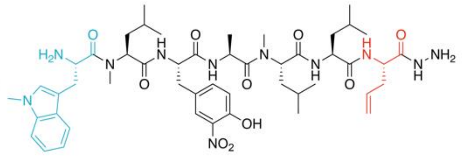

10b$_{D\text{-1-Me-Trp}}$

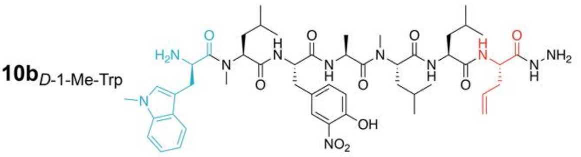

11a

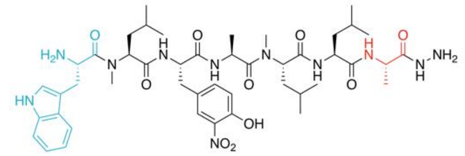

12a

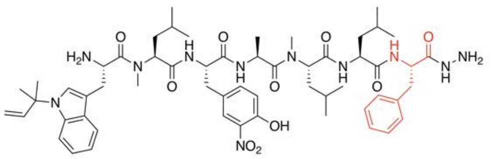

13a

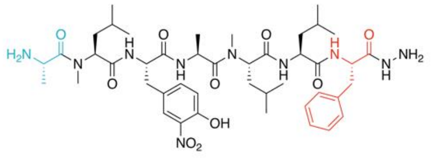

14a

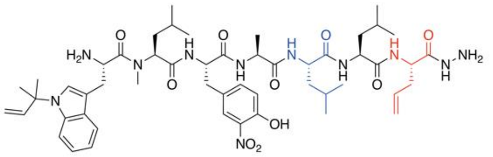

15a

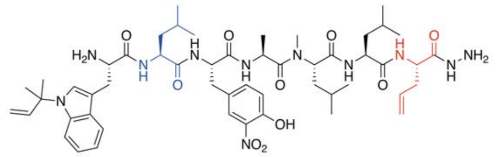

16a

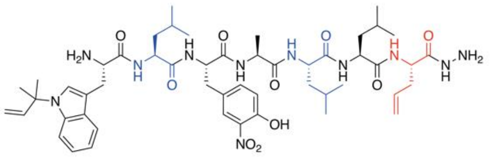

17a

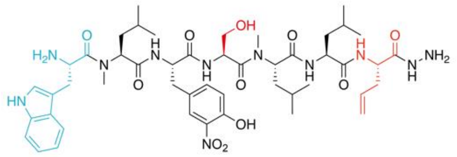

18a

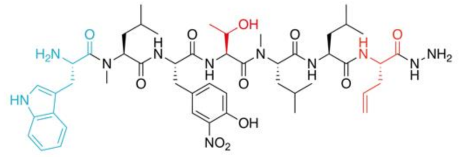

Figure S11. Linear peptide library of rufomycin derivatives prepared at semi-prep scale. Sequence of synthetic *C*-terminal hydrazide peptides, peptide **8a** is the natural sequence of rufomycin. The difference inside chains compared to native rufomycin are shown in colour. *C*-terminal amino acid shown in red, *N*-terminal amino acid shown in cyan and the other residues shown in blue.

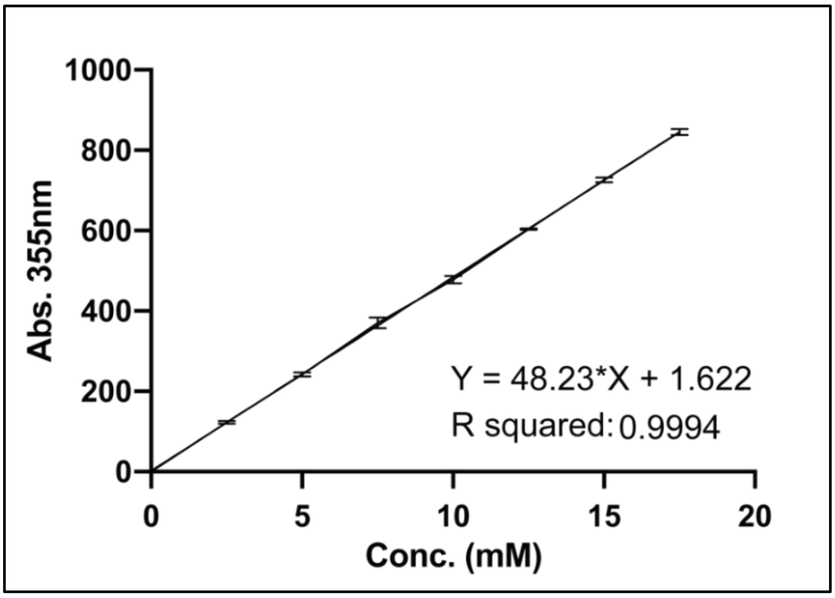

Figure S12. Fmoc-3-nitro-*L*-tyrosine calibration curve. Fmoc-3-nitro-*L*-tyrosine stock solution (20 mM) was prepared in triplicate in a 3 mL volumetric flask. 26.9 mg of Fmoc-3-nitro-*L*-tyrosine were dissolved in acetonitrile, few drops DMF was required to solubilize compound. HPLC samples were serially diluted to give samples at six different concentrations, then each sample (1 µL) was injected into HPLC (analyzed using Gradient 24). Absorbance was monitored at 355 nm, area under the curve was recorded and a calibration curve was plotted, the R squared value is 0.9994.

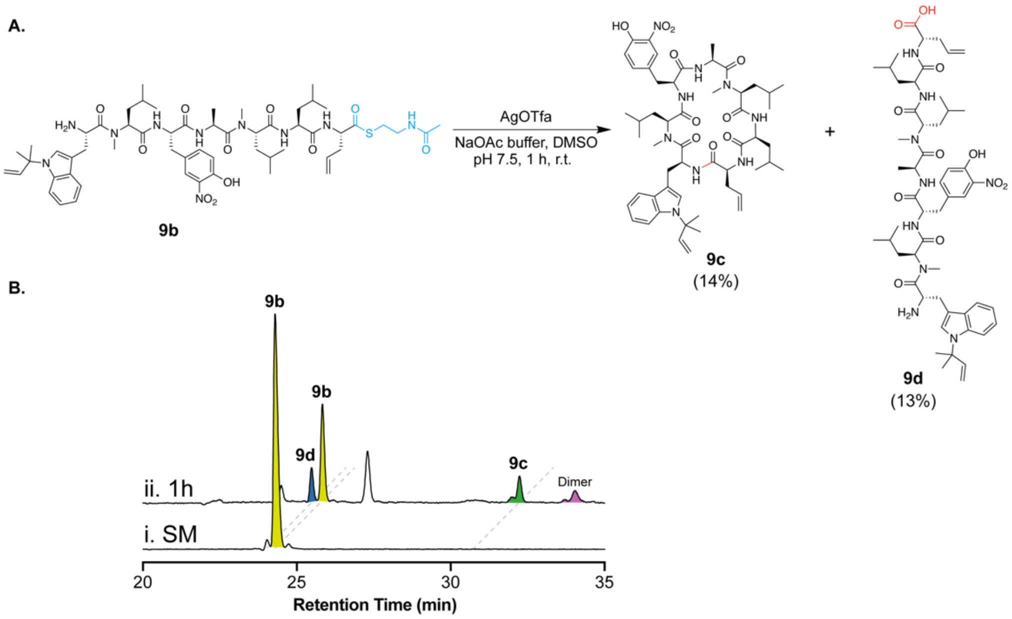

Figure S13. Silver-assisted SNAC peptide cyclization. A. Reaction scheme. B. HPLC analysis (355 nm) of crude reaction mixture. i. purified SNAC peptide **9b**. ii. reaction crude mixture after 1h. Conversions in A. are calculated from HPLC.

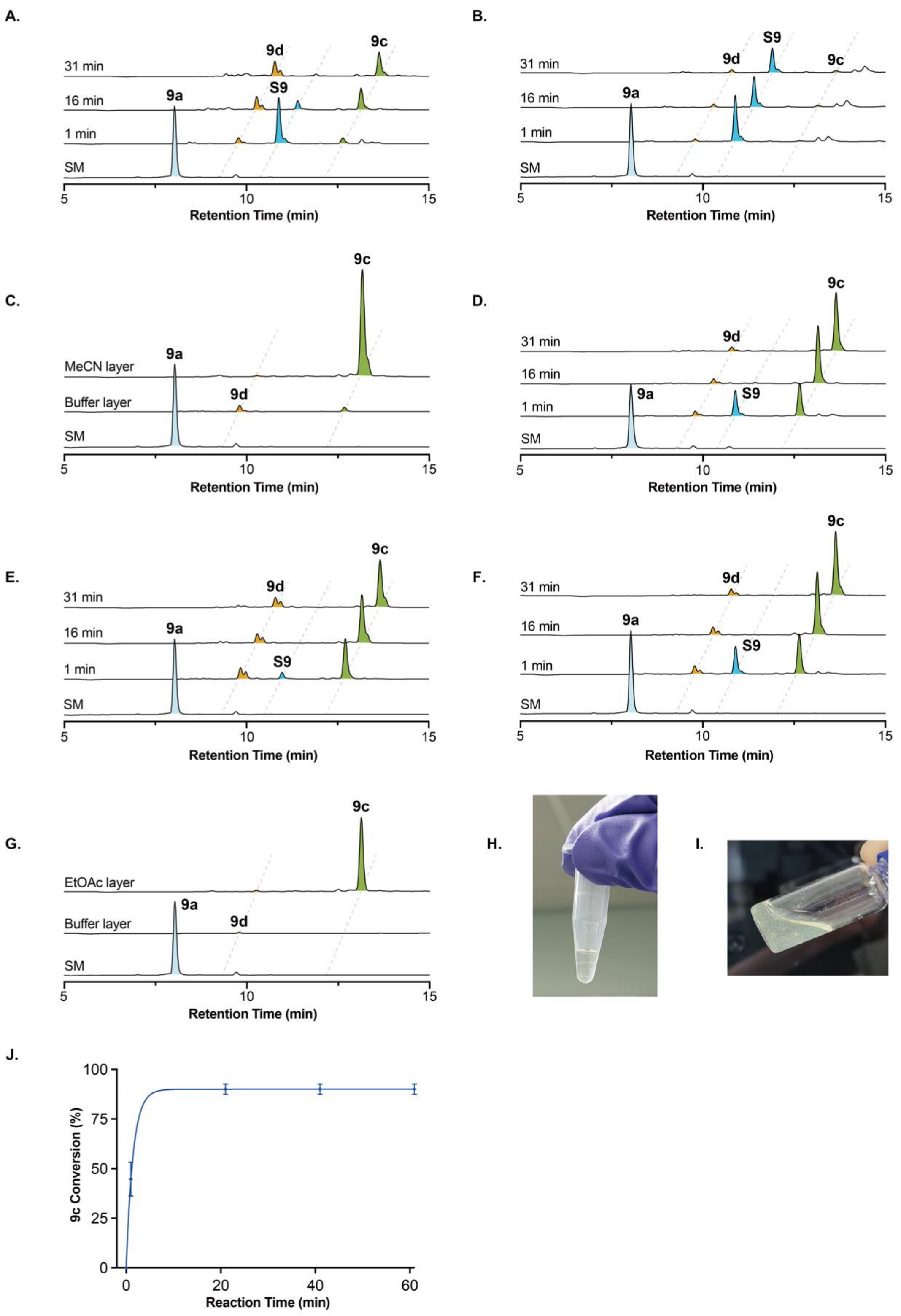

Figure S14. HPLC analysis of chemical cyclization of 9a. After raising the pH to 7.0 ~ 7.5, the reaction mixture was diluted with various solvents to monitor the reaction. Chromatogram SM is the purified acyl-hydrazide peptide **9a** before oxidation. Timing started after adjusting the pH with sodium bicarbonate. **A.** Reaction buffer pH ~7.5. **B.** Water only, reducing Gdm concentration resulted in the acyl azide peptide crashing out of the buffer (see **I.**). **C.** MeCN (2 vol) was immiscible with concentrated buffer solution resulting in biphasic solution (see **H.**). **D.** 50% MeCN + 0.1% TFA. **E.** 50% MeCN no TFA. **F.** 50% MeCN no TFA (however, pH only adjusted to 7). **G.** Semi-preparative scale procedure. Adding EtOAc into the reaction mixture. **J.** Cyclization of peptide **9a** was carried out in triplicate to ensure the reliability of the results. HPLC analysis (355 nm) using HPLC Gradient 3.

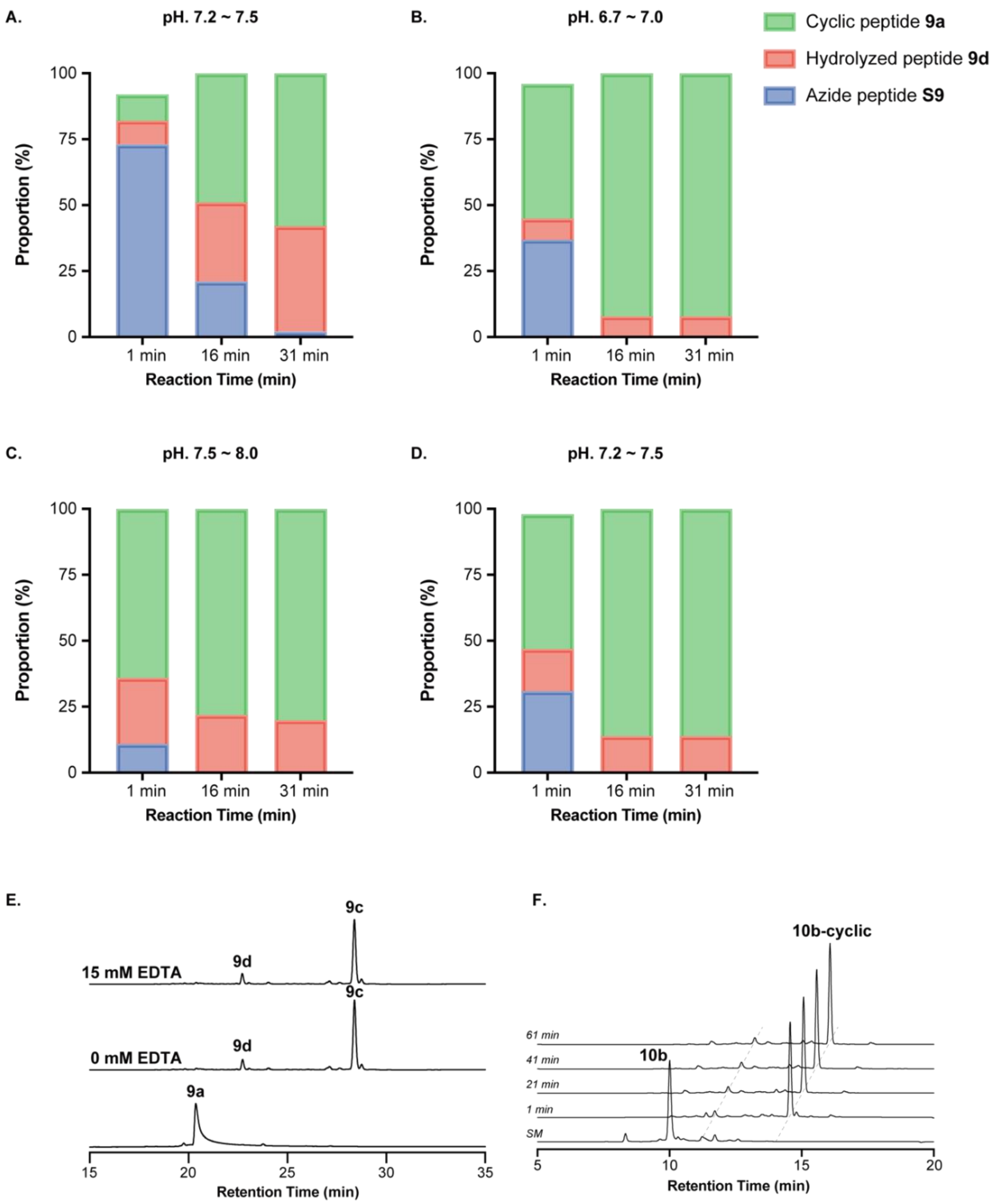

Figure S15. Chemical cyclization product distribution as a function of reaction conditions and stereochemistry. Graphs **A. B. C.** and **D.** corresponding to HPLC of **Supplementary Figure 14A. 14D. 14E.** and **14F.** which analyze product distribution as a function of pH. Buffer pH following addition of $NaHCO_3$ at the cyclization step is indicated in the title of each graph. **E**. Effect of EDTA concentration on chemical cyclization monitored by HPLC (355 nm) using Gradient 2. **F.** Effect of *N*-terminal stereochemistry on chemical cyclization of **10b** with *N*-terminal *N*-Me-*D*-Trp monitored by HPLC (355 nm) using Gradient 7. HPLC analysis (355 nm) of chemical cyclization. All reactions were carried out on analytical scale (1 mL reaction buffer).

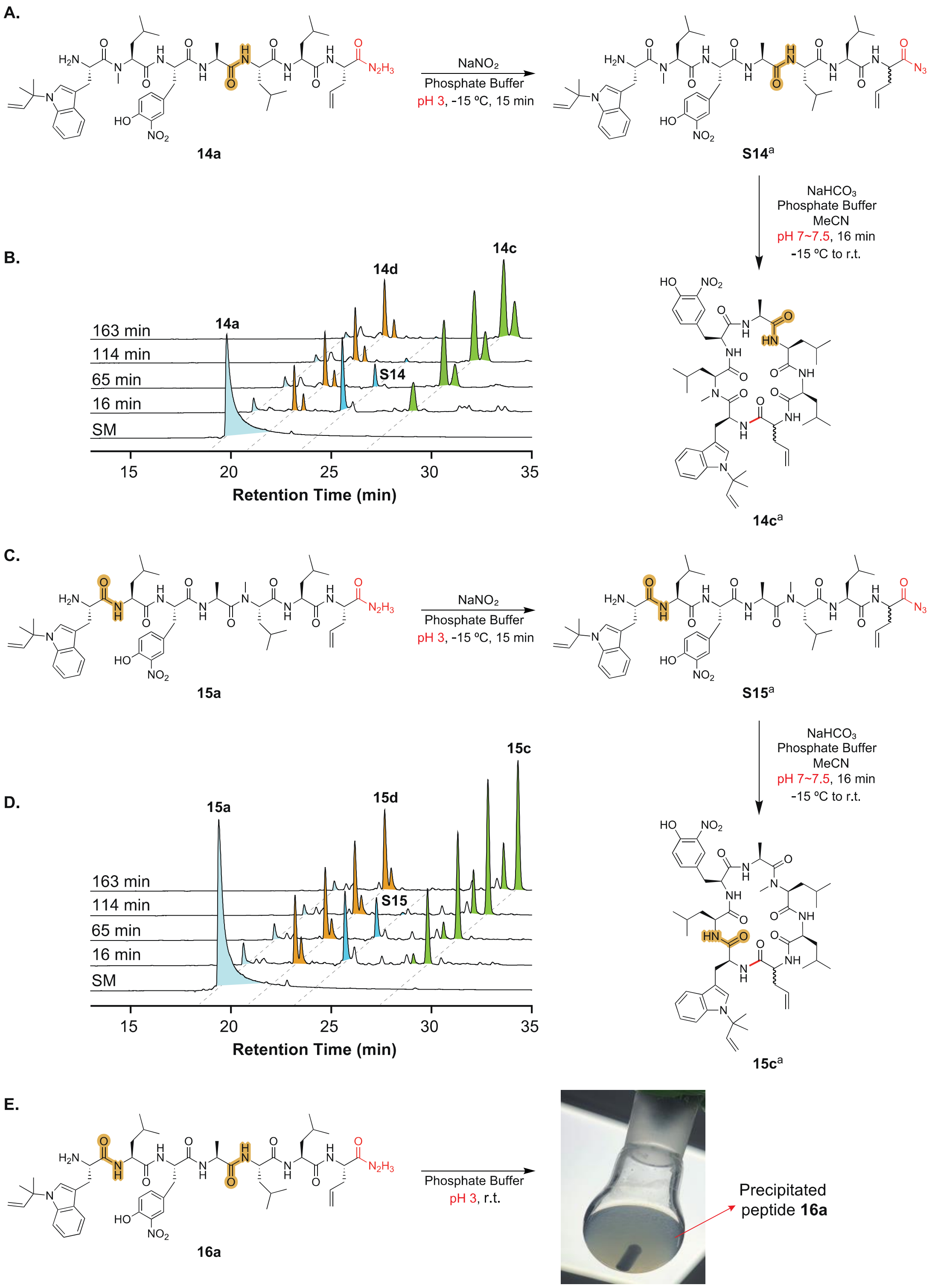

Figure S16. Effect of backbone *N*-methylation on chemical cyclization. A. Reaction scheme of **14a** cyclization. **B.** HPLC analysis (355 nm). Chromatogram SM is the purified acyl-hydrazide peptide **14a** before oxidation. Peaks labelled with the same colour have the same mass. **C.** Reaction scheme of **15a** cyclization. **D.** HPLC analysis (355 nm). Chromatogram SM is the purified acyl-hydrazide peptide **15a** before oxidation. Peaks labelled same colour have the same mass. HPLC analysis using Gradient 2. **E.** Acyl-azide peptide **16a** in which both backbone *N*-methyl groups have been removed precipitated in phosphate buffer, even in the presence of DMF or MeCN.

A.

❖ Reaction components in chemical cyclisation

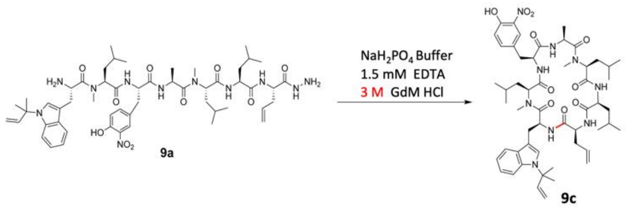

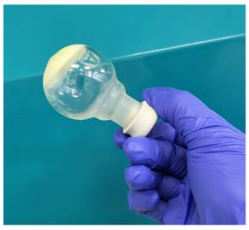

Frozen mixture

B.

❖ Reaction components in chemical cyclisation

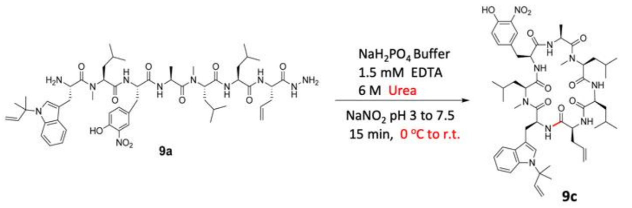

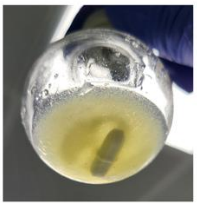

Peptide-hydrazide soluble

But after oxidation

Peptide-azide insoluble

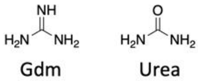

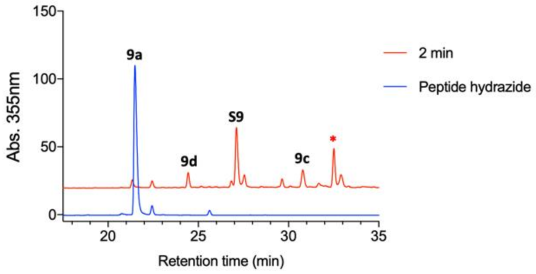

Figure S17. **Investigation of the effects of reaction components on cyclisation reaction outcome.** **A**. Investigation of guanidinium chloride (Gdm) concentration. Reducing the concentration of GdmCl to 3 M resulting frozen reaction mixture at reaction temperature -15 °C (picture). **B**. Substitution of guanidinium chloride with urea. Replacing GdmCl with urea resulted in poor solubility of acyl-azide peptide intermediate (picture). The poor solubility leads to dimer formation (labelled with a star on HPLC trace).

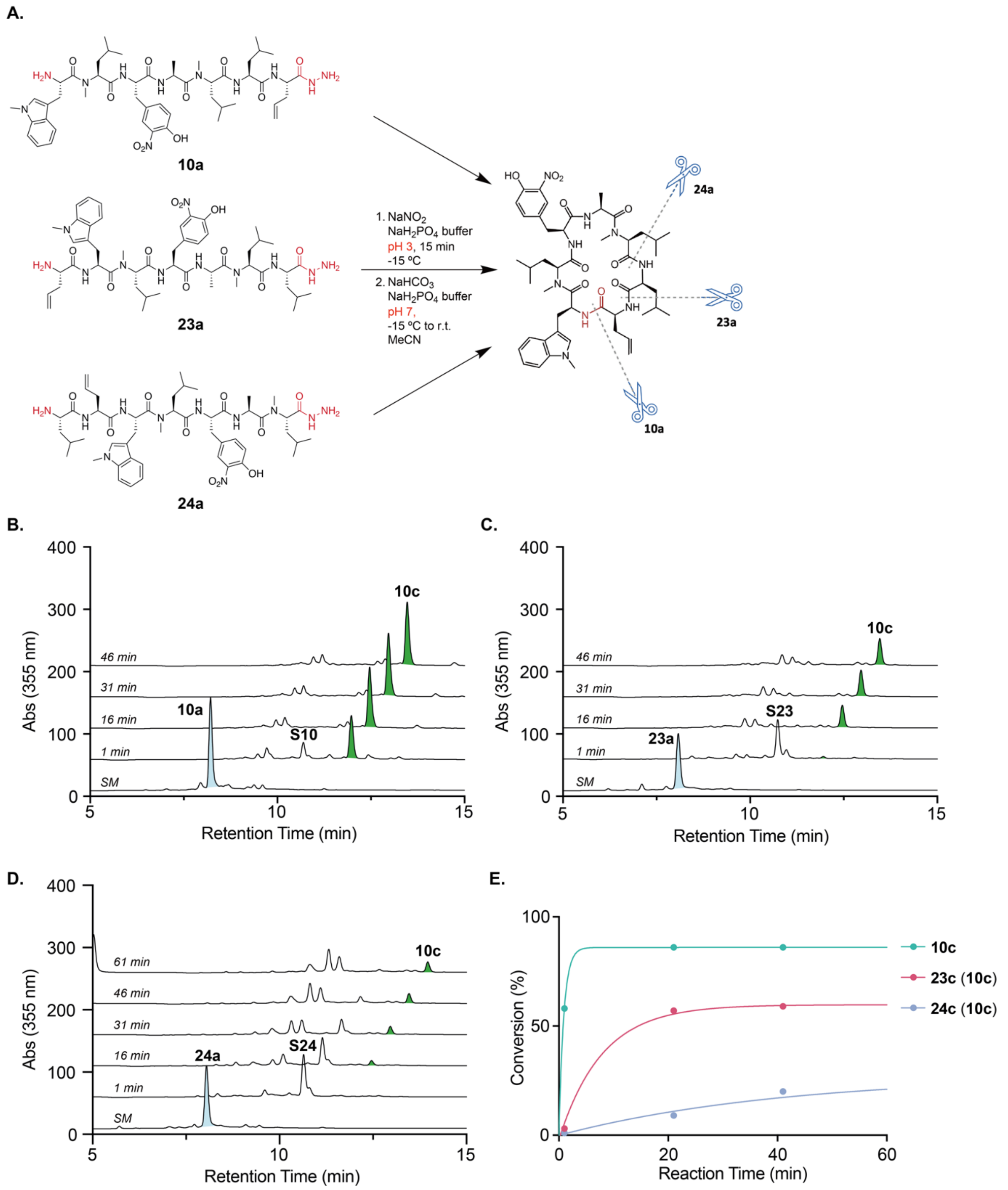

Figure S18. Macrocyclization to generate 10c starting from different points of cyclization. A. Reaction scheme indicating linear precursors and resulting points of cyclisation. Peptide **10a** represents the sequence of the biosynthetic linear precursor. **B. C. D.** HPLC analysis (355 nm) of each cyclisation reaction using Gradient 3. **E.** Relative cyclization rate for peptide **10a**, **23a** and **24a**. Data was fitted using a non-linear regression. Non-native linear peptide **23a** and **24a** resulting in a slower reaction rate and reduced conversion.

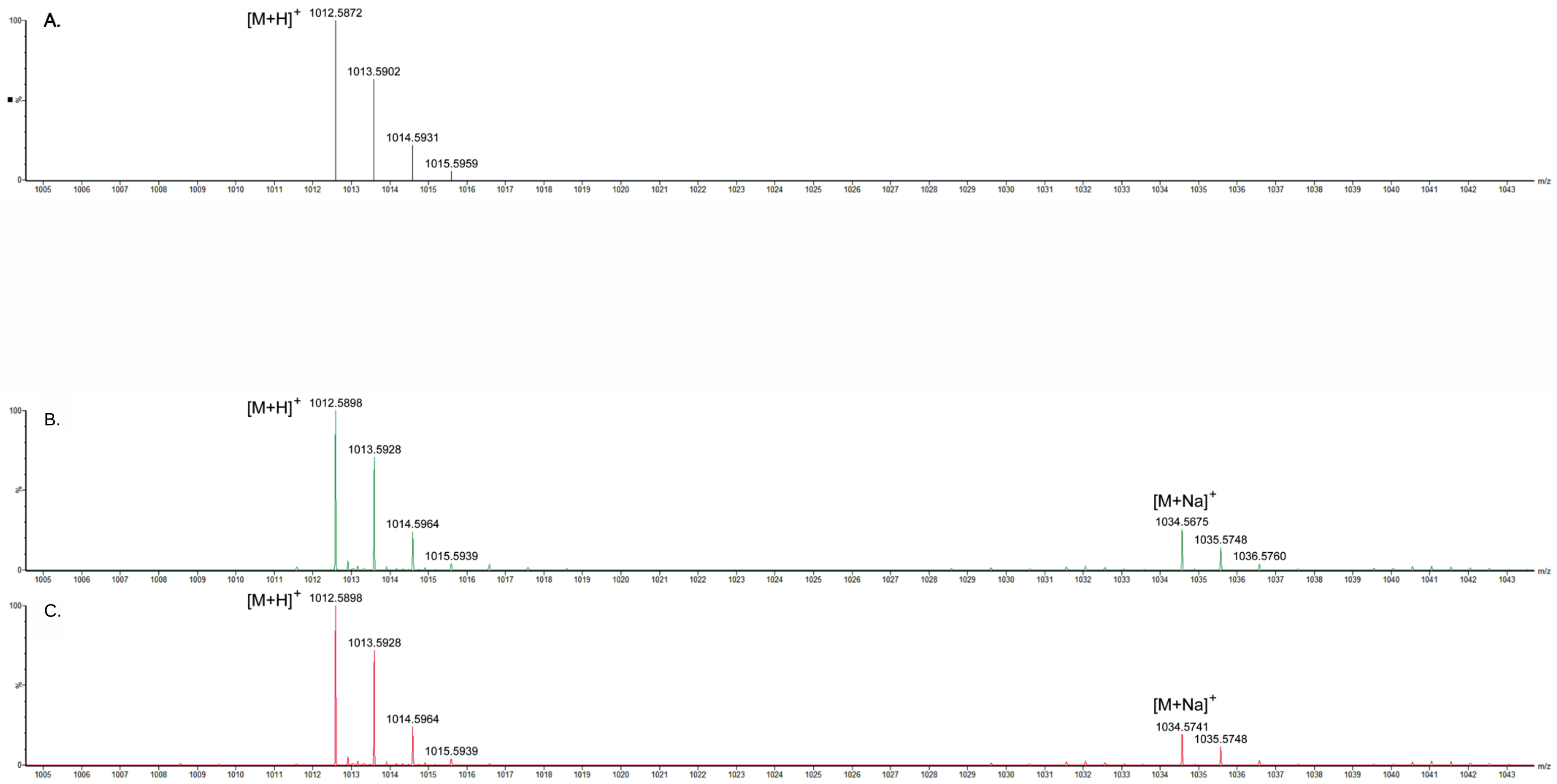

Figure S19. HRMS analysis of cyclic peptide 8c rufomycin B. A. Simulated mass spectrum of **8c** rufomycin B. **B.** Mass spectrum of enzymatically cyclized **8c**. **B.** Mass Spectrum of chemically cyclized **8c**. **C.** Mass spectrum of **8c** rufomycin B isolated from *Streptomyces atratus* as previously described. [23]

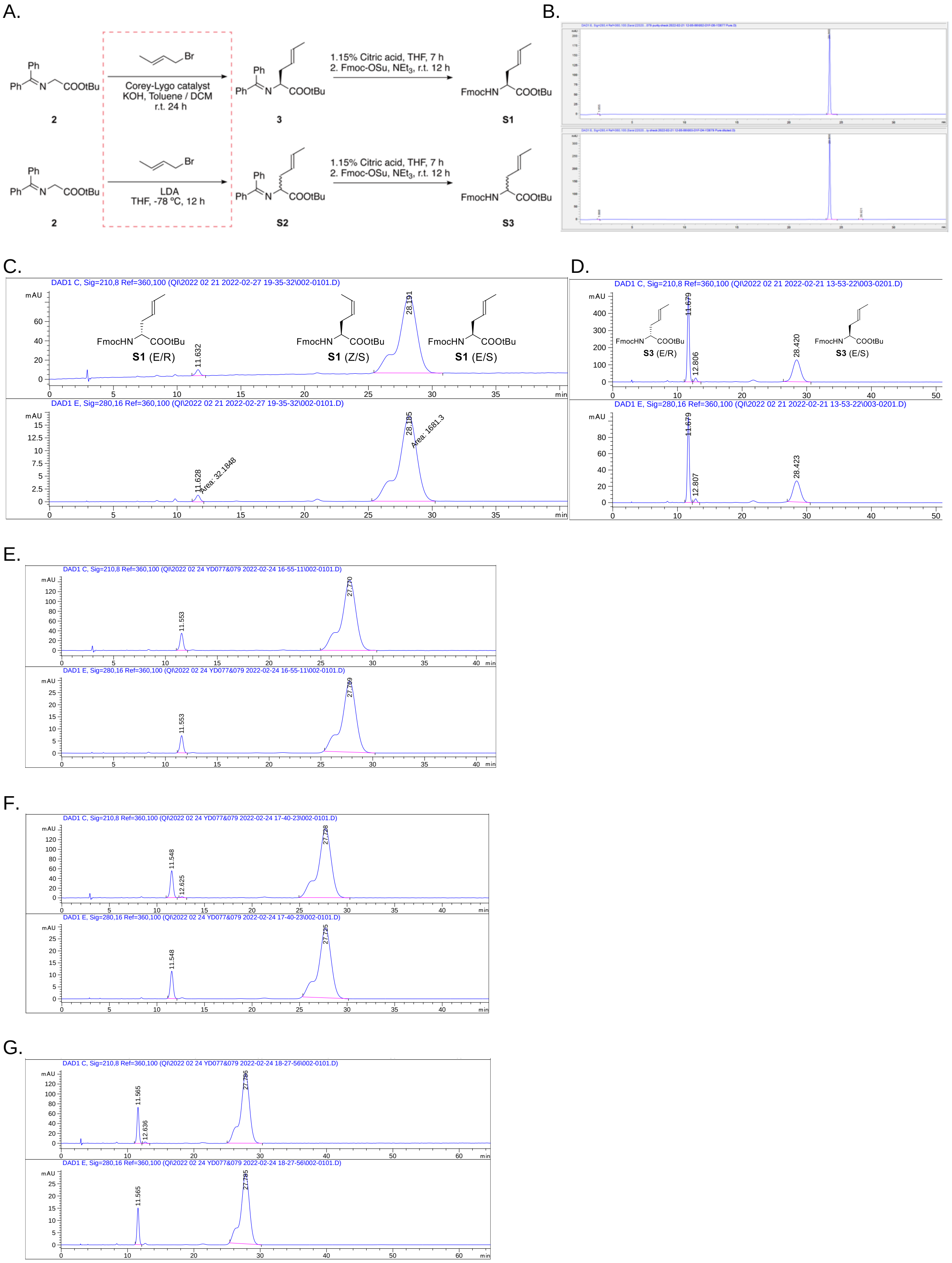

Figure S20. Chiral HPLC analysis of synthesized amino acid S1 and S3. A. Reaction scheme of Fmoc protected amino acid synthesis. **S2** synthesis without adding chiral auxiliary. **B.** Reverse phase HPLC chromatogram of purified **S1**(top) and **S3** (bottom) monitored at 280 nm. **C.** Chiral normal phase HPLC chromatogram of **S1** (monitored at 210 nm (top trace) and 280 nm (bottom trace)). **D.** Chiral normal phase HPLC chromatogram of **S3** (monitored at 210 nm (top trace) and 280 nm (bottom trace)). **E.** Sample co-injection for peak confirmation. (1.2 mM of **S1** and 0.12 mM of **S3**). **F.** Sample co-injection for peak confirmation. (1.2 mM of **S1** and 0.32 mM of **S3**). **G.** Sample co-injection of **S1** and **S3** for peak confirmation. (1.2 mM of **S1** and 0.45 mM of **S3**). (see also section 2.1.3)

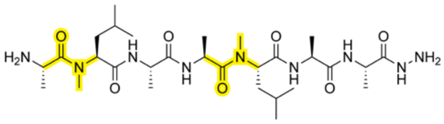

19a

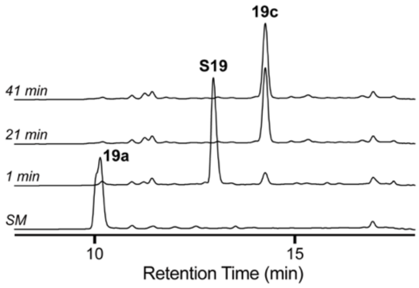

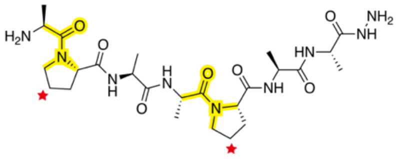

20a

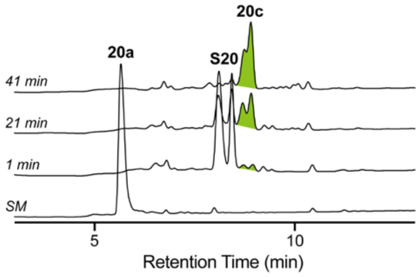

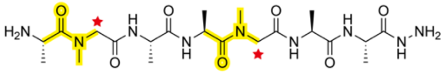

21a

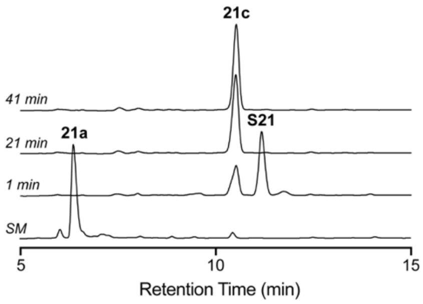

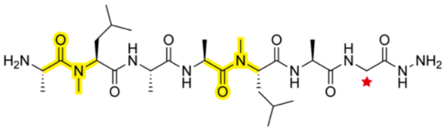

22a

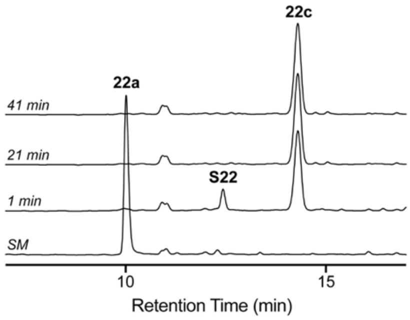

Figure S21. HPLC analysis (214 nm) of peptide cyclization of peptides **19a**, **20a**, **21a** and **22a**. HPLC analysis of peptide **19a 20a 22a** analytical scale (reaction volume 1 mL) cyclization using HPLC Gradient 4, for peptide **21a** using HPLC Gradient 5. **S19-22** indicates peptide azide formation, **19-22c** indicates cyclic peptide. **20c** appears as two peaks, likely resulting from *cis*–*trans* proline isomers.

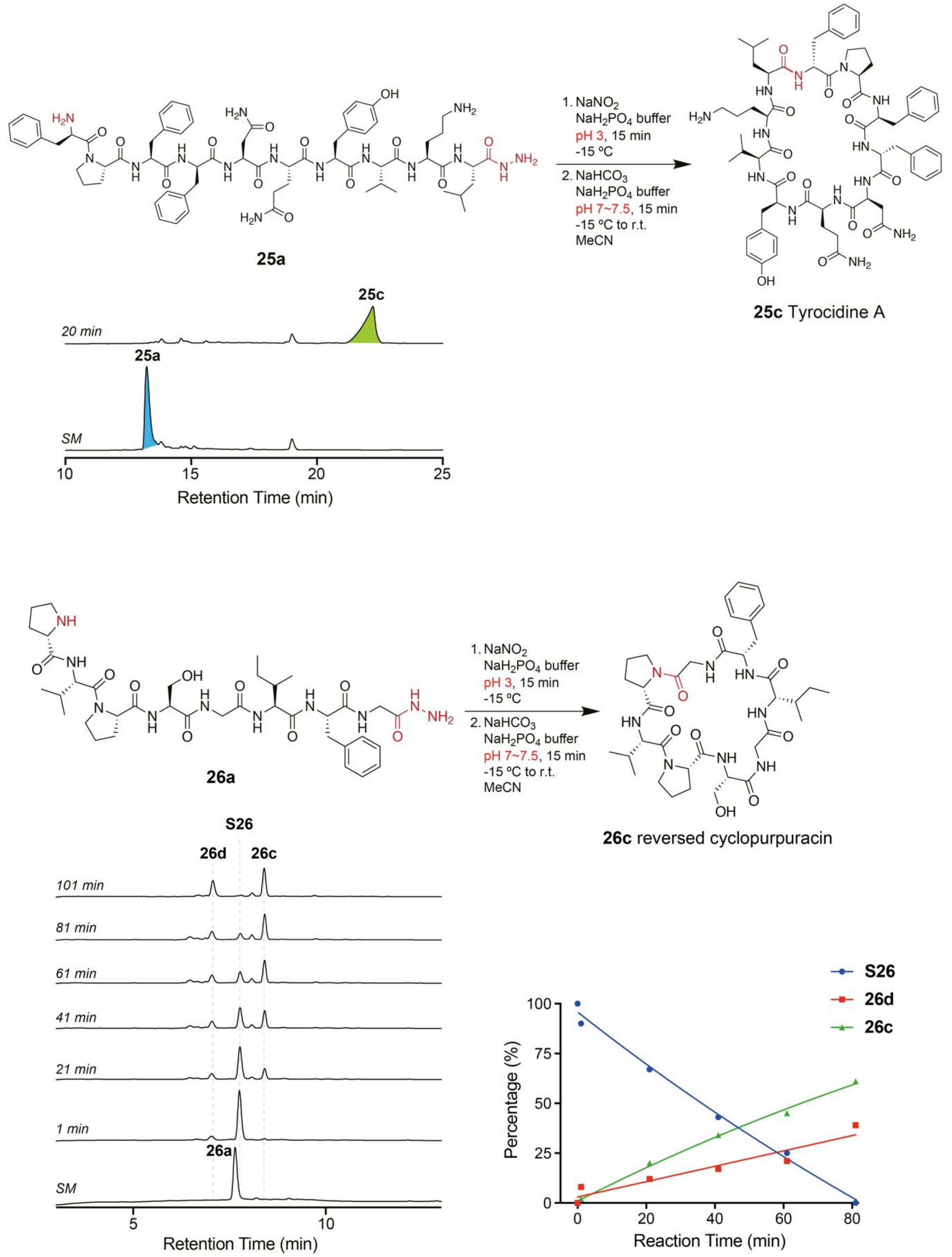

Figure S22. HPLC analysis (214 nm) of peptide cyclization of **25a** and **26a** over time to give tyrocidine A and R-cyclopurpuracin respectively. HPLC analysis of peptide **25a** analytical scale cyclization using HPLC Gradient 6. For peptide **26a** HPLC Gradient 7 was used. **26d** is hydrolysed linear peptide. **S26** indicates intermediate acyl azide formation. (Bottom right) Graph indicating relative rates of consumption of intermediate **S26** and formation of hydrolysed product **26d**, cyclised product **26c** (% conversion calculated from HPLC).

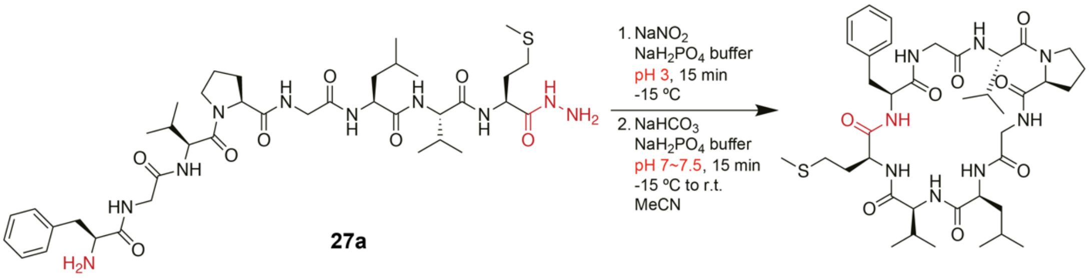

27c Planktocyclin

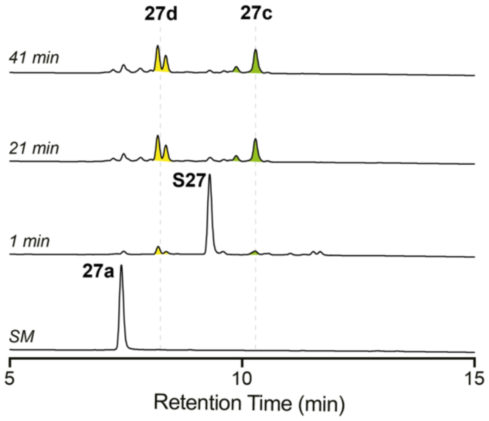

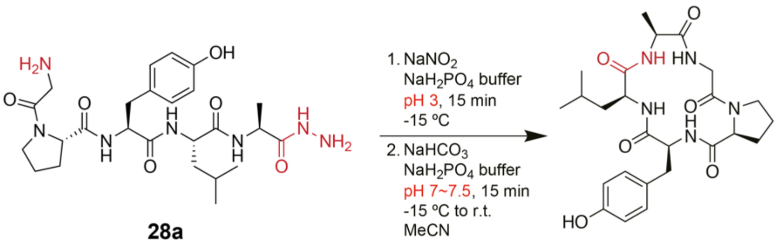

28c Pseudostellarin A

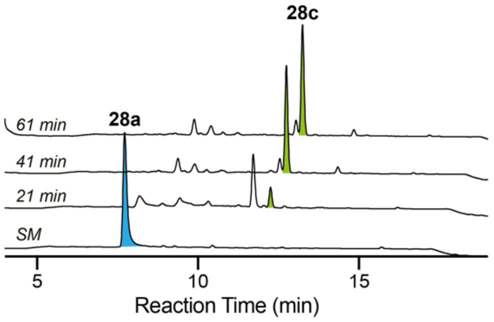

Figure S23. HPLC analysis (214 nm) of peptide cyclization of **27a** and **28a**. HPLC analysis of peptide **27a** and **28a** analytical scale cyclization to give planktocyclin **27c** and pseudostellarin **28c** respectively using HPLC Gradient 7. HPLC analysis of peptide **28a** analytical scale cyclization using HPLC Gradient 6. **S27** indicates acyl azide formation. **27d** is hydrolyzed peptide.

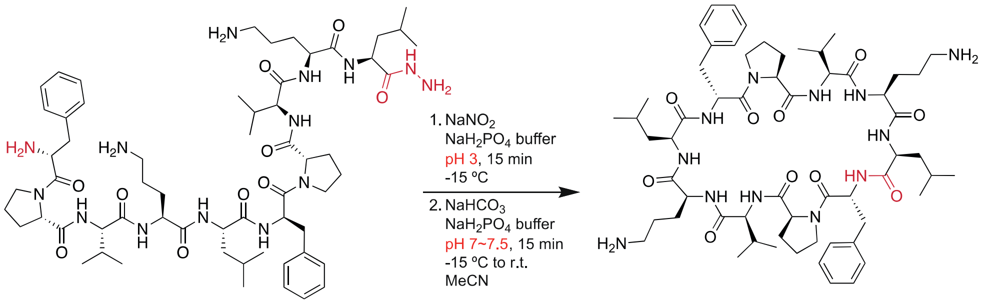

29a **29c** Gramicidin S

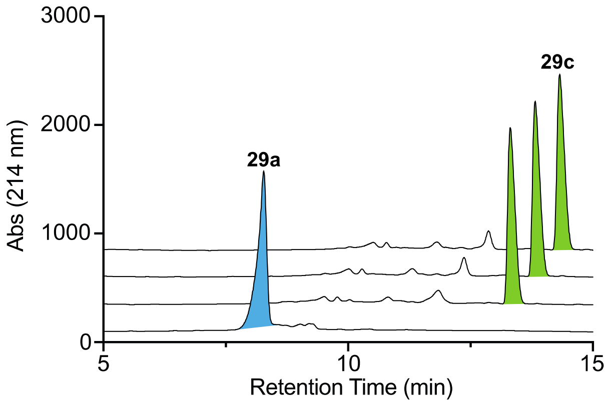

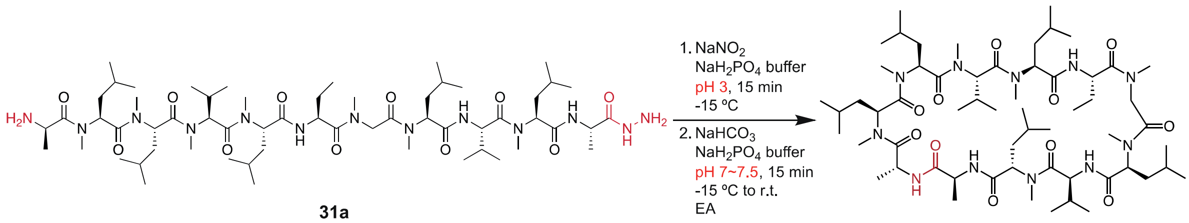

31a

31c Cyclosporine analog

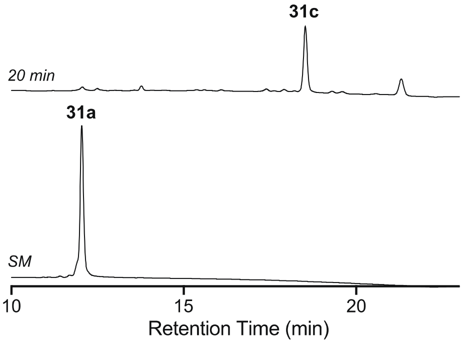

Figure S24. HPLC analysis (214 nm) of synthesis of gramicidin S **29c** and cyclosporin analogue **31c**. HPLC analysis of peptide **29a** analytical scale (1 mL reaction buffer) cyclization using HPLC Gradient 7. Chemical cyclization study of **31a** used purified peptide hydrazide, the purification of linear peptide **31a** was carried out on semi-prep HPLC using Gradient 10. HPLC analysis of peptide **31a** analytical scale (1 mL reaction buffer) cyclization using HPLC Gradient 8.

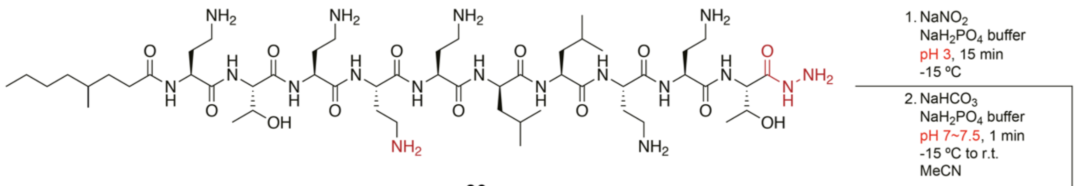

30a

20 min HPLC for monitoring the reaction

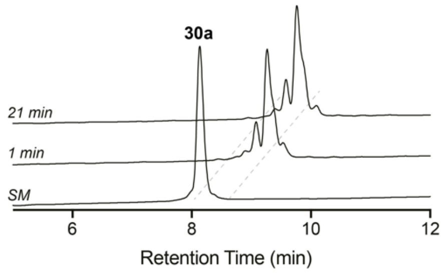

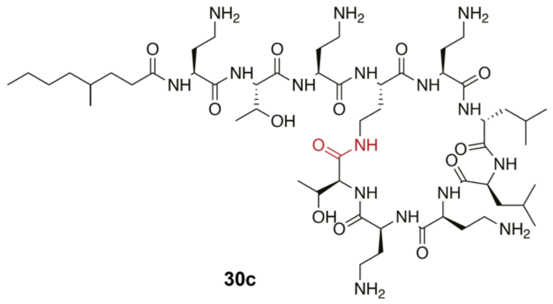

30c

35 min HPLC for separating the reaction mixture

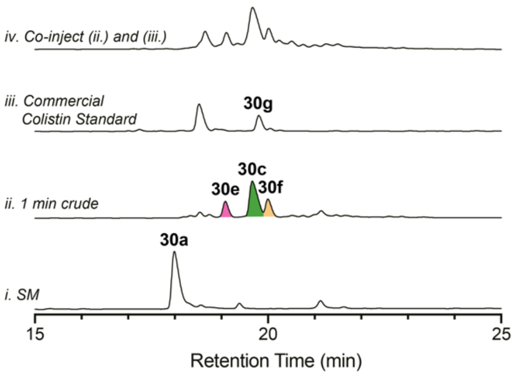

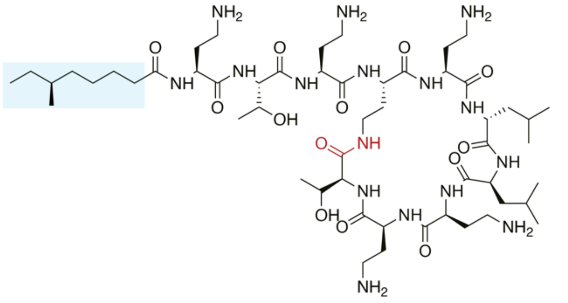

30g Colistin Standard

Figure S25. HPLC analysis (214 nm) of peptide cyclization of **30a** to produce colistin analogue **30c**. Cyclisation method is the same used for linear peptide **25a**. HPLC analysis (20 min) of peptide **30a** analytical scale (1 mL reaction buffer) cyclization using HPLC Gradient 8, HPLC analysis (35 min) using HPLC Gradient 9.

A.

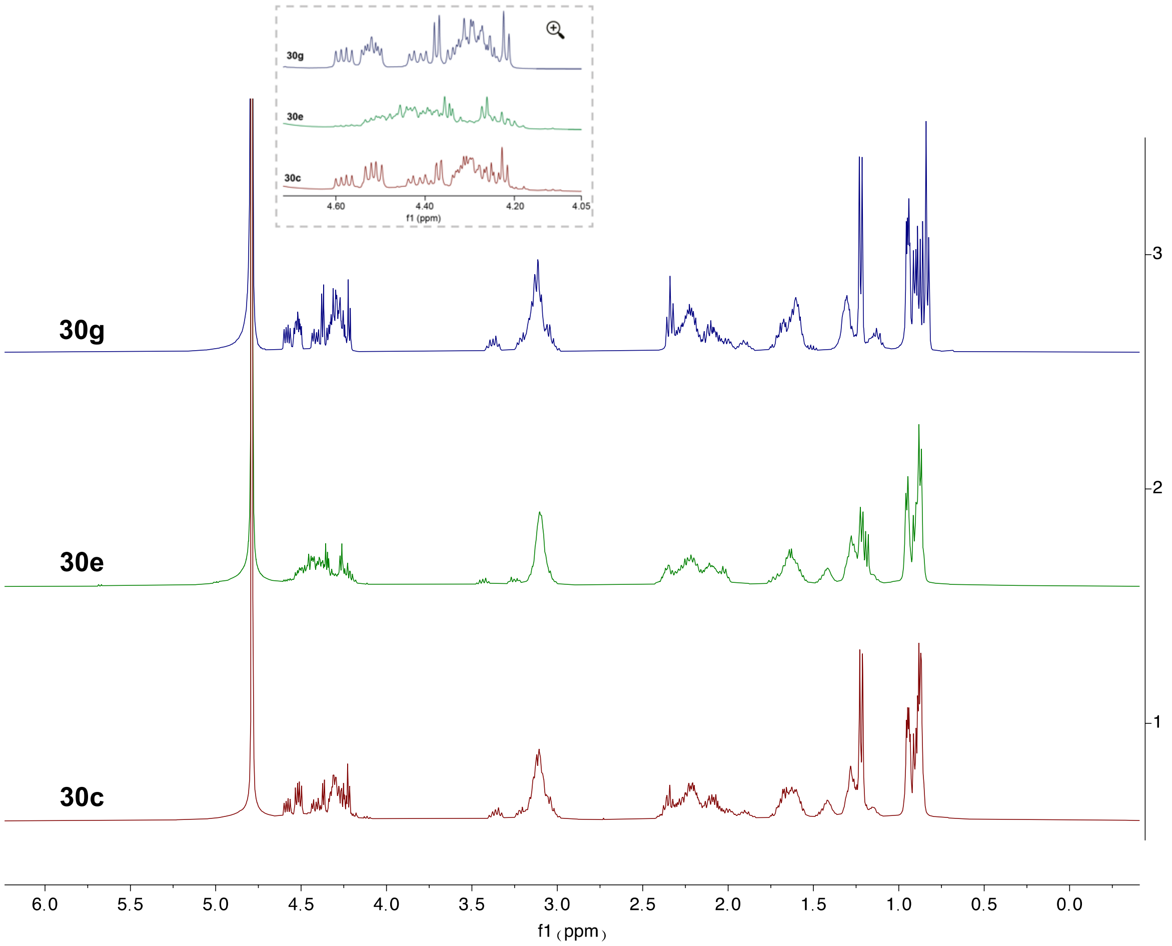
30g
30e
30c
4.60
4.40
4.20
4.05
f1 (ppm)
6.0
5.5
5.0
4.5
4.0
3.5
3.0
2.5
2.0
1.5
1.0
0.5
0.0
f1 (ppm)
3
2
1

B.

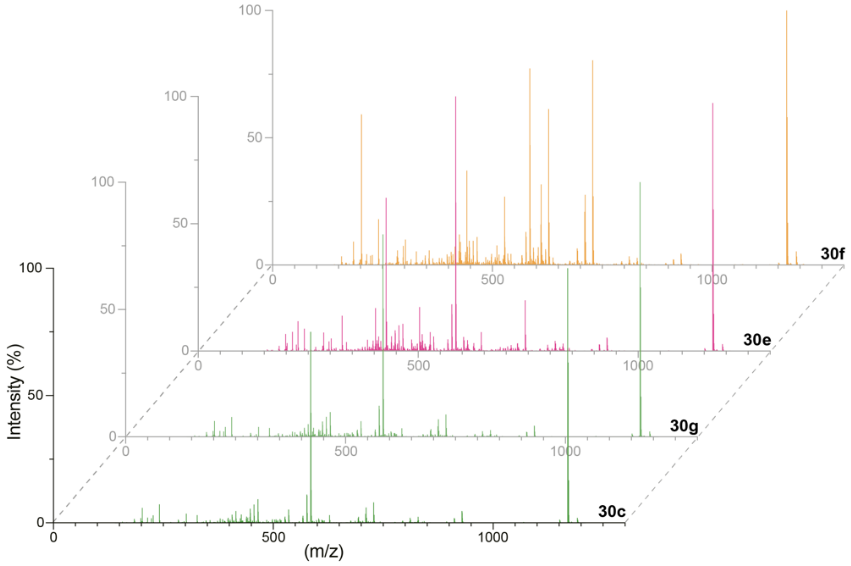
30f
30e
30g
30c
100
50
0
500
1000
Intensity (%)
(m/z)

C.

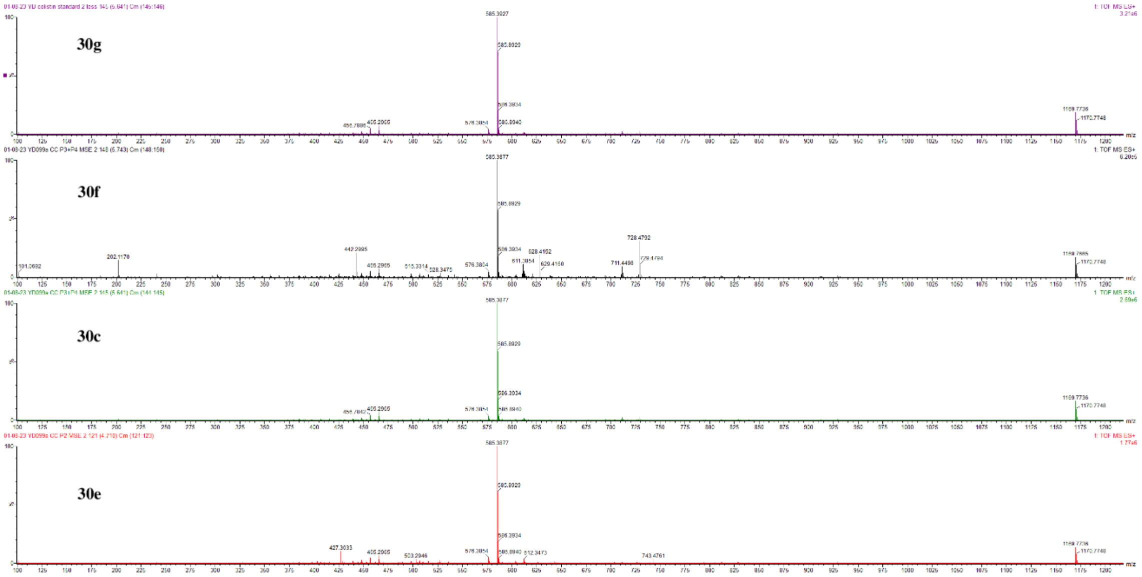

Figure S26. Comparison of ^{1}H-NMR and MS^{E} spectra of colistin analogues and a commercial standard 30g. A. NMR (D_2O, 400 MHz) and **B** & **C** Mass fragmentation patterns of all products **30c**, **30e** and **30f** compared to standard **30g**. All peptides were purified through semi-preparative HPLC using Gradient 23. (**C.** enlarged and proposed fragments in supplementary spectra)

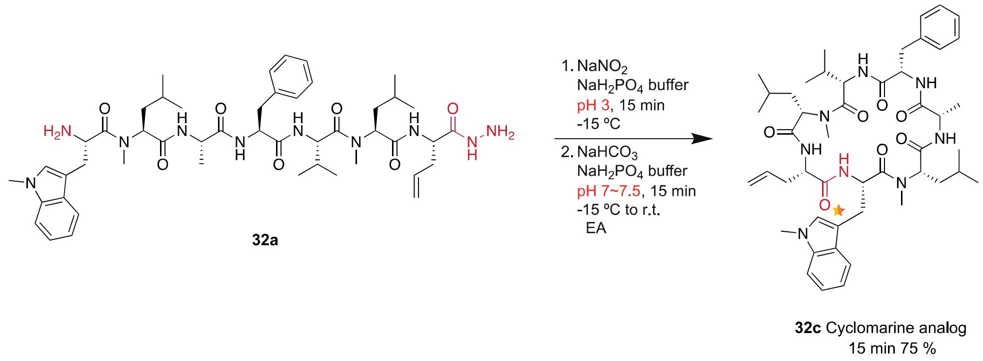

32a cyclization in 50% MeCN/H_2O

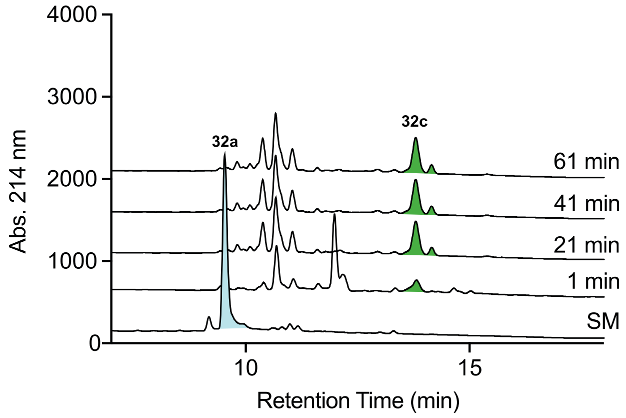

32a cyclization employing two-phase reaction

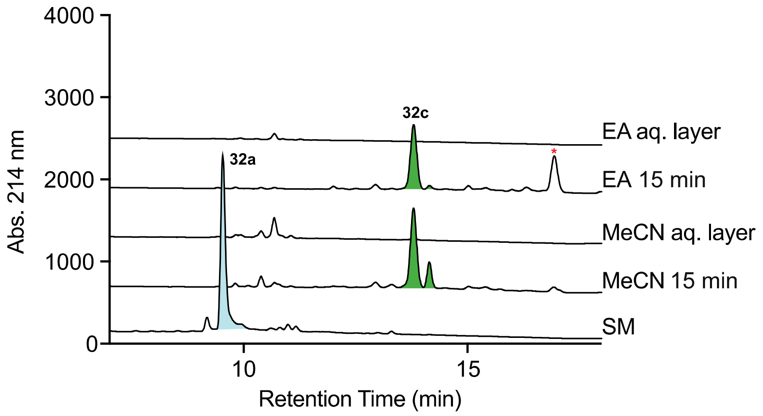

Figure S27. **Effect of solvent conditions on cyclization reaction outcome** HPLC analysis (214 nm) of peptide cyclization of **32a** to give cyclomarin analogue **32c**. HPLC analysis (20 min) of peptide **32a** analytical scale cyclization using HPLC Gradient 7. The results highlight the role of co-solvent in both product selectivity and conversion during the cyclization. Employing a two-phase reaction strategy, where the peptide is extracted into the organic layer from the aqueous phase, minimises the risk of hydrolysis, hence enhancing the cyclization by reducing the side reactions. The peak label star is an artefact of peptide aggregation on the HPLC.

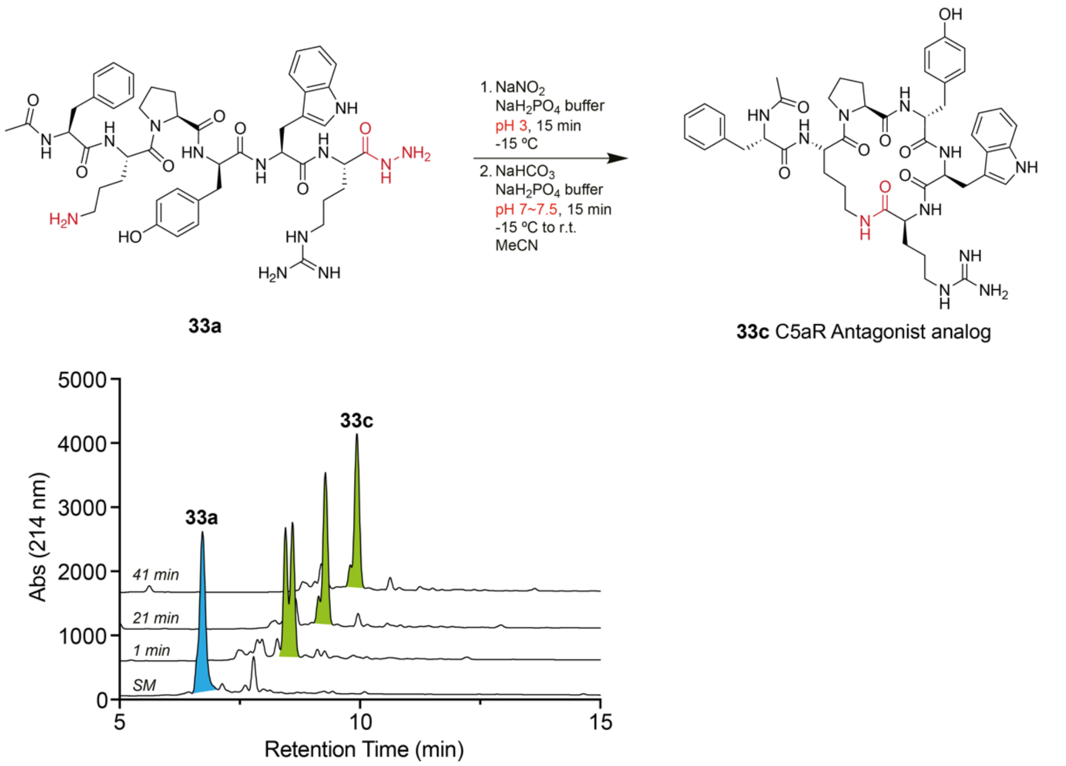
1. $NaNO_2$
NaH_2PO_4 buffer
pH 3, 15 min
-15 ºC
2. $NaHCO_3$
NaH_2PO_4 buffer
pH 7~7.5, 15 min
-15 ºC to r.t.
MeCN
33a
33c C5aR Antagonist analog
33a
33c
41 min
21 min
1 min
SM
Abs (214 nm)
Retention Time (min)

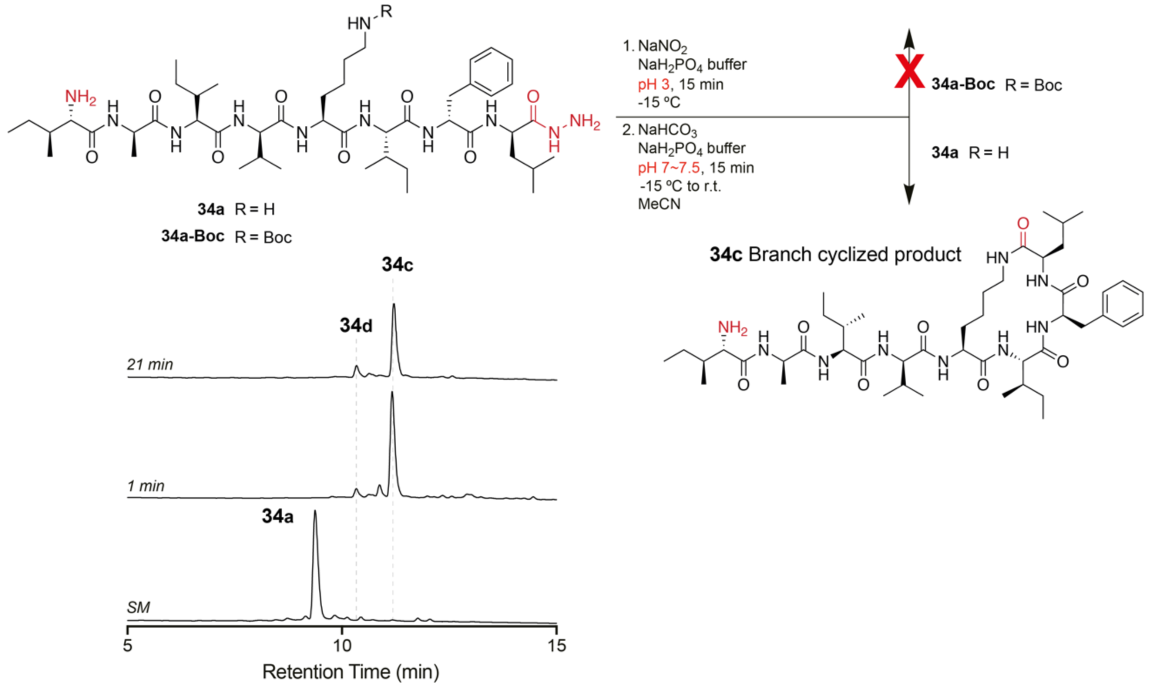
1. $NaNO_2$
NaH_2PO_4 buffer
pH 3, 15 min
-15 ºC
2. $NaHCO_3$
NaH_2PO_4 buffer
pH 7~7.5, 15 min
-15 ºC to r.t.
MeCN
34a-Boc R = Boc
34a R = H
34a R = H
34a-Boc R = Boc
34c Branch cyclized product
34c
34d
34a
21 min
1 min
SM
Retention Time (min)

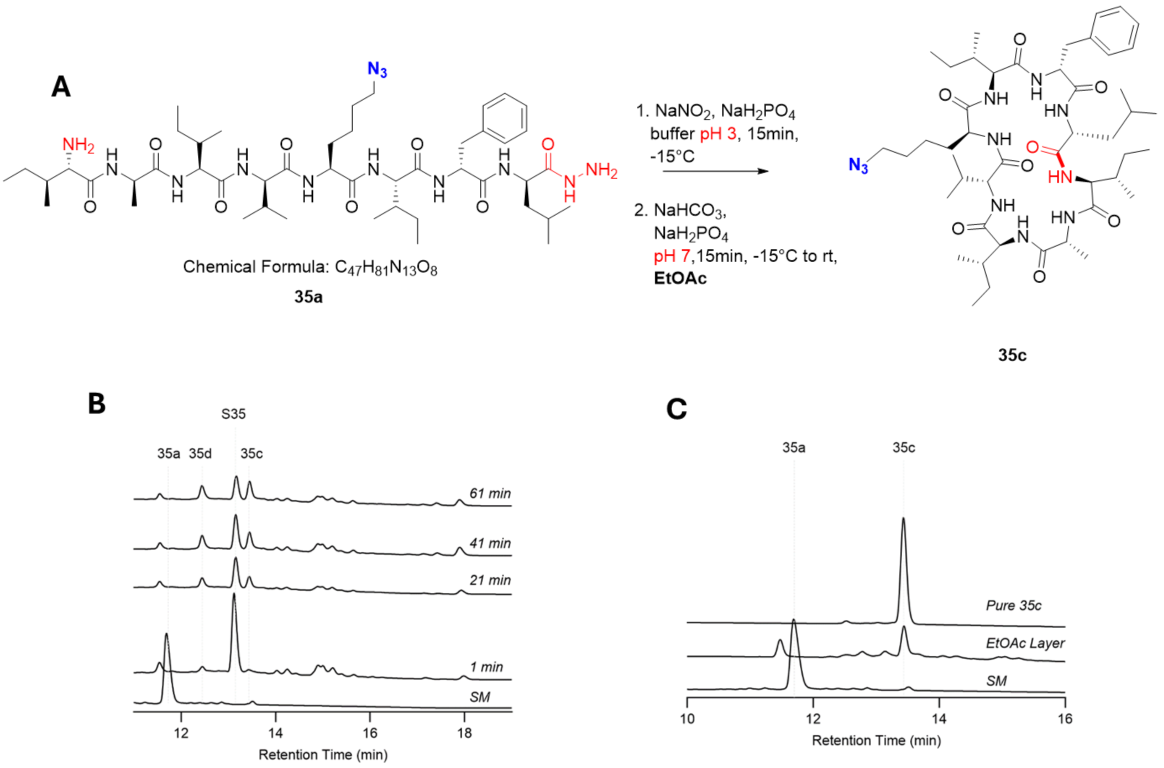

Figure S28. HPLC analysis (214 nm) of peptide cyclization of **33a, 34a, Boc-34a** and **35a**. (Top) Conversion of peptide precursor **33a** to cyclised C5aR antagonist analogue **33c. 33c** appears as *cis-trans* isomers due to proline. HPLC analysis (20 min) using HPLC Gradient 7. (Below) Attempted cyclisation of Surugamide B precursor **34a** and **Boc-34a** analytical scale cyclization using HPLC Gradient 7. **34a** results in branched cyclized product **34c**. **34d** is hydrolyzed linear peptide. **Boc-34a** gave no reaction as the peptide precipitated. **A.** Cyclisation of **35a** to give azido-surugamide B **35c** Cyclisation of **35a** is monitored using HPLC Gradient 8. **B.** shows the analytical scale reaction using acetonitrile (MeCN:Buffer) monitored over time. **C.** Indicates the reaction using EtOAc in a biphasic preparative scale reaction (Buffer 15mL, EtOAc 20 mL). **S35** is the acyl azide intermediate, **35d** is hydrolyzed peptide.

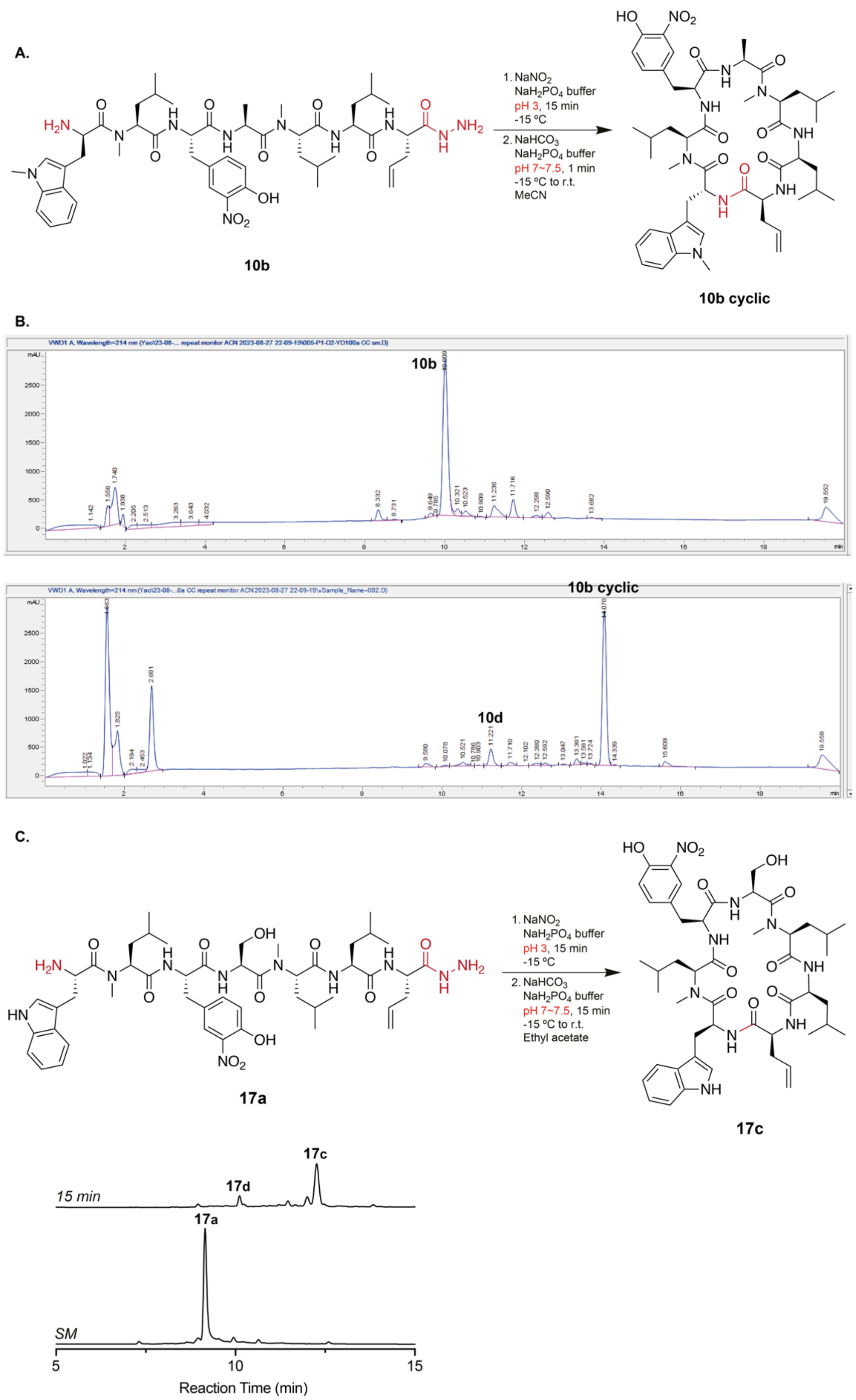

Figure S29. HPLC analysis (214 nm) of analytical scale (2.2 mM in 1 mL buffer) peptide cyclization **10b**. **A.** Reaction scheme of **10a** cyclization. **B.** HPLC analysis (20 min) of peptide **10b** using HPLC Gradient 7. **10d** is hydrolyzed linear peptide. **C.** HPLC analysis (214 nm) of peptide **17a** semi-prep scale (3 mM in 10 mL buffer) cyclization using HPLC Gradient 7. **17d** is hydrolyzed linear peptide.

A.

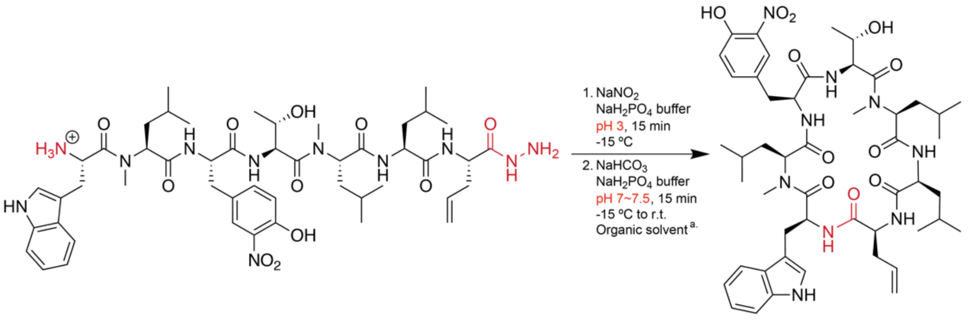

18a **18c**

B.

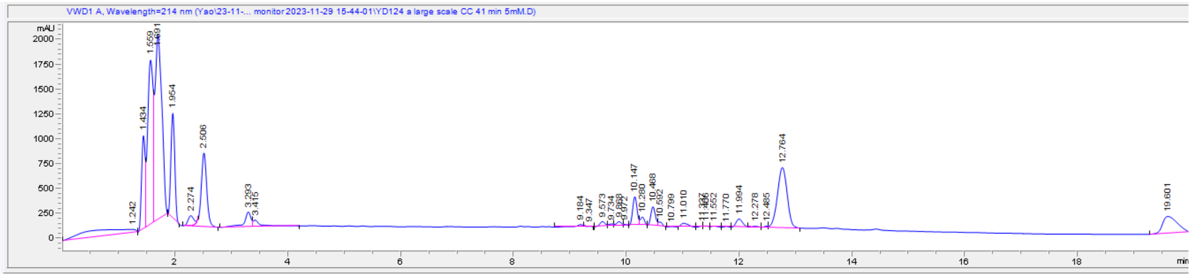

C.

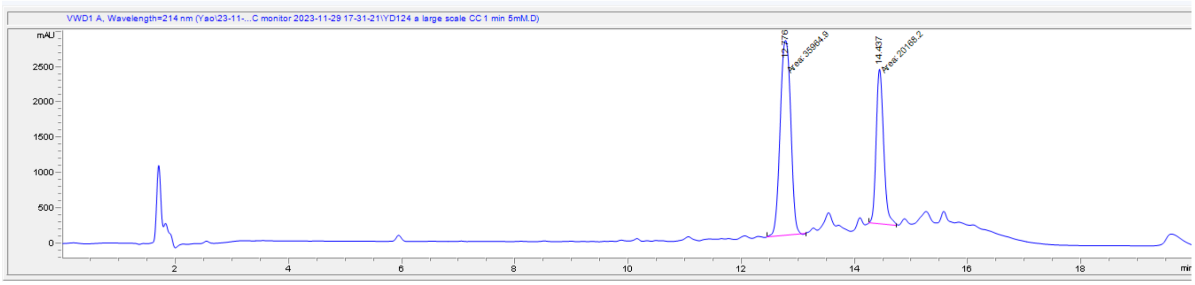

Figure S30. HPLC analysis (214 nm) of prep scale (8 mM in 40 mL buffer) peptide cyclization **18a**. **A.** reaction scheme of **18a** cyclization. **B.** using 60 mL of 50% Acetonitrile in Water + 0.1% TFA as a co-solvent. **C.** Using ethyl acetate (40 mL) as co-solvent. Poor mixing in a large round bottom flask (250 mL) leads to the peptide dimer (27%, rt: 14.437 min). HPLC analysis (20 min) of peptide **18a** cyclization using HPLC Gradient 7.

Table S2. HRMS data of synthesised hydrazide peptides (a) and cyclic peptides (c). All peptide mass listed here for $[M+H]^+$ and only monoisotopic peaks were listed in the table. All peptide samples were analyzed on UPLC-HRMS using Gradient 16.

Peptide	Acyl hydrazide-peptide (**a**)				Cyclic peptide (**c**)			
	Chemical Formula	Calc. (m/z)	Found (m/z)	Error (ppm)	Chemical Formula	Calc. (m/z)	Found (m/z)	Error (ppm)
**8**	$C_{54}H_{81}N_{11}O_{10}$	1044.6246	1044.6235	-1.05	$C_{54}H_{77}N_{9}O_{10}$	1012.5872	1012.5898	2.56
**9**	$C_{53}H_{79}N_{11}O_{10}$	1030.6090	1030.6094	0.39	$C_{53}H_{75}N_{9}O_{10}$	998.5715	998.5717	0.20
**10a**	$C_{49}H_{73}N_{11}O_{10}$	976.5620	976.5613	-0.72	$C_{49}H_{69}N_{9}O_{10}$	944.5245	944.5281	3.81
**10b**	$C_{49}H_{73}N_{11}O_{10}$	976.5620	976.5637	1.74	$C_{49}H_{69}N_{9}O_{10}$	944.5245	944.5251	0.64
**11**	$C_{46}H_{69}N_{11}O_{10}$	936.5307	936.5313	0.64	$C_{46}H_{65}N_{9}O_{10}$	904.4933	904.4977	4.86
**12**	$C_{57}H_{81}N_{11}O_{10}$	1080.6246	1080.6243	-0.28	$C_{57}H_{77}N_{9}O_{10}$	1048.5872	1048.5884	1.14
**13**	$C_{44}H_{68}N_{10}O_{10}$	897.5198	897.5229	3.44	$C_{44}H_{64}N_{8}O_{10}$	865.4824	865.4812	-1.38
**14**	$C_{52}H_{77}N_{11}O_{10}$	1016.5933	1016.5936	0.30	$C_{52}H_{73}N_{9}O_{10}$	984.5558	984.5600	4.26
**15**	$C_{52}H_{77}N_{11}O_{10}$	1016.5933	1016.5936	0.30	$C_{52}H_{73}N_{9}O_{10}$	984.5558	984.5583	2.54
**16**	$C_{51}H_{75}N_{11}O_{10}$	1002.5776	1002.5772	-0.40				

**17**	$C_{48}H_{71}N_{11}O_{11}$	978.5413	978.5443	3.06	$C_{48}H_{67}N_{9}O_{11}$	946.5038	946.5082	4.64
**18**	$C_{49}H_{73}N_{11}O_{11}$	992.5569	992.5560	-0.90	$C_{49}H_{69}N_{9}O_{11}$	960.5195	960.5178	1.76
Peptide	Acyl hydrazide-peptide (**a**)				Cyclic peptide (**c**)			
	Chemical Formula	Calc. (m/z)	Found (m/z)	Error (ppm)	Chemical Formula	Calc. (m/z)	Found (m/z)	Error (ppm)
**19**	$C_{29}H_{55}N_{9}O_{7}$	642.4303	642.4325	3.42	$C_{29}H_{51}N_{7}O_{7}$	610.3928	610.3939	1.80
**20**	$C_{25}H_{43}N_{9}O_{7}$	582.3364	582.3345	-3.26	$C_{25}H_{39}N_{7}O_{7}$	550.2990	550.2979	-1.99
**21**	$C_{21}H_{39}N_{9}O_{7}$	530.3051	530.3063	2.26	$C_{21}H_{35}N_{7}O_{7}$	498.2676	498.2657	-3.81
**22**	$C_{28}H_{53}N_{9}O_{7}$	628.4146	628.4152	0.95	$C_{28}H_{49}N_{7}O_{7}$	596.3772	596.3776	0.67
**23**	$C_{49}H_{73}N_{11}O_{10}$	976.5620	976.5607	-1.33	$C_{49}H_{69}N_{9}O_{10}$	944.5245	944.5260	1.58
**24**	$C_{49}H_{73}N_{11}O_{10}$	976.5620	976.5607	-1.33	$C_{49}H_{69}N_{9}O_{10}$	944.5245	944.5260	1.58
**25**	$C_{66}H_{91}N_{15}O_{13}$	1302.7000	1302.6963	-2.84	$C_{66}H_{87}N_{13}O_{13}$	1270.6625	1270.6639	1.10
**26**	$C_{37}H_{58}N_{10}O_{9}$	787.4467	787.4471	0.51	$C_{37}H_{54}N_{8}O_{9}$	755.4092	755.4119	3.57
Peptide	Acyl hydrazide-peptide (**a**)				Cyclic peptide (**c**)			

	Chemical Formula	Calc. (m/z)	Found (m/z)	Error (ppm)	Chemical Formula	Calc. (m/z)	Found (m/z)	Error (ppm)
**27**	$C_{39}H_{64}N_{10}O_8S$	833.4708	833.4727	2.28	$C_{39}H_{60}N_8O_8S$	801.4333	801.4362	3.62
**28**	$C_{25}H_{39}N_7O_6$	534.3040	534.3059	3.55	$C_{25}H_{35}N_5O_6$	502.2666	502.2666	0.00
**29**	$C_{60}H_{96}N_{14}O_{10}$	1173.7512	1173.7493	-1.61	$C_{60}H_{92}N_{12}O_{10}$	1141.7137	1141.7142	0.44
**30**	$C_{53}H_{104}N_{18}O_{13}$	1201.8108	1201.8123	-1.25	$C_{53}H_{100}N_{16}O_{13}$	1169.7734	1169.7736	0.17
**31**	$C_{59}H_{111}N_{13}O_{11}$	1178.8605	1178.8635	2.54	$C_{59}H_{107}N_{11}O_{11}$	1146.8230	1146.8260	2.61
**32**	$C_{48}H_{72}N_{10}O_7$	901.5663	901.5663	0.00	$C_{48}H_{68}N_8O_7$	869.5289	869.5289	0.00
**33**	$C_{47}H_{63}N_{13}O_8$	938.5001	938.5041	4.25	$C_{47}H_{59}N_{11}O_8$	906.4626	906.4641	1.65
**34**	$C_{47}H_{83}N_{11}O_8$	930.6505	930.6502	-0.32	$C_{47}H_{79}N_9O_8$	898.6130	898.6132	0.22
**Boc-34**	$C_{52}H_{91}N_{11}O_{10}$	1030.7029	1030.6995	-3.3				
**35**	$C_{47}H_{81}N_{13}O_8$	956.6409	956.6416	0.73	$C_{47}H_{77}N_{11}O_8$	924.6025	924.6007	-1.95

3. Supplementary Characterization Data

3.1 ^{1}H and ^{13}C NMR spectra

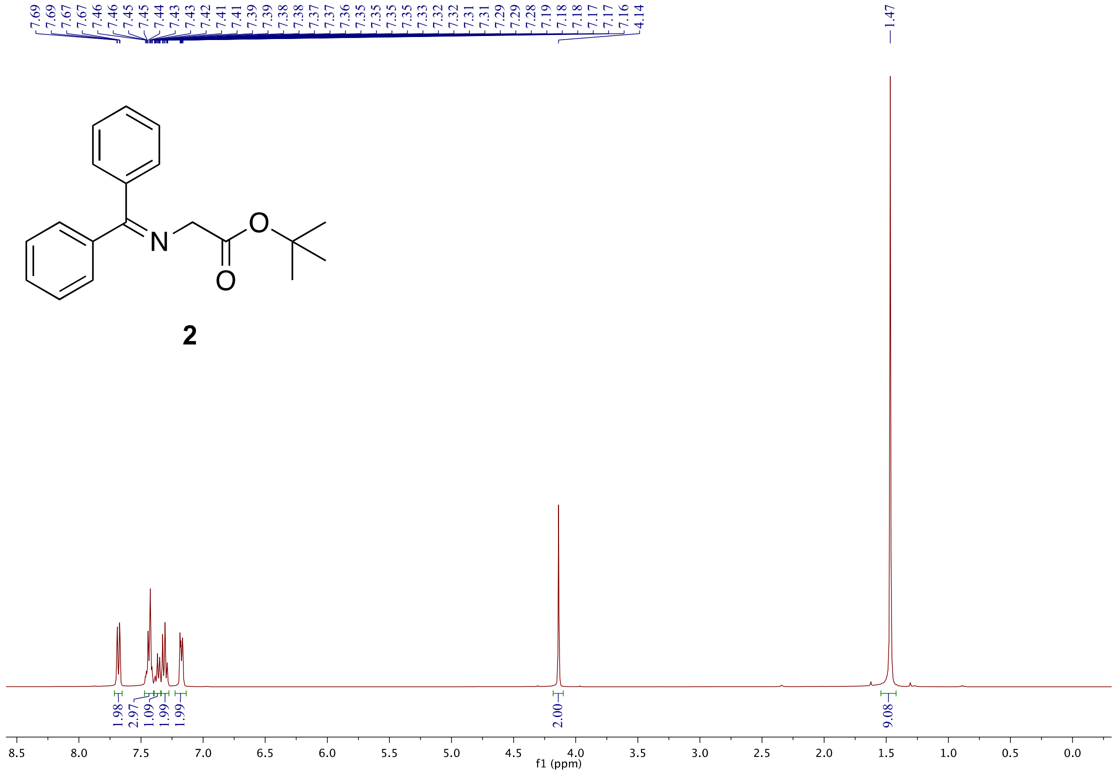

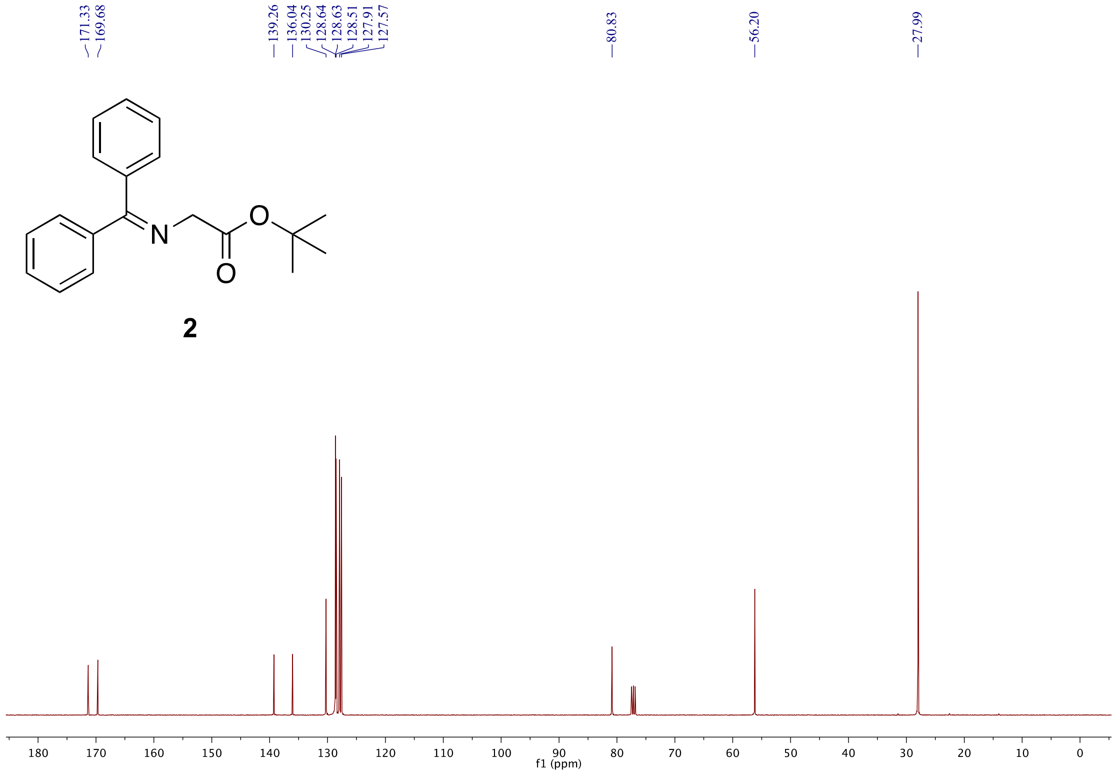

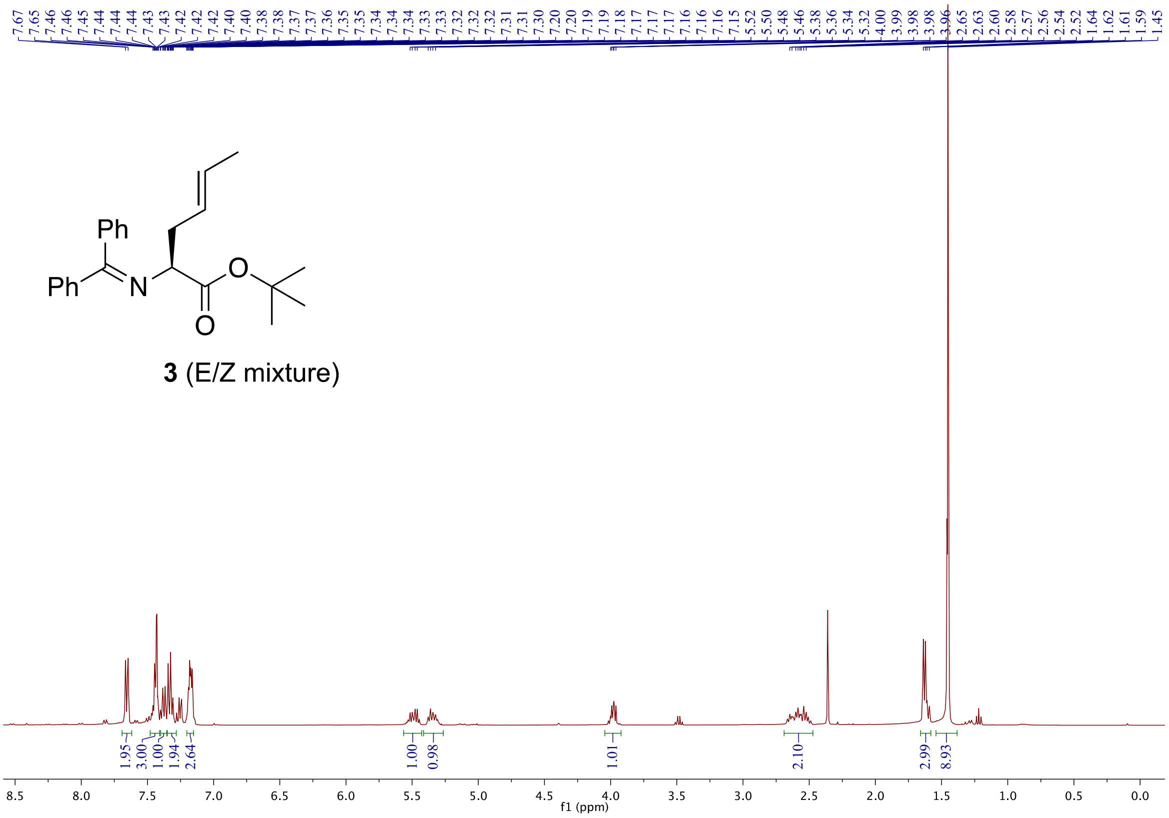

3 (E/Z mixture)

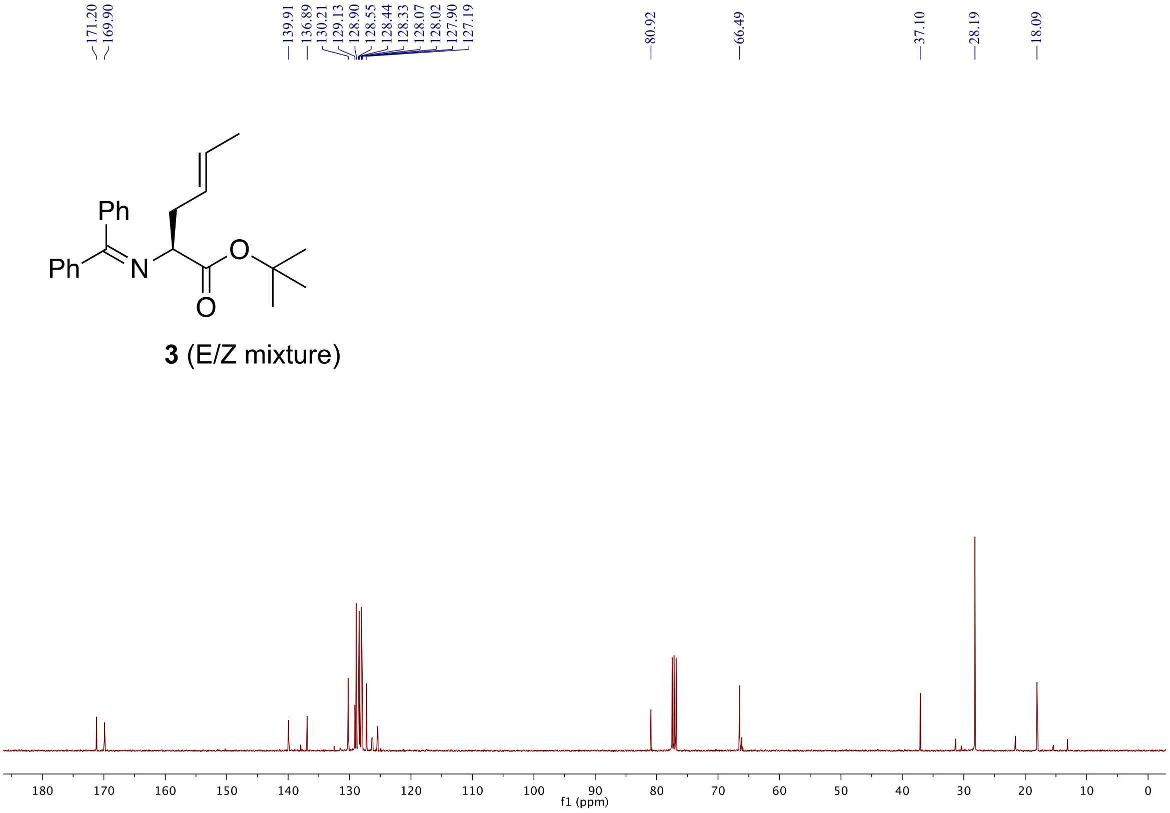

3 (E/Z mixture)

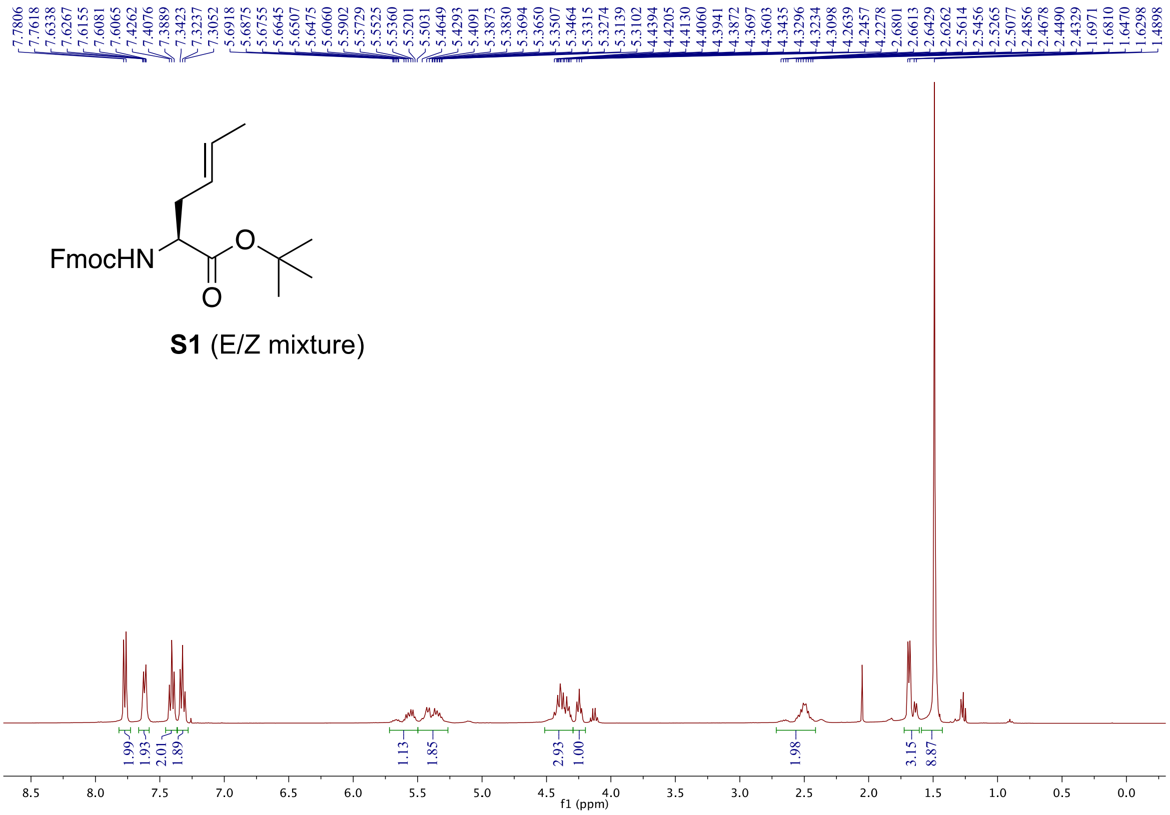

S1 (E/Z mixture)

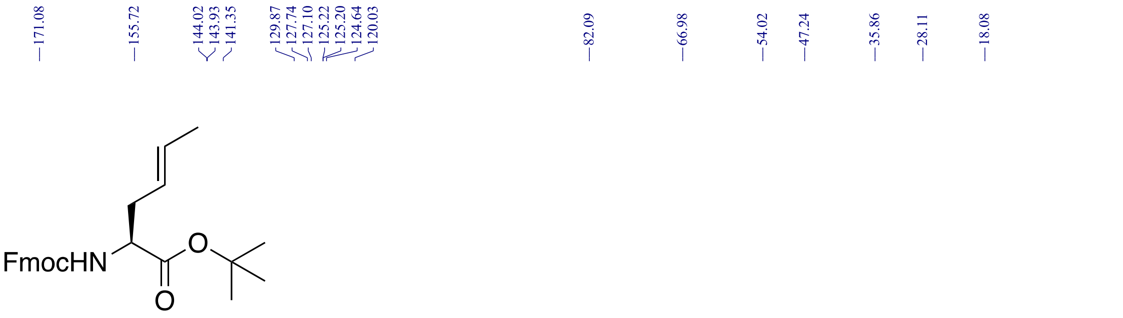

S1 (E/Z mixture)

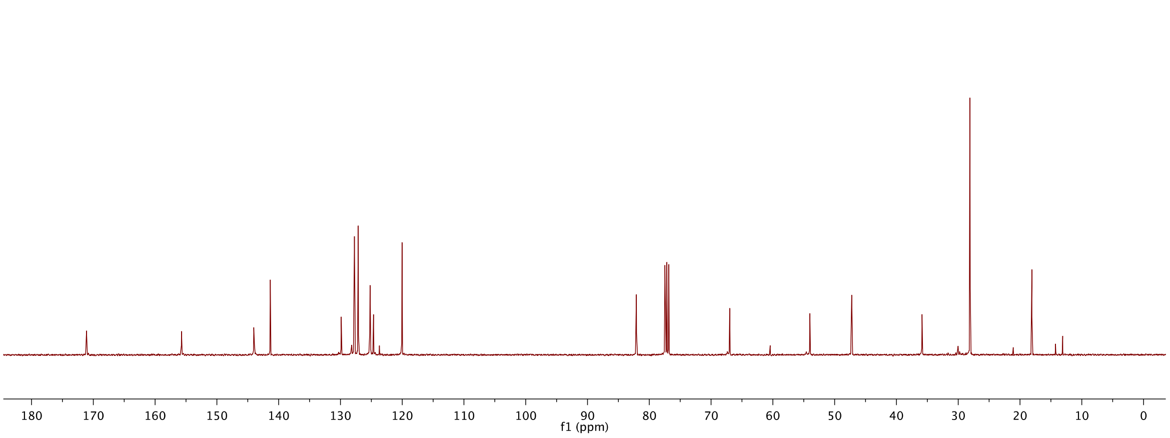

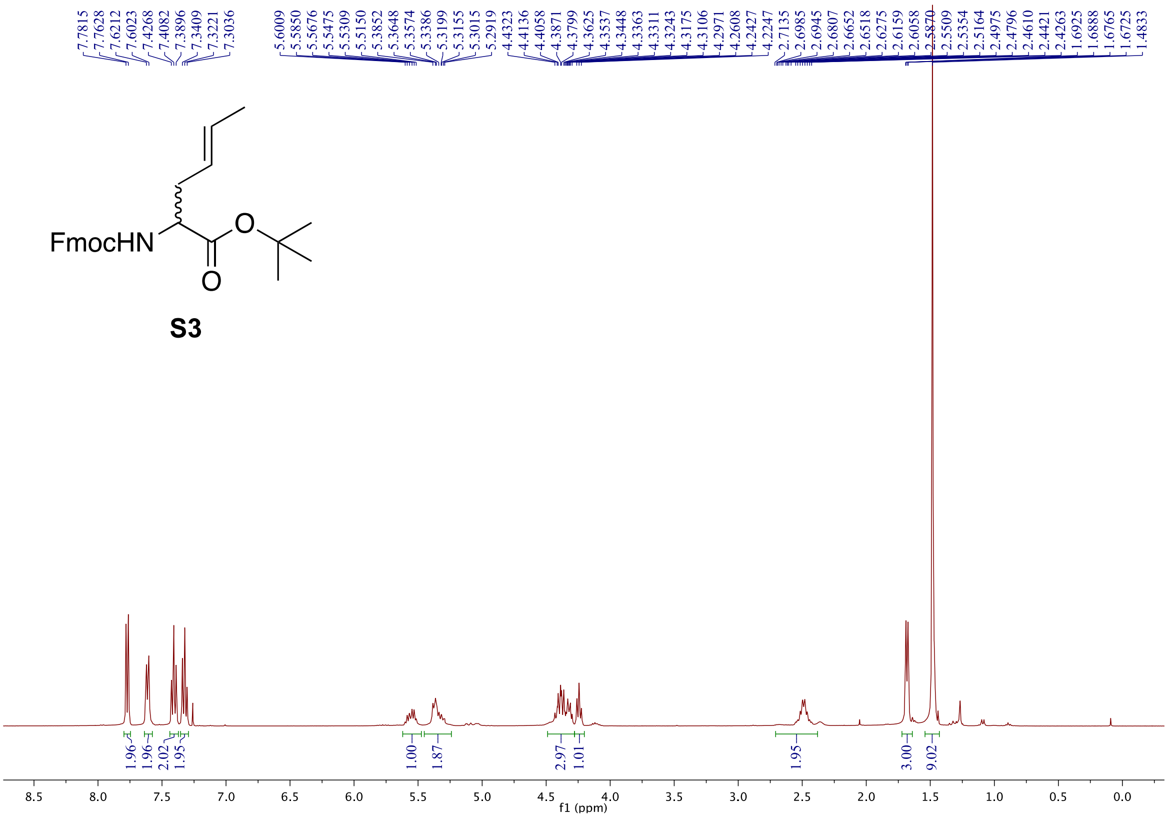
FmocHN
O
O
7.7815
7.7628
7.6212
7.6023
7.4268
7.4082
7.3896
7.3409
7.3221
7.3036
5.6009
5.5850
5.5676
5.5475
5.5309
5.5150
5.3852
5.3648
5.3574
5.3386
5.3199
5.3155
5.3015
5.2919
4.4323
4.4136
4.4058
4.3871
4.3799
4.3625
4.3537
4.3448
4.3363
4.3311
4.3243
4.3175
4.3106
4.2971
4.2608
4.2427
4.2247
2.7135
2.6985
2.6945
2.6807
2.6652
2.6518
2.6275
2.6159
2.6058
2.5870
2.5509
2.5354
2.5164
2.4975
2.4796
2.4610
2.4421
2.4263
1.6925
1.6888
1.6765
1.6725
1.4833
1.96
1.96
2.02
1.95
1.00
1.87
2.97
1.01
1.95
3.00
9.02
f1 (ppm)

S3

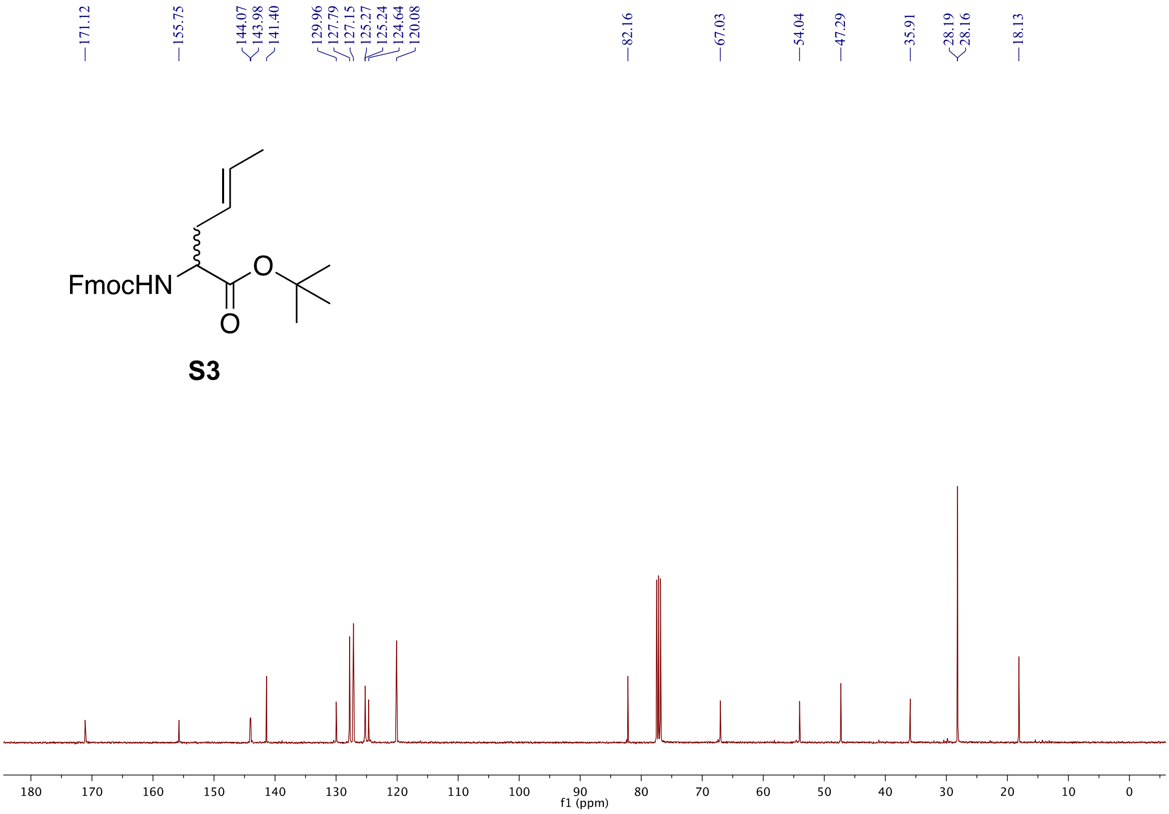
FmocHN
O
O
171.12
155.75
144.07
143.98
141.40
129.96
127.79
127.15
125.27
125.24
124.64
120.08
82.16
67.03
54.04
47.29
35.91
28.19
28.16
18.13
f1 (ppm)

S3

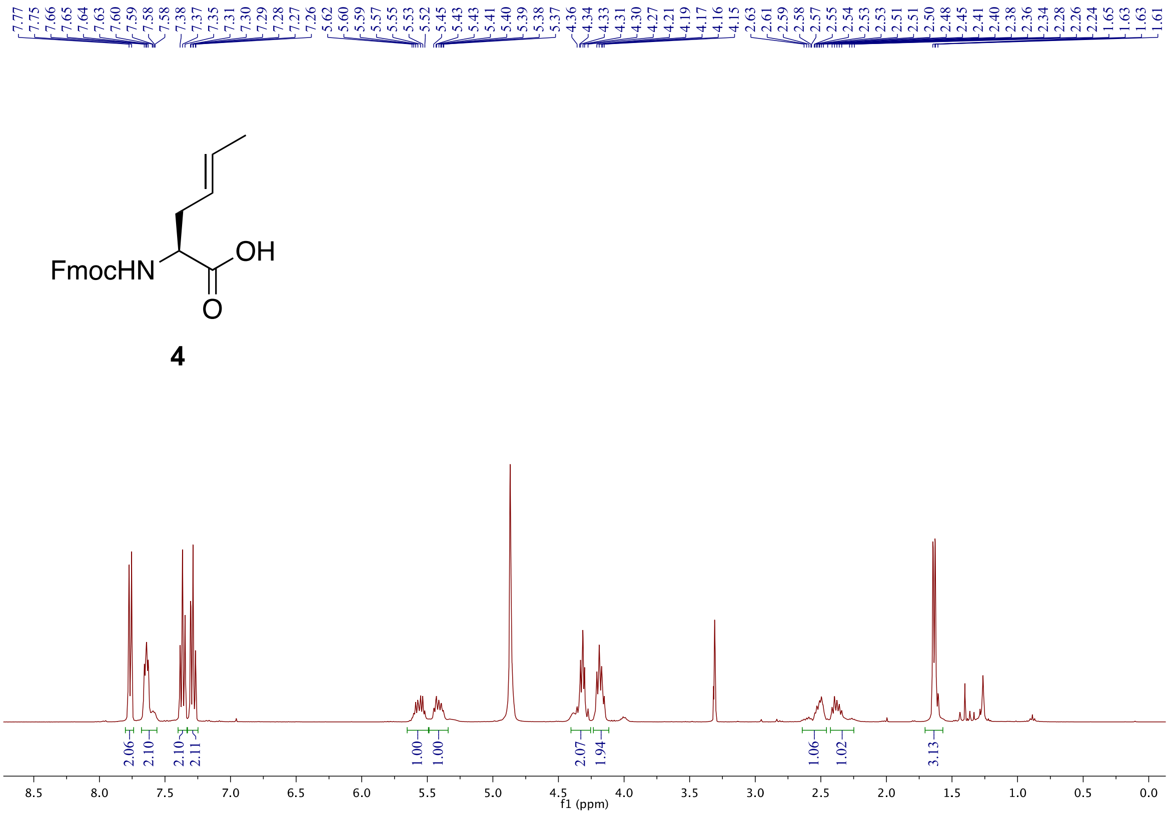

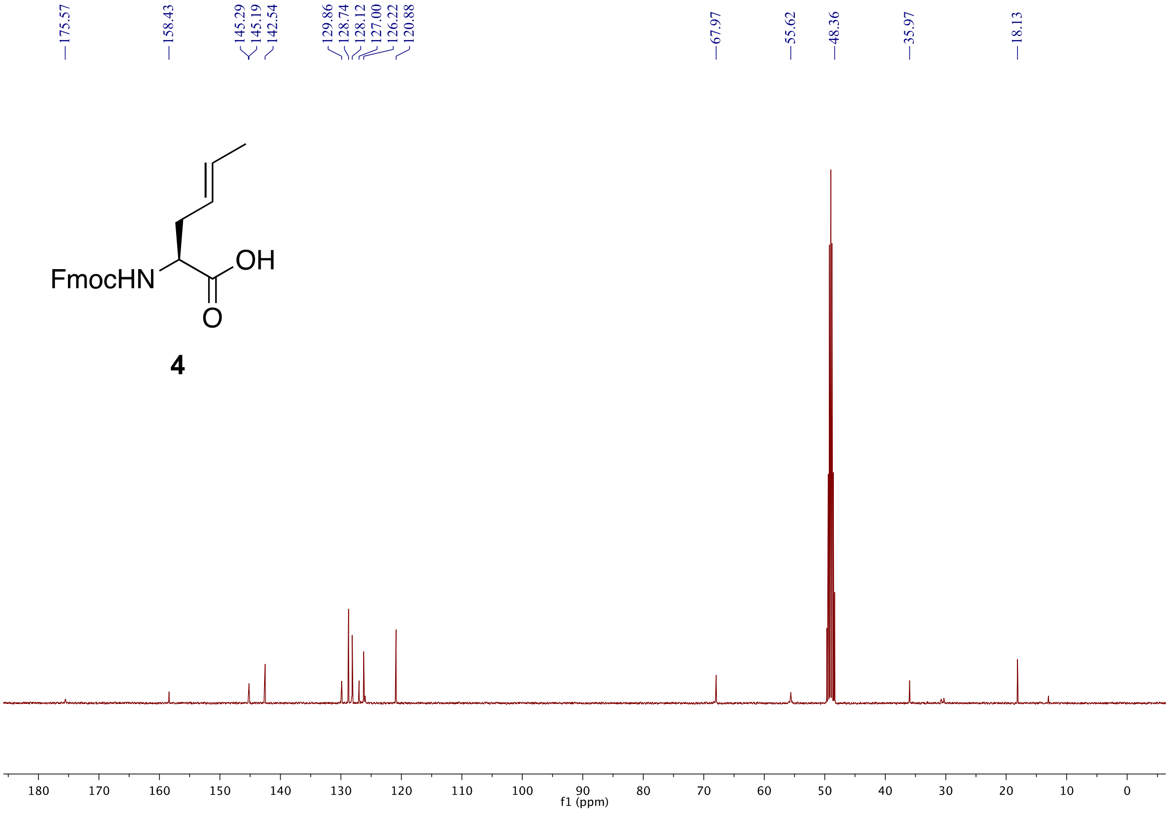

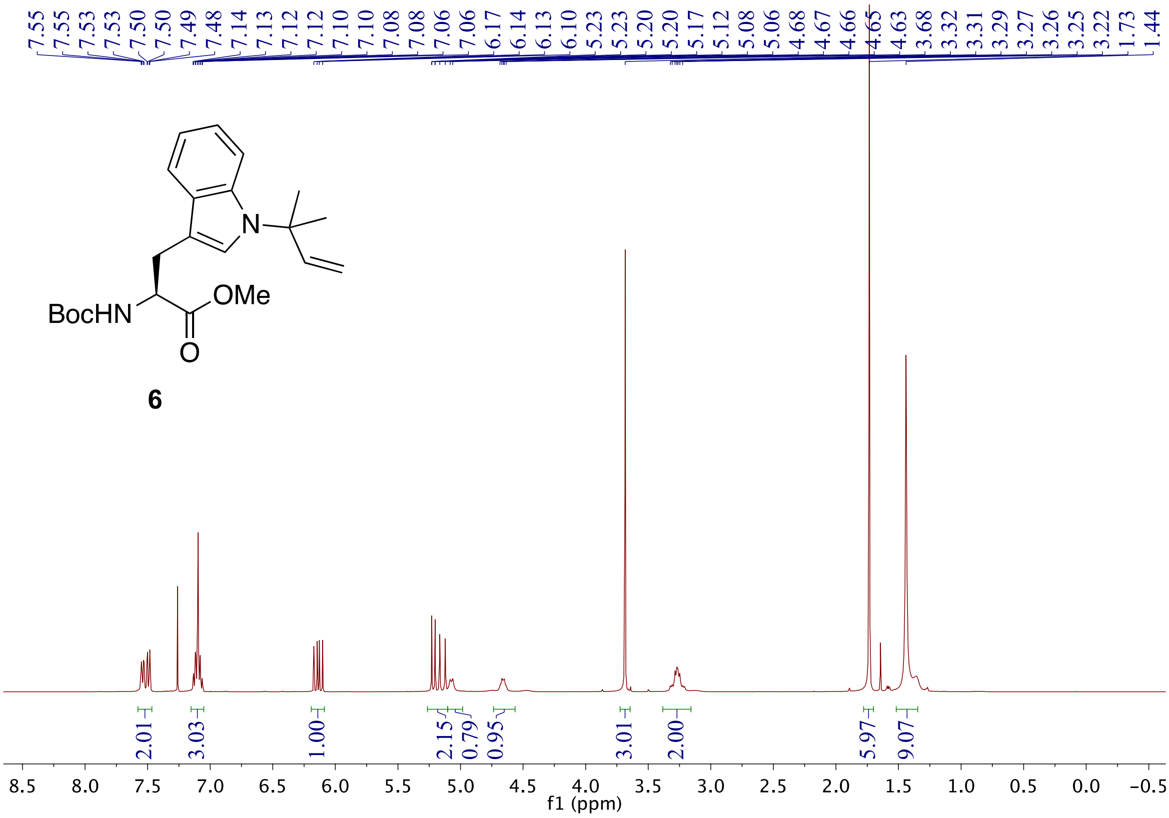
7.55
7.55
7.53
7.53
7.50
7.50
7.49
7.48
7.14
7.13
7.12
7.12
7.10
7.10
7.08
7.08
7.06
7.06
6.17
6.14
6.13
6.10
5.23
5.23
5.20
5.20
5.17
5.12
5.08
5.06
4.68
4.67
4.66
4.65
4.63
3.68
3.32
3.31
3.29
3.27
3.26
3.25
3.22
1.73
1.44
BocHN
OMe
O
N
6
2.01
3.03
1.00
2.15
0.79
0.95
3.01
2.00
5.97
9.07
f1 (ppm)

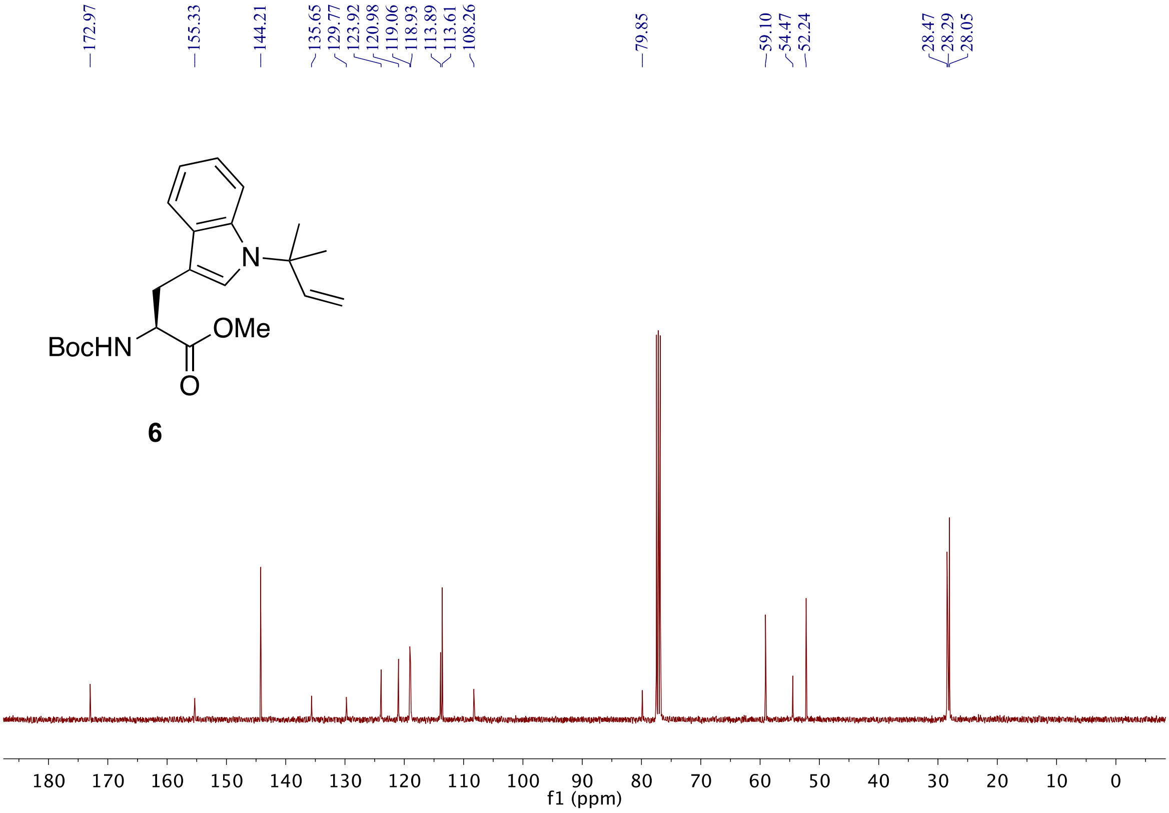
172.97
155.33
144.21
135.65
129.77
123.92
120.98
119.06
118.93
113.89
113.61
108.26
79.85
59.10
54.47
52.24
28.47
28.29
28.05
BocHN
OMe
O
N
6
f1 (ppm)

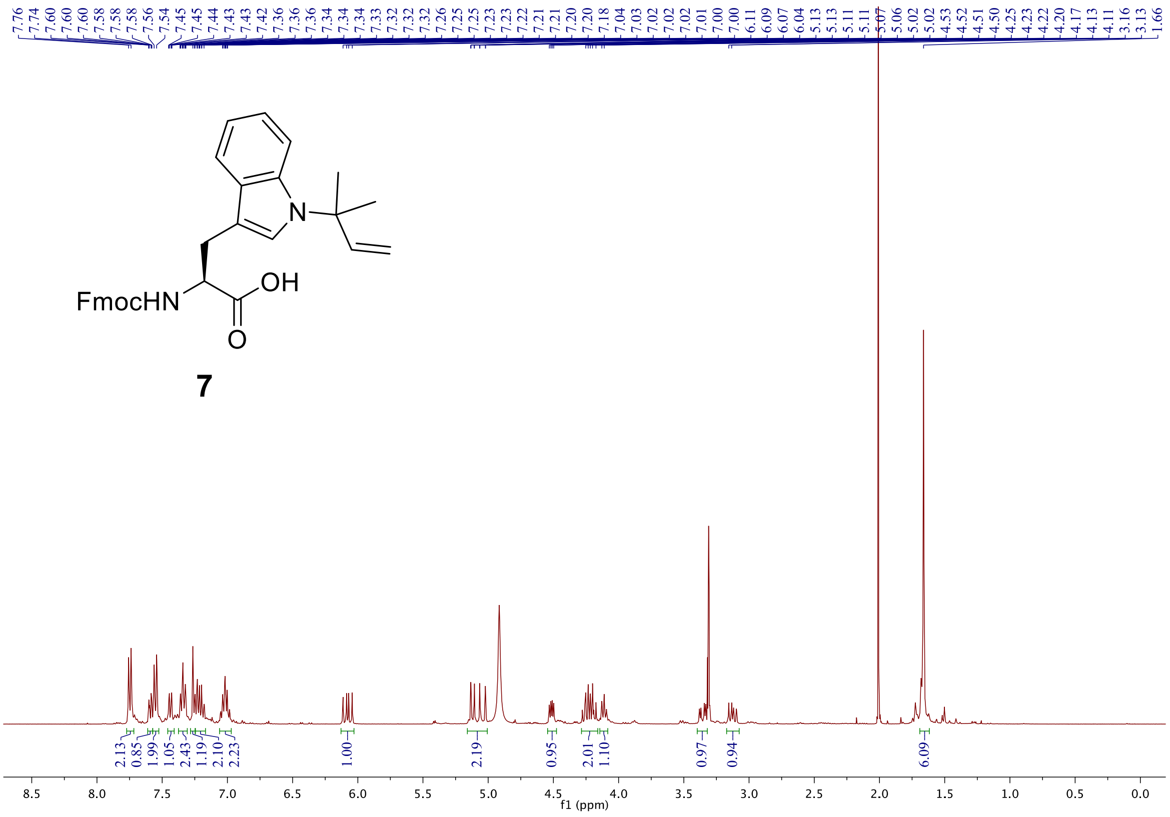

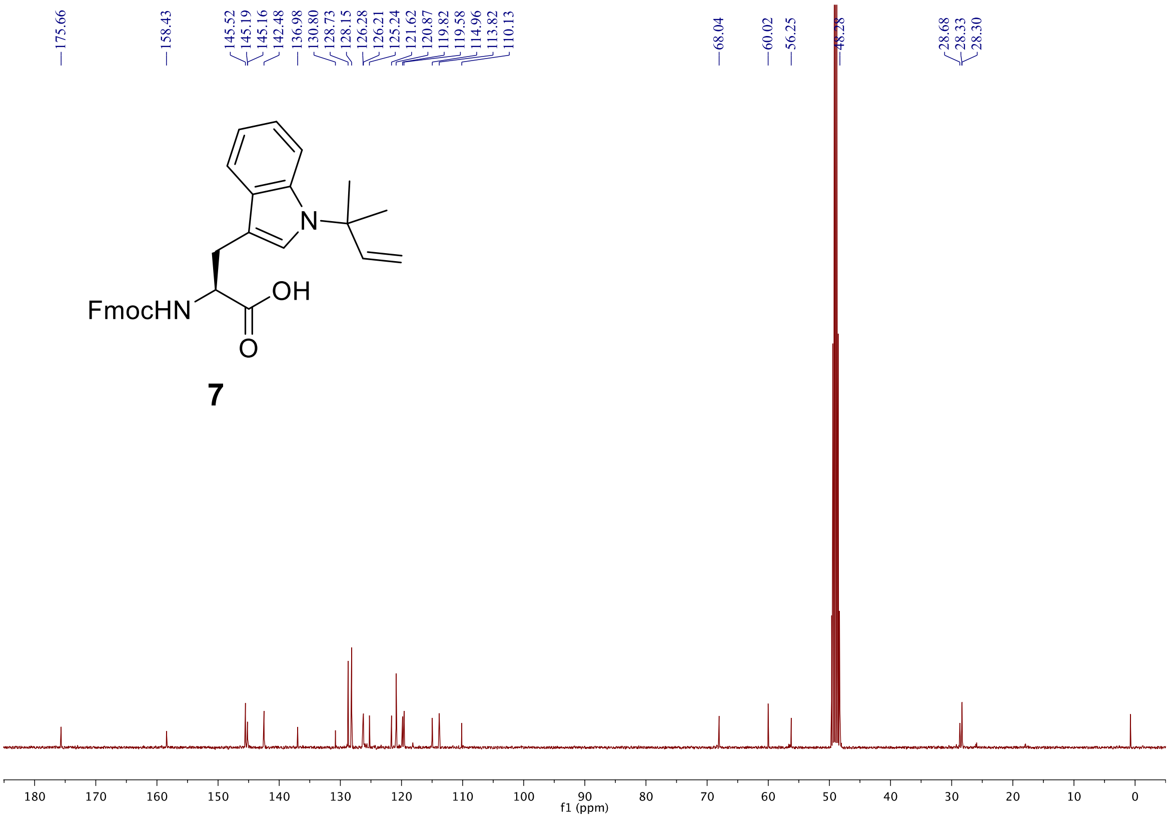

^{1}H NMR spectrum of **8c** in CD_3OD at 700 MHz.

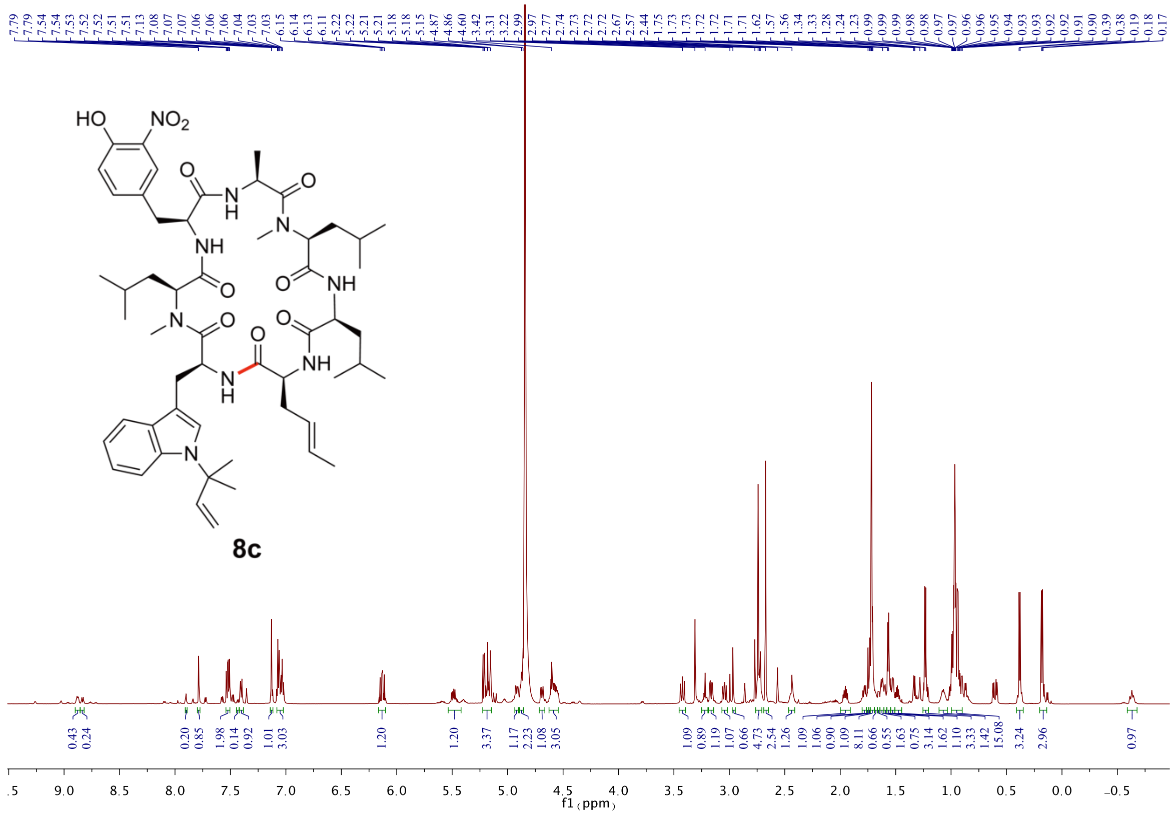

^{13}C NMR spectrum of **8c** in CD_3OD at 176 MHz.

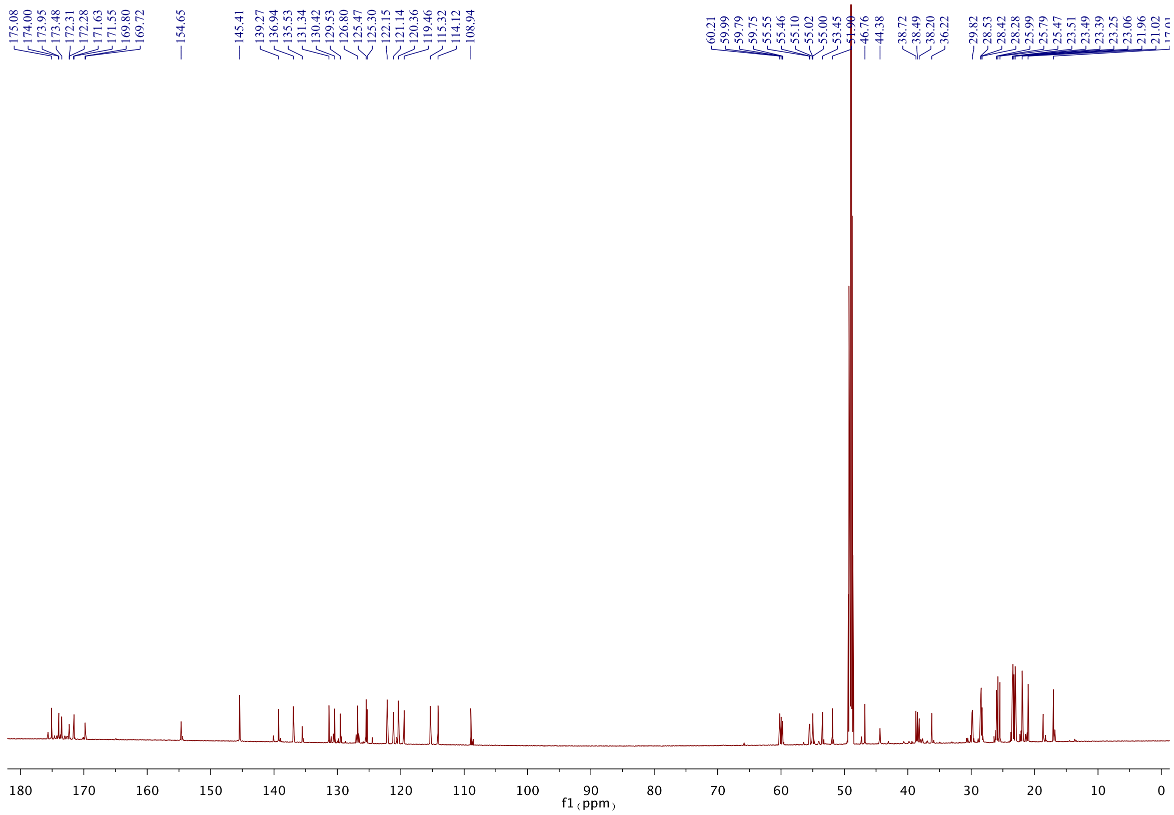

COSY spectrum of ***8c*** *in CD_3OD.*

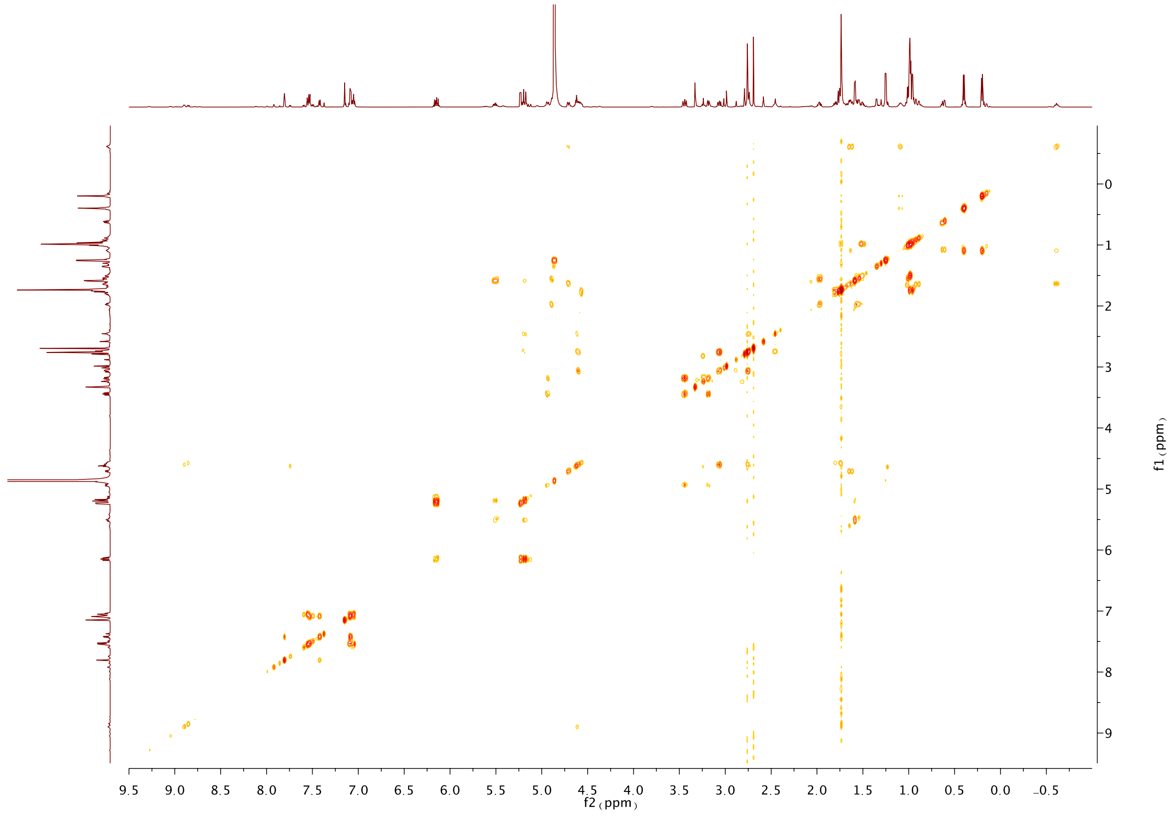

HSQC spectrum of ***8c*** *in CD_3OD.*

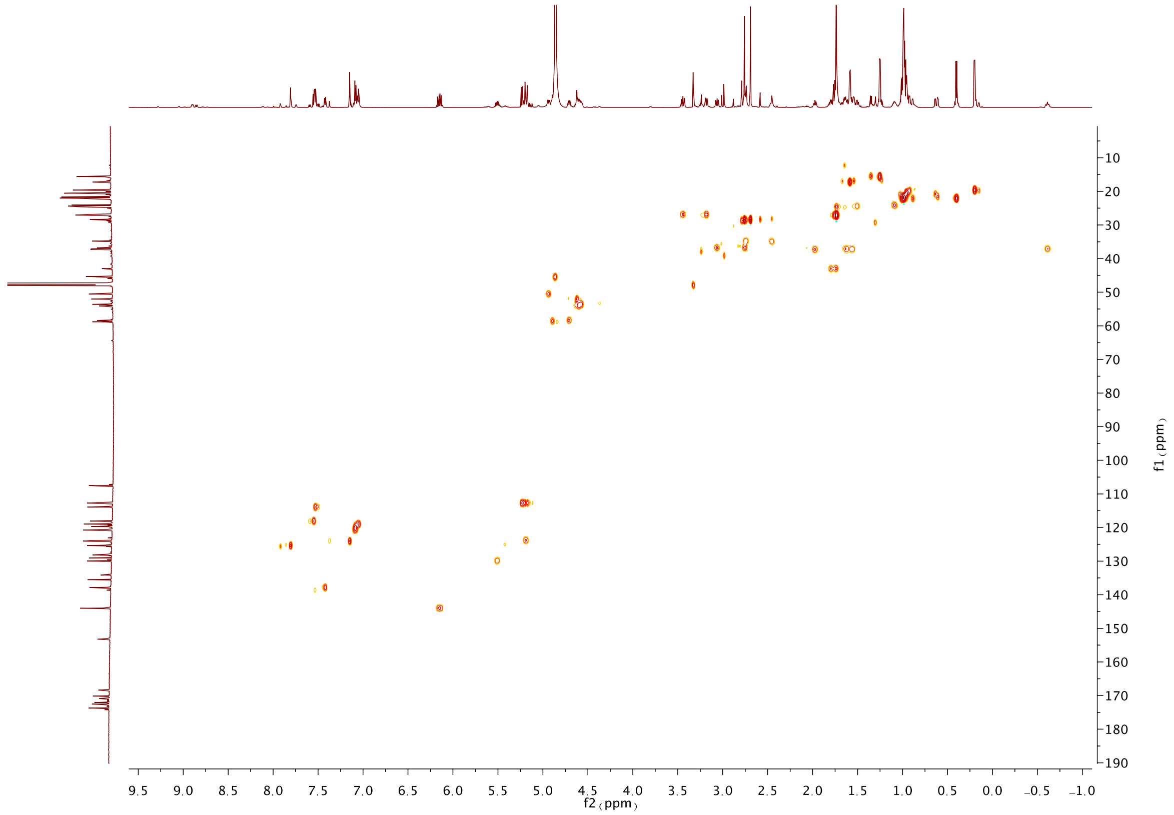

*HMBC spectrum of **8c** in CD_3OD.*

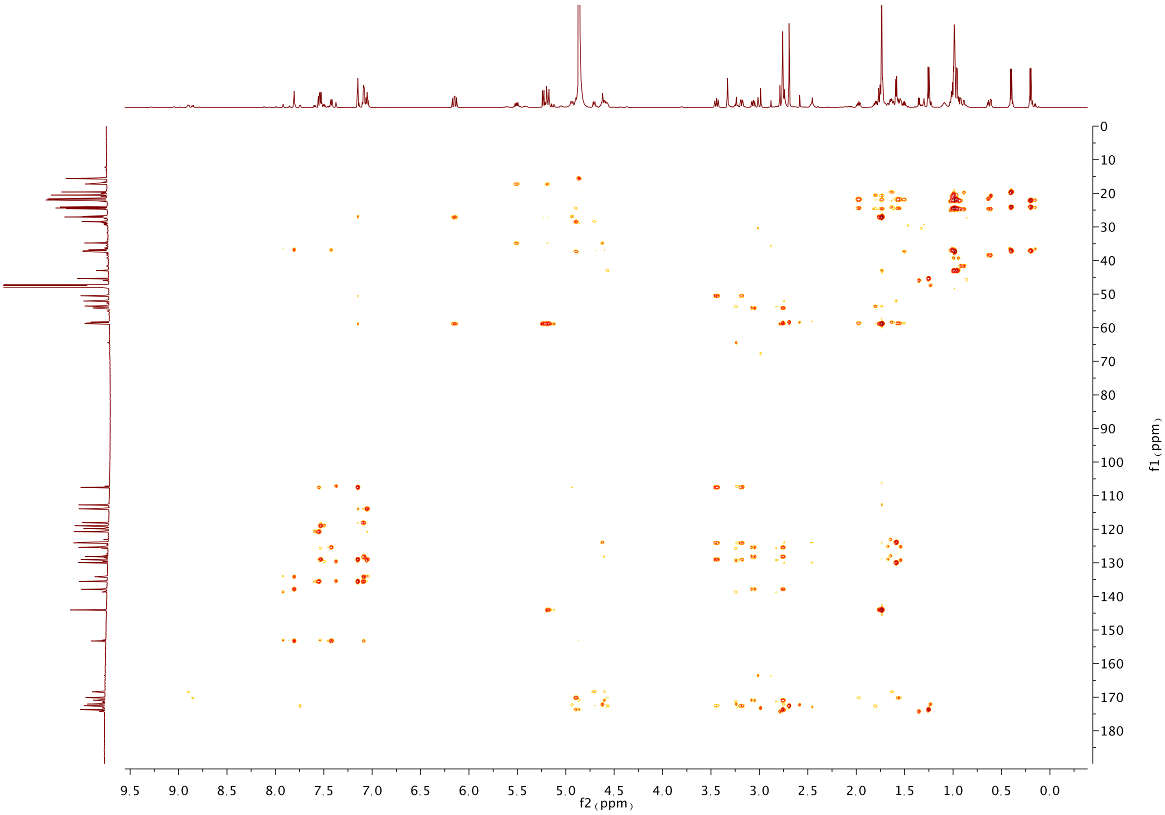

^{1}H NMR spectrum of rufomycin analogue **9c** in CD_3OD at 700 MHz.

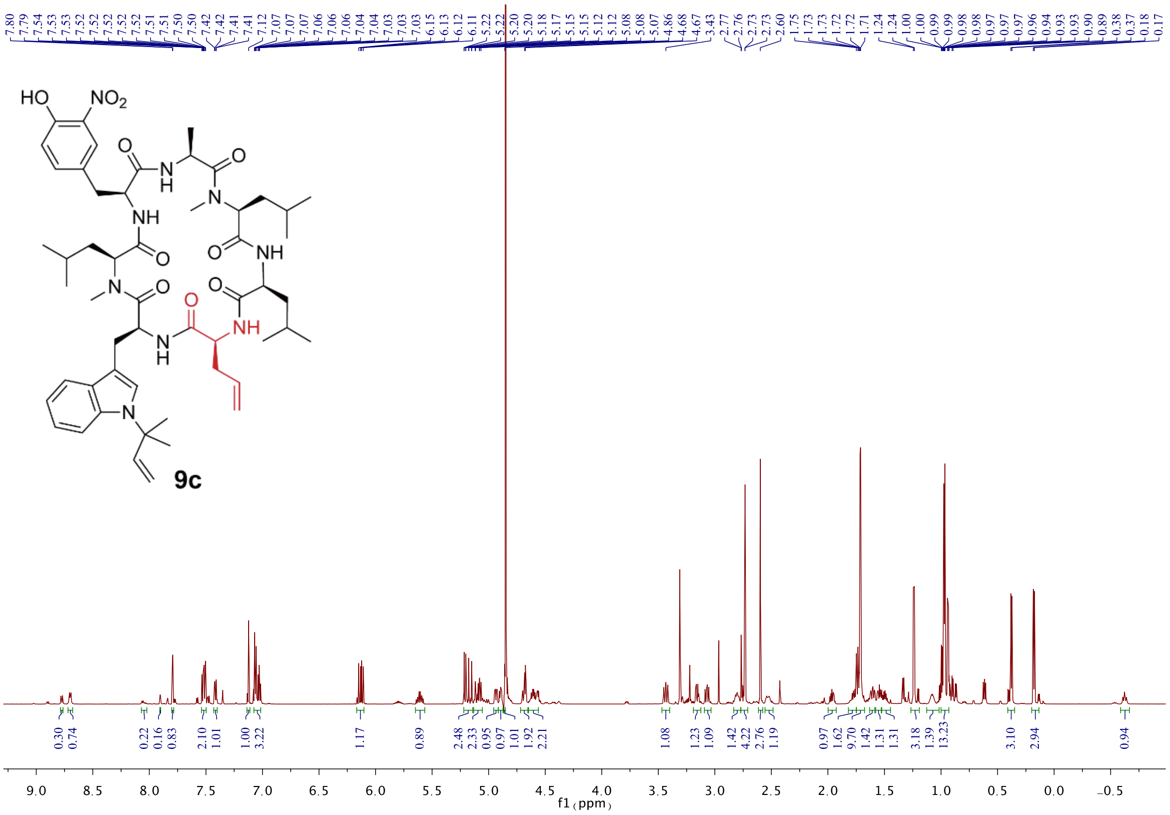

^{13}C NMR spectrum of rufomycin analogue **9c** in CD_3OD at 176 MHz.

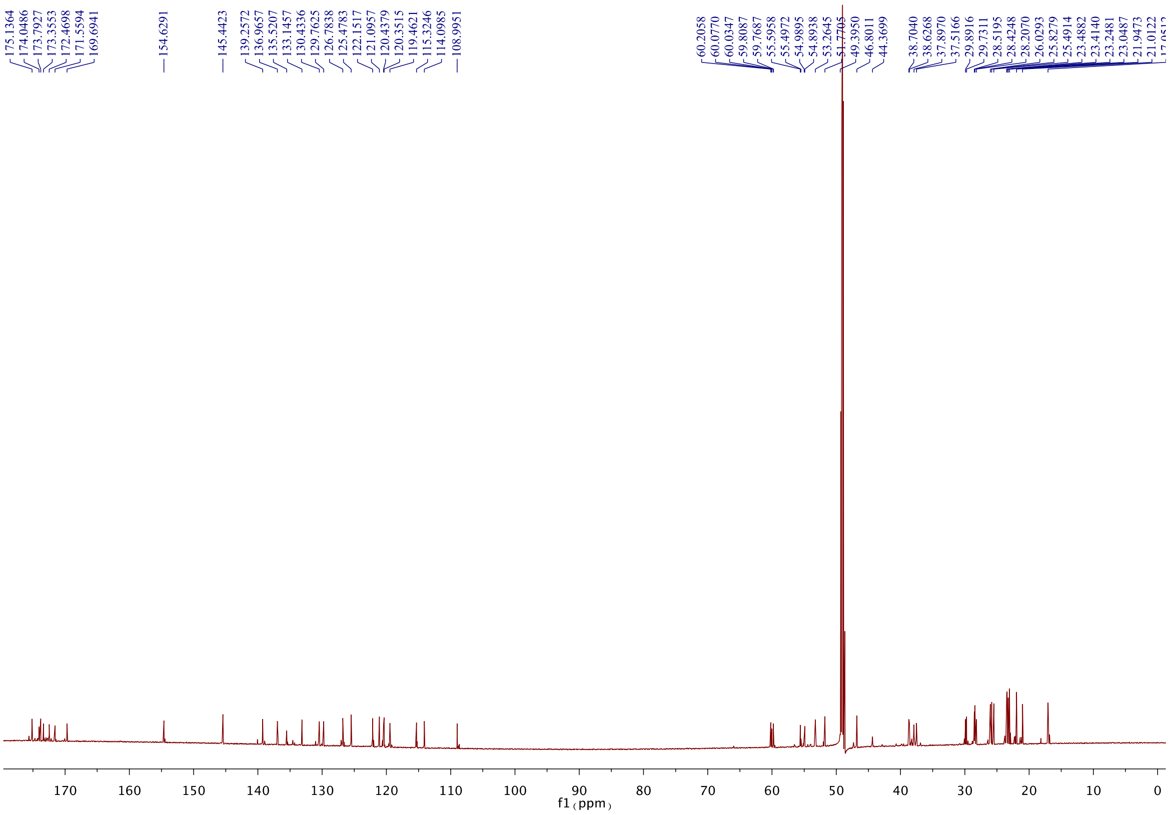

*COSY spectrum of rufomycin analogue **9c** in CD_3OD.*

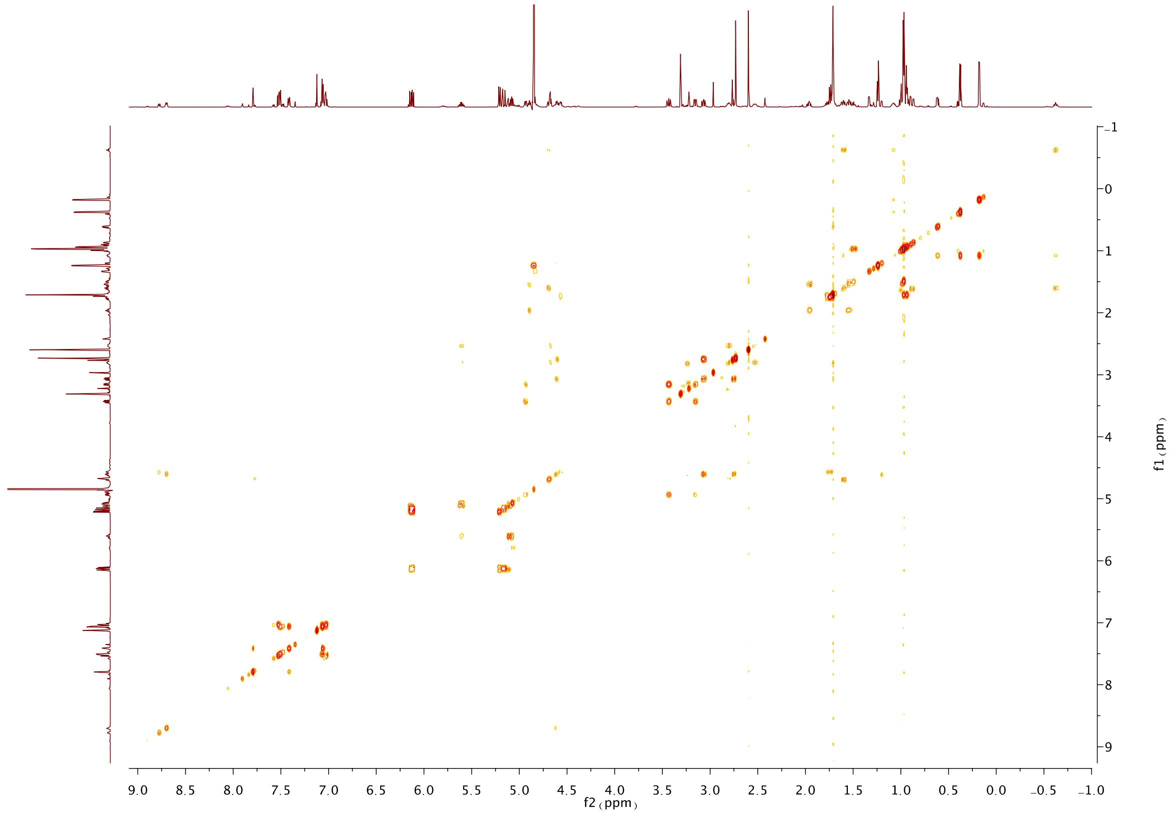

*HSQC spectrum of rufomycin analogue **9c** in CD_3OD.*

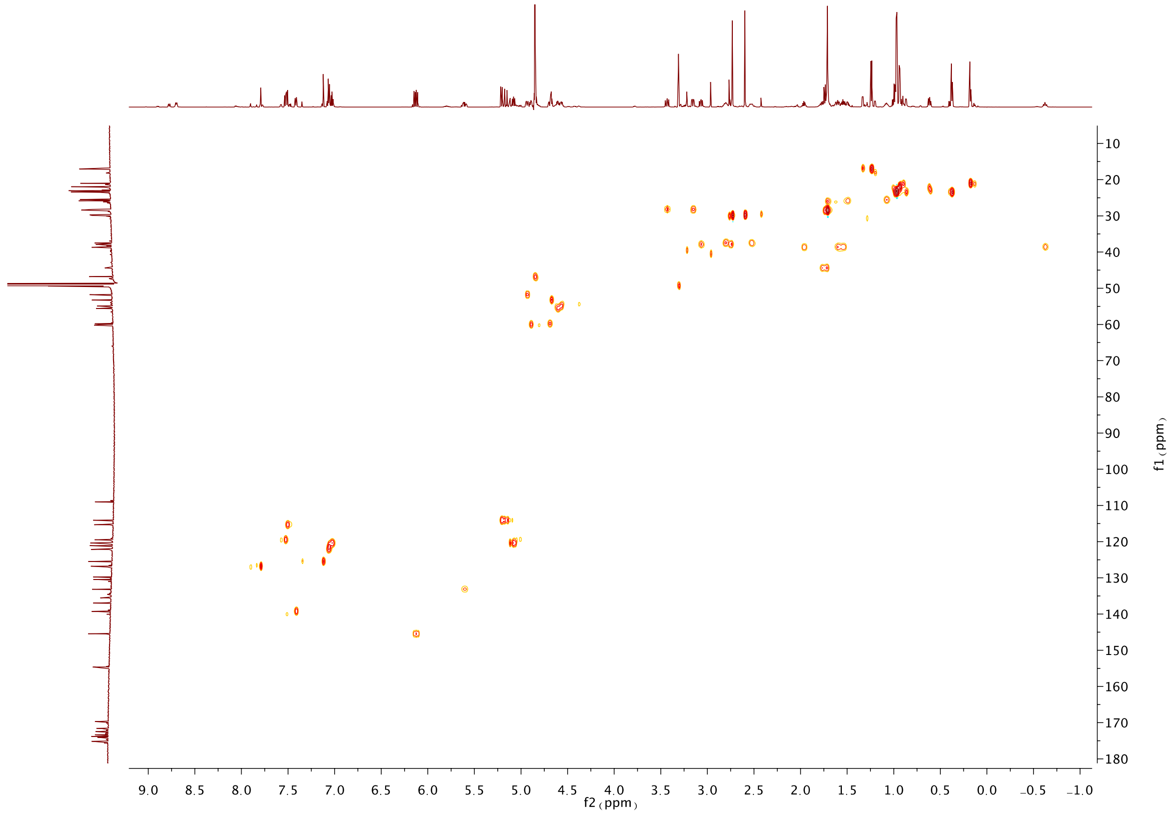

*HMBC spectrum of rufomycin analogue **9c** in CD_3OD.*

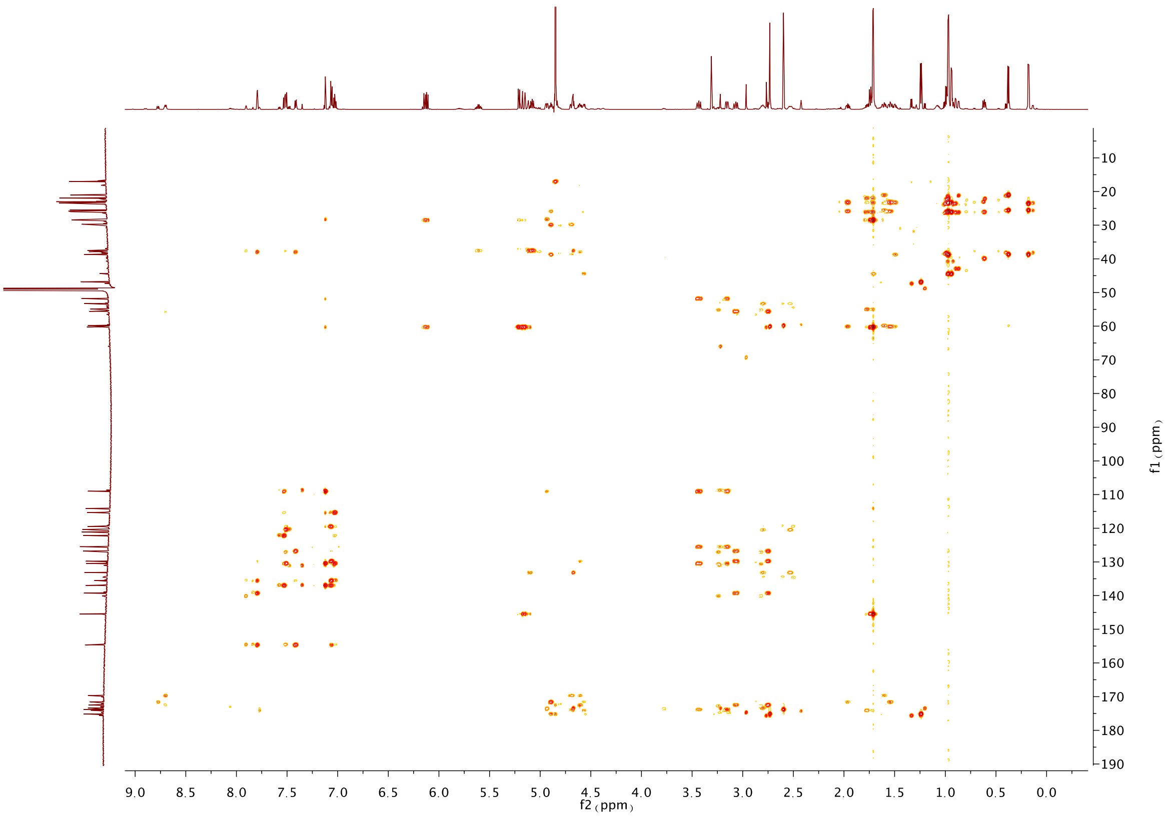

^{1}H NMR spectrum of rufomycin analogue **10c** in CD_3OD at 600 MHz.

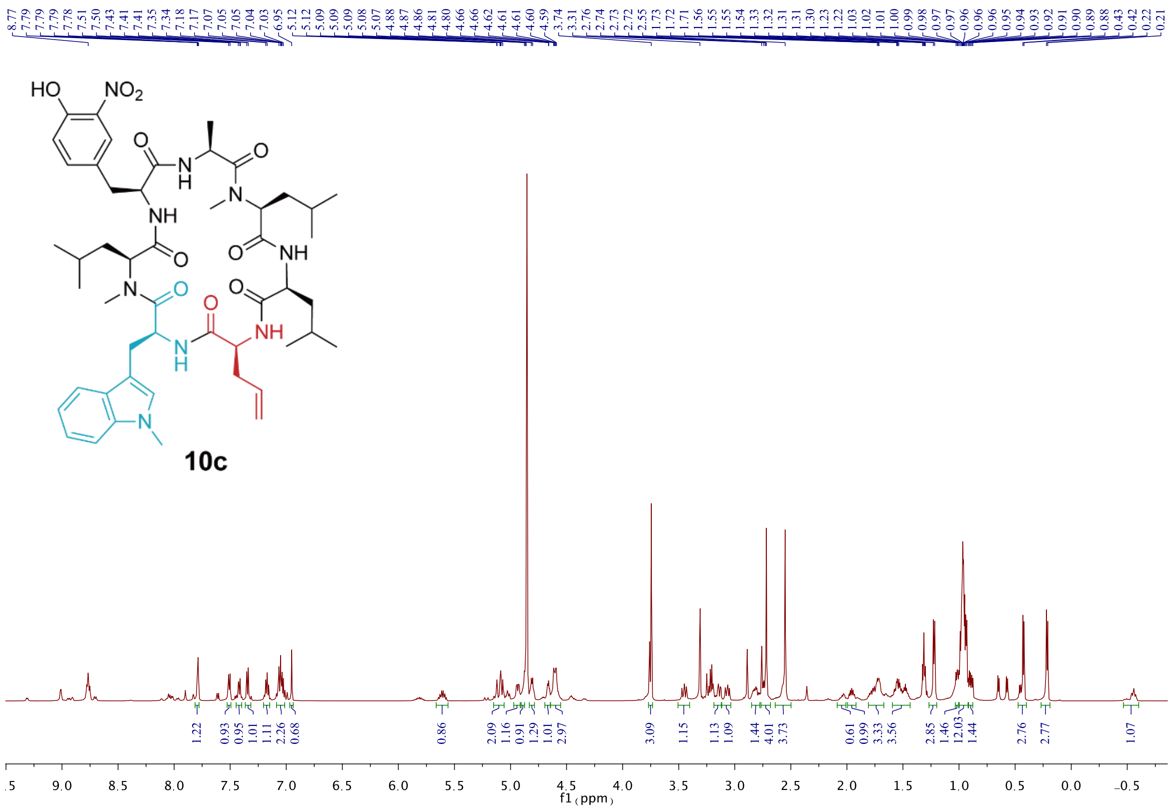

^{13}C NMR spectrum of rufomycin analogue **10c** in CD_3OD at 151 MHz.

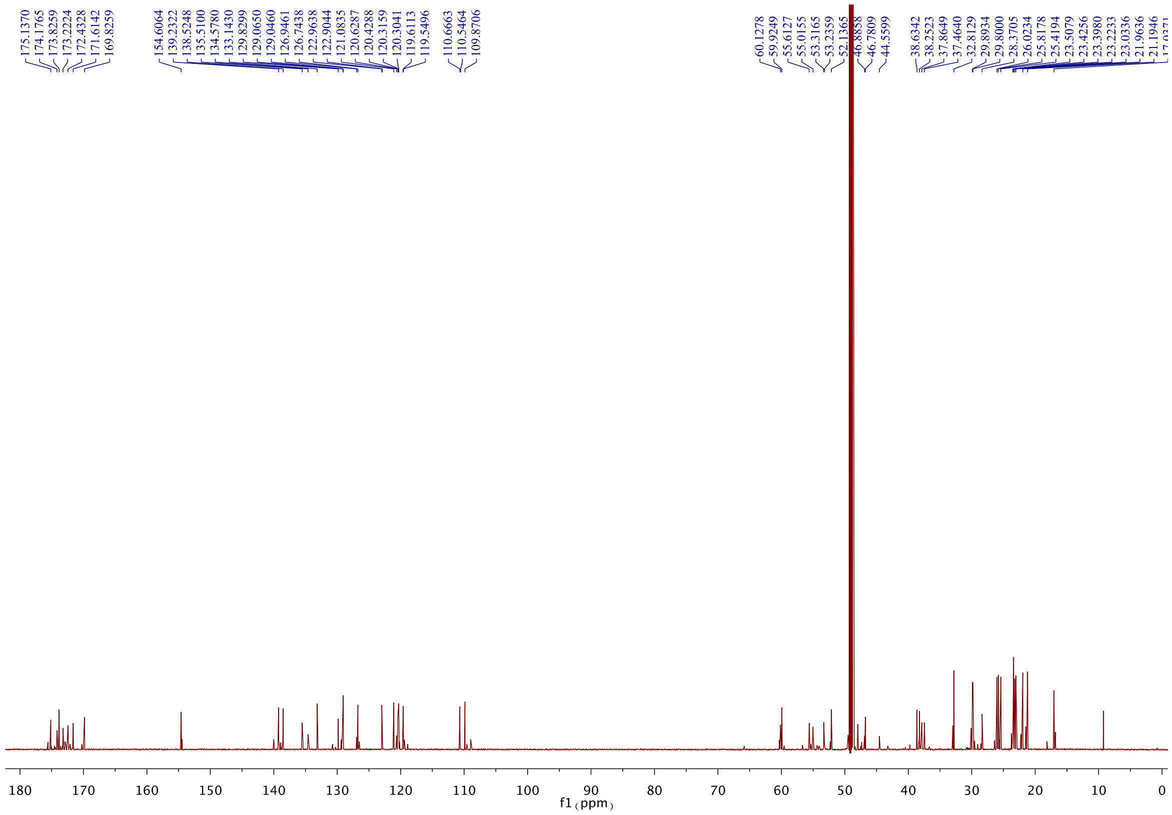

*COSY spectrum of rufomycin analogue **10c** in CD_3OD.*

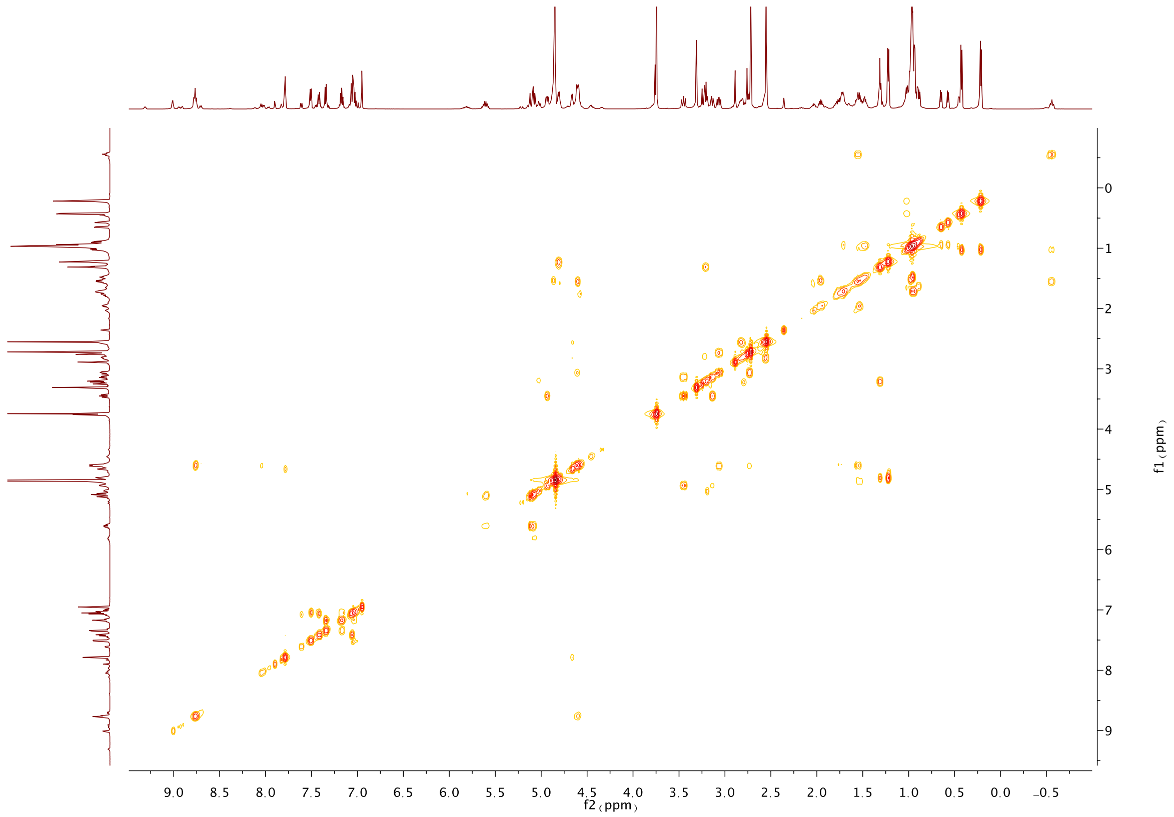

*HSQC spectrum of rufomycin analogue **10c** in CD_3OD.*

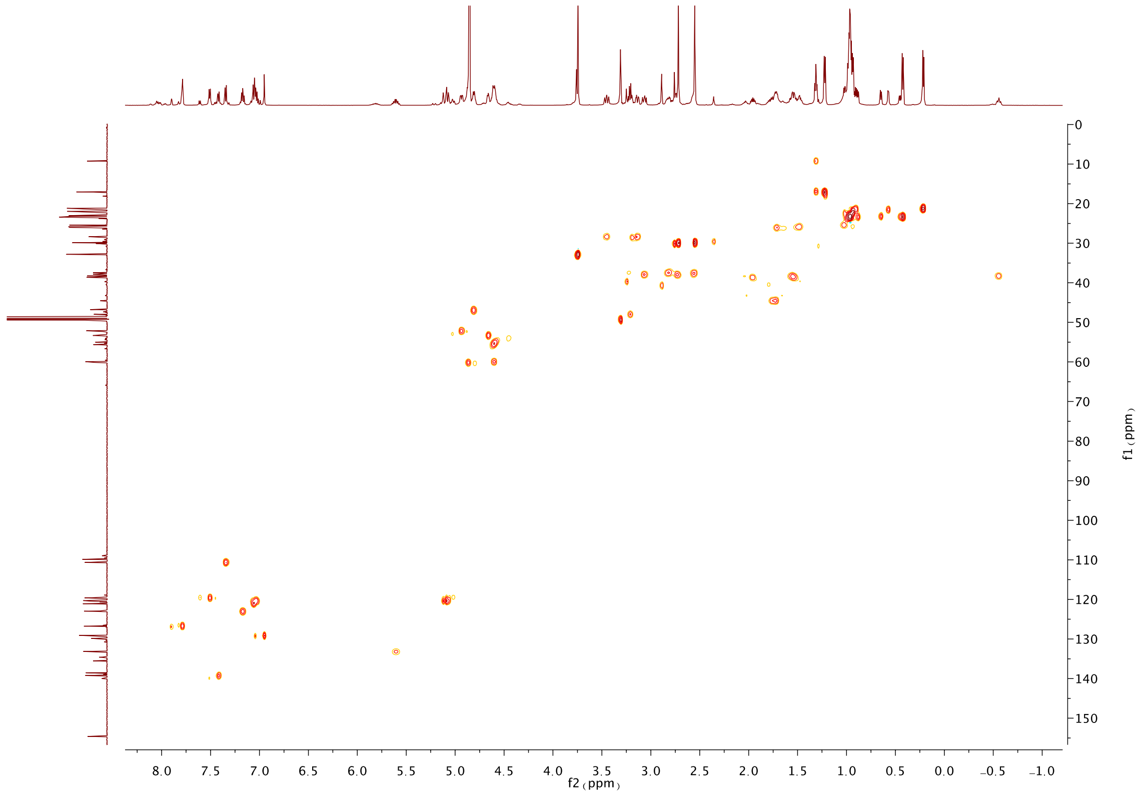

*HMBC spectrum of rufomycin analogue **10c** in CD_3OD.*

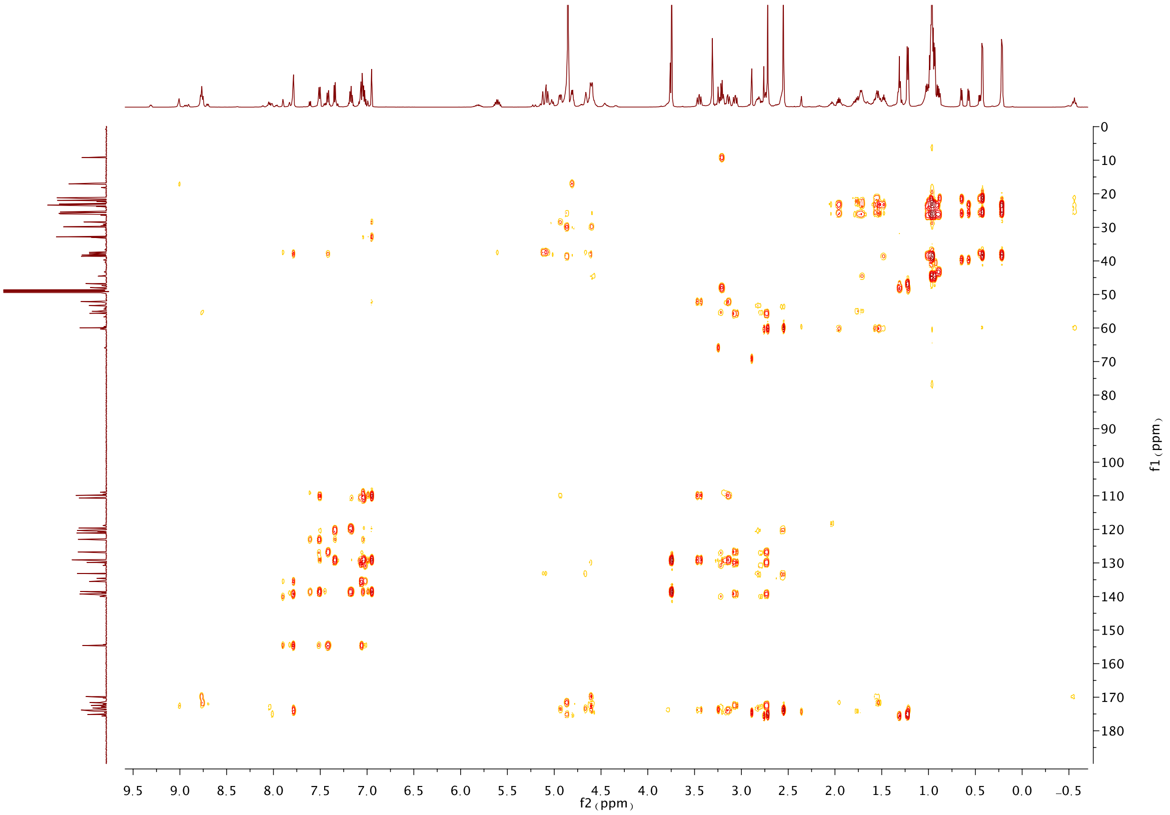

*^{1}H NMR spectrum of rufomycin analogue **11c** in CD_3OD at 700 MHz.*

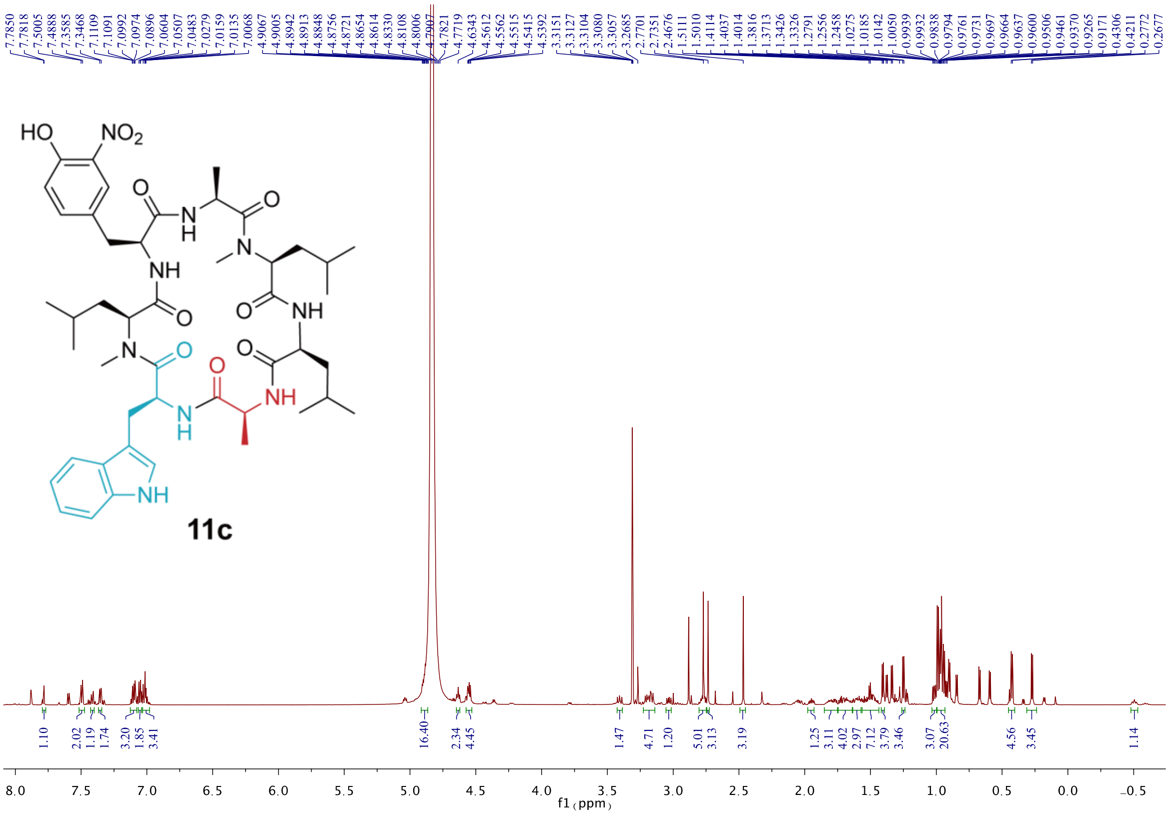

*^{13}C NMR spectrum of **11c** in CD_3OD at 176 MHz.*

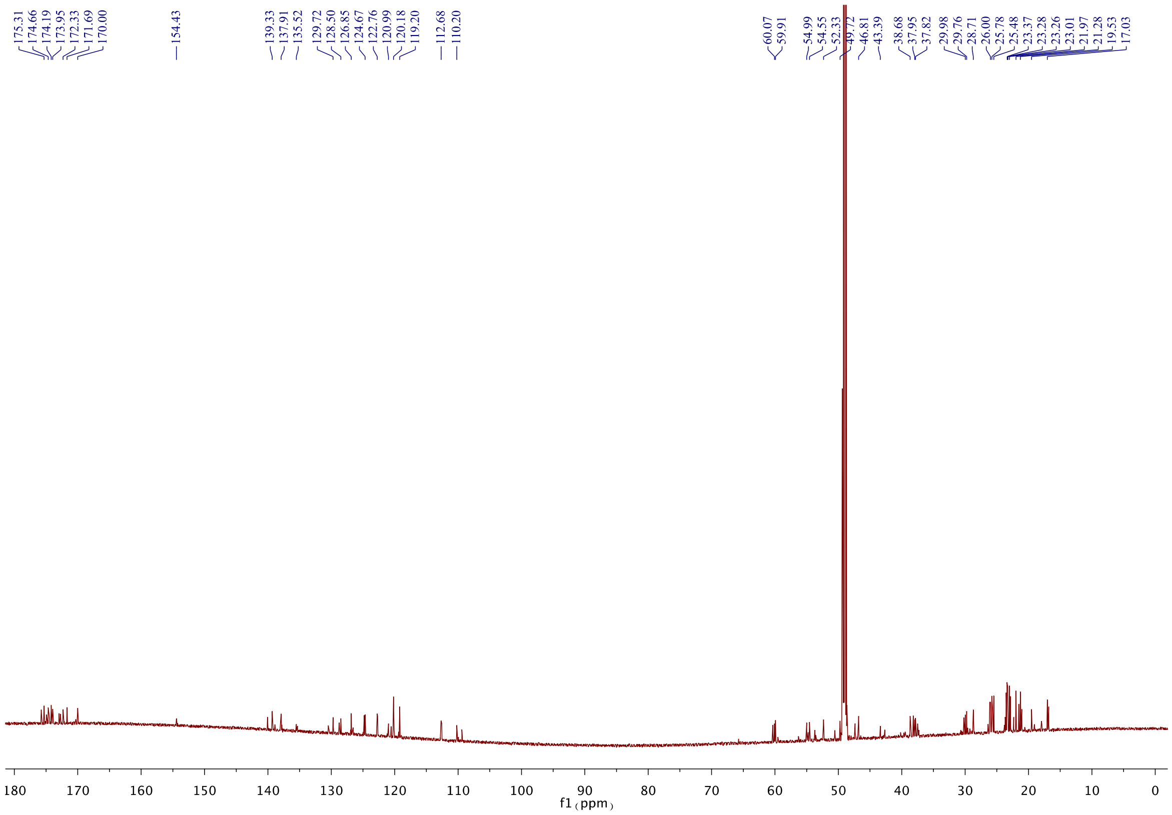

*COSY spectrum of rufomycin analogue **11c** in CD_3OD.*

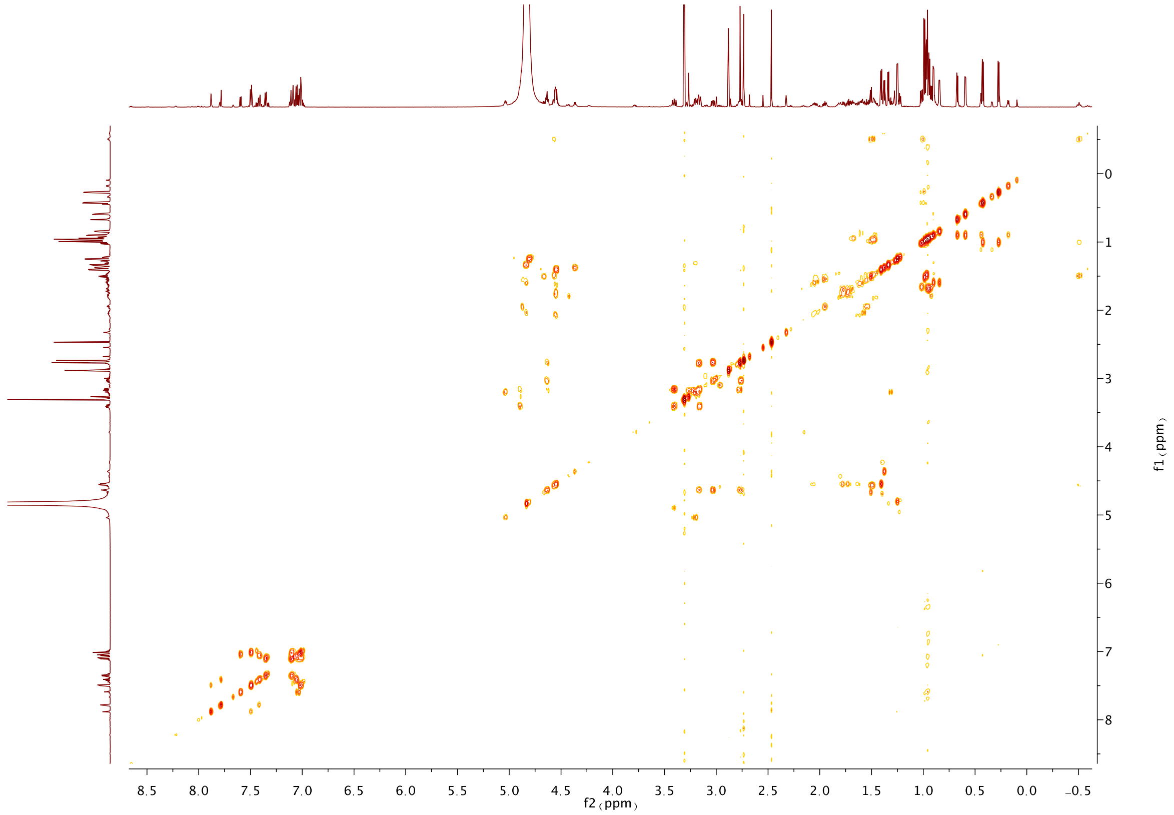

*HSQC spectrum of rufomycin analogue **11c** in CD_3OD.*

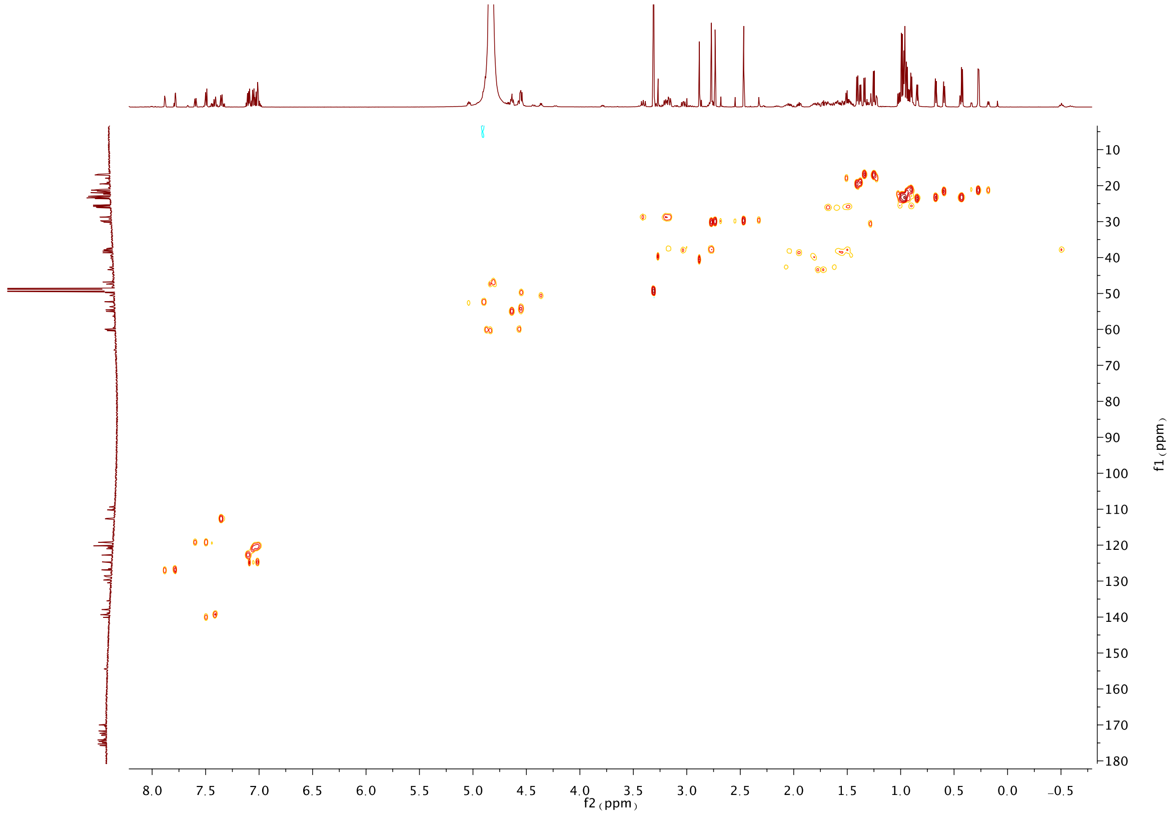

*HMBC spectrum of rufomycin analogue **11c** in CD_3OD.*

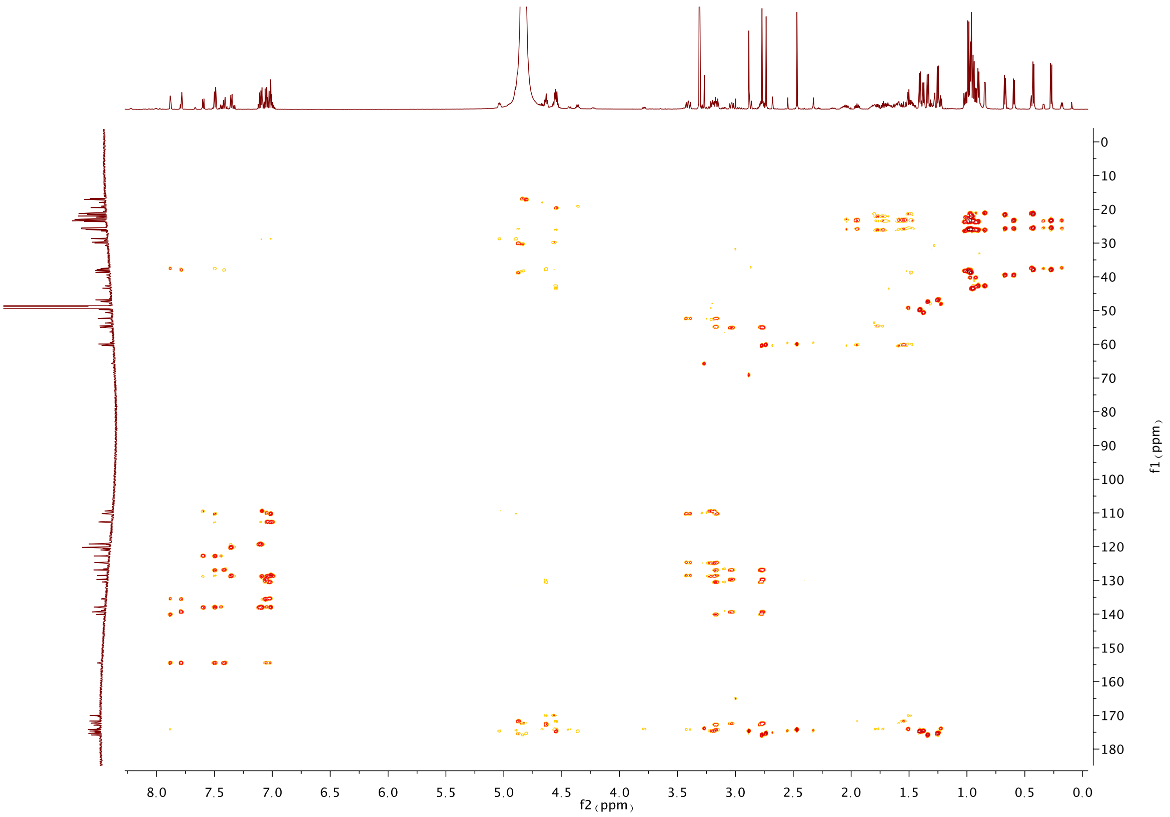

^{1}H NMR spectrum of rufomycin analogue **12c** in CD_3OD at 700 MHz.

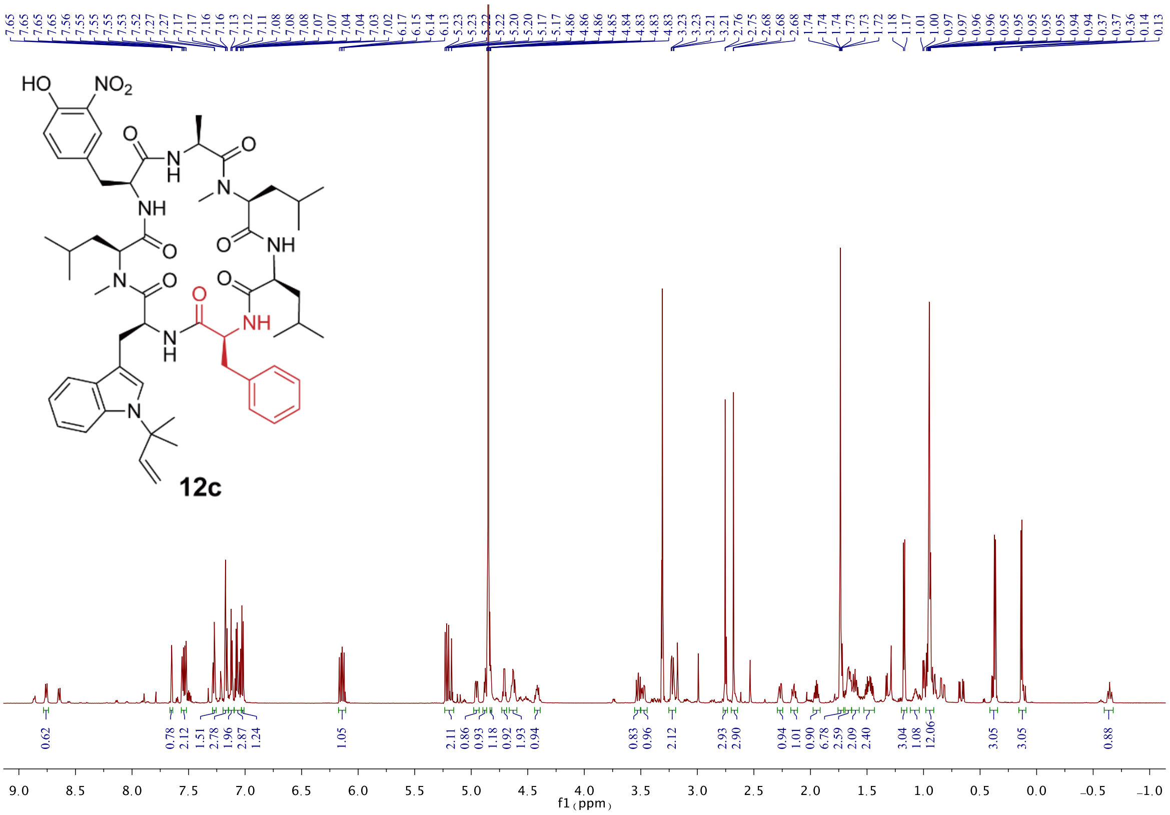

^{13}C NMR spectrum of rufomycin analogue **12c** in CD_3OD at 176 MHz.

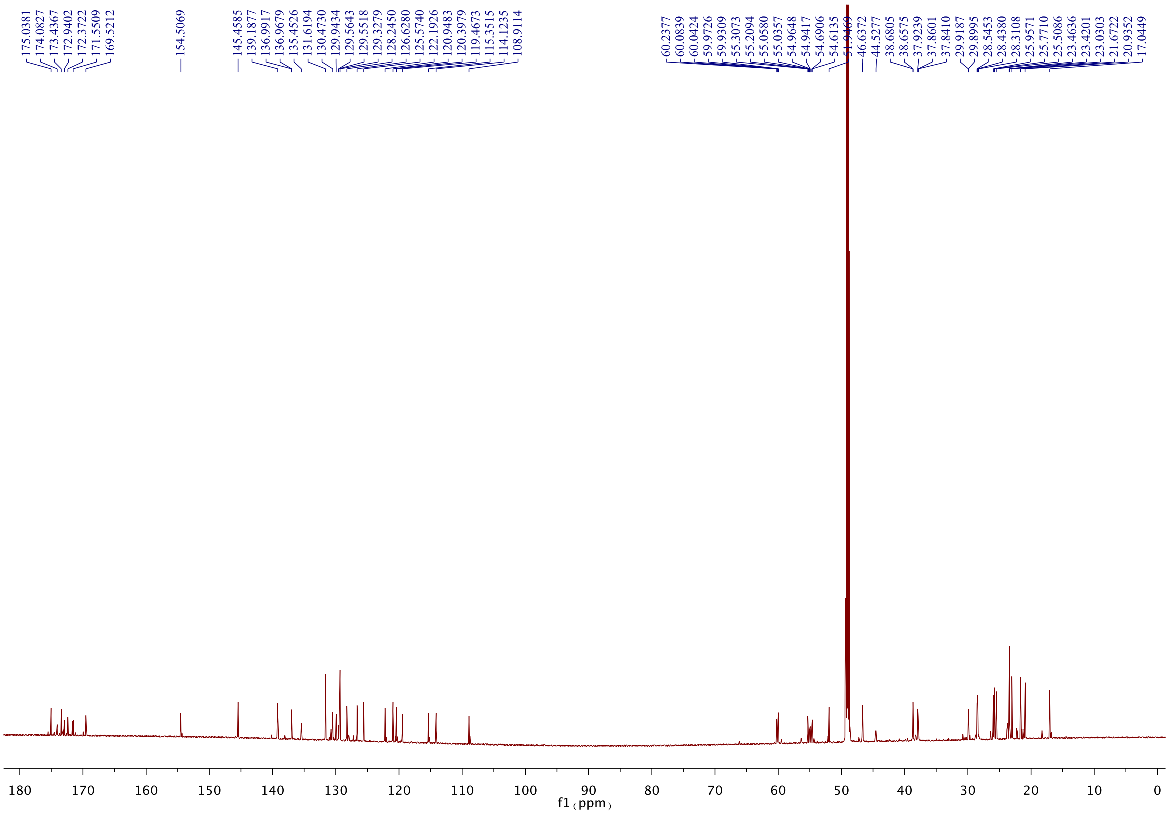

*COSY spectrum of rufomycin analogue **12c** in CD_3OD.*

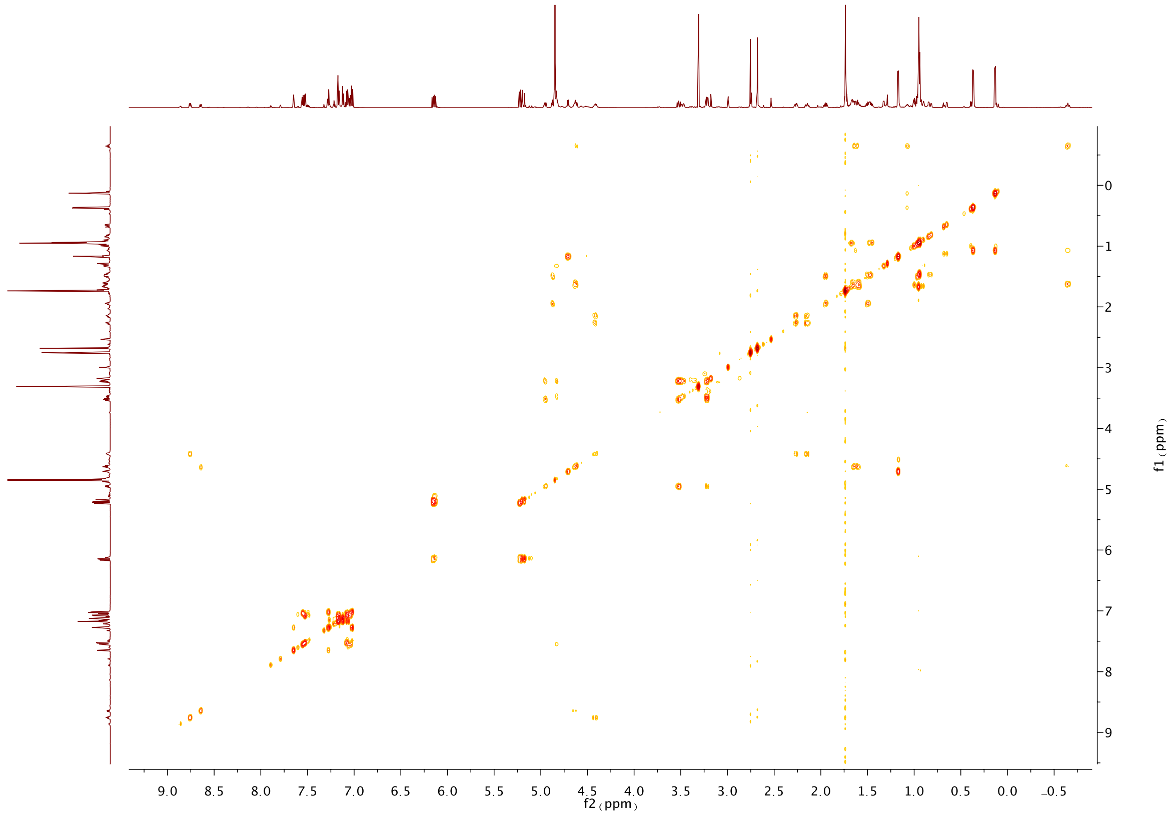

*HSQC spectrum of rufomycin analogue **12c** in CD_3OD.*

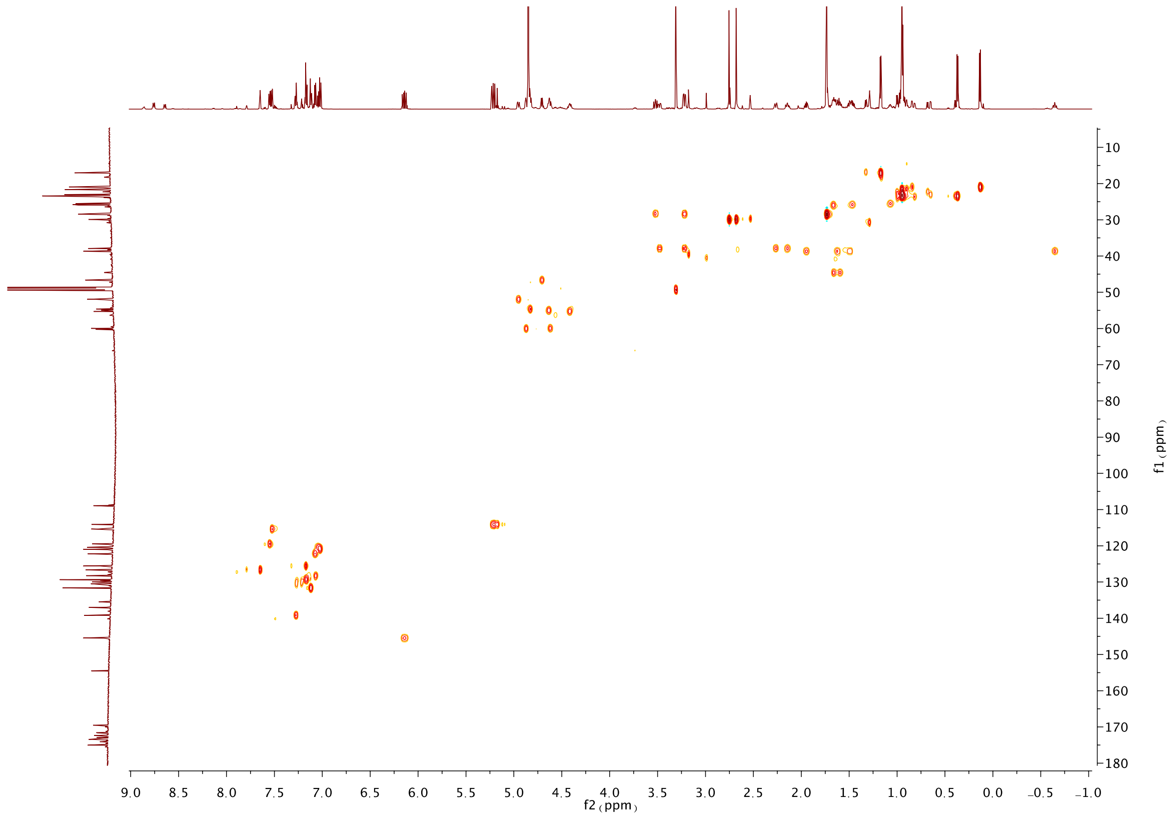

HMBC spectrum of rufomycin analogue ***12c*** *in CD_3OD.*

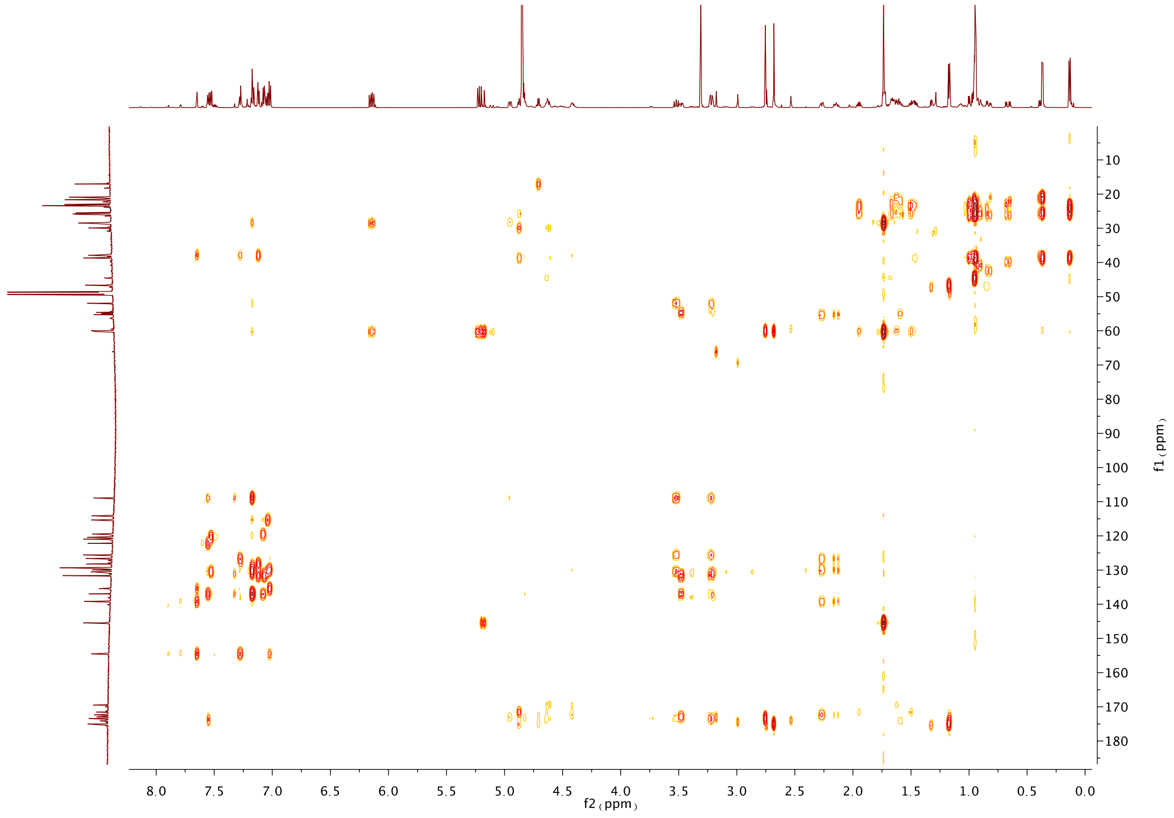

^{1}H NMR spectrum of rufomycin analogue ***13c*** *in CD_3OD at 700 MHz.*

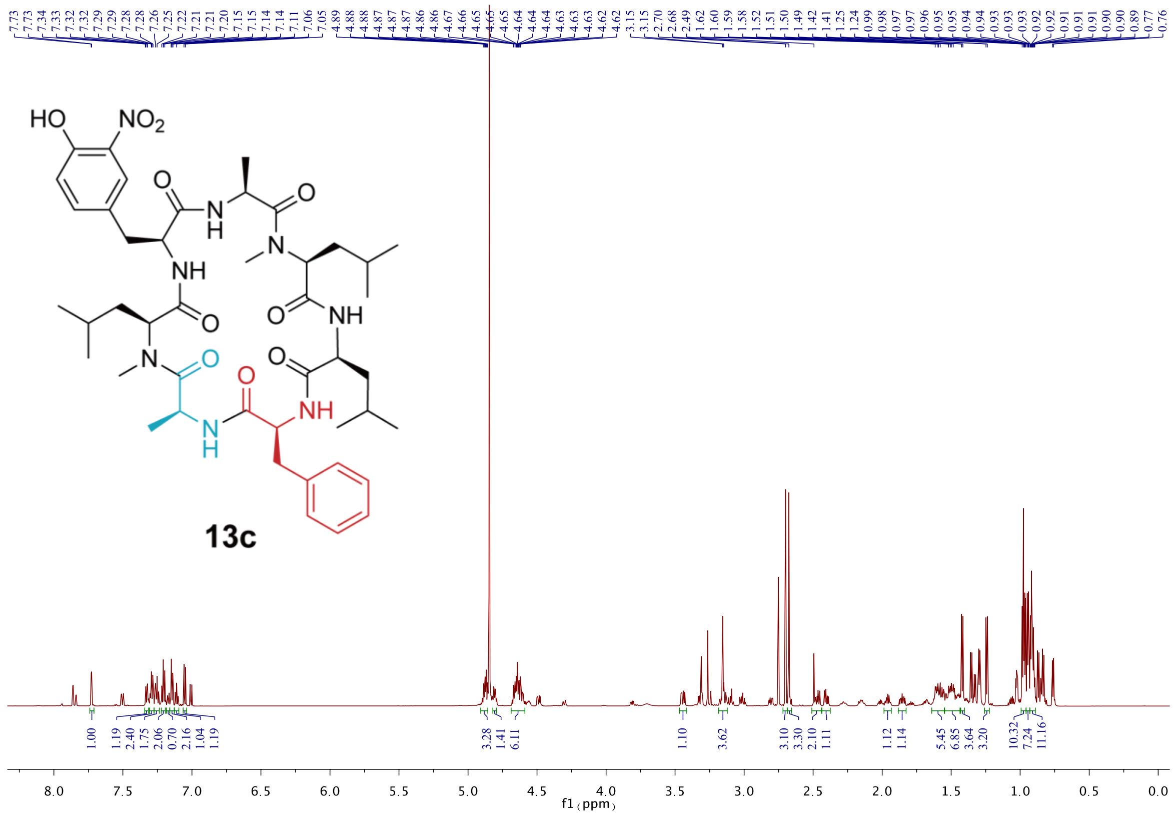

^{13}C NMR spectrum of rufomycin analogue ***13c*** *in CD_3OD at 176 MHz.*

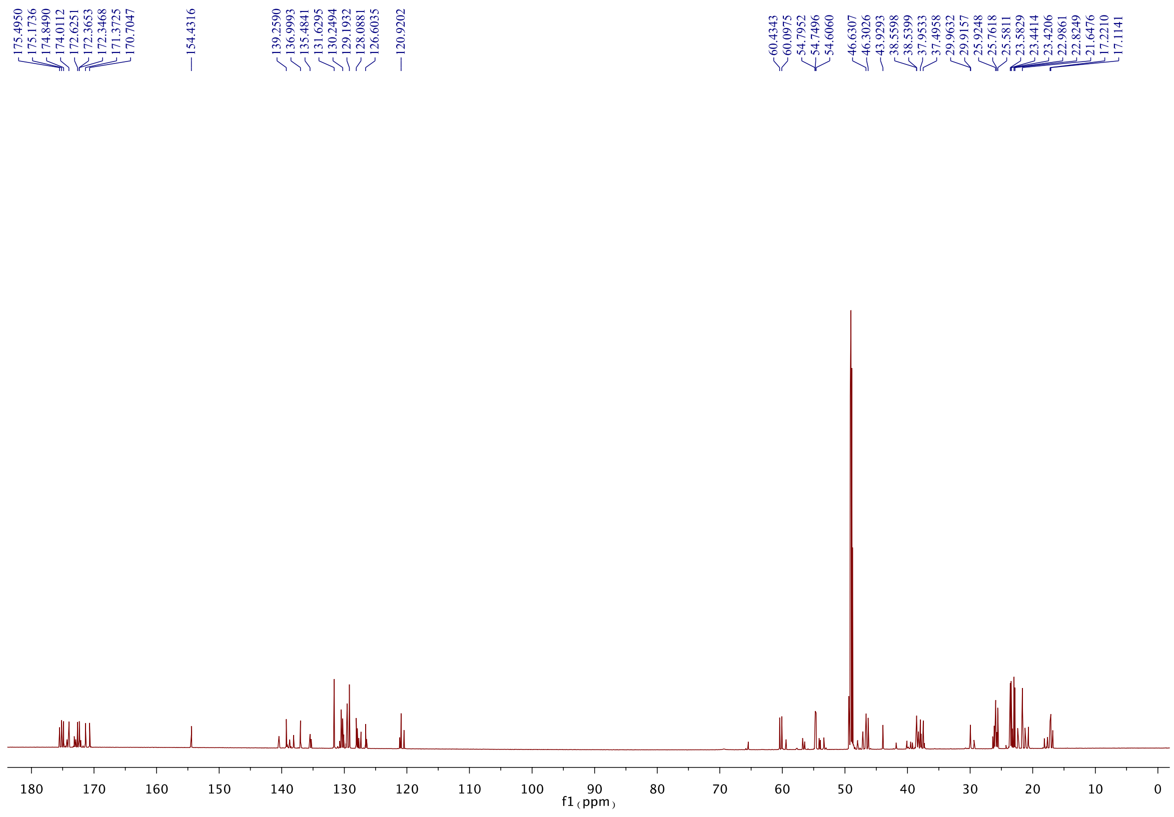

COSY spectrum of rufomycin analogue ***13c*** *in* CD_3OD*.*

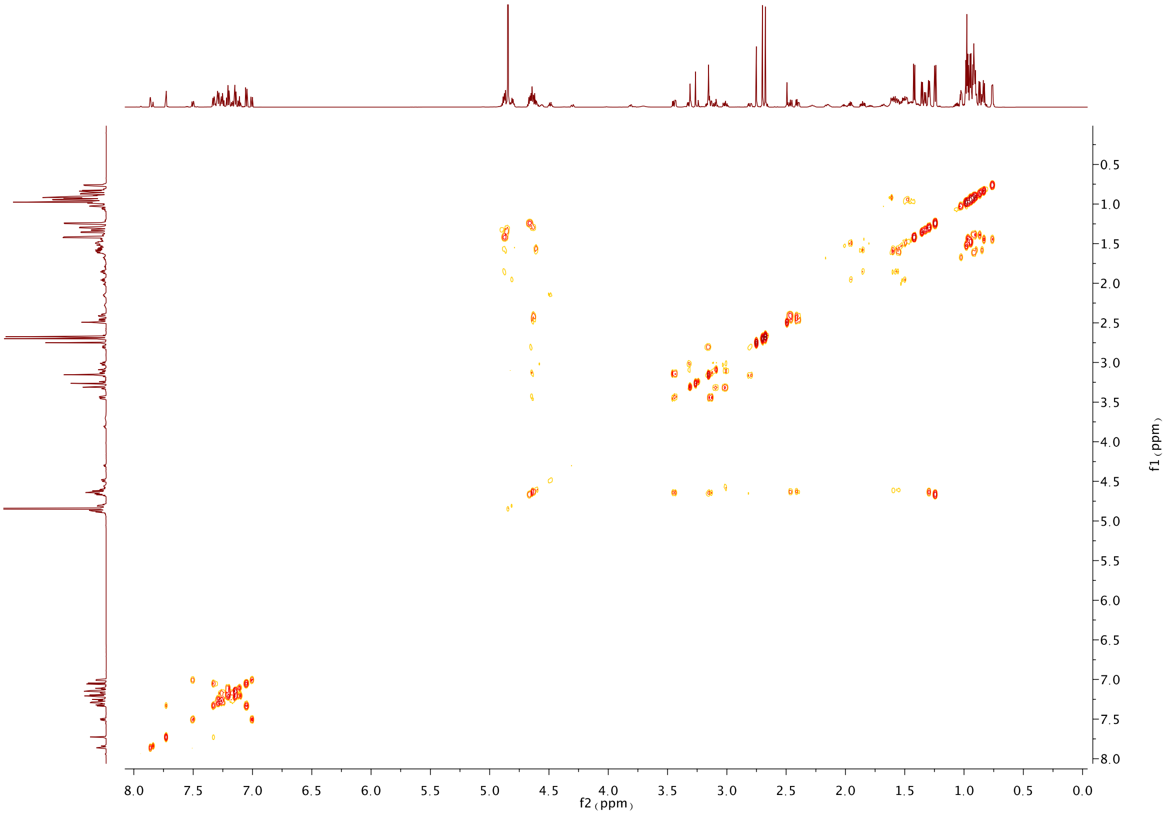

HSQC spectrum of rufomycin analogue ***13c*** *in* CD_3OD*.*

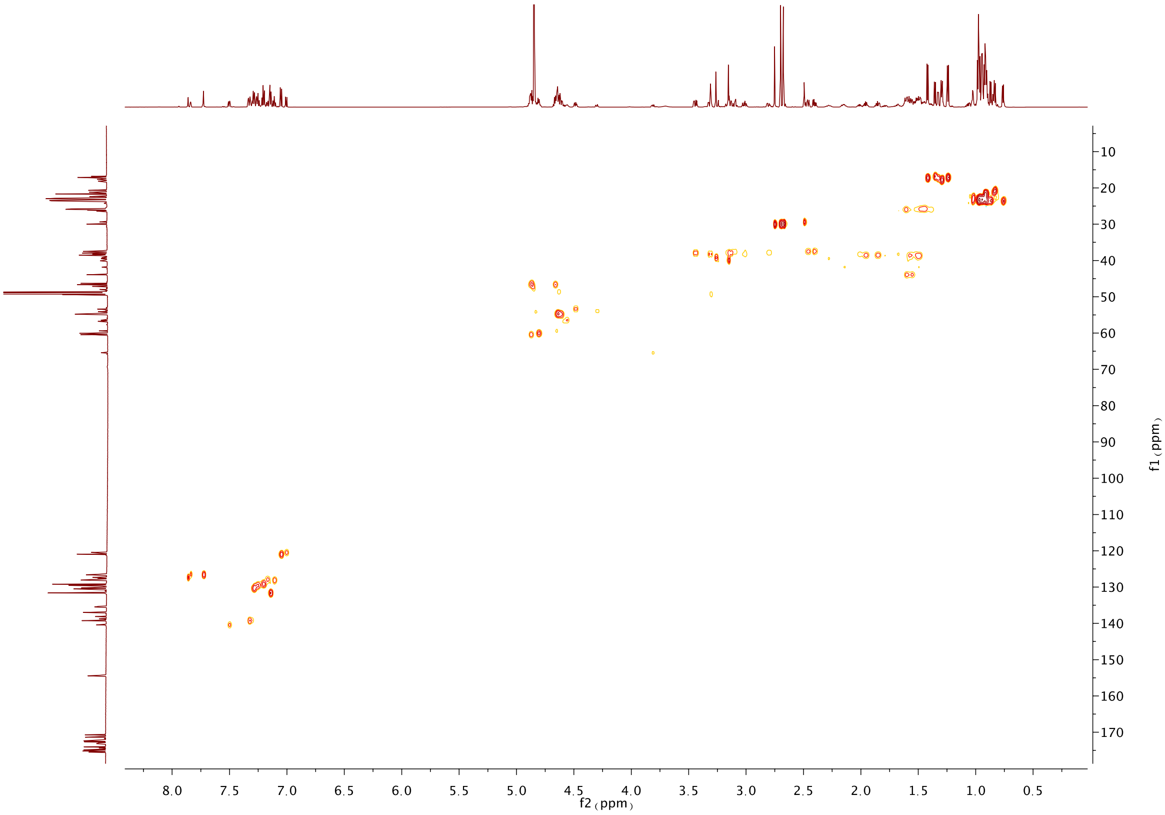

*HMBC spectrum of rufomycin analogue **13c** in CD_3OD.*

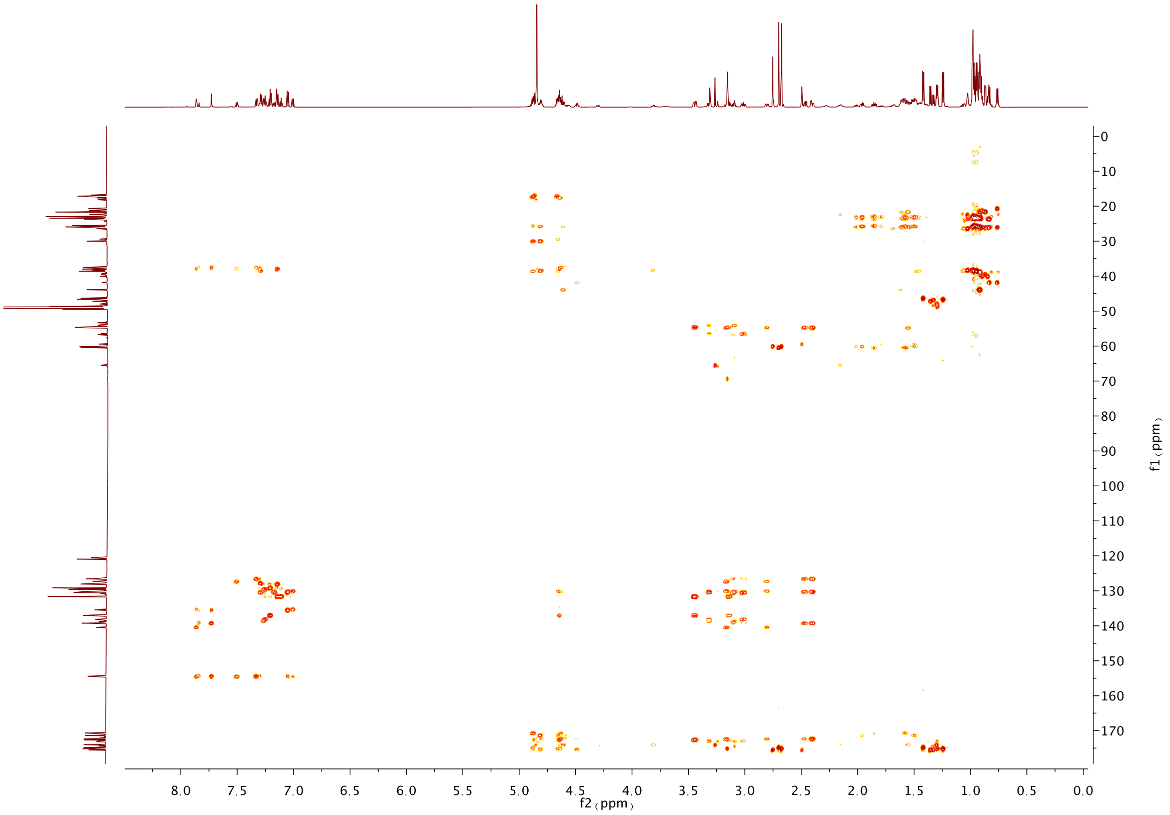

^{1}H NMR spectrum of rufomycin analogue **17c** in $(CD_3)_2SO$ at 400 MHz.

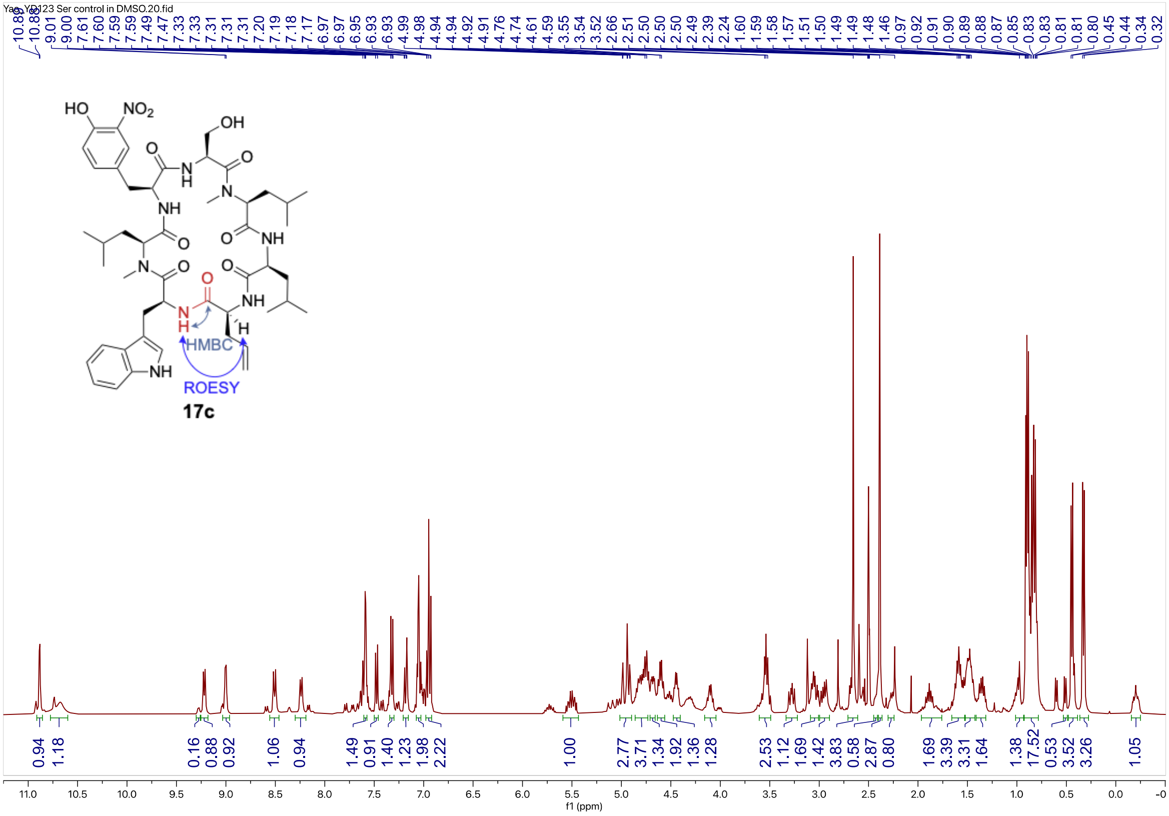

^{13}C NMR spectrum of rufomycin analogue **17c** in $(CD_3)_2SO$ at 101 MHz.

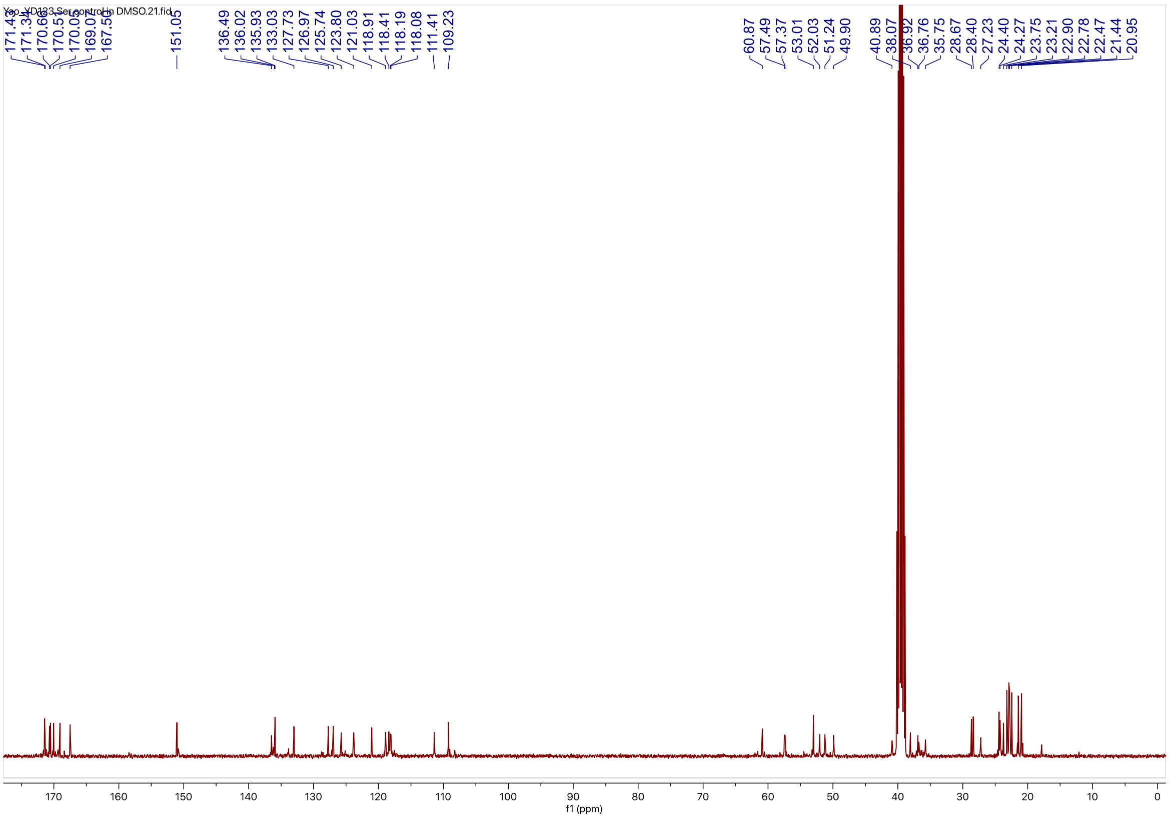

*COSY spectrum of rufomycin analogue **17c** in $(CD_3)_2SO$ at 400 MHz.*

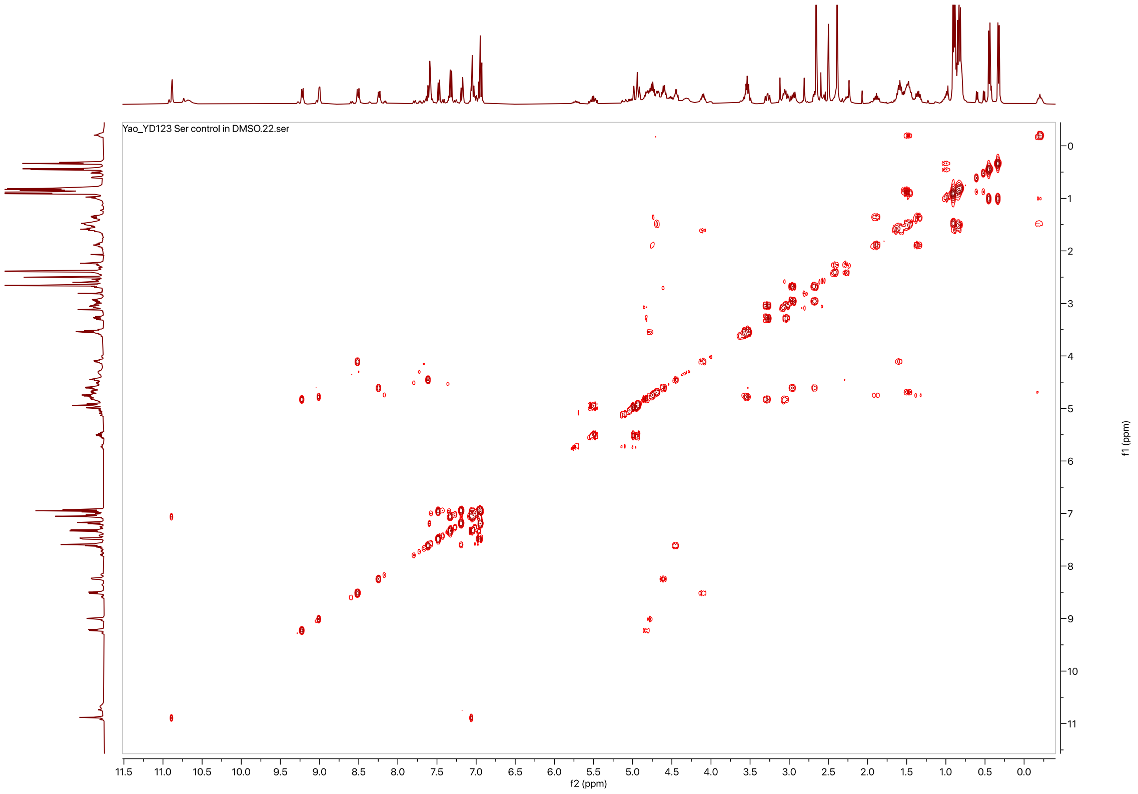

*HSQC spectrum of rufomycin analogue **17c** in $(CD_3)_2SO$.*

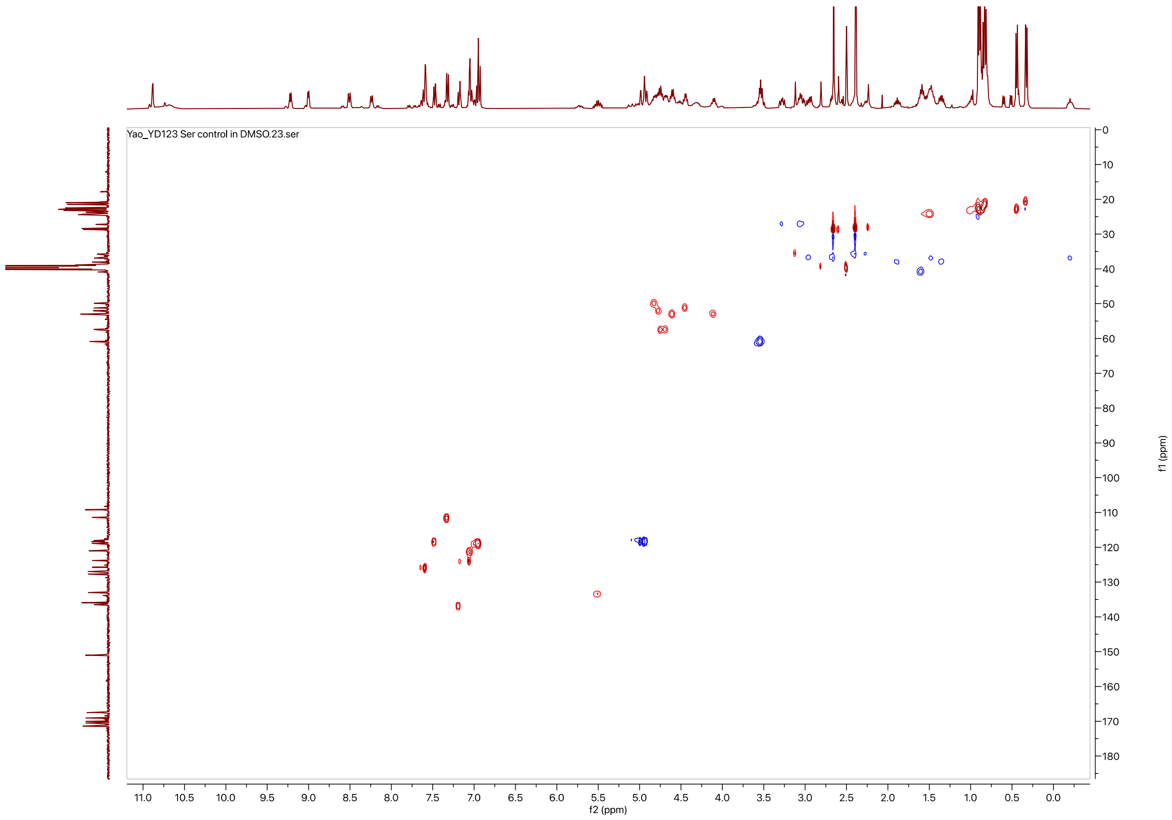

HMBC spectrum of rufomycin analogue ***17c*** *in* $(CD_3)_2SO$.

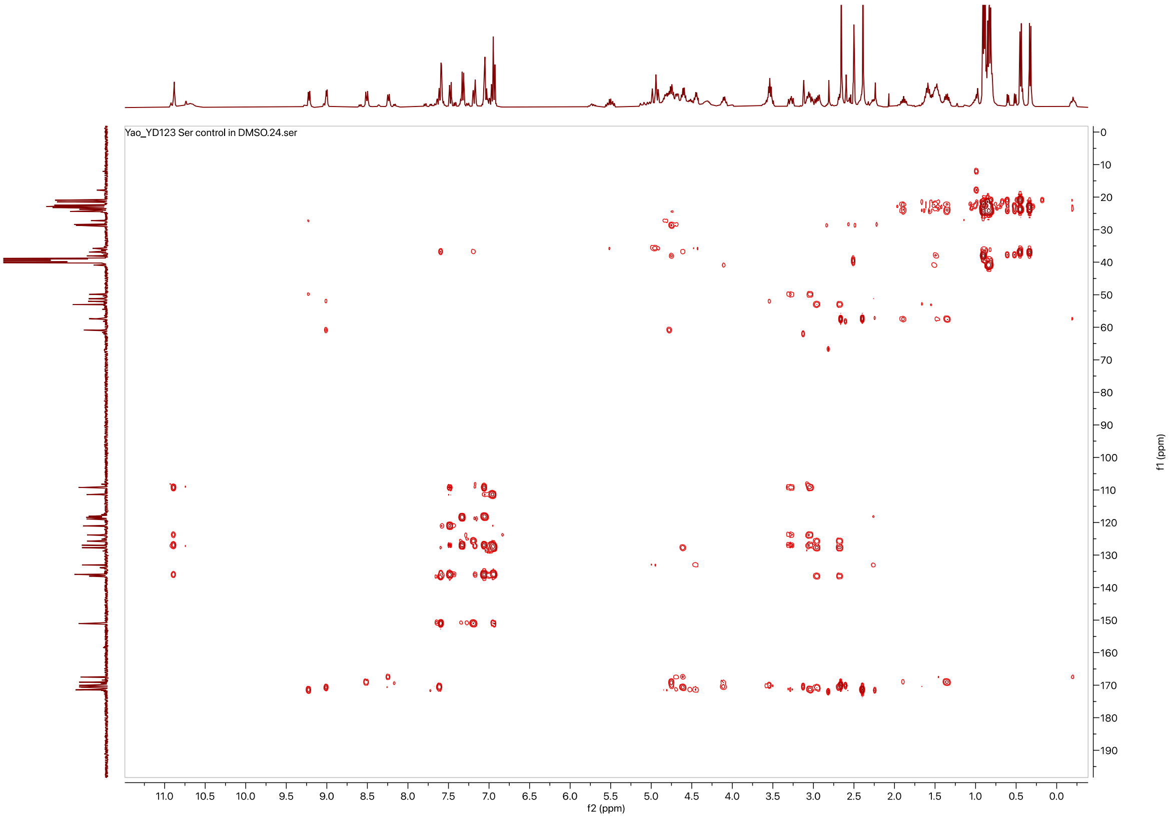

ROESY spectrum of rufomycin analogue ***17c*** *in* $(CD_3)_2SO$ *at 400 MHz.*

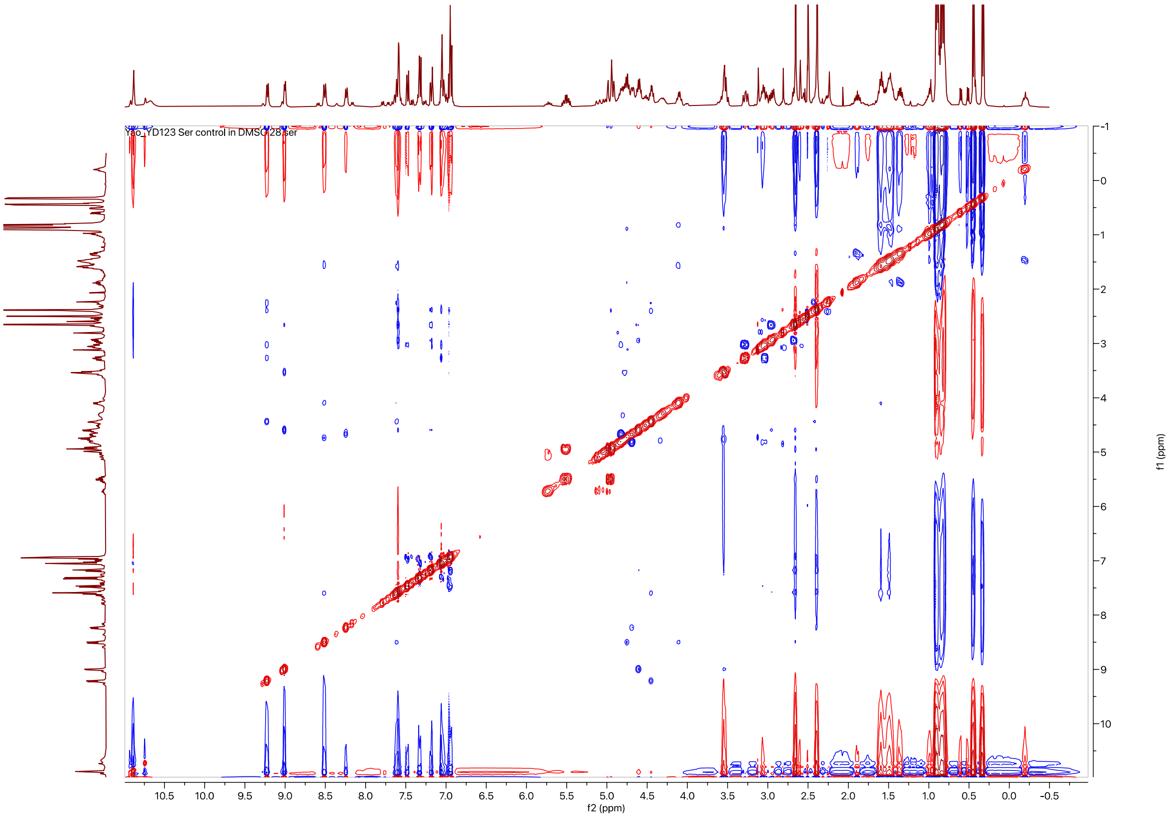

^{1}H NMR spectrum of rufomycin analogue **18c** in $(CD_3)_2SO$ at 400 MHz.

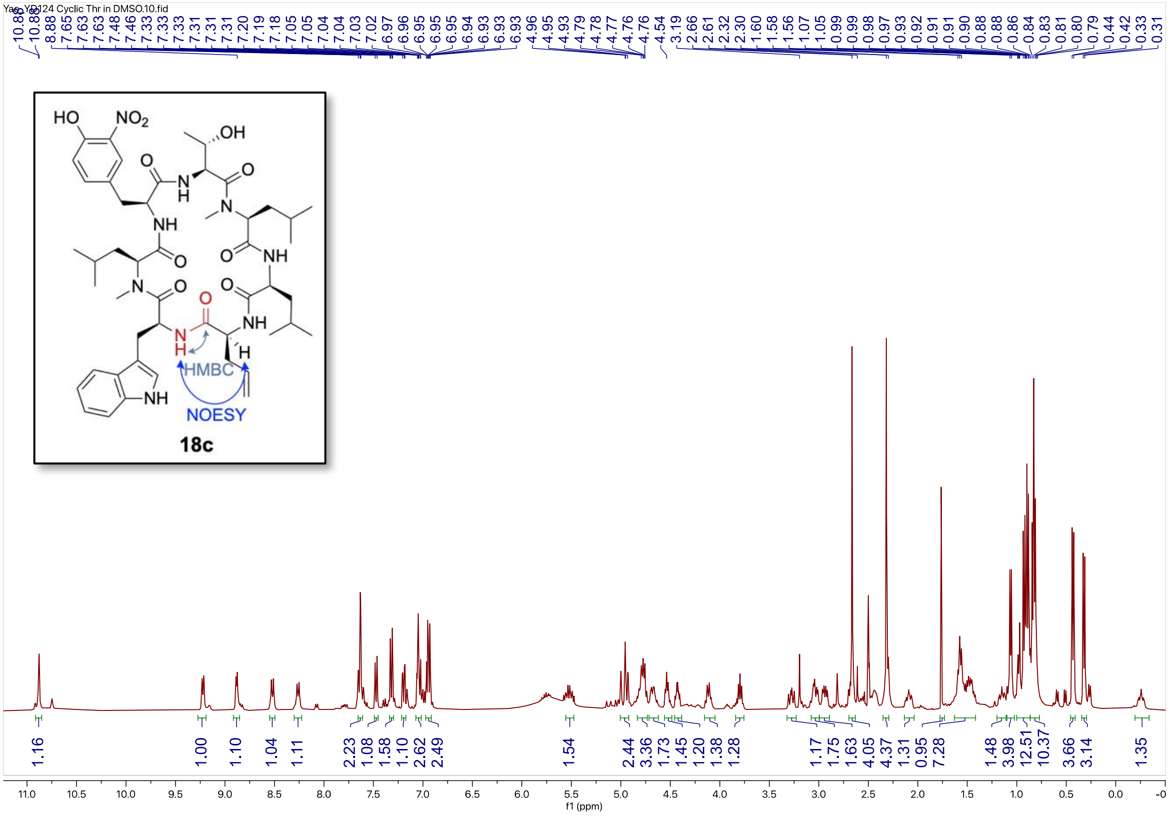

^{13}C NMR spectrum of rufomycin analogue **18c** in $(CD_3)_2SO$ at 101 MHz.

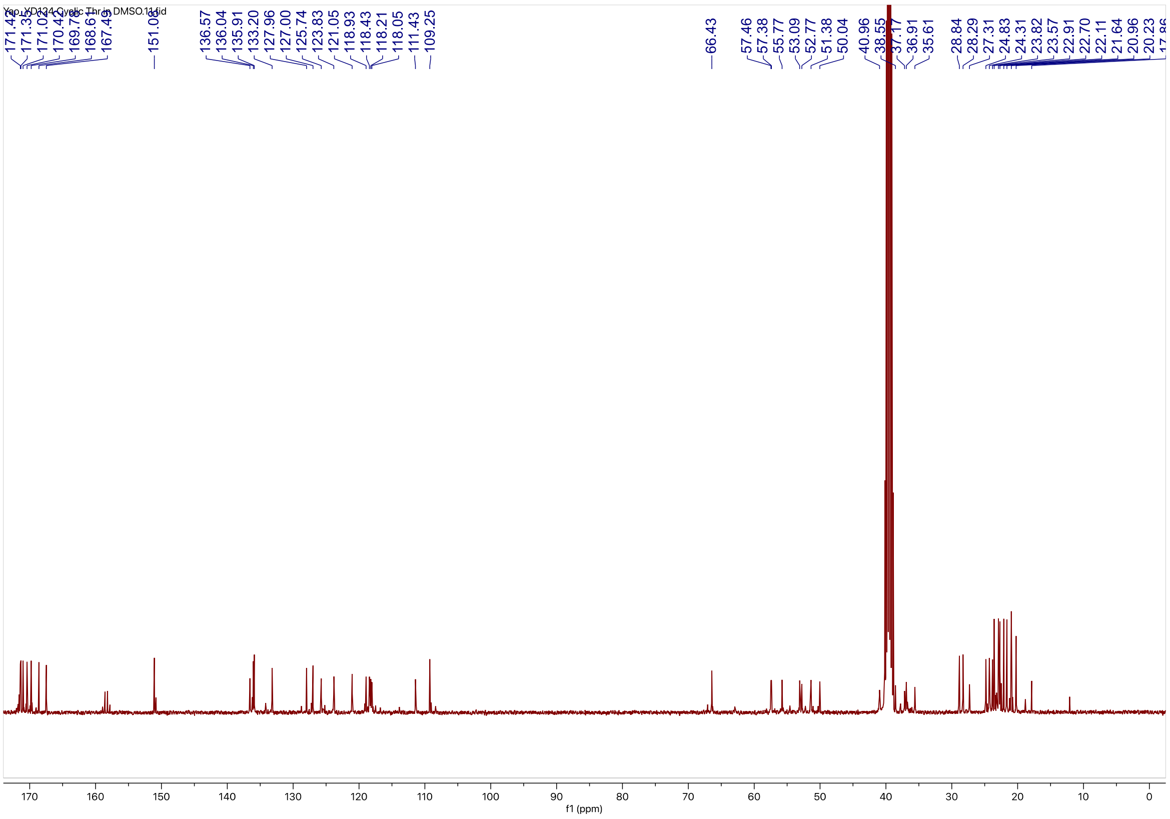

COSY spectrum of rufomycin analogue ***18c*** *in* $(CD_3)_2SO$ *at 400 MHz.*

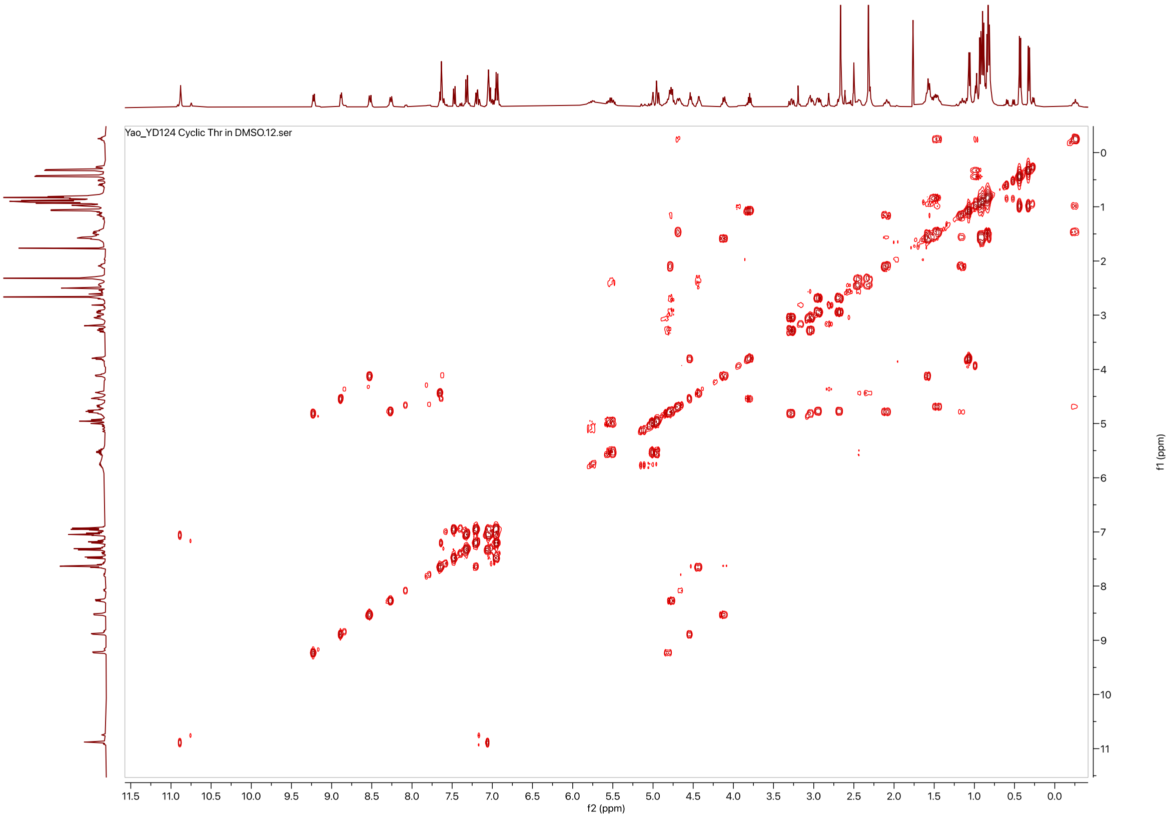

HSQC spectrum of rufomycin analogue ***18c*** *in* $(CD_3)_2SO$*.*

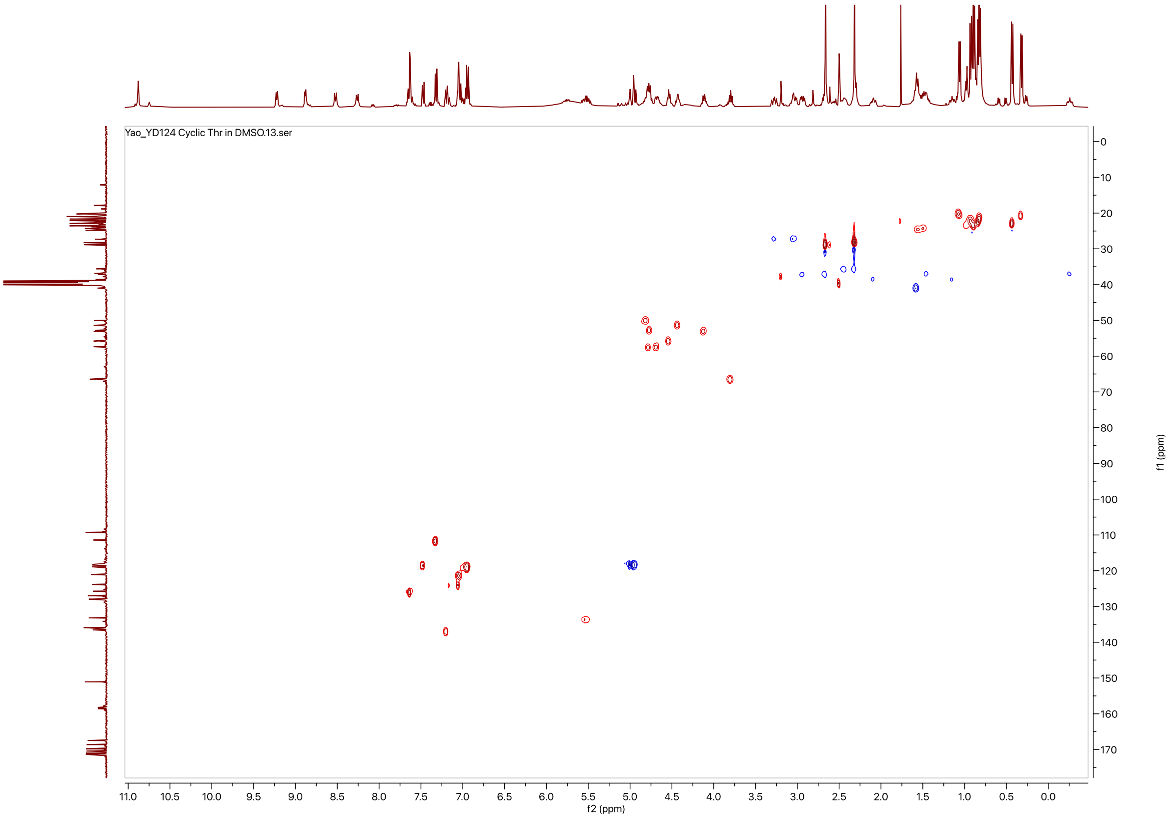

*HMBC spectrum of rufomycin analogue **18c** in $(CD_3)_2SO$.*

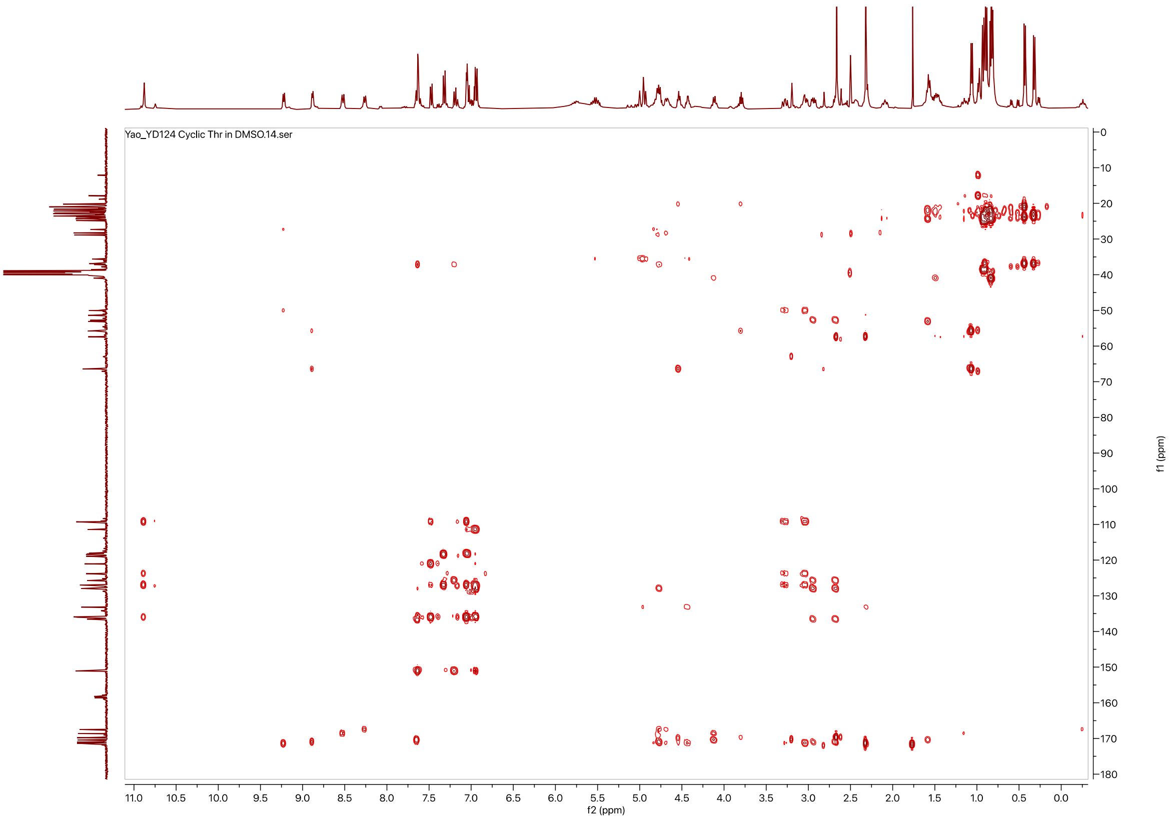

*NOESY spectrum of rufomycin analogue **18c** in $(CD_3)_2SO$ at 400 MHz.*

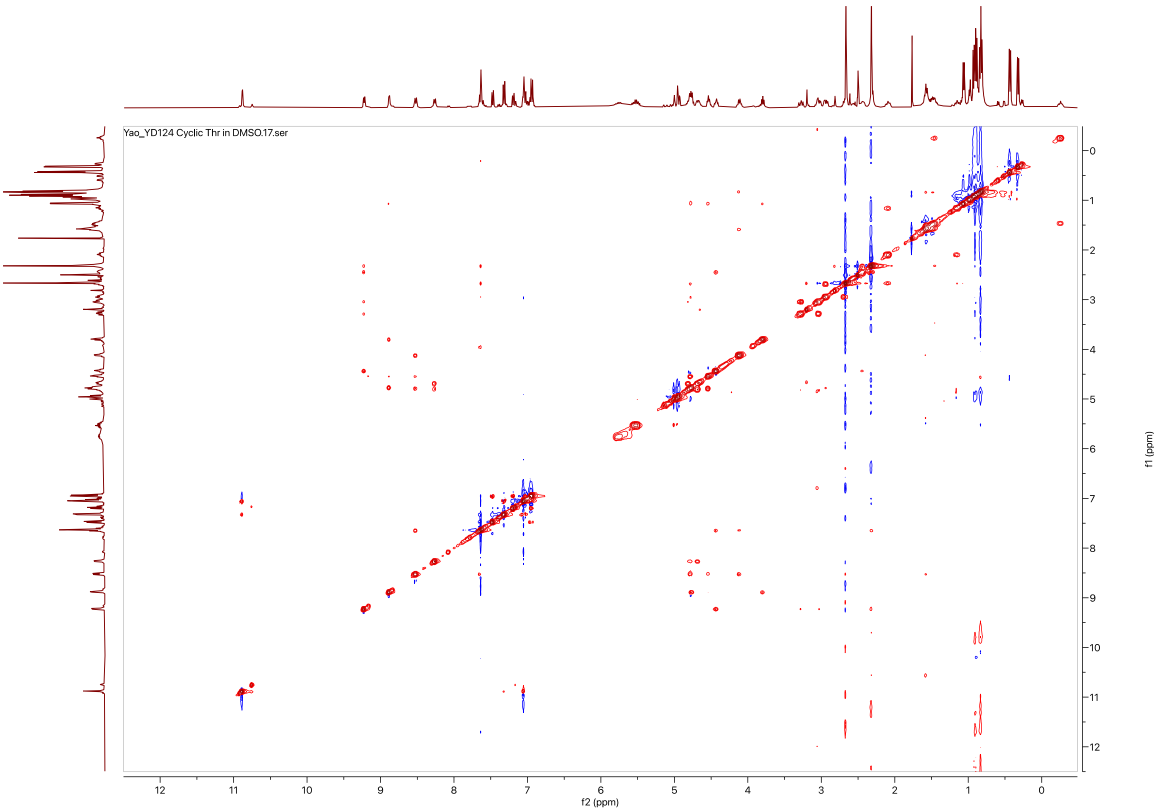

^{1}H NMR spectrum of tyrocidine **25c** in CD_3OD at 700 MHz.

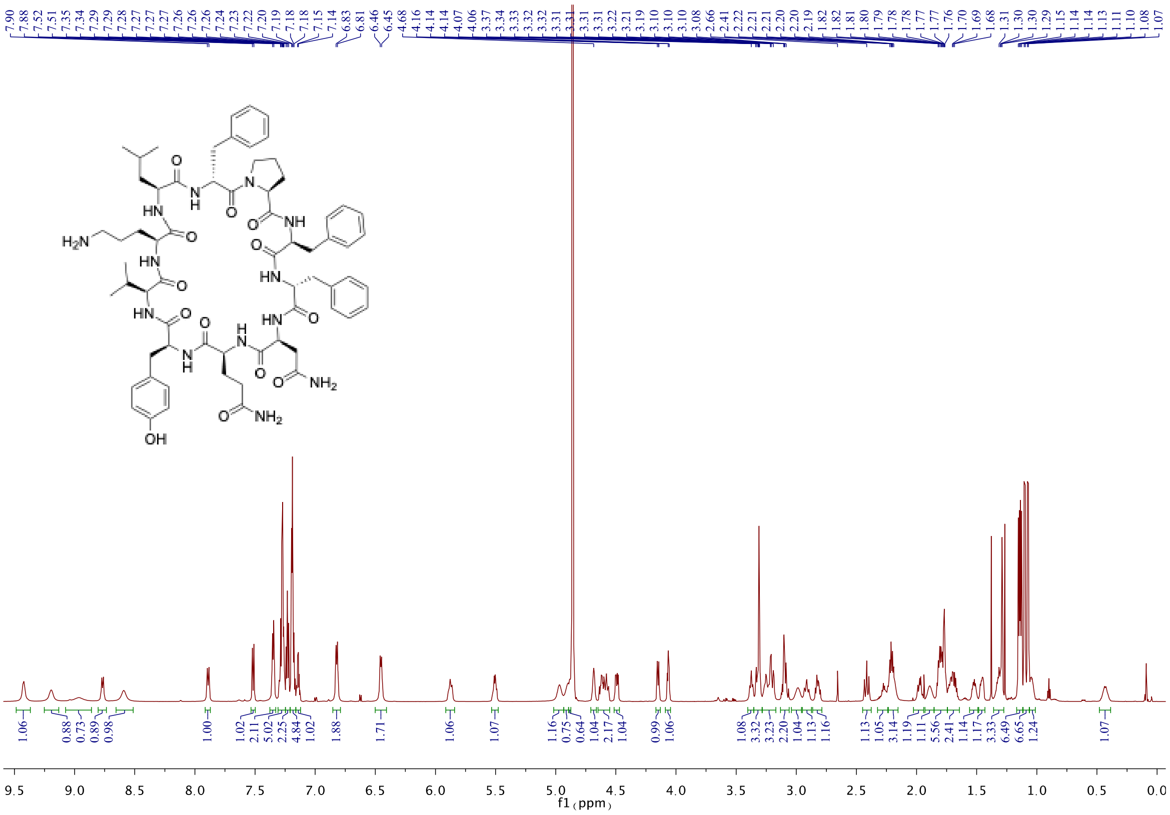

^{13}C NMR spectrum of tyrocidine **25c** in CD_3OD at 176 MHz.

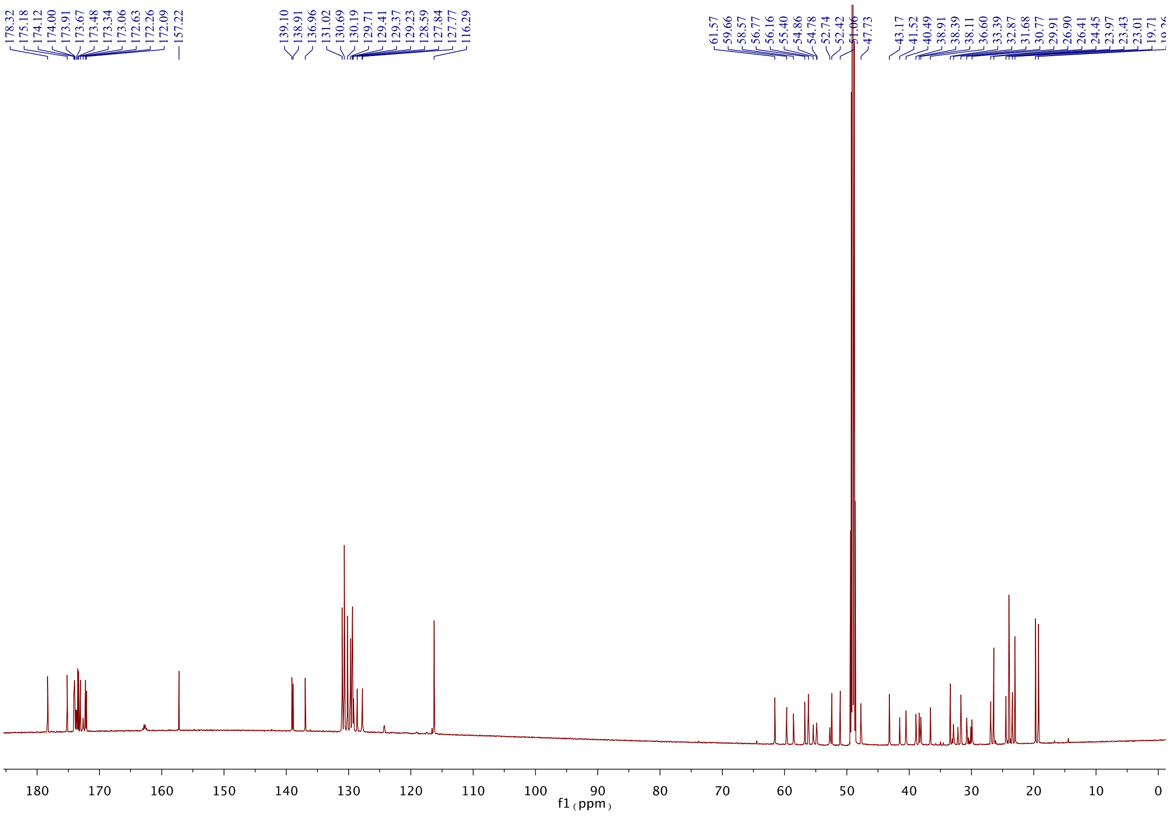

*COSY spectrum of tyrocidine **25c** in CD_3OD at 700 MHz.*

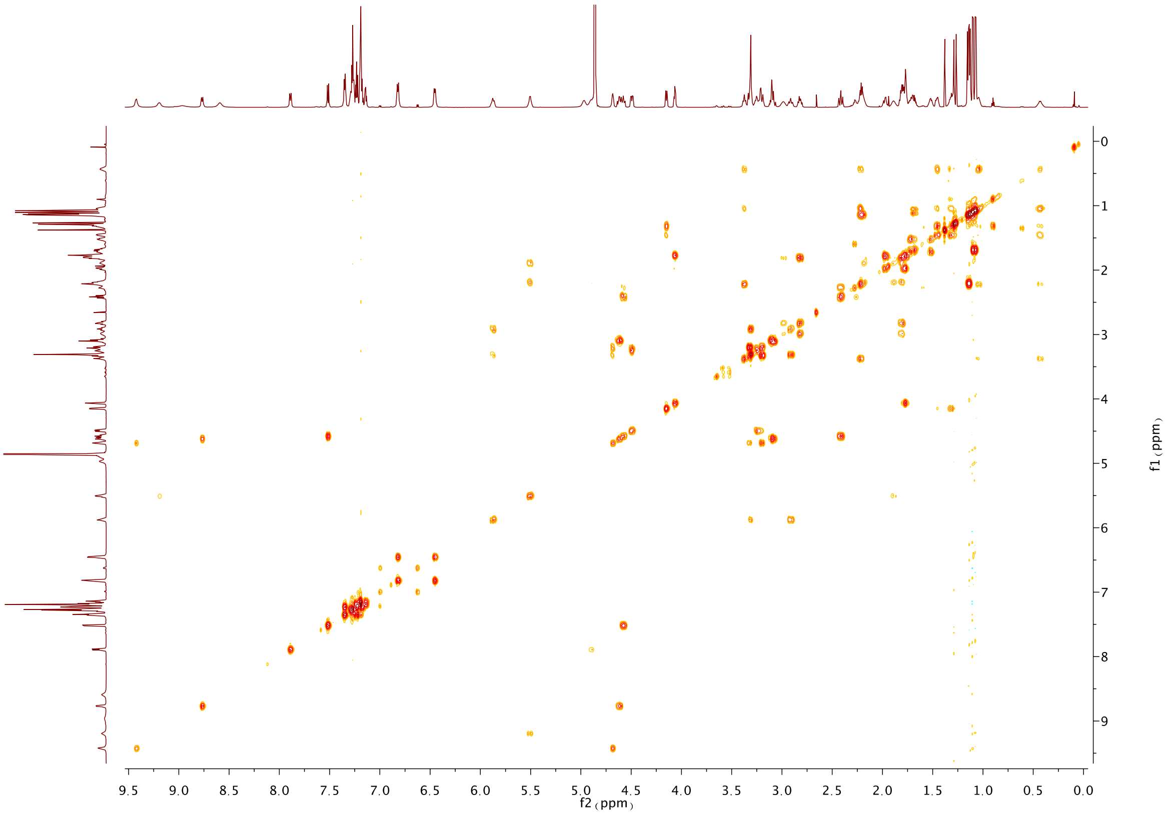

*HSQC spectrum of tyrocidine **25c** in CD_3OD.*

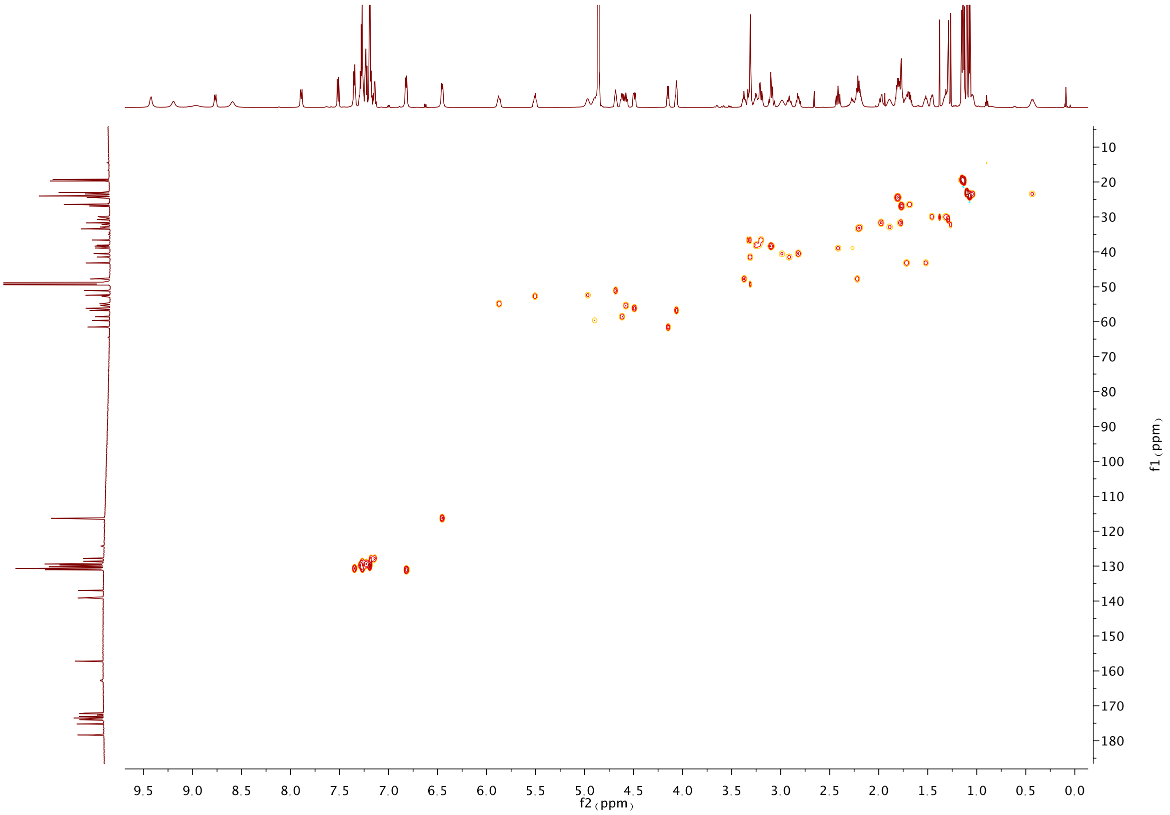

*HMBC spectrum of tyrocidine **25c** in CD_3OD.*

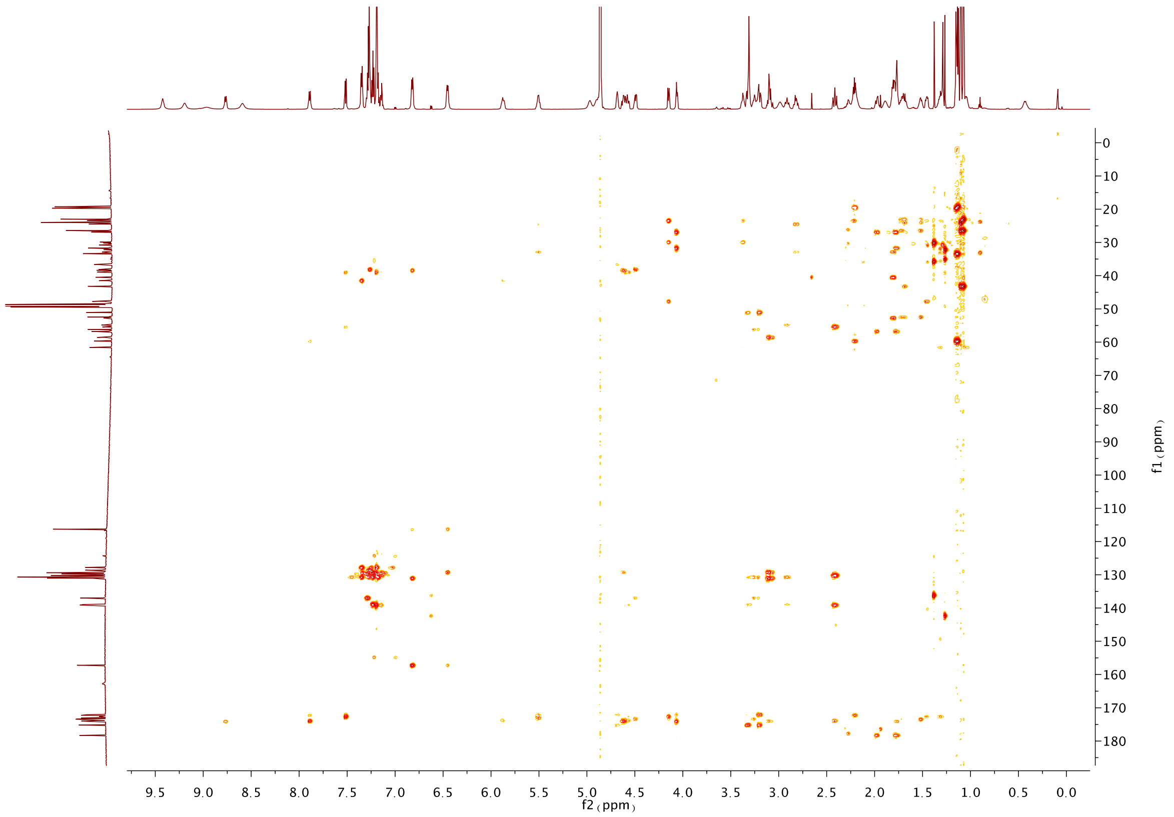

*TOCSY spectrum of tyrocidine **25c** in CD_3OD at 400 MHz.*

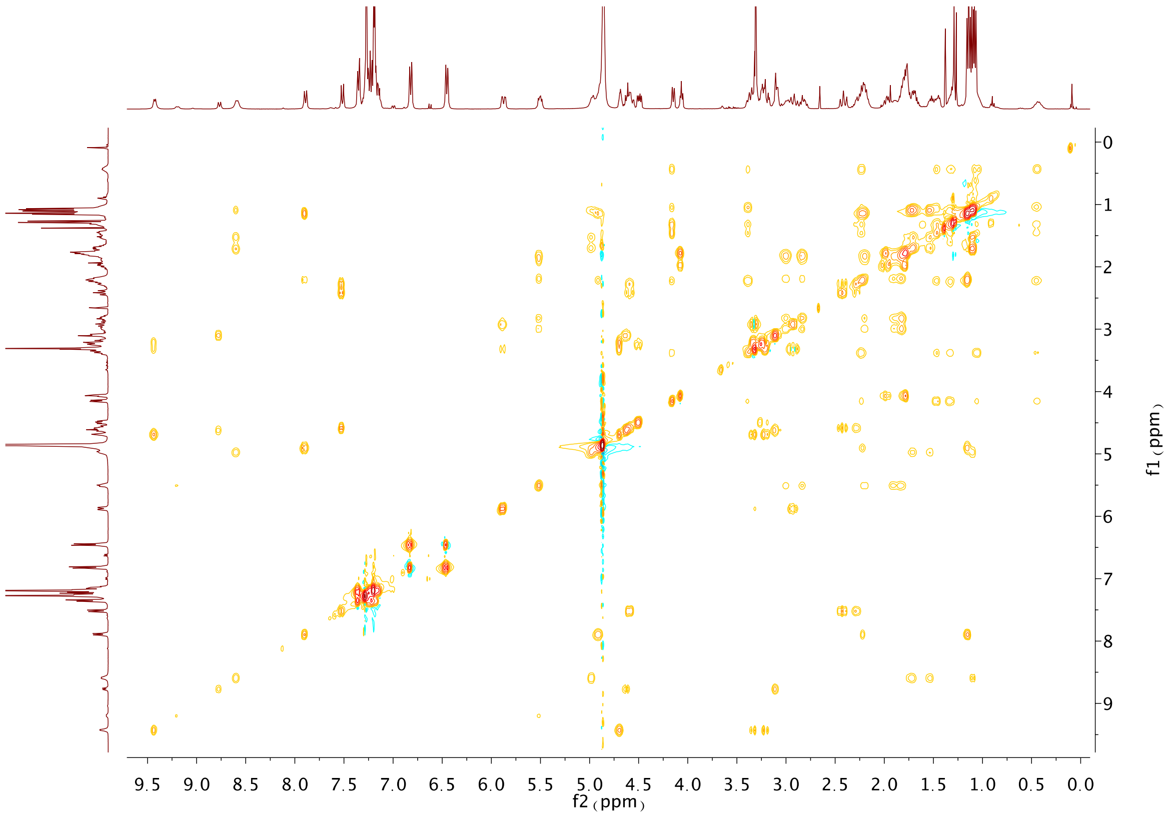

*NOESY spectrum of tyrocidine **25c** in CD_3OD at 400 MHz.*

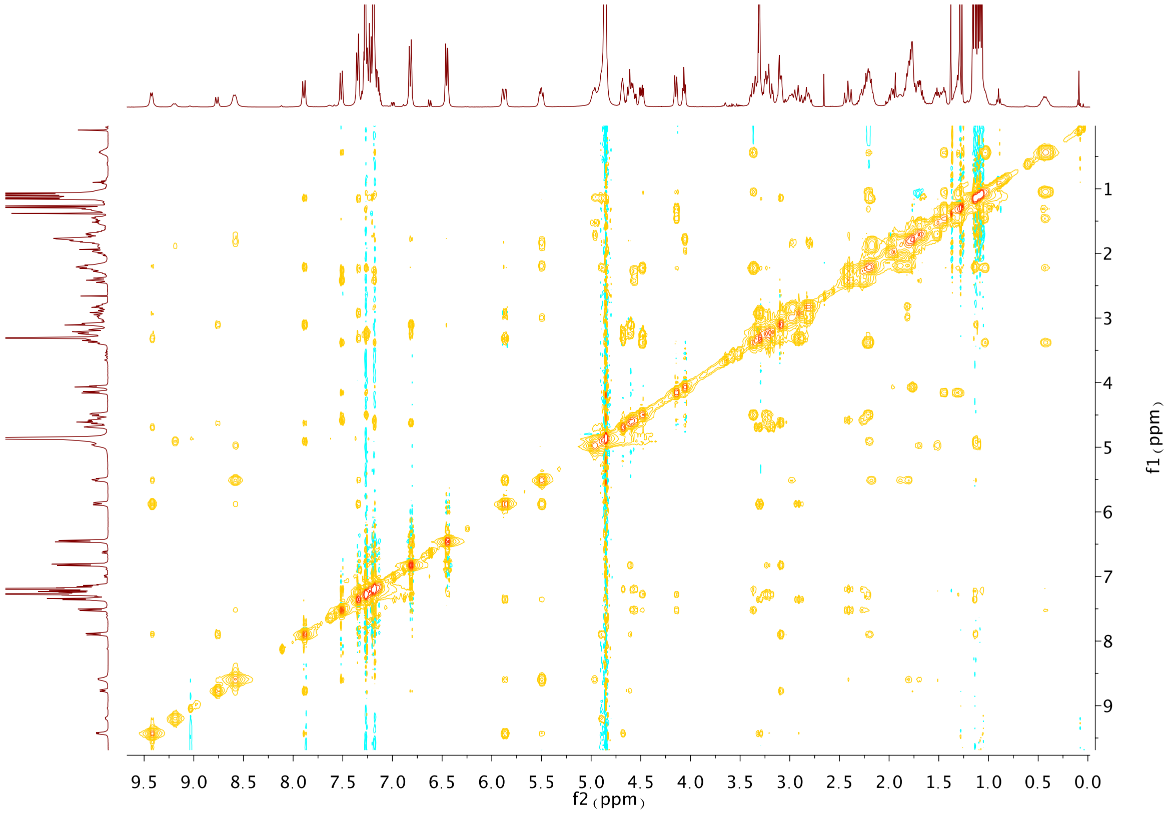

*^{1}H NMR spectrum of **26c** reversed cyclopurpuracin in $(CD_3)_2SO$ at 400 MHz.*

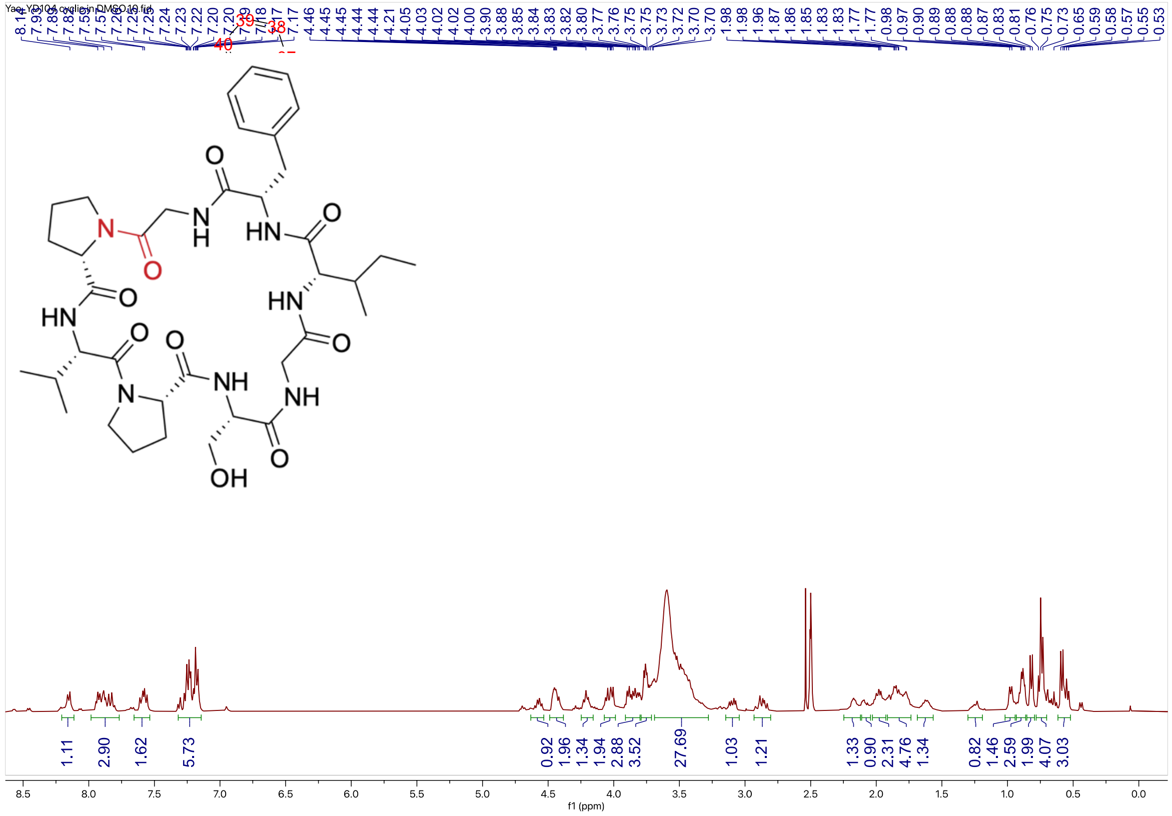

*^{13}C NMR spectrum of **26c** reversed cyclopurpuracin in $(CD_3)_2SO$ at 101 MHz.*

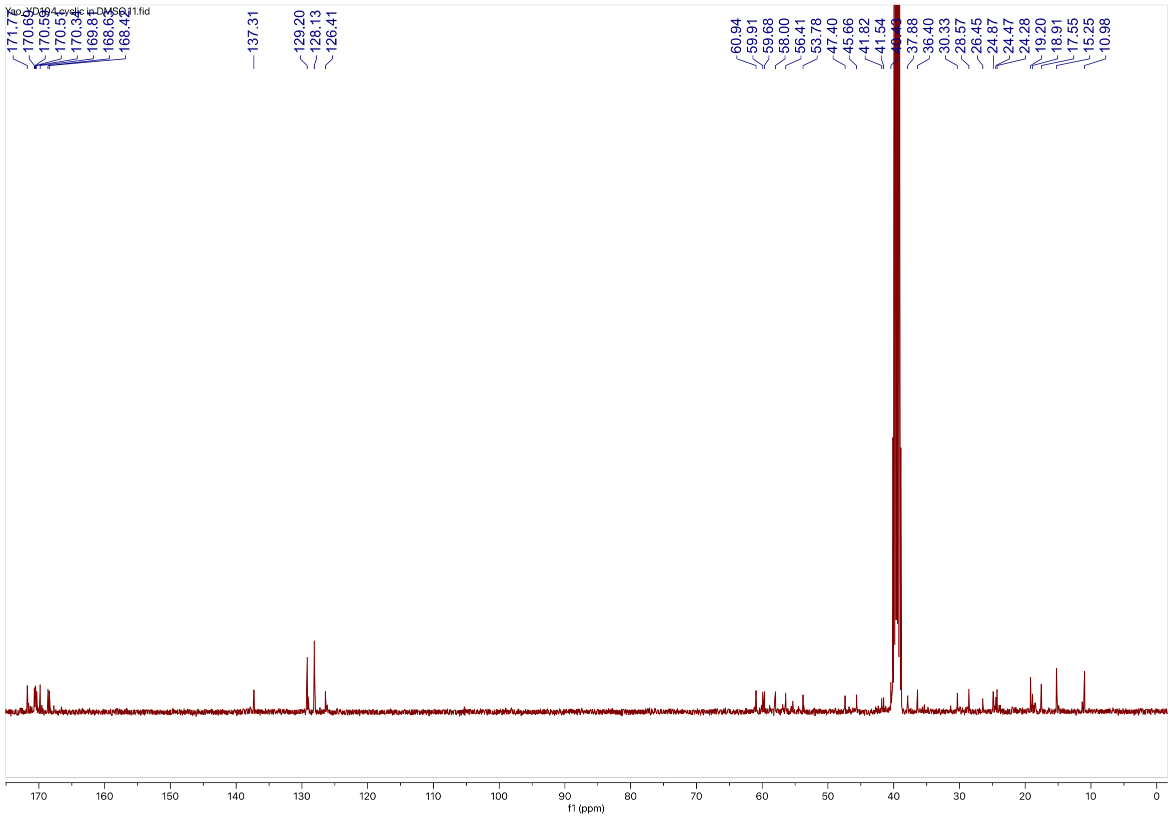

*COSY spectrum of **26c** reversed cyclopurpuracin in $(CD_3)_2SO$ at 400 MHz.*

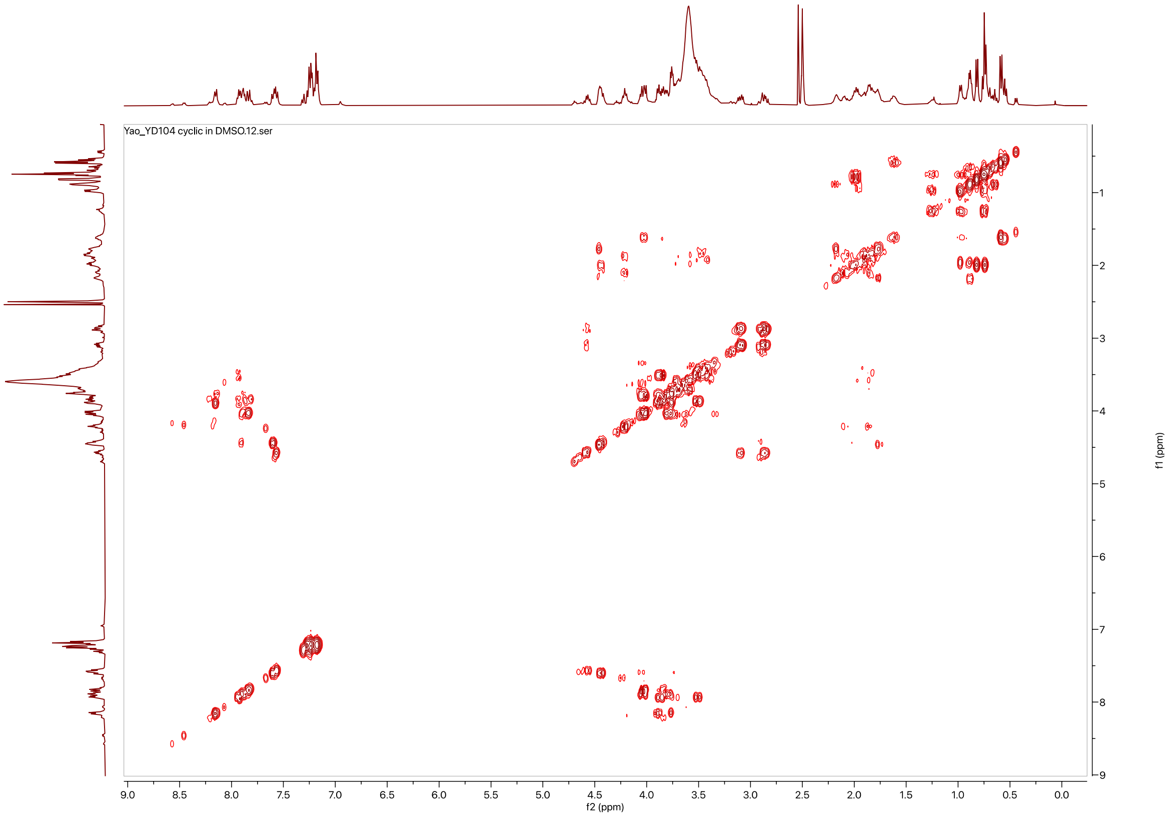

*HSQC spectrum of **26c** reversed cyclopurpuracin in $(CD_3)_2SO$.*

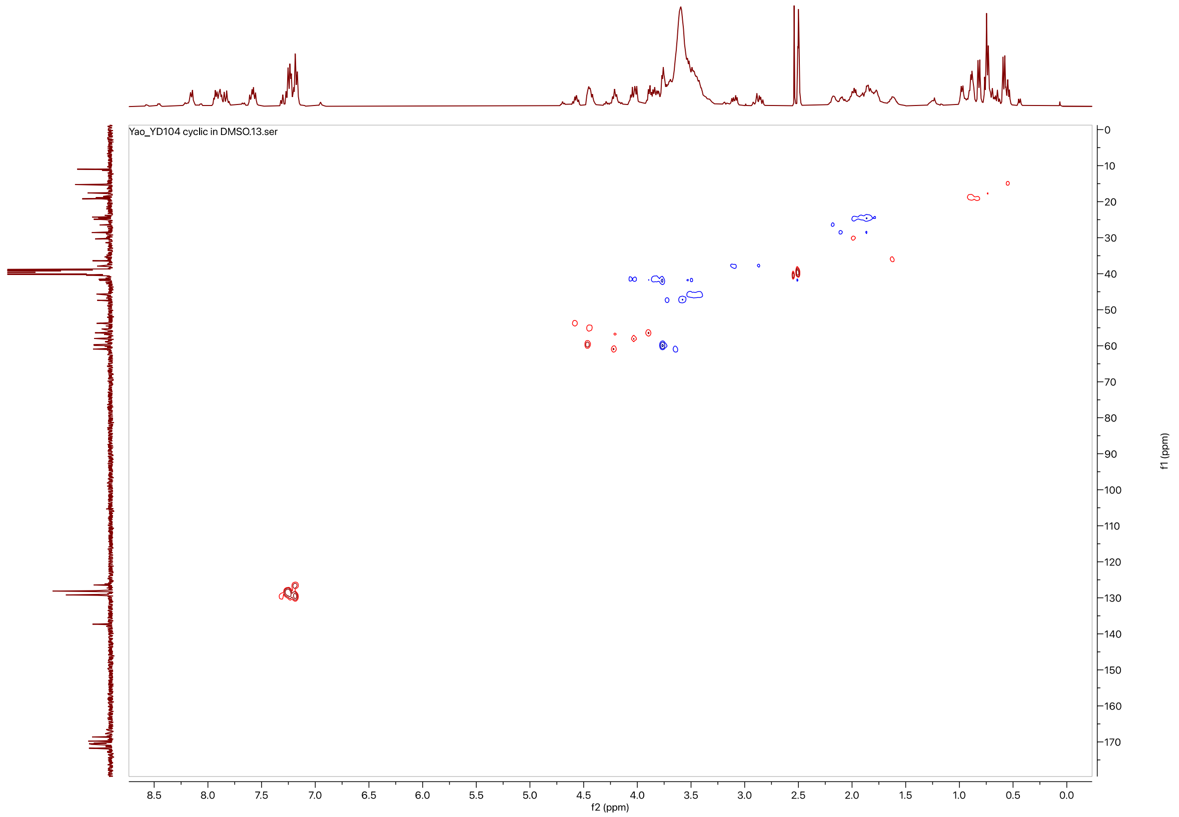

*HMBC spectrum of **26c** reversed cyclopurpuracin in $(CD_3)_2SO$.*

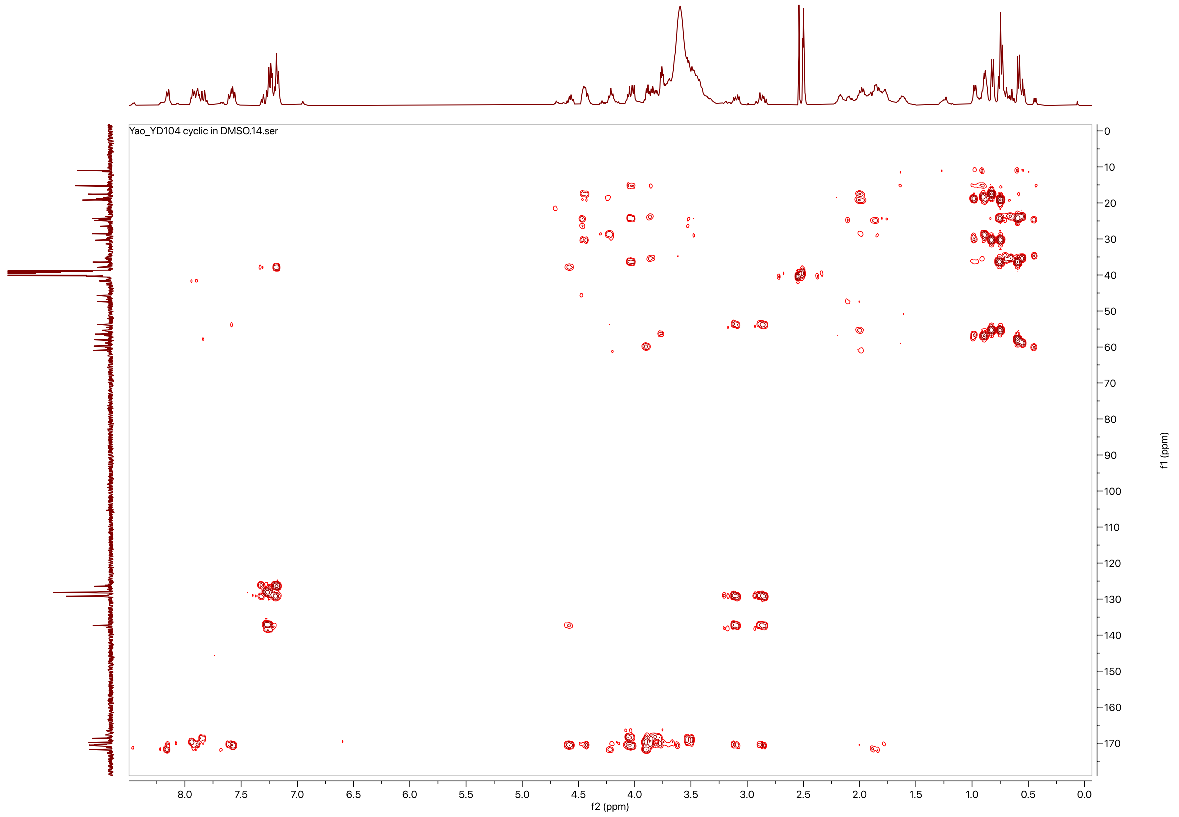

^{1}H NMR spectrum of **27c** planktocyclin in $(CD_3)_2SO$ at 400 MHz.

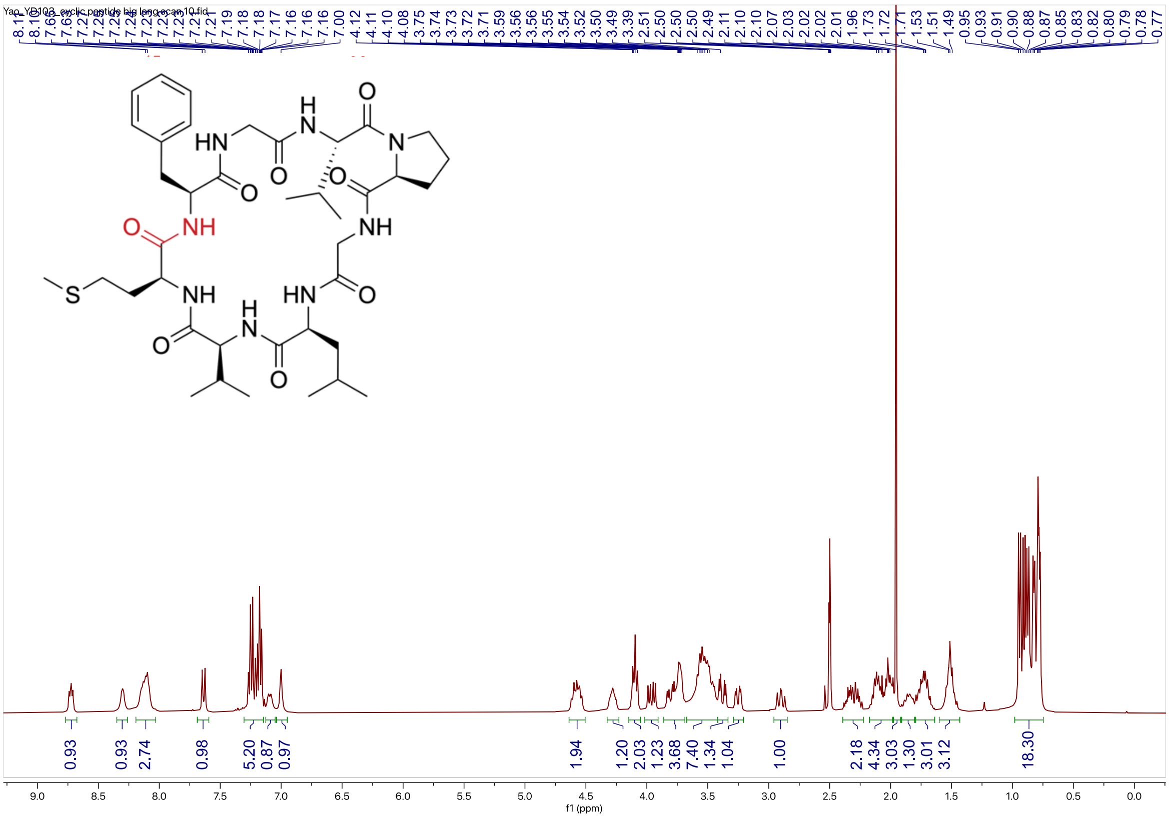

^{13}C NMR spectrum of **27c** planktocyclin in $(CD_3)_2SO$ at 101 MHz.

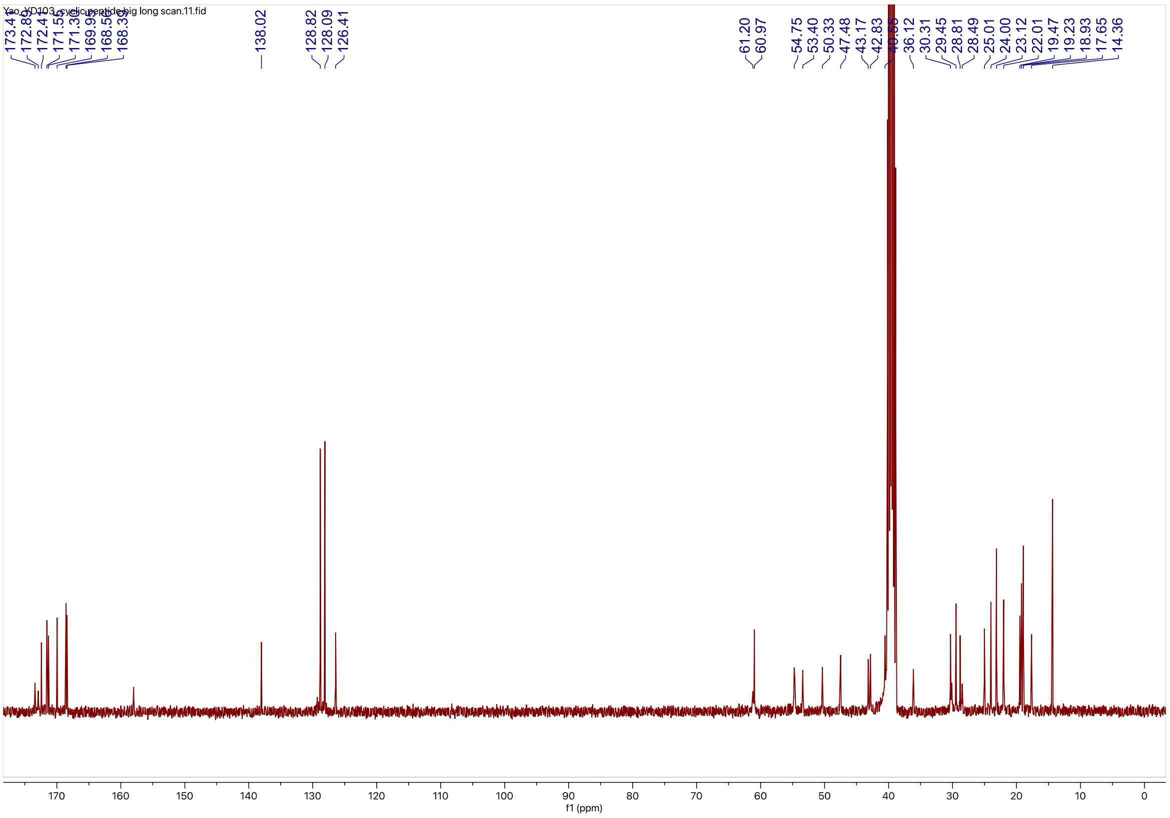

*COSY spectrum of **27c** planktocyclin in $(CD_3)_2SO$ at 400 MHz.*

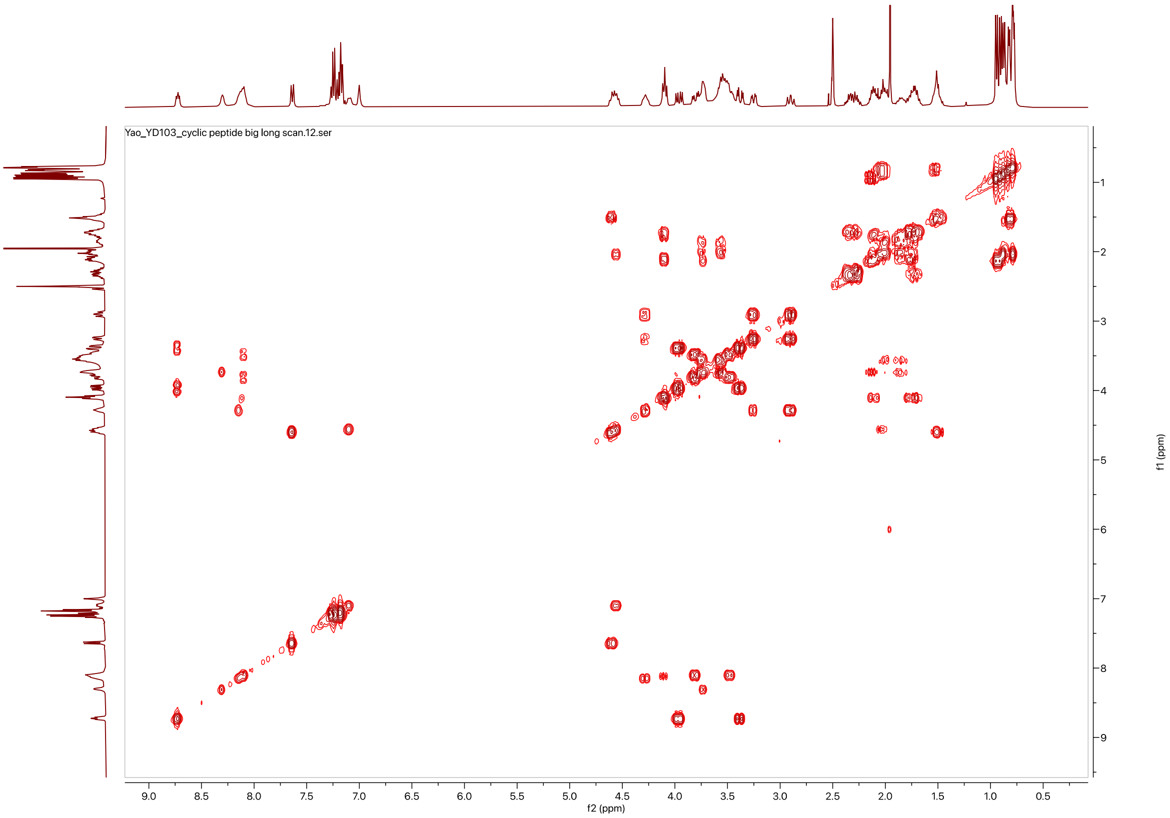

*HSQC spectrum of **27c** planktocyclin in $(CD_3)_2SO$.*

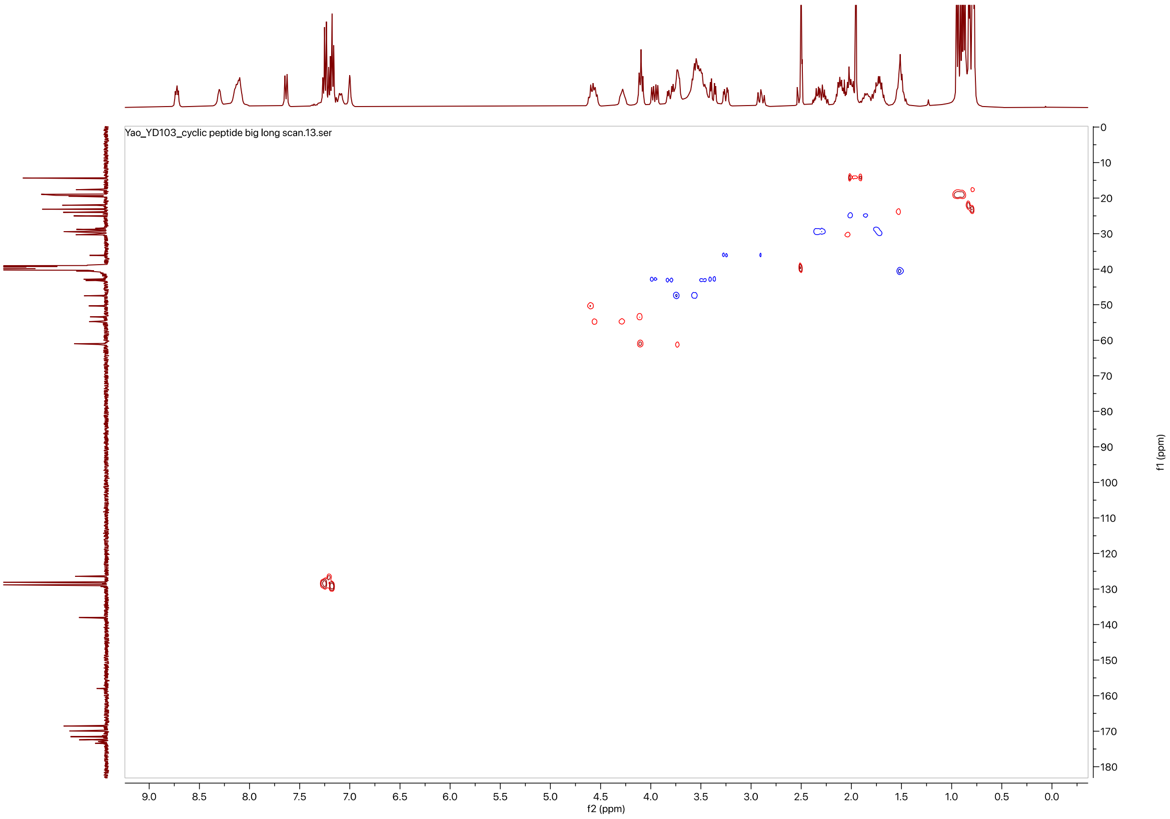

*HMBC spectrum of **27c** planktocyclin in $(CD_3)_2SO$.*

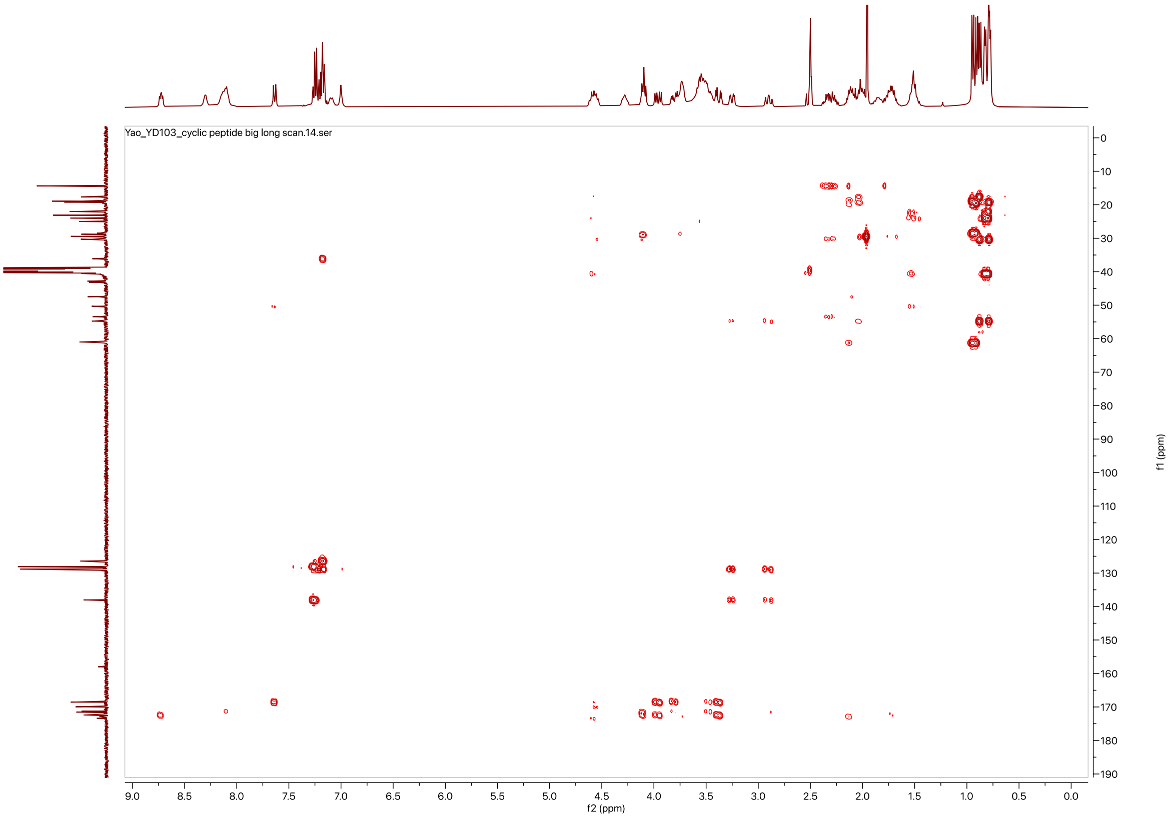

*^{1}H NMR spectrum of pseudostellarin A **28c** in $(CD_3)_2SO$ at 400 MHz.*

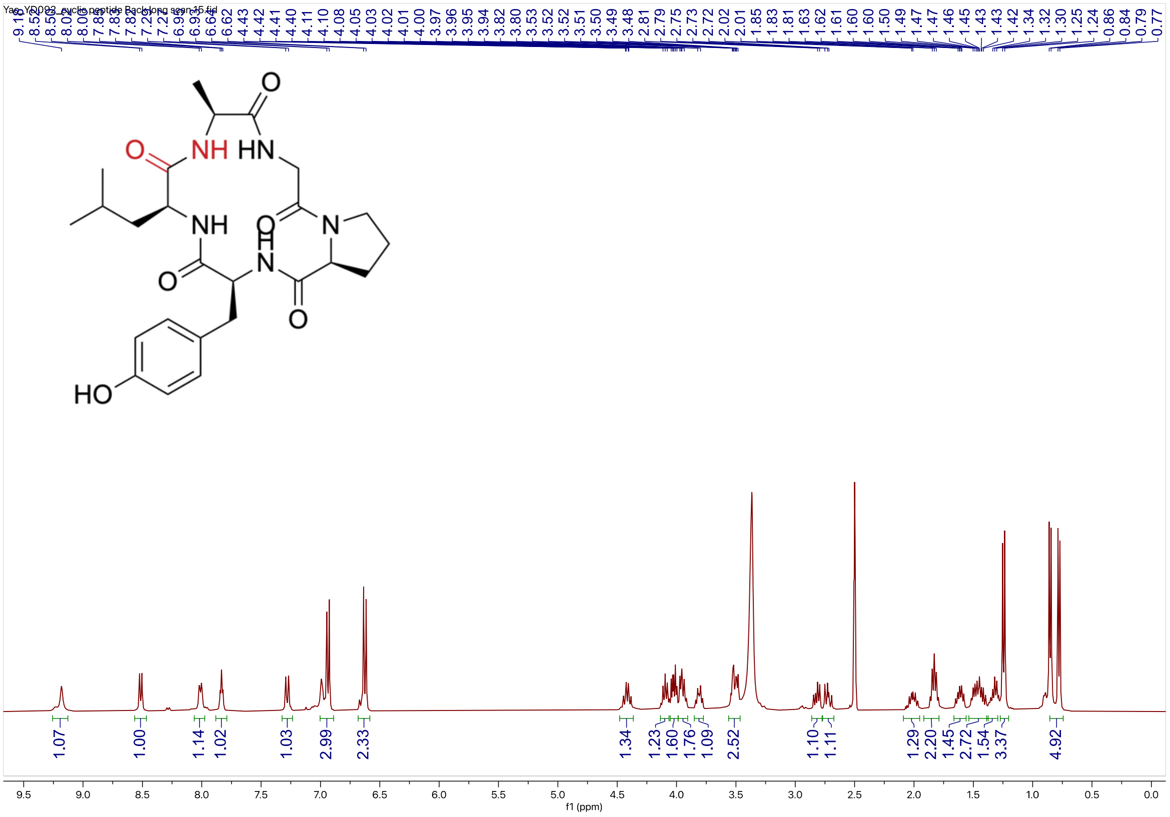

^{13}C *NMR spectrum of pseudostellarin A* ***28c*** *in* $(CD_3)_2SO$ *at 101 MHz.*

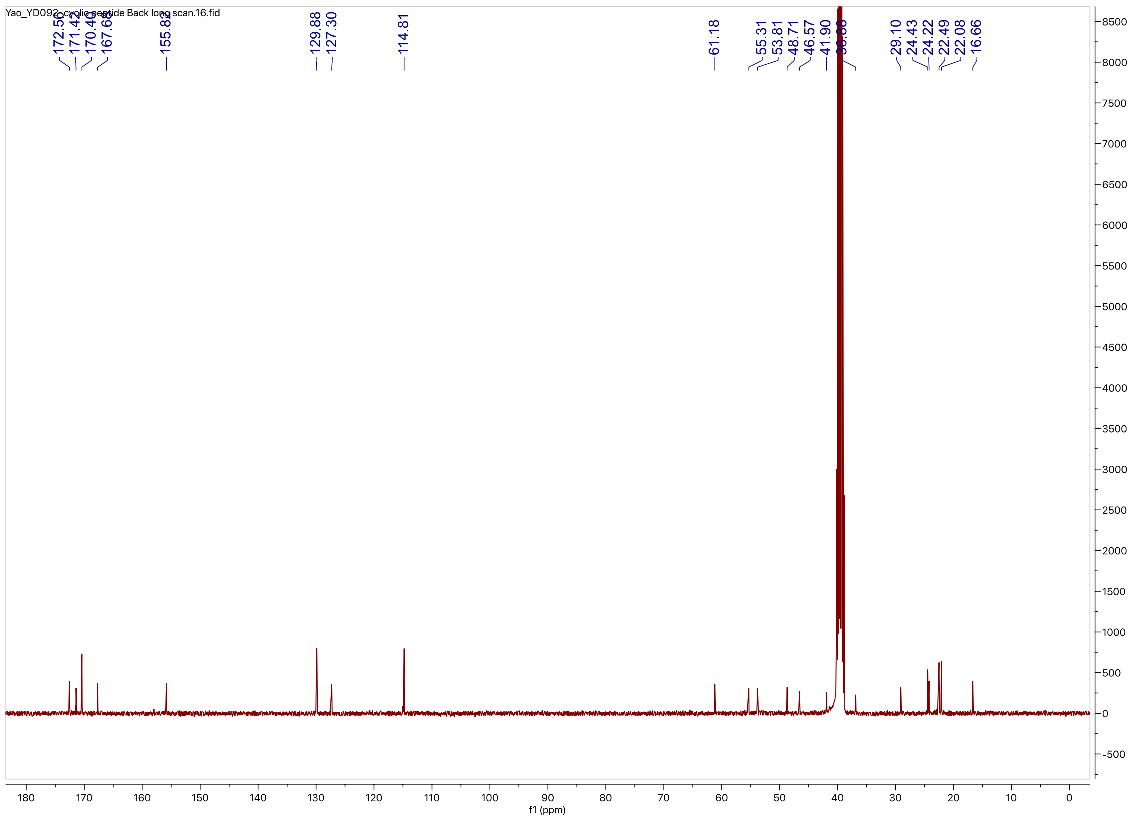

COSY spectrum of pseudostellarin A ***28c*** *in* $(CD_3)_2SO$ *at 400 MHz.*

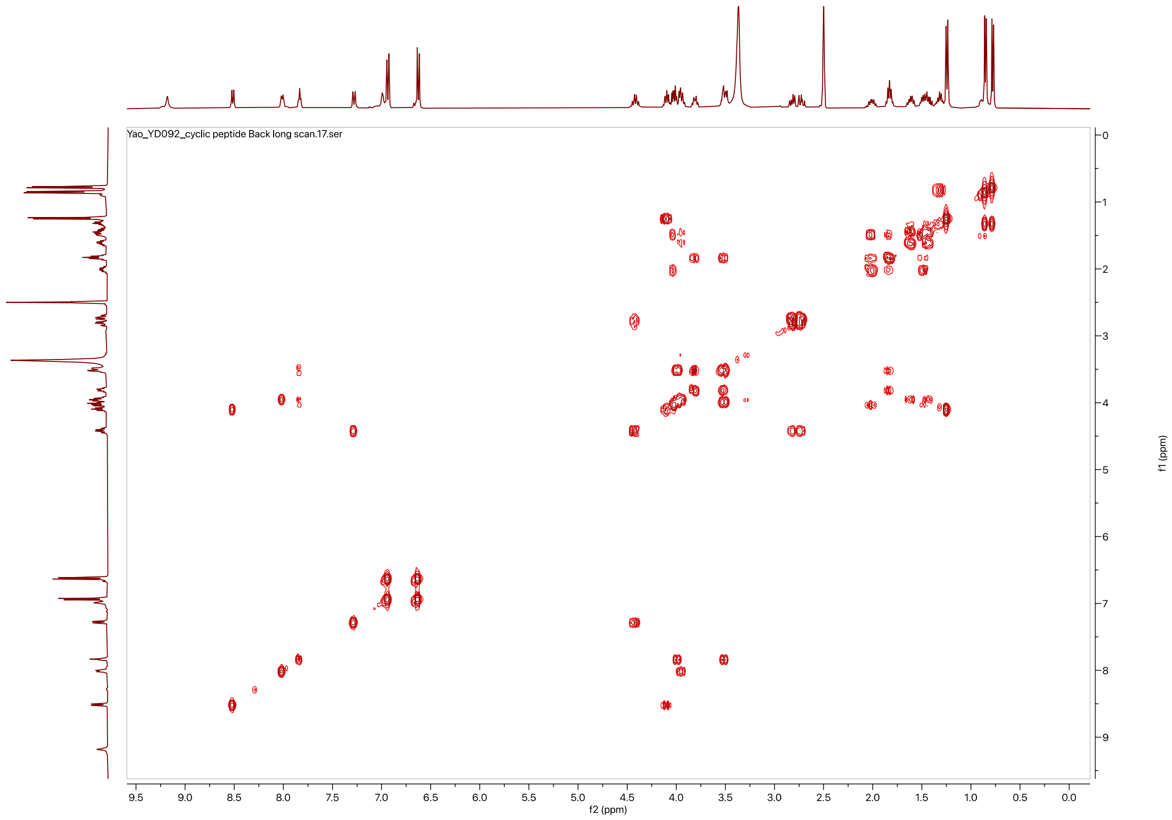

*HSQC spectrum of pseudostellarin A **28c** in $(CD_3)_2SO$.*

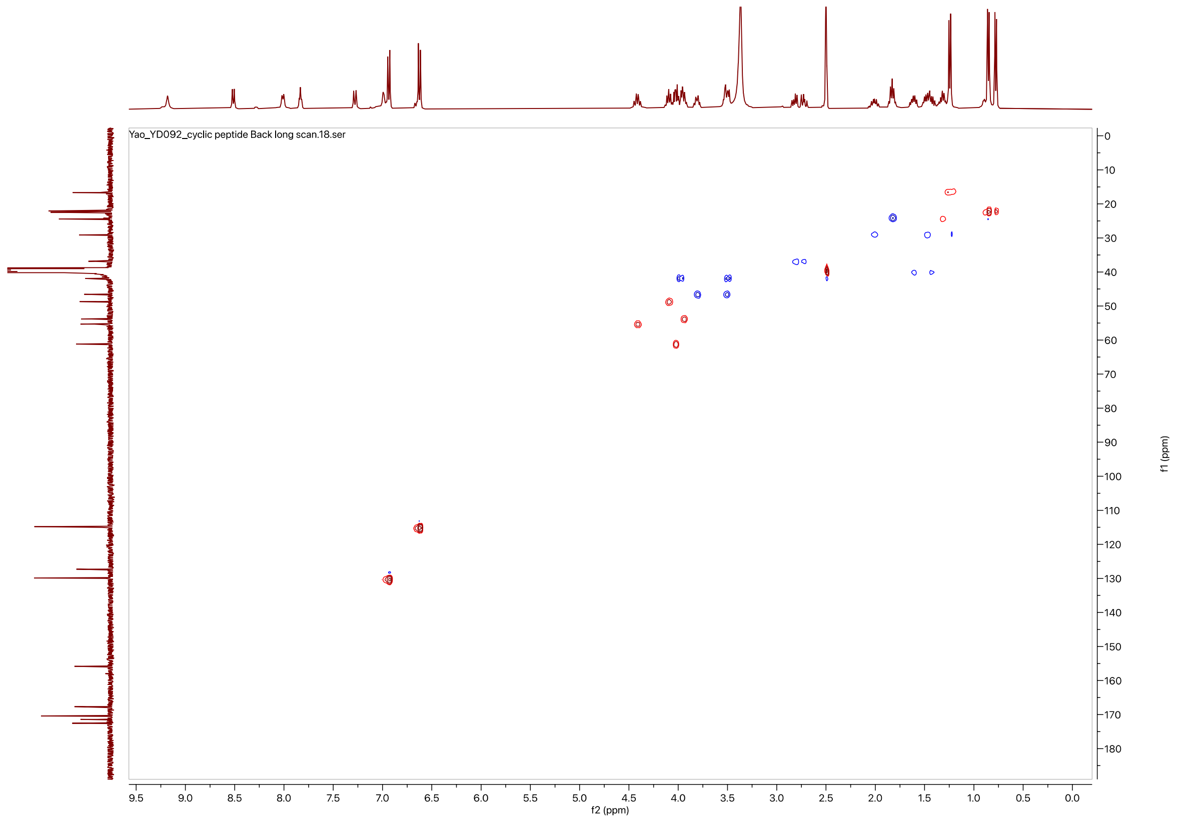

*HMBC spectrum of pseudostellarin A **28c** in $(CD_3)_2SO$.*

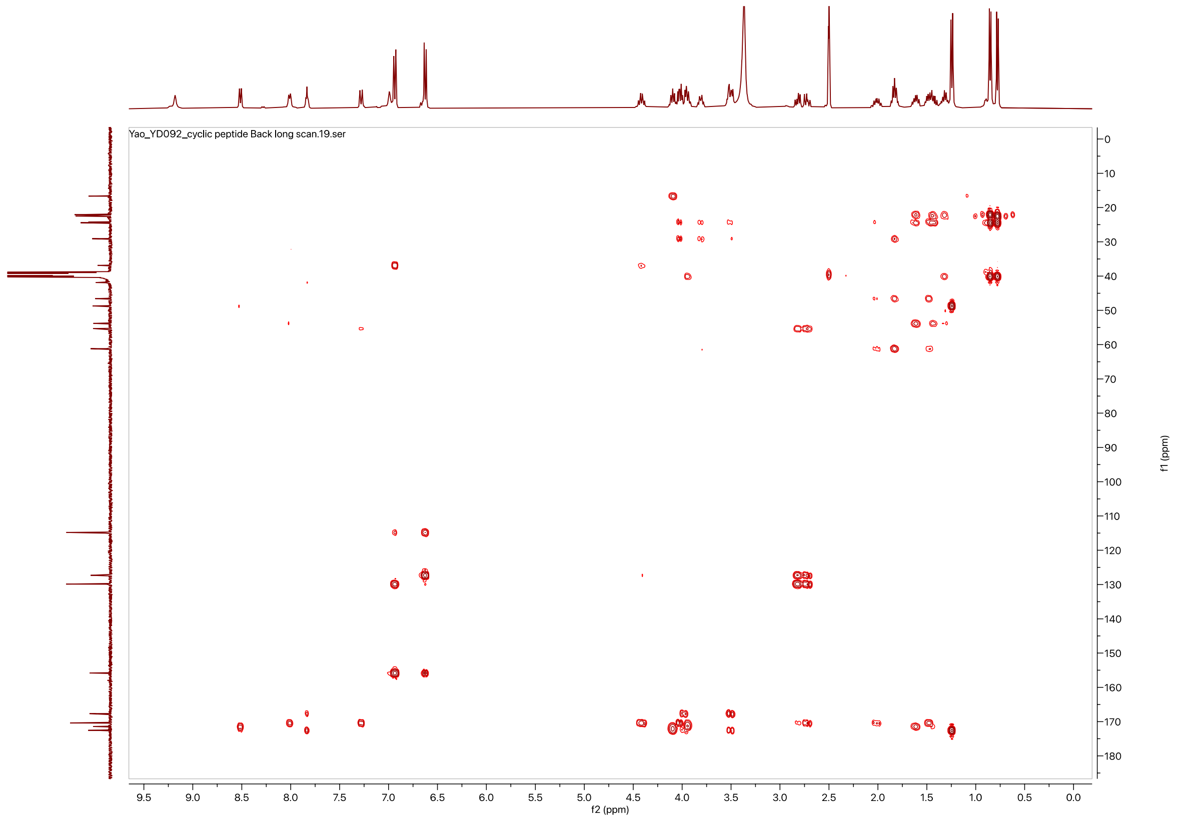

*ROESY spectrum of pseudostellarin A **28c** in $(CD_3)_2SO$ at 400 MHz.*

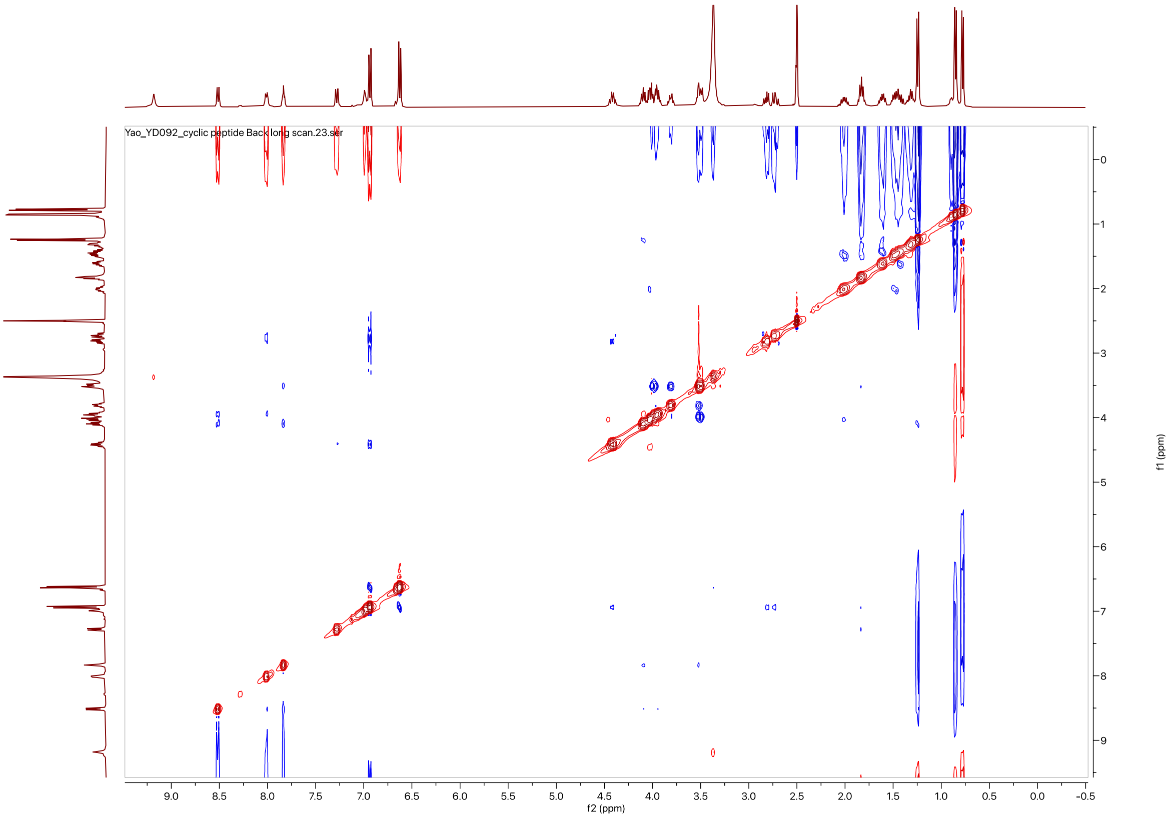

*^1H NMR spectrum of Gramicidin S **29c** in $(CD_3)_2SO$ at 400 MHz.*

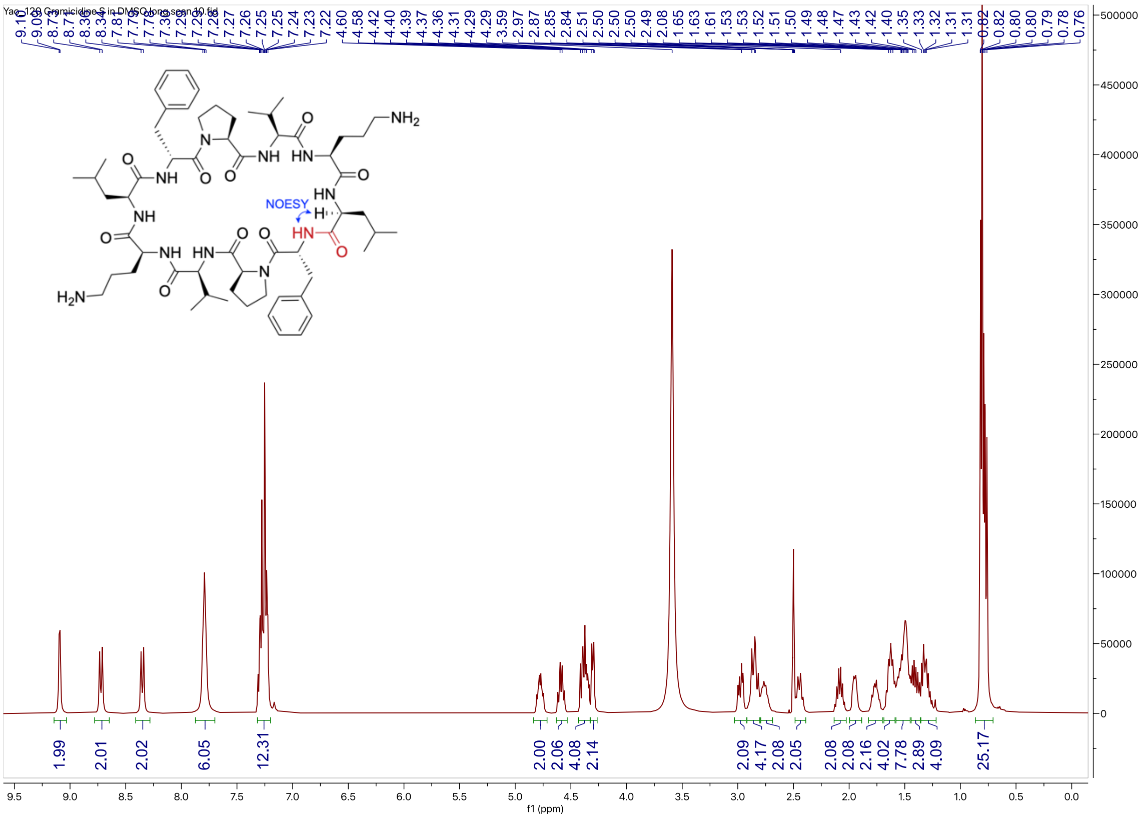

*^{13}C NMR spectrum of Gramicidin S **29c** in $(CD_3)_2SO$ at 101 MHz.*

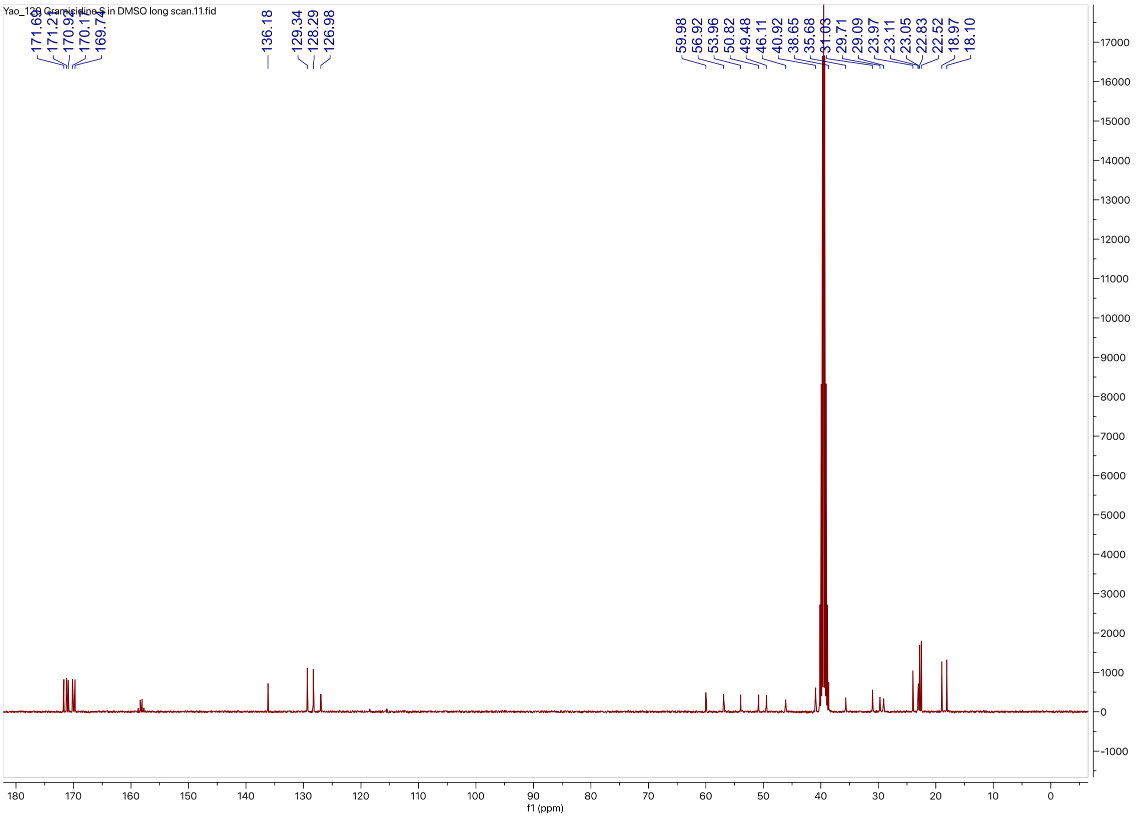

*COSY spectrum of Gramicidin S **29c** in $(CD_3)_2SO$ at 400 MHz.*

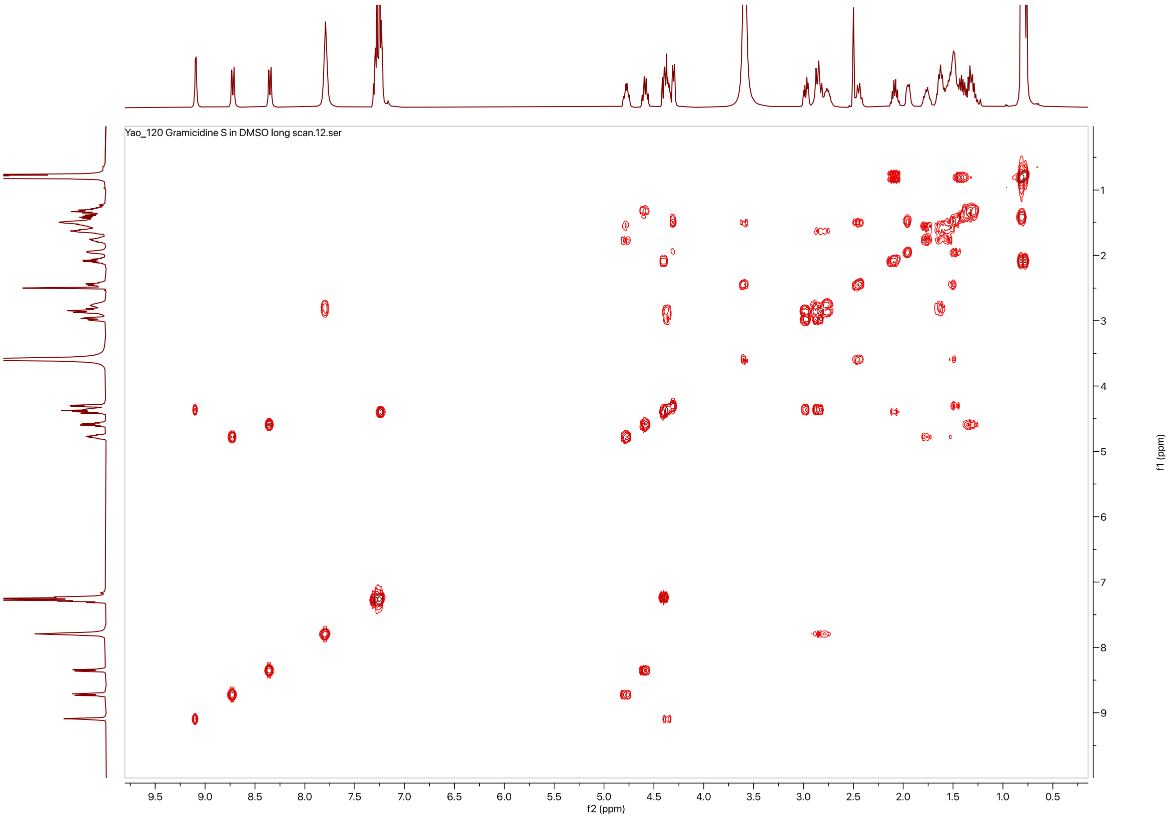

HSQC spectrum of Gramicidin S ***29c*** *in $(CD_3)_2SO$.*

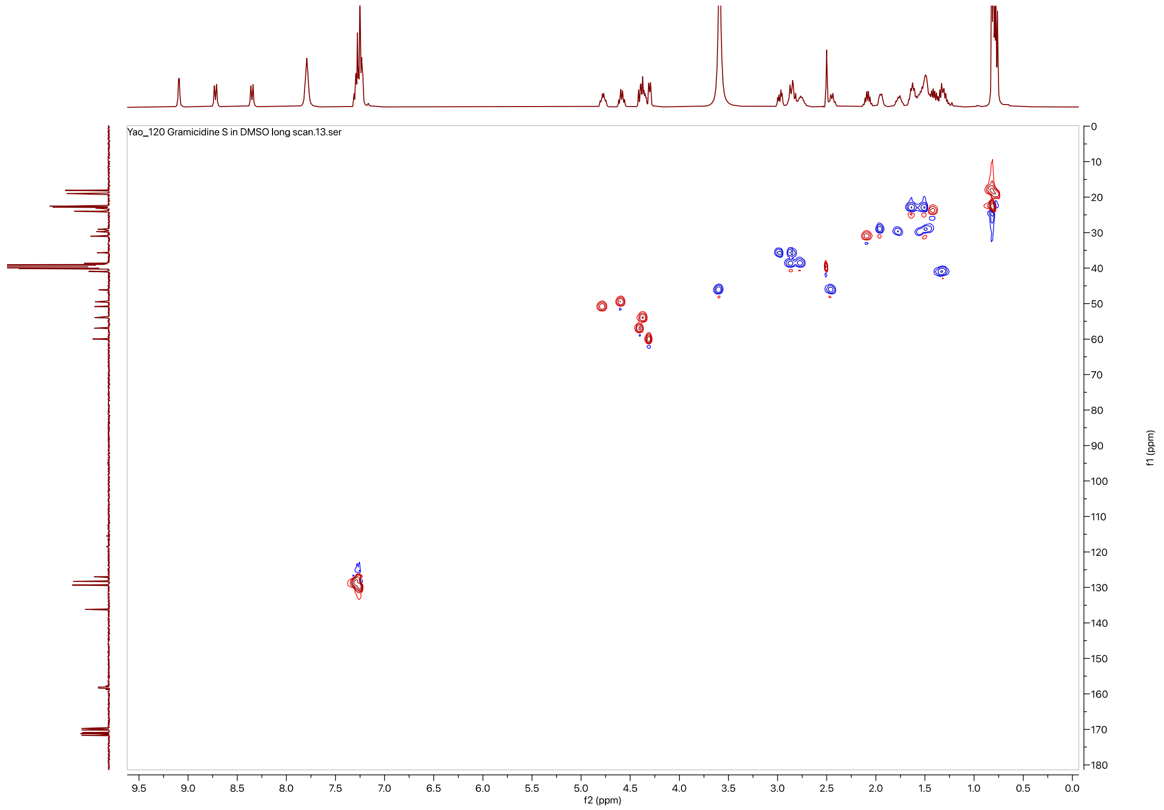

HMBC spectrum of Gramicidin S ***29c*** *in $(CD_3)_2SO$.*

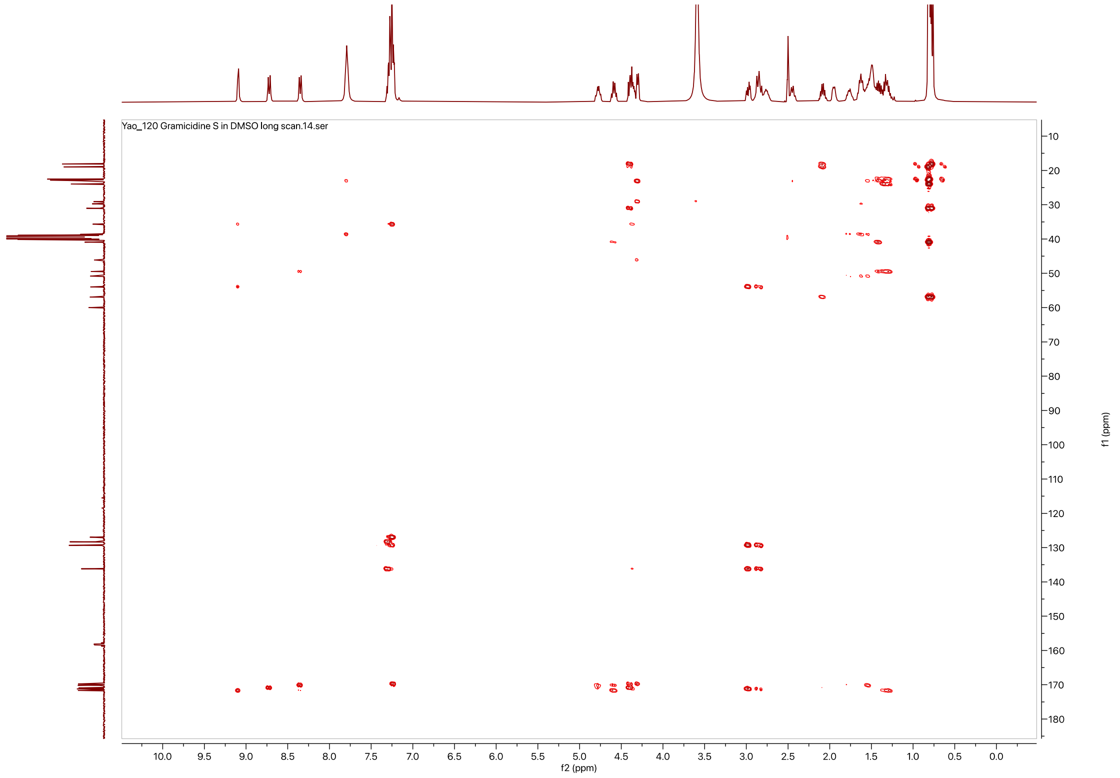

*NOESY spectrum of Gramicidin S **29c** in $(CD_3)_2SO$ at 400 MHz.*

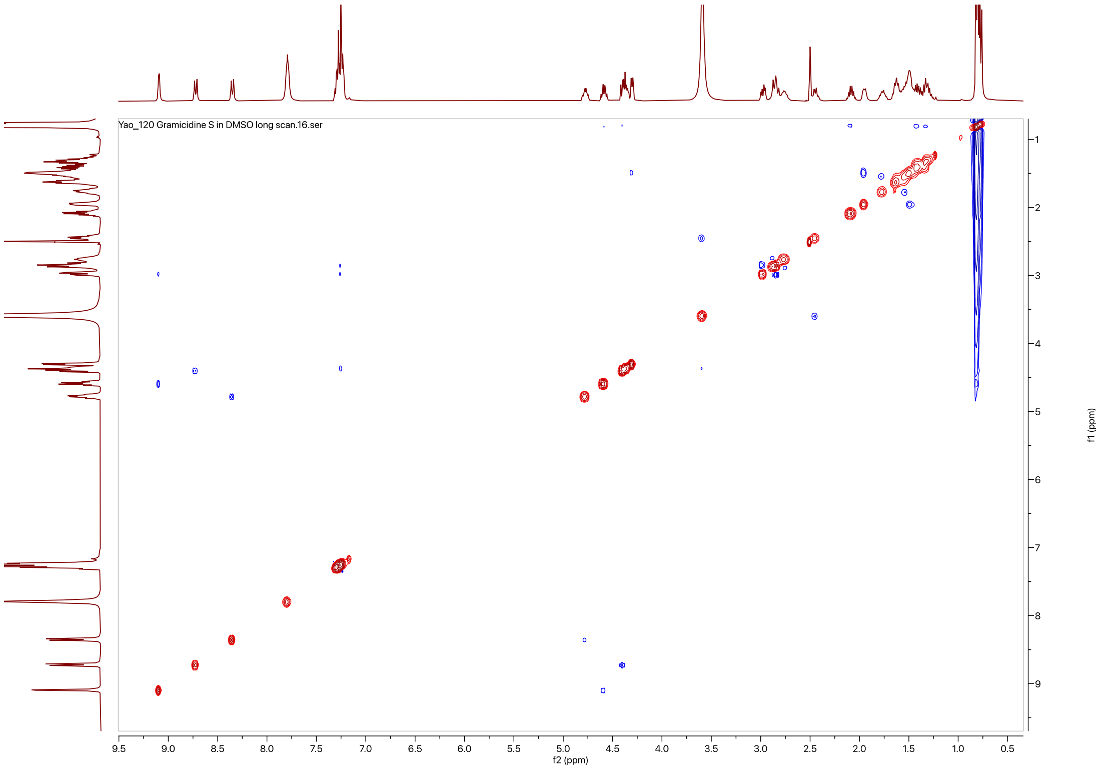

*^1H NMR spectrum of colistin analogue **30e** in D_2O at 400 MHz.*

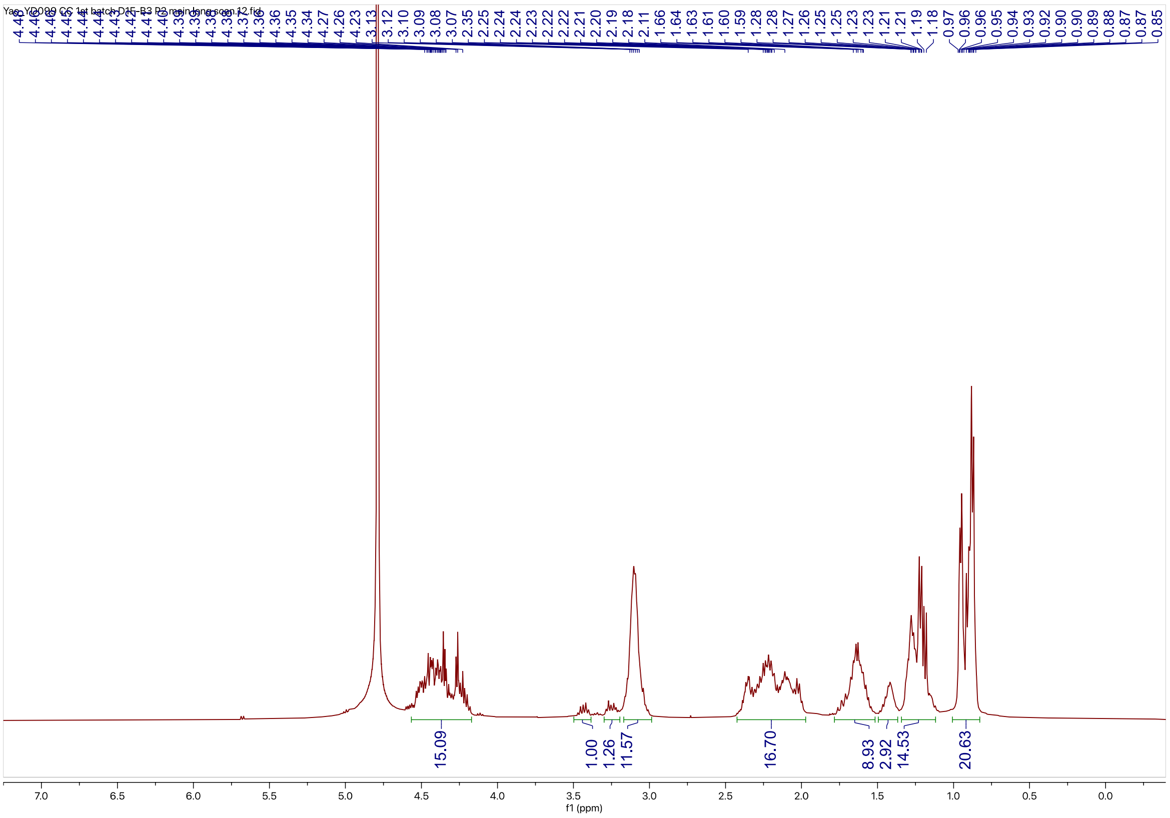

^{1}H NMR spectrum of colistin analogue **30c** in D_2O at 400 MHz.

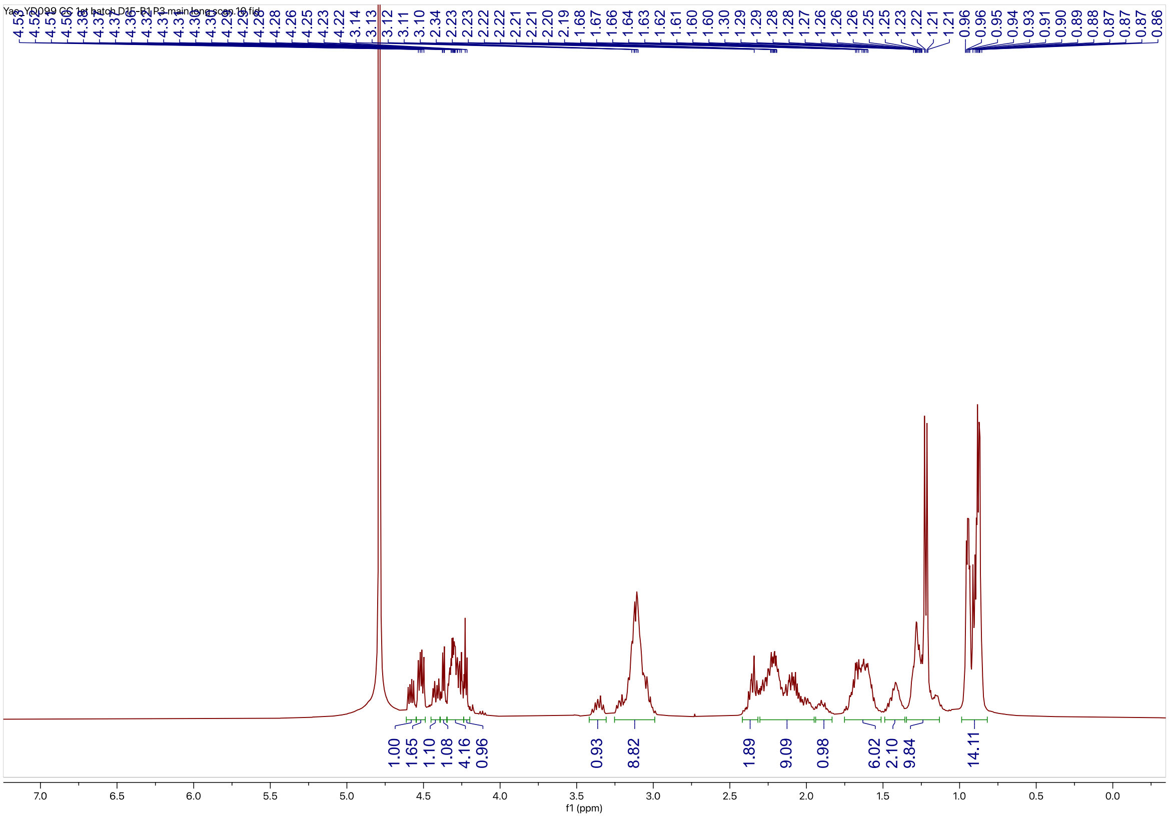

^{1}H NMR spectrum of commercial colistin **30g** in D_2O at 400 MHz.

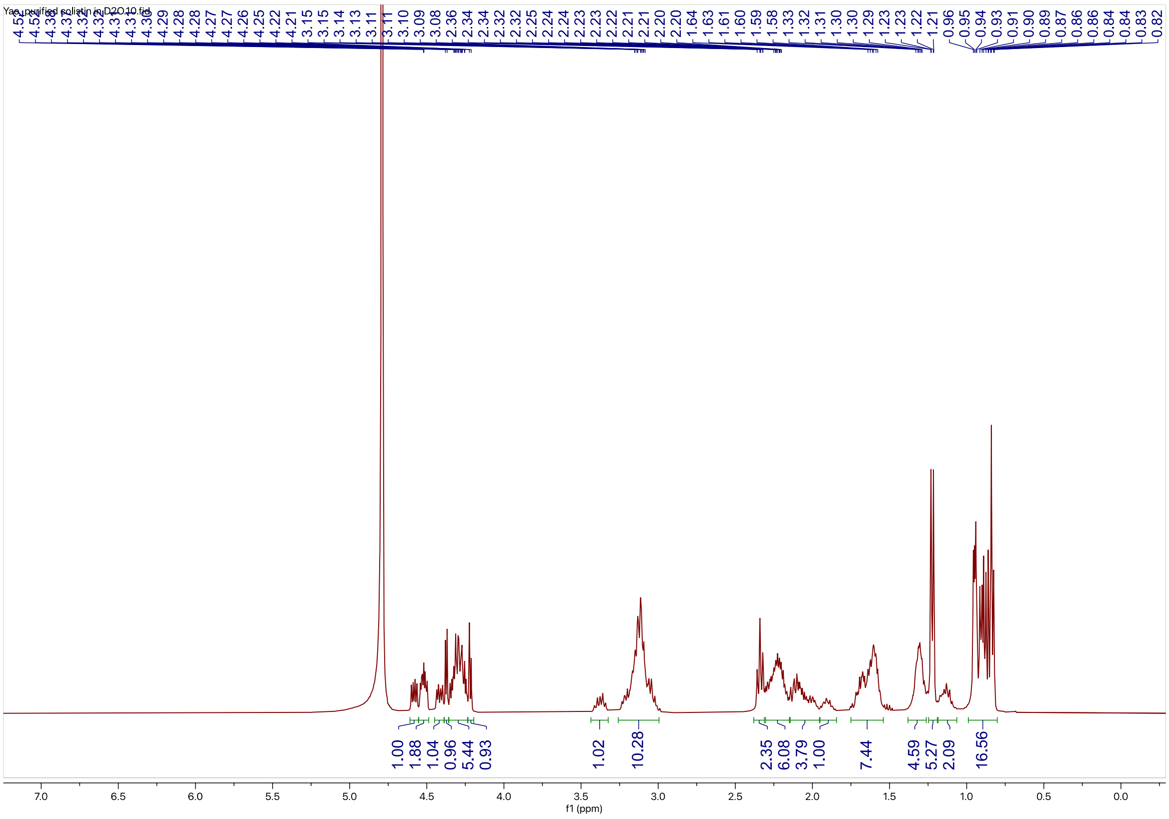

*^{1}H NMR spectrum of cyclosporine analogue **31c** in $(CD_3)_2SO$ at 400 MHz.*

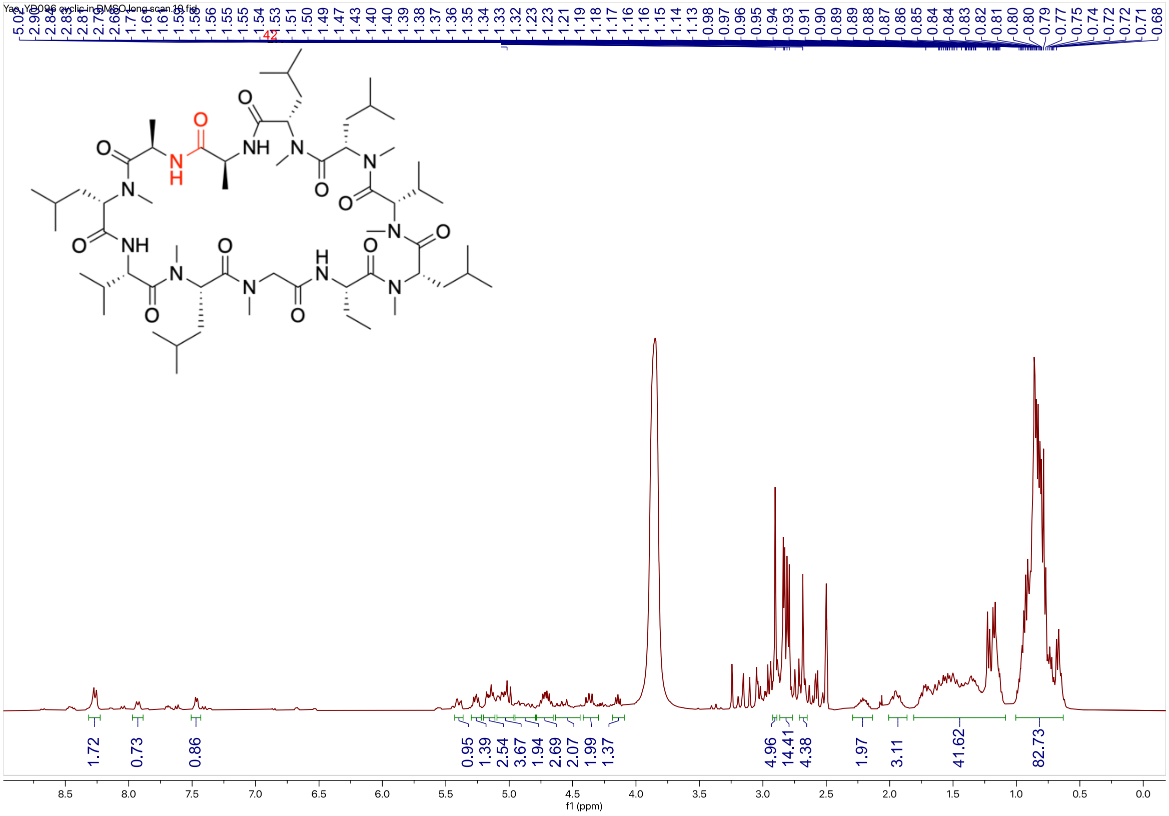

*^{13}C NMR spectrum of cyclosporine analogue **31c** in $(CD_3)_2SO$ at 101 MHz.*

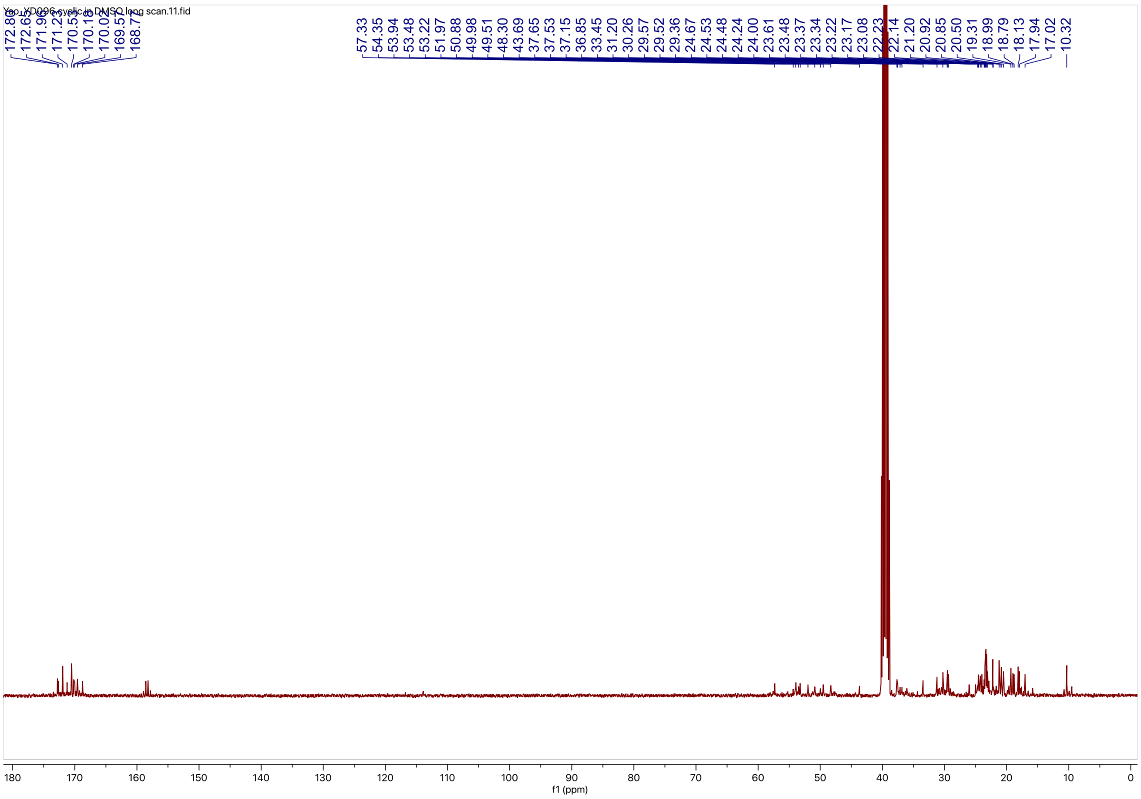

*COSY spectrum of cyclosporine analogue **31c** in $(CD_3)_2SO$ at 400 MHz.*

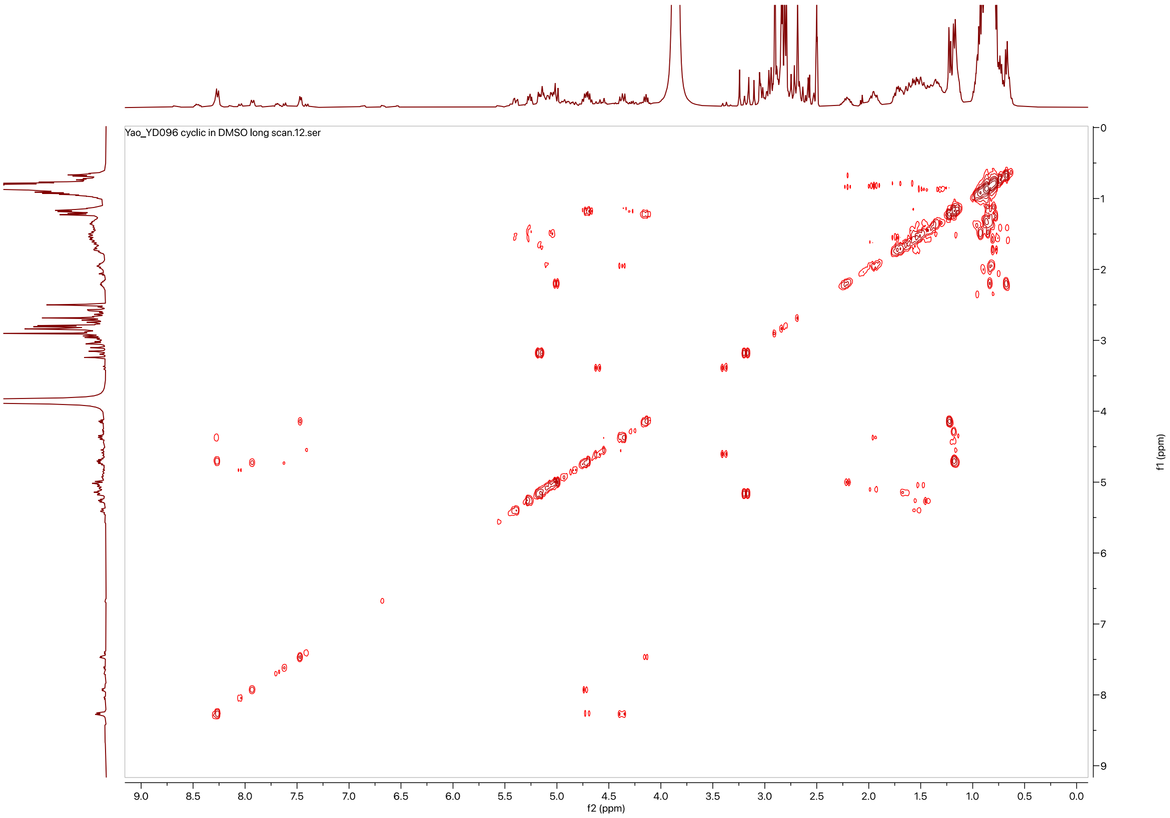

*HSQC spectrum of cyclosporine analogue **31c** in $(CD_3)_2SO$.*

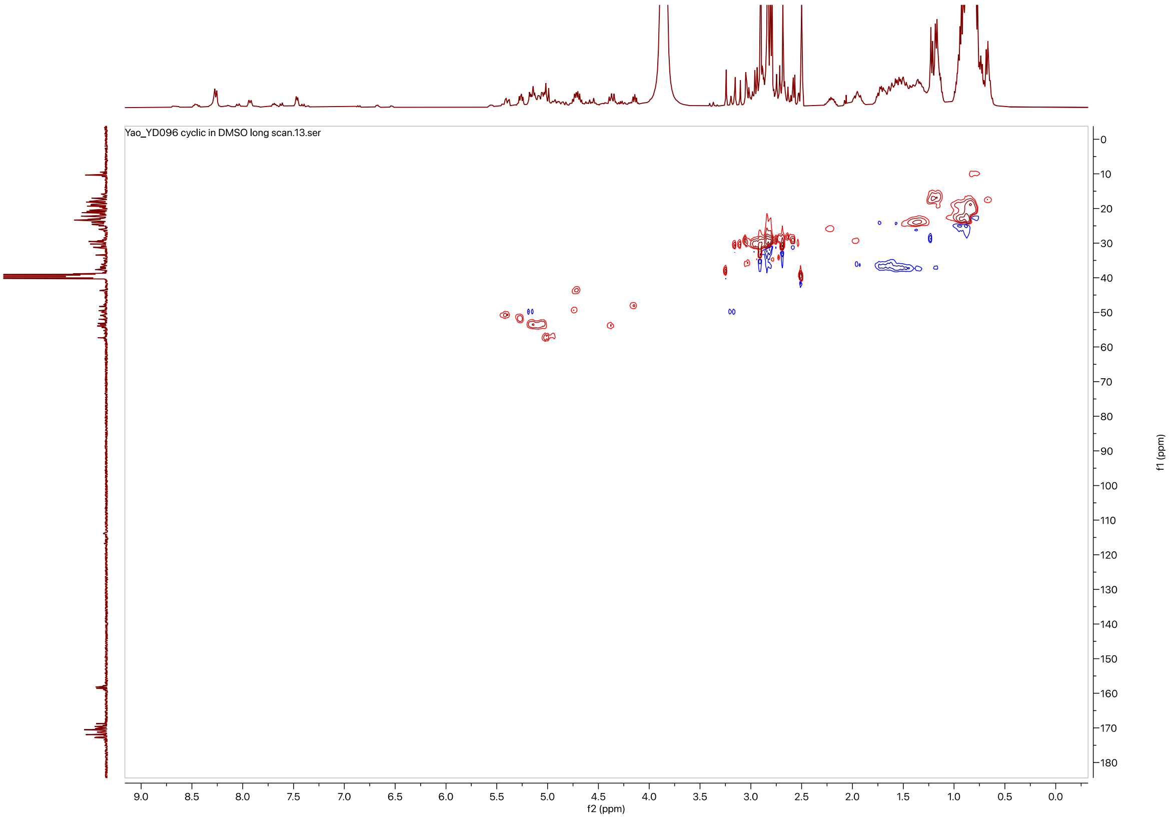

*HMBC spectrum of cyclosporine analogue **31c** in $(CD_3)_2SO$.*

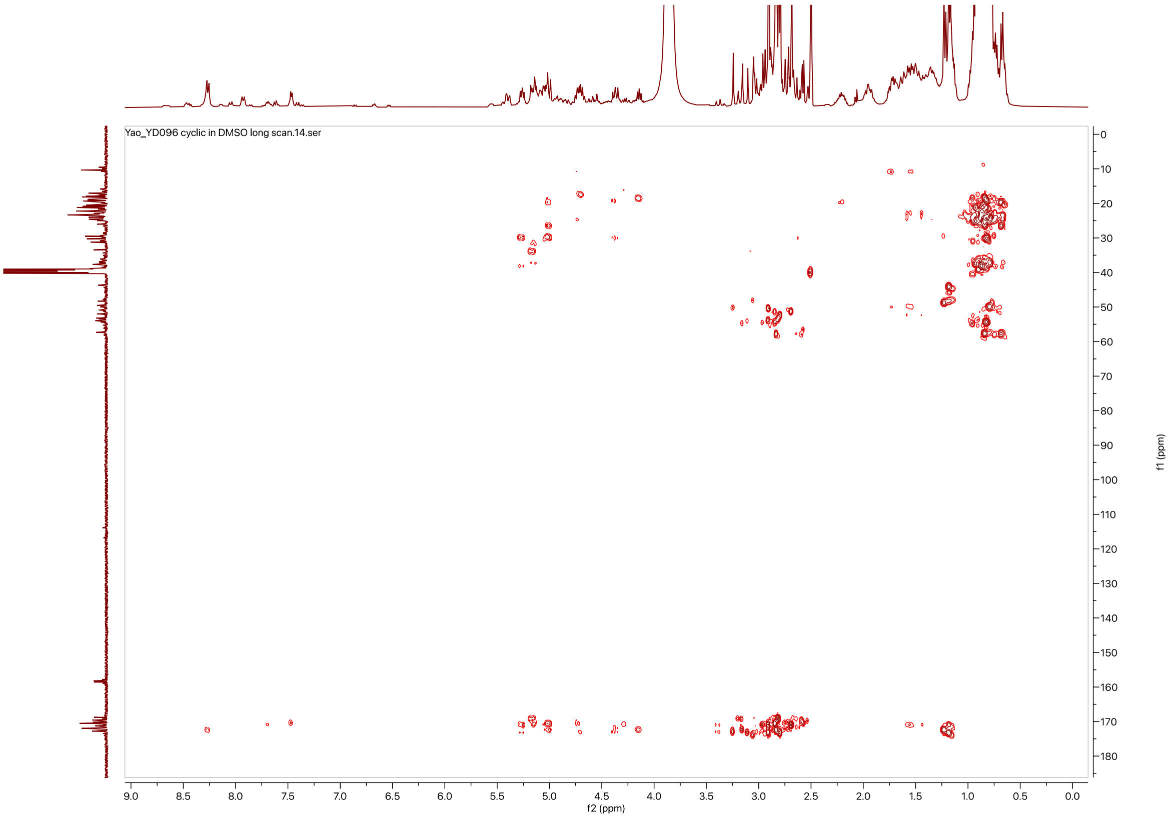

*^1H NMR spectrum of cyclomarin analogue **32c** in $(CD_3)_2SO$ at 400 MHz.*

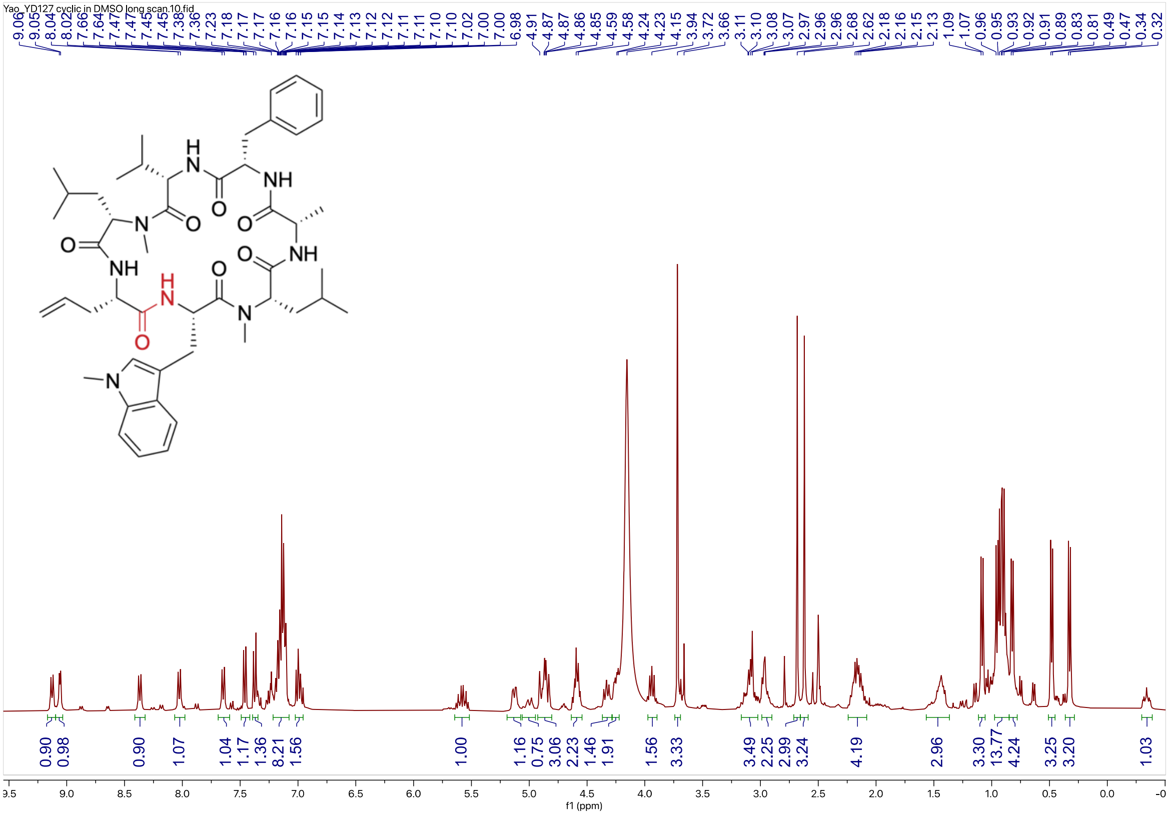

*^{13}C NMR spectrum of cyclomarin analogue **32c** in $(CD_3)_2SO$ at 101 MHz.*

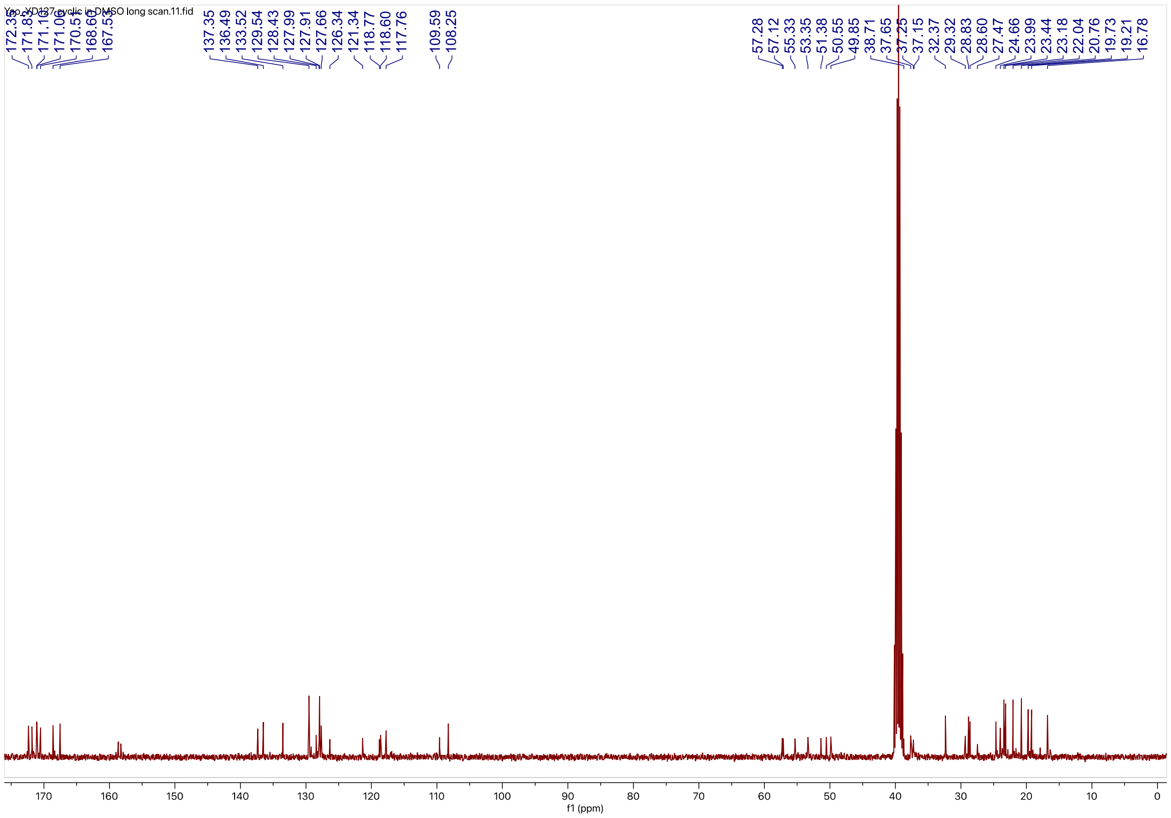

*COSY spectrum of cyclomarin analogue **32c** in $(CD_3)_2SO$ at 400 MHz.*

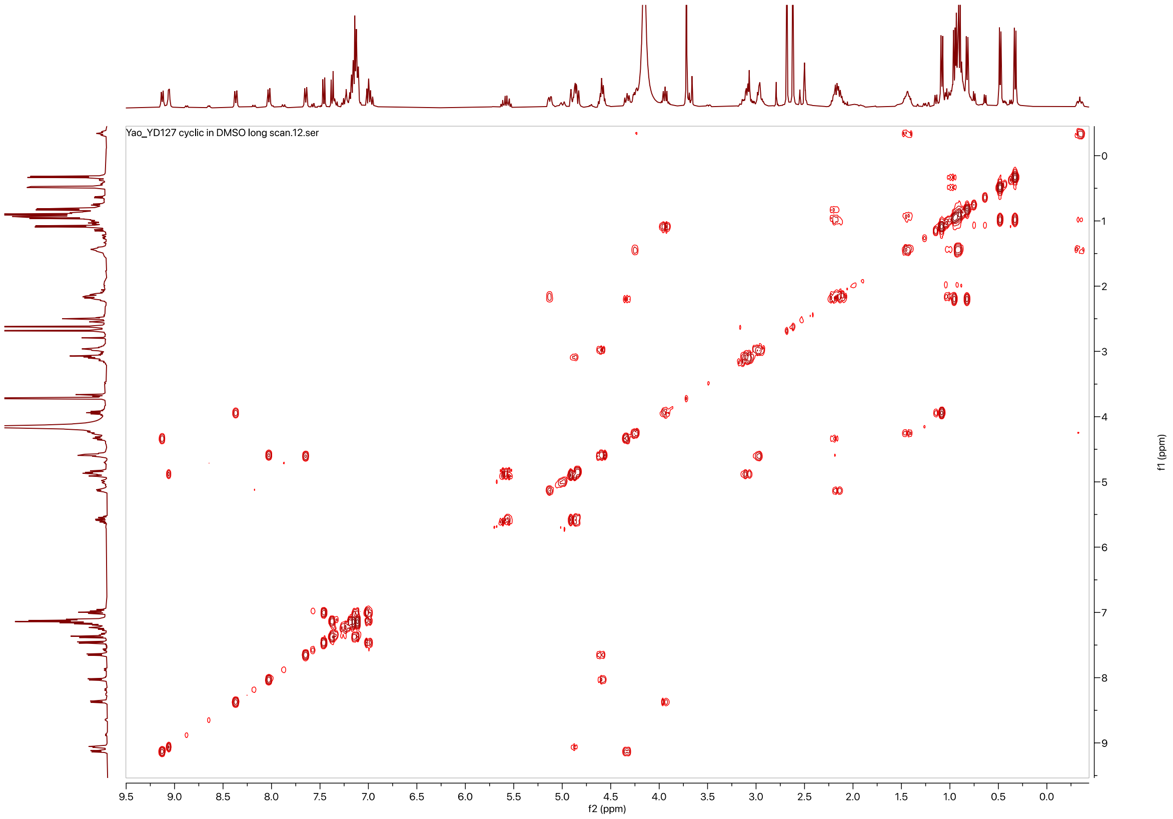

*HSQC spectrum of cyclomarin analogue **32c** in $(CD_3)_2SO$.*

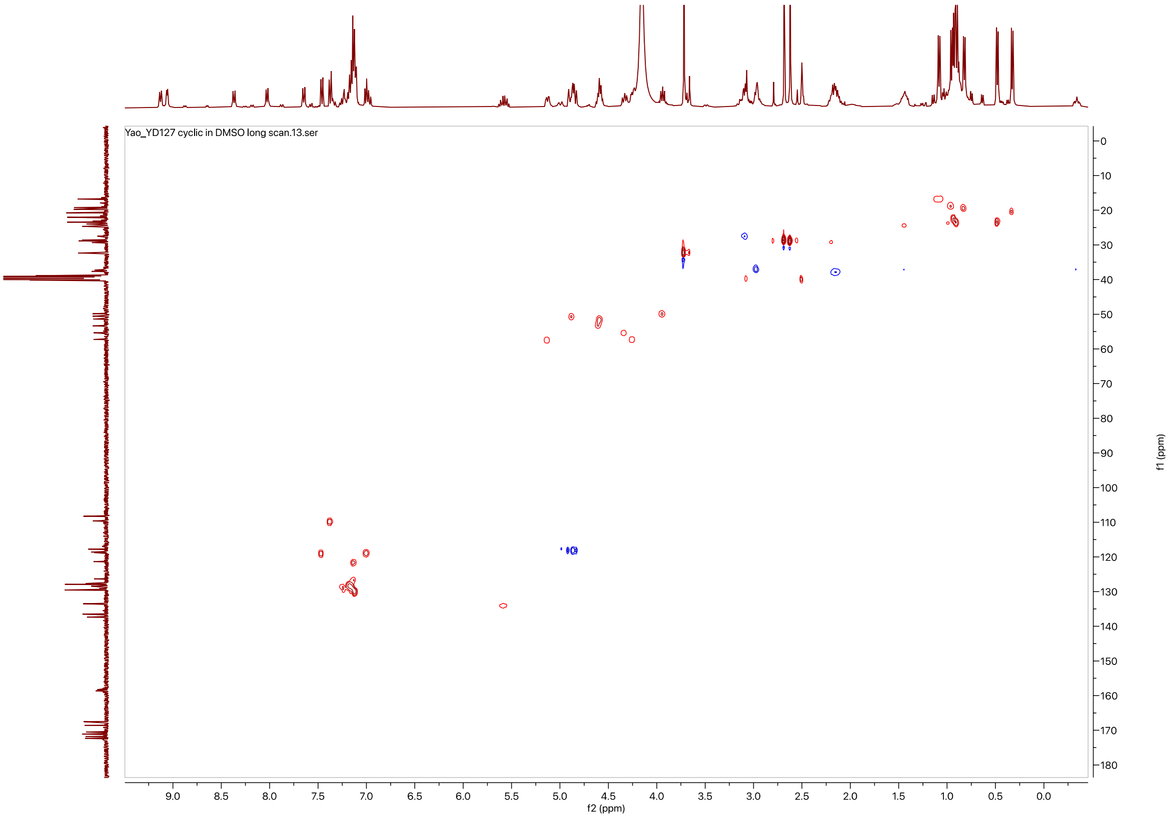

*HMBC spectrum of cyclomarin analogue **32c** in $(CD_3)_2SO$.*

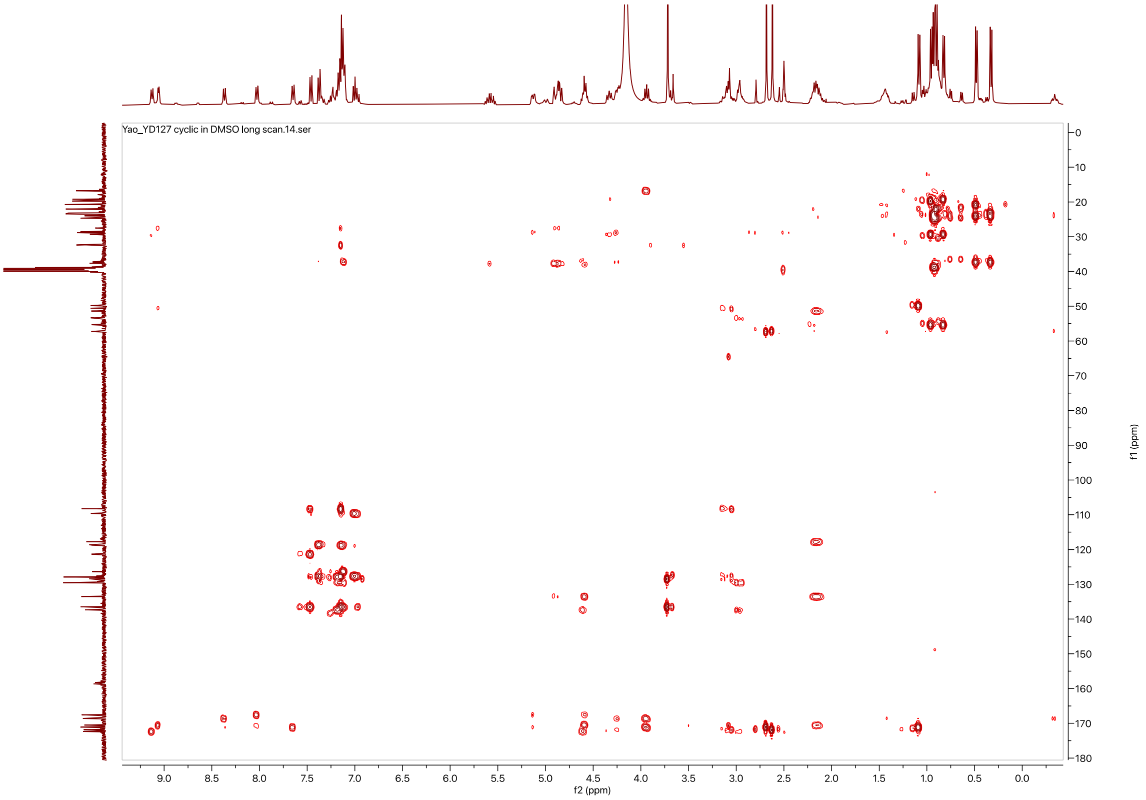

*^{1}H NMR spectrum of C5aR antagonist analogue **33c** in $(CD_3)_2SO$ at 400 MHz.*

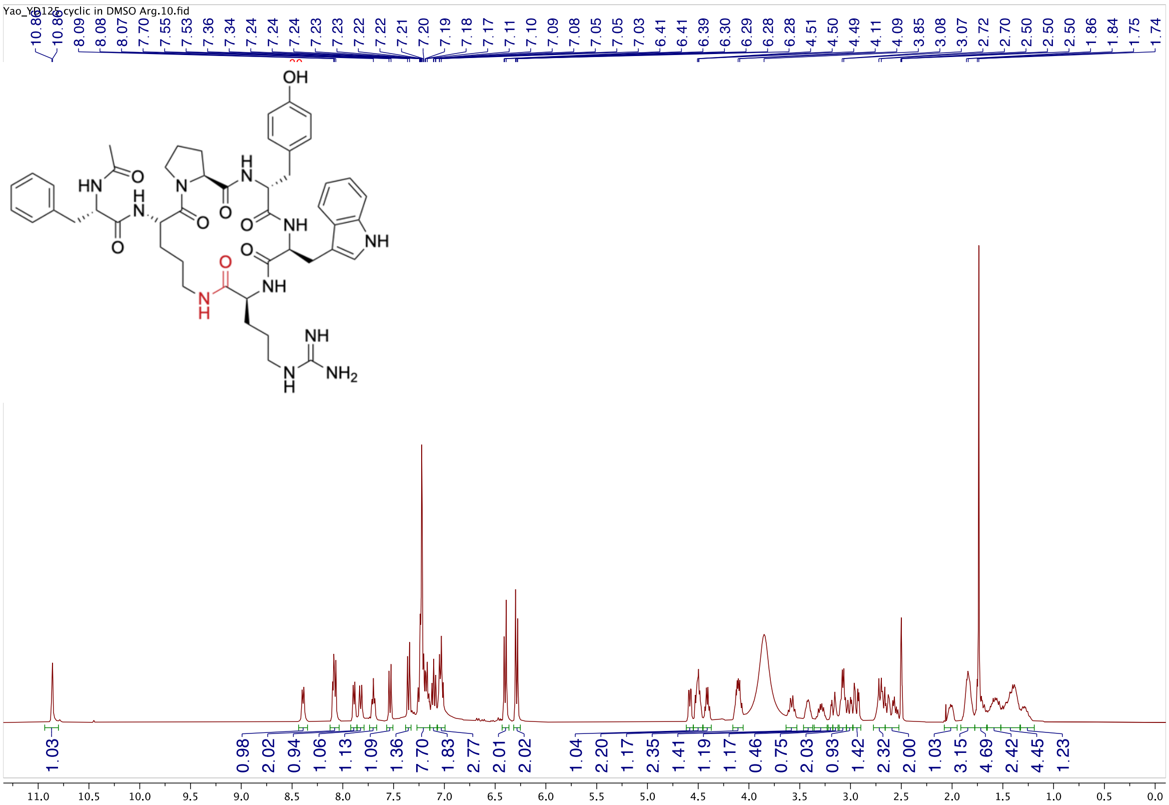

*^{13}C NMR spectrum of C5aR antagonist analogue **33c** in $(CD_3)_2SO$ at 101 MHz.*

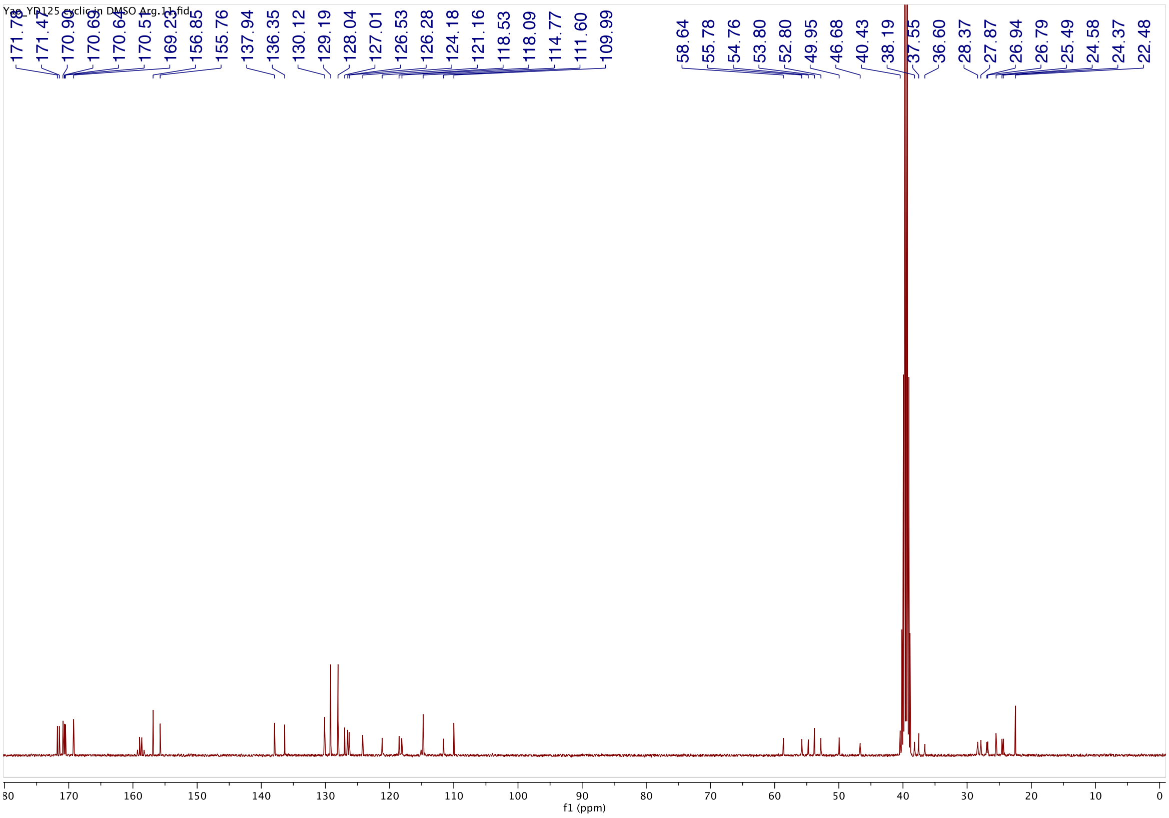

*COSY spectrum of C5aR antagonist analogue **33c** in $(CD_3)_2SO$ at 400 MHz.*

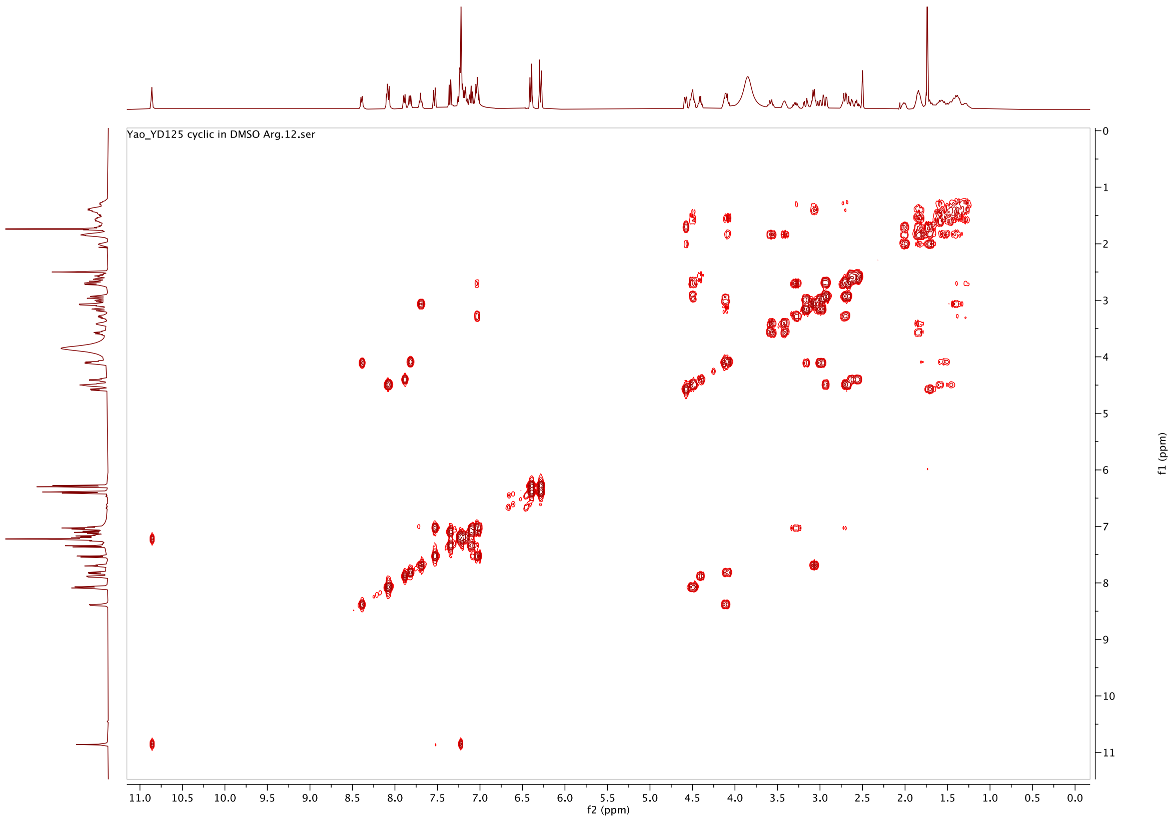

*HSQC spectrum of C5aR antagonist analogue **33c** in $(CD_3)_2SO$.*

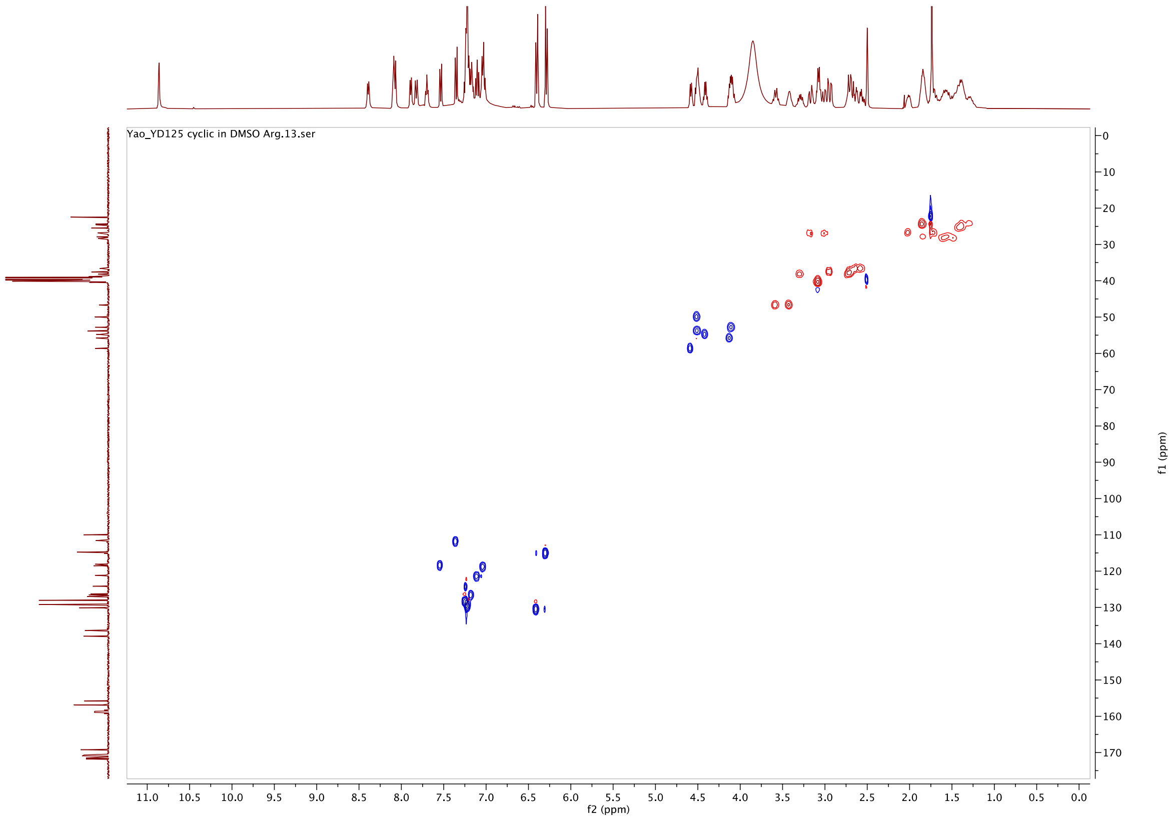

*HMBC spectrum of C5aR antagonist analogue **33c** in $(CD_3)_2SO$.*

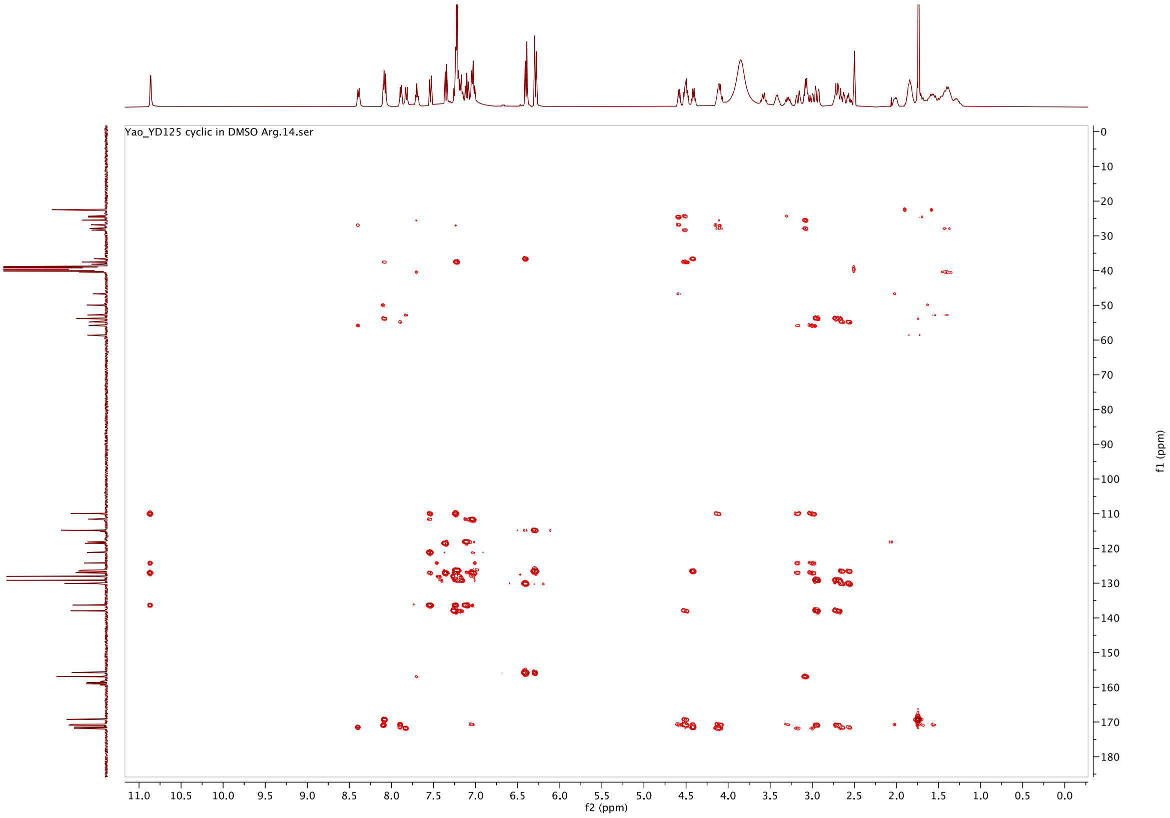

*^{1}H NMR spectrum of **9a** in (90% CD_3CN 10% D_2O) at 400 MHz.*

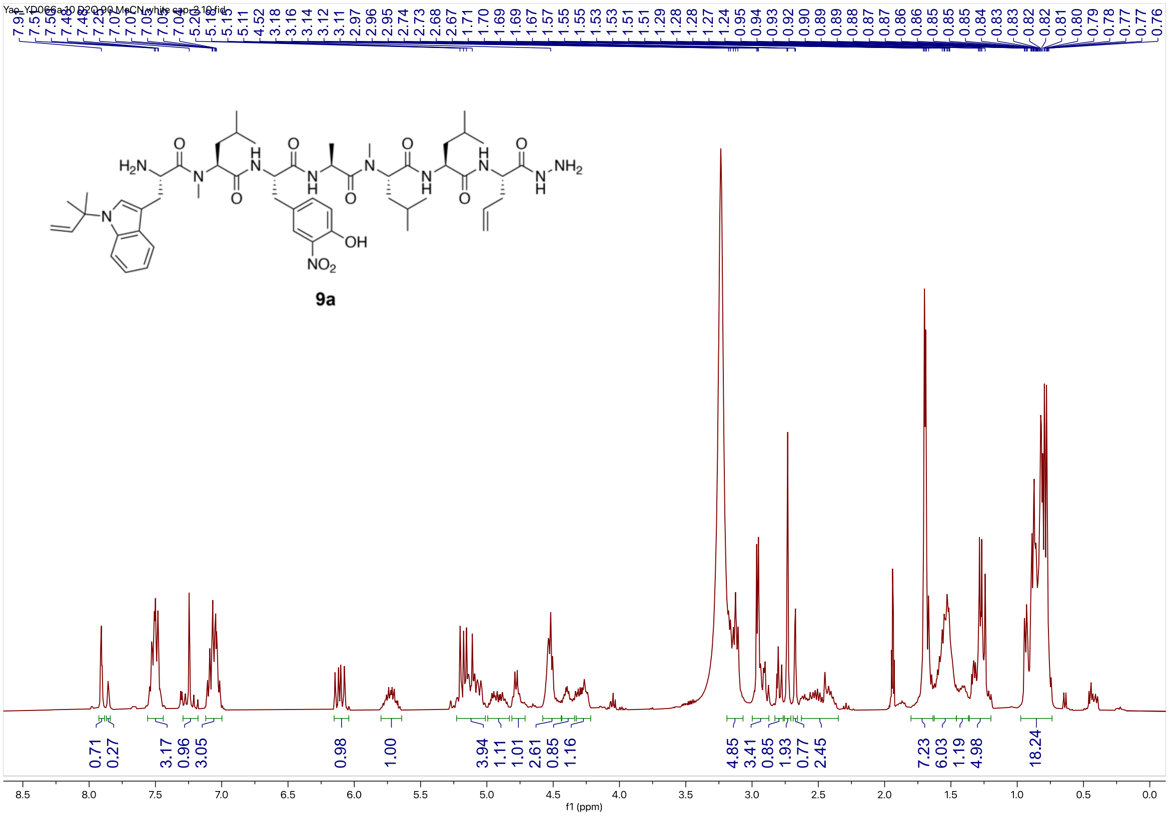

^{13}C NMR spectrum of **9a** in (90% CD_3CN 10% D_2O) at 101 MHz.

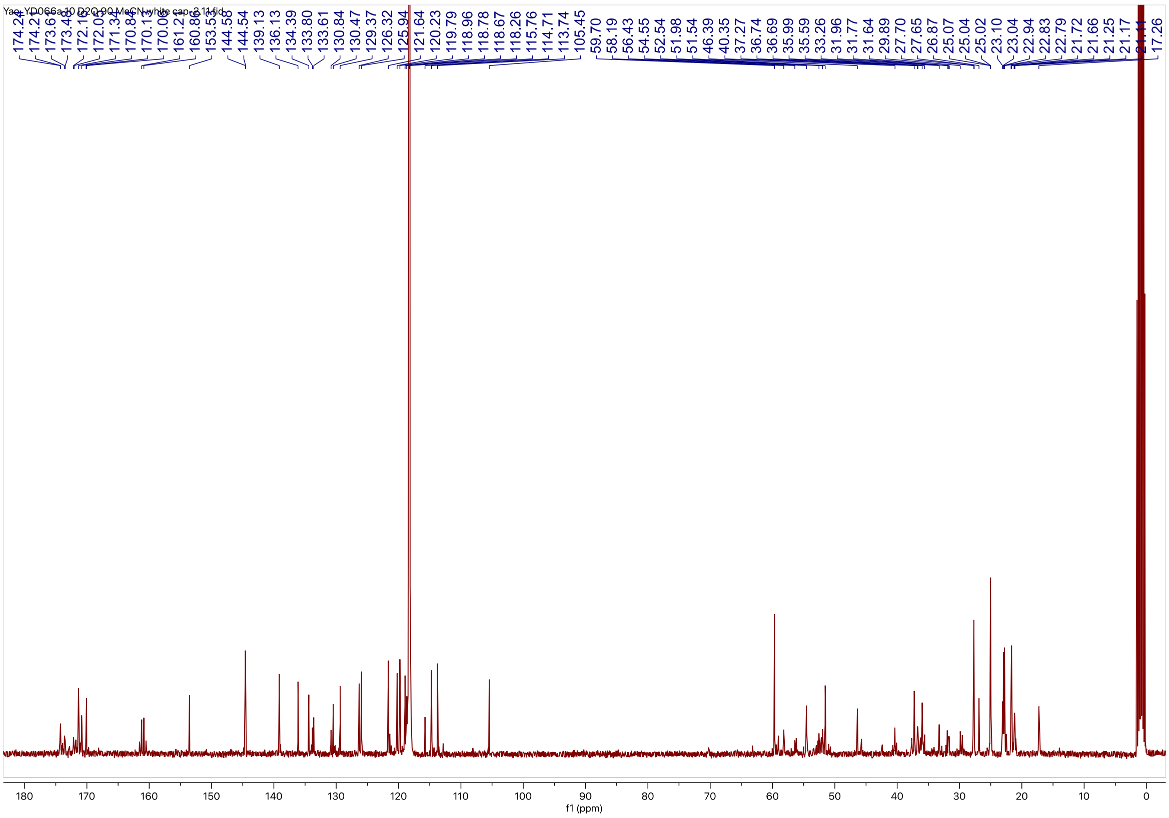

COSY spectrum of **9a** in (90% CD_3CN 10% D_2O) at 400 MHz.

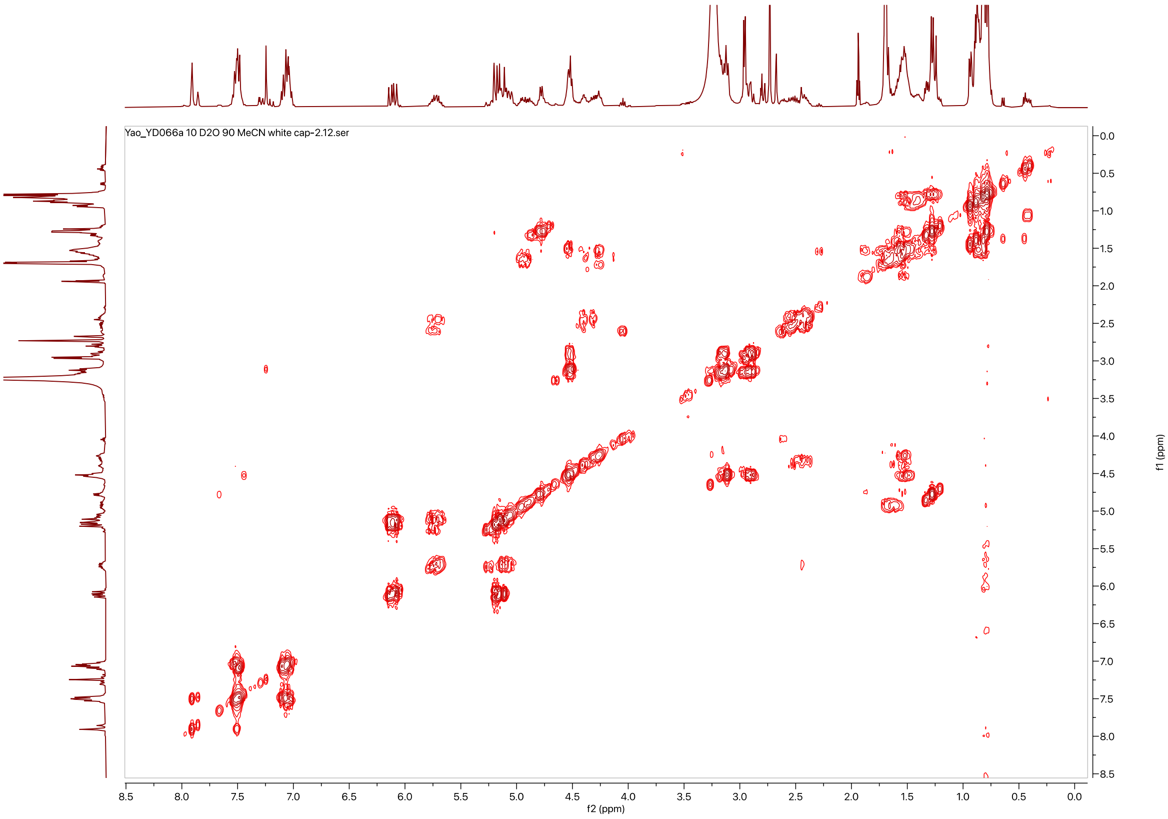

*HSQC spectrum of **9a** in (90% CD_3CN 10% D_2O).*

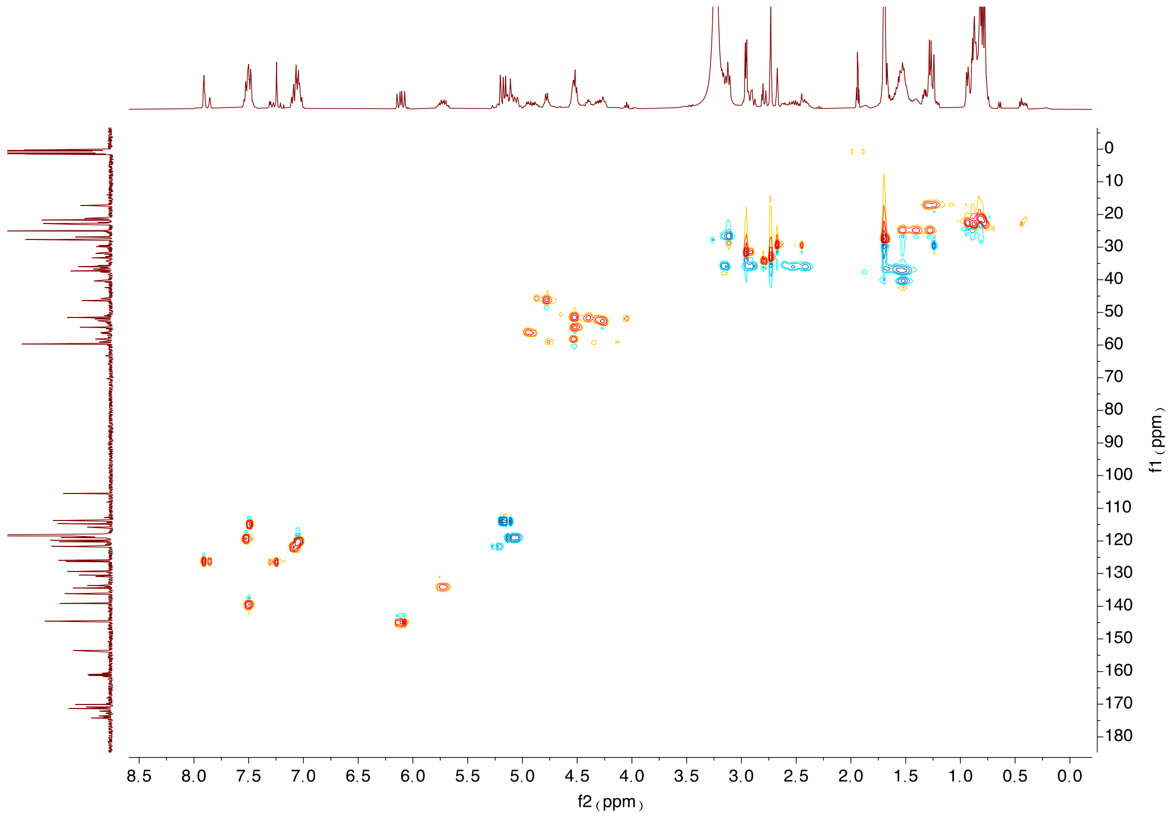

*HMBC spectrum of **9a** in (90% CD_3CN 10% D_2O).*

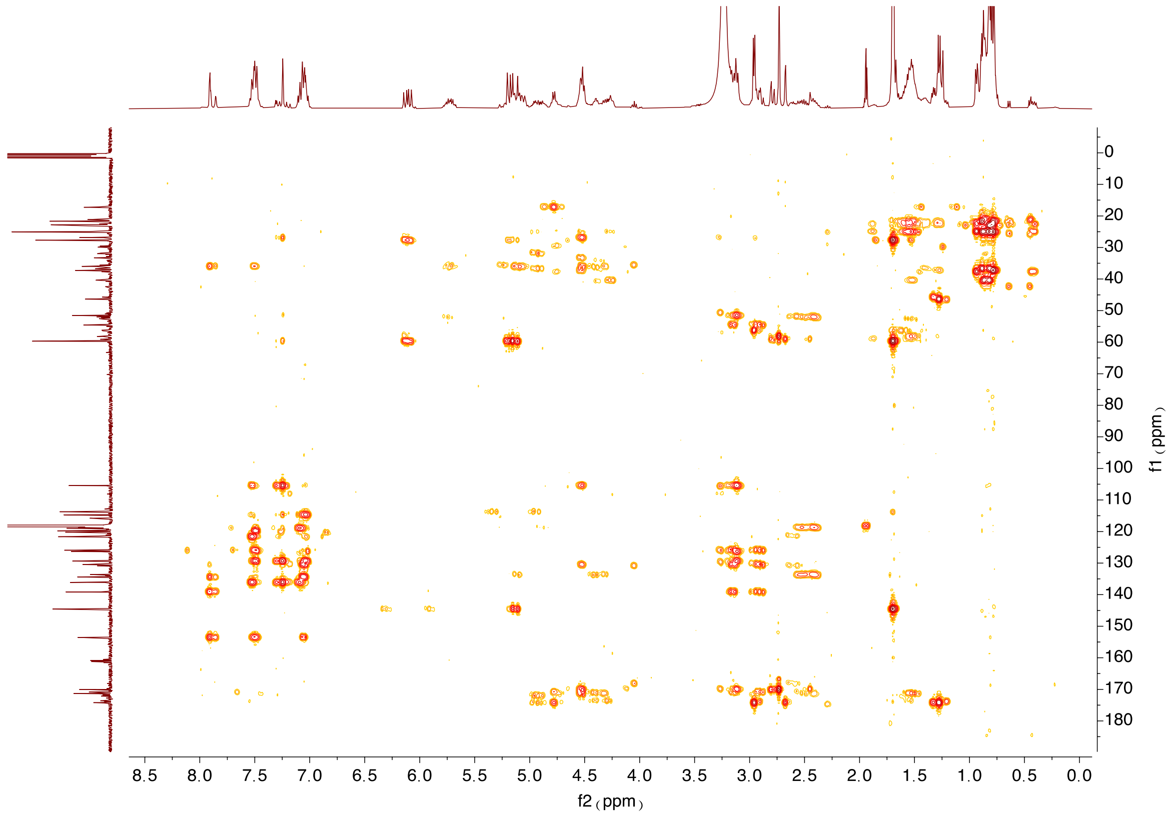

*ROESY spectrum of **9a** in (90% CD_3CN 10% D_2O) at 400 MHz.*

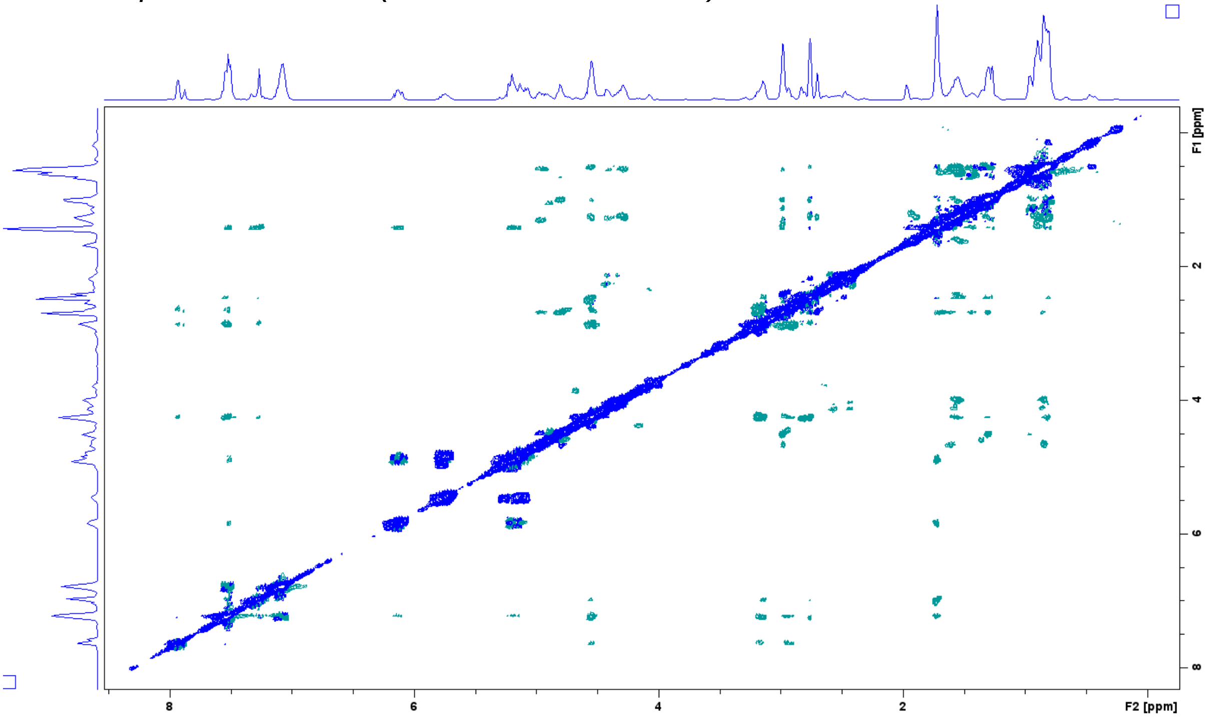

*^{1}H NMR spectrum of **14a** in (90% CD_3CN 10% D_2O) at 400 MHz.*

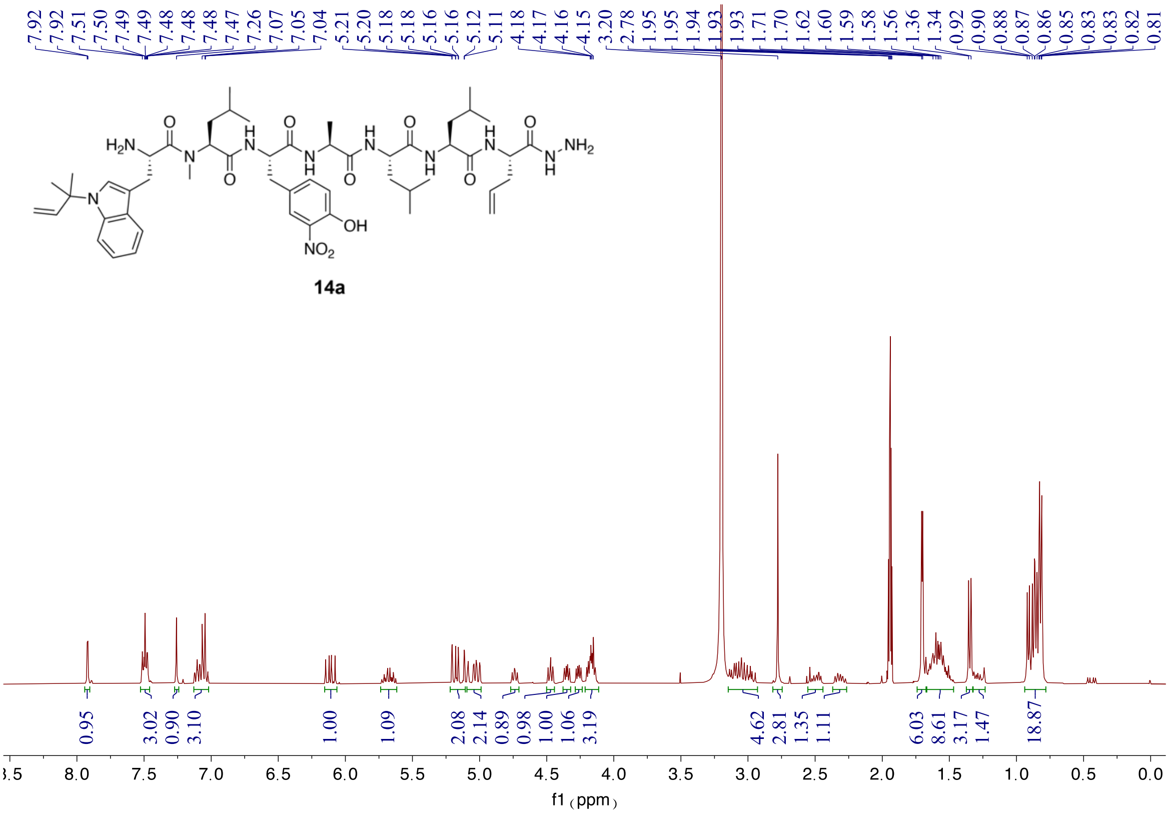

^{13}C NMR spectrum of **14a** in (90% CD_3CN 10% D_2O) at 101 MHz.

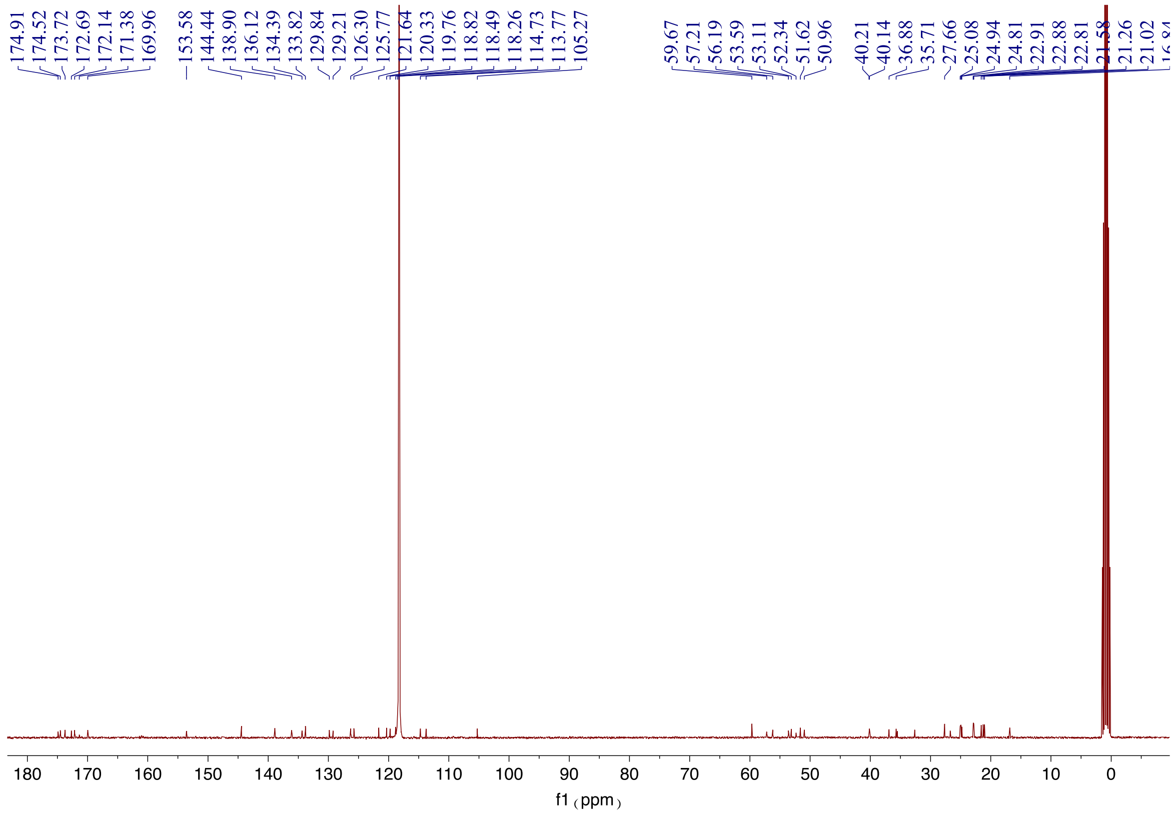

COSY spectrum of **14a** in (90% CD_3CN 10% D_2O) at 400 MHz.

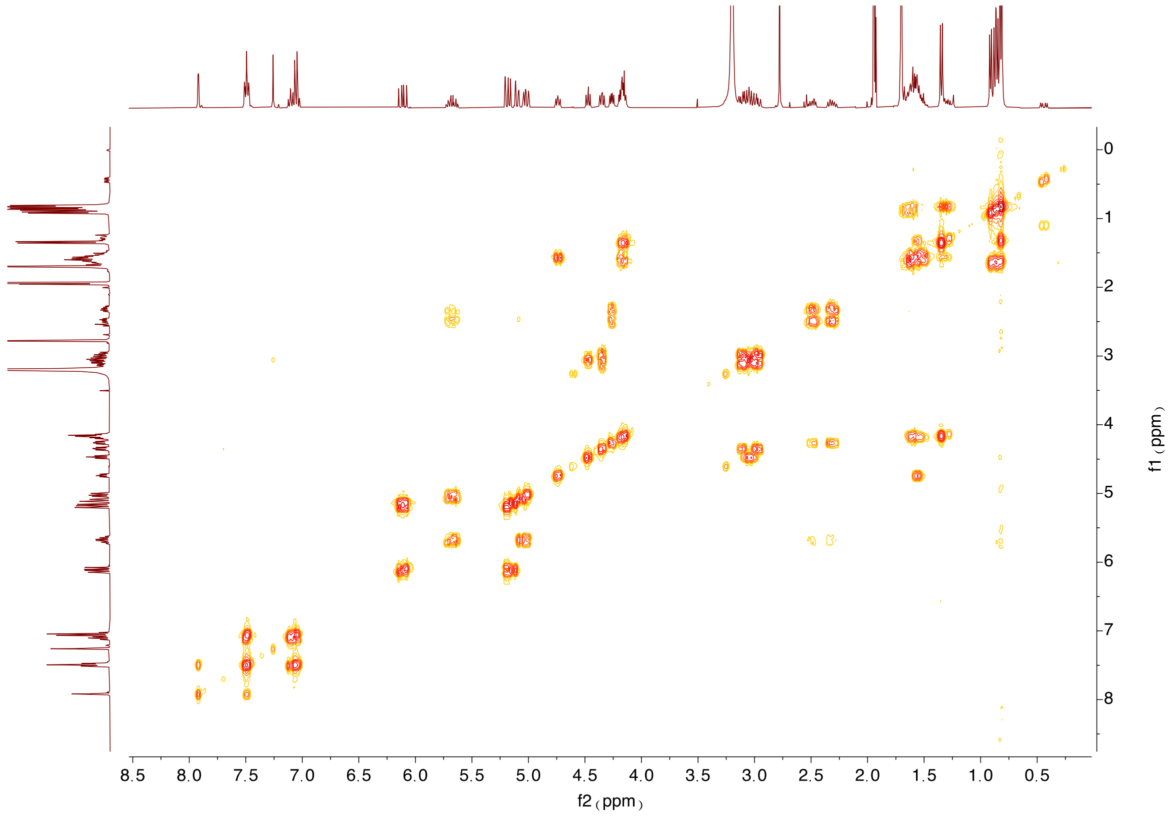

*HSQC spectrum of **14a** in (90% CD_3CN 10% D_2O).*

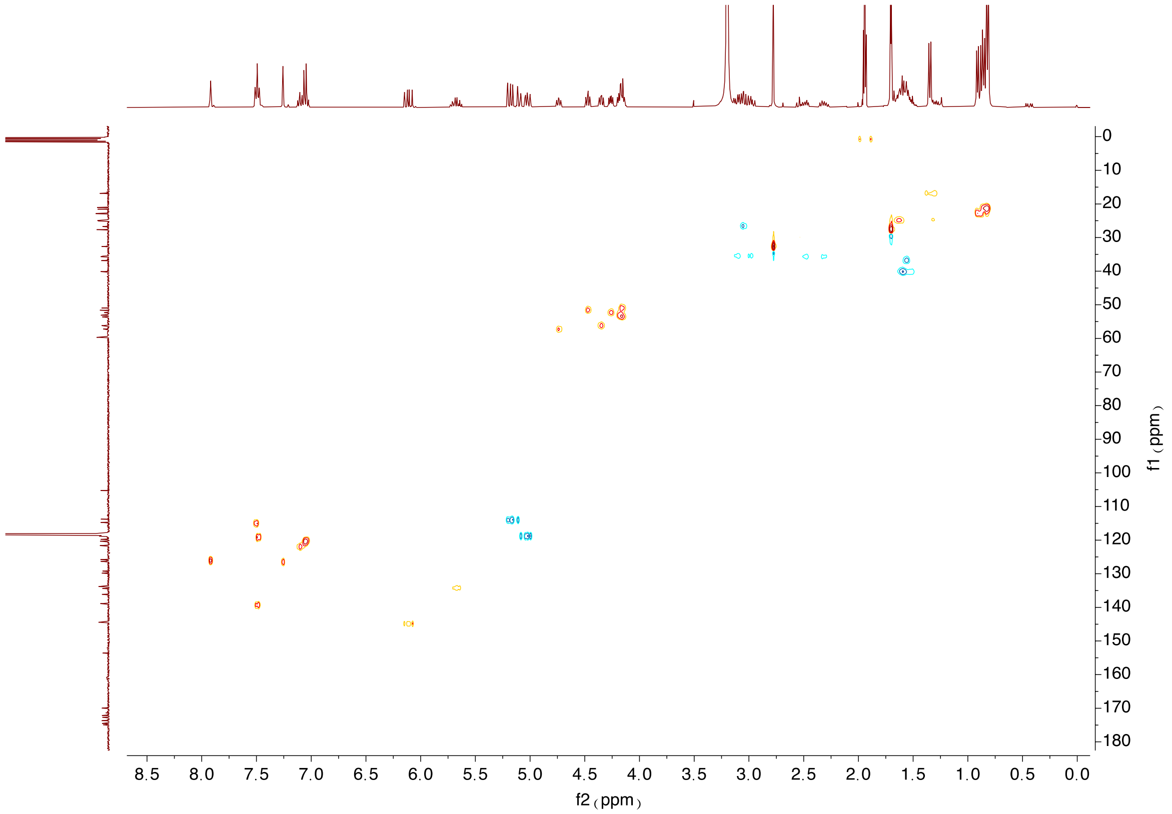

*HMBC spectrum of **14a** in (90% CD_3CN 10% D_2O).*

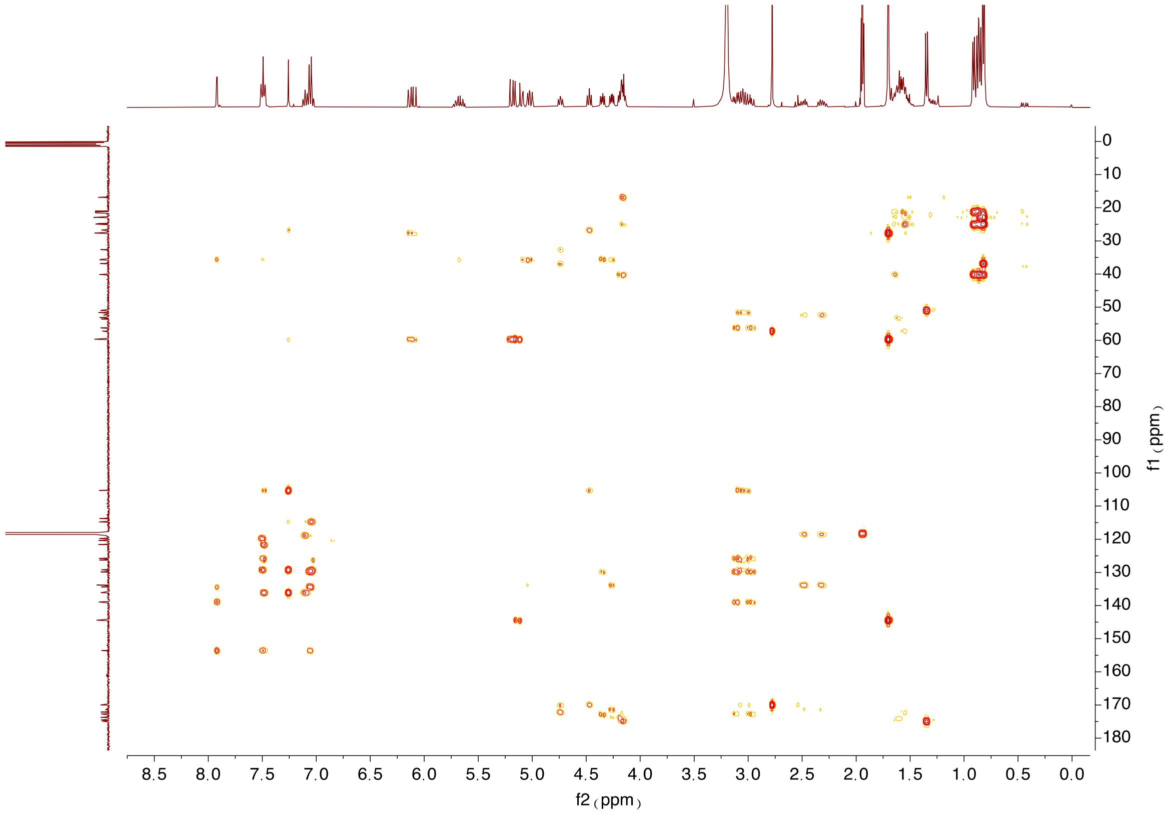

*ROESY spectrum of **14a** in (90% CD_3CN 10% D_2O) at 400 MHz.*

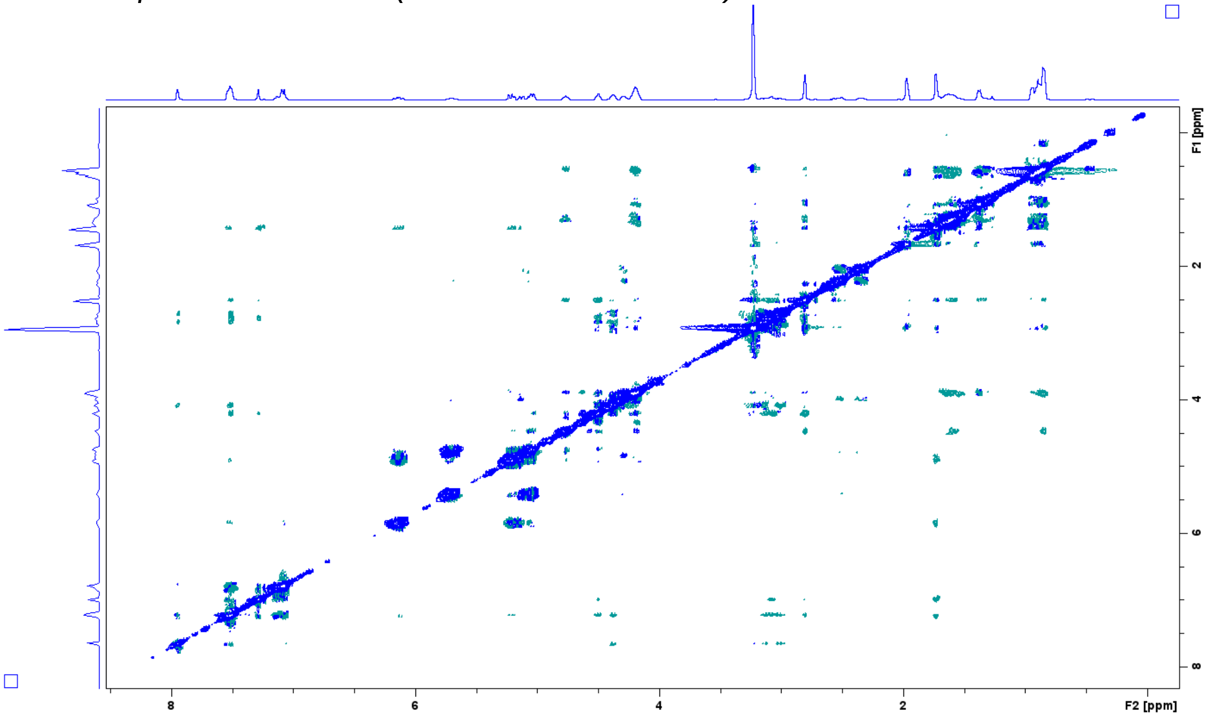

*^{1}H NMR spectrum of branched surugamide B **34c** in $(CD_3)_2SO$ at 400 MHz.*

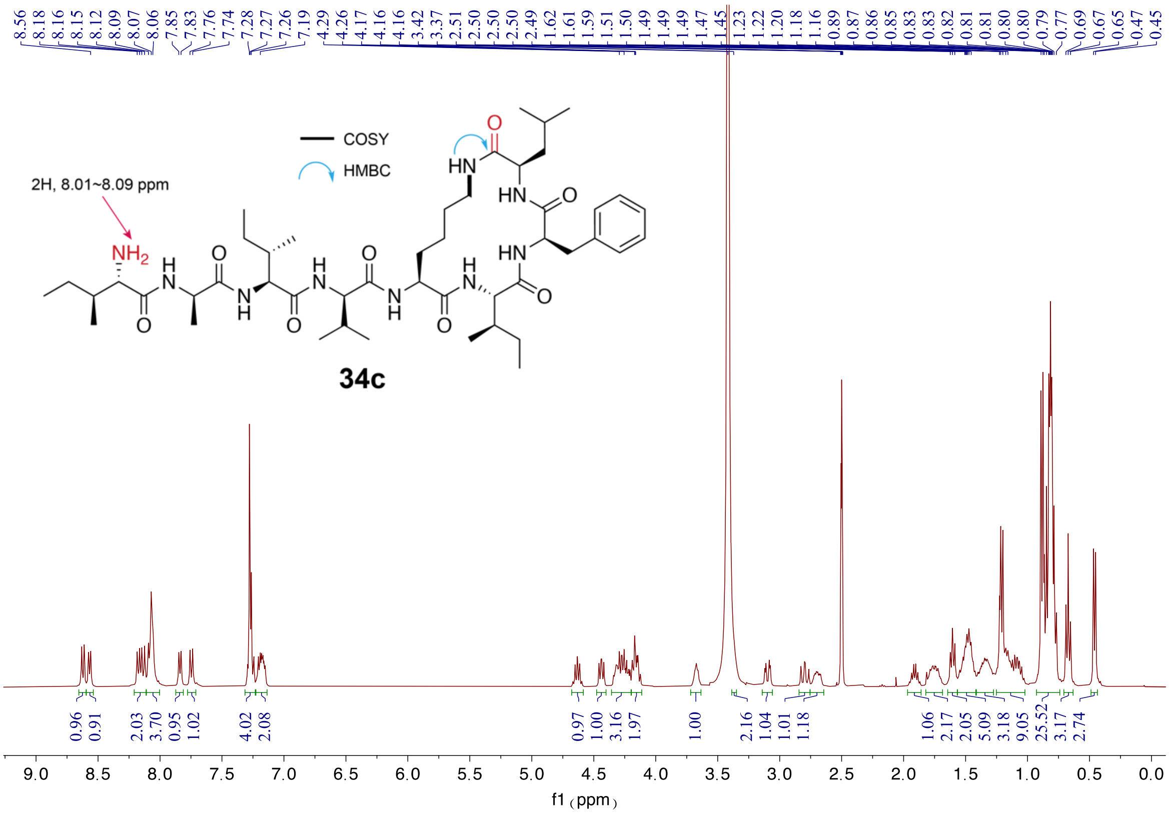

*^{13}C NMR spectrum of branched surugamide B **34c** in $(CD_3)_2SO$ at 100 MHz.*

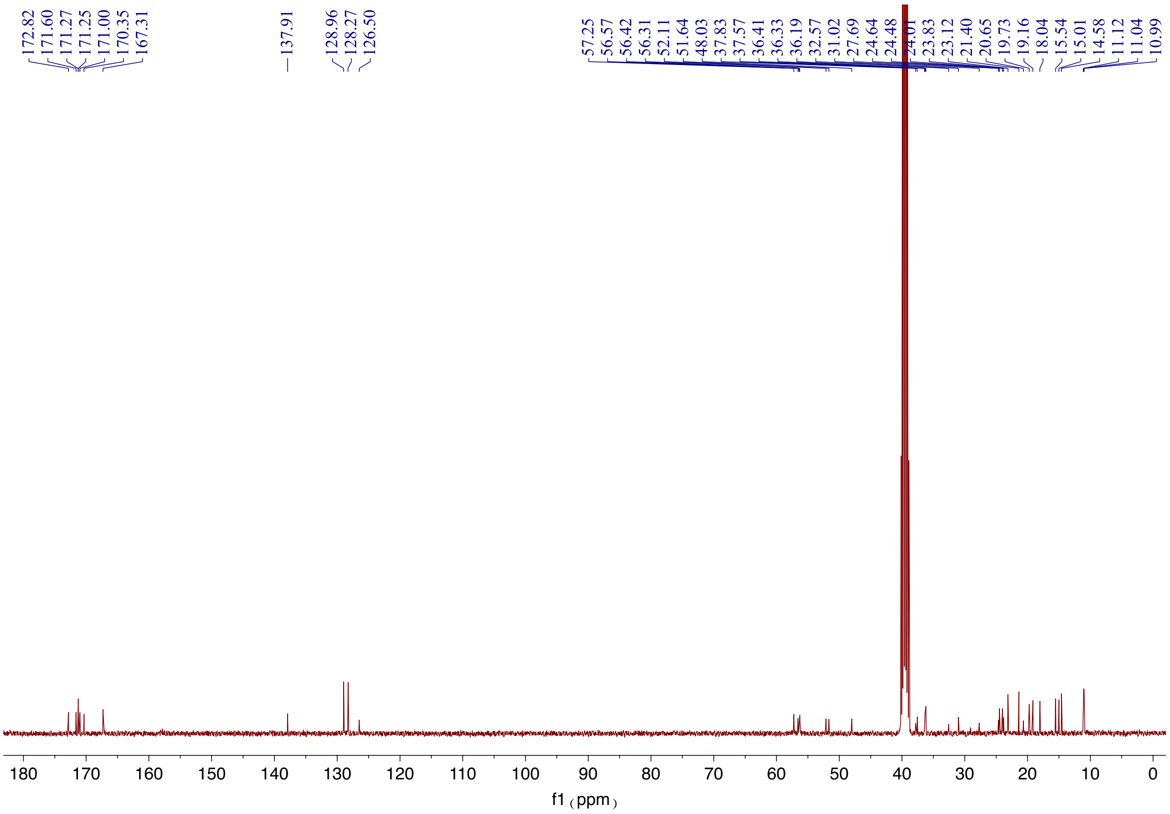

*COSY spectrum of branched surugamide B **34c** in $(CD_3)_2SO$ at 400 MHz.*

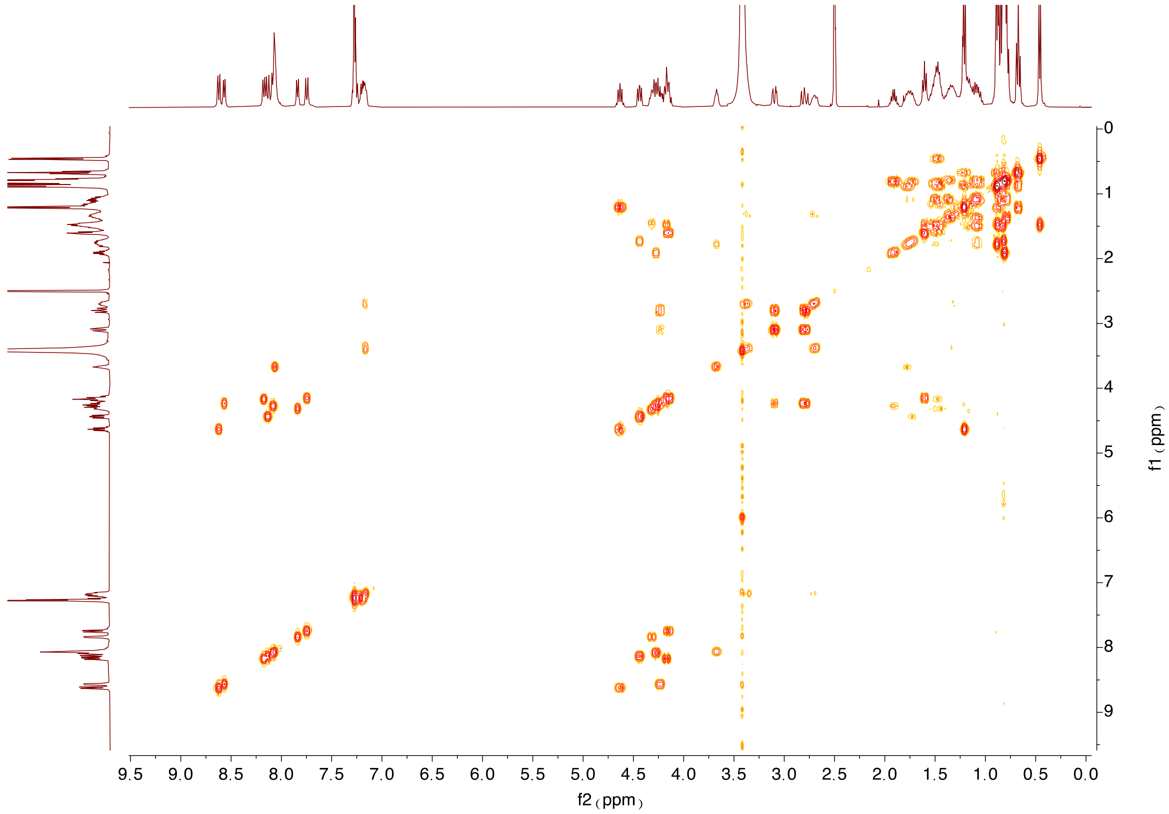

*HSQC spectrum of branched surugamide B **34c** in $(CD_3)_2SO$.*

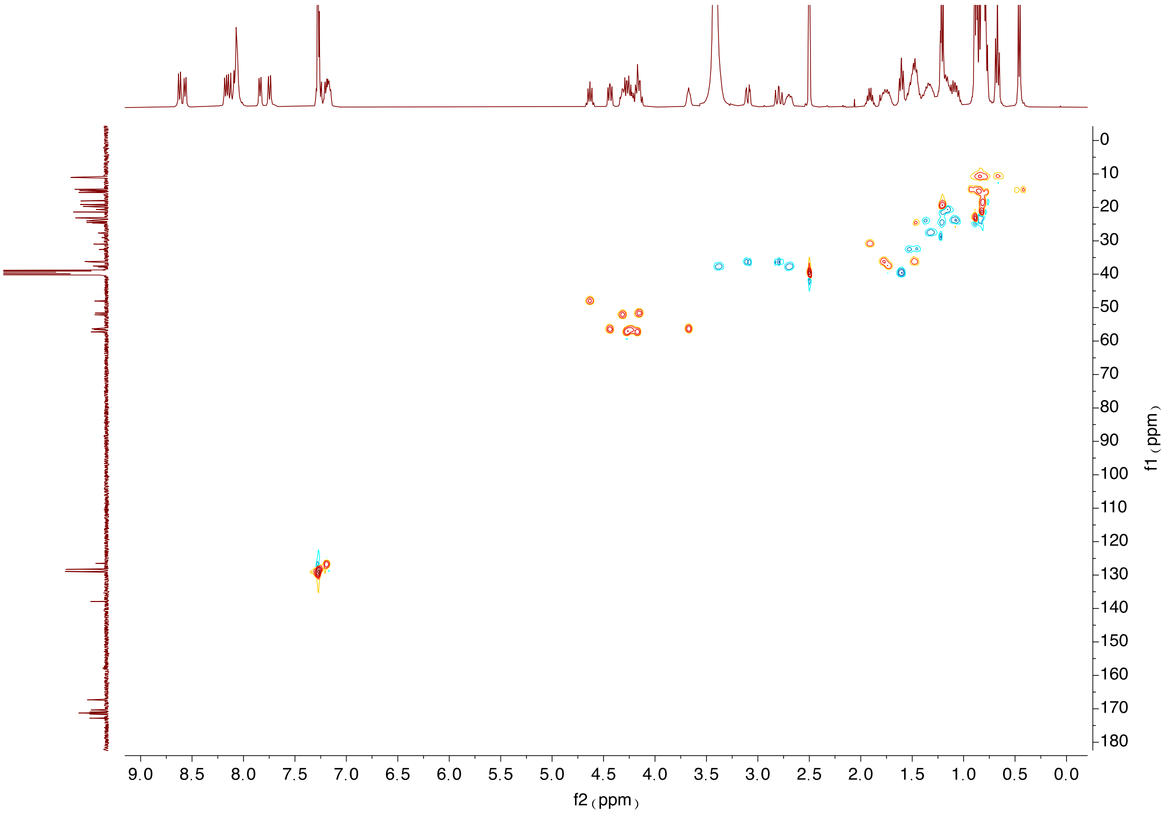

*HMBC spectrum of branched surugamide B **34c** in $(CD_3)_2SO$.*

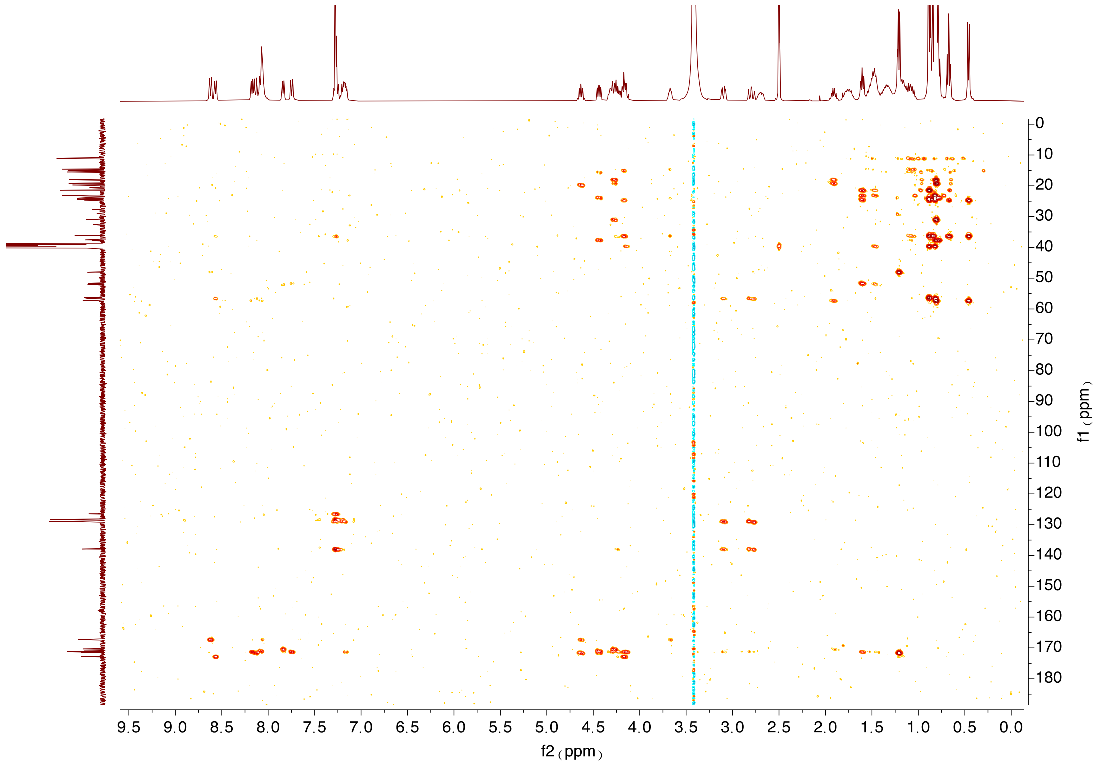

*^{1}H NMR spectrum of azido-surugamide **35c** in $(CD_3)_2SO$ at 700 MHz.*

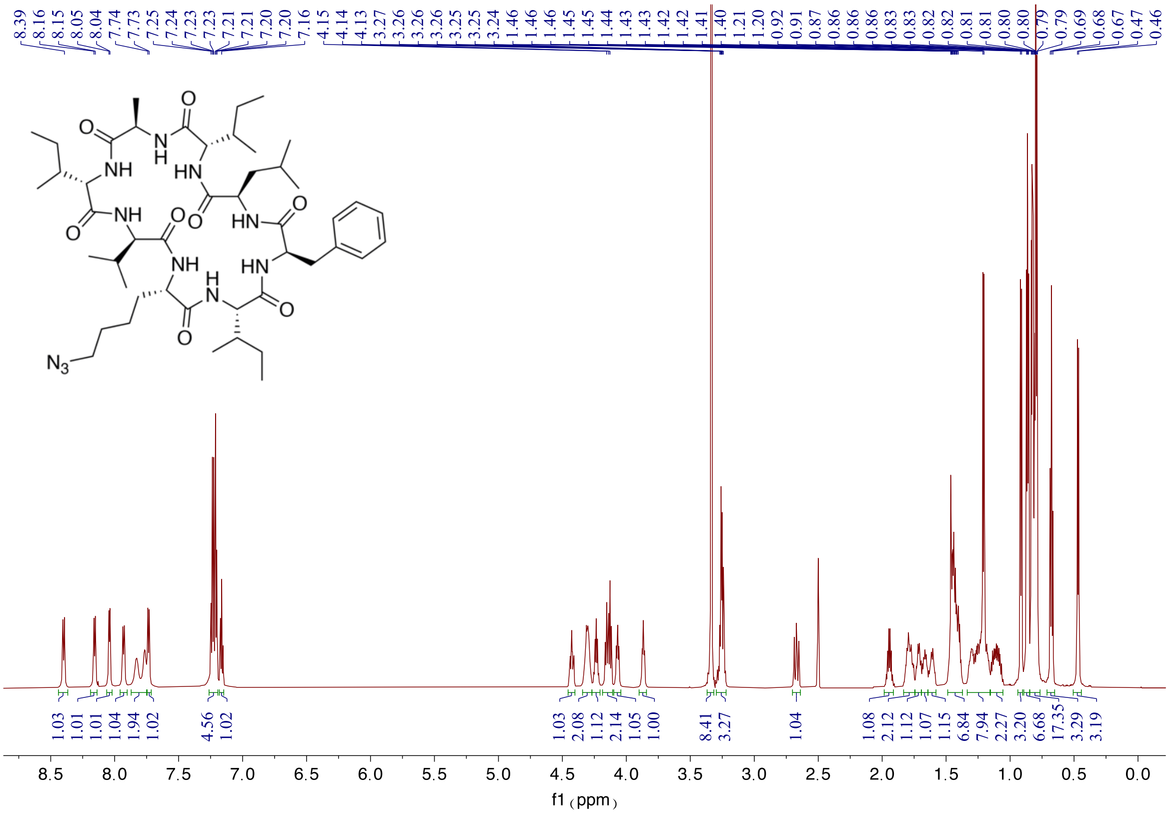

*^{13}C NMR spectrum of azido-surugamide **35c** in $(CD_3)_2SO$ at 176 MHz.*

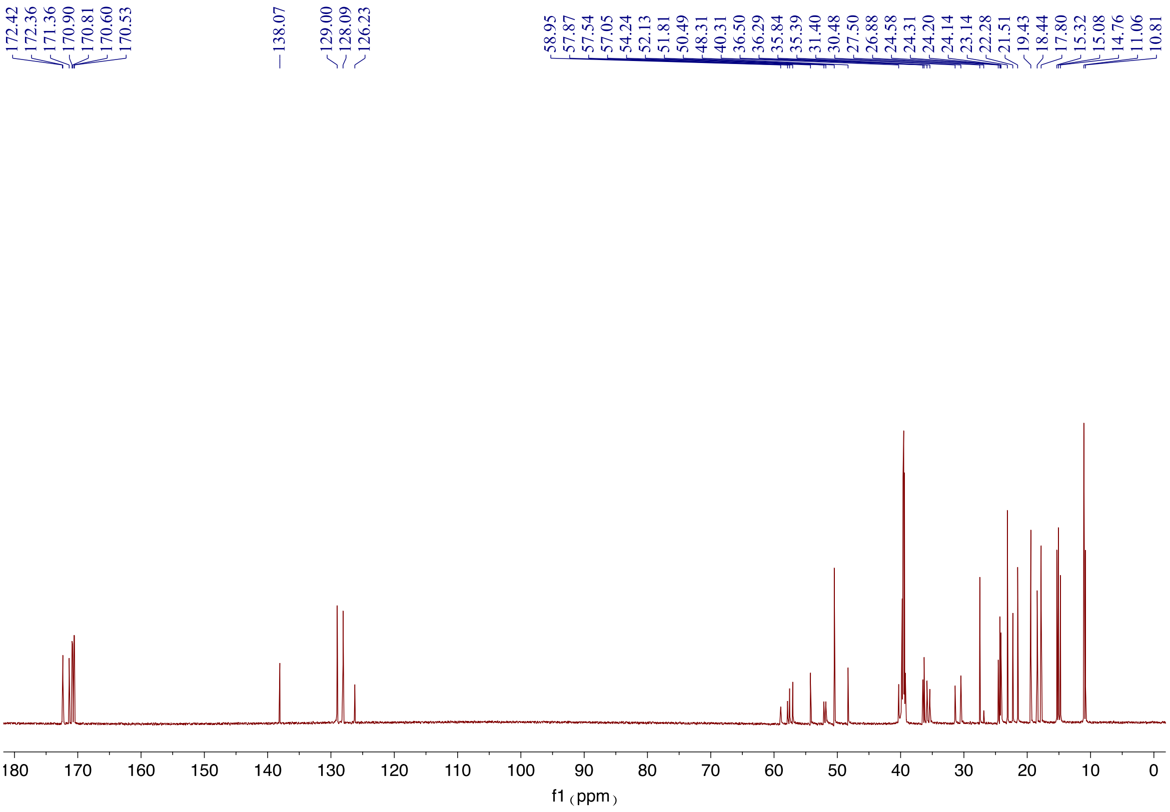

*COSY spectrum of azido-surugamide analogue **35a** in $(CD_3)_2SO$ at 700 MHz.*

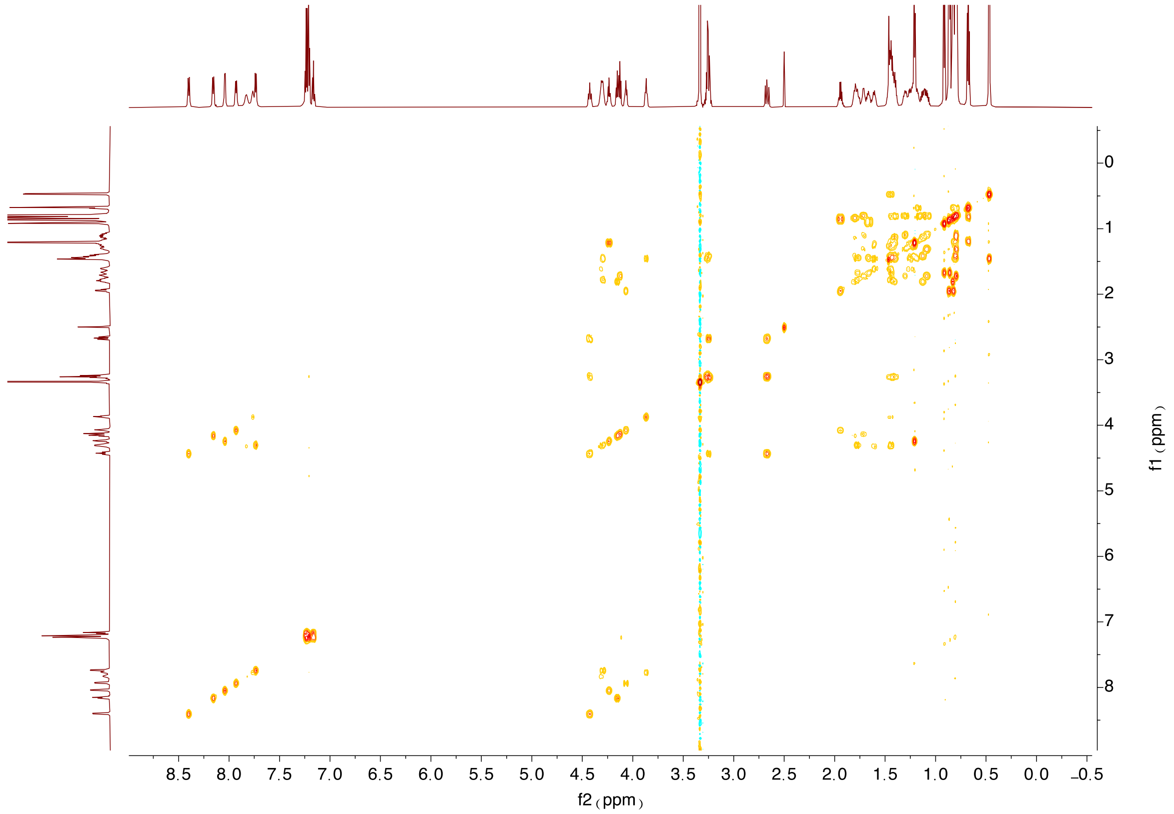

*HSQC spectrum of azido-surugamide **35c** in $(CD_3)_2SO$.*

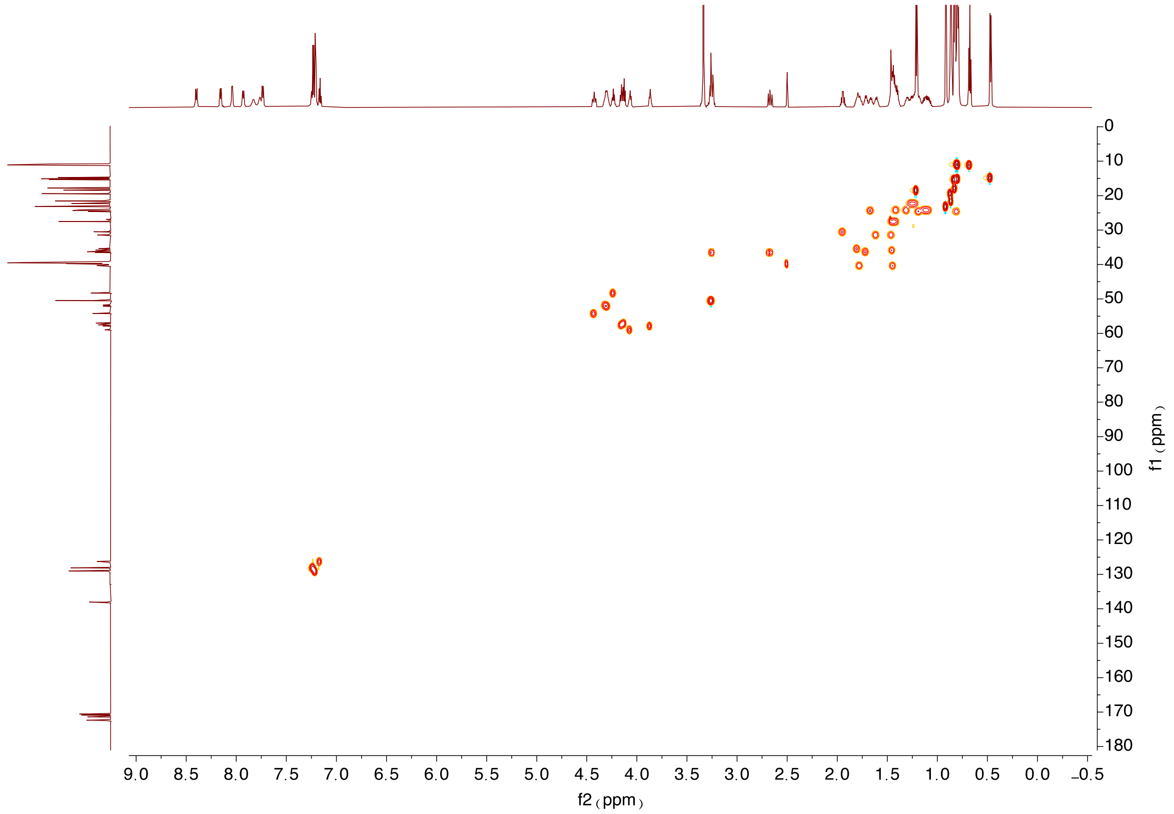

*HMBC spectrum of azido-surugamide **35c** in $(CD_3)_2SO$.*

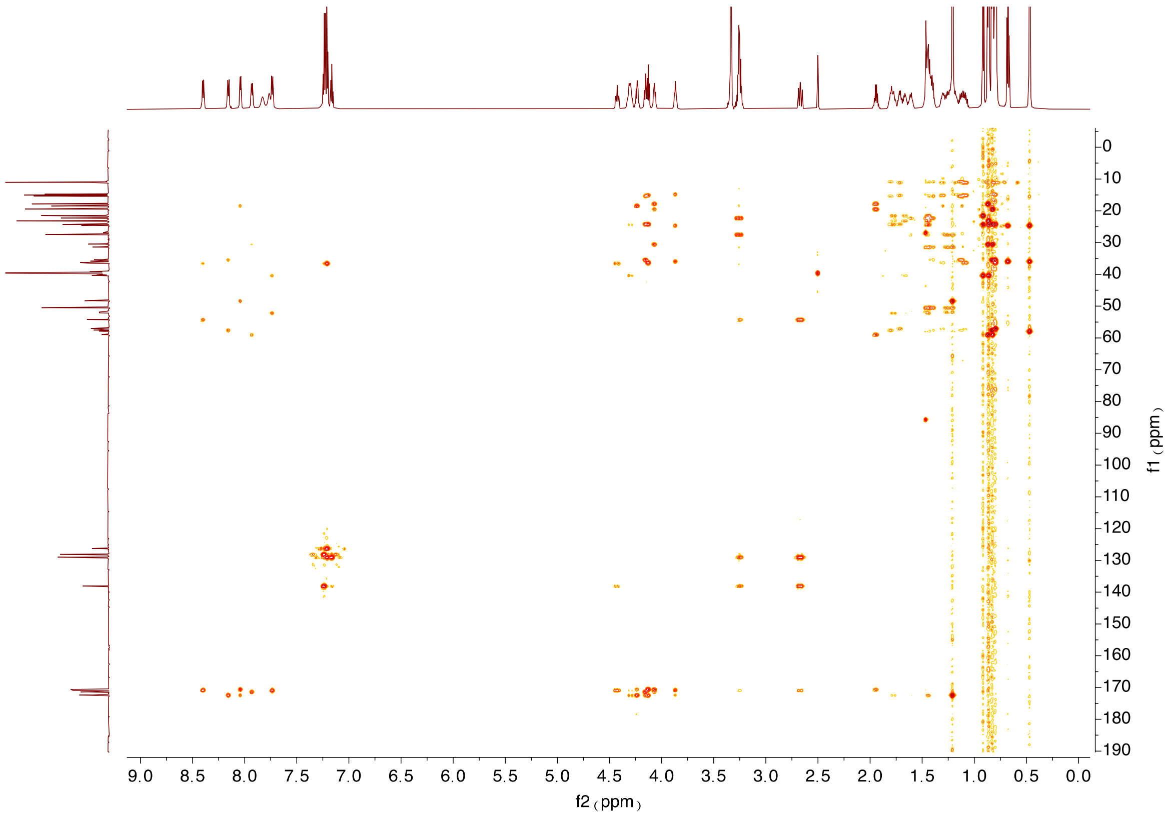

*ROESY spectrum of azido-surugamide **35c** in $(CD_3)_2SO$ at 700 MHz.*

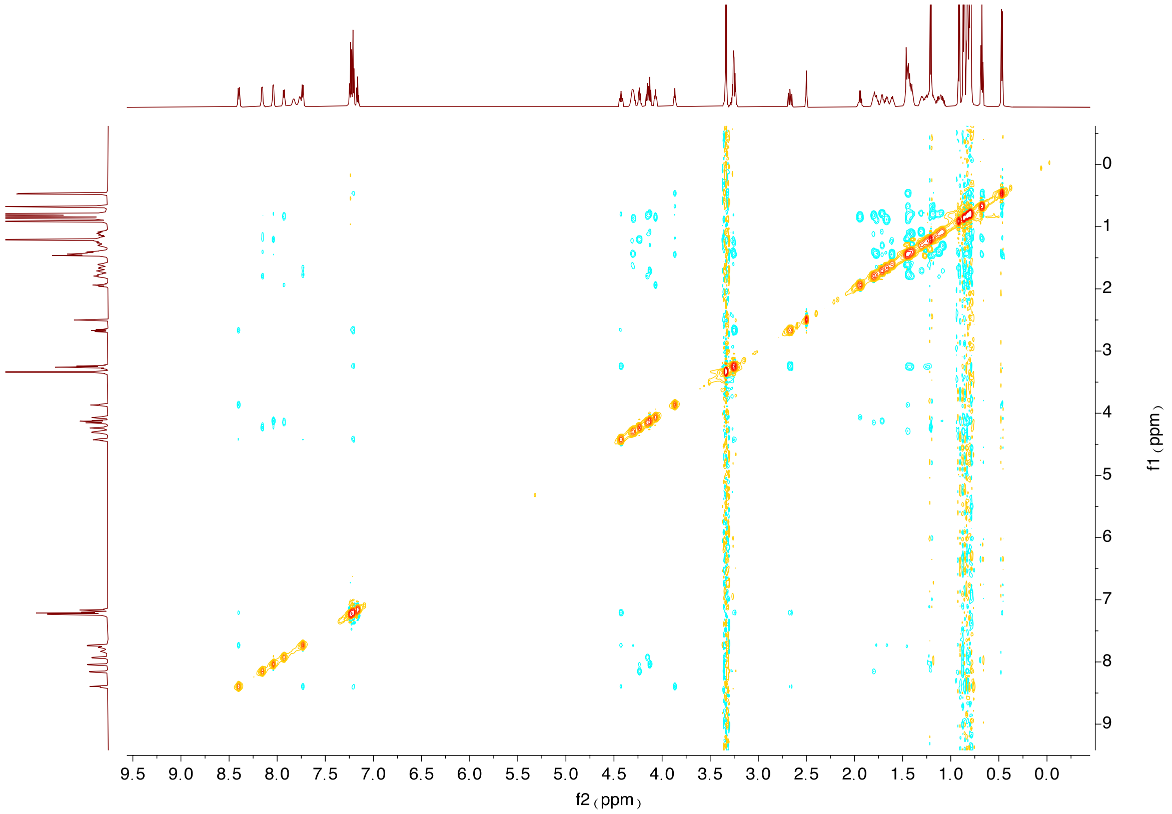

3.2 High Resolution Mass Spectra and Fragmentation of Peptides

HRMS spectra for peptide ***8a*** *(predicted mass spectrum (top) measured (bottom))*

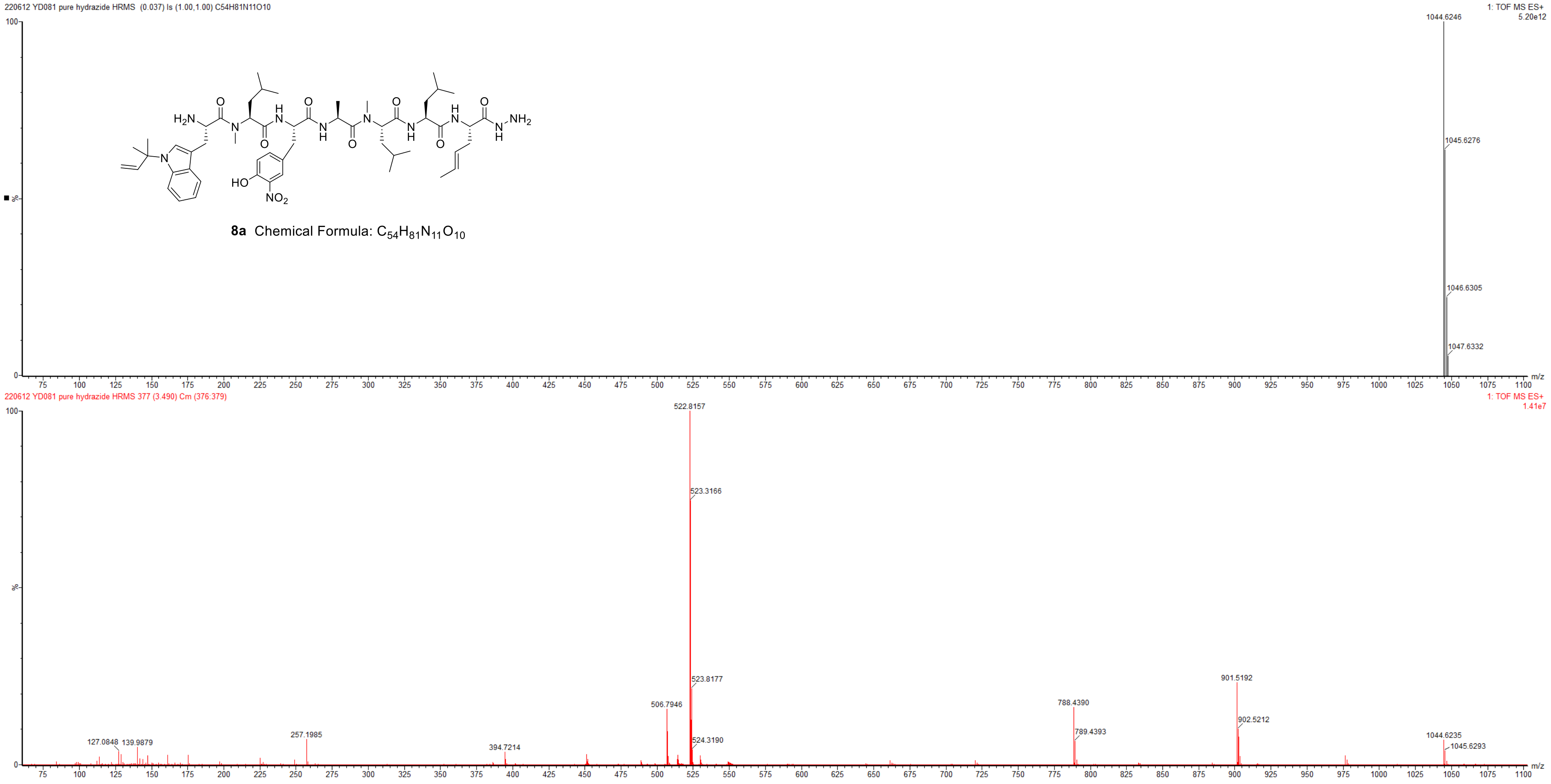

*MS^E spectrum for peptide **8a***

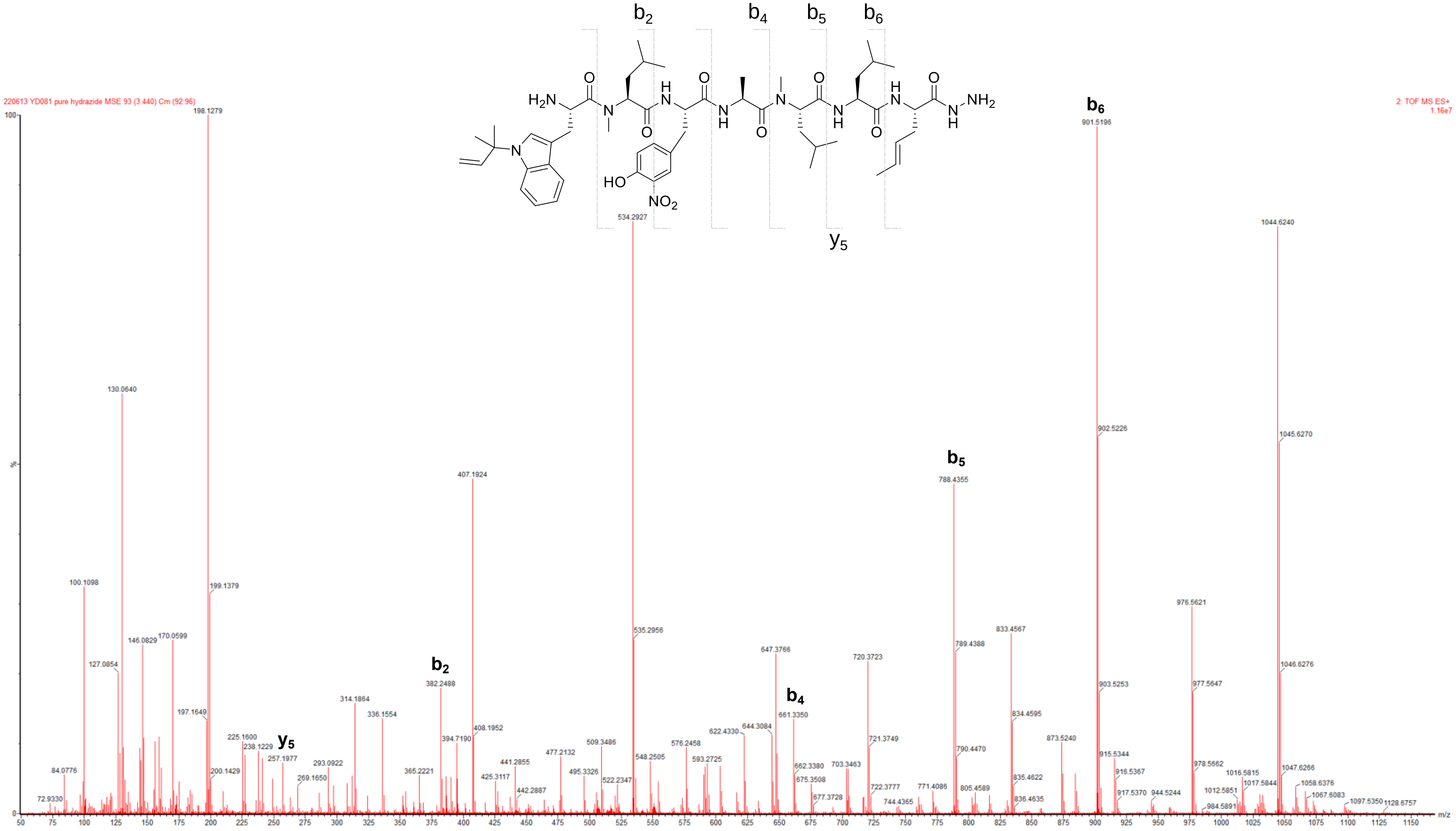

*HRMS spectra for peptide **9a** (predicted mass spectrum (top) measured (bottom))*

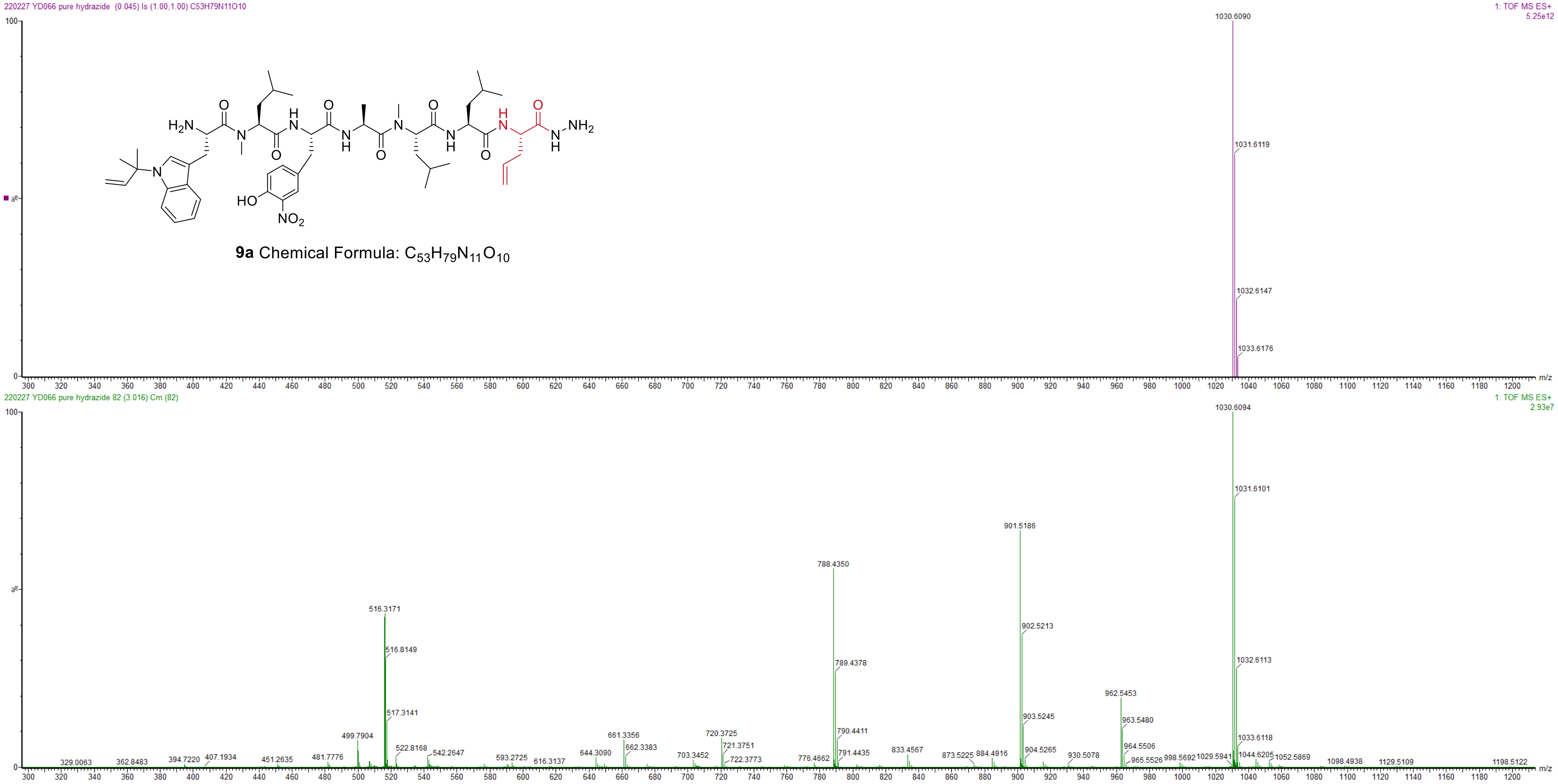

*MS^E spectrum for peptide **9a***

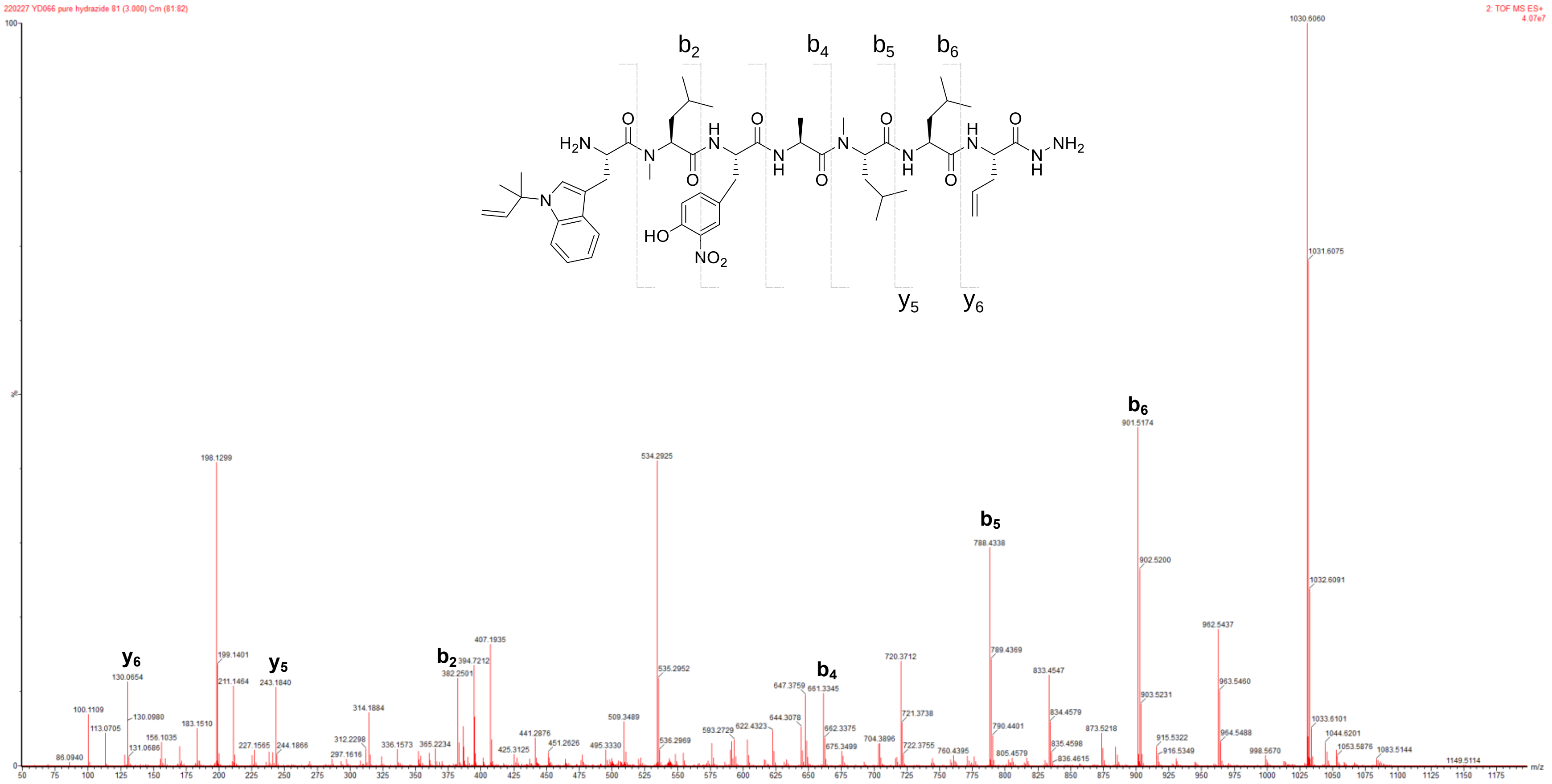

*HRMS spectra for peptide **9b** (predicted mass spectrum (top) measured (bottom))*

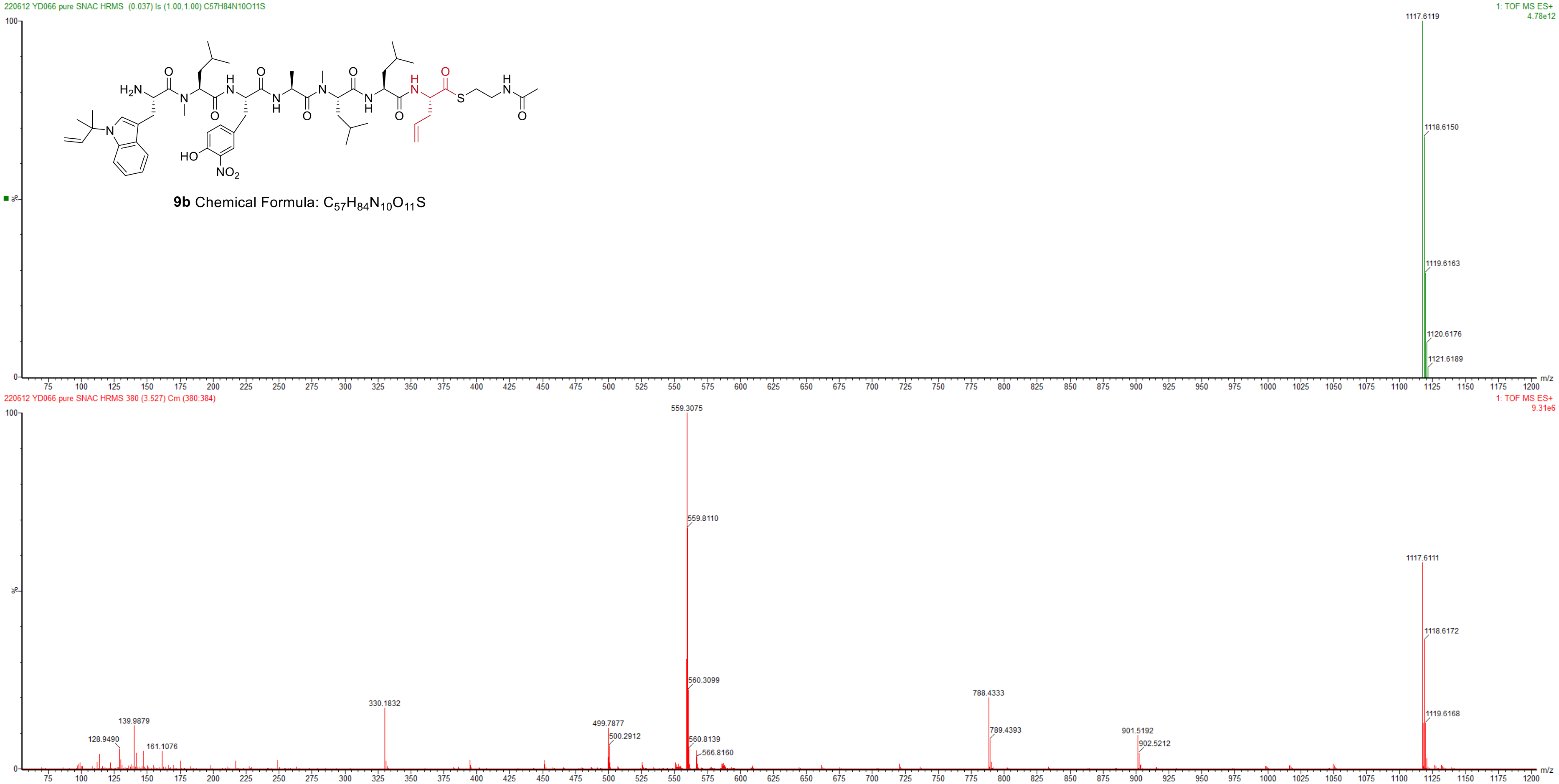

*MS^E spectrum for peptide **9b***

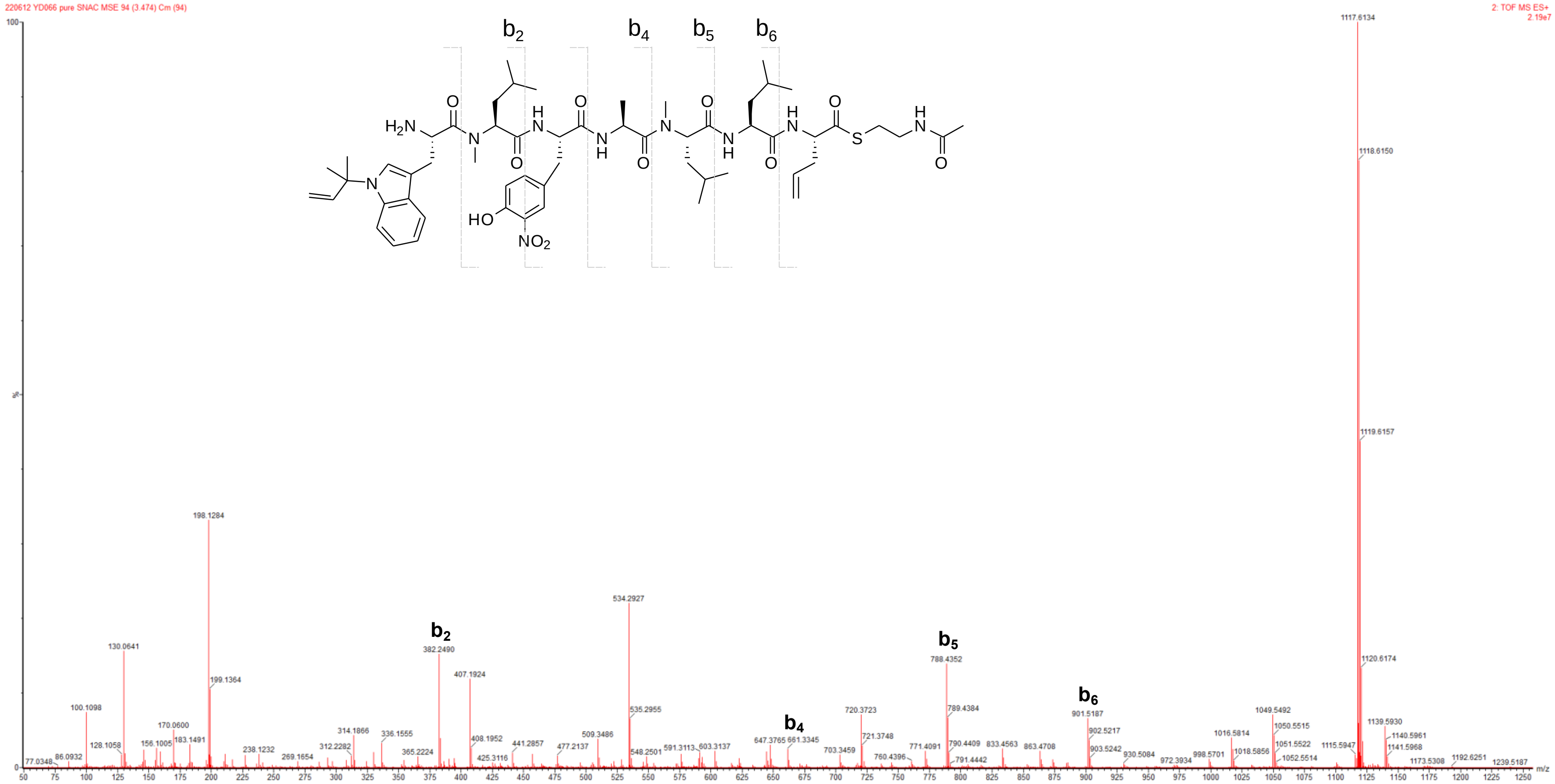

*HRMS spectra for peptide **9c** (predicted mass spectrum (top) measured (bottom))*

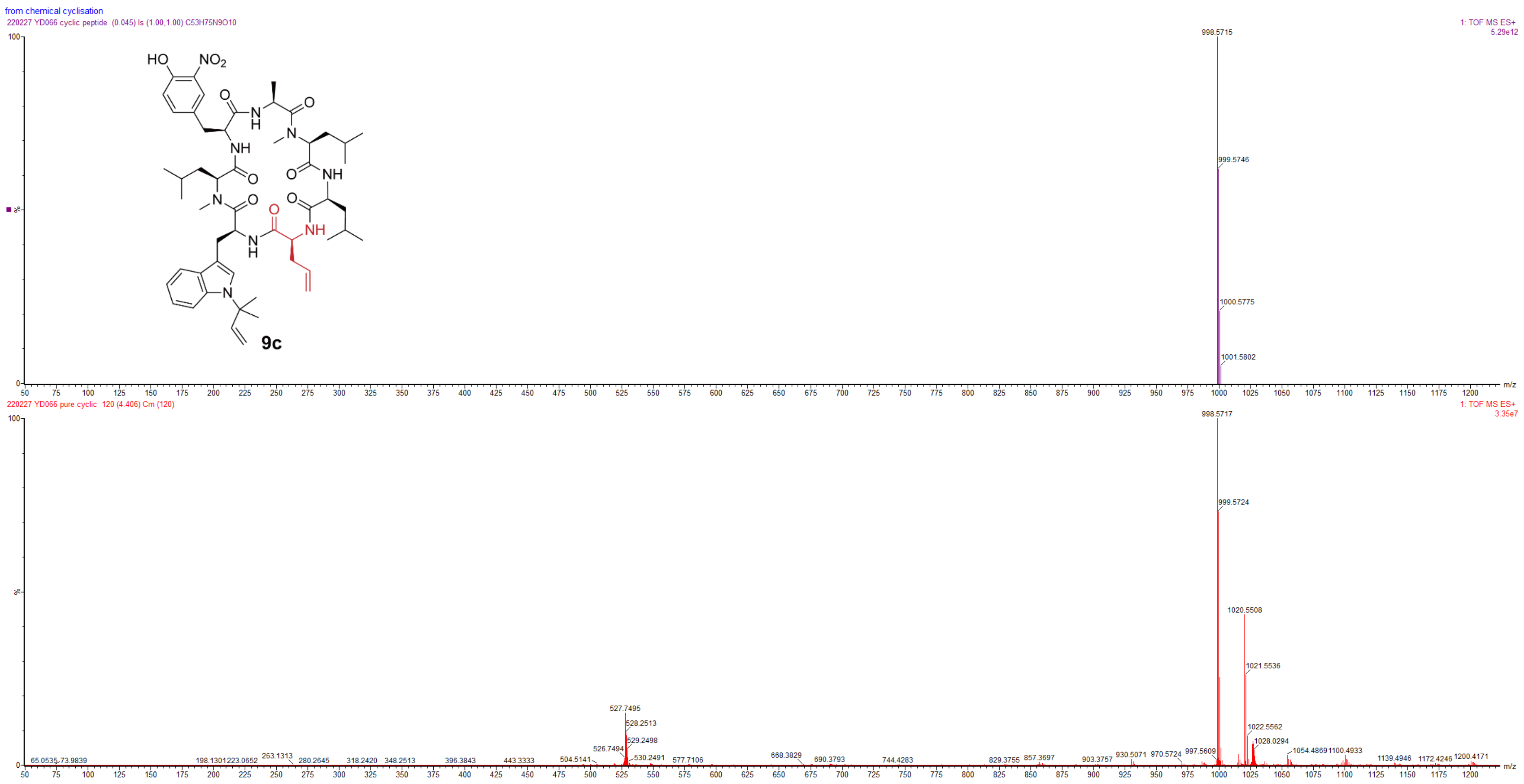

*HRMS spectra for peptide **10a**$_{L\text{-}1\text{-}Me\text{-}Trp}$ (predicted mass spectrum (top) measured (bottom))*

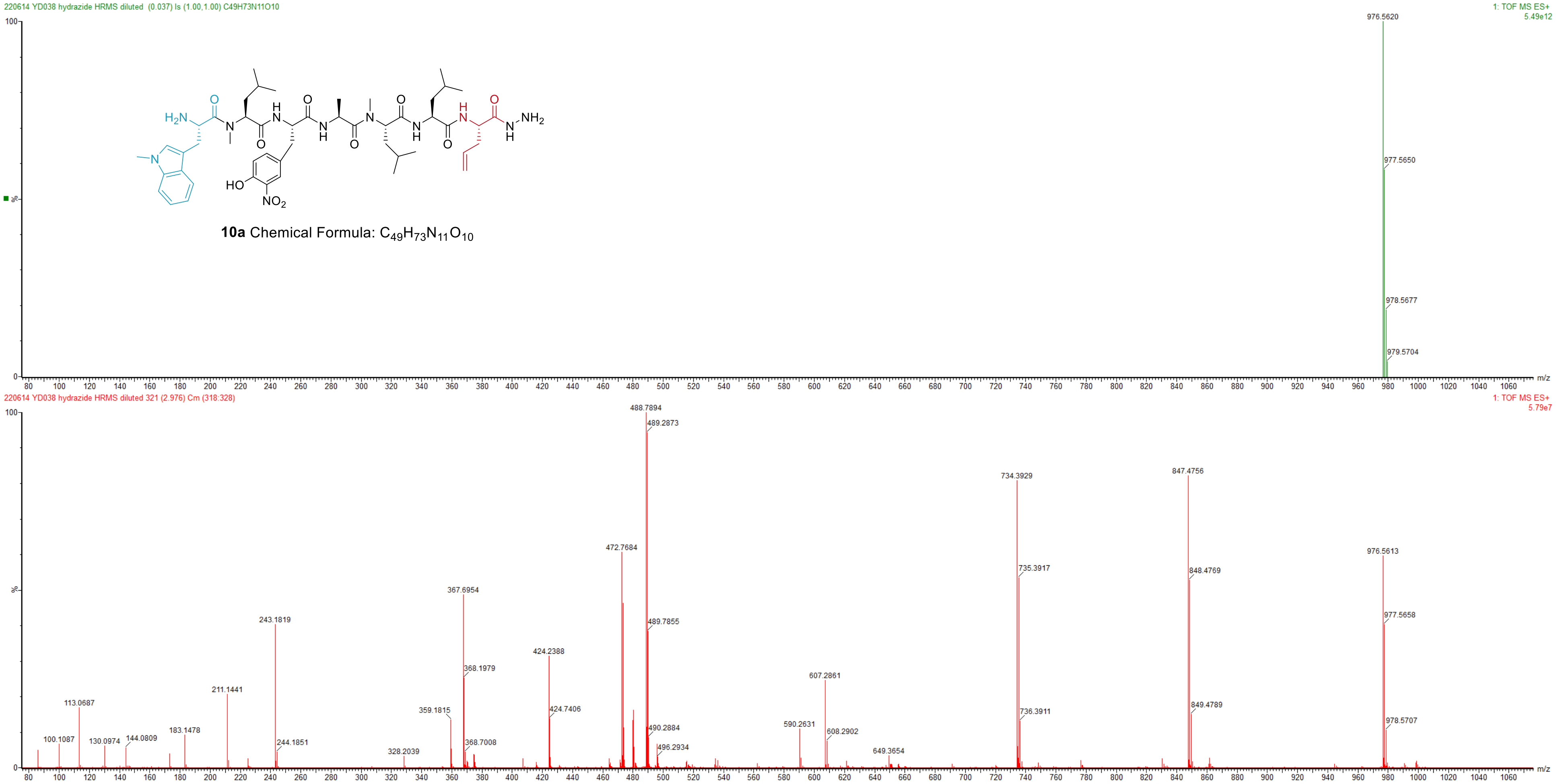

MS^E spectrum for peptide ***10a***$_{L\text{-}1\text{-}Me\text{-}Trp}$

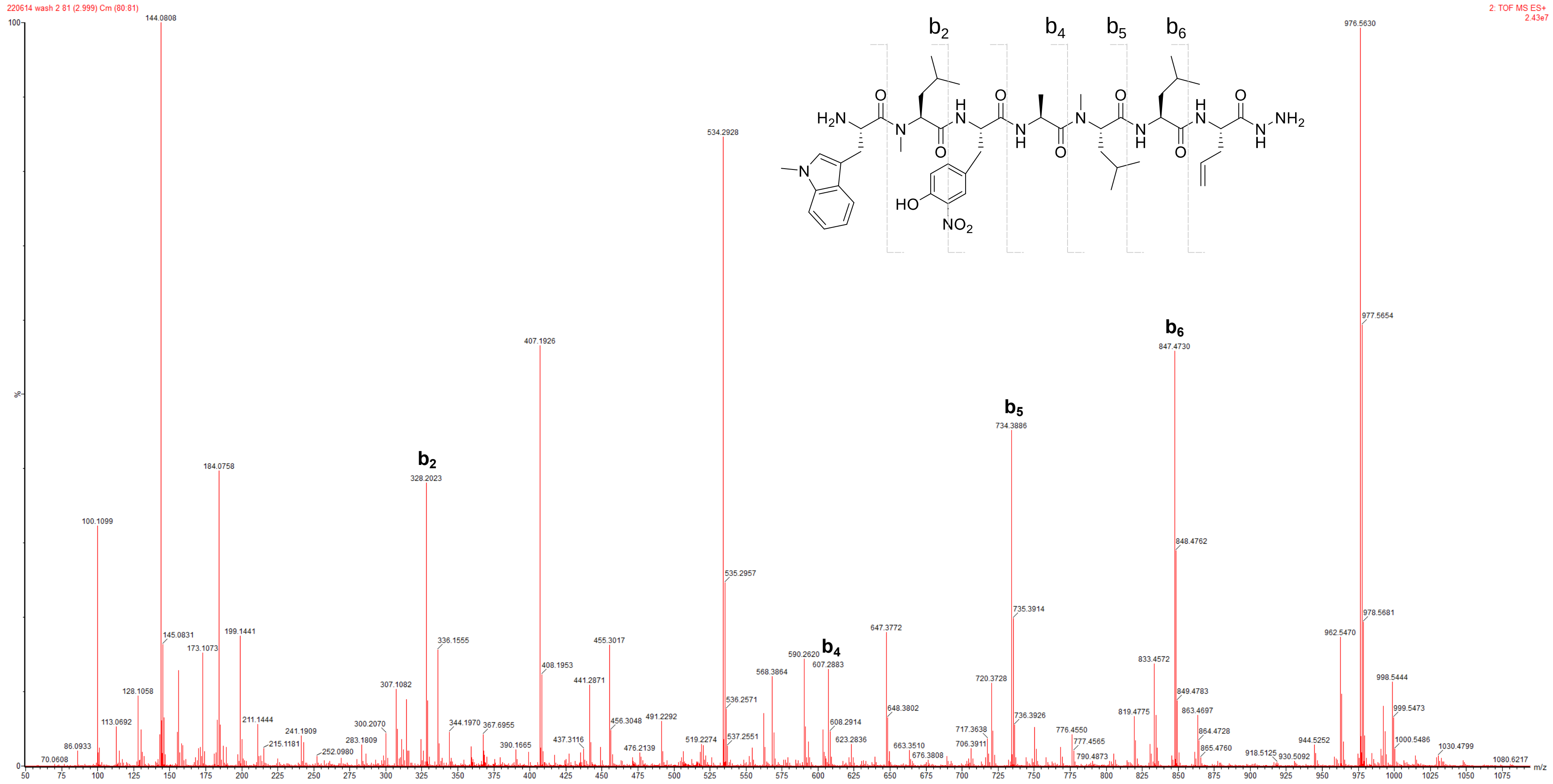

*HRMS spectra for peptide **10b**$_{D\text{-}1\text{-}Me\text{-}Trp}$ (predicted mass spectrum (top) measured (bottom))*

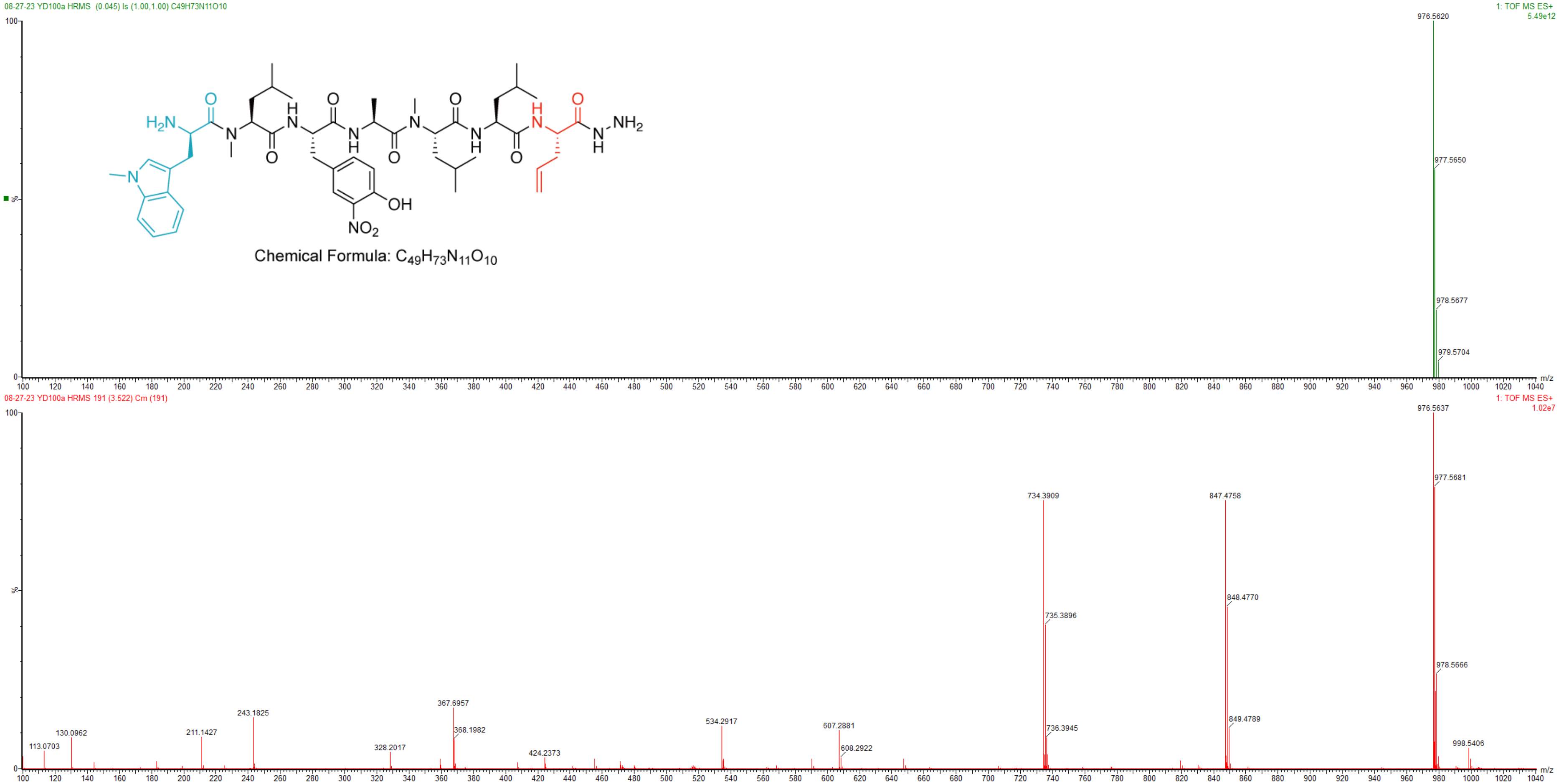

*HRMS spectra for peptide **11a** (predicted mass spectrum (top) measured (bottom))*

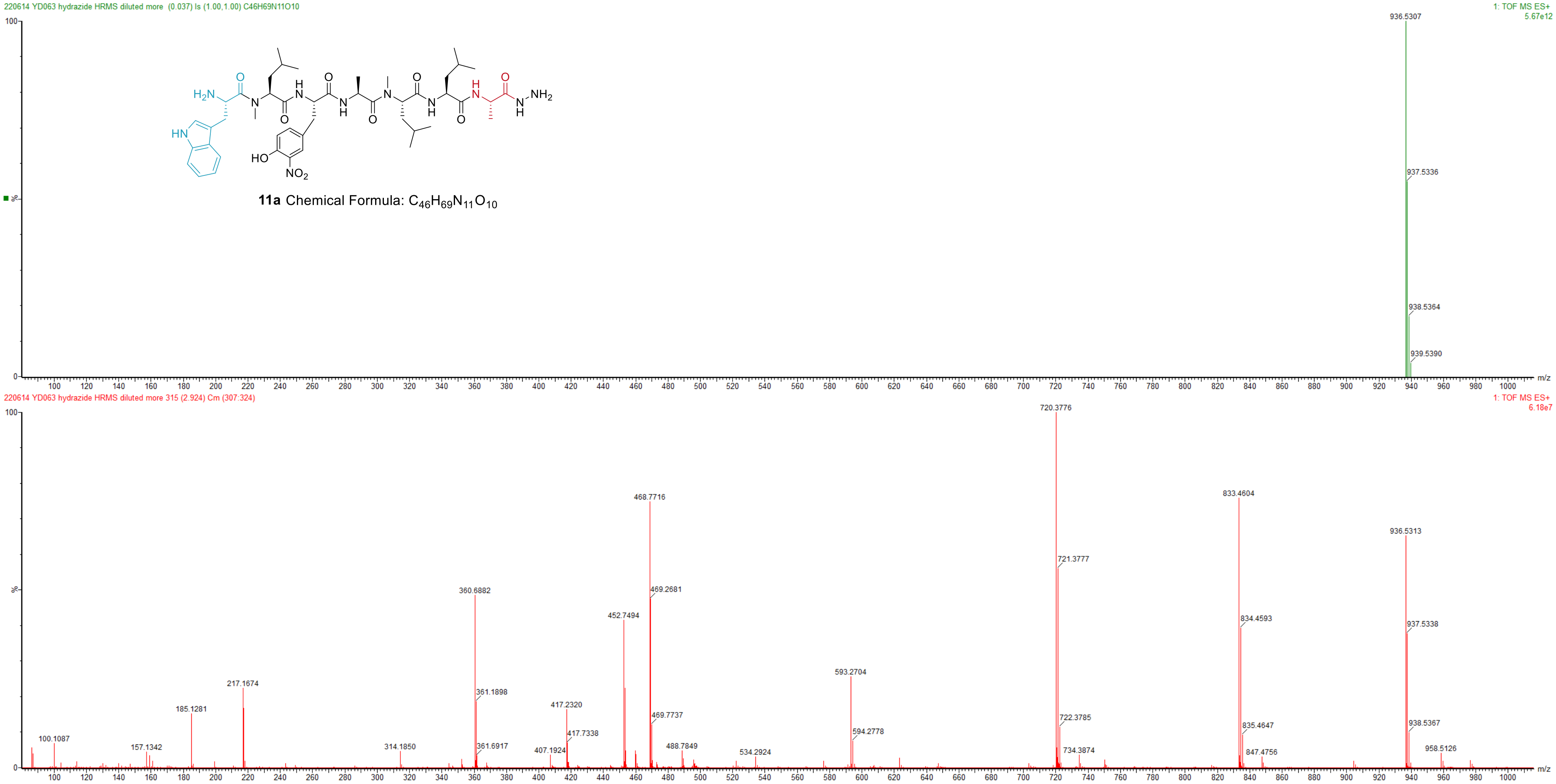

*MS^E spectrum for peptide **11a***

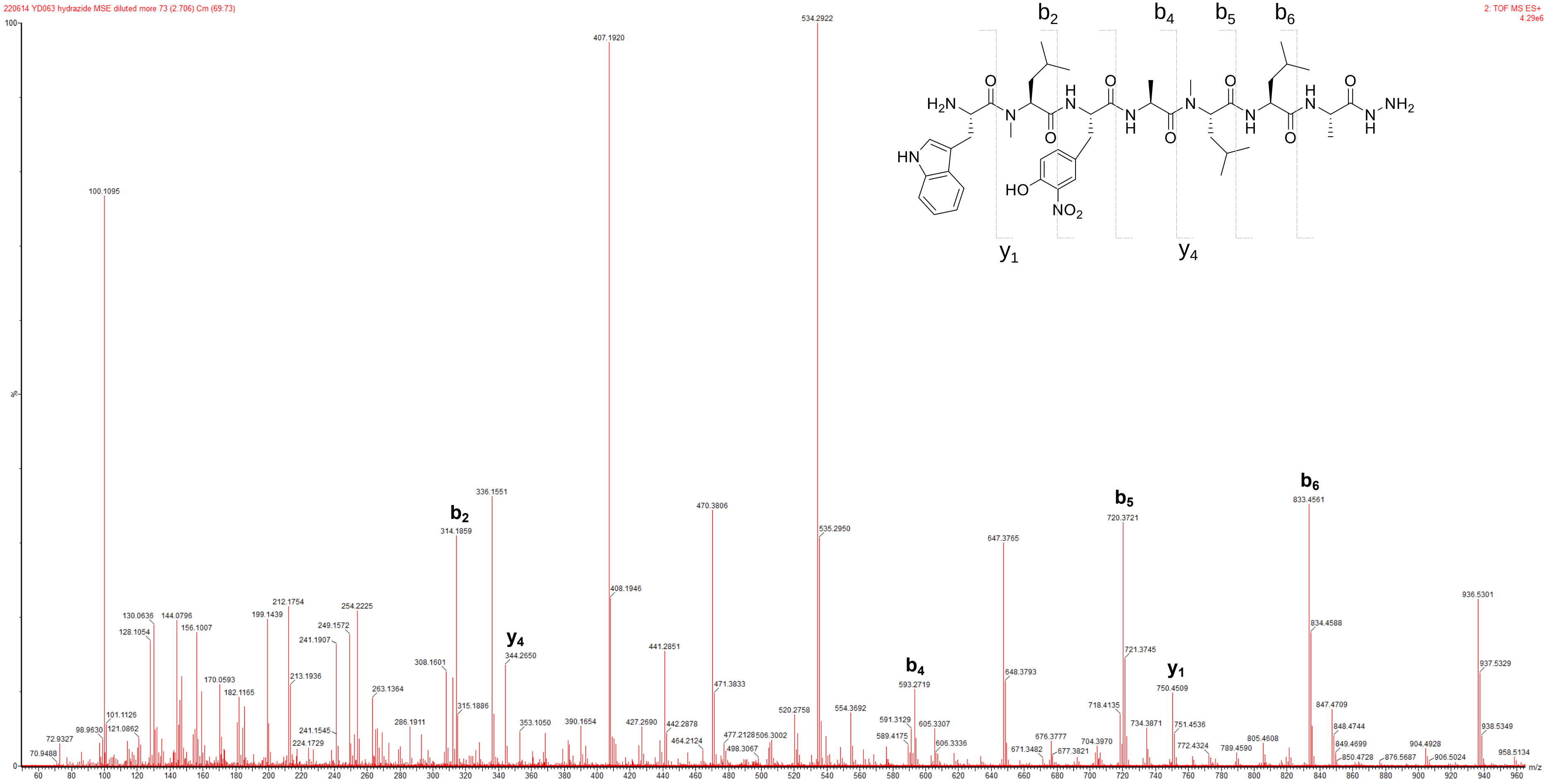

*HRMS spectra for peptide **12a** (predicted mass spectrum (top) measured (bottom))*

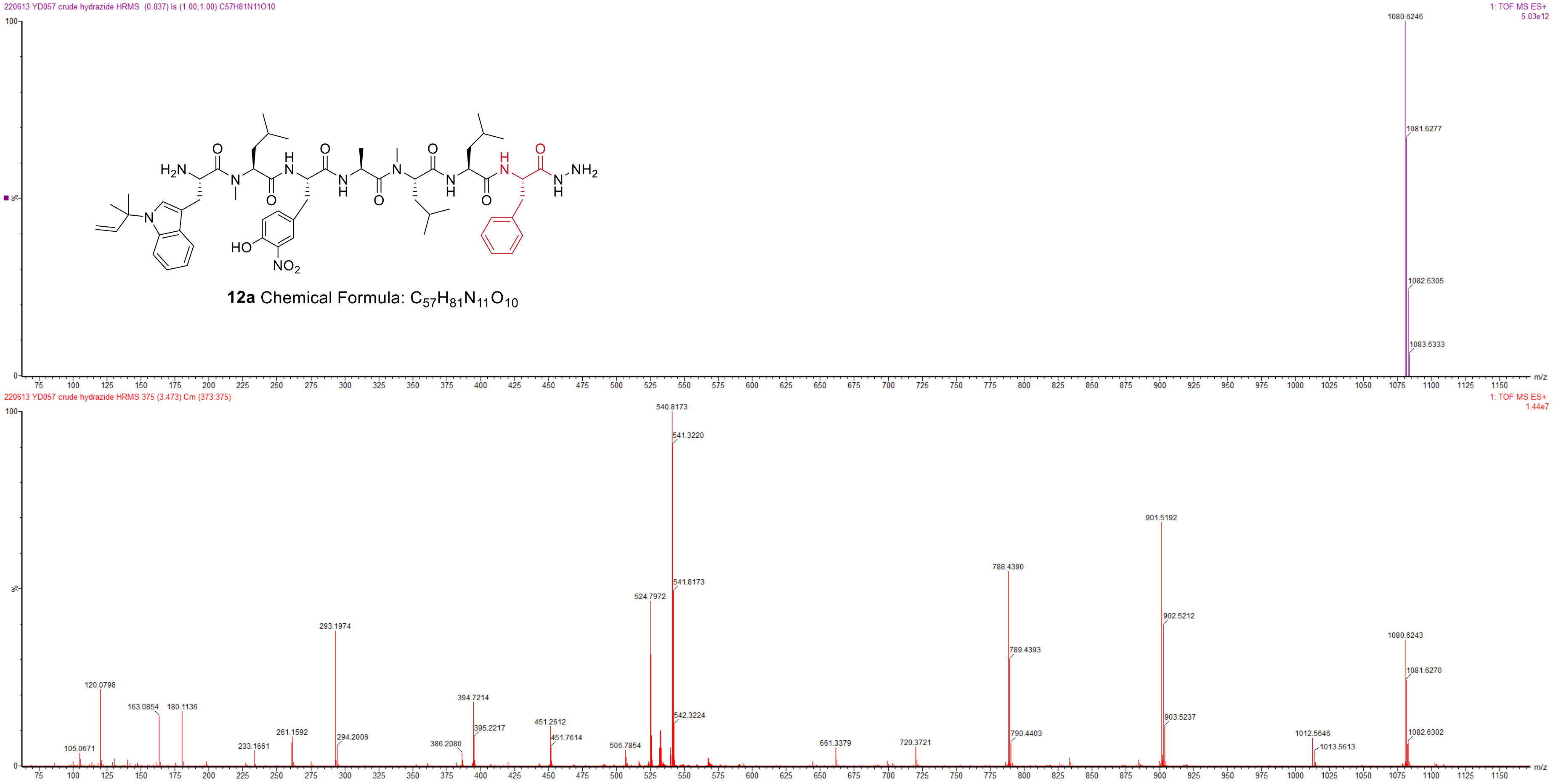

*MS^E spectrum for peptide **12a***

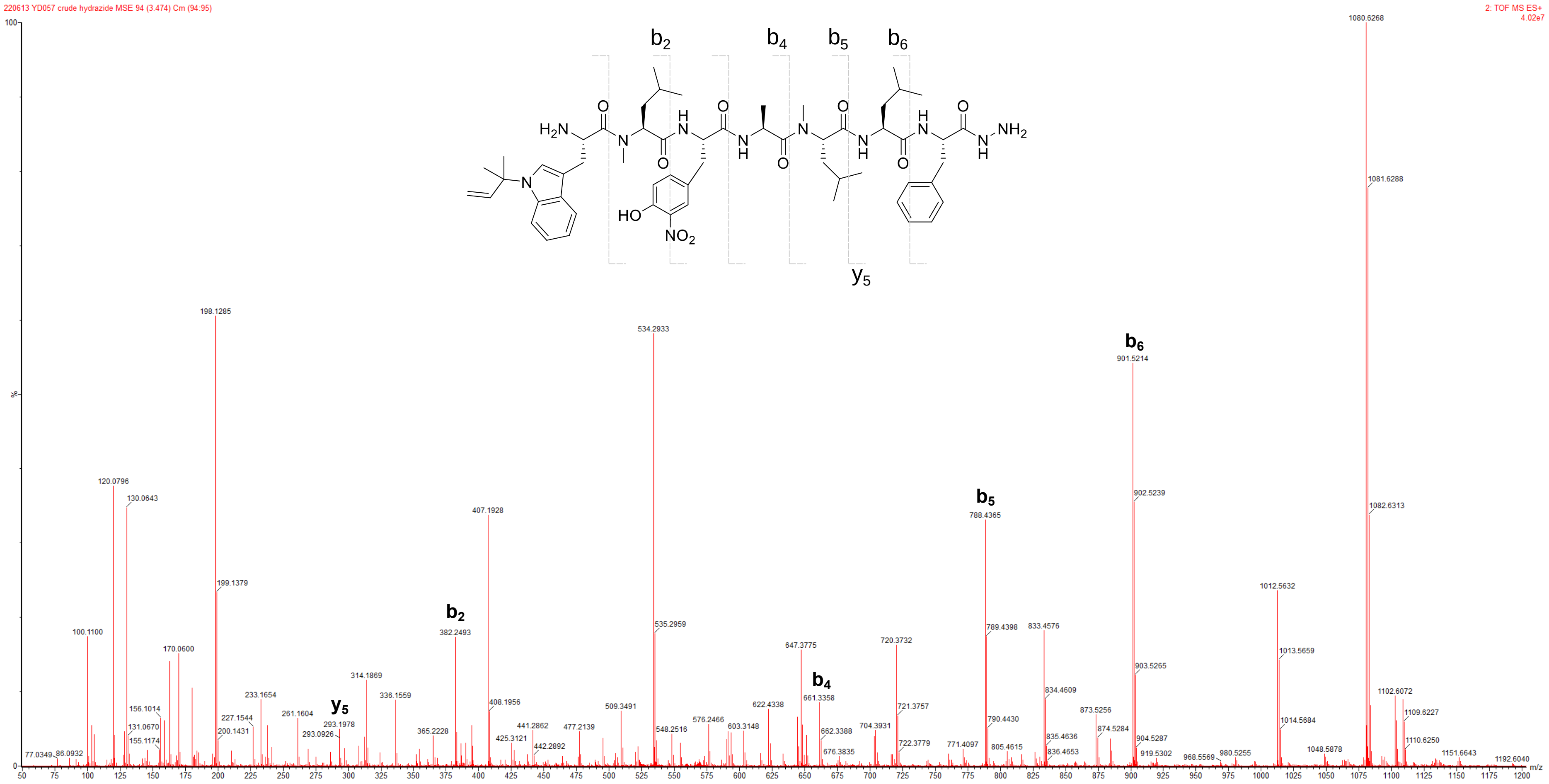

*HRMS spectra for peptide **13a** (predicted mass spectrum (top) measured (bottom))*

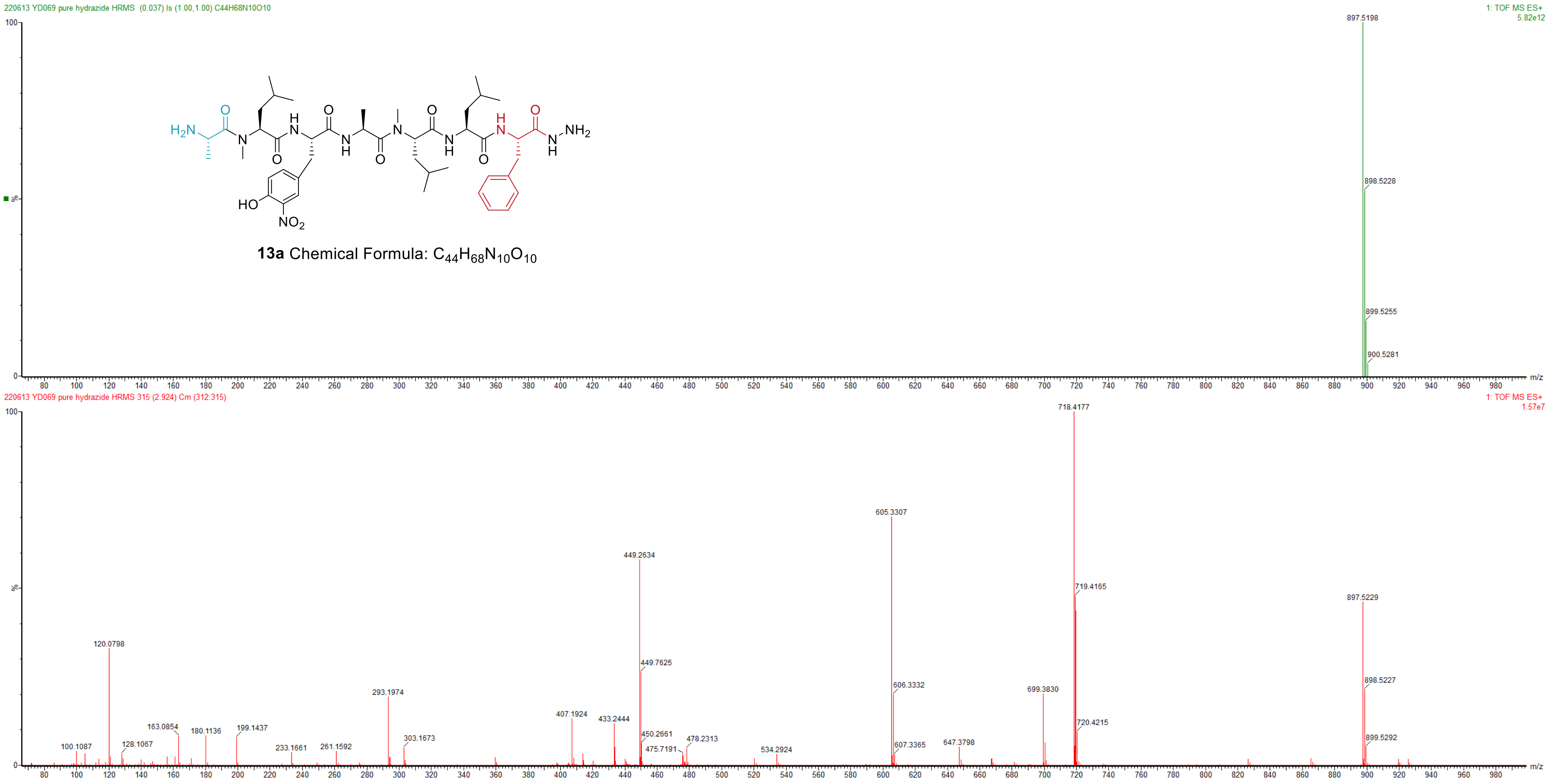

*MS^E spectrum for peptide **13a***

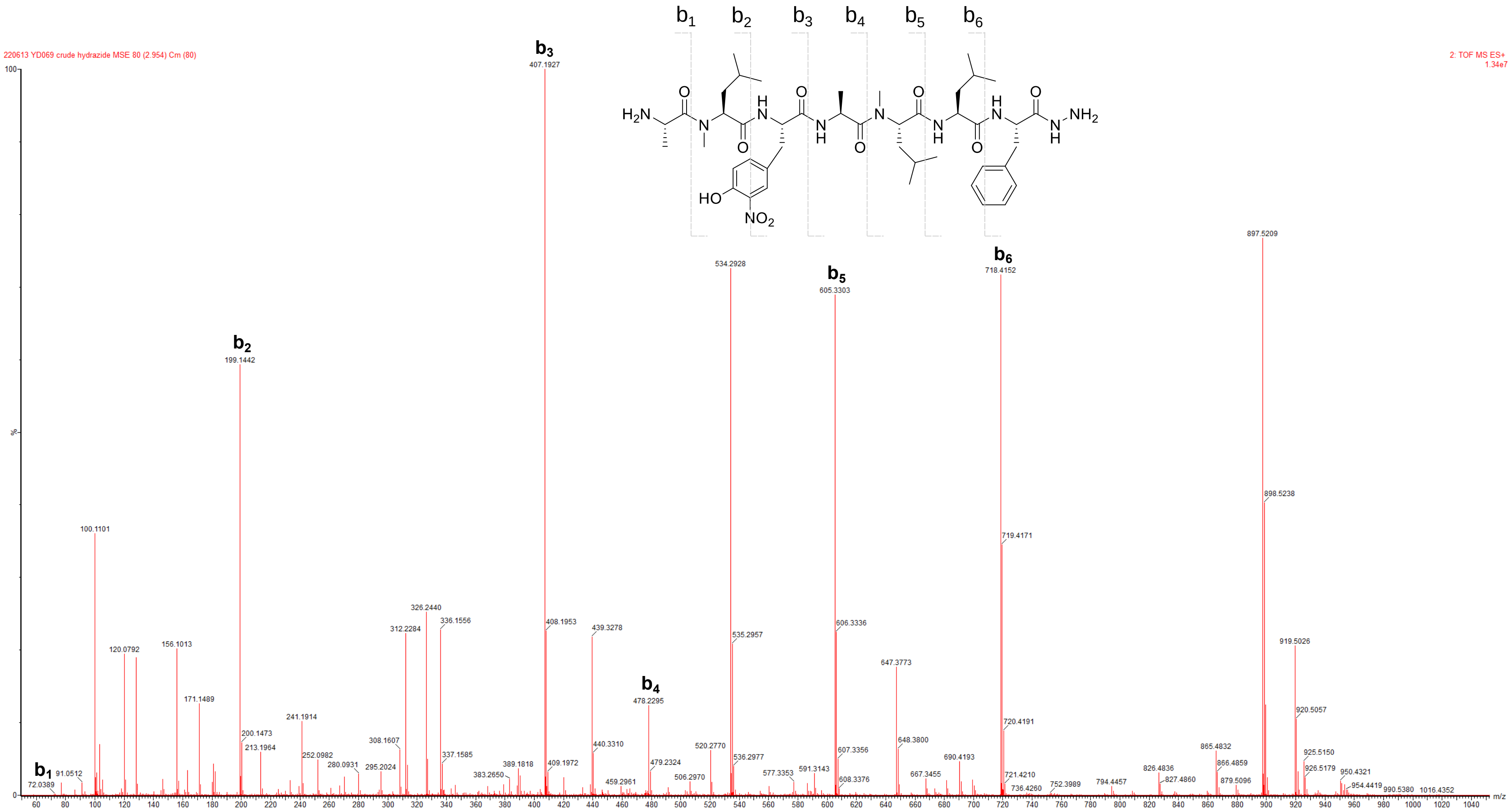

*HRMS spectra for peptide **14a** (predicted mass spectrum (bottom) measured (top))*

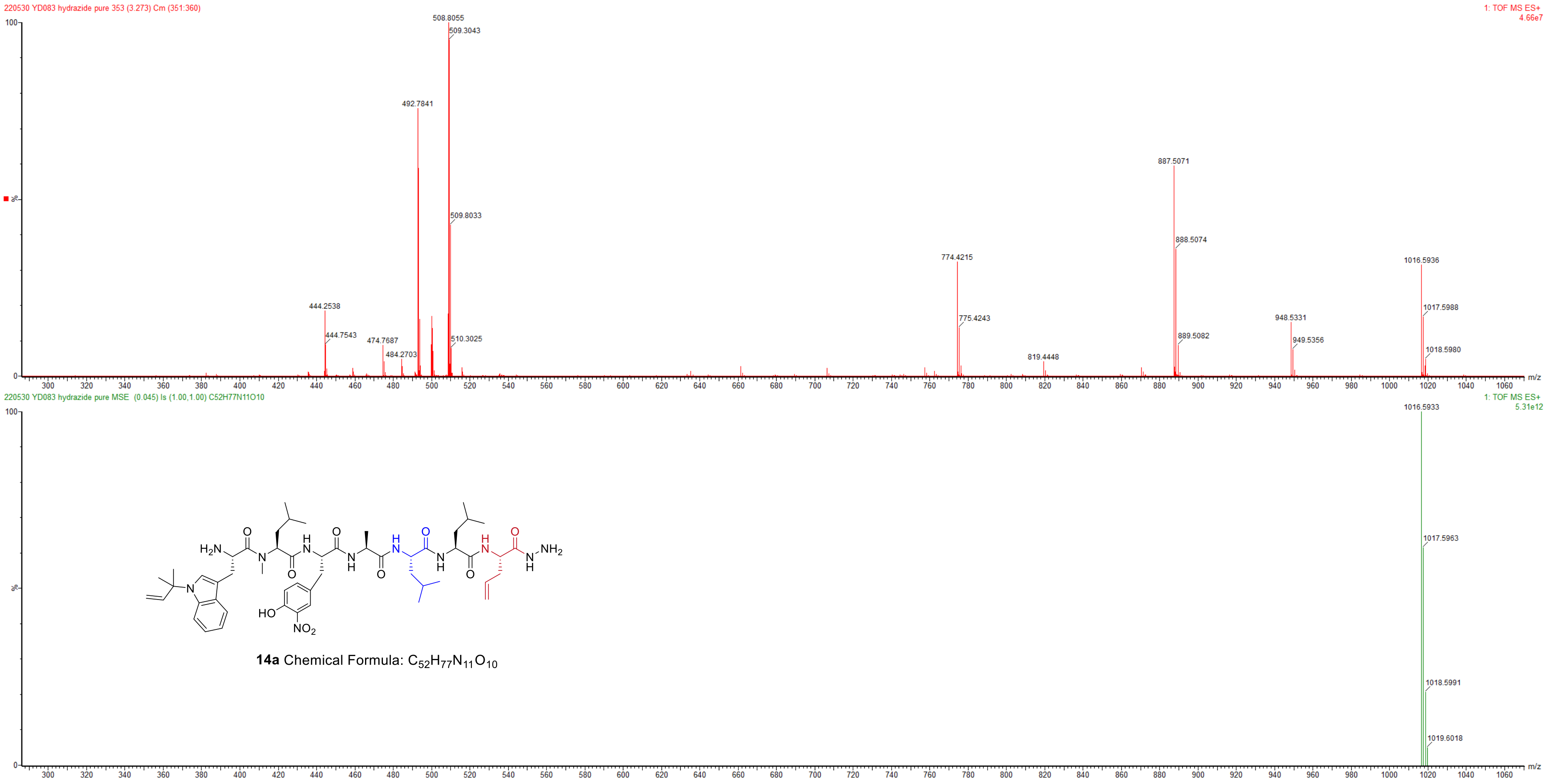

14a Chemical Formula: $C_{52}H_{77}N_{11}O_{10}$

*MS^E spectrum for peptide **14a***

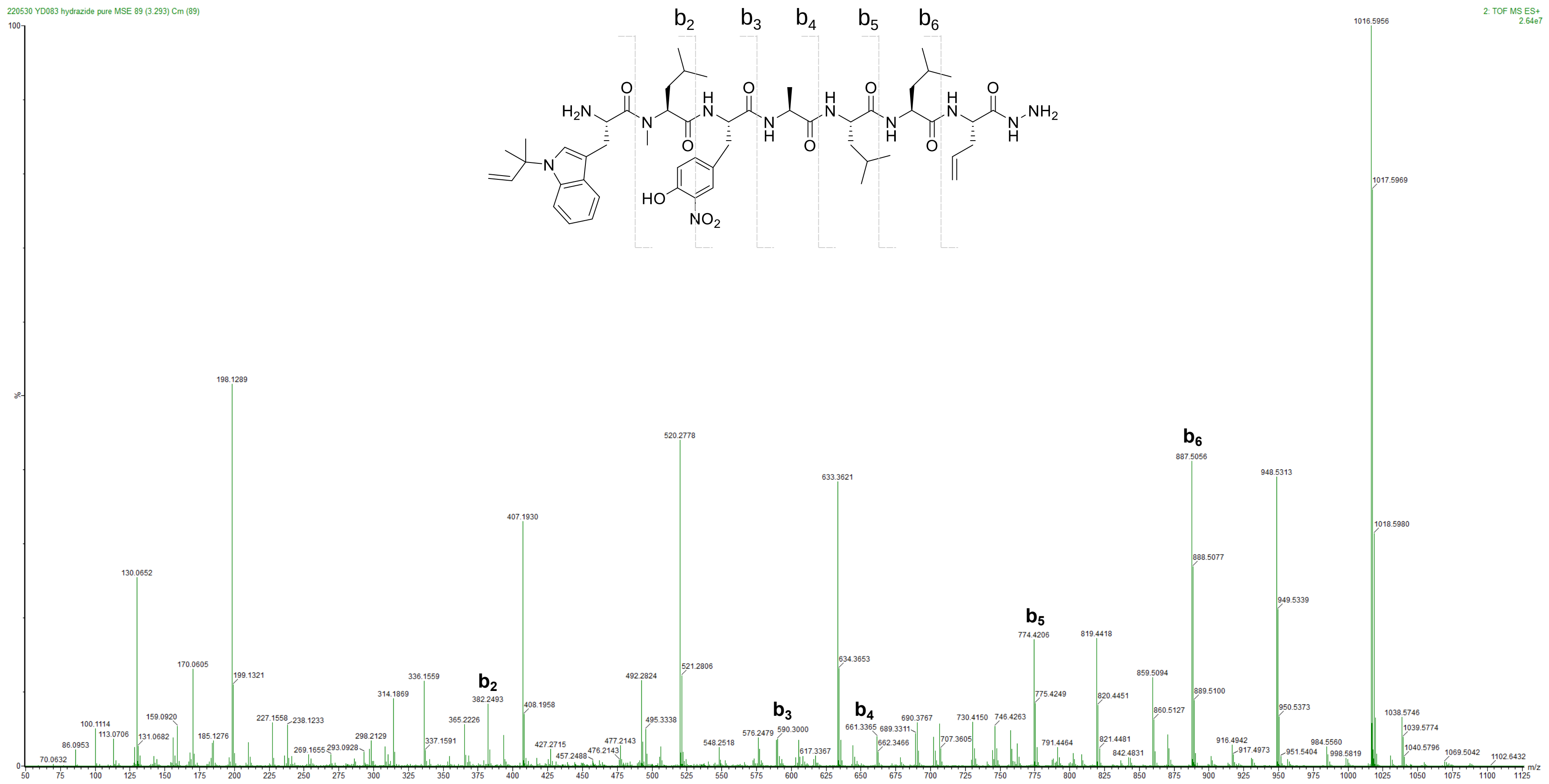

*HRMS spectrum for peptide **15a** (predicted mass spectrum (top) measured (bottom))*

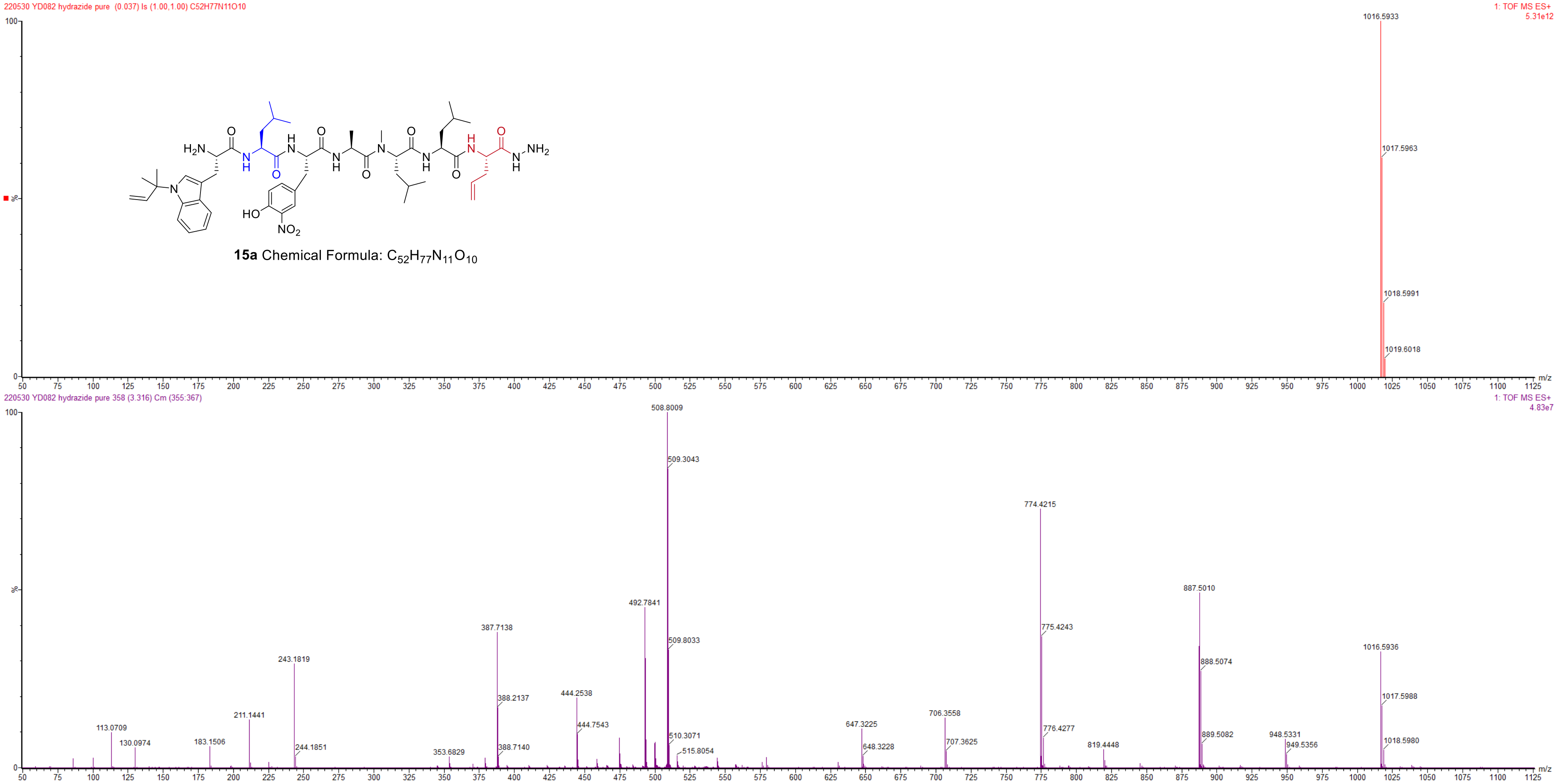

MS^E spectrum for peptide ***15a***

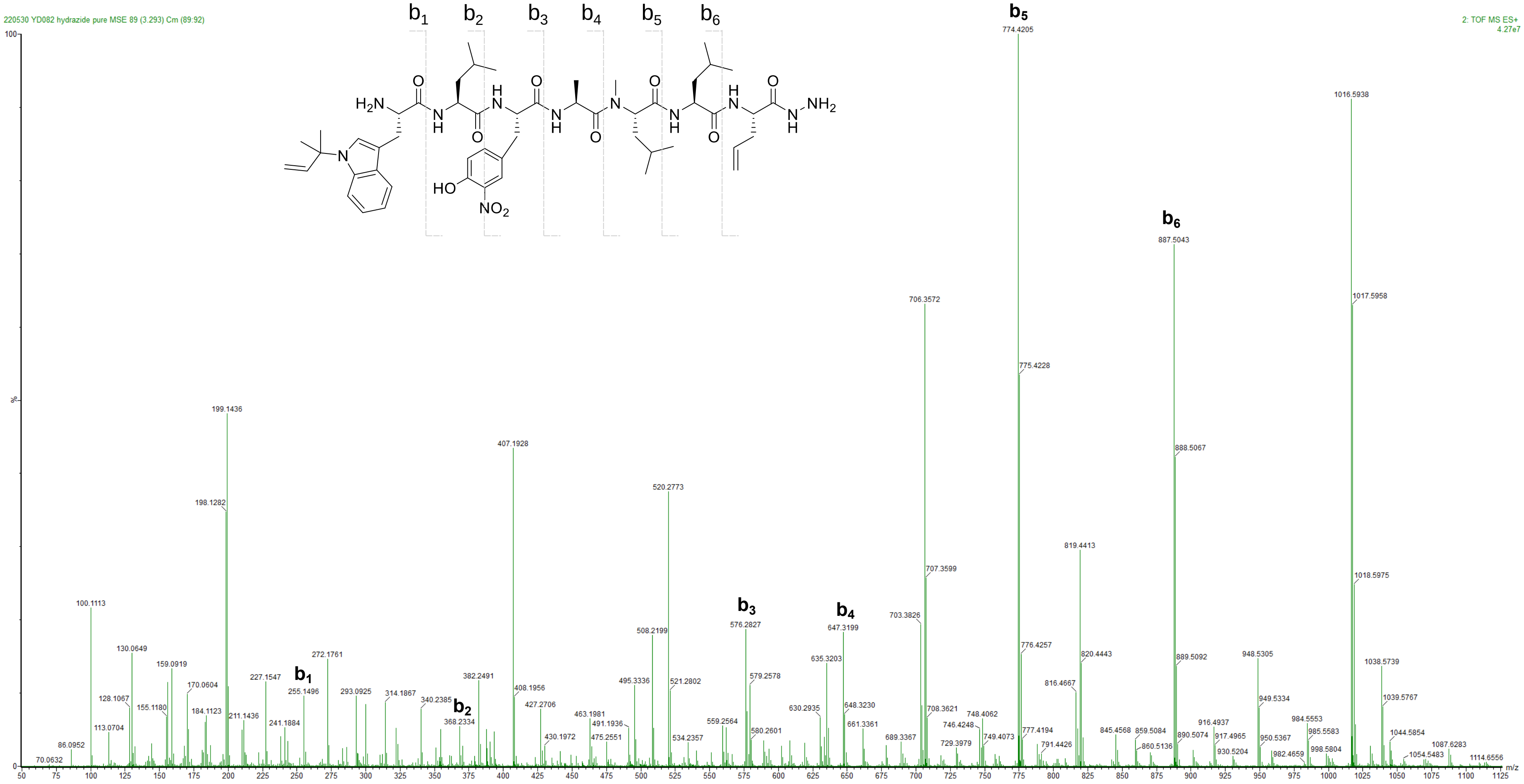

*HRMS spectrum for peptide **16a** (predicted mass spectrum (top) measured (bottom))*

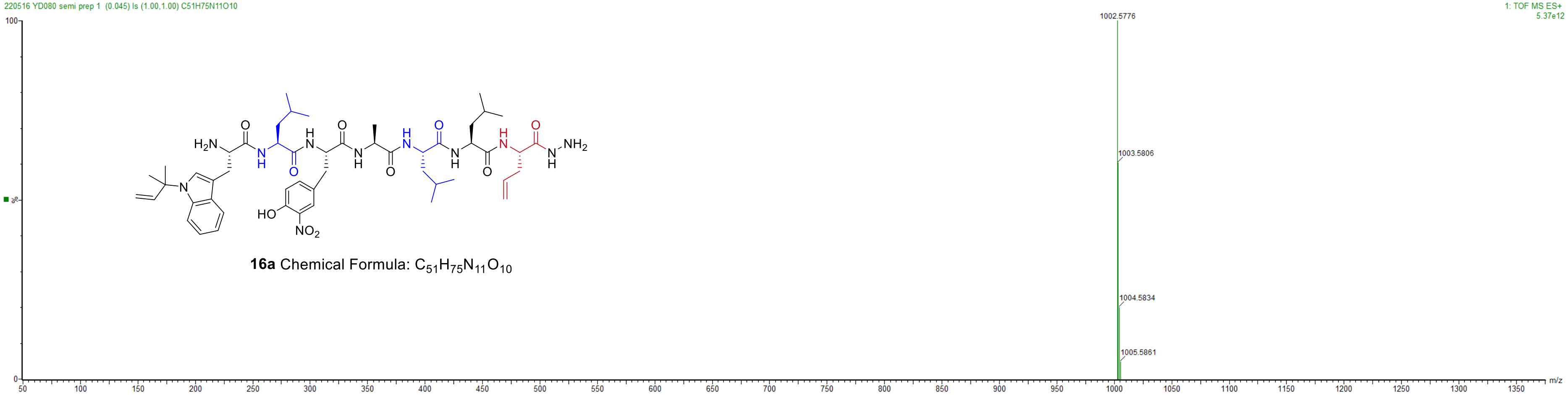

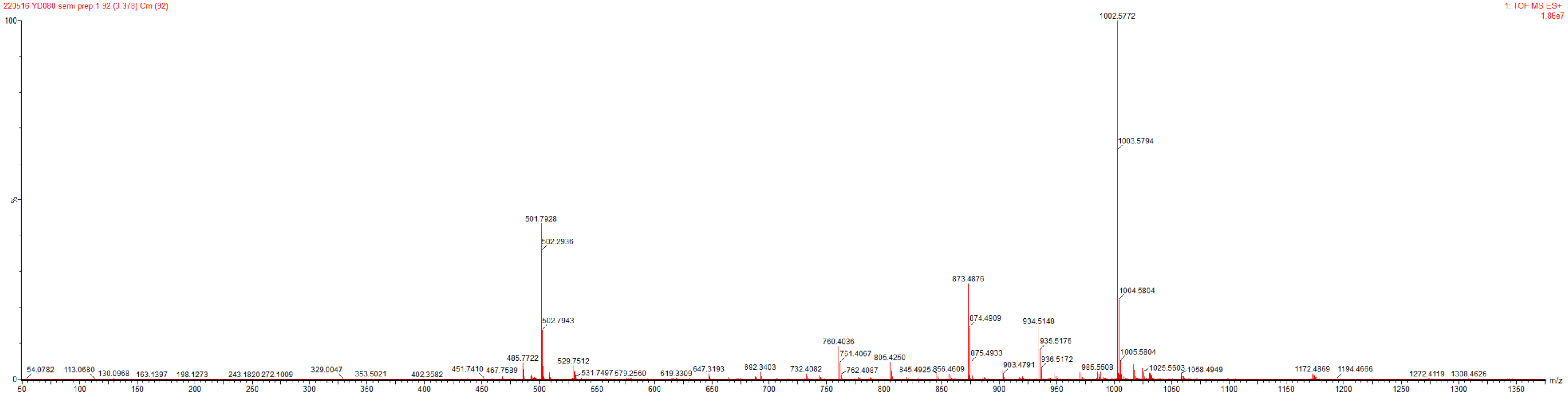

*MS^E spectrum for peptide **16a***

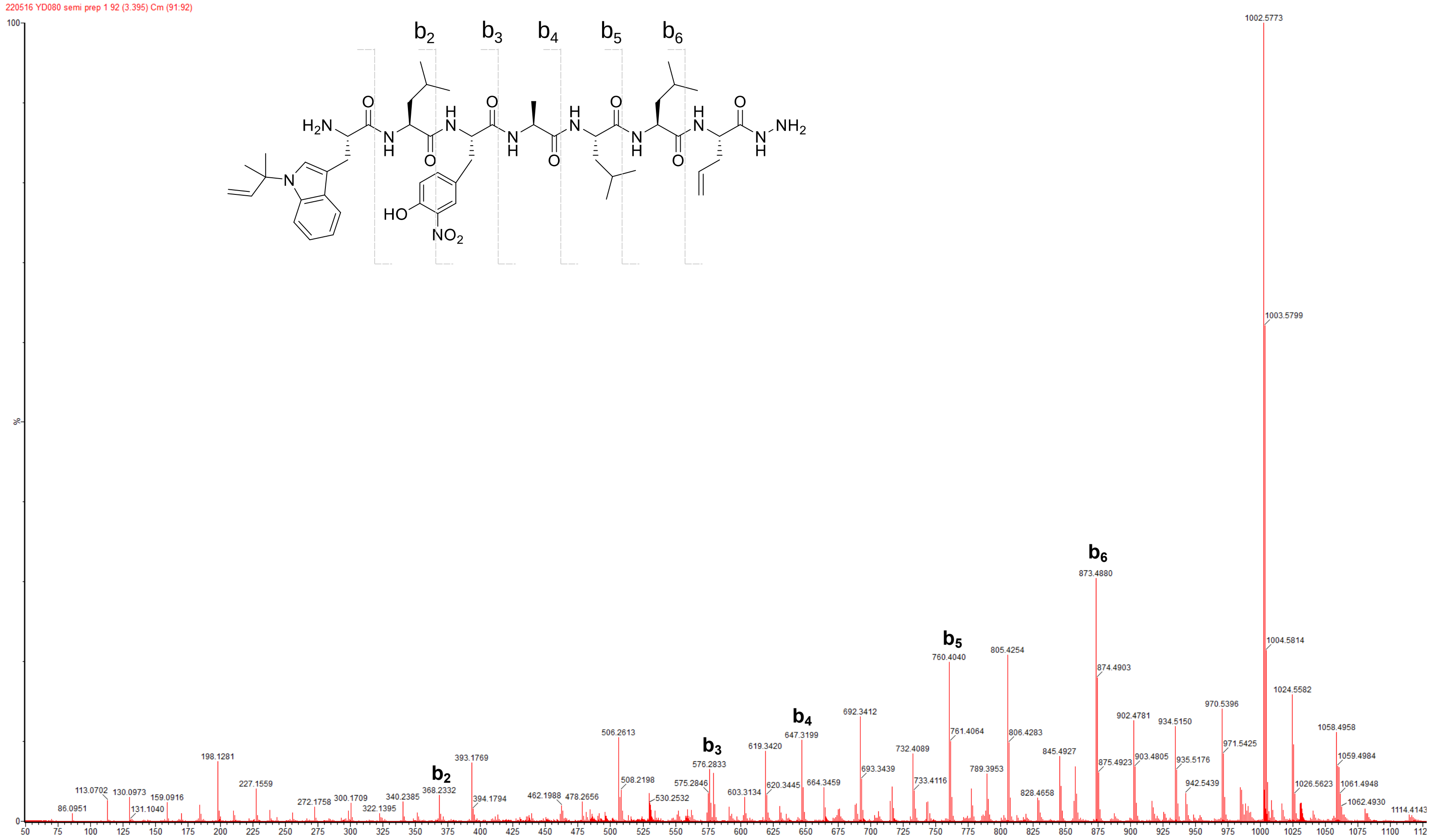

*HRMS spectrum for peptide **17a** (predicted mass spectrum (top) measured (bottom))*

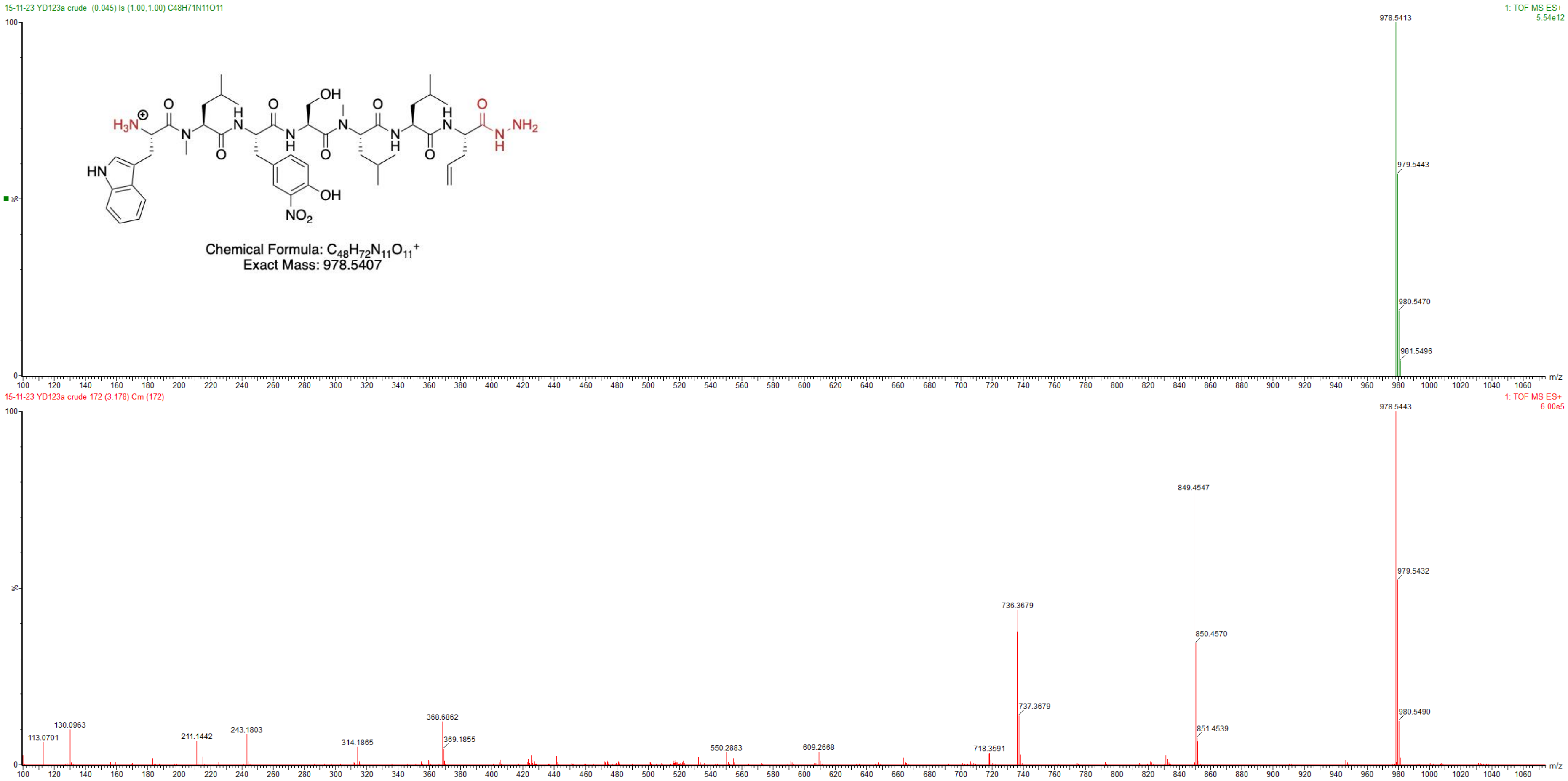

MS^E spectrum for peptide ***17a***

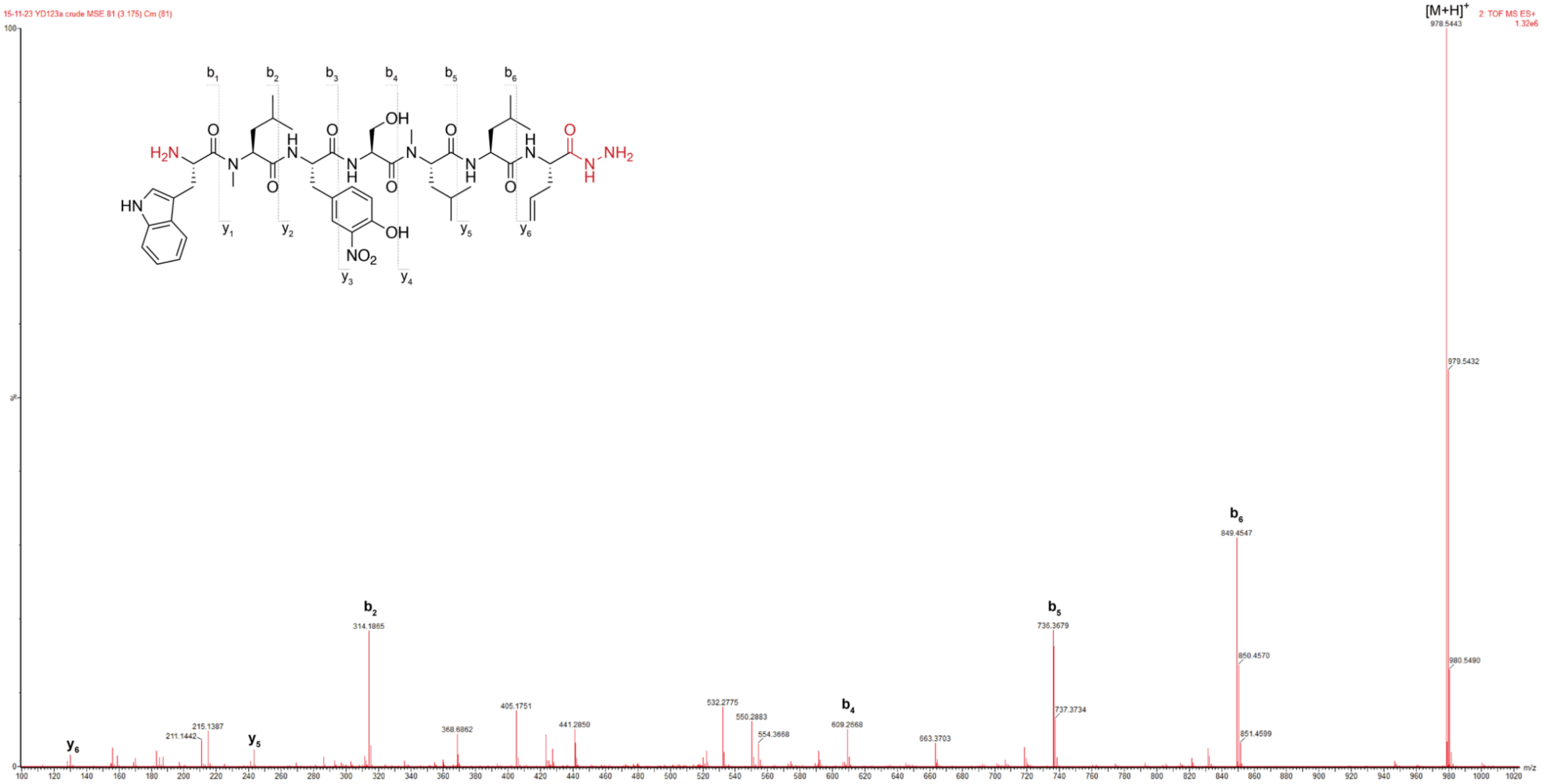

*HRMS spectrum for peptide **17c** (predicted mass spectrum (top) measured (bottom))*

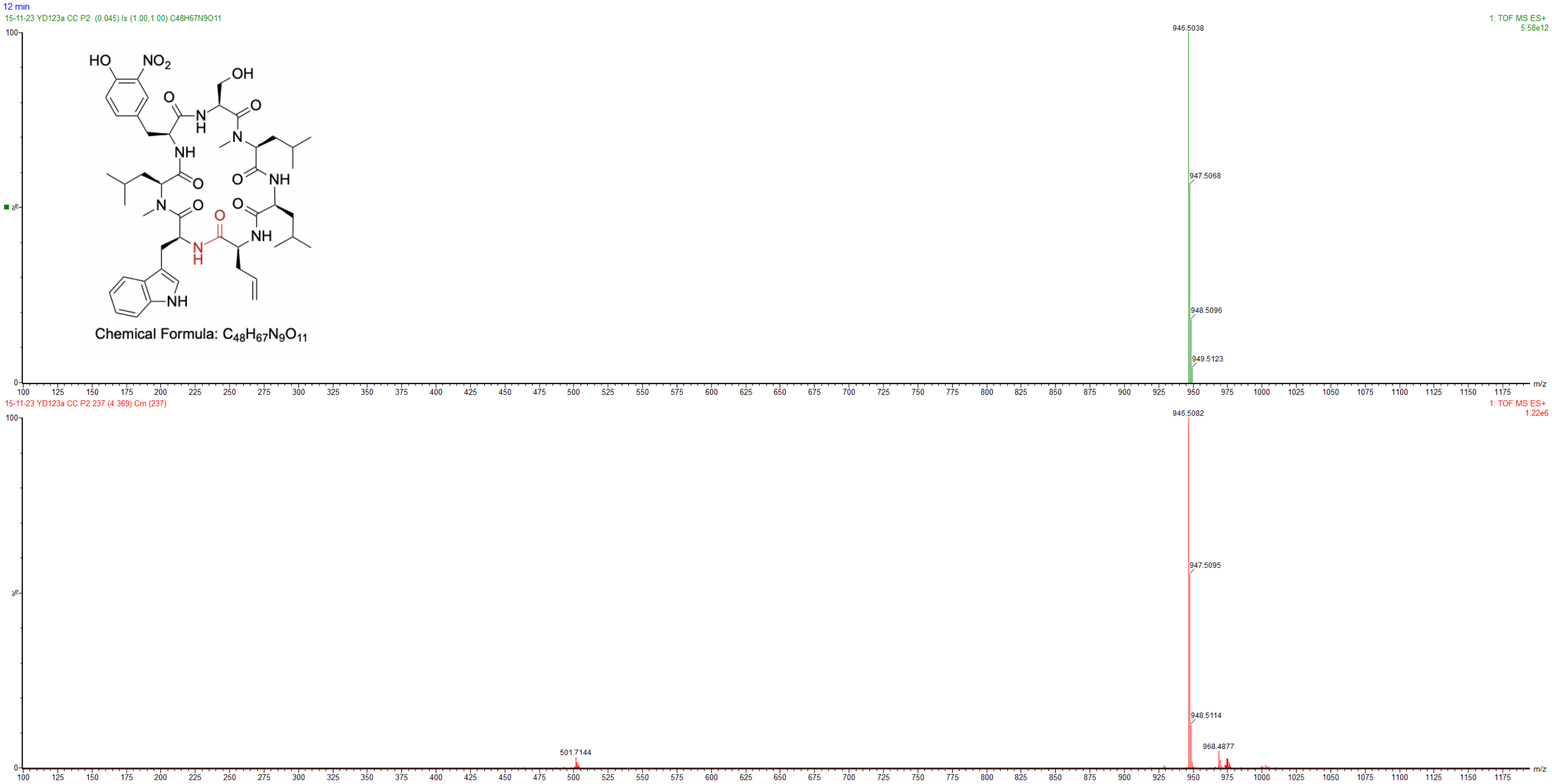

*HRMS spectrum for peptide **18a** (predicted mass spectrum (top) measured (bottom)*

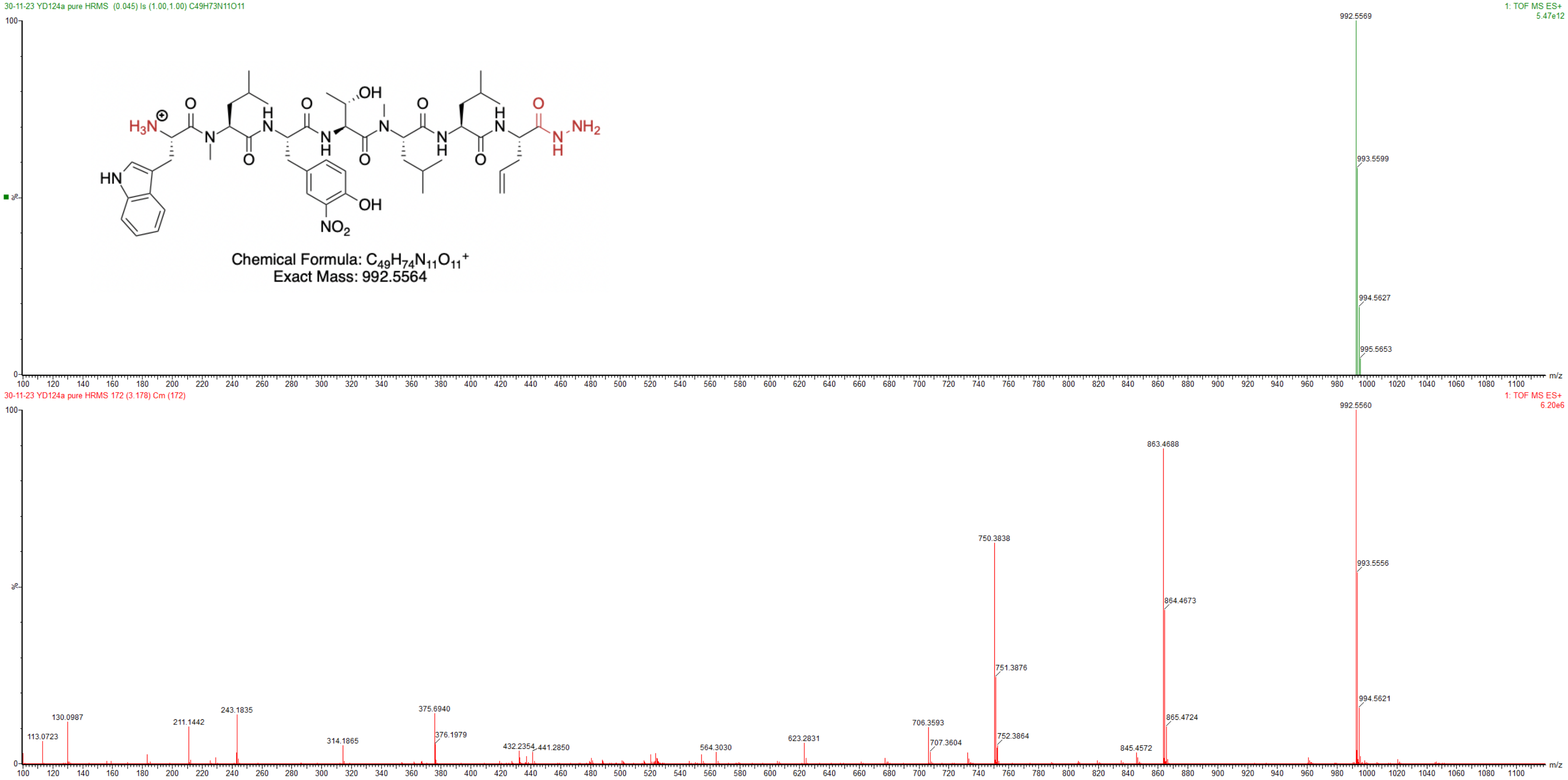

*MS^E spectrum for peptide **18a***

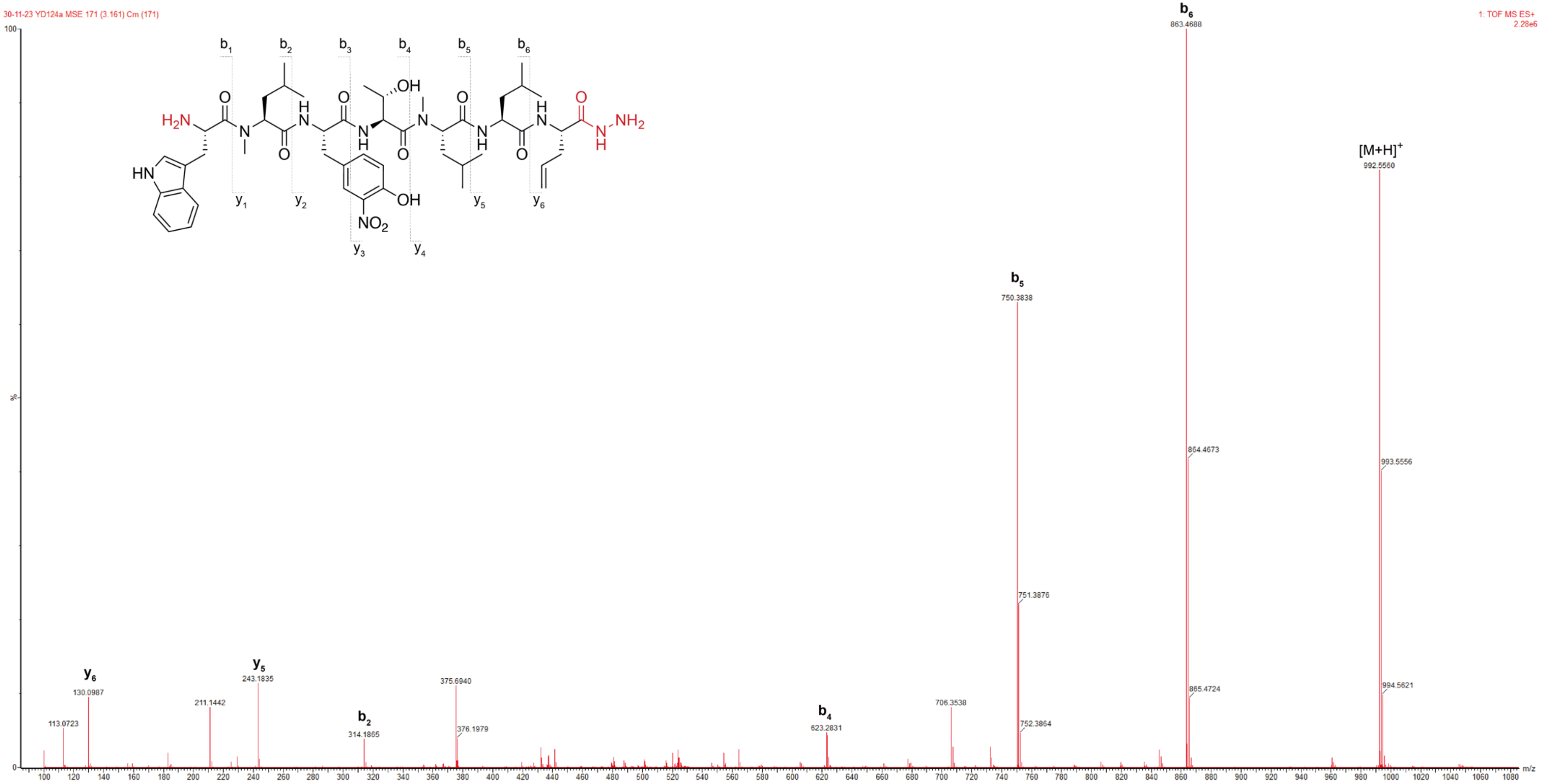

*HRMS spectrum for peptide **18c** (predicted mass spectrum (top) measured (bottom))*

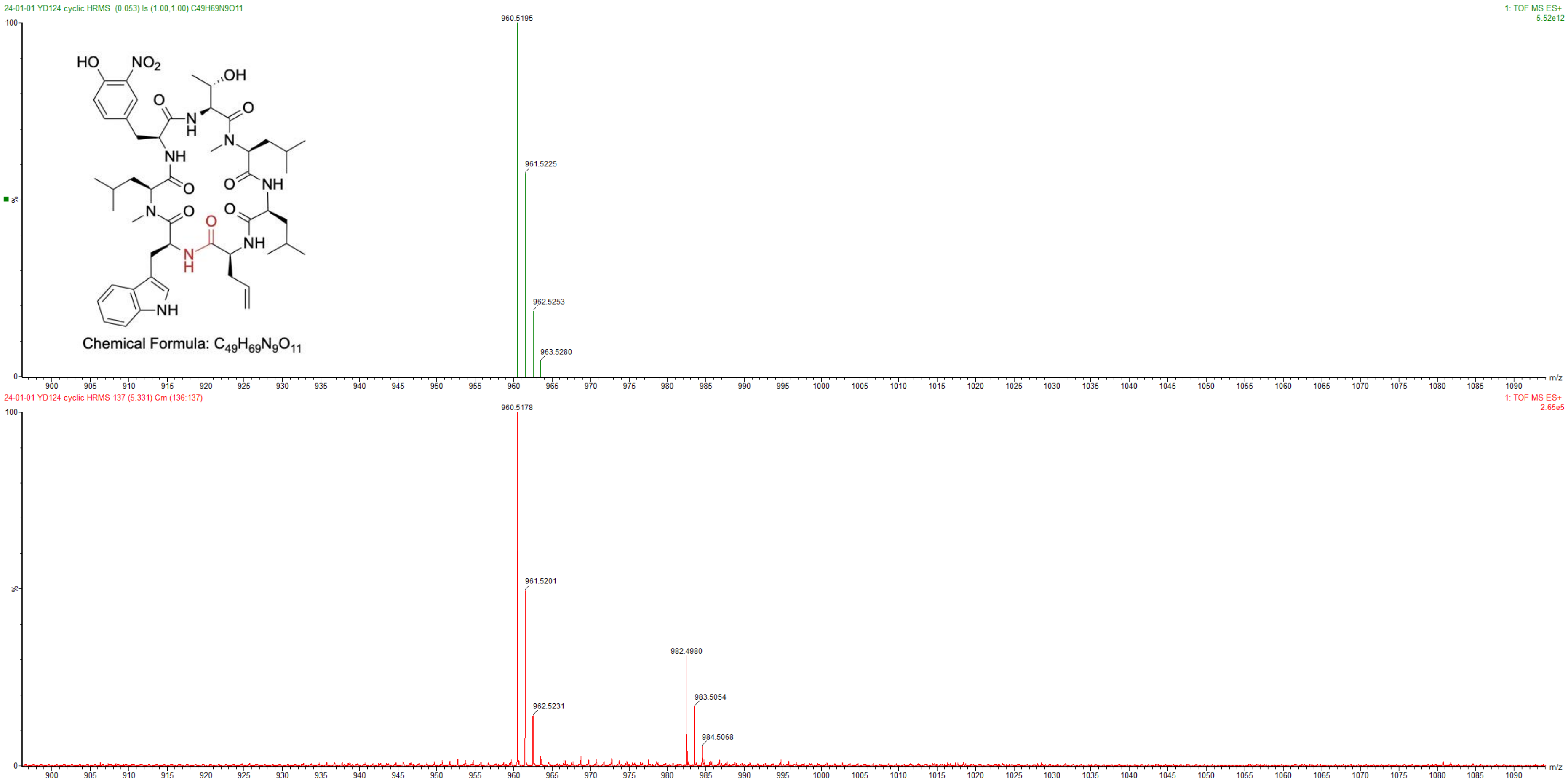

*HRMS spectrum for peptide **19a** (predicted mass spectrum (top) measured (bottom))*

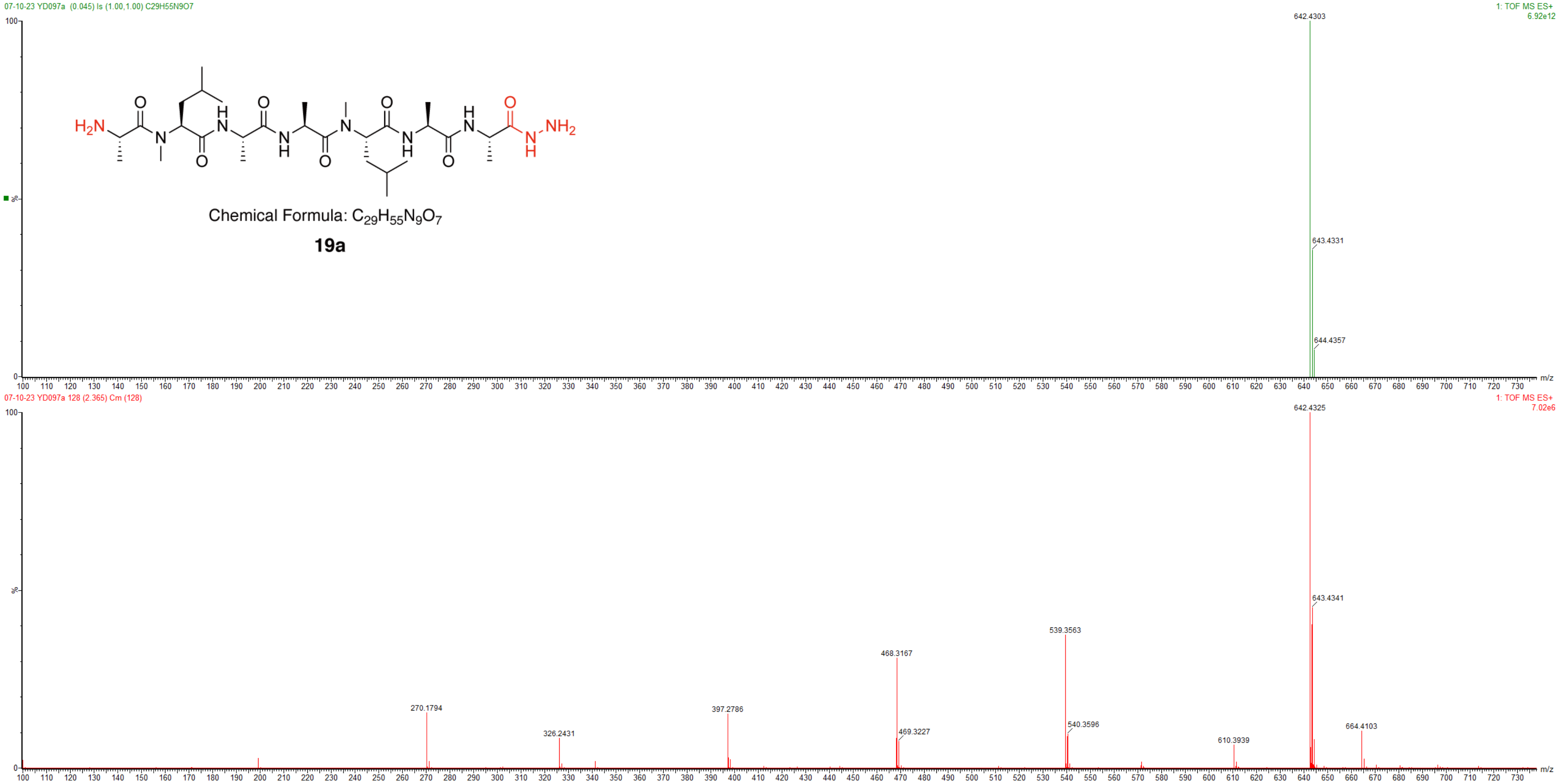

*MS^E spectrum for peptide **19a***

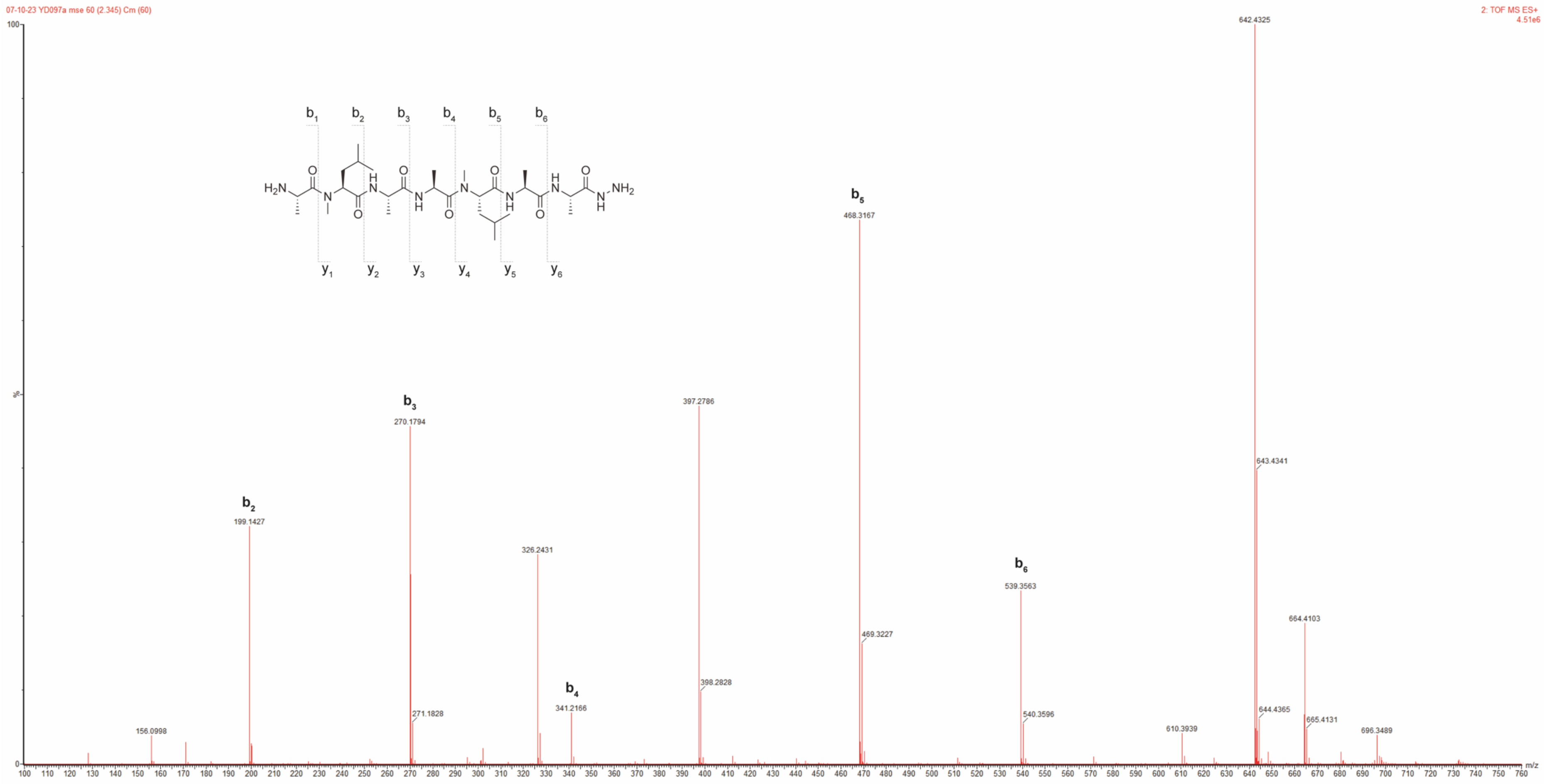

*HRMS spectrum for peptide **19c** (predicted mass spectrum (top) measured (bottom))*

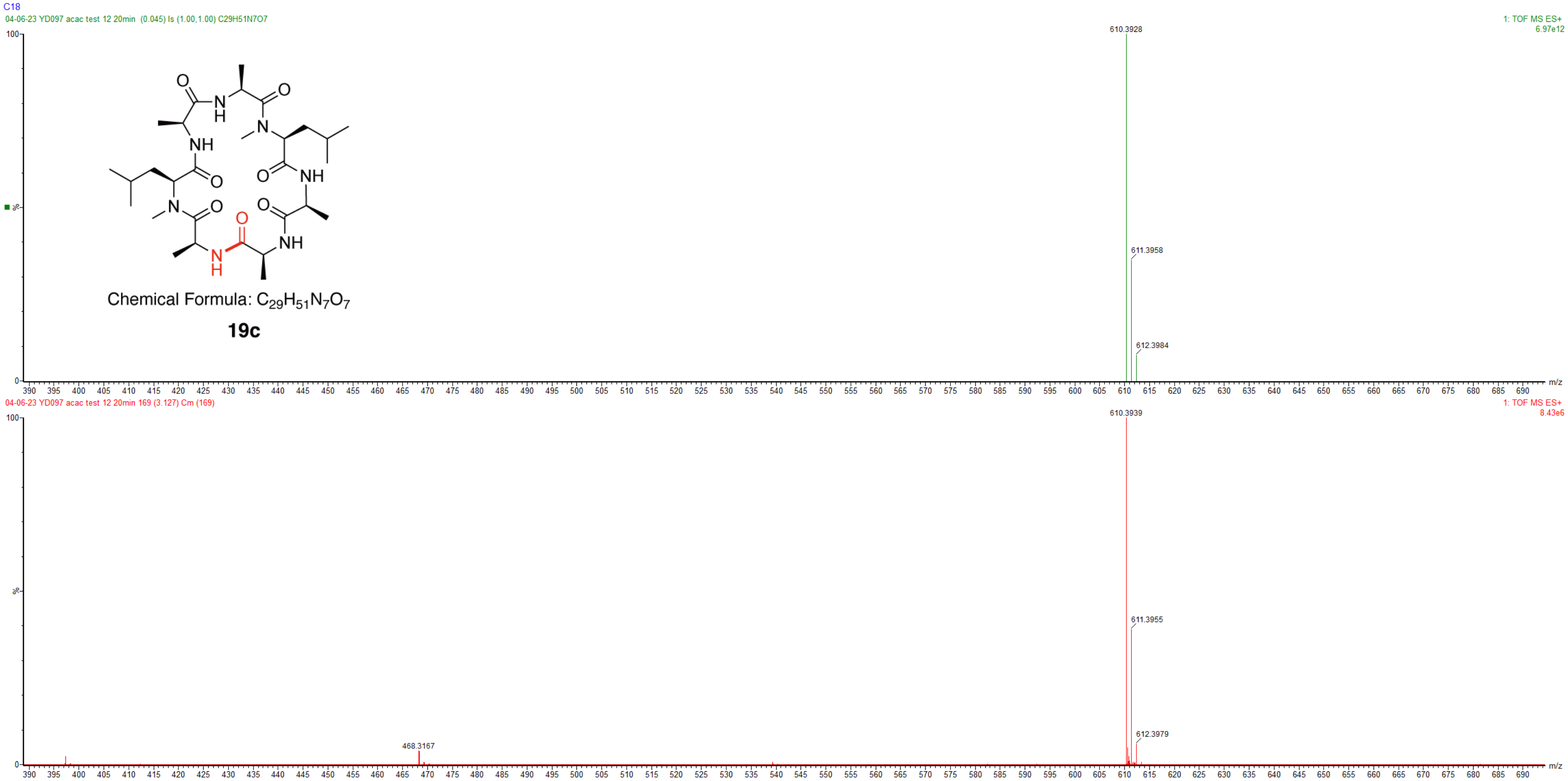

*HRMS spectrum for peptide **20a** (predicted mass spectrum (top) measured (bottom))*

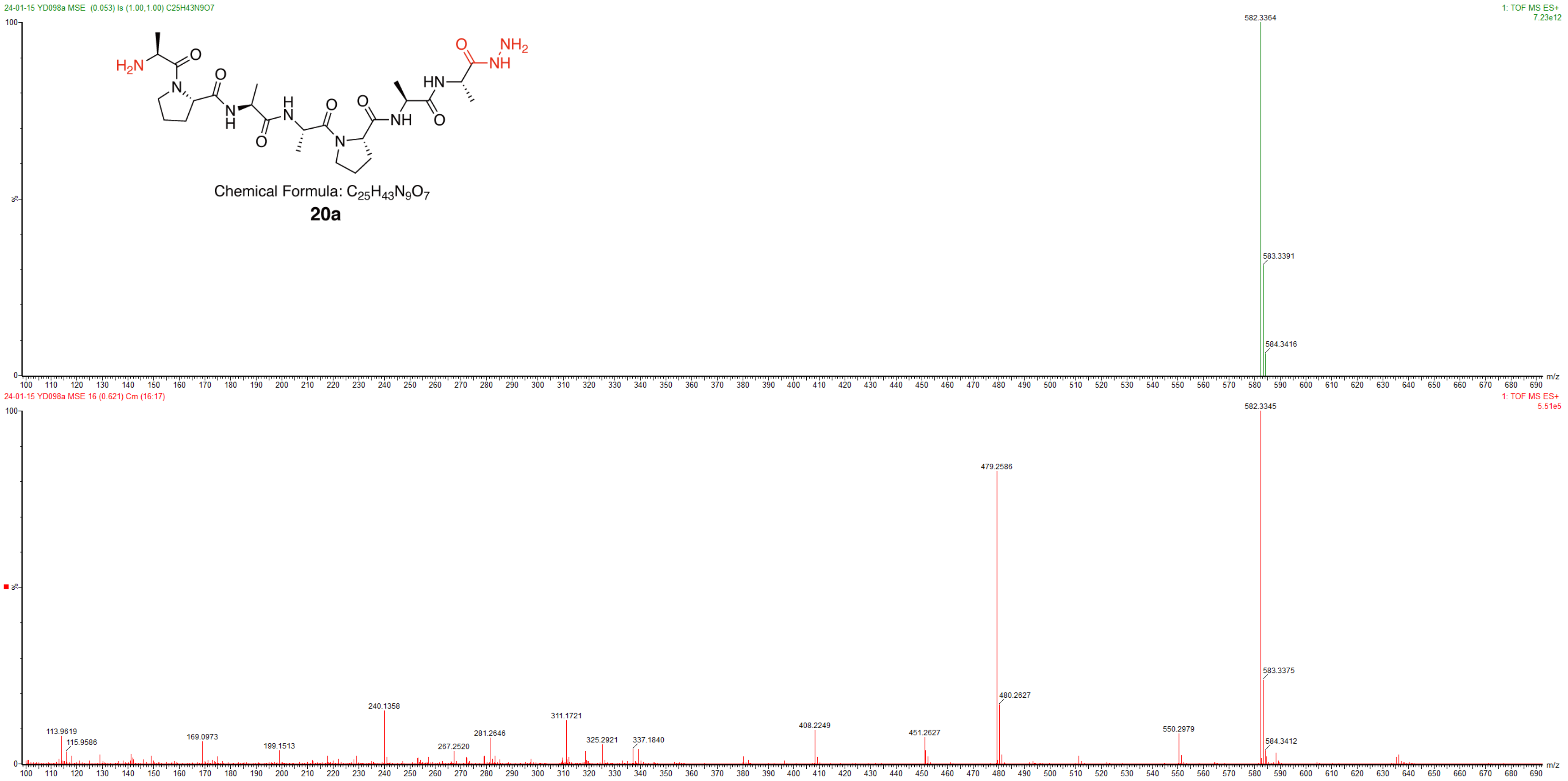

*MS^E spectrum for peptide **20a***

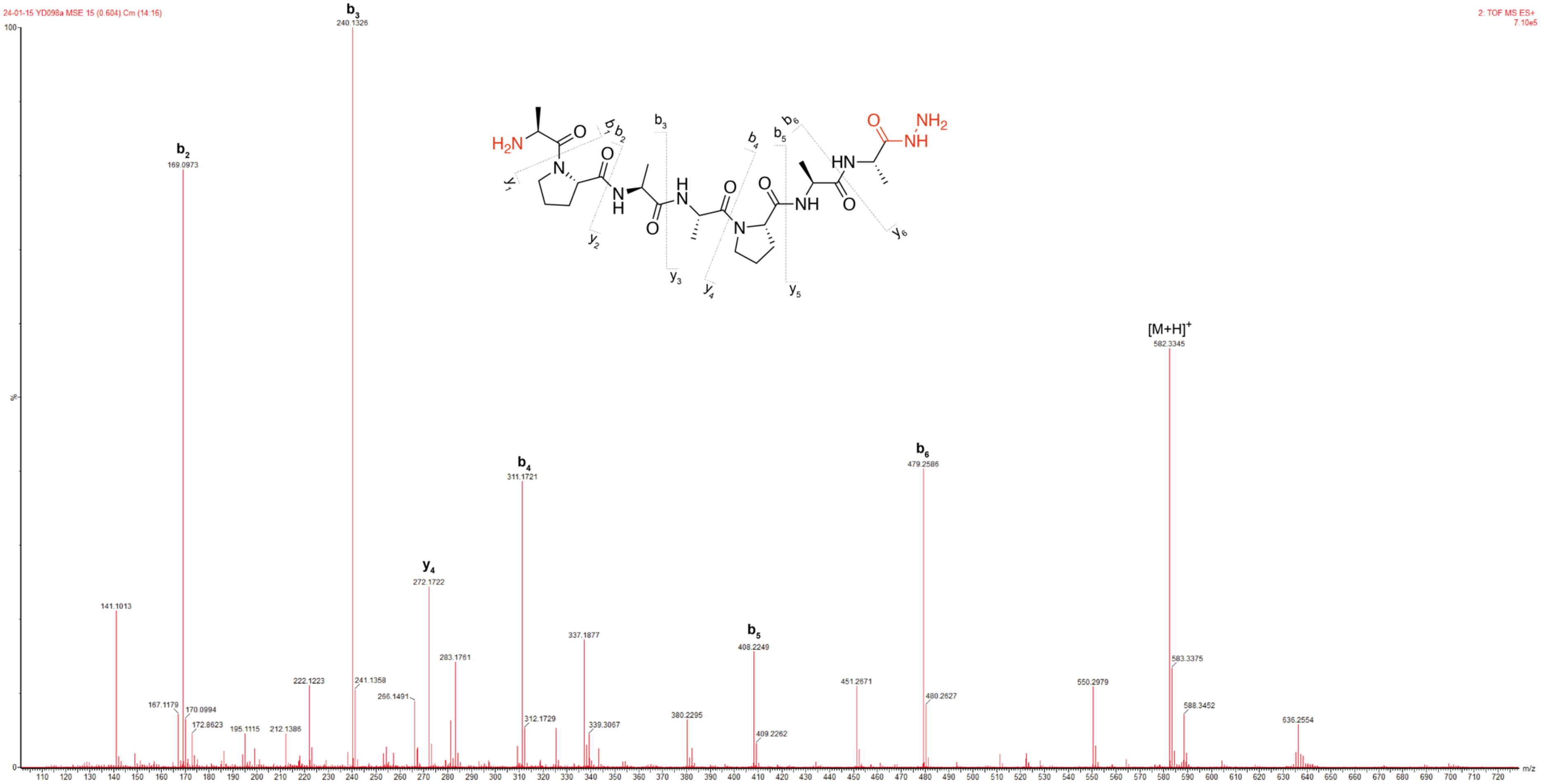

*HRMS spectrum for peptide **20c** (predicted mass spectrum (top) measured (bottom))*

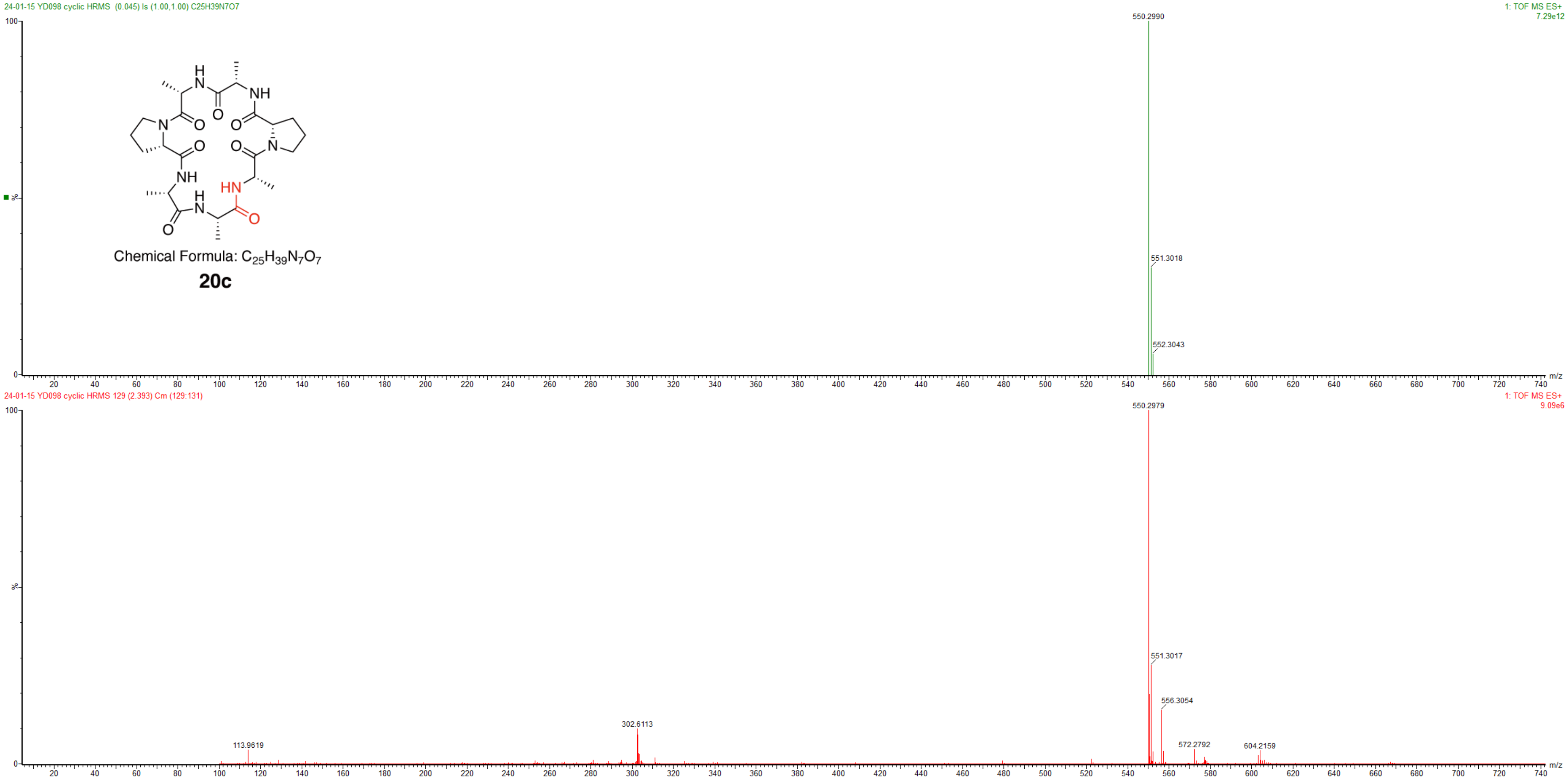

*HRMS spectrum for peptide **21a** (predicted mass spectrum (top) measured (bottom))*

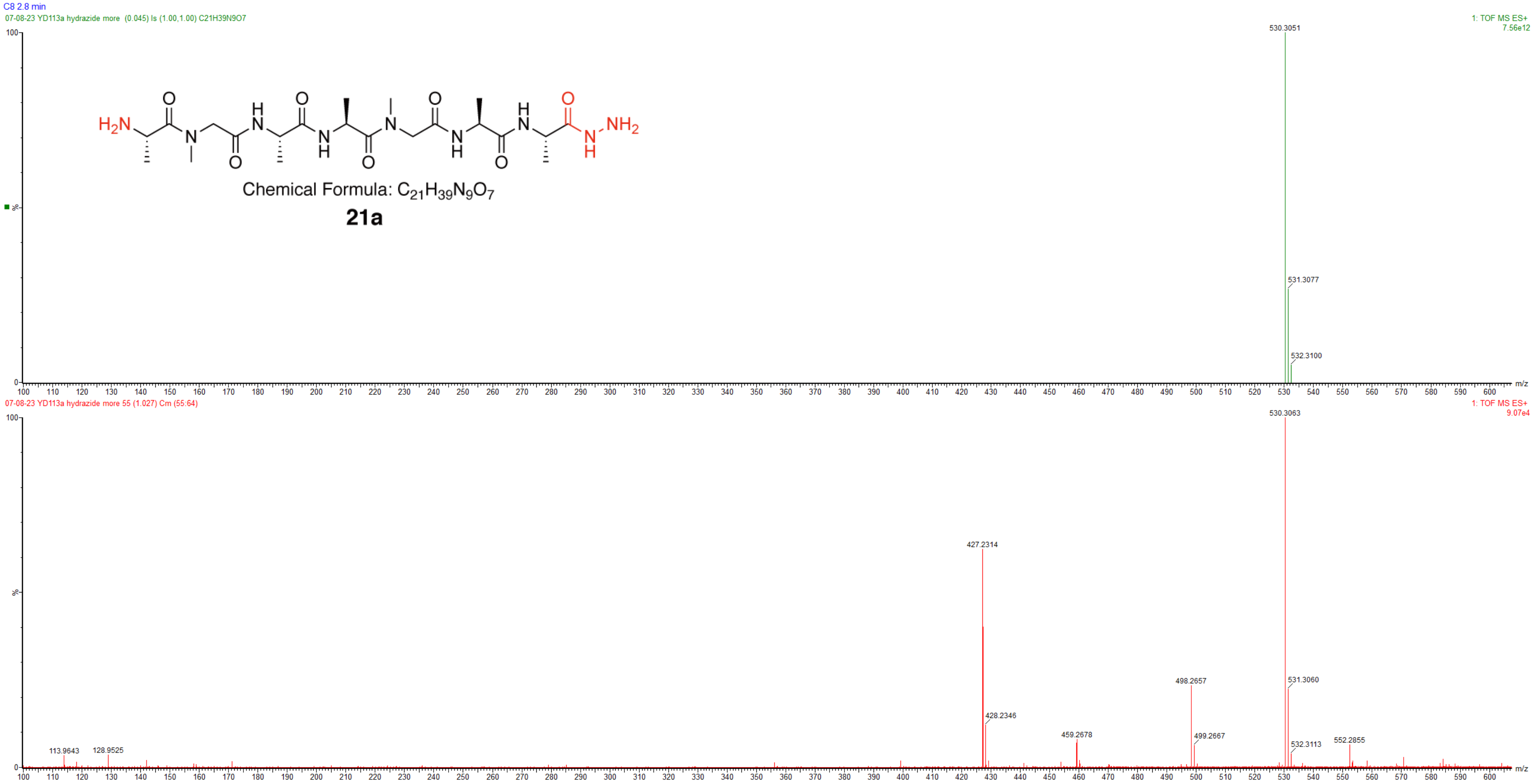

MS

*MS^E spectrum for peptide **21a***

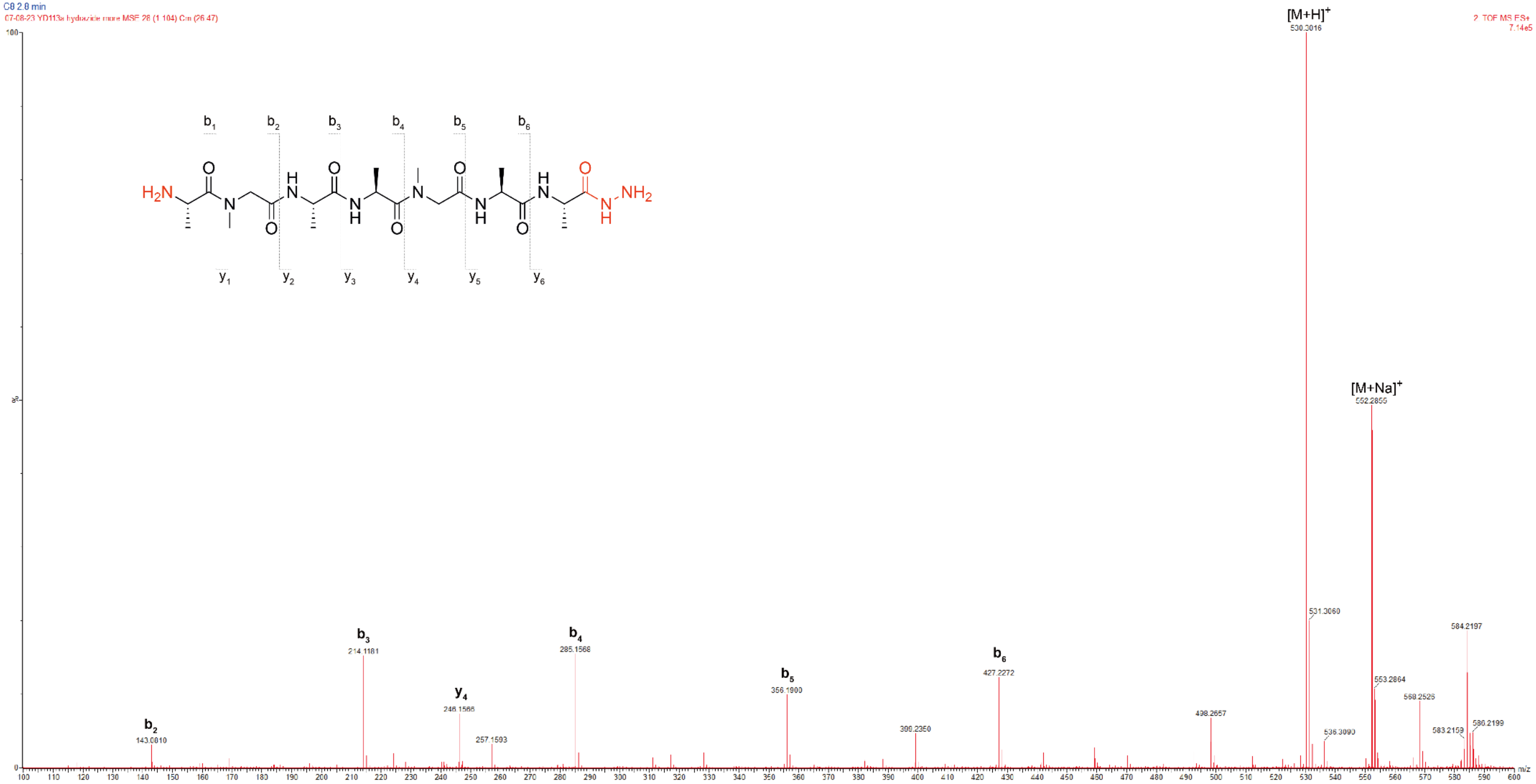

*HRMS spectrum for peptide **21c** (predicted mass spectrum (top) measured (bottom))*

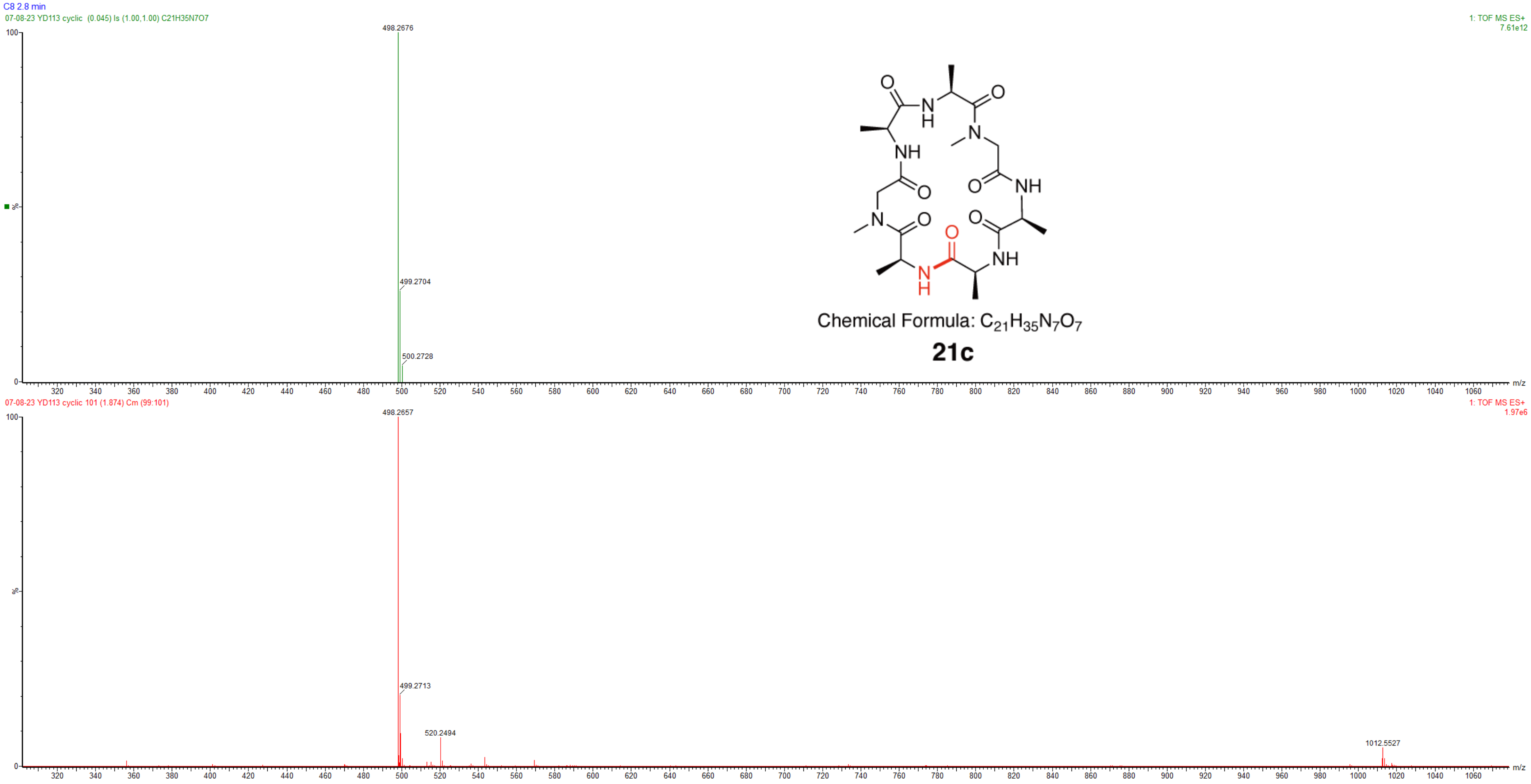

*HRMS spectrum for peptide **22a** (predicted mass spectrum (top) measured (bottom))*

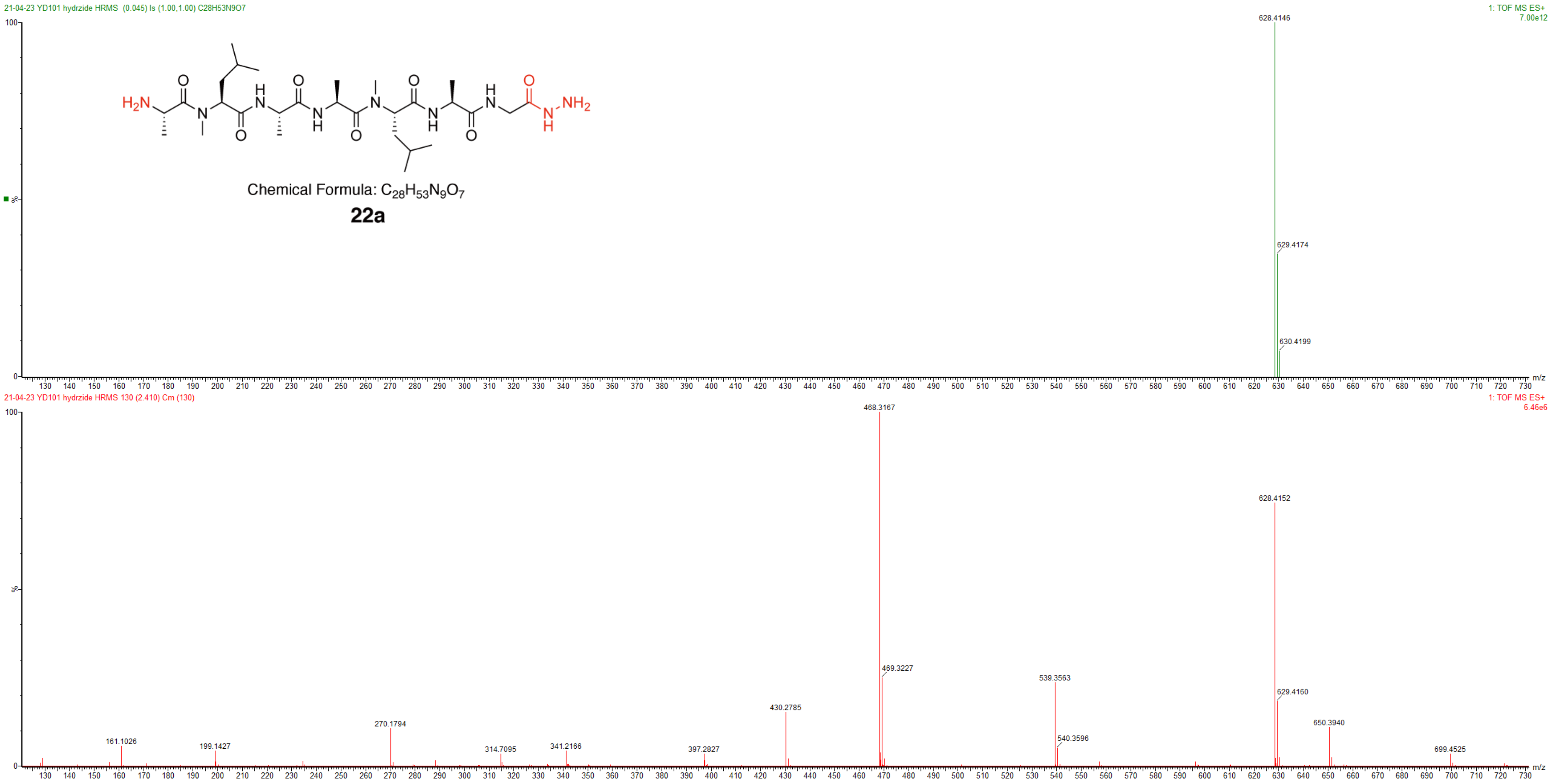

MS^E spectrum for peptide ***22a***

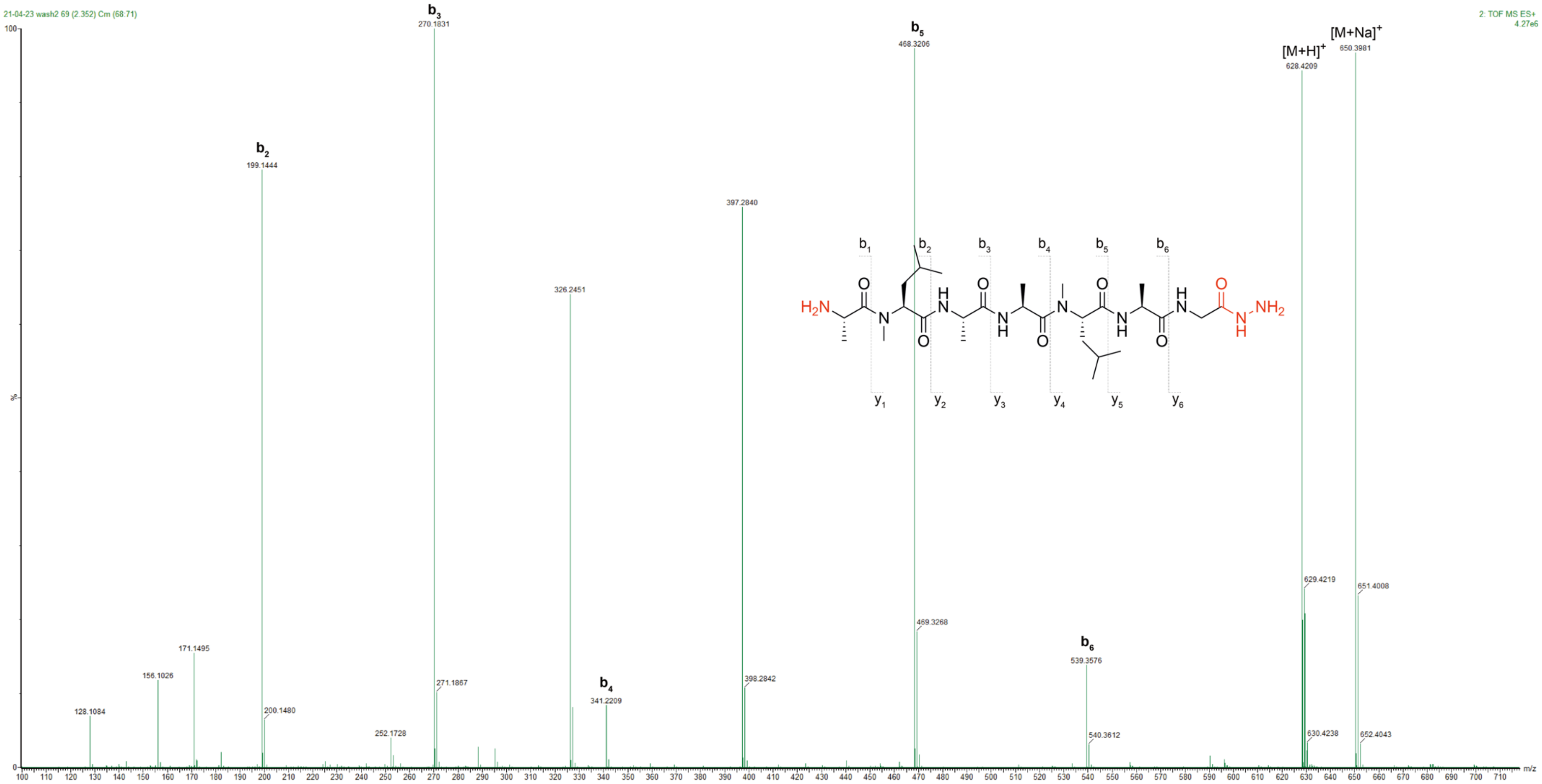

*HRMS spectrum for peptide **22c** (predicted mass spectrum (top) measured (bottom))*

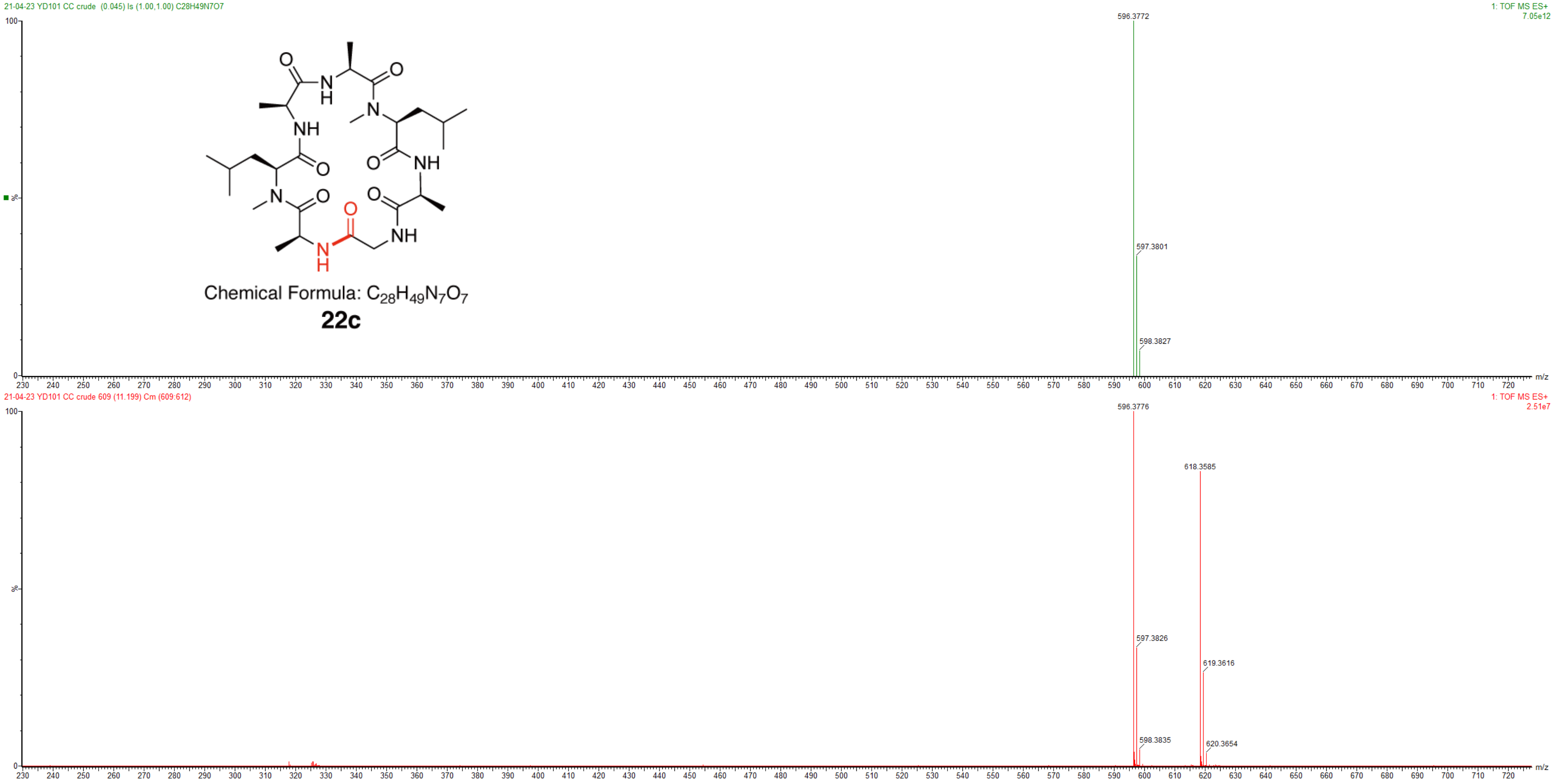

*HRMS spectrum for peptide **23a** (predicted mass spectrum (top) measured (bottom))*

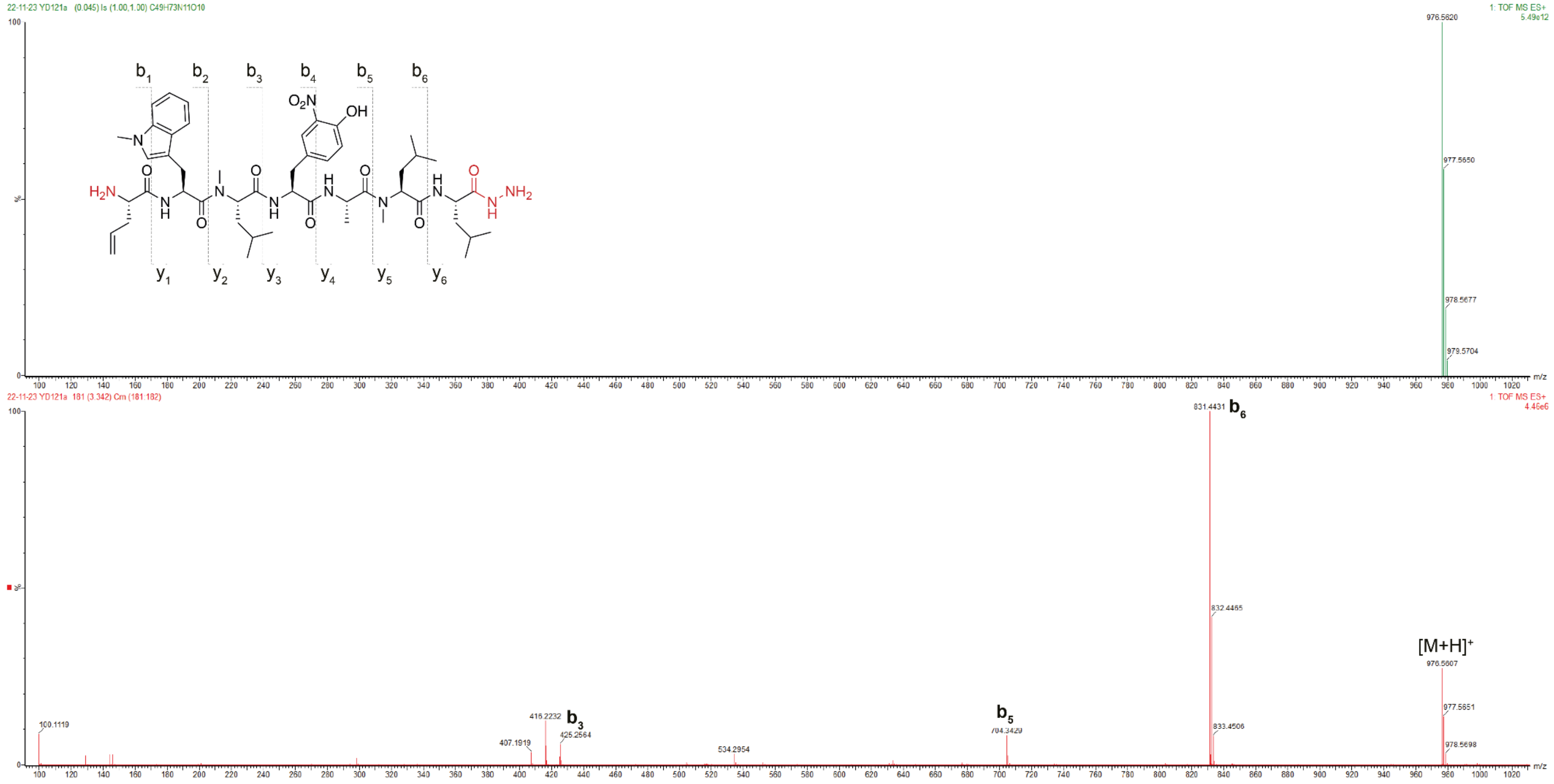

*HRMS spectrum for peptide **24a** (predicted mass spectrum (top) measured (bottom))*

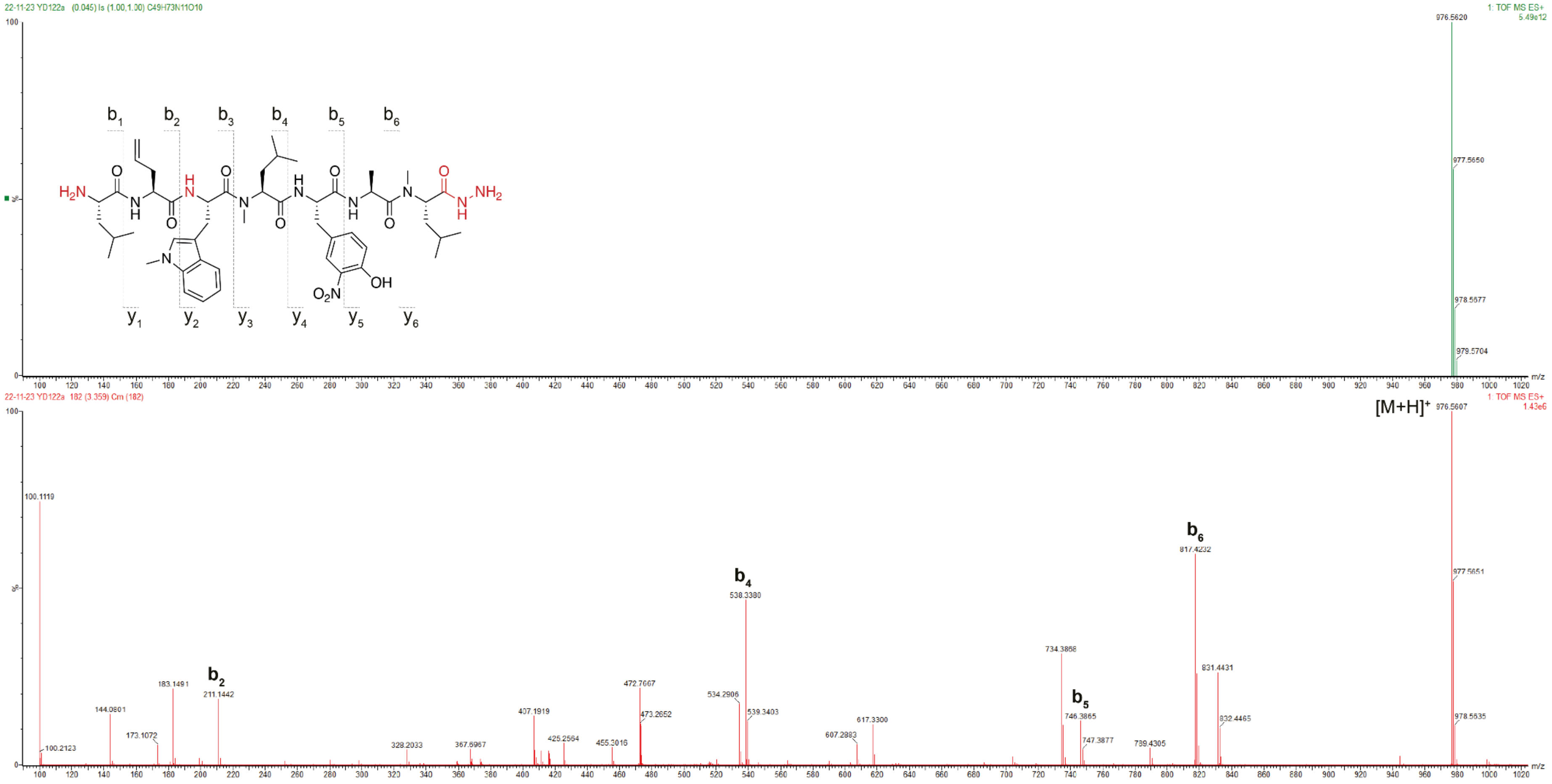

*HRMS spectrum for peptide **23c & 24c** (the products of these reactions are the same structure as **10c**). The spectra given confirm they are the same products.*

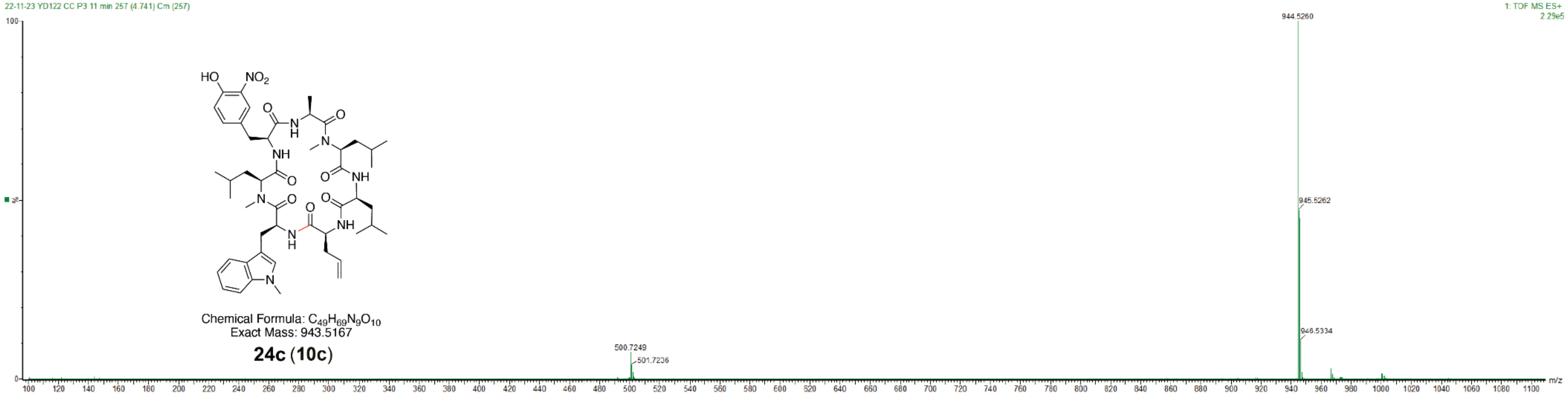

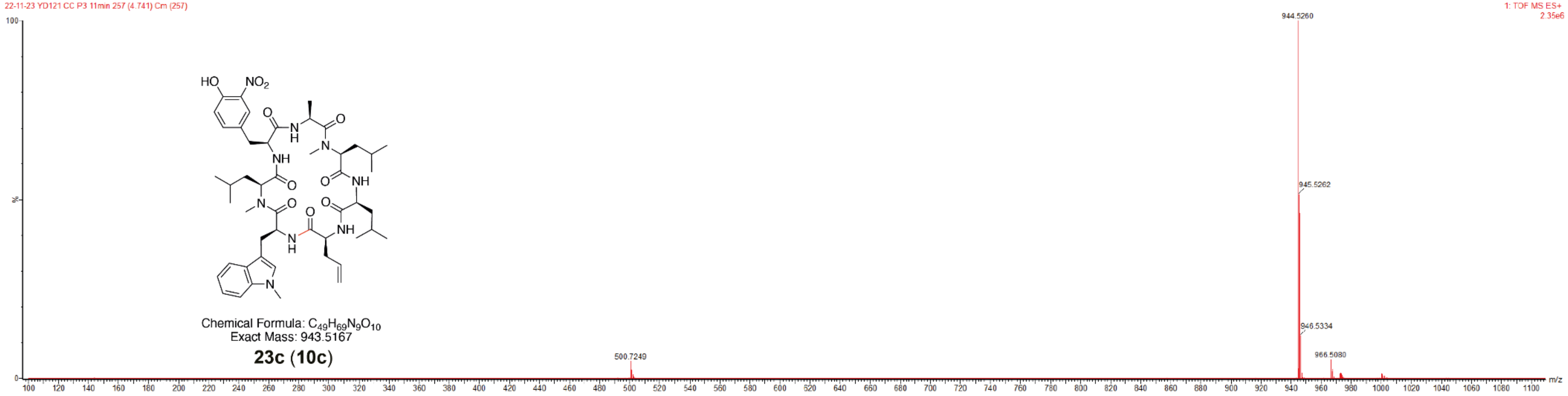

*HRMS spectrum for peptide **25a** (predicted mass spectrum (top) measured (bottom))*

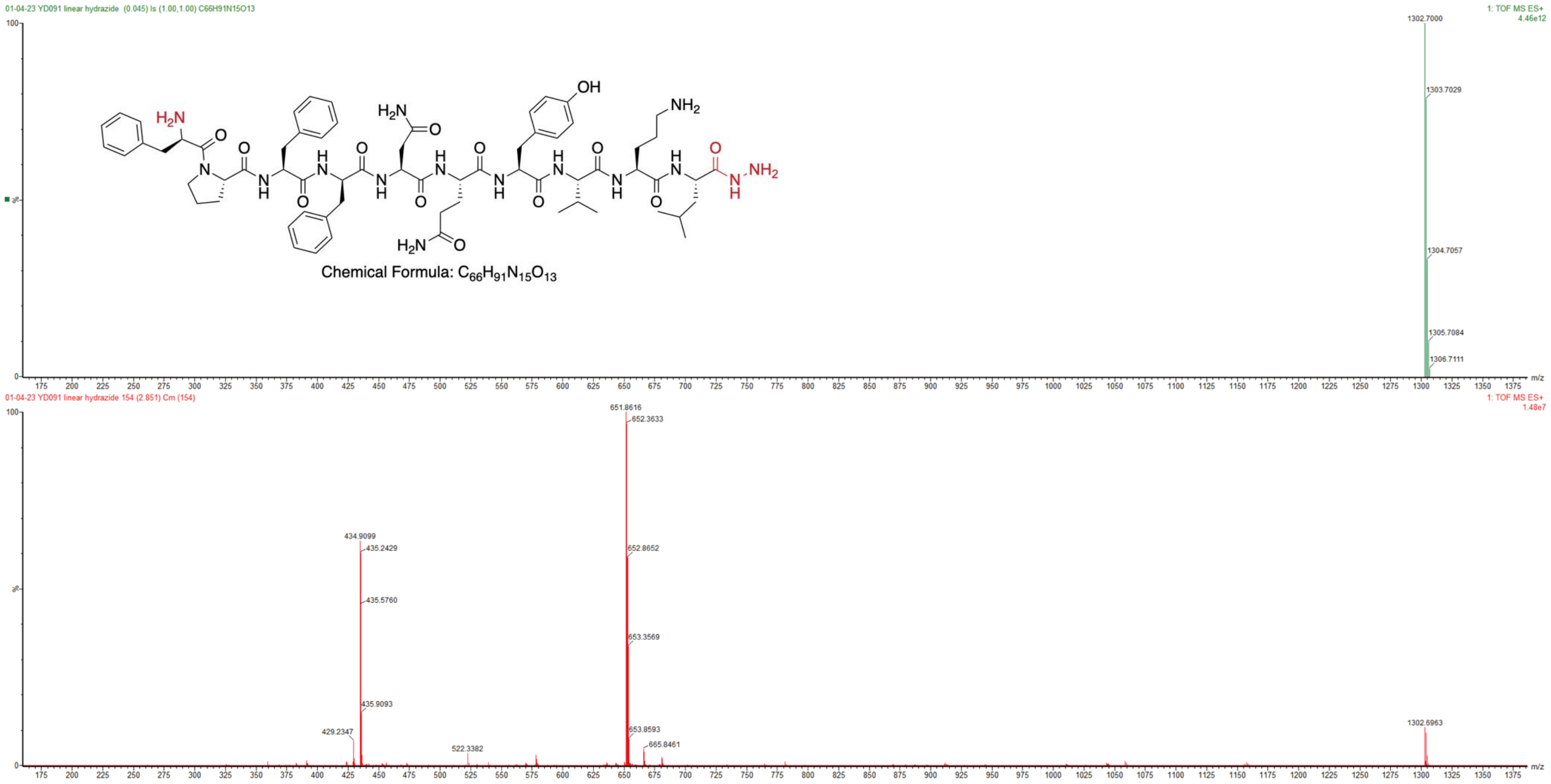

MS^E spectrum for peptide ***25a***

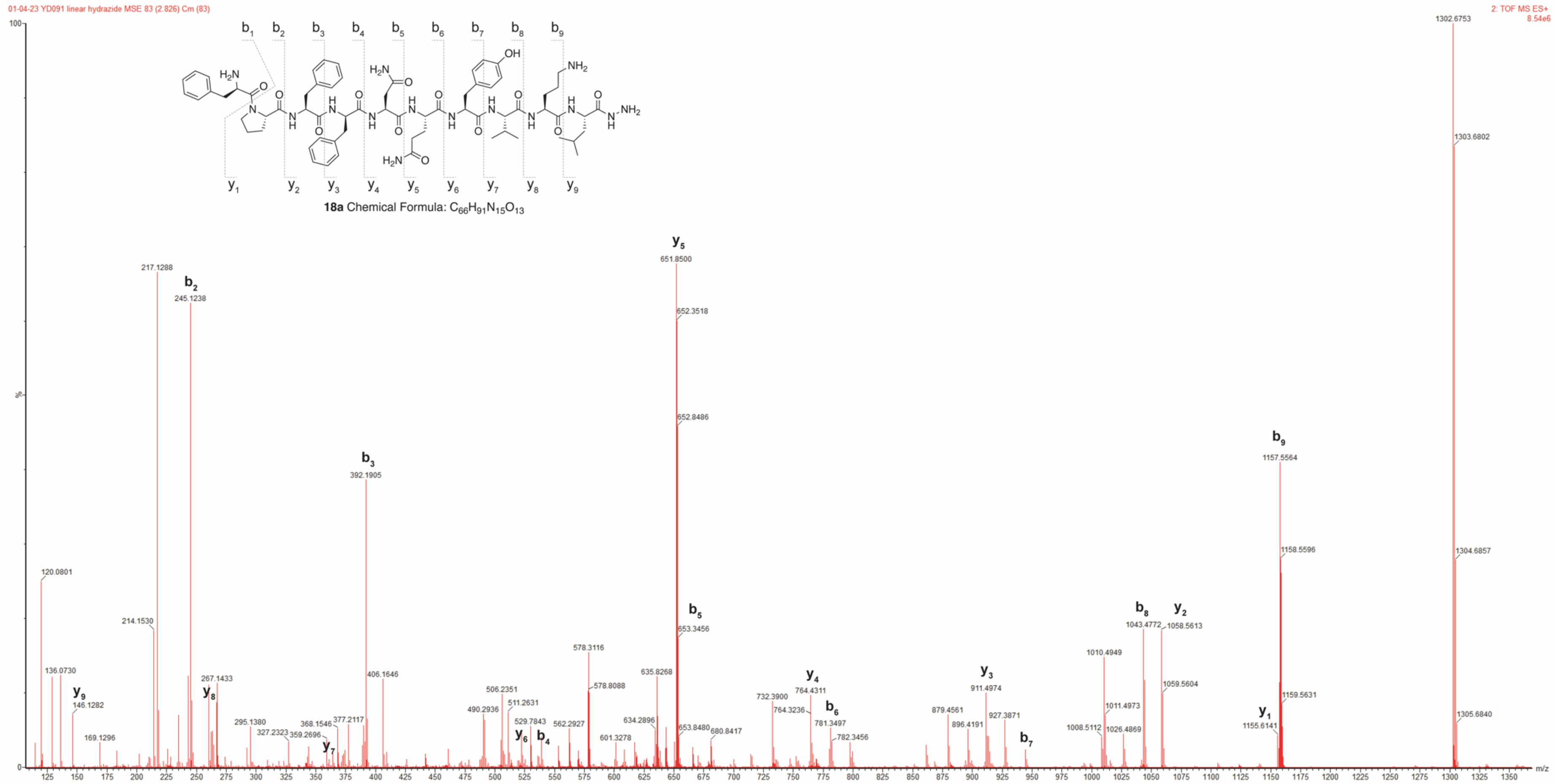

*HRMS spectrum for peptide **25c** (predicted mass spectrum (top) measured (bottom))*

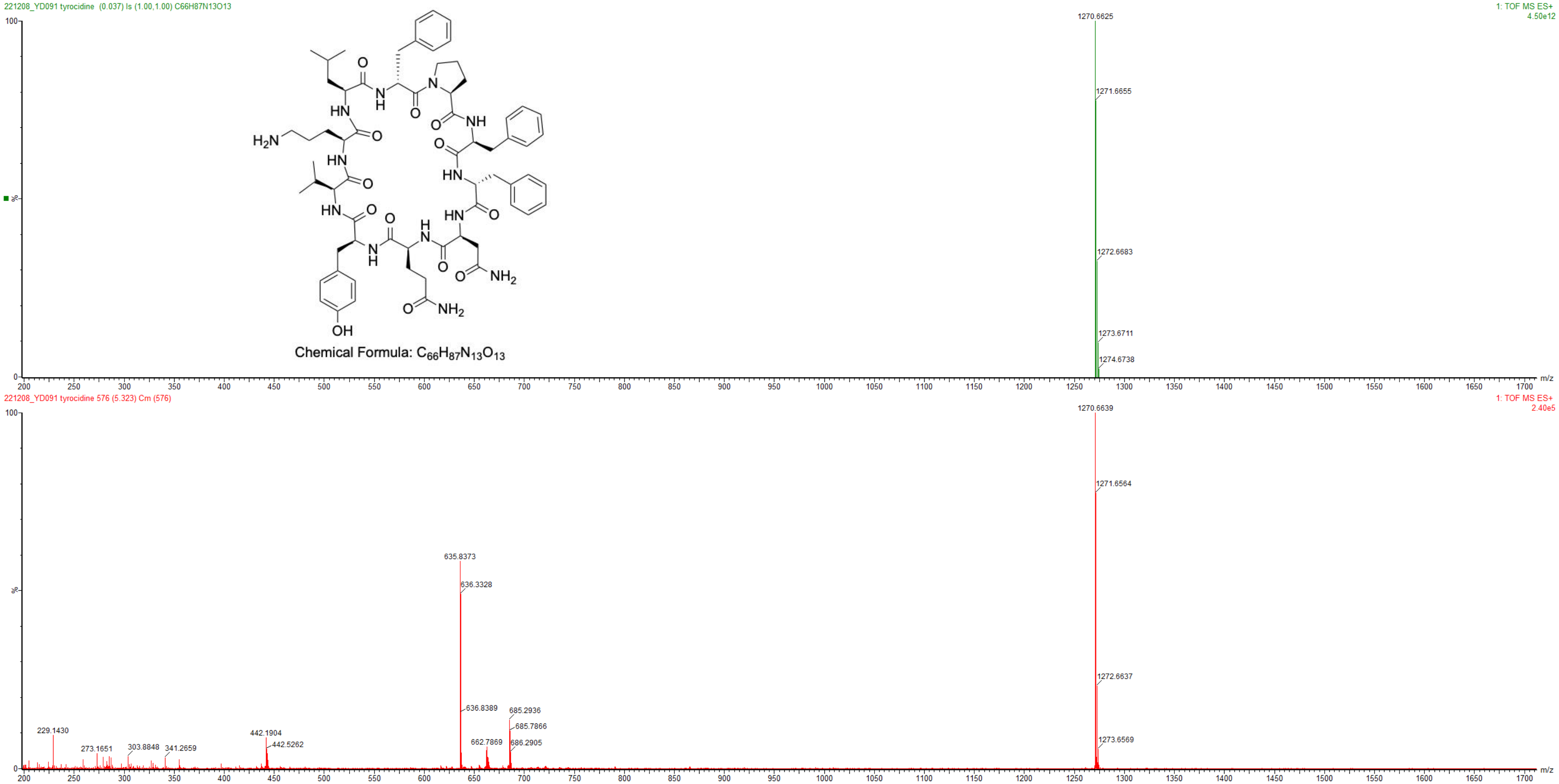

*HRMS spectrum for peptide **26a** (predicted mass spectrum (top) measured (bottom))*

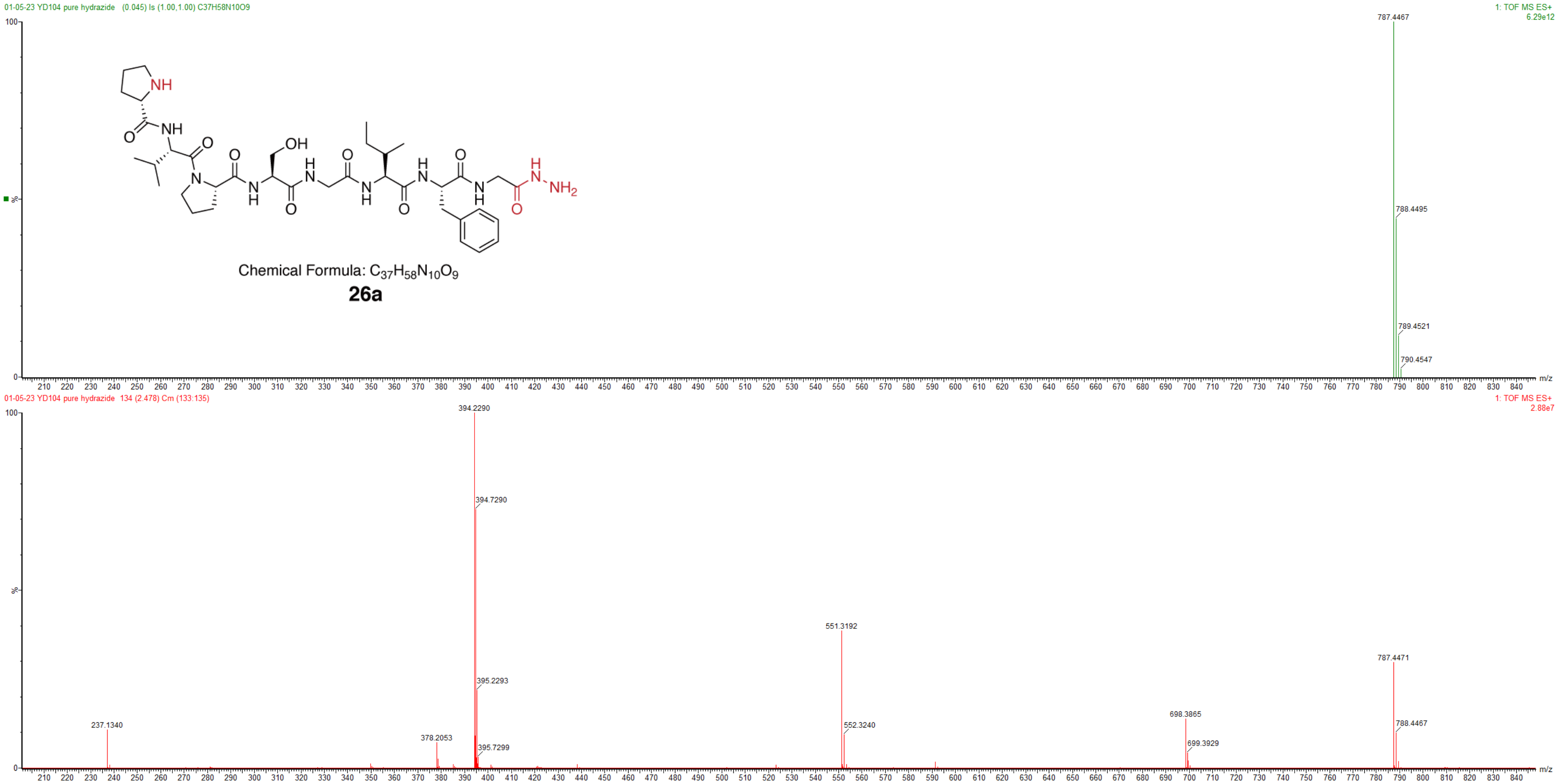

*MS^E spectrum for peptide **26a***

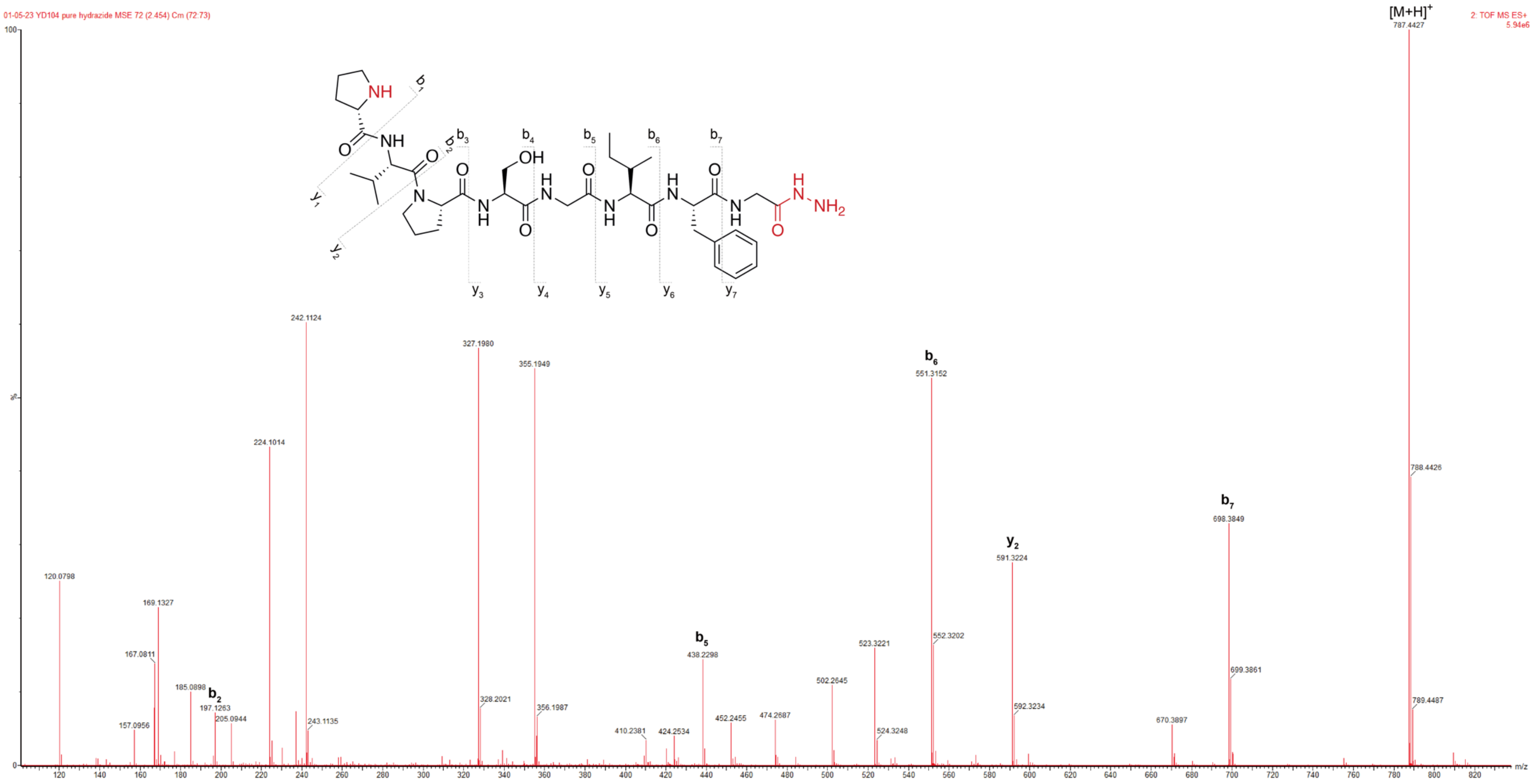

*HRMS spectrum for peptide **26c** (predicted mass spectrum (top) measured (bottom))*

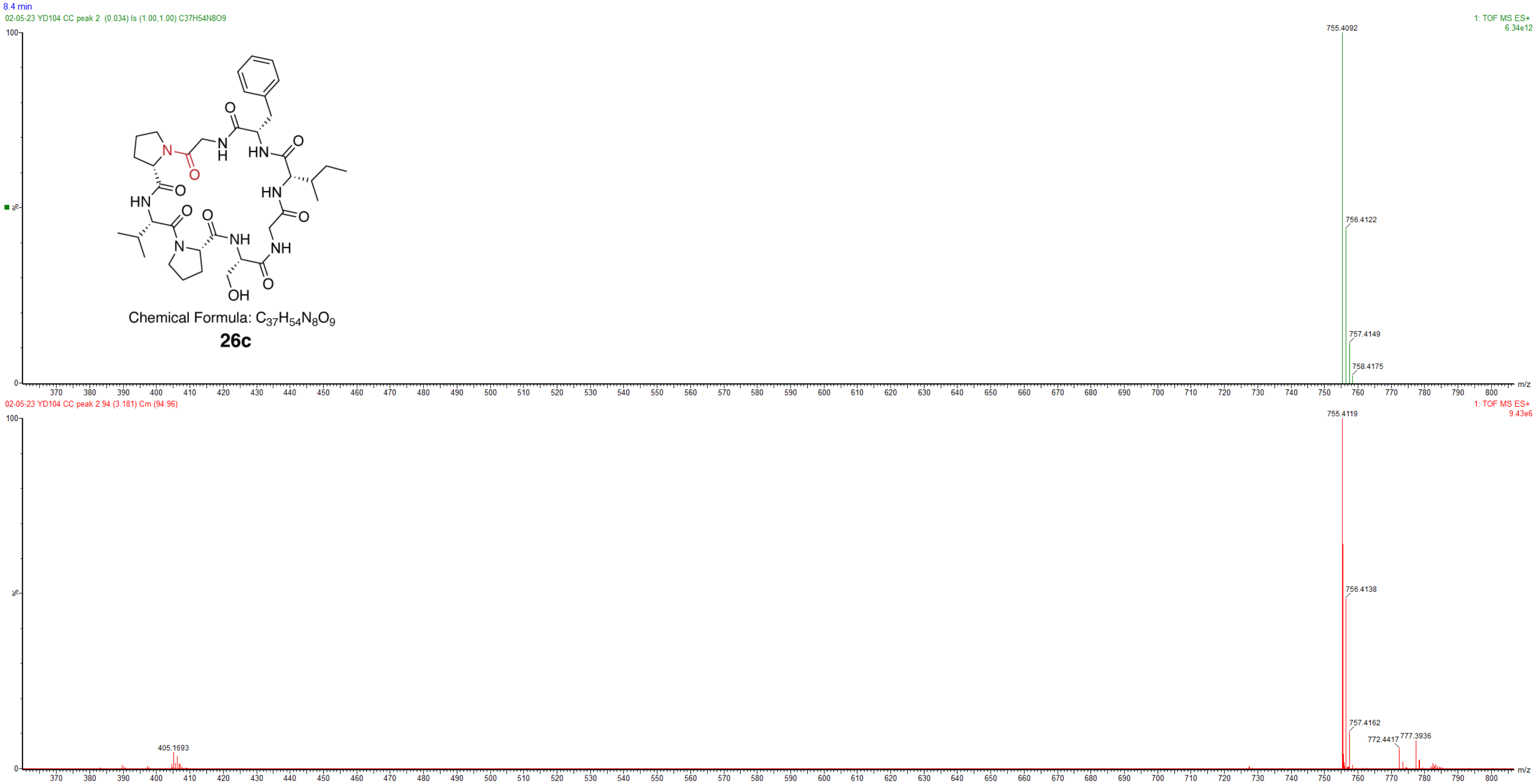

*HRMS spectrum for peptide **27a** (predicted mass spectrum (top) measured (bottom))*

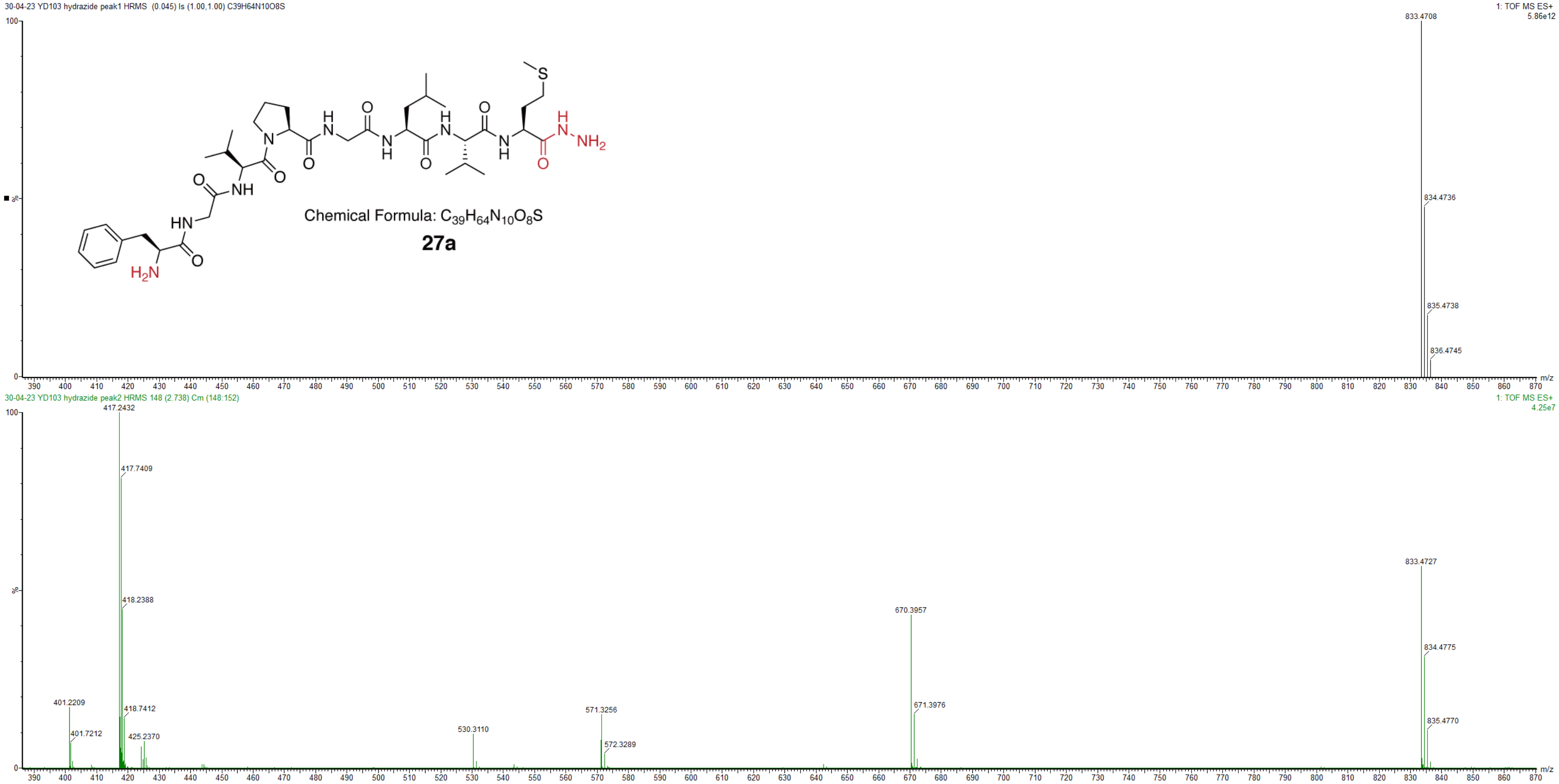

MS^E spectrum for peptide ***27a***

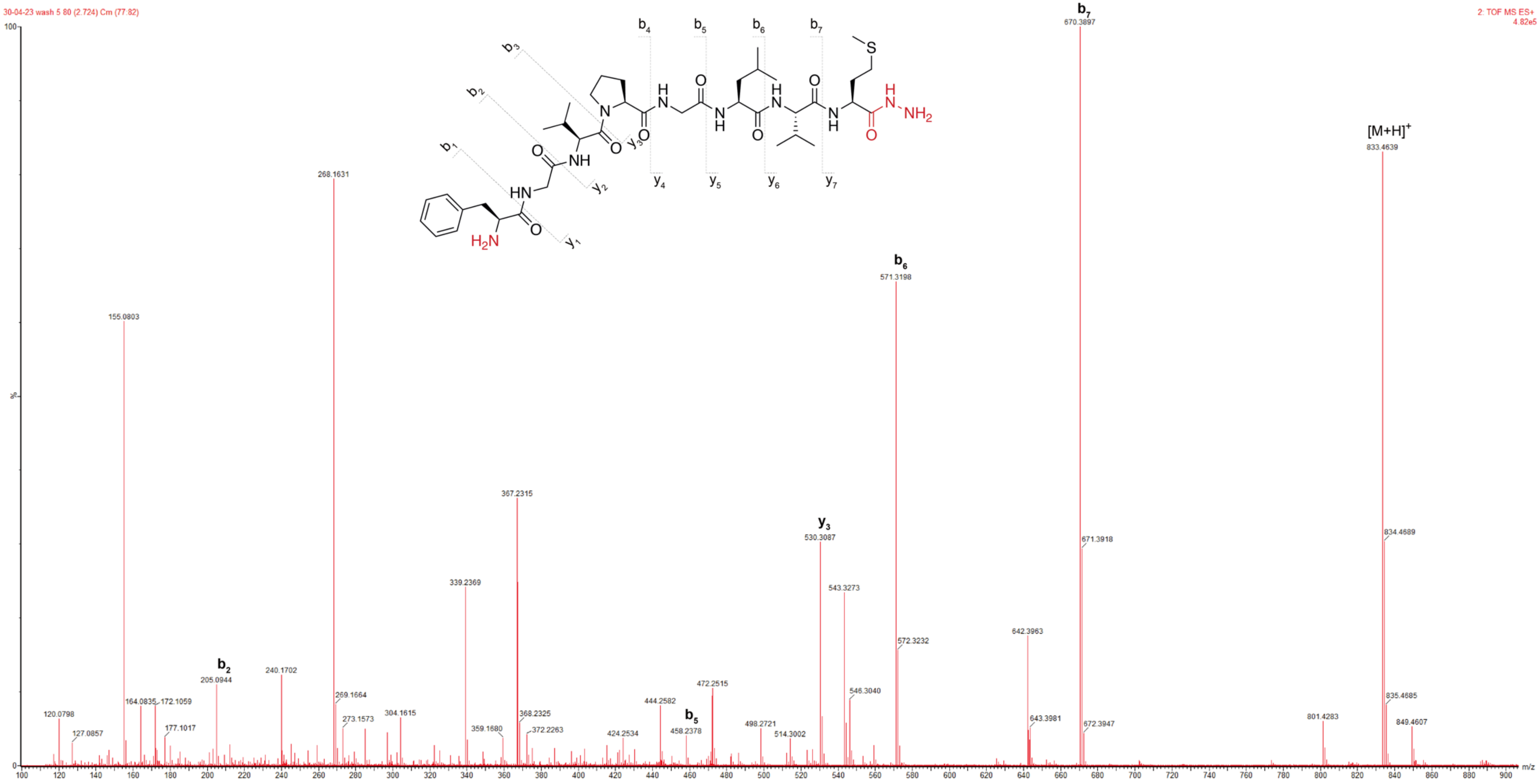

*HRMS spectrum for peptide **27c** (predicted mass spectrum (top) measured (bottom))*

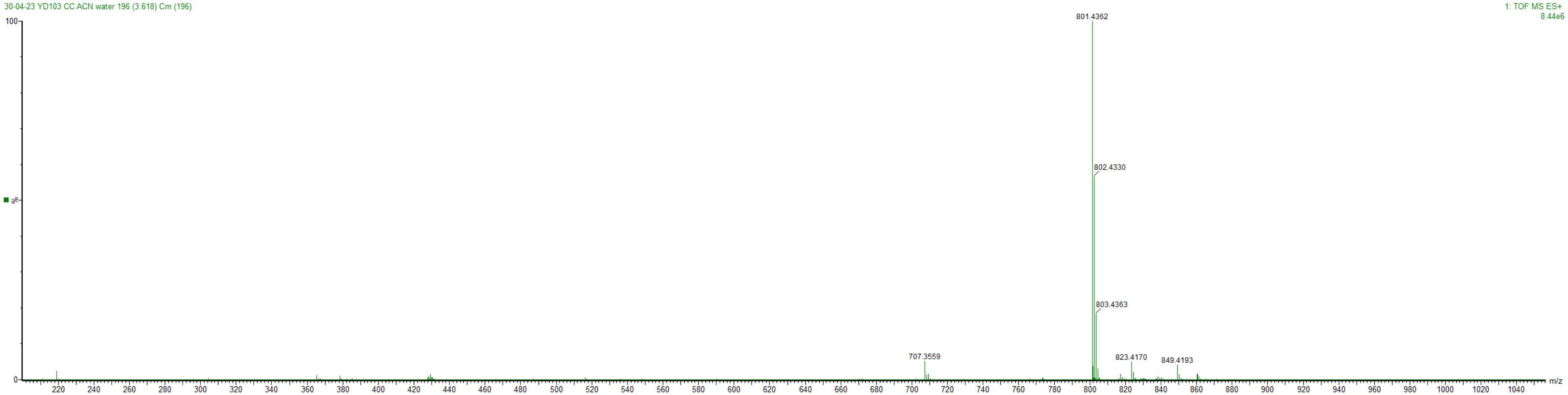

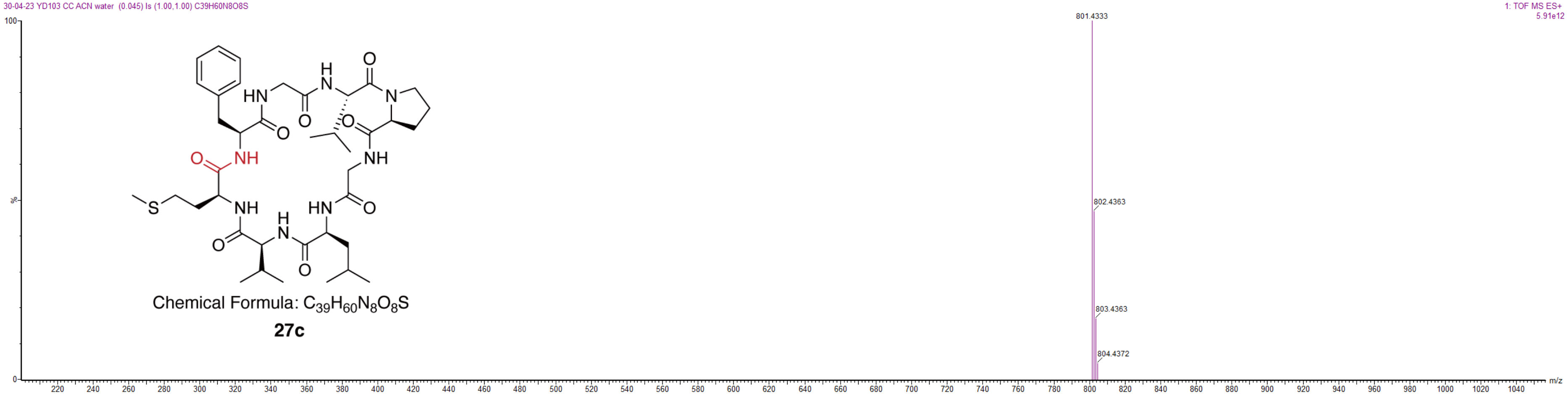

*HRMS spectrum for peptide **28a** (predicted mass spectrum (top) measured (bottom))*

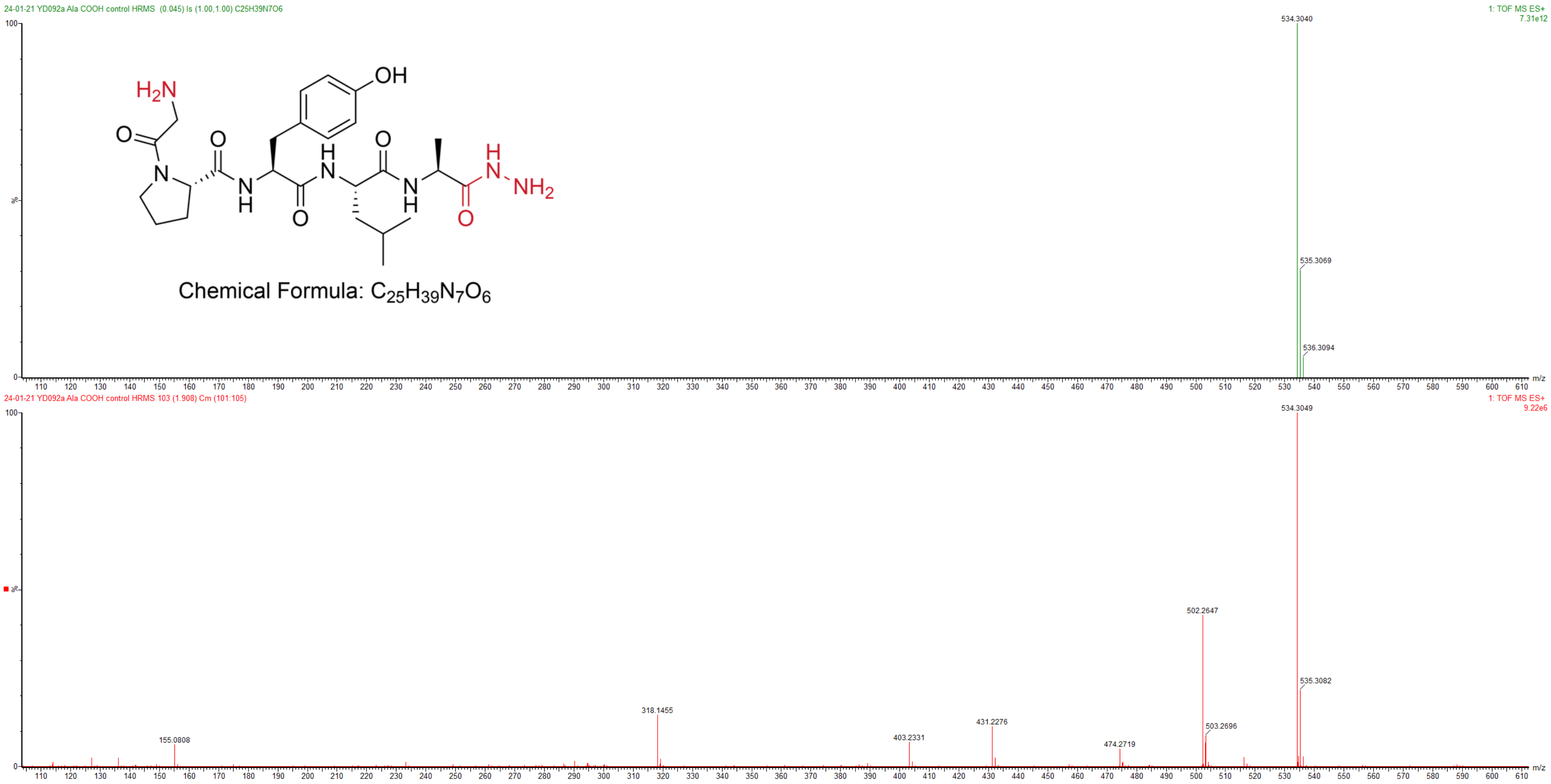

*MS^E spectrum for peptide **28a***

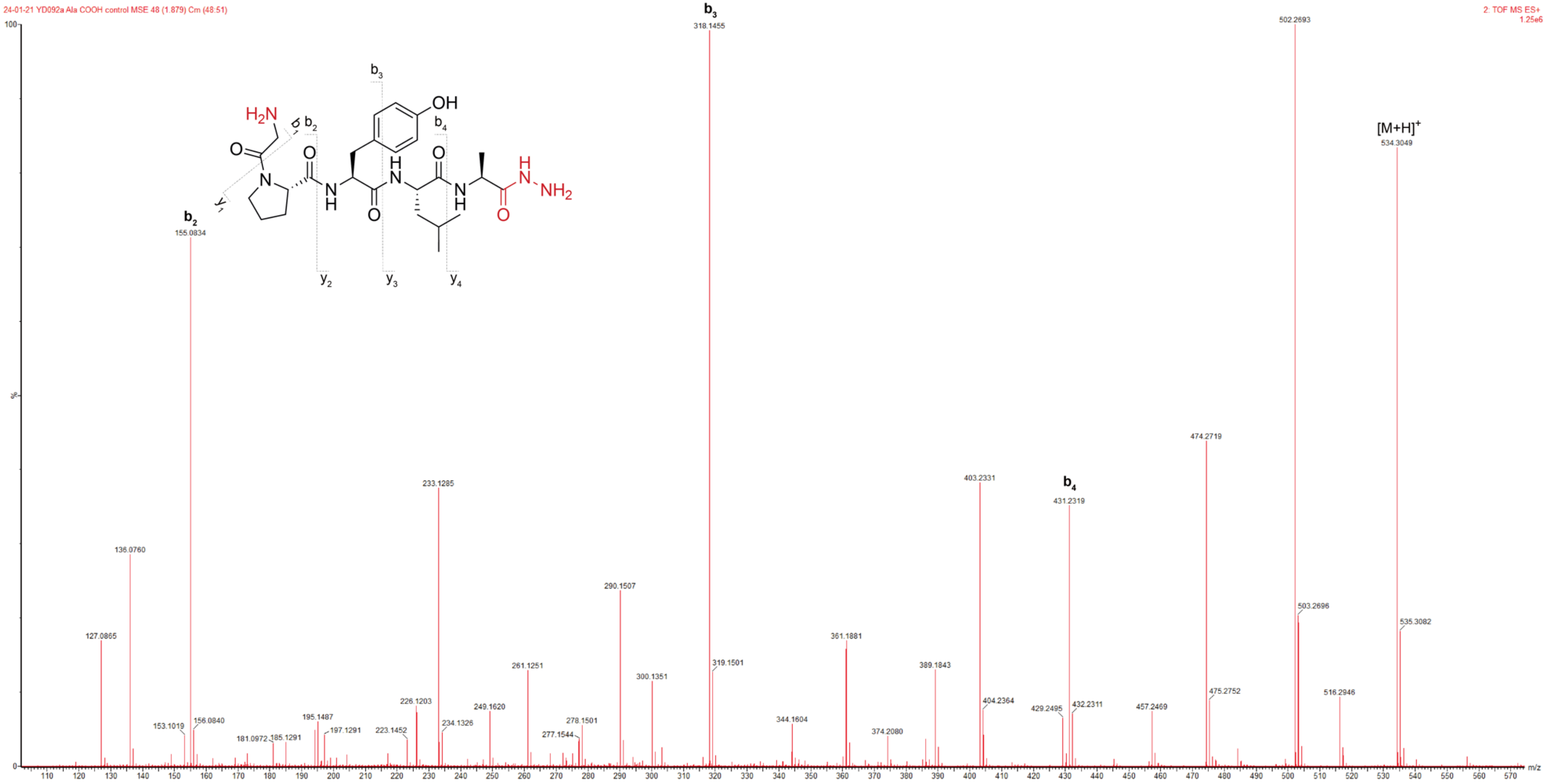

*HRMS spectrum for peptide **28c** (predicted mass spectrum (top) measured (bottom))*

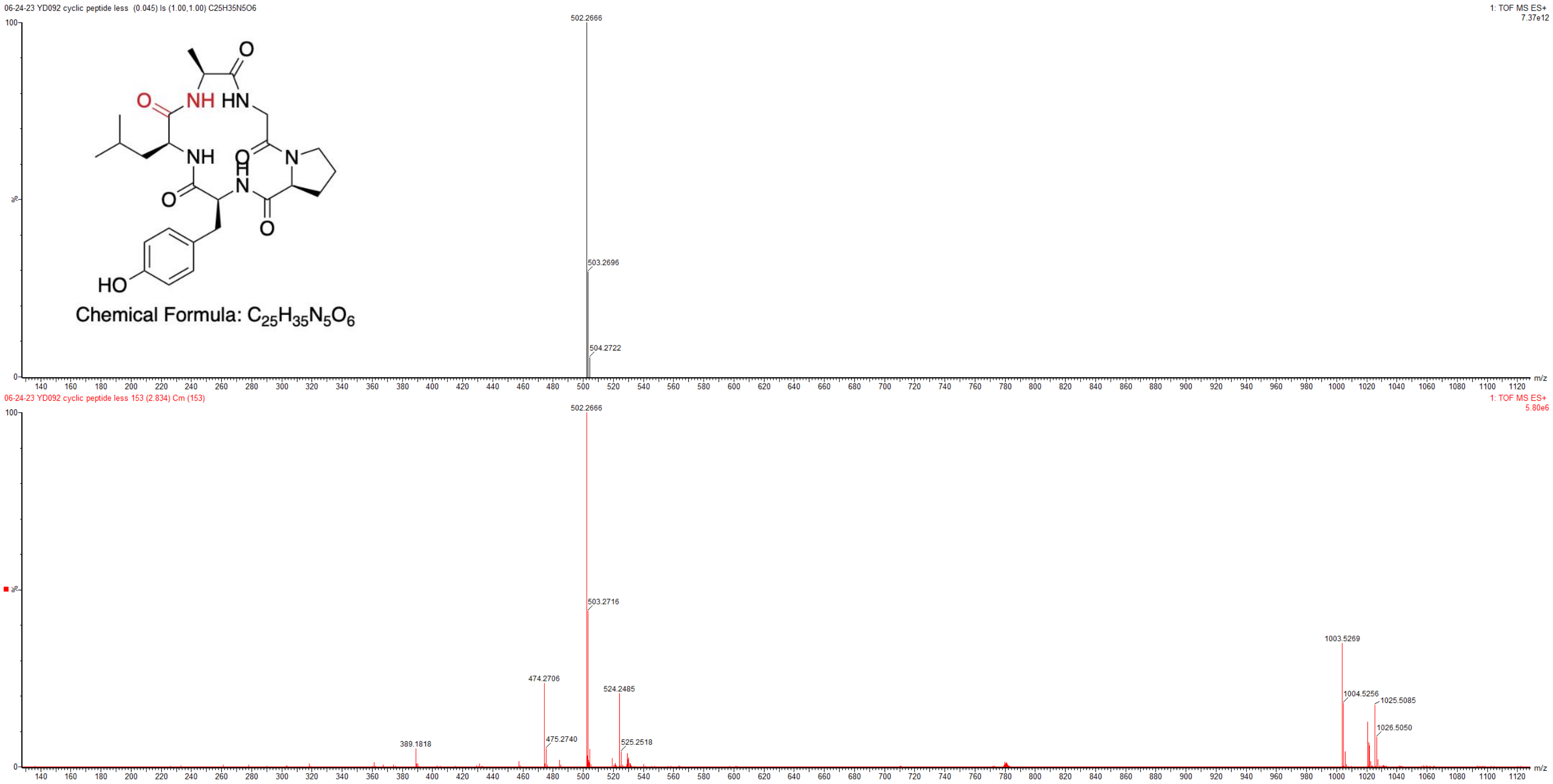

*HRMS spectrum for peptide **29a** (predicted mass spectrum (top and middle) measured (bottom))*

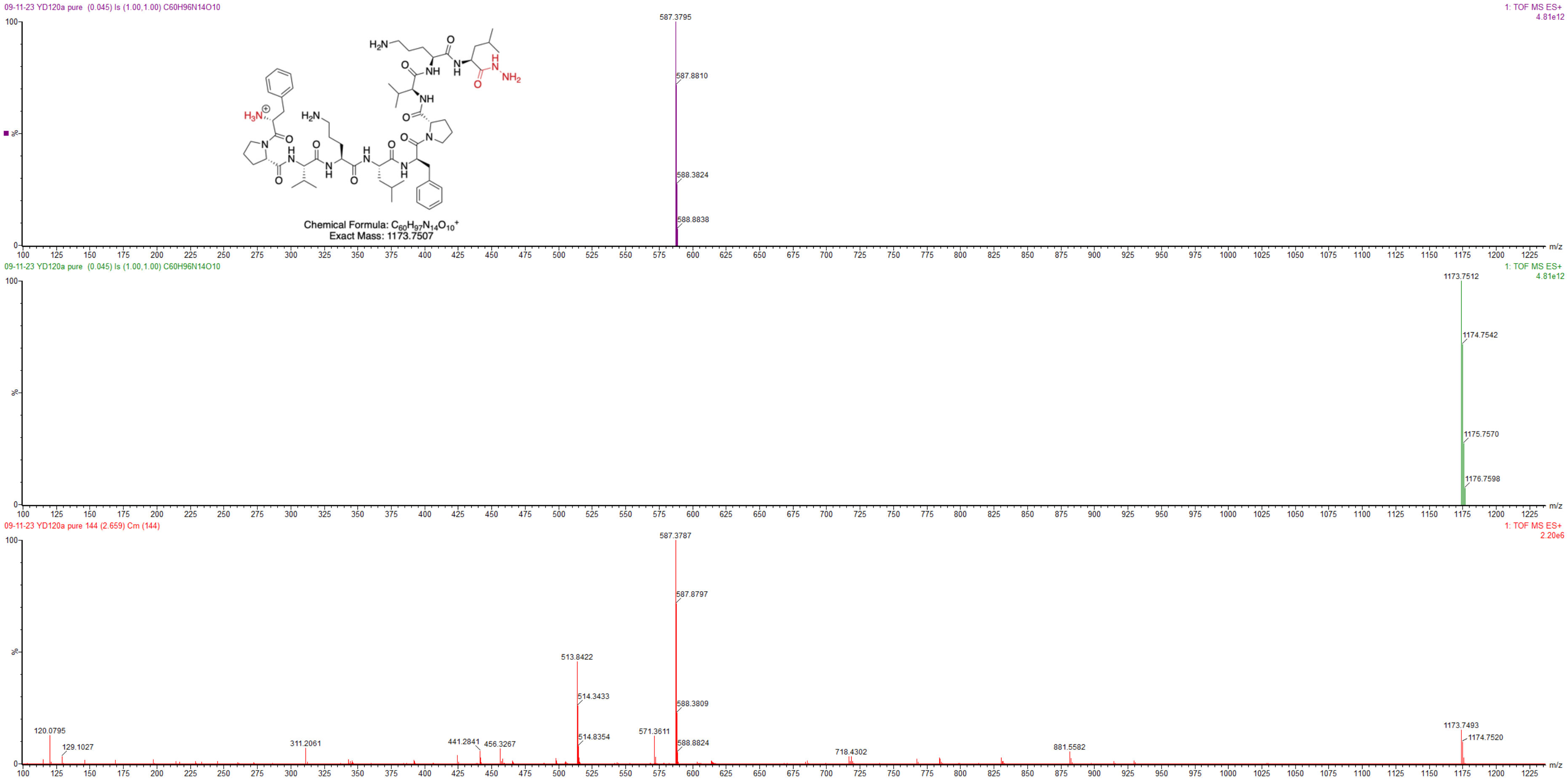

*MS^E spectrum for peptide **29a***

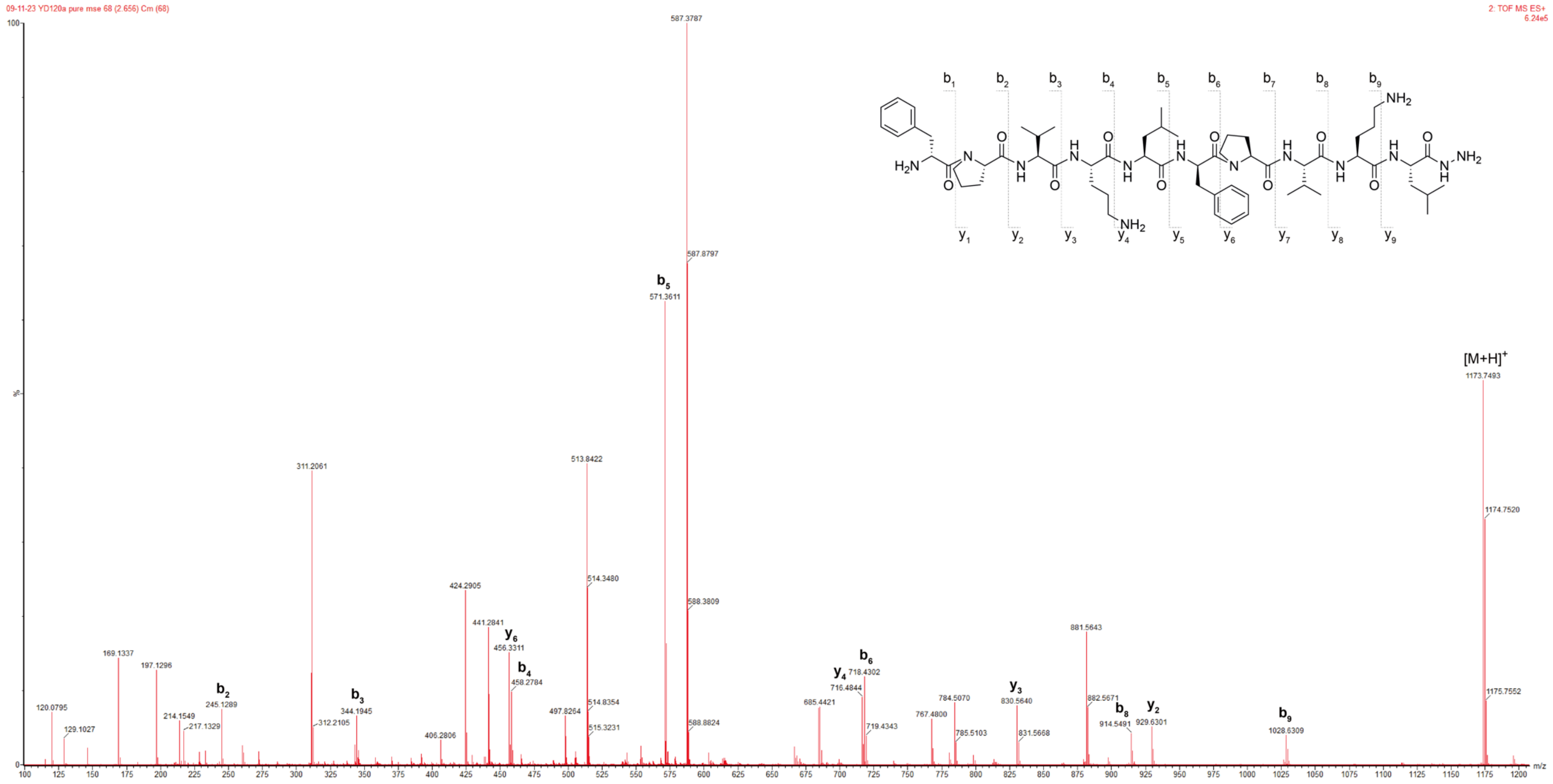

*HRMS spectrum for peptide **29c** (predicted mass spectrum (top and middle) measured (bottom))*

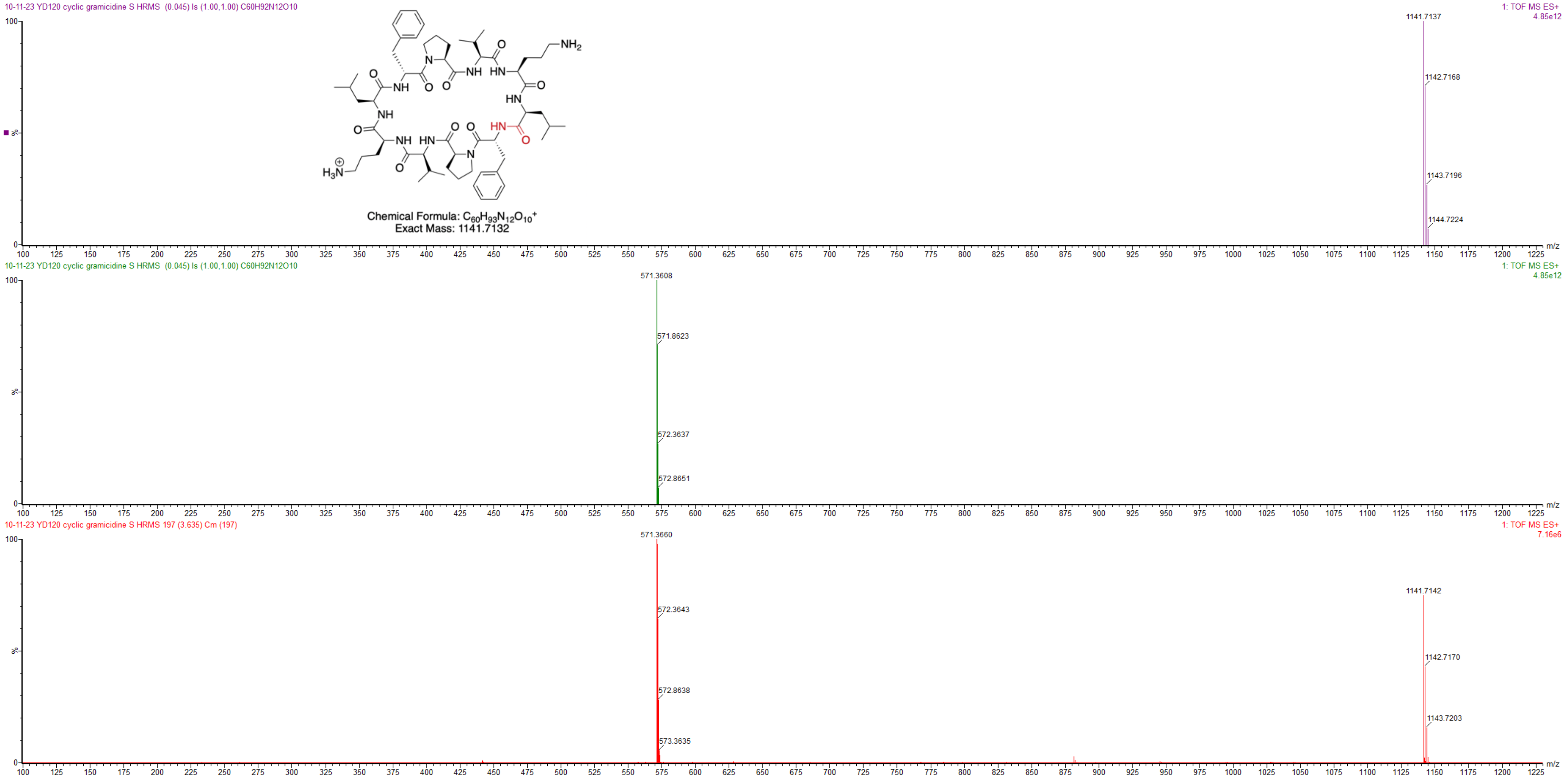

*HRMS spectrum for peptide **31a** (predicted mass spectrum (top) measured (bottom)*

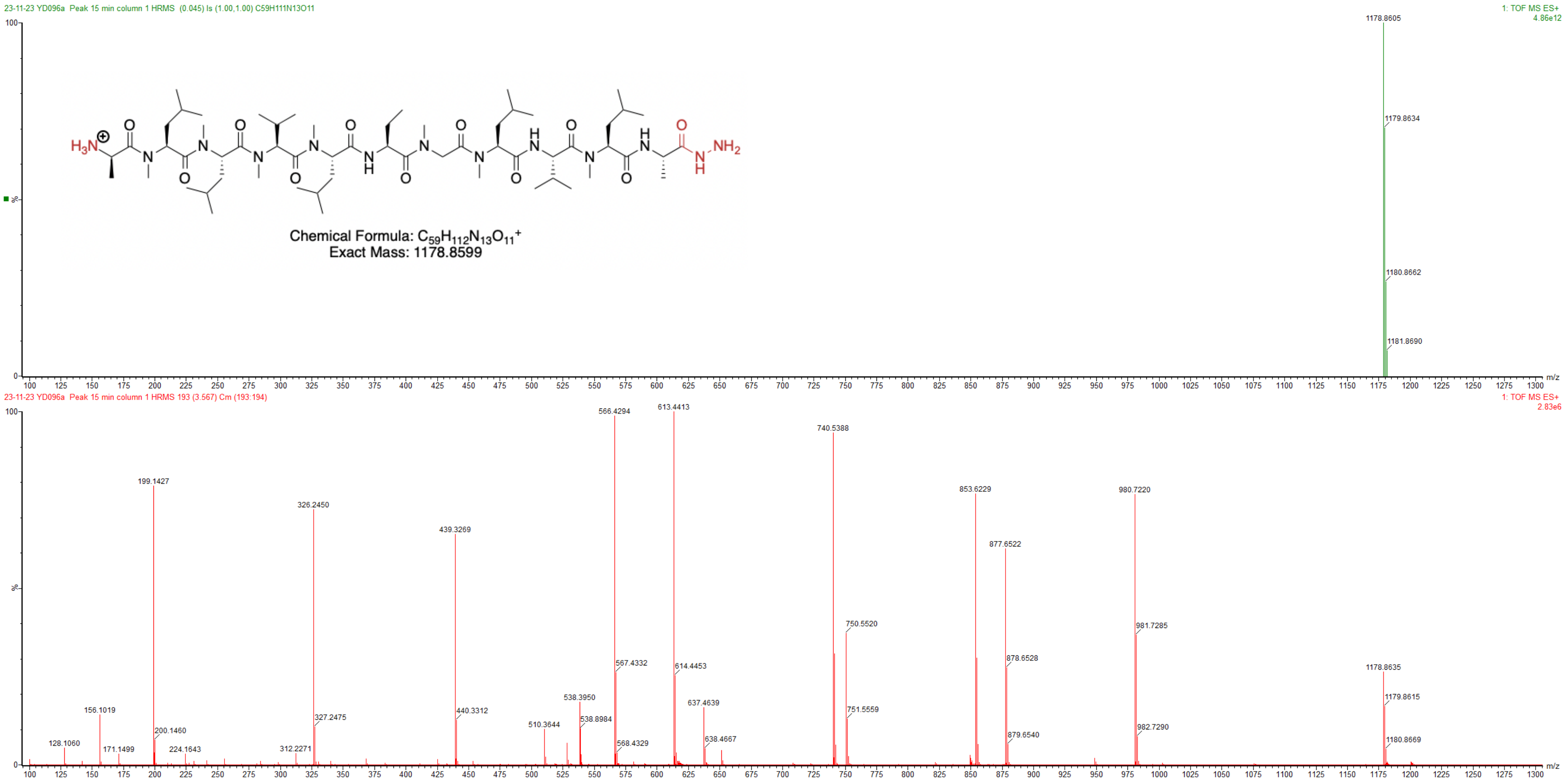

MS^E spectrum for peptide ***31a***

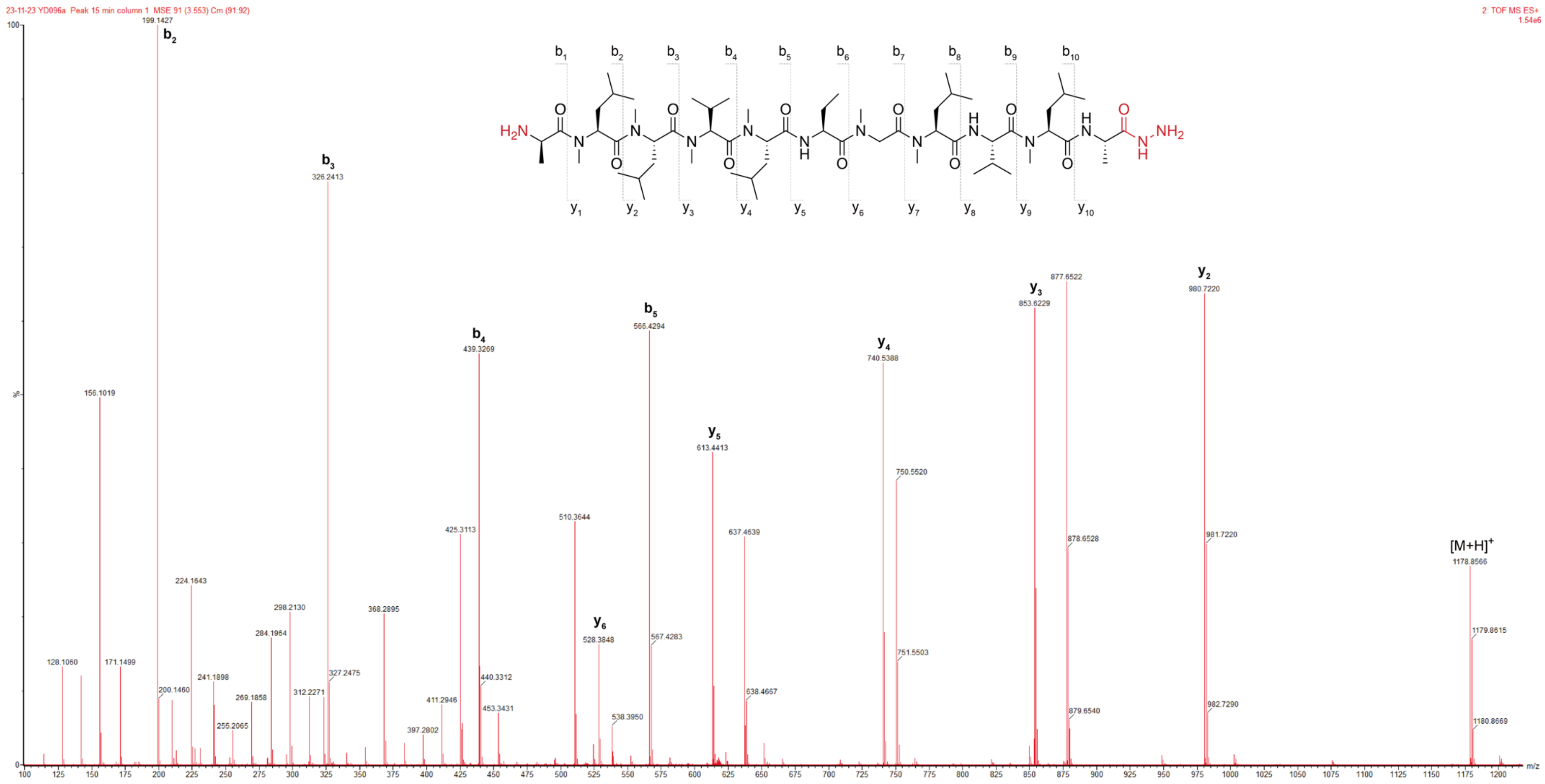

*HRMS spectrum for peptide **31c** (predicted mass spectrum (top) measured (bottom))*

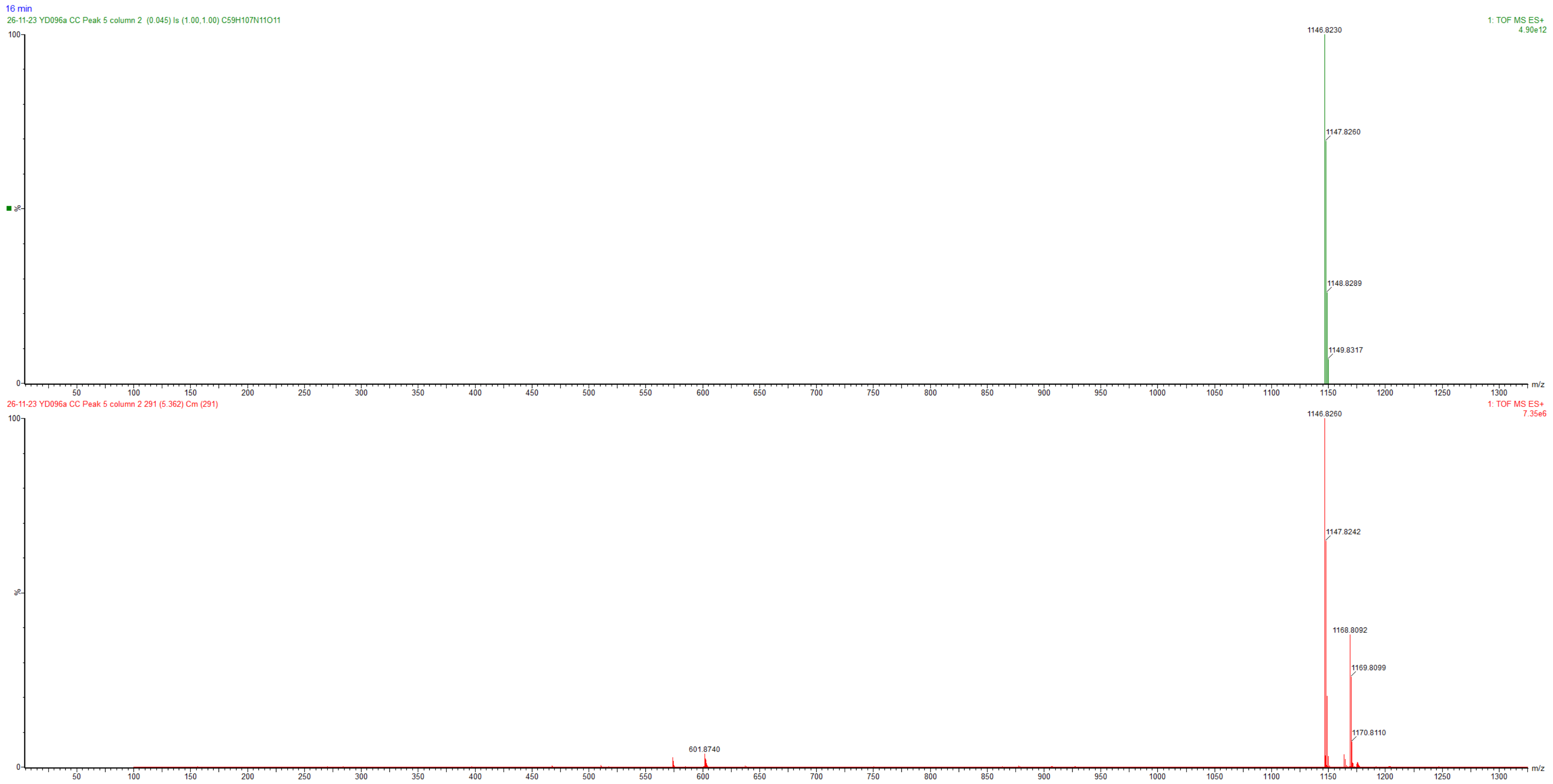

*HRMS spectrum for peptide **32a** (predicted mass spectrum (top) measured (bottom))*

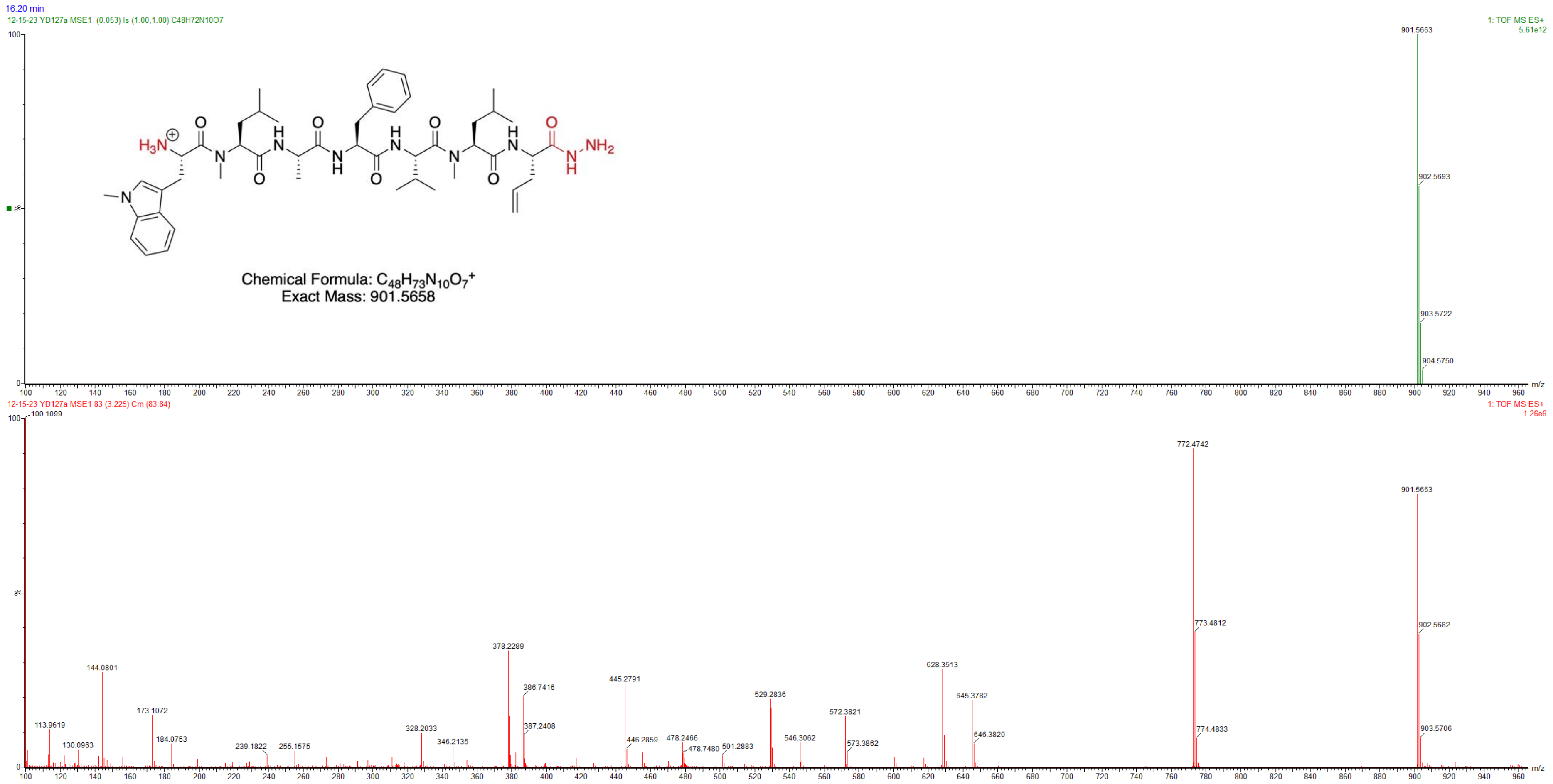

*MS^E spectrum for peptide **32a***

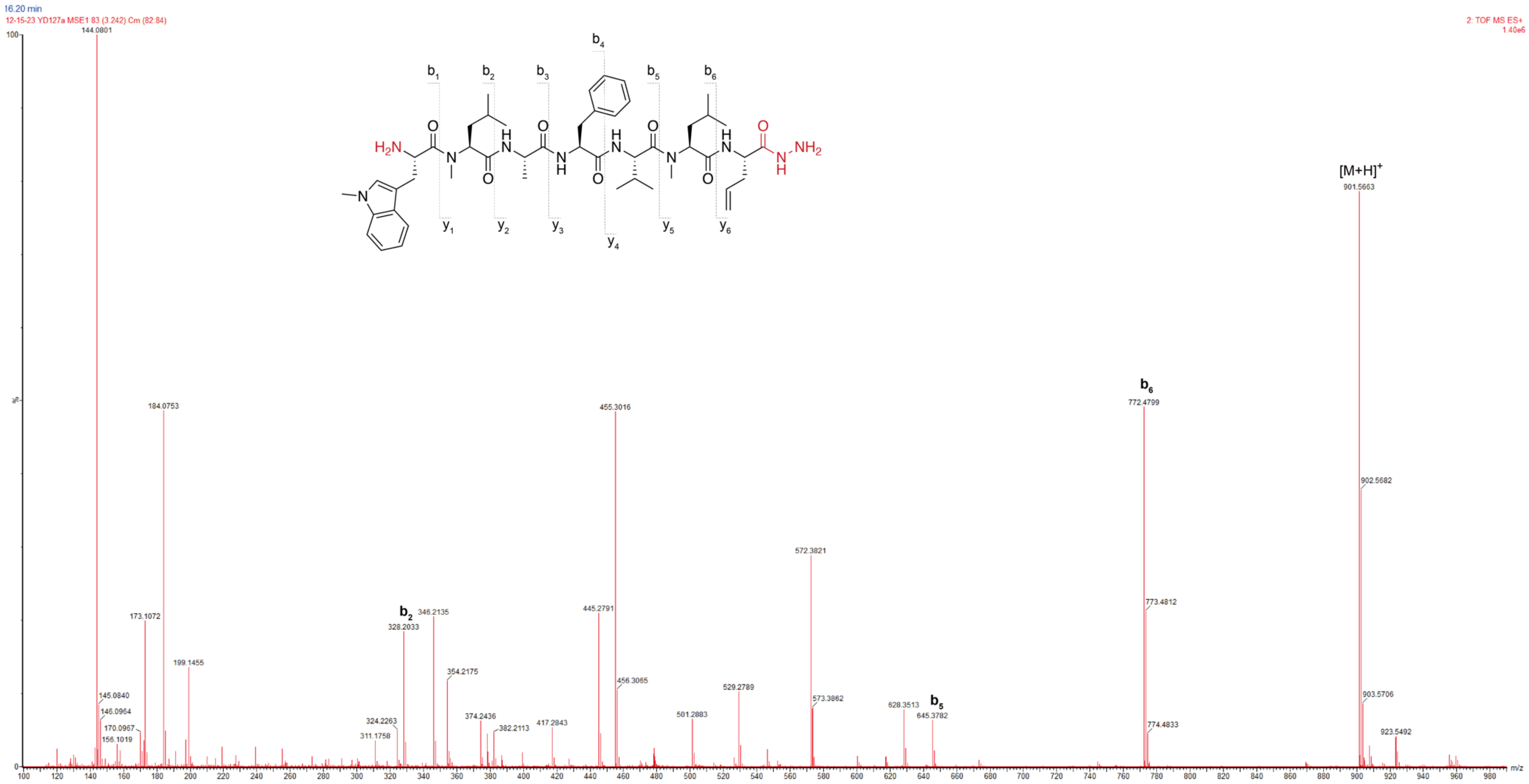

*HRMS spectrum for peptide **32c** (predicted mass spectrum (top) measured (bottom))*

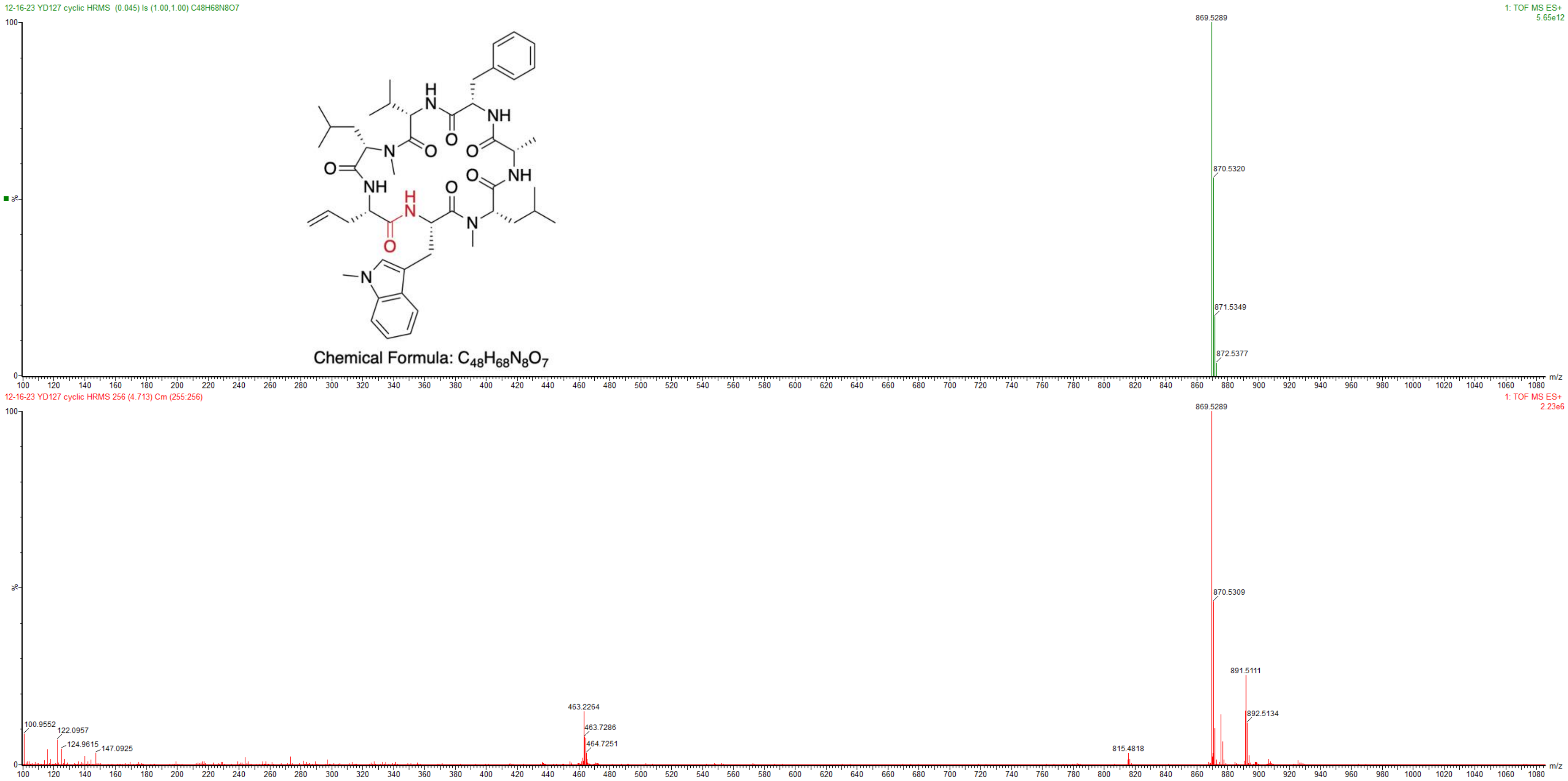

*HRMS spectrum for peptide **33a** (predicted mass spectrum (top and middle) measured (bottom))*

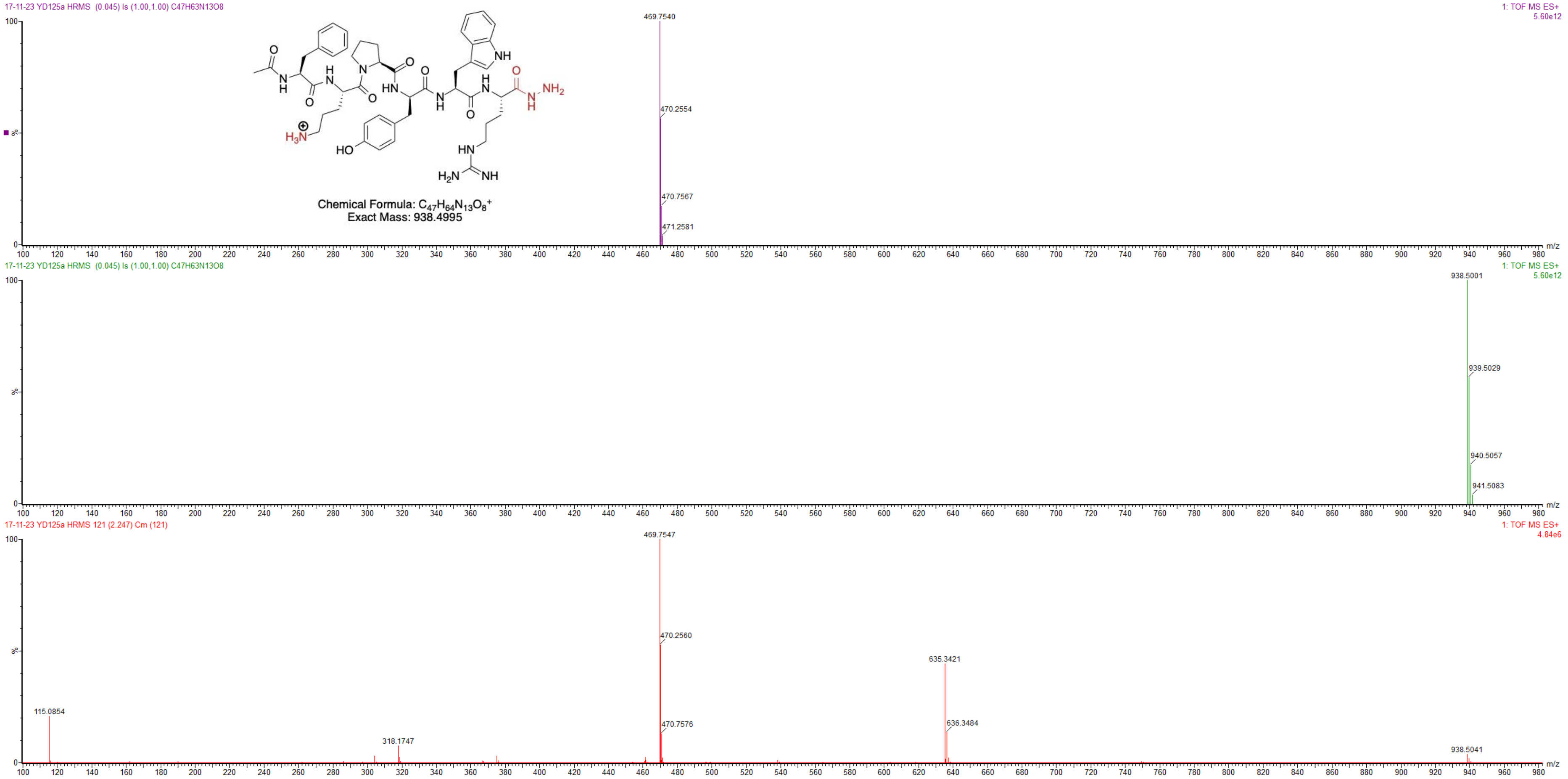

*MS^E spectrum for peptide **33a***

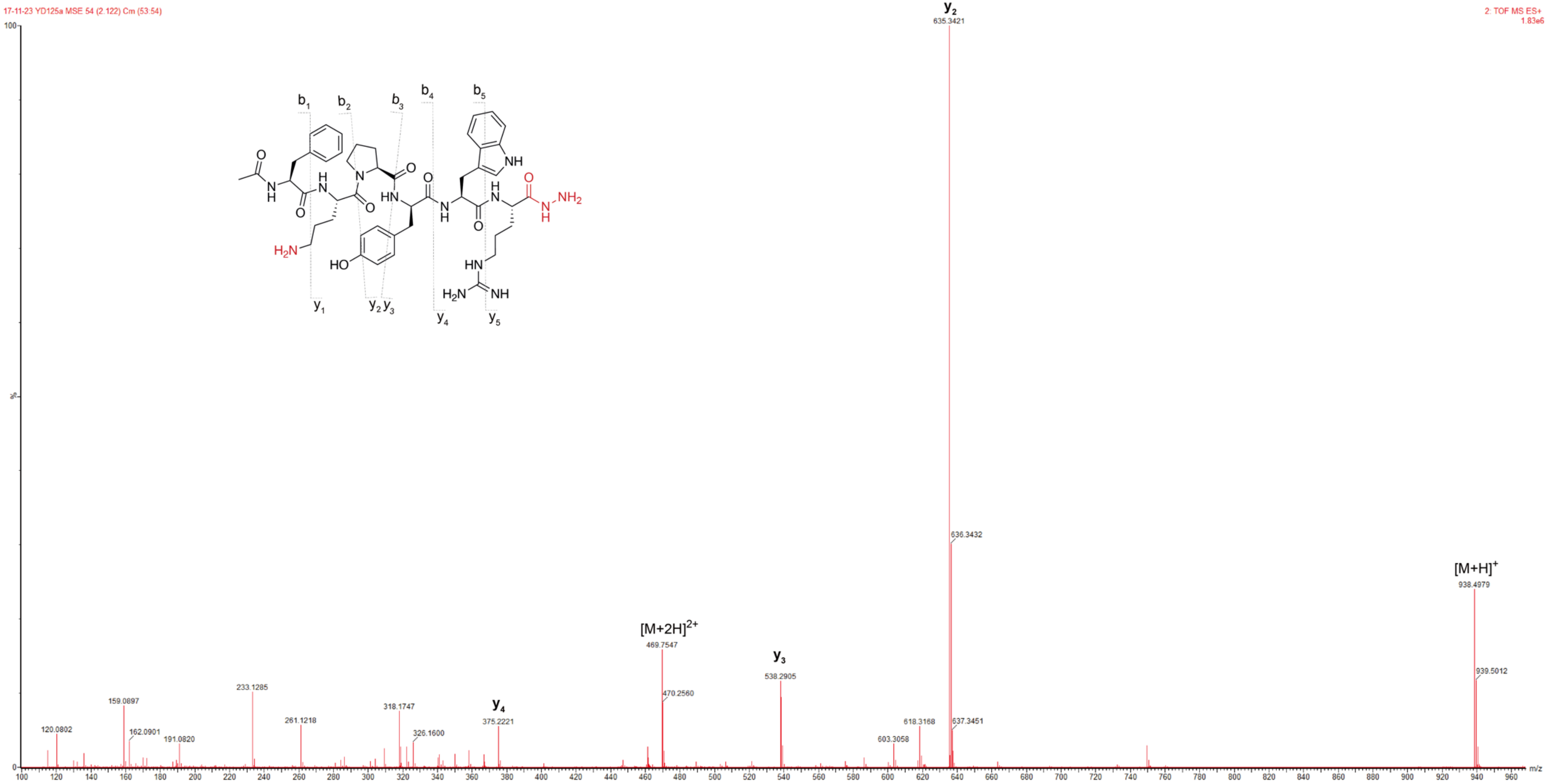

*HRMS spectrum for peptide **33c** (predicted mass spectrum (top and middle) measured (bottom))*

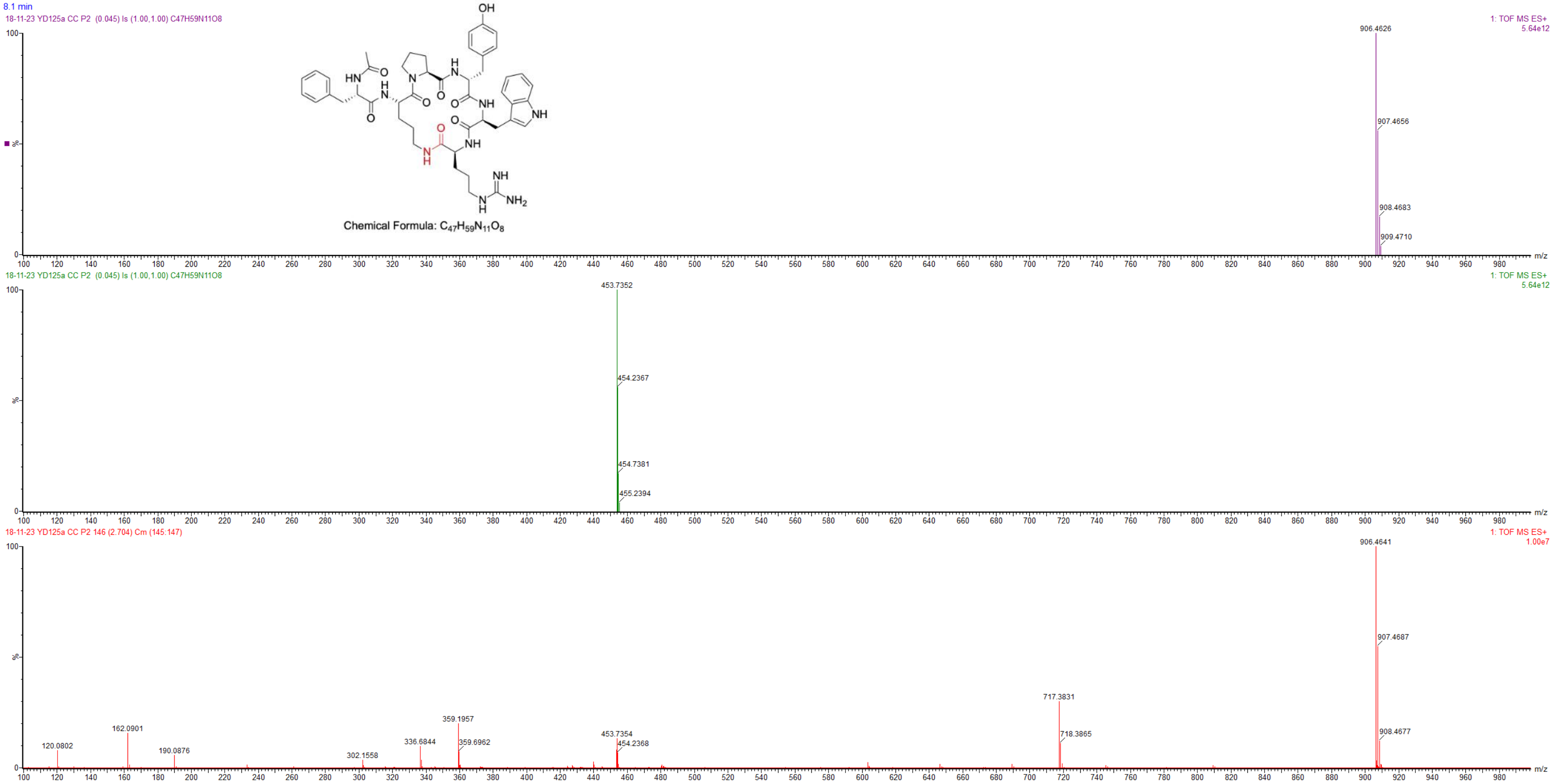

*HRMS spectrum for peptide **30a** (predicted mass spectrum (top) measured (bottom))*

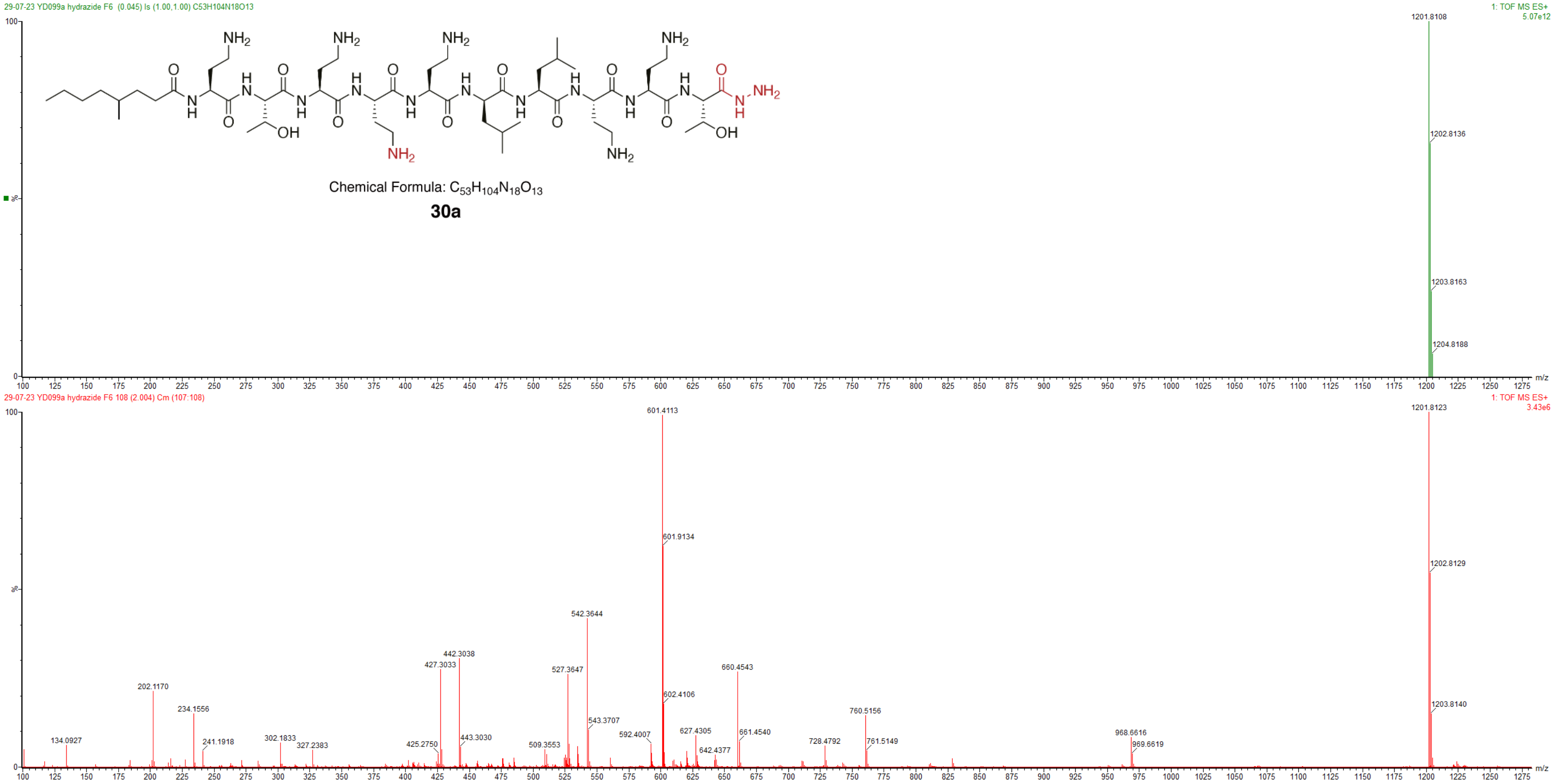

MS^E spectrum for peptide ***30a***

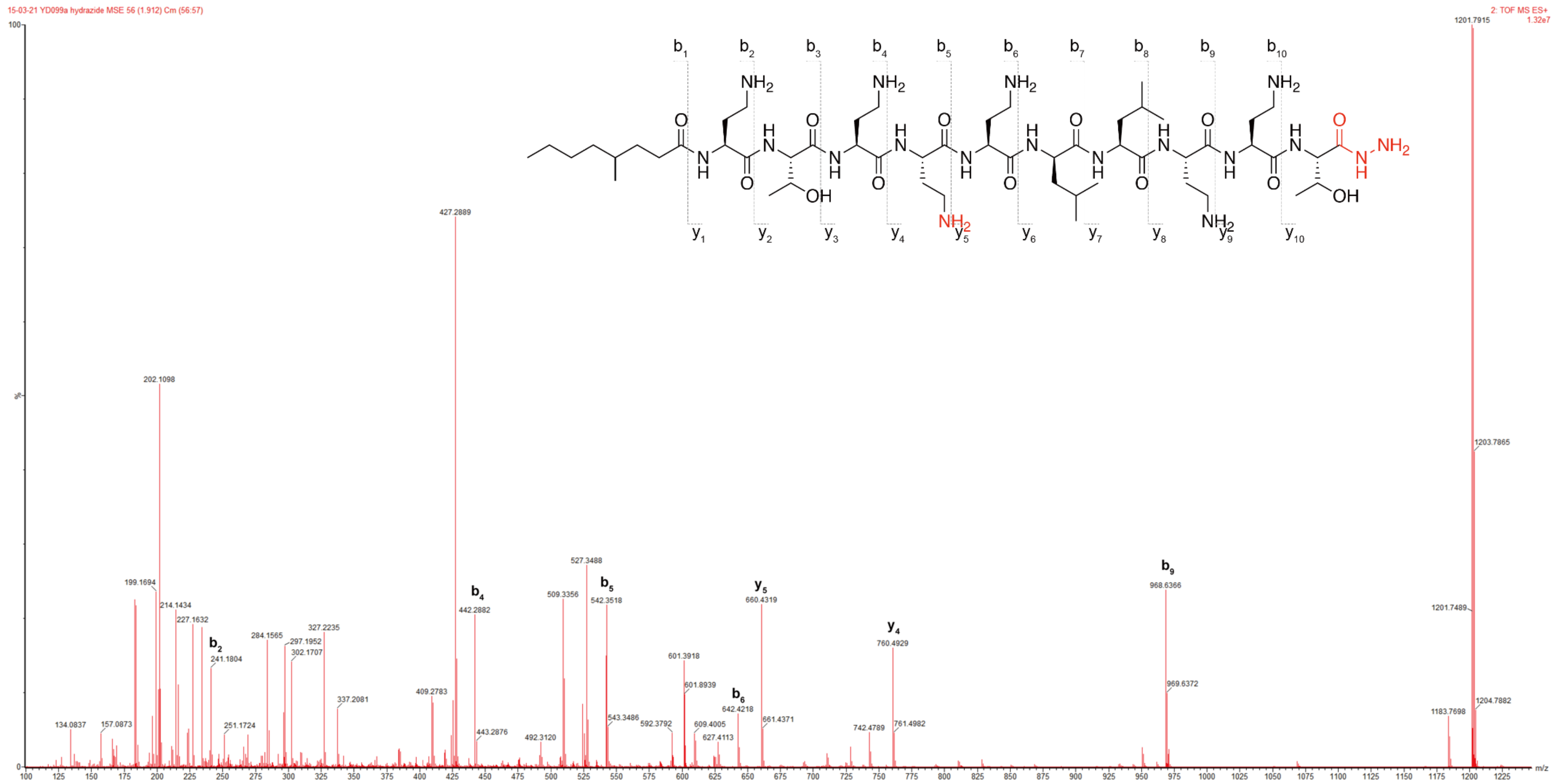

HRMS spectrum for peptide ***30c*** *(predicted mass spectrum (top) measured (bottom))*

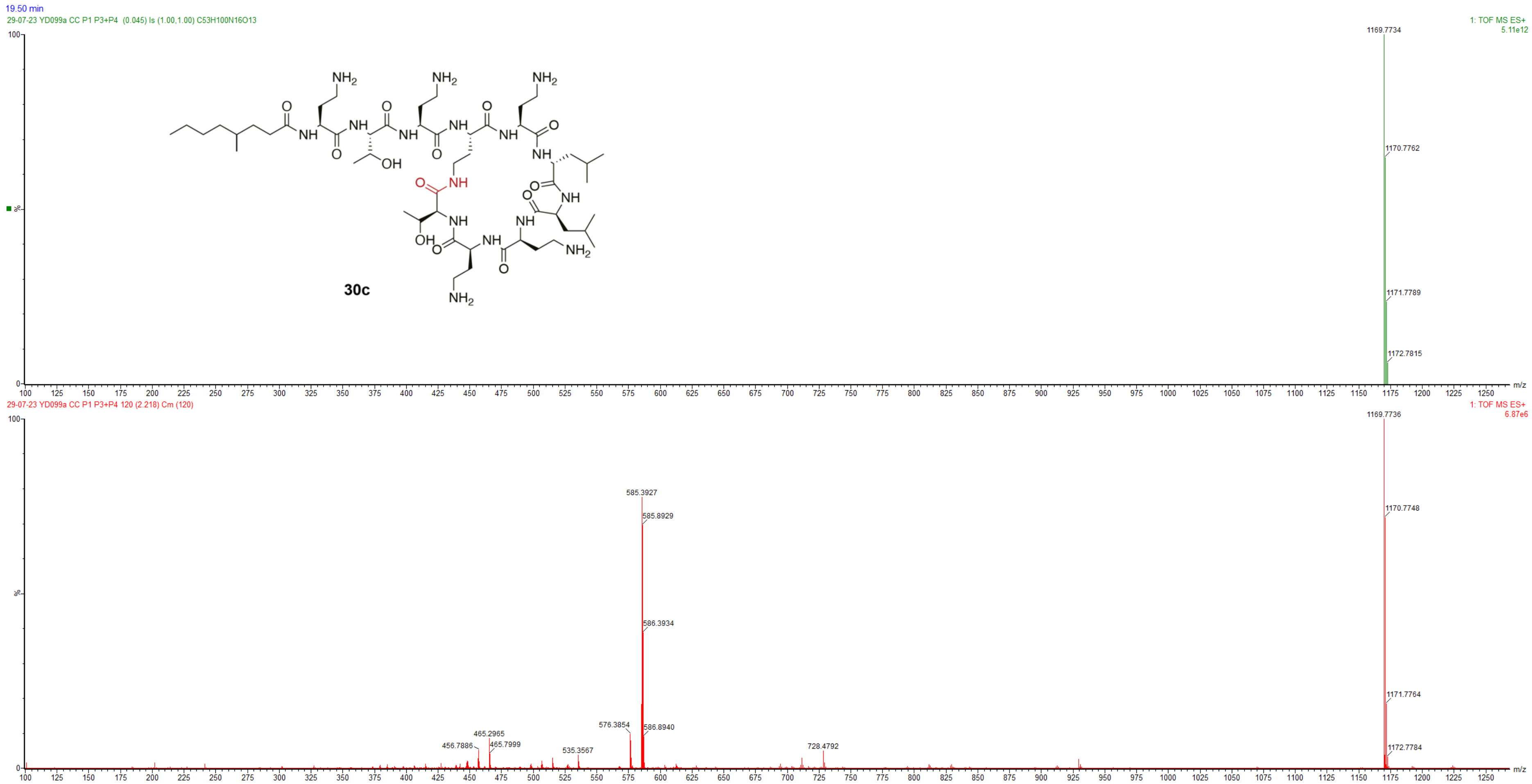

*HRMS spectra for peptide **30c**, **30e**, **30f**, **30g** (commercial colistin).*

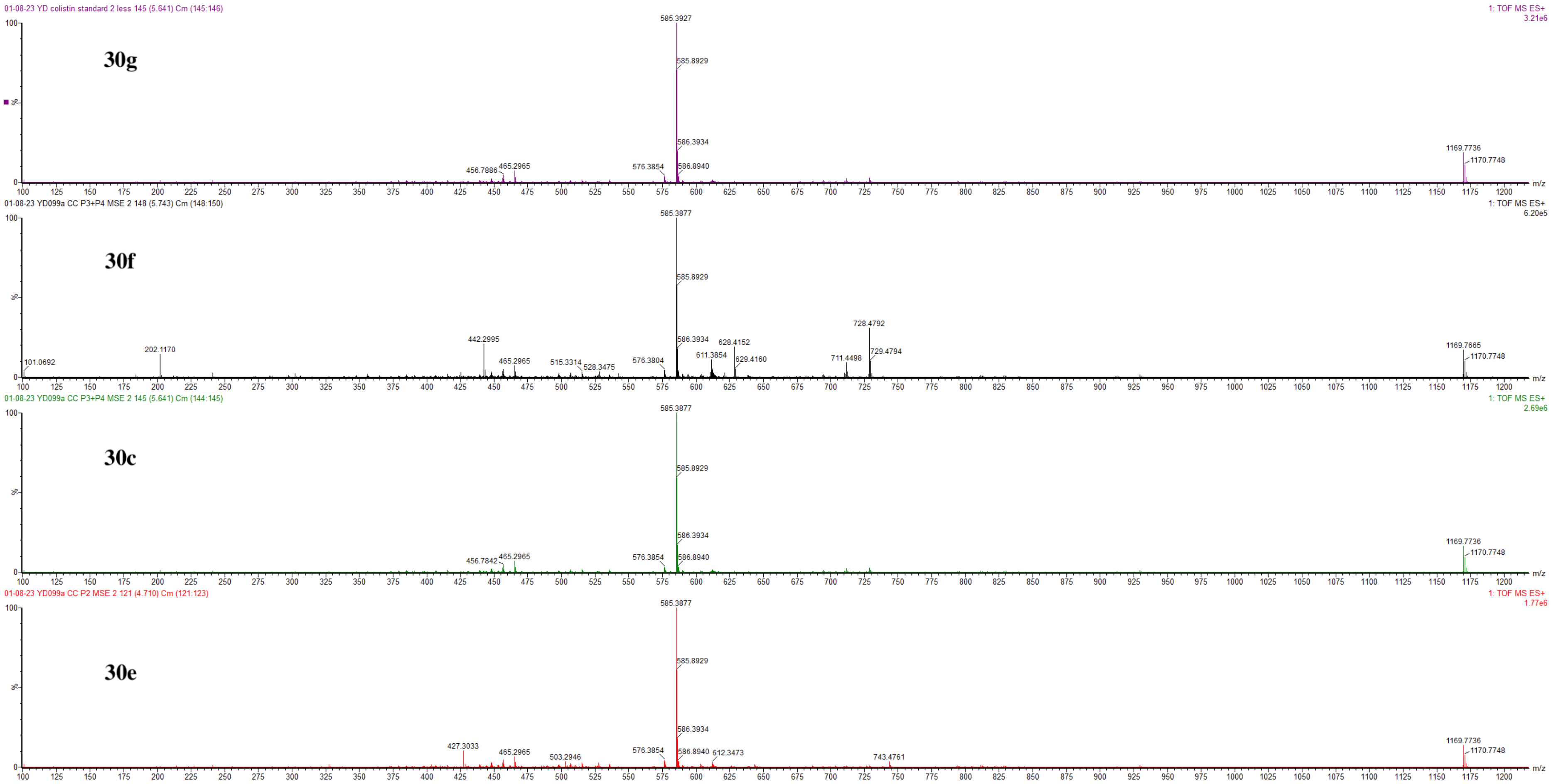

*MS^E spectra for peptide **30c**, **30e**, **30f**, **30g** (commercial colistin).*

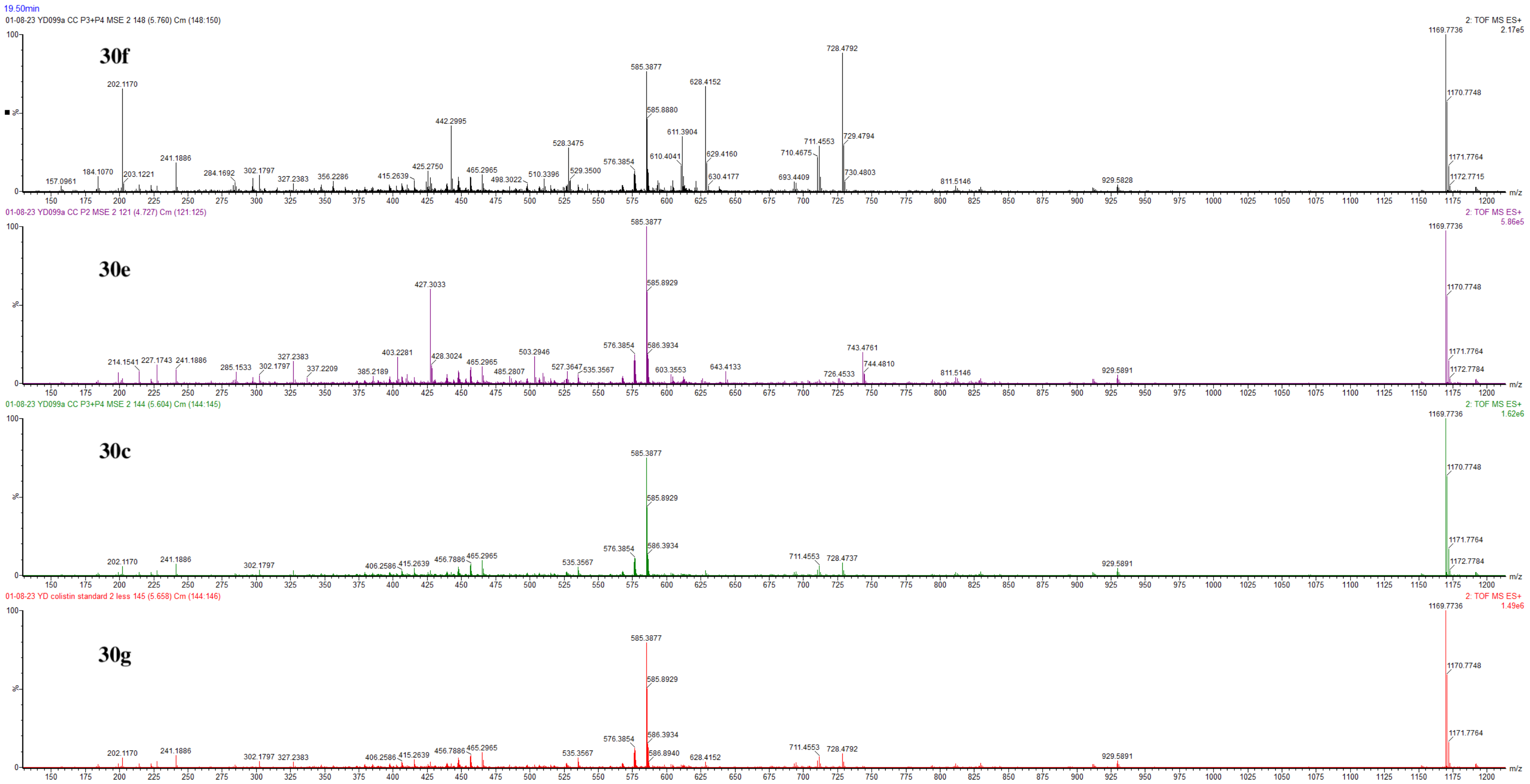

*MS^E spectrum for peptide **30c** (proposed fragments)*

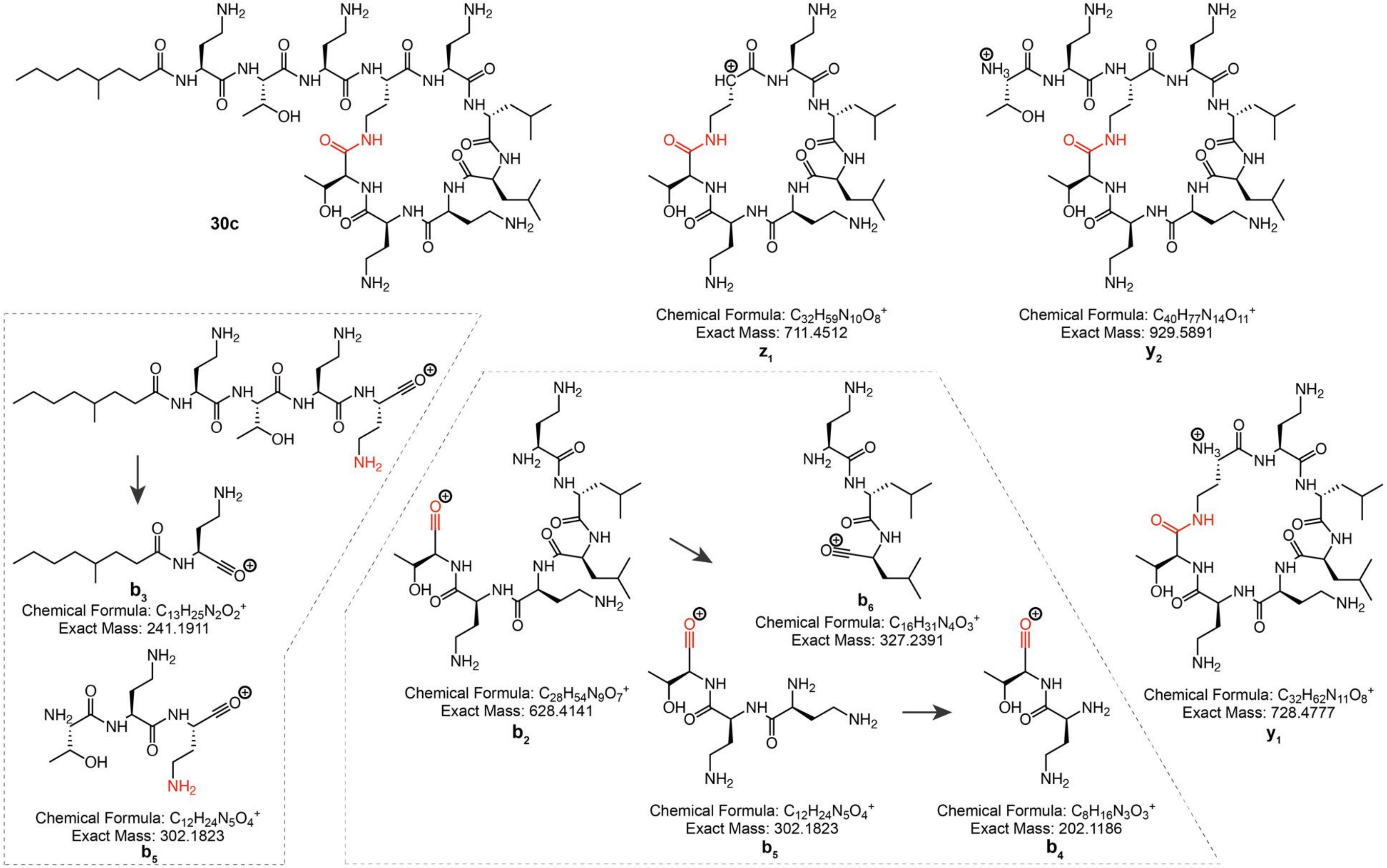

*MS^E spectrum for peptide **30c***

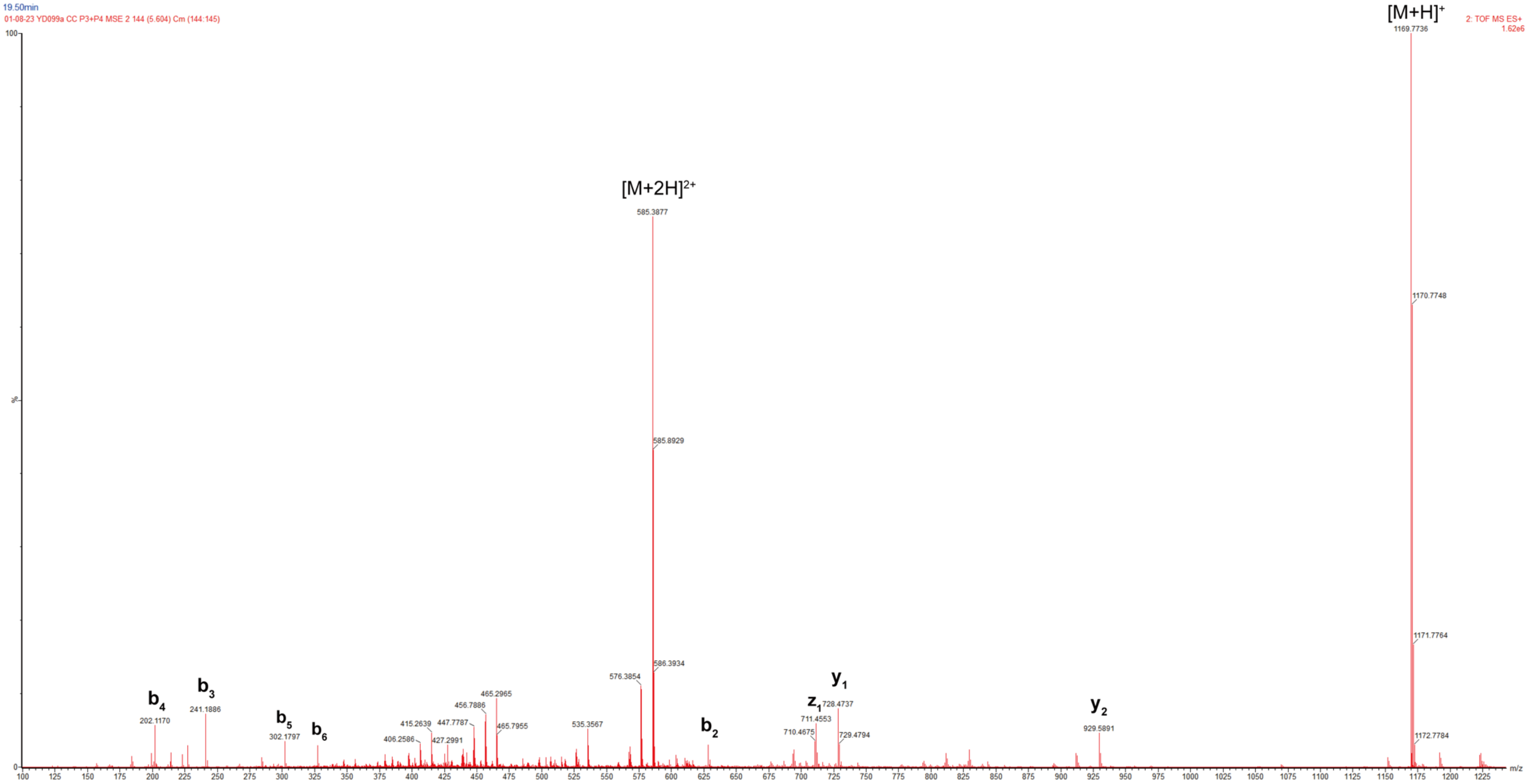

*MS^{E} spectrum for peptide **30f** (proposed side product **30f** and its fragments)*

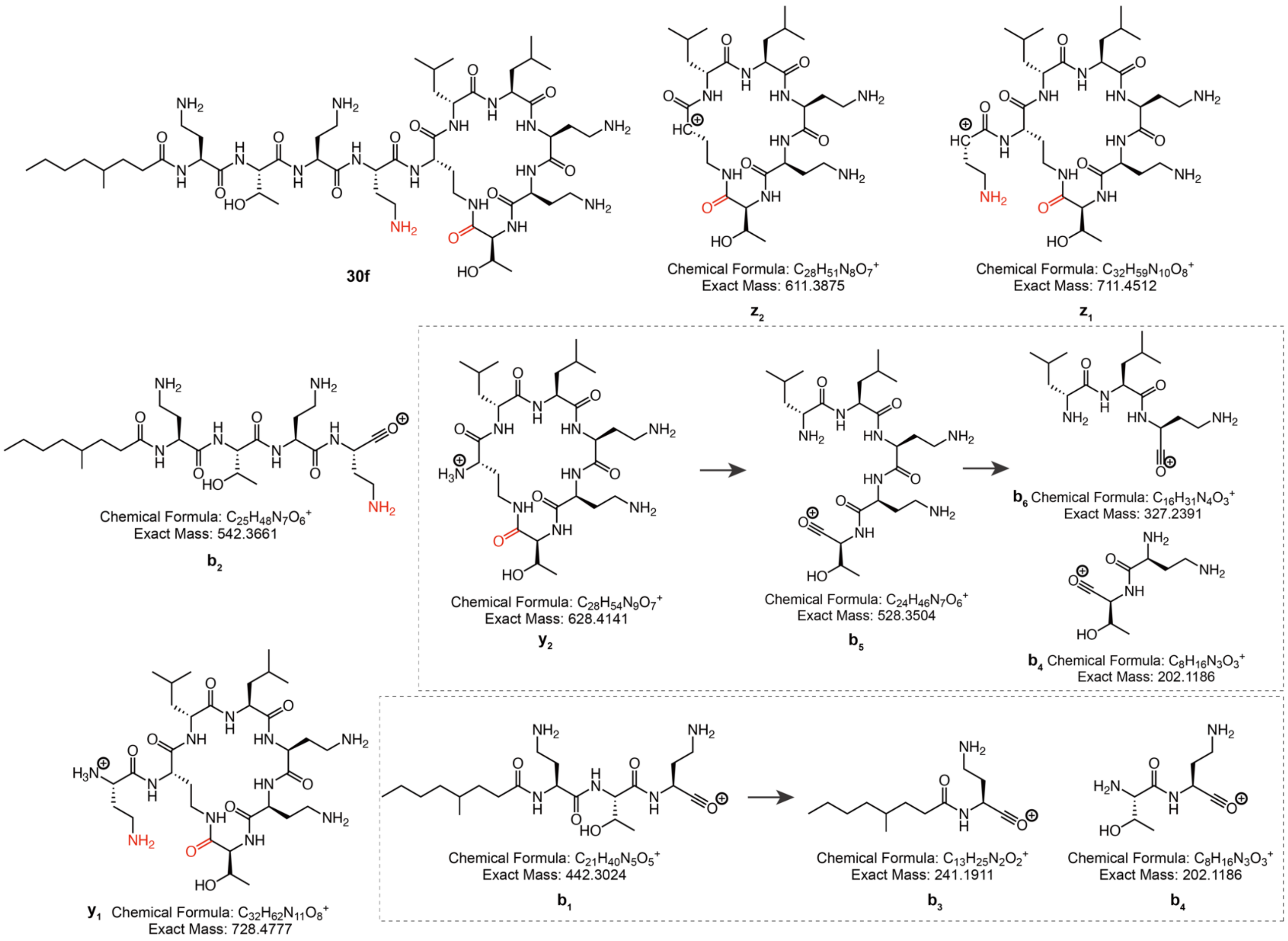

*MS^E spectrum for peptide **30f***

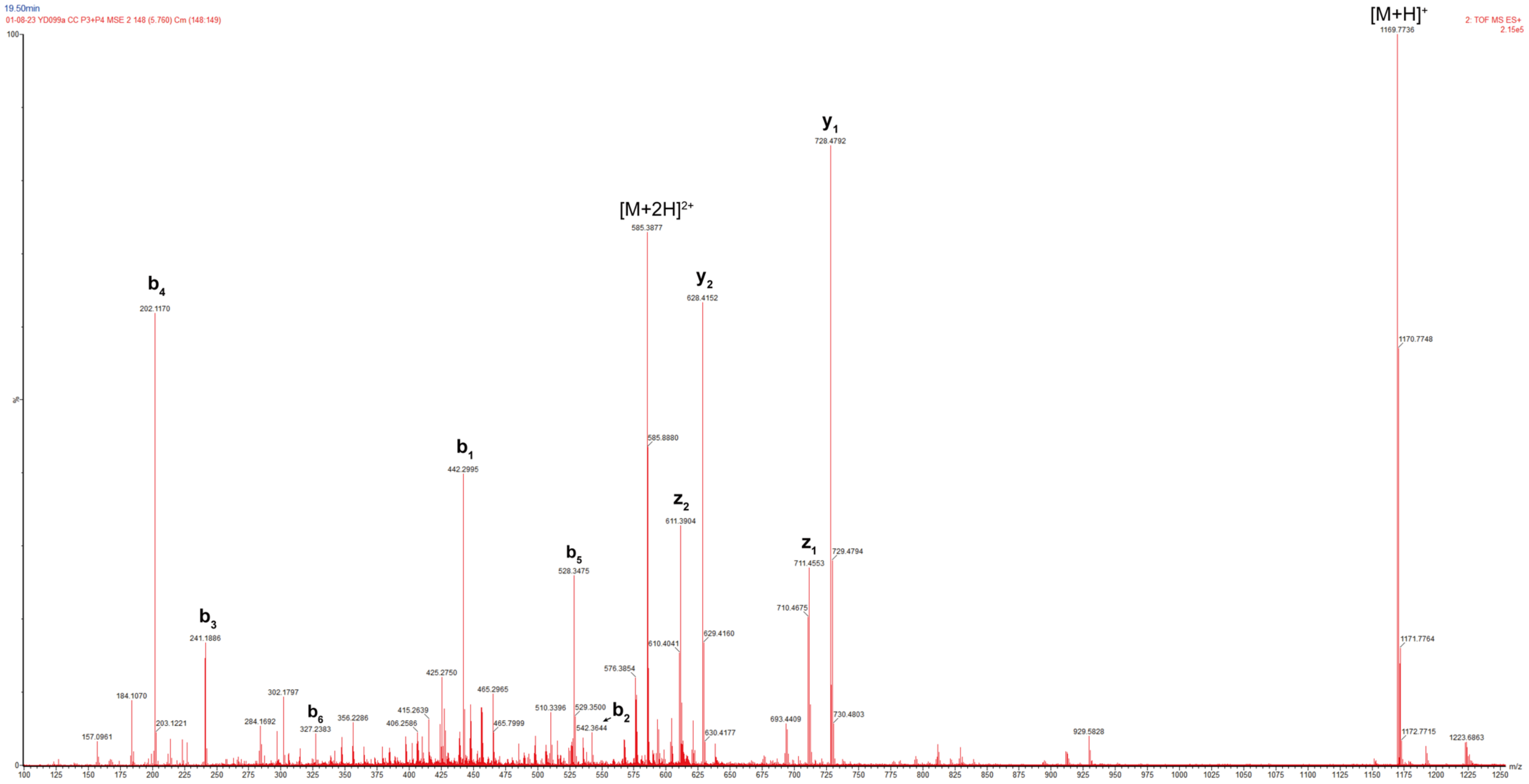

*HRMS spectrum for commercial colistin (Top: **30g** Colistin A (polymyxin E_1), Bottom: Colistin B (polymyxin E_2))*

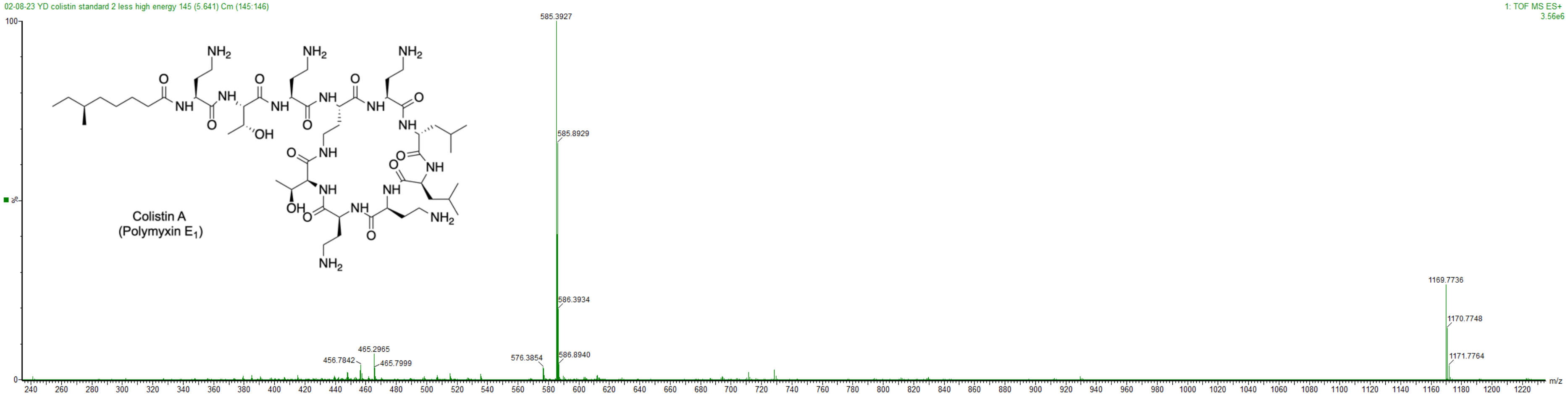

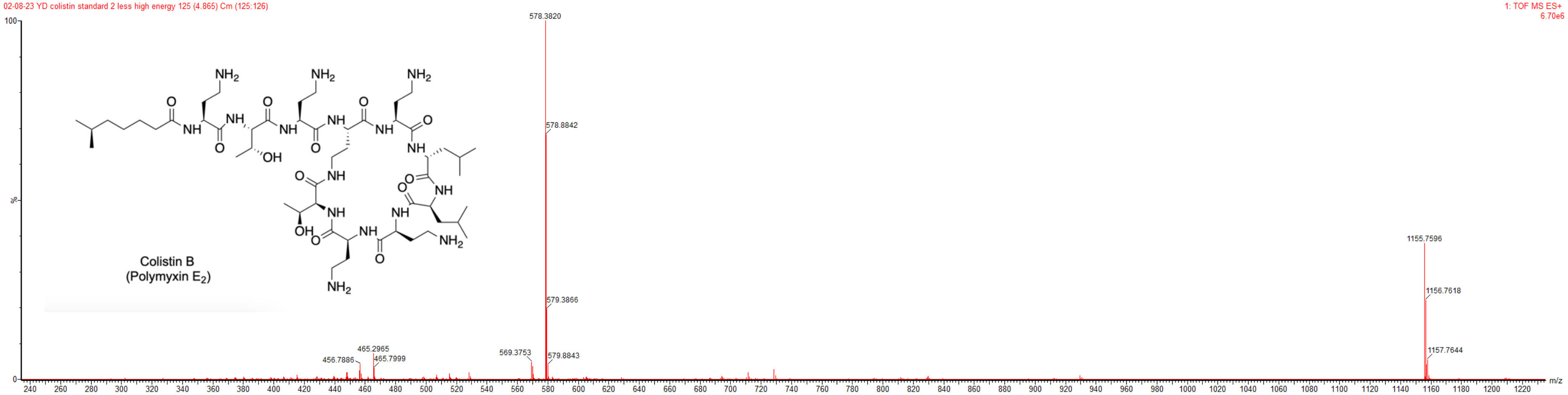

*HRMS spectrum for peptide **34a** (predicted mass spectrum (top) measured (bottom))*

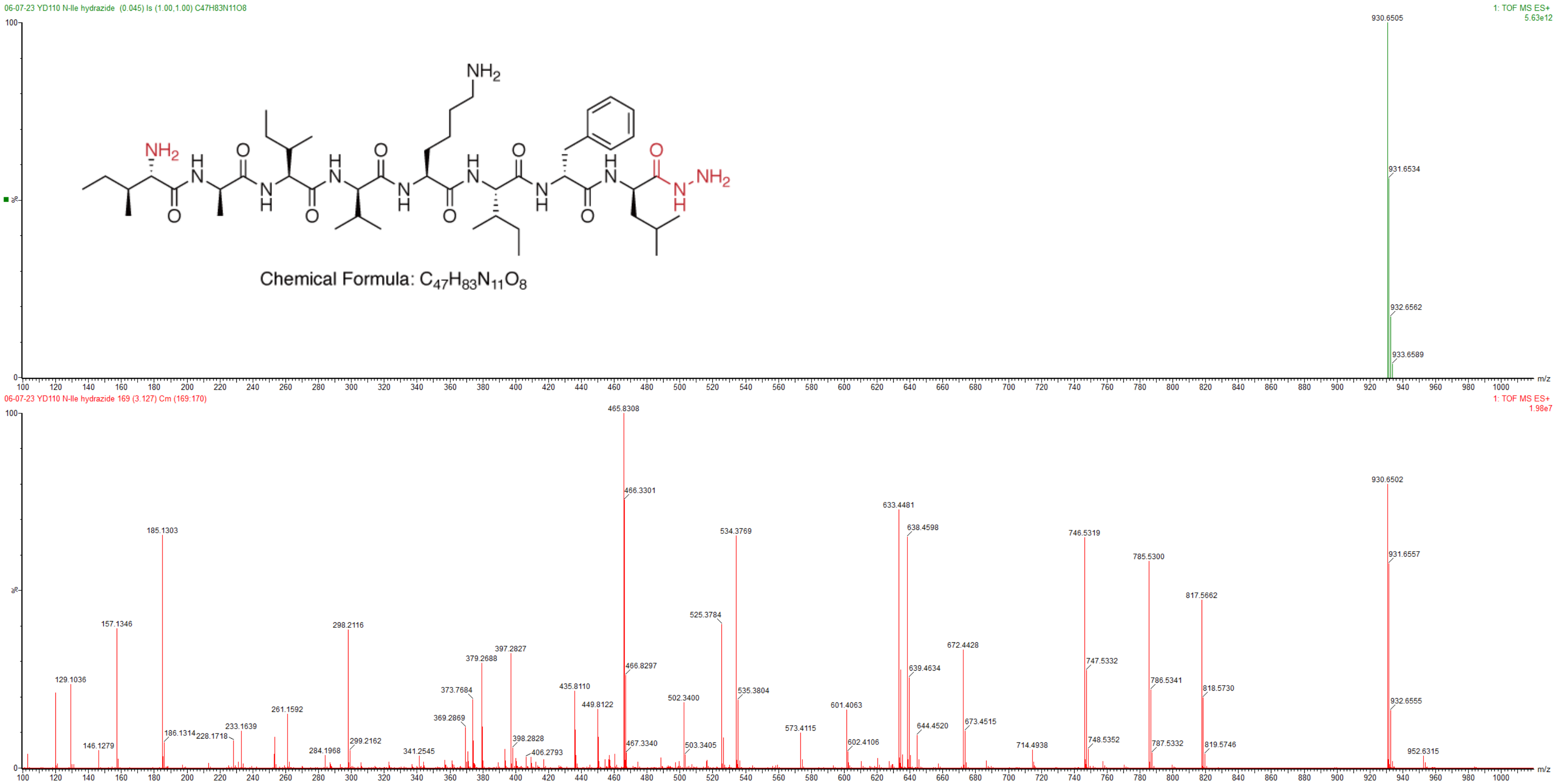

MS^E spectrum for peptide ***34a***

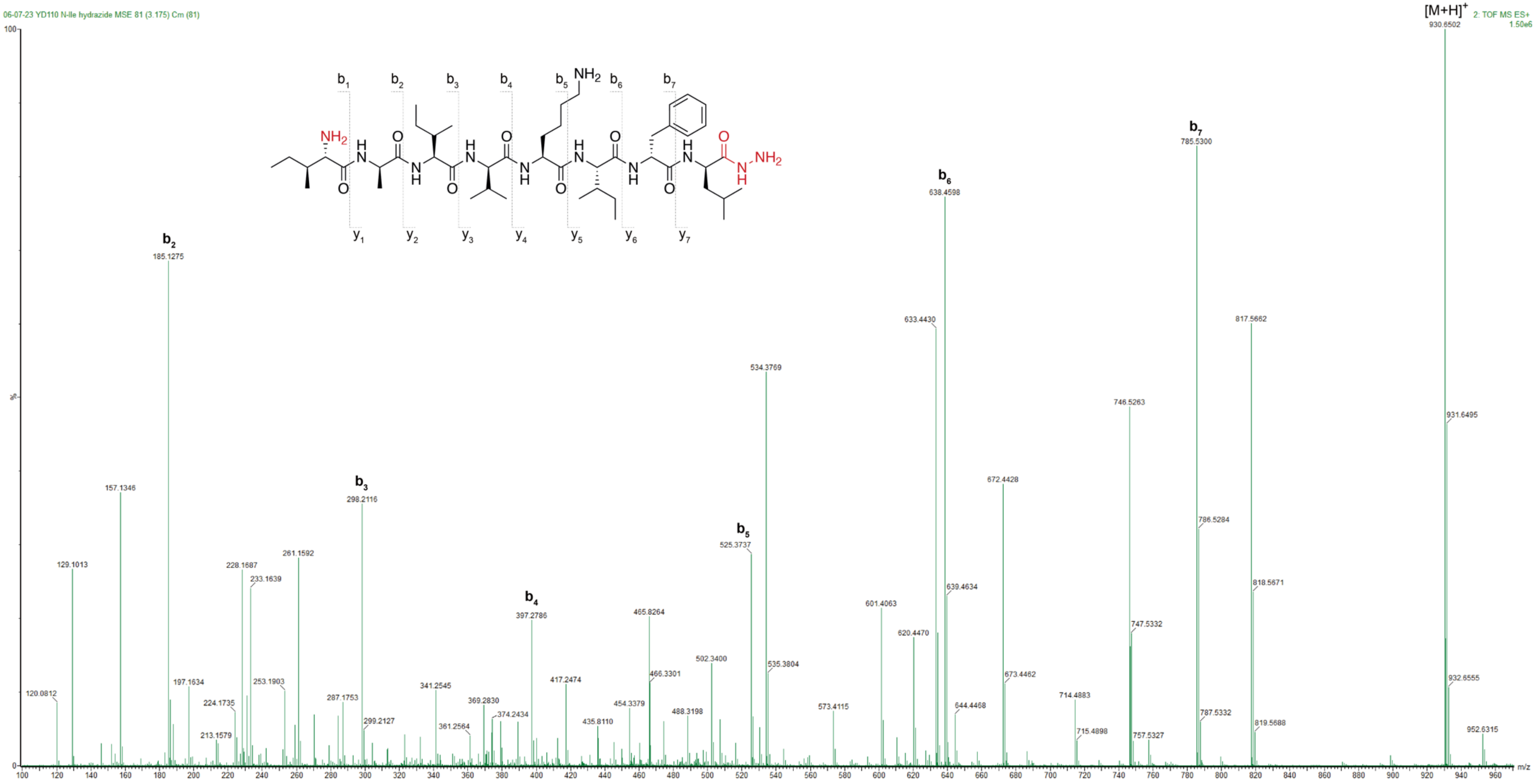

*HRMS spectrum for peptide **34c** (predicted mass spectrum (top) measured (bottom))*

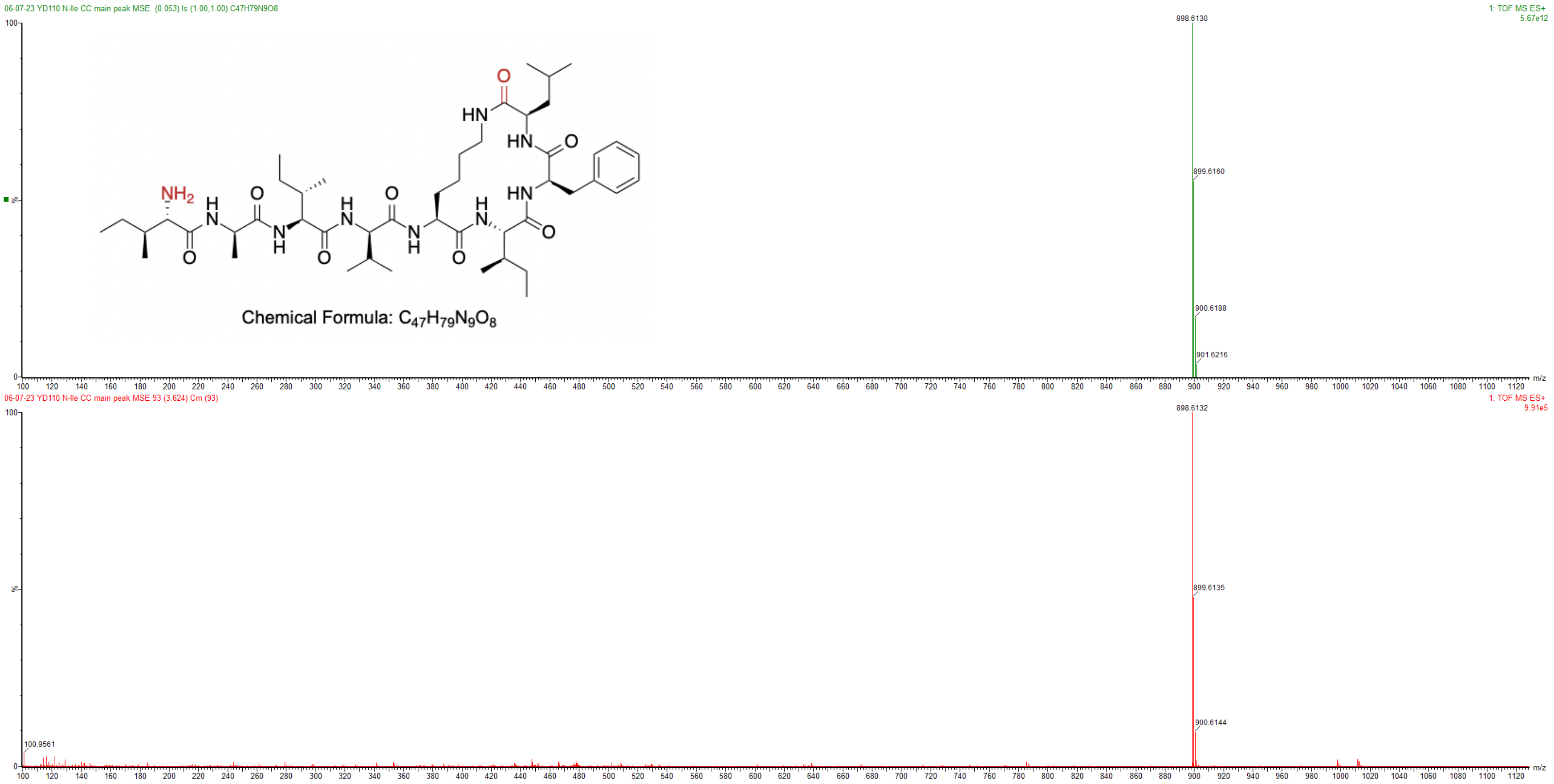

*HRMS spectrum for peptide **Boc-34a** (predicted mass spectrum (top) measured (bottom))*

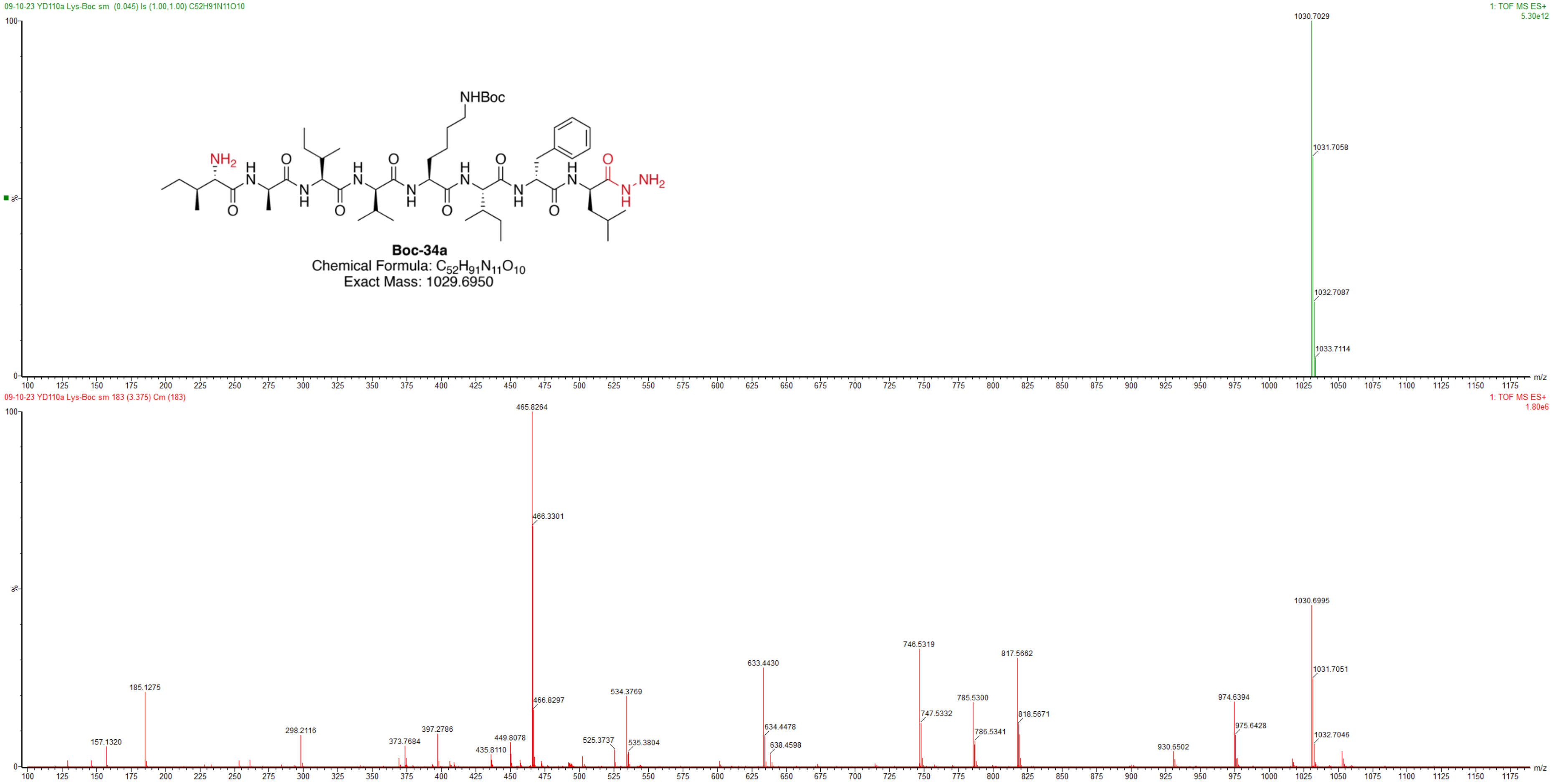

*HRMS spectrum for peptide **35a** (predicted mass spectrum (top) measured (bottom))*

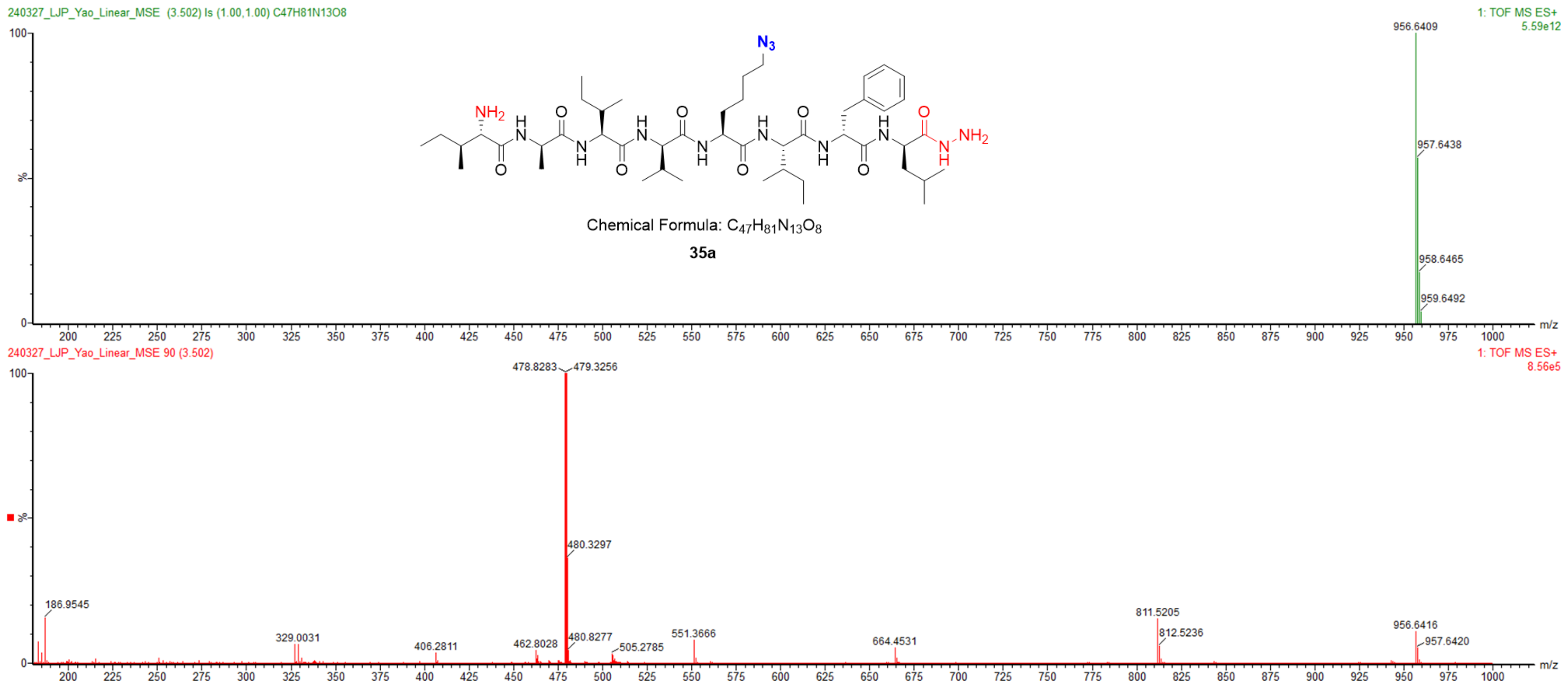

MS^E spectrum for peptide ***35a***

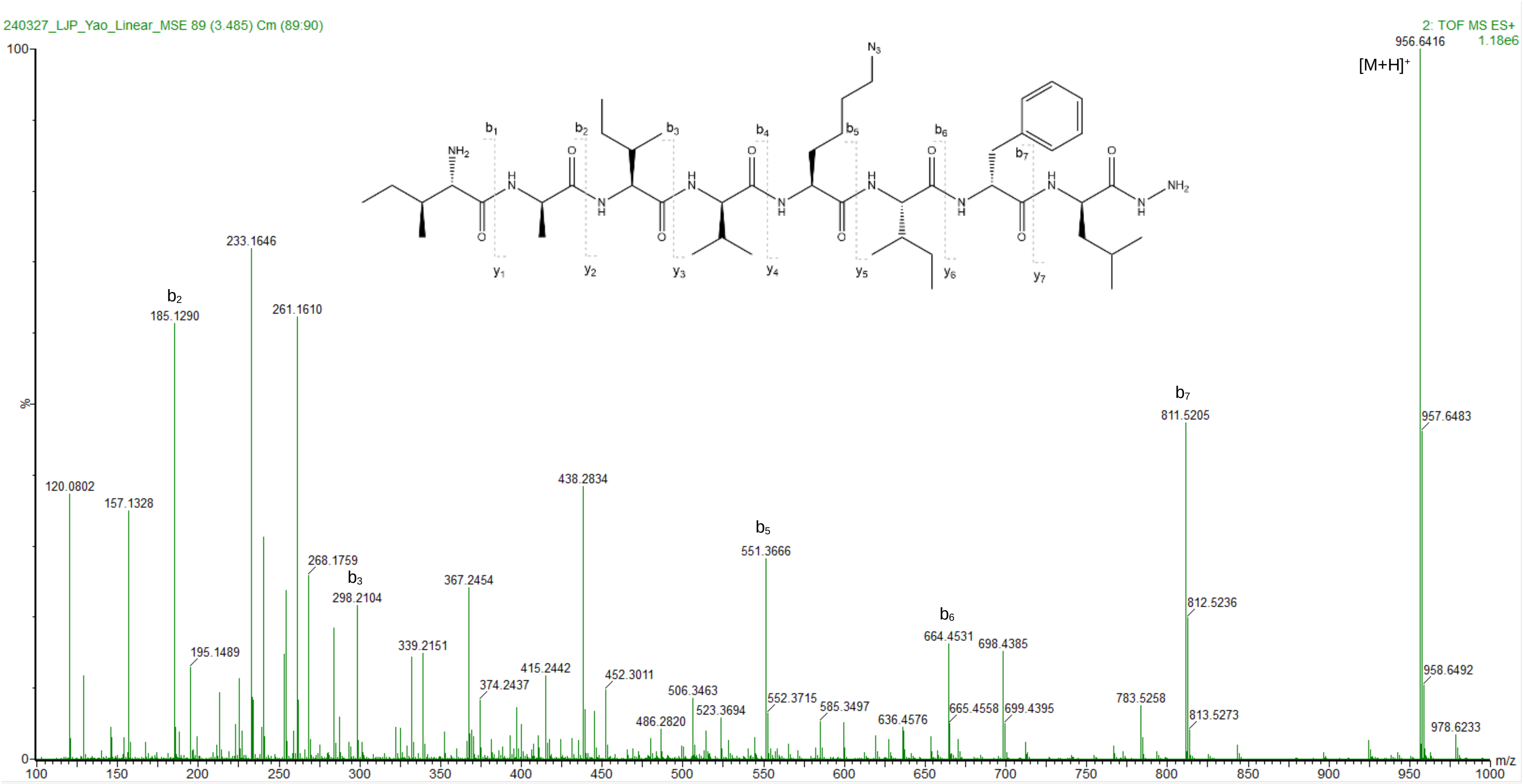

*HRMS spectrum for peptide **35c** (predicted mass spectrum (top) measured (bottom))*

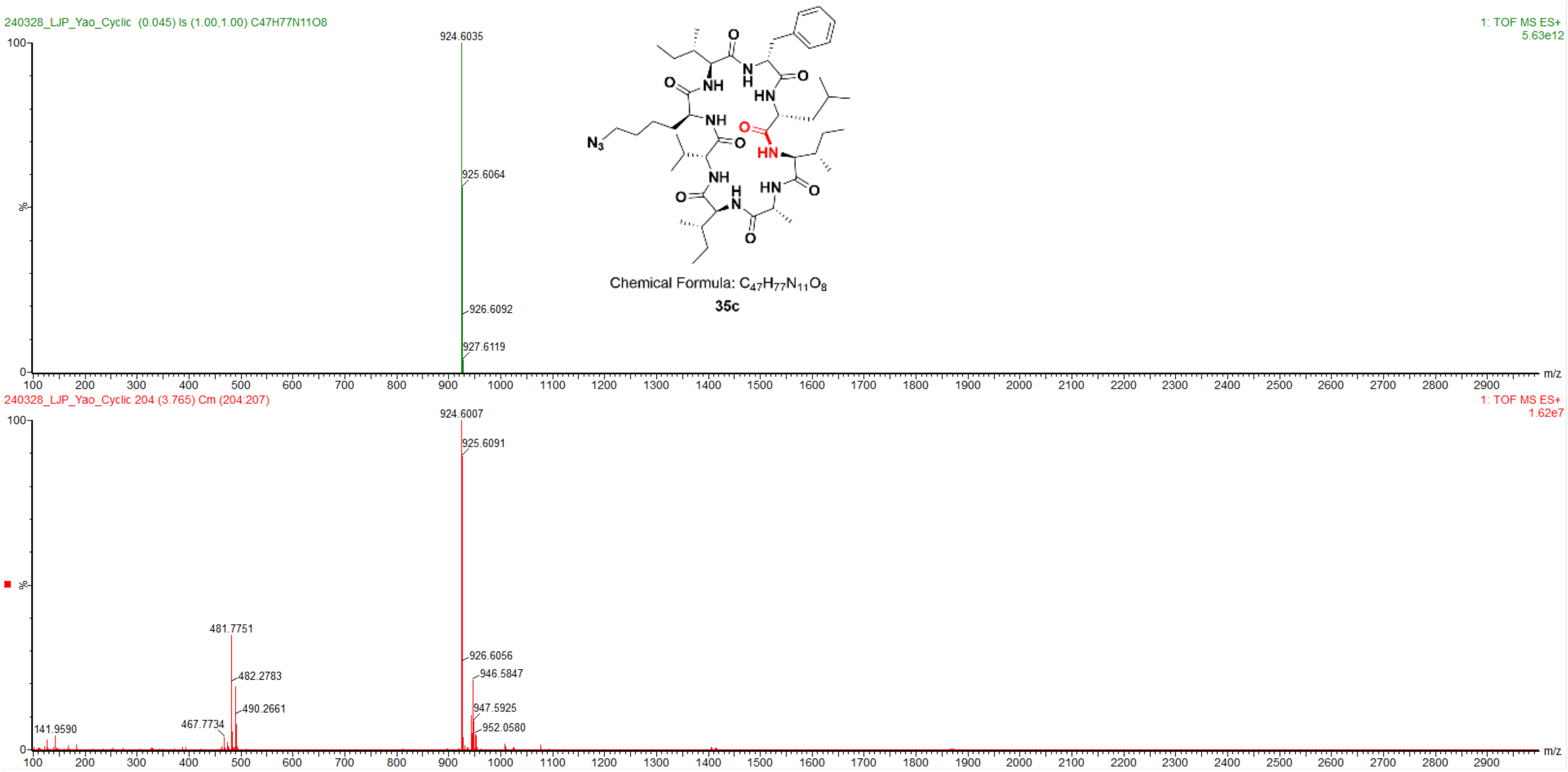

MS^E spectrum for peptide ***35c***

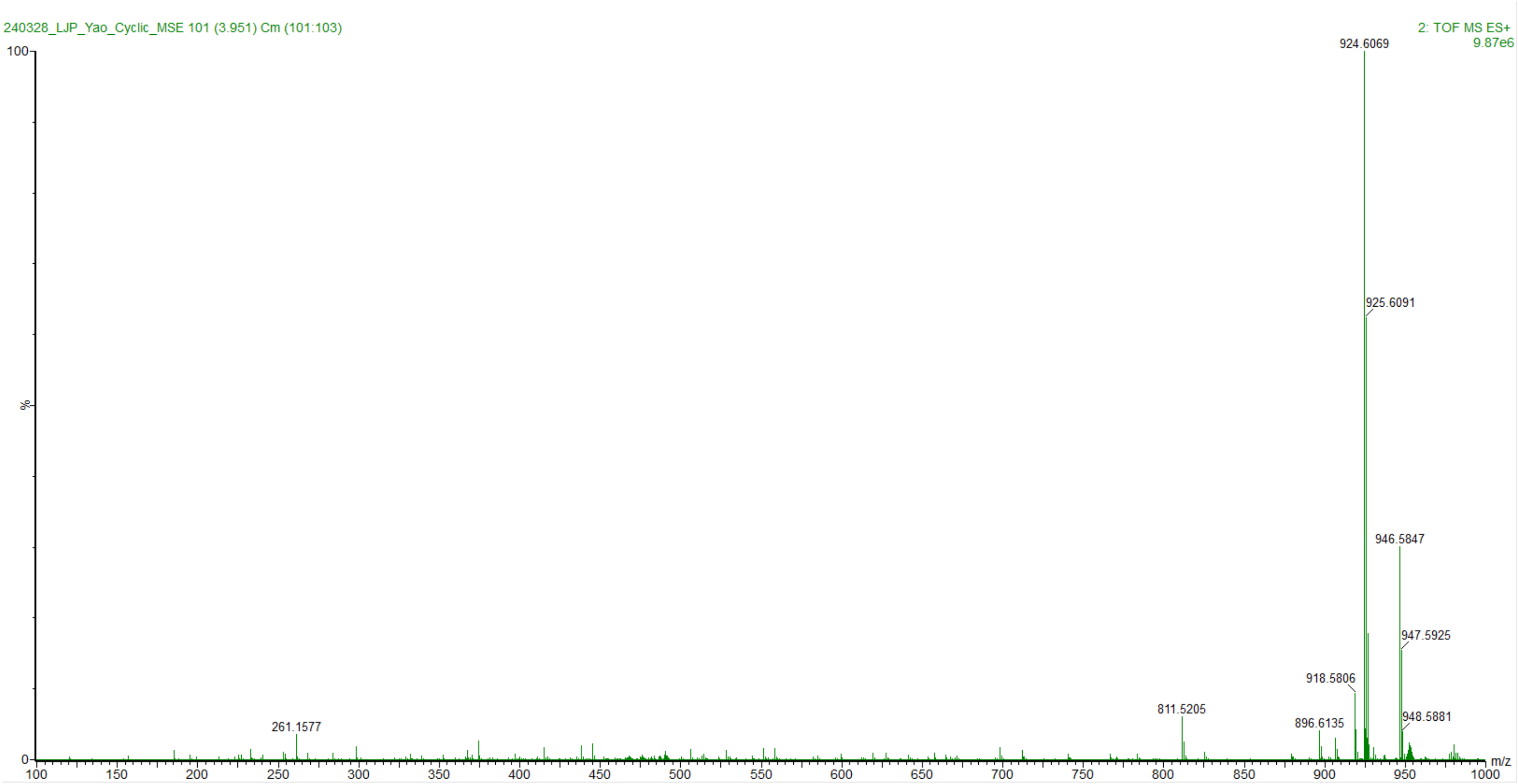

4. Supplementary References

1. Fanelli, R., Jeanne-Julien, L., René, A., Martinez, J. & Cavelier, F. Stereoselective synthesis of unsaturated α-amino acids. *Amino Acids* **47**, 1107–1115 (2015).
2. Stavropoulos, G., Gatos, D., Magafa, V. & Barlos, K. Preparation of polymer-bound trityl-hydrazines and their application in the solid phase synthesis of partially protected peptide hydrazides. *Lett. Pept. Sci.* **2**, 315–318 (1996).
3. *Peptide Synthesis and Applications*. vol. 1047 (Humana Press, Totowa, NJ, 2013).
4. Zheng, J.-S., Tang, S., Qi, Y.-K., Wang, Z.-P. & Liu, L. Chemical synthesis of proteins using peptide hydrazides as thioester surrogates. *Nat. Protoc.* **8**, 2483–2495 (2013).
5. Kaiser, E., Colescott, R. L., Bossinger, C. D. & Cook, P. I. Color test for detection of free terminal amino groups in the solid-phase synthesis of peptides. *Anal. Biochem.* **34**, 595–598 (1970).
6. Vojkovsky, T. Detection of secondary amines on solid phase. *Pept. Res.* **8**, 236–237 (1995).
7. Zhang, L. & Tam, J. P. Lactone and Lactam Library Synthesis by Silver Ion-Assisted Orthogonal Cyclization of Unprotected Peptides. *J. Am. Chem. Soc.* **121**, 3311–3320 (1999).
8. Wishart, D. S. Interpreting protein chemical shift data. *Prog. Nucl. Magn. Reson. Spectrosc.* **58**, 62–87 (2011).
9. O'Boyle, N. M. *et al.* Open Babel: An open chemical toolbox. *J. Cheminformatics* **3**, 33 (2011).
10. Vanommeslaeghe, K. *et al.* CHARMM general force field: A force field for drug-like molecules compatible with the CHARMM all-atom additive biological force fields. *J. Comput. Chem.* NA-NA (2009) doi:10.1002/jcc.21367.
11. Smith, A. K., Wilkerson, J. W. & Knotts, T. A. Parameterization of Unnatural Amino Acids with Azido and Alkynyl R-Groups for Use in Molecular Simulations. *J. Phys. Chem. A* **124**, 6246–6253 (2020).
12. Abraham, M. J. *et al.* GROMACS: High performance molecular simulations through multi-level parallelism from laptops to supercomputers. *SoftwareX* **1–2**, 19–25 (2015).
13. Essmann, U. *et al.* A smooth particle mesh Ewald method. *J. Chem. Phys.* **103**, 8577–8593 (1995).

14. Nosé, S. A unified formulation of the constant temperature molecular dynamics methods. *J. Chem. Phys.* **81**, 511–519 (1984).

15. Hoover, W. G. Canonical dynamics: Equilibrium phase-space distributions. *Phys Rev A* **31**, 1695–1697 (1985).

16. Parrinello, M. & Rahman, A. Polymorphic transitions in single crystals: A new molecular dynamics method. *J. Appl. Phys.* **52**, 7182–7190 (1981).

17. King's College London e-Research team. King's Computational Research, Engineering and Technology Environment (CREATE). (2022) doi:10.18742/RNVF-M076.

18. Michaud-Agrawal, N., Denning, E. J., Woolf, T. B. & Beckstein, O. MDAnalysis: A toolkit for the analysis of molecular dynamics simulations. *J. Comput. Chem.* **32**, 2319–2327 (2011).

19. Smith, P., Ziolek, R. M., Gazzarrini, E., Owen, D. M. & Lorenz, C. D. On the interaction of hyaluronic acid with synovial fluid lipid membranes. *Phys. Chem. Chem. Phys.* **21**, 9845–9857 (2019).

20. DeLano, W. L. Pymol: An open-source molecular graphics tool. *CCP4 Newsl Protein Crystallogr* **40**, 82–92 (2002).

21. Tomita, H., Katsuyama, Y., Minami, H. & Ohnishi, Y. Identification and characterization of a bacterial cytochrome P450 monooxygenase catalyzing the 3-nitration of tyrosine in rufomycin biosynthesis. *J. Biol. Chem.* **292**, 15859–15869 (2017).

22. Ma, J. *et al.* Biosynthesis of ilamycins featuring unusual building blocks and engineered production of enhanced anti-tuberculosis agents. *Nat. Commun.* **8**, 391 (2017).

23. Perez Ortiz, G.; Sidda, J. D.; de los Santos, E. L. C.; Hubert, C. B.; Barry, S. M. *In Vitro* Elucidation of the Crucial but Complex Oxidative Tailoring Steps in Rufomycin Biosynthesis Enables One Pot Conversion of Rufomycin B to Rufomycin C. *Chem. Commun.*, **57** (89), 11795–11798 (2021).
